# Analysis of 1,000 Type-Strain Genomes Improves Taxonomic Classification of *Bacteroidetes*

**DOI:** 10.3389/fmicb.2019.02083

**Published:** 2019-09-23

**Authors:** Marina García-López, Jan P. Meier-Kolthoff, Brian J. Tindall, Sabine Gronow, Tanja Woyke, Nikos C. Kyrpides, Richard L. Hahnke, Markus Göker

**Affiliations:** ^1^Department of Microorganisms, Leibniz Institute DSMZ – German Collection of Microorganisms and Cell Cultures, Braunschweig, Germany; ^2^Department of Energy, Joint Genome Institute, Walnut Creek, CA, United States

**Keywords:** G+C content, genome size, genome BLAST distance phylogeny, chemotaxonomy, morphology, phylogenetic systematics, phylogenomics

## Abstract

Although considerable progress has been made in recent years regarding the classification of bacteria assigned to the phylum *Bacteroidetes*, there remains a need to further clarify taxonomic relationships within a diverse assemblage that includes organisms of clinical, piscicultural, and ecological importance. *Bacteroidetes* classification has proved to be difficult, not least when taxonomic decisions rested heavily on interpretation of poorly resolved 16S rRNA gene trees and a limited number of phenotypic features. Here, draft genome sequences of a greatly enlarged collection of genomes of more than 1,000 *Bacteroidetes* and outgroup type strains were used to infer phylogenetic trees from genome-scale data using the principles drawn from phylogenetic systematics. The majority of taxa were found to be monophyletic but several orders, families and genera, including taxa proposed long ago such as *Bacteroides, Cytophaga*, and *Flavobacterium* but also quite recent taxa, as well as a few species were shown to be in need of revision. According proposals are made for the recognition of new orders, families and genera, as well as the transfer of a variety of species to other genera. In addition, emended descriptions are given for many species mainly involving information on DNA G+C content and (approximate) genome size, both of which can be considered valuable taxonomic markers. We detected many incongruities when comparing the results of the present study with existing classifications, which appear to be caused by insufficiently resolved 16S rRNA gene trees or incomplete taxon sampling. The few significant incongruities found between 16S rRNA gene and whole genome trees underline the pitfalls inherent in phylogenies based upon single gene sequences and the impediment in using ordinary bootstrapping in phylogenomic studies, particularly when combined with too narrow gene selections. While a significant degree of phylogenetic conservation was detected in all phenotypic characters investigated, the overall fit to the tree varied considerably, which is one of the probable causes of misclassifications in the past, much like the use of plesiomorphic character states as diagnostic features.

## Introduction

The *Bacteroidetes* constitute a cosmopolitan phylum that inhabits a broad array of habitats on Earth. One of the key members of the microbiota of animals belongs to this phylum, as they occur especially in the gastrointestinal tract and the oral cavity as commensal microorganisms (Moore et al., 1994; Thomas et al., 2011). *Bacteroidetes* colonize a variety of other natural habitats such as soils, sediments, sea water and freshwater (Thomas et al., 2011), and some even tolerate extreme environmental conditions (Anders et al., 2014). This is reflected in the great metabolic diversity of the phylum, which includes aerobes (Bernardet, 2011a; Nakagawa, 2011a; Hahnke R. L. et al., 2016) as well as anaerobes (Thomas et al., 2011), even though *Bacteroidetes* are generally chemoorganoheterotrophs. Some strains are known as pathogens, particularly of fish (Bernardet and Grimont, 1989; Bernardet et al., 1996). Many *Bacteroidetes* produce pigments, particularly carotenoids (Goodwin, 1980; Sowmya and Sachindra, 2016) and flexirubin-like pigments (Reichenbach et al., 1974, 1980); in some cases proteorhodopsin has been observed (González et al., 2008; Yoshizawa et al., 2012). The cell shape ranges from short rods to long filaments (Saputra et al., 2015; Hahnke R. L. et al., 2016); longer cells are often flexible, including curved or helical shapes (Eder et al., 2015; Shakeela et al., 2015a). Spherical degenerative forms called “spheroblasts” are known from *Flavobacterium* (Bernardet and Bowman, 2006). Many *Bacteroidetes* are motile and, if so, display motility by a special form of gliding (McBride and Zhu, 2013; Nan et al., 2014; McBride and Nakane, 2015; Nan, 2017). Their isoprenoid quinones are menaquinones, usually unsaturated ones, with varying chain lengths (Collins and Jones, 1981). Striking features of *Bacteroidetes* are their specialized carbohydrate decomposition machineries, oligosaccharide uptake systems, and storage features that enables them to play a key role in the decomposition of particulate organic matter (Martens et al., 2009; Sonnenburg et al., 2010; Dodd et al., 2011; Fischbach and Sonnenburg, 2011; Thomas et al., 2011; Reintjes et al., 2017; Mathieu et al., 2018).

The phylum *Bacteroidetes* is subdivided into the classes *Bacteroidia, Chitinophagia, Cytophagia, Flavobacteriia, Saprospiria*, and *Sphingobacteriia*, some of which emerged only in the most recent taxonomic studies on the group (Hahnke R. L. et al., 2016; Munoz et al., 2016). The taxonomic history of the phylum is indeed somehow convoluted. The name *Bacteroidaeota* was recently suggested for the phylum in the course of a proposal to include the rank phylum in the International Code of Nomenclature of Prokaryotes (Oren et al., 2015; Whitman et al., 2018). Yet the name *Bacteroidetes* is not much older (Krieg et al., 2010a) since beforehand these organisms had been referred to as “*Cytophaga-Flavobacteria-Bacteroides* group” (Woese, 1987; Paster et al., 1994). The early classification of the bacteria now placed in *Bacteroidetes* was mainly based on morphological, metabolic, and physiological properties. Genera such as *Flavobacterium* (class *Flavobacteriia*), *Cytophaga*, and *Flexibacter* were differentiated by presence or absence of gliding motility (Bernardet et al., 1996); *Cytophaga* and *Flexibacter*, which are now assigned to the class *Cytophagia*, were also delineated based on cell morphology, G+C content and habitat (Reichenbach, 1989). Anaerobic representatives of the class *Bacteroidia*, which form an important part of the flora of the gastrointestinal tract of animals (Thomas et al., 2011), can be differentiated from aerobic groups such as the classes *Flavobacteriia* and *Cytophagia* (Bernardet, 2011a; Nakagawa, 2011a; Hahnke R. L. et al., 2016) and only relatively recently were recognized as belonging to the same phylum, as based on 16S rRNA gene sequencing (Weisburg et al., 1985; Paster et al., 1994). Anaerobic and aerobic *Bacteroidetes* are still targeted by distinct subcommittees of the International Committee on Systematics of Prokaryotes (Bernardet et al., 2002; Olsen and Shah, 2008). Whereas, some *Bacteroidetes* are halotolerant (Lau et al., 2006a) or thermotolerant (Albuquerque et al., 2011), those originally described as halophilic or thermophilic *Bacteroidetes* are now placed in other phyla (Hahnke R. L. et al., 2016; Munoz et al., 2016). Known from phylogenetic analyses as close relatives of the phylum *Chlorobi, Bacteroidetes* were recently suggested to belong to the “*Bacteroidaeota-Rhodothermaeota-Balneolaeota-Chlorobaeota* superphylum” (Hahnke R. L. et al., 2016), following a proposal to use the ending -aeota for the names of phyla in microbial taxonomy (Oren et al., 2015). If adhering to the simplification of the suffix to -ota (Whitman et al., 2018), this superphylum would need to be called “*Bacteroidota-Rhodothermota-Balneolota-Chlorobiota* superphylum.”

As in other groups of *Bacteria* and *Archaea*, advances in molecular systematics led to the view that taxonomic classification should be based on the integrated use of genotypic and phenotypic data (Wayne et al., 1987; Stackebrandt, 1992), an approach known as polyphasic taxonomy (Colwell, 1970; Vandamme et al., 1996; Gillis et al., 2005; Kämpfer and Glaeser, 2012). In particular, 16S rRNA gene sequences have been routinely applied to infer phylogenetic trees or in conjunction with simpler approaches such as pairwise distance or similarities (Meier-Kolthoff et al., 2013b; Kim and Chun, 2014; Yarza and Munoz, 2014). However, trees based on a few thousand nucleotides such as those based on a single phylogenetic marker (1,400–1,500 nucleotides in the case of the 16S rRNA gene), or even a few concatenated housekeeping genes throughout the technique named Multi-Locus Sequence Analysis (Glaeser and Kämpfer, 2015), tend to have branches with low bootstrap values (Klenk and Göker, 2010). A significant proportion of the current taxonomic classification may thus be lacking statistical evidence (Klenk and Göker, 2010; Hahnke R. L. et al., 2016; Montero-Calasanz et al., 2017; Nouioui et al., 2018). Moreover, while integrating phenotypic information is part of the polyphasic approach, the phenotype is rarely used in a manner that could provide independent evidence because it is not normally separately analyzed but only screened for “diagnostic” features of (often unsupported) groups seen in 16S rRNA gene trees (Montero-Calasanz et al., 2017). The principles of phylogenetic systematics instead insist on monophyletic taxa, which implies that they must be based on apomorphic (derived) character states and not merely on “diagnostic” ones (Hennig, 1965; Wiley and Lieberman, 2011; Montero-Calasanz et al., 2017; Nouioui et al., 2018). Thus, the question arises which taxa proposed by polyphasic taxonomy are actually monophyletic.

Indeed, given the rapid and ongoing progress in sequencing technologies (Mavromatis et al., 2012), classifications based on whole genome sequences and associated bioinformatic tools can exploit millions of characters and thereby provide a step change in reliability, as evidenced by high average bootstrap support in phylogenomic trees (Breider et al., 2014; Meier-Kolthoff et al., 2014a). Yet the ordinary bootstrap is not necessarily the most reliable approach when dealing with supermatrices potentially comprised of genes with distinct evolutionary histories (Siddall, 2010; Simon et al., 2017). The taxonomic classification of *Bacteroidetes* has recently been revised based on the 16S and 23S rRNA genes in conjunction with 29 orthologous protein sequences (Munoz et al., 2016), which resulted in the proposal of several new taxa within *Bacteroidetes* as well as the new phylum *Rhodothermaeota*. A number of additional *Bacteroidales* families have been proposed in a recent phylogenomic study (Ormerod et al., 2016), whereas genome sequences from phase I of the One Thousand Microbial Genomes (KMG) project (Mukherjee et al., 2017) were used in an initial study covering the entire phylum (Hahnke R. L. et al., 2016). Again, reclassifications resulted at all levels of the taxonomic hierarchy. Additionally, it was shown that DNA G+C composition values directly calculated from genome sequences have a significantly better fit to the phylogeny than the experimentally determined ones cited in many species descriptions (Hahnke R. L. et al., 2016). This is in line with the observation that within-species variation is at most 1% when G+C content is calculated from genome sequences (Meier-Kolthoff et al., 2014c) and that previous reports in the literature that the variation in G+C content within bacterial species is at most 3 mol% (Mesbah et al., 1989) or even 5% (Rosselló-Mora and Amann, 2001) can be attributed to experimental error in traditional methods (Mesbah et al., 1989; Moreira et al., 2011).

Despite the recent progress, coverage of *Bacteroidetes* type strains with genome sequences was far from complete in the cited studies, and many taxonomic questions remained unanswered. This is problematic particularly since taxonomic classification is not an end in itself but affects all other biological disciplines, in particular ecology, including the ecology of *Bacteroidetes* (Hahnke R. L. et al., 2016). Moreover, in contrast to the G+C content (Hahnke R. L. et al., 2016) genome size has been minimally investigated as taxonomic marker. In a recent study on the phylum *Actinobacteria* genome sized appeared to work reasonably well as marker albeit less well than the G+C content (Nouioui et al., 2018). Likewise, it has not yet systematically been explored how well the phenotypic markers traditionally used in *Bacteroidetes* taxonomy actually fit to trees inferred from genome-scale data. In fact, phylogenetic conservation needs not even be measurable in features traditionally used in microbial taxonomy, but the same holds for genomic features, including individual alignment positions in individual genes (Carro et al., 2018).

Expanding our previous study on *Bacteroidetes* (Hahnke R. L. et al., 2016) and in analogy to our study on the phylum *Actinobacteria* (Nouioui et al., 2018), we here use genome sequences from phase II of the KMG project augmented with publicly available ones generated by third parties, yielding a phylogenomic dataset covering more than 1,000 type-strains of *Bacteroidetes* and outgroup taxa. A comprehensive collection of type-strain 16S rRNA gene sequences from the literature was used to further complement these data. Genome-scale phylogenetic trees were constructed to address the following questions: (i) to what extent are phylogenies calculated from whole genome sequences still in conflict with the current classification of *Bacteroidetes* and with their 16S (or 23S) rRNA gene phylogenies? (ii) Which taxa need to be revised because they are evidently non-monophyletic? (iii) What are historical causes for the establishment of these non-monophyletic taxa? (iv) Which taxon descriptions should be modified because of inaccurate or missing G+C values? and (v) How do standard phenotypic markers, G+C values and genome sizes of *Bacteroidetes* relate to their phylogeny and to which degree can they serve as a taxonomic markers?

## Materials and Methods

A total number of 1,040 *Bacteroidetes* and outgroup type-strain genome sequences and annotations (Supplementary Table 1) were taken from an earlier study (Hahnke R. L. et al., 2016) and augmented with additional ones collected from GenBank but mainly with genome sequences obtained *de novo* in the course of the KMG project phase II (Mukherjee et al., 2017) and annotated and deposited in the Integrated Microbial Genomes platform (Chen et al., 2019) and in the Type-Strain Genome Server database (Meier-Kolthoff and Göker, 2019). All newly generated KMG sequences underwent standard quality control at DSMZ for DNA extraction and at JGI for genome sequencing documented on the respective web pages and yielded <100 contigs. All genome sequences had <500 contigs and matched the 16S rRNA gene reference database described below. Structural annotation at JGI and DSMZ was done using Prodigal v. 2.6.2 (Hyatt et al., 2010). These annotated genome sequences were processed further as in our previous study using the high-throughput version of the Genome BLAST Distance Phylogeny (GBDP) approach in conjunction with BLAST+ v2.2.30 in blastp mode (Auch et al., 2006; Camacho et al., 2009; Meier-Kolthoff et al., 2014a) and FastME v 2.1.6.1 using the improved neighbor-joining algorithm BioNJ for obtaining starting trees followed by branch swapping under the balanced minimum evolution criterion (Desper and Gascuel, 2004) using the subtree-pruning-and-regrafting algorithm (Desper and Gascuel, 2006; Lefort et al., 2015). One hundred pseudo-bootstrap replicates (Meier-Kolthoff et al., 2013a, 2014a) were used to obtain branch-support values for these genome-scale phylogenies. Trees were visualized using Interactive Tree Of Life (Letunic and Bork, 2011) in conjunction with the script deposited at https://github.com/mgoeker/table2itol. The choice of outgroup taxa was based on previous results (Hahnke R. L. et al., 2016) but now yielded a broader sampling of species. Species and subspecies boundaries were explored using digital DNA:DNA hybridization (dDDH) as implemented in the Genome-To-Genome Distance Calculator (GGDC) version 2.1 (Meier-Kolthoff et al., 2013a) and in the Type (Strain) Genome Server (Meier-Kolthoff and Göker, 2019). The features of all genome sequences that entered these analyses are provided in Supplementary Table 1.

A comprehensive set of aligned, near full-length 16S rRNA gene sequences was generated by augmenting the previous collection (Hahnke R. L. et al., 2016) with sequences from more recent species descriptions. The taxonomic affiliation of genomes was checked using RNAmmer version 1.2 (Lagesen et al., 2007) to extract 16S rRNA gene sequences, which where compared with the 16S rRNA gene reference database using BLAST and phylogenetic trees. Non-matching genome sequences were discarded from further analyses. A comprehensive sequence alignment was generated using MAFFT version 7.271 with the “localpair” option (Katoh et al., 2005), using either the sequences extracted from the genome sequences or the previously published 16S rRNA gene sequences, depending on the length and number of ambiguous bases. Trees were inferred from the alignment with RAxML (Stamatakis, 2014) version 8.2.12 under the maximum-likelihood (ML) criterion and with TNT (Goloboff et al., 2008) version Dec. 2017 under the maximum-parsimony (MP). In addition to unconstrained, comprehensive 16S rRNA gene trees (UCT), constrained comprehensive trees (CCT) were inferred with ML and MP using the bipartitions of the GBDP tree with ≥95% support as backbone constraint, as previously described (Hahnke R. L. et al., 2016). The purpose of the constraint, which enforces the monophyly of the well-supported groups from the GBDP, is to inject information from the phylogenomic analysis into the 16S rRNA gene analyses, which cover more organisms but fewer characters. Finally, unconstrained 16S rRNA gene trees reduced to genome-sequenced strains (URT) were inferred, as well as unconstrained 23S (i.e., large subunit) rRNA gene trees (ULT).

The previously collected hierarchical taxonomic classification of ingroup and outgroup taxa (Hahnke R. L. et al., 2016) was augmented by screening the taxonomic literature. As in the previous study, taxa were analyzed to determine whether they were monophyletic, paraphyletic or polyphyletic (Farris, 1974; Wood, 1994). Taxa non-monophyletic according to the GBDP tree were tested for evidence for their monophyly in the UCT, ULT, URT, and the 16S rRNA gene trees, if any, in the original publication.

In the case of a significant conflict (i.e., high support values for contradicting bipartitions, with ≥95% support considered as high) between trees or low support in the GBDP tree, additional phylogenomic analyses of selected taxa were conducted. To this end, MCL (Markov Chain Clustering) version 14–137 (Enright et al., 2002) under default settings and an e-value filter of 10^−5^ was used to the reciprocal best hits from GBDP/BLAST in analogy to OrthoMCL (Li et al., 2003). The resulting sets of orthologous proteins were aligned with MAFFT and concatenated to form a supermatrix after discarding the few clusters that still contained more than a single protein for at least one genome. Comprehensive supermatrices were compiled from all the orthologs that occurred in at least four genomes, whereas core-genome supermatices were constructed for the orthologs that occurred in all of the genomes. Supermatrices were analyzed with TNT, and with RAxML under the “PROTCATLGF” model, in conjunction with 100 partition bootstrap replicates, i.e., by sampling (with replacement) not the single alignment positions but entire orthologs (Siddall, 2010; Simon et al., 2017; Nouioui et al., 2018). Shimodaira-Hasegawa (SH) paired-site tests as implemented in RAxML were conducted with accordingly reduced 16S rRNA gene alignments in such cases using the supermatrix ML trees as constraint to assess whether these tests also indicated a significant (α = 0.01) conflict between 16S rRNA gene and genome-scale data. When it was of interest, supermatrices where also subjected to ordinary bootstrapping as implemented in RAxML, as were single genes selected from these supermatrices.

G+C content values and genome sizes are trivial to calculate (Hahnke R. L. et al., 2016; Nouioui et al., 2018). Additionally, selected phenotypic features relevant for the taxonomic classification of *Bacteroidetes* were as comprehensively as possible collected from the taxonomic literature: average cell length, average cell width, motility by gliding, absence or presence of carotenoids, absence or presence of flexirubin-like pigments, average number of isoprene residues of the major menaquinones (MK), and relationship to oxygen. To this end, a recently published collection of phenotypic data from taxonomic publications (Barberán et al., 2017) was corrected and augmented. To avoid circular reasoning, missing features of a species were only inferred from features of its genus when species and genus were described in the same publication or when the species description had explicitly been declared as adding to the features of the genus. The reported relationships to oxygen were checked against the cultivation conditions used for KMG at DSMZ and where necessary augmented. Oxygen conditions were coded as ordered multi-state character: (1) strictly anaerobic, (2) facultatively aerobic, (3) facultatively anaerobic, (4) strictly aerobic; microaerophilic was treated like missing data. Among all coding options tested, this yielded the highest fit to the tree (Supplementary Table 1); the second best option was to code microaerophilic and strictly aerobic as the same character state. MK percentages would be more informative than just statements about being “major” but mostly only the latter are provided in the literature. Phylogenetic conservation of selected phenotypic and genomic characters with respect to the GBDP tree (reduced to represent each set of equivalent strains by only a single genome) was evaluated using a tip-permutation test in conjunction with the calculation of maximum-parsimony scores with TNT as previously described (Simon et al., 2017; Carro et al., 2018) and 10,000 permutations. While more sophisticated tests of phylogenetic conservation are available particularly for continuous characters, this approach eases the comparison of discrete and continuous characters as TNT deals with both. TNT input files were generated with opm (Vaas et al., 2013). The proportion of times the score of a permuted tree was at least as low as the score of the original tree yielded the *p*-value. Maximum-parsimony retention indices (Farris, 1989; Wiley and Lieberman, 2011) were calculated to further differentiate between the fit of each character to the tree.

Unambiguously non-monophyletic taxa according to the genome-scale analyses were screened for published phenotypic evidence of their monophyly. Published evidence was judged as inconclusive when based on probably homoplastic characters or on probable plesiomorphic character states. Importantly, “diagnostic” features alone are insufficient in phylogenetic systematics, as plesiomorphies might well be diagnostic but just for paraphyletic groups (Hennig, 1965; Wiley and Lieberman, 2011; Montero-Calasanz et al., 2017; Nouioui et al., 2018). Finally, taxonomic consequences were proposed to fix all obviously non-monophyletic taxa by new taxon delineations sufficiently supported by the CCT, i.e., not hindered by the uncertain phylogenetic placement of taxa whose genome sequences were not available at the time of writing.

## Results

The presentation of the results of this study is organized as follows. After a brief overview on the figures and tables the outcome of the tests for the phylogenetic conservation are illustrated. Next, the phylogenetic results for the outgroup taxa and certain phylogenetically deviating *Bacteroidetes* are described and put in the context of their current taxonomic classification. Finally, the hierarchical classification of the phylum *Bacteroidetes* itself, arranged according to the six classes in which it is currently subdivided, is compared to the phylogenomic trees. These sections motivate the need for a variety of reclassifications, whereas the actual taxonomic consequences are listed at the end of the Discussion chapter.

**Figure 1:**
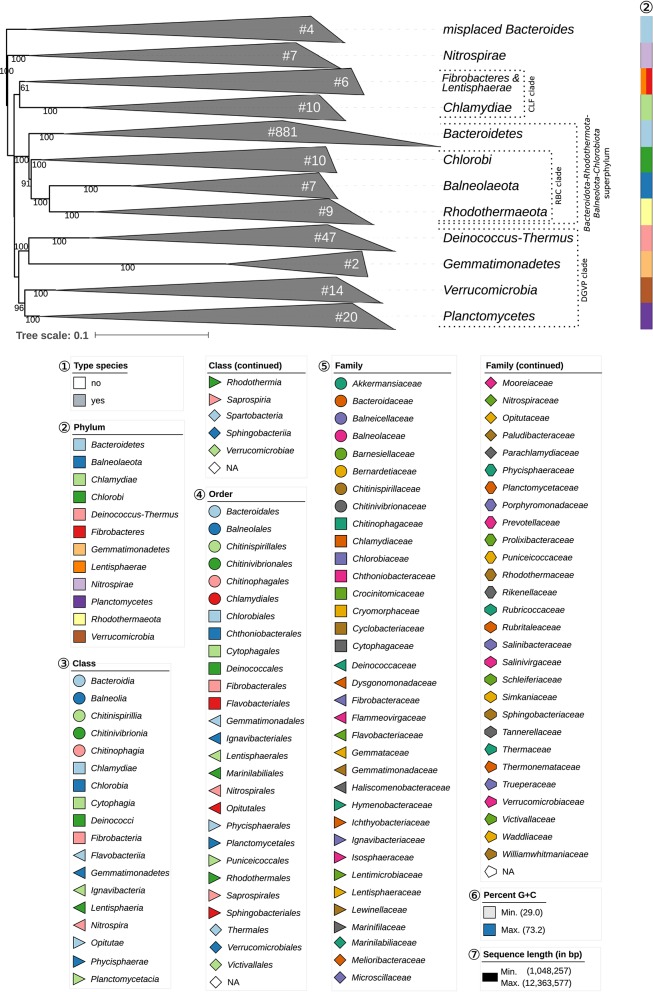
Overview of the phylogenomic tree inferred with FastME from GBDP distances calculated from whole proteomes. The numbers above branches are GBDP pseudo-bootstrap support values from 100 replications. Collapsed clades are displayed as triangles whose side lengths are proportional to the branch-length distances to least and most distant leave, respectively. The total number (#) of leaves per collapsed clade is shown within the triangles. The legend indicates the symbols and colors used in all subsequent figures, which show details of all clades of interest. These clades are composed of the following phyla: CLF, *Chlamydiae-Lentisphaerae-Fibrobacteres* clade; DGPV, *Deinococcus-Thermus-Gemmatimonadetes-Planctomycetes-Verrucomicrobia* clade; RBC, *Rhodothermaeota-Balneolaeota-Chlorobi* clade. These clades are weakly supported and annotated for display purposes only; they are not suggested as reliable groupings. Figures 2–8 show specific sections of the same tree in greater detail; while the underlying topology is exactly the same, the ordering of the clades may slightly differ.

**Figure 2:**
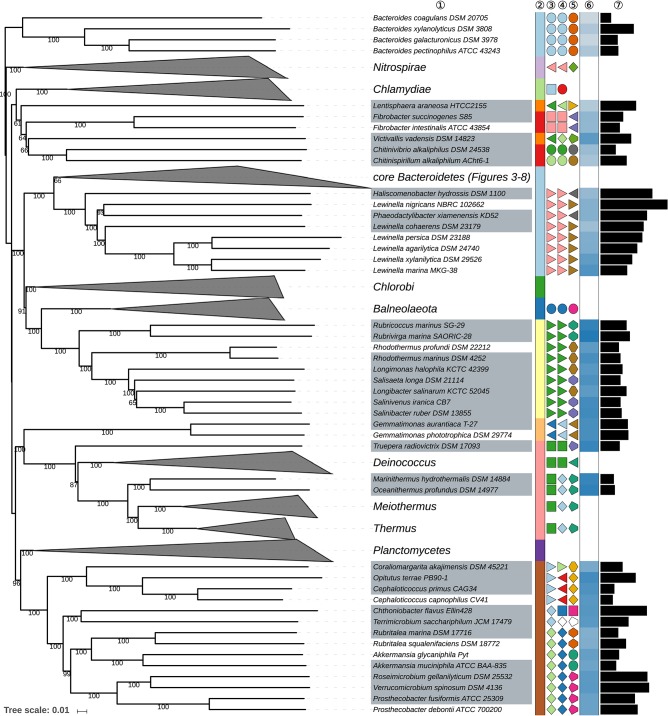
First part of the GBDP tree shown in Figure 1, focusing on misplaced *Bacteroides* species, on taxa outside the phylum *Bacteroidetes* and on the class *Saprospiria*. Tip labels with gray background indicate type species of genera, colors, and symbols to the right of the tips indicate, from left to right, phylum, class, order, and family; for details and abbreviations (see Figure 1). The blue color gradient right indicates the G+C content as calculated from the genome sequences, followed by black bars indicating the (approximate) genome size in base pairs.

**Figure 3:**
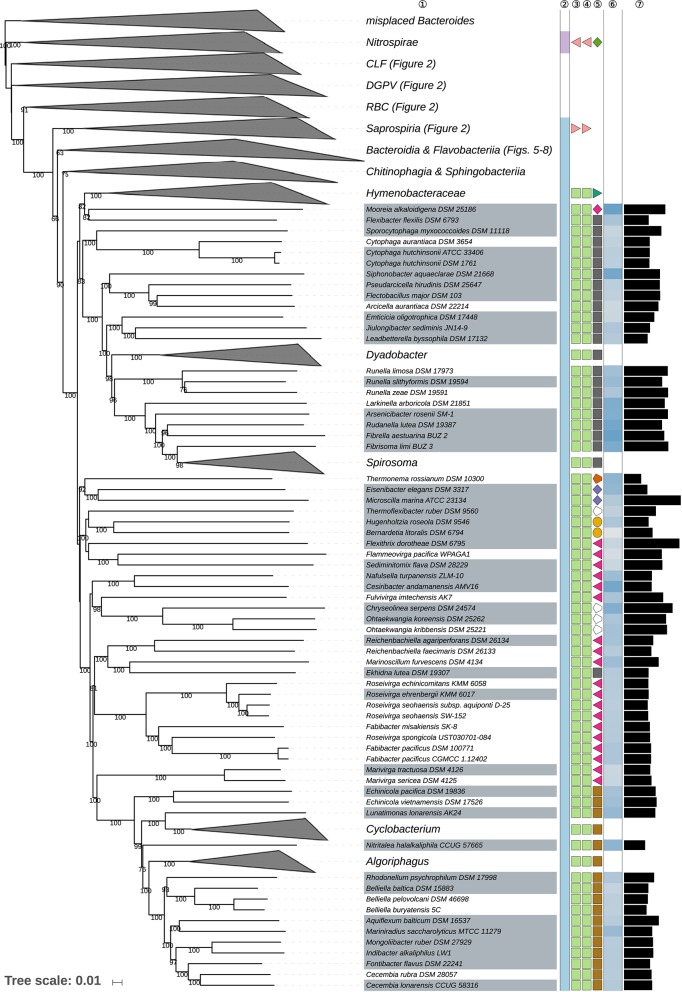
Second part of the GBDP tree shown in Figure 1, focusing on the class *Cytophagia*. Tip labels with gray background indicate type species of genera, colors, and symbols to the right of the tips indicate, from left to right, phylum, class, order, and family; for details and abbreviations (see Figure 1). The blue color gradient right indicates the G+C content as calculated from the genome sequences, followed by black bars indicating the (approximate) genome size in base pairs.

**Figure 4:**
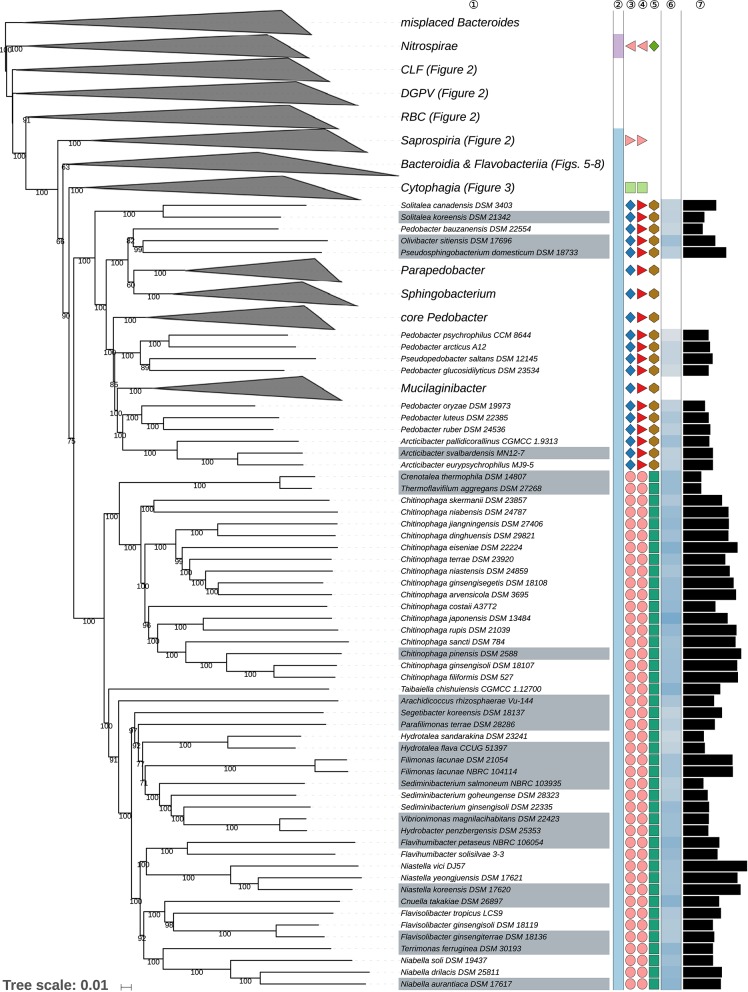
Third part of the GBDP tree shown in Figure 1, focusing on the classes *Chitinophagia* and *Sphingobacteriia*. Tip labels with gray background indicate type species of genera, colors, and symbols to the right of the tips indicate, from left to right, phylum, class, order, and family; for details and abbreviations (see Figure 1). The blue color gradient right indicates the G+C content as calculated from the genome sequences, followed by black bars indicating the (approximate) genome size in base pairs.

**Figure 5:**
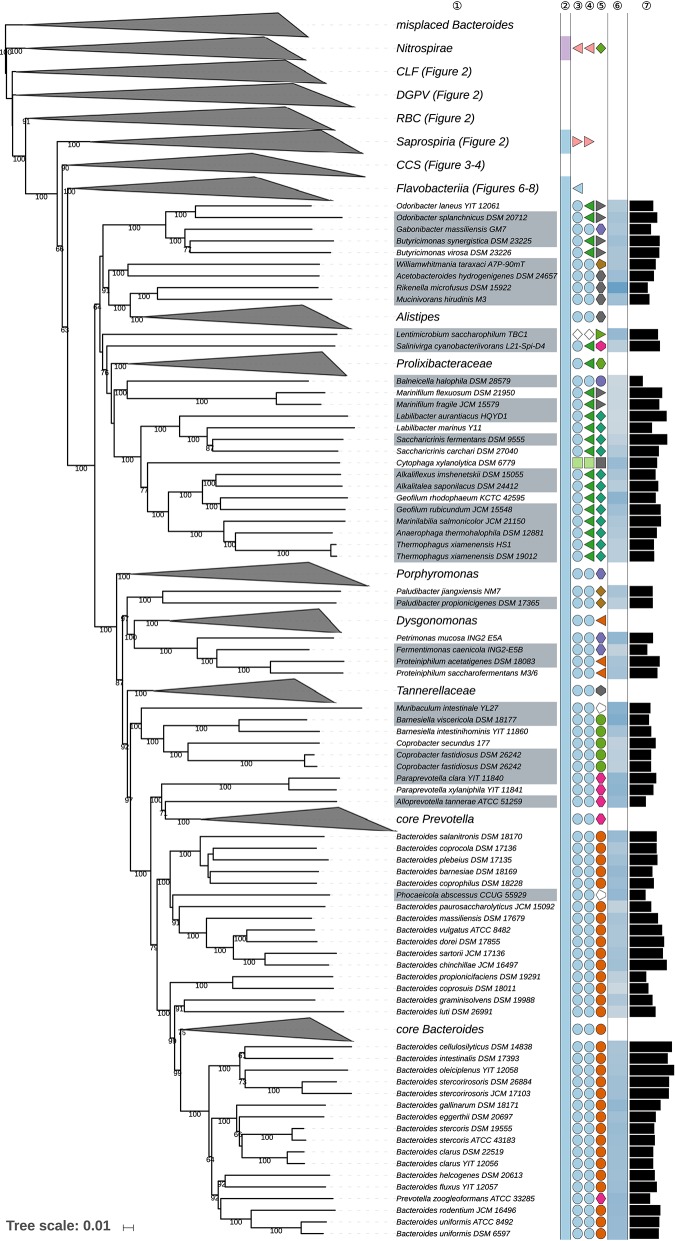
Fourth part of the GBDP tree shown in Figure 1, focusing on the class *Bacteroidia*. Tip labels with gray background indicate type species of genera, colors, and symbols to the right of the tips indicate, from left to right, phylum, class, order, and family; for details and abbreviations (see Figure 1). The blue color gradient right indicates the G+C content as calculated from the genome sequences, followed by black bars indicating the (approximate) genome size in base pairs. CCS, *Cytophagia-Chitinophagia-Sphingobacteriia* clade.

**Figure 6:**
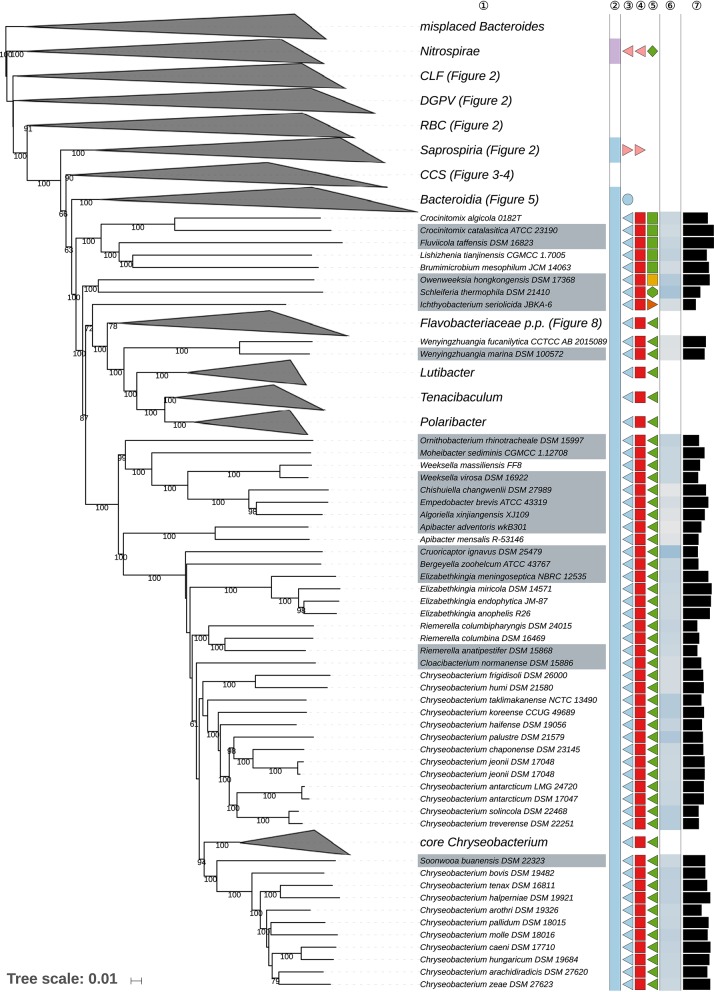
Fifth part of the GBDP tree shown in Figure 1, focussing on parts of the class *Flavobacteriia*. Tip labels with gray background indicate type species of genera, colors, and symbols to the right of the tips indicate, from left to right, phylum, class, order, and family; for details and abbreviations (see Figure 1). The blue color gradient right indicates the G+C content as calculated from the genome sequences, followed by black bars indicating the (approximate) genome size in base pairs. CCS, *Cytophagia-Chitinophagia-Sphingobacteriia* clade.

**Figure 7:**
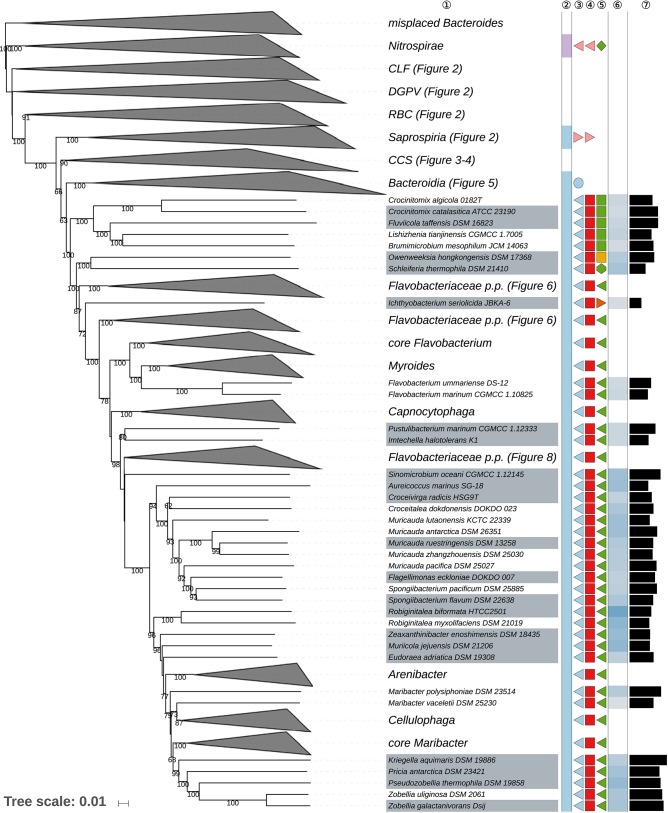
Sixth part of the GBDP tree shown in Figure 1, focussing on parts of the class *Flavobacteriia*. Tip labels with gray background indicate type species of genera, colors, and symbols to the right of the tips indicate, from left to right, phylum, class, order, and family; for details and abbreviations (see Figure 1). The blue color gradient right indicates the G+C content as calculated from the genome sequences, followed by black bars indicating the (approximate) genome size in base pairs. CCS, *Cytophagia-Chitinophagia-Sphingobacteriia* clade.

**Figure 8:**
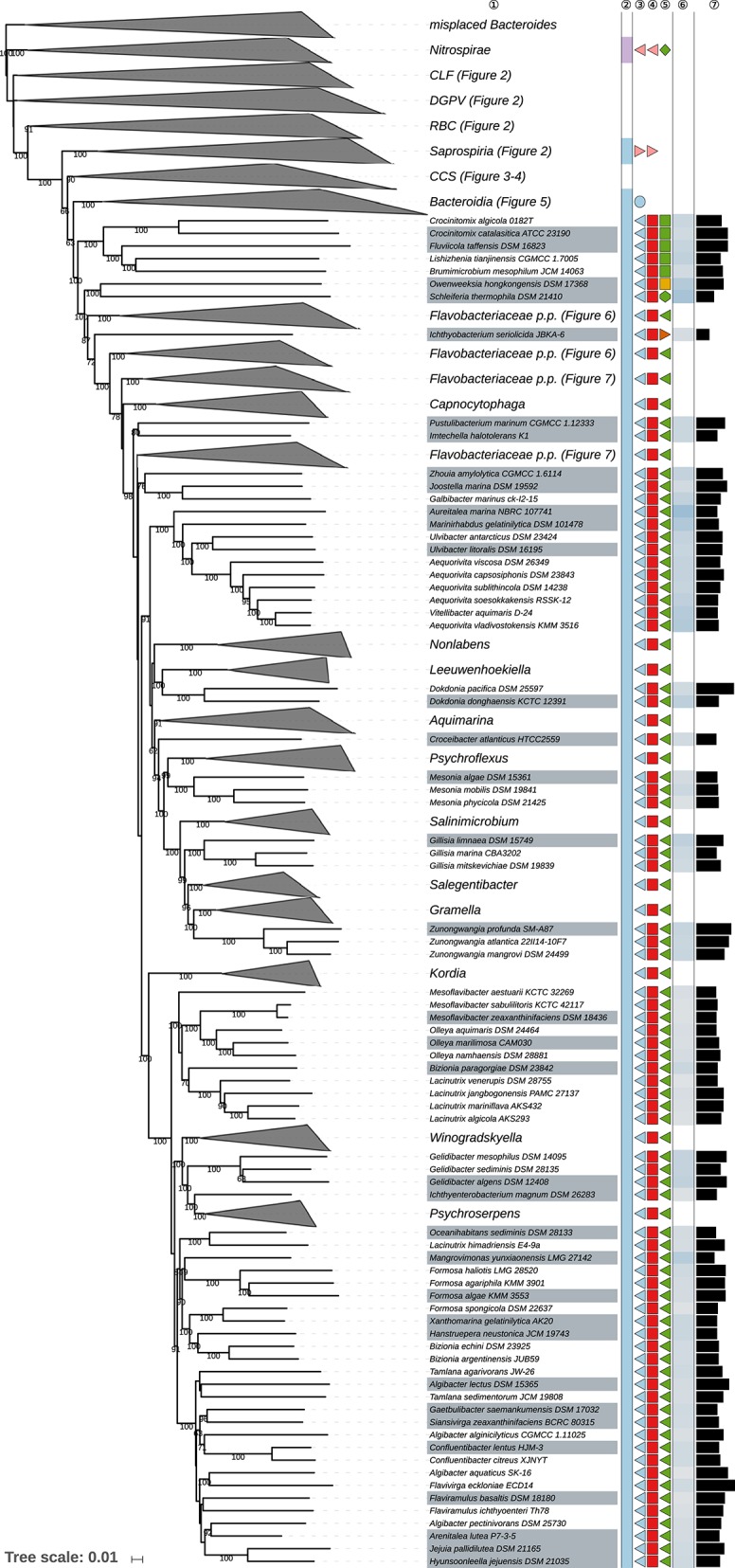
Seventh part of the GBDP tree shown in Figure 1, focussing on parts of the class *Flavobacteriia*. Tip labels with gray background indicate type species of genera, colors, and symbols to the right of the tips indicate, from left to right, phylum, class, order, and family; for details and abbreviations (see Figure 1). The blue color gradient right indicates the G+C content as calculated from the genome sequences, followed by black bars indicating the (approximate) genome size in base pairs. CCS, *Cytophagia-Chitinophagia-Sphingobacteriia* clade.

The GBDP tree is shown in Figures 1–8; Figure 1 provides an overview whereas Figures 2–8 display specific sections of the same tree in greater details. Phenotypic information for groups of taxa whose taxonomic classification is treated in detail below is summarized in Supplementary Table 1. This supplementary table also includes the complete list of genome sequences used in this study as well as additional dDDH values for pairs of strains of interest. Additional phylogenetic trees, including the GBDP tree in a single figure and with phenotypic annotation, are found in Supplementary Data Sheet 1.

Most of the *Bacteroidetes* and outgroup taxa appeared to be monophyletic in the GBDP tree, mainly with high bootstrap support. For instance, with the exception of few deviating *Bacteroides* species that did not even appear to phylogenetically belong to the phylum, all six classes were shown as monophyletic with strong support. However, other taxa seemed to be in need of a taxonomic revision because they appeared as paraphyletic or polyphyletic. For instance, most genera appeared as monophyletic, usually with high support. In some cases, however, genera were shown as non-monophyletic, as exemplified by *Algibacter* (Nedashkovskaya et al., 2004b, 2007c; Park et al., 2013d; Shakeela et al., 2015b) *Flaviramulus* (Einen and Øvreås, 2006; Zhang Y. et al., 2013)*, Maribacter* (Nedashkovskaya et al., 2004a, 2010a; Barbeyron et al., 2008; Lo et al., 2013; Weerawongwiwat et al., 2013; Hu et al., 2015; Jackson et al., 2015), and *Tamlana* (Lee, 2007; Jeong et al., 2013b). In most of these cases, 16S rRNA gene sequence analyses (Supplementary Data Sheet 1) already revealed the same taxonomic problems albeit often with lower support for certain clades. All discrepancies are described below assorted by phylum or class. Where non-monophyletic taxa depicted in a figure, “core” marks the clade that contains the respective type.

While Tables 1, 2 show the results on phylogenetic conservation, Table 3 provides dDDH values (Meier-Kolthoff et al., 2013a; Meier-Kolthoff and Göker, 2019) of selected pairs of strains. Some dDDH values between pairs of species were found to be higher than 70%, the current accepted threshold to differentiate among species (Wayne et al., 1987). In turn, some of these were lower than 79%, the threshold defined to differentiate among subspecies (Meier-Kolthoff et al., 2014b). All of the pairs of genome sequences of deposits considered to represent the same type strain were found to have dDDH similarities of 99.0% or above with the exception of *Bacteroides faecis* (Kim M.-S. et al., 2010), *B. ovatus* (Eggerth and Gagnon, 1932; Hahnke R. L. et al., 2016), *B. stercoris* (Johnson et al., 1986), *B. uniformis* (Eggerth and Gagnon, 1932; Hahnke R. L. et al., 2016), *Filimonas lacunae* (Shiratori et al., 2009; Leandro et al., 2013), *Flavobacterium akiainvivens* (Kuo et al., 2013), *F. chilense* (Kämpfer et al., 2012b), *F. johnsoniae, Polaribacter dokdonensis* (Yoon et al., 2006), *Prevotella scopos* and *Thermophagus xiamenensis* (Gao et al., 2013), whose dDDH values ranged between 89.6 and 98.9% dDDH, results which may account for the separation of each pair of these strains.

**Table 1:** *P*-values from the tip-permutation test of the GBDP tree shown in Figures 1–8 and other results obtained for the selected genomic and phenotypic features.

**Feature**	**Data type**	**Coverage**	**RI**	***P*-value**
Percent G+C content	Continuous	100%	0.762	1e-04
Approximate genome size in bp	Continuous	100%	0.632	1e-04
Cell length in μm	Continuous	92%	0.270	1e-04
Cell width in μm	Continuous	85%	0.372	1e-04
Motility by gliding	Discrete, binary	89%	0.478	1e-04
Relationship to oxygen	Discrete, ordered multi-state	93%	0.855	1e-04
Carotenoids	Discrete, binary	19%	0.629	1e-04
Flexirubin-like pigments	Discrete, binary	55%	0.647	1e-04
Average number of isoprene residues in major menaquinones	Continuous	62%	0.899	1e-04

*Genome size in base pairs is necessarily approximate in many cases because of unfinished genome sequences. The retention index (RI) can be used to compare the fit of distinct characters to a tree; the maximum of 1.0 indicates a perfect fit without any homoplasies*.

**Table 2:** Counts of the average number of isoprene residues of the major menaquinones per class of the phylum *Bacteroidetes*, rounded to zero decimal places.

**Class**	**4**	**5**	**6**	**7**	**8**	**9**	**10**	**11**	**12**	**N.A.**
*Bacteroidia*	0	0	1	30	12	3	37	7	7	125
*Chitinophagia*	0	0	5	106	0	0	0	0	0	9
*Cytophagia*	2	0	2	281	0	0	0	0	0	58
*Flavobacteriia*	0	2	614	5	0	0	0	0	0	215
*Saprospiria*	0	0	0	15	0	0	0	0	0	3
*Sphingobacteriia*	0	0	10	162	2	0	0	0	0	27

*Literature sources where taken from the database of the DSMZ Prokaryotic Nomenclature Up-To-Date service. N.A., not available in description of species or subspecies*.

**Table 3:** Outcome of applying GGDC to calculate intergenomic dDDH values.

**Strain 1**	**Strain 2**	**dDDH**	**Consequence**
*Chryseobacterium aquaticum* KCTC 12483	*Chryseobacterium greenlandense* NRRL B-59357	71.1	new subspecies of *C. aquaticum* from *C. greenlandense*
*Elizabethkingia anophelis* R26	*Elizabethkingia endophytica* JM-87	76.9	new subspecies of *E. anophelis* from species *E. endophytica*
*Flavobacterium johnsoniae* ATCC 17061	*Flexibacter aurantiacus* DSM 6792	71.1	new subspecies of *F. johnsoniae* from *F. aurantiacus*
*Flavobacterium tructae* CCUG 60100	*Flavobacterium spartansii* ATCC BAA-2541	80.4	*F. spartansii is later heterotypic synonym*
*Hydrobacter penzbergensis* DSM 25353	*Vibrionimonas magnilacihabitans* DSM 22423	83.2	*H. penzbergensis* is later heterotypic synonym
*Lactobacillus rogosae* ATCC 27753	*Bacteroides galacturonicus* DSM 3978	93.4	*B. galacturonicus* is later heterotypic synonym
*Mesoflavibacter zeaxanthinifaciens* DSM 18436	*Mesoflavibacter sabulilitoris* KCTC 42117	74.7	new subspecies of *M. zeaxanthinifaciens* from *M. sabulilitoris*
*Myroides odoratimimus* ATCC BAA-634	*Myroides xuanwuensis* DSM 27251	78.8	new subspecies of *M. odoratimimus* from species *M. xuanwuensis*
*Nonlabens tegetincola* JCM 12886	*Nonlabens sediminis* NBRC 100970	79.0	*N. sediminis* is later heterotypic synonym
*Prevotella dentalis* DSM 3688	*Hallella seregens* ATCC 51272	87.4	*H. seregens* is later heterotypic synonym
*Thermodesulfovibrio yellowstonii* DSM 11347	*Thermodesulfovibrio islandicus* DSM 12570	92.4	*T. islandicus* is later heterotypic synonym

*Only results that yield taxonomic consequences are shown. Note that some of the examined taxa, such as Hallella seregens, were already known as later heterotypic synonyms*.

### Phylogenetic Conservation of Genomic and Phenotypic Markers of Interest

Table 1 shows the *p*-values obtained by the tip-permutation test and the retention values of selected genomic and phenotypic features. All investigated characters showed a significant phylogenetic conservation (α = 0.001) but the fit of each character to the tree as indicated by the retention index varied considerably. The relatively high correspondence between G+C content and phylogeny comes as no surprise (Hahnke R. L. et al., 2016), while the occurrence of phylogenetic conservation of genome size can easily be spotted in the GBDP tree (Figures 1–8, Supplementary Data Sheet 1). For instance, genera with uniformly rather small genomes include *Capnocytophaga* (2.7 ± 0.2 Mbp), *Porphyromonas* (2.2 ± 0.2 Mbp), and *Prevotella* (3.0 ± 0.3 Mbp) whereas *Dyadobacter* (7.1 ± 1.3 Mbp), *Niastella* (9.1 ± 0.7 Mbp), and *Spirosoma* (7.3 ± 1.0 Mbp) are genera with uniformly relatively large genomes. Within *Flavobacterium* genome size apparently increased within a certain subclade. In contrast, within the clade corresponding to core *Bacteroides*, i.e., the clade containing the type species, genome size (4.9 ± 1.1 Mbp) appeared to change rapidly in evolutionary terms relative to the moderate average size.

Morphology showed a significant but low conservation. In the case of cell length a certain degree of inertia was visible in the tendency to filamentous cells in related groups such as *Eisenibacter* and *Microscilla* or the genus *Marivirga* while elongated cells also occurred in taxa dispersed through the phylogeny such as *Chitinophaga filiformis* and *Chryseobacterium solincola* (Supplementary Data Sheet 1). Cell width showed a slightly higher retention index (Table 1); there was an obvious tendency for broader cells in groups such as *Bacteroides* but there are also many isolated occurrences of cells with a width above average (Supplementary Data Sheet 1). The almost as well-sampled character “gliding motility” showed a retention index comparable to the one of cell width. While absent in almost all outgroup taxa, motility by gliding has a rather scattered occurrence in the phylum *Bacteroidetes* (Supplementary Data Sheet 1). Relationship to oxygen showed a considerably higher retention index, which probably mainly reflected the concentration of strictly anaerobic strains in certain outgroup taxa such as the phylum *Chlorobi* and certain subgroups of the class *Bacteroidia*; additionally, *Bacteroidia* did not contain any strict aerobes (Supplementary Data Sheet 1).

As for chemotaxonomy, presence or absence of carotenoids showed a higher retention index but while many *Bacteroidetes* were described as pigmented, it was only rarely explicitly reported whether or not the contained pigments were carotenoids (Supplementary Data Sheet 1), which renders it difficult to attribute the apparent conservation to presence or absence in specific groups. Presence or absence of flexirubins was more frequently reported and obtained a somewhat higher retention index, which may in part be attributable to the complete lack of flexirubins in the outgroup taxa (Supplementary Data Sheet 1).

The average number of isoprene residues of the major menaquinones was even more frequently reported and achieved the highest retention index of all characters investigated. The distribution of the character states over the phylogeny indicated that this outcome reflected the occurrence of longer isoprenoid chains in some outgroup phyla but also in specific subgroups of the class *Bacteroidia*, including *Alistipes, Bacteroides, Parabacteroides*, and *Prevotella*, as well as usually shorter isoprenoid chains in the class *Flavobacteriia* (Table 2, Supplementary Data Sheet 1). The GBDP tree (Figures 1–8) indicated that neither *Flavobacteriia* nor *Bacteroidia* separated first from the other classes of *Bacteroidetes*, which was confirmed by supermatrix analyses (Supplementary Data Sheet 1). Accordingly, the plesiomorphic state is most likely the occurrence of seven isoprene residues in the major menaquinone, whereas a reduction to six is an apomorphy of *Flavobacteriia* and an increase to ten and more is an apomorphy of certain subgroups of *Bacteroidia* (Table 2).

### Deviating *Bacteroidetes*

Four species classified in *Bacteroides* were phylogenetically neither placed within the phylum *Bacteroidetes* nor any of the chosen outgroup phyla, *B. coagulans, B. galacturonicus, B. pectinophilus*, and *B. xylanolyticus* (Figures 1, 2); they were thus used to root the tree. Additional 16S rRNA gene and GBDP analyses confirmed the placement of these species at distinct positions within *Eubacteriales* (Supplementary Data Sheet 1). This order within the phylum *Firmicutes* is perhaps more widely known as *Clostridiales* but as long as *Eubacterium* is included in the order, it is of relevance that *Eubacteriales* has priority (Gerritsen et al., 2014). The four deviant *Bacteroides* species are comparatively old and were originally proposed based on phenotypic features with an emphasis on physiology while sequencing the 16S rRNA gene or any other genetic marker could not yet be carried through at that time. Unlike *Eubacteriales*, these *Bacteroides* species were described as Gram-negative. Gram staining can however change with the age of cultures in some *Eubacteriales* (Bryant and Small, 1956) and it is not always evident from the literature whether the staining reactions were actually conducted or inferred from general properties of the genus *Bacteroides*.

In the 16S rRNA gene tree specifically inferred to resolve its position (Supplementary Data Sheet 1), *B. coagulans* (Eggerth and Gagnon, 1932) formed a strongly supported clade together with *Ezakiella peruensis* (Patel et al., 2015); this clade in turn appeared as sister group of *Fenollaria* (Pagnier et al., 2014) with almost equally strong support. While a genome sequence of *Ezakiella* was not available at the time of writing, the *Eubacteriales* GBDP tree confirmed the sister-group relationship between *Fenollaria* and *B. coagulans* (Supplementary Data Sheet 1). *E. peruensis* and *B. coagulans* share a 16S rRNA gene similarity of 96.15%, which indicates they do not belong to the same species (Meier-Kolthoff et al., 2013b) but may well be placed in the same genus, which is the taxonomically most conservative solution and not precluded by their scarcely recorded phenotypic features (Supplementary Table 1).

*B. pectinophilus* (Jensen and Canale-Parola, 1986) shows a comparatively isolated position within the *Eubacteriales* 16S rRNA gene tree with no obvious affiliation to an existing genus (Supplementary Data Sheet 1). In the *Eubacteriales* GBDP tree it formed the sister group of a clade comprising *Lachnospira multipara* (Bryant and Small, 1956) and the apparently taxonomically misplaced *Eubacterium eligens* (Holdeman and Moore, 1974) and *Lactobacillus rogosae* (Holdeman and Moore, 1974); this clade in turn appeared as sister group of *Coprococcus eutactus* (Holdeman and Moore, 1974). Additional genera only represented in the 16S rRNA gene tree that could potentially form a clade together with *B. pectinophilus* were monotypic and did not display a particularly high similarity to *B. pectinophilus* (Supplementary Data Sheet 1). We conclude that *B. pectinophilus* is best assigned to a genus of its own. However, since the type strain of *B. pectinophilus* appears to have been deposited in a single culture collection only, a new combination for this species name cannot currently be validly published (Parker et al., 2015).

As shown in Table 3, *B. galacturonicus* (Jensen and Canale-Parola, 1986), which was isolated together with *B. pectinophilus* from the human intestinal tract, should be regarded as a later heterotypic synonym of *Lactobacillus rogosae*. However, *L. rogosae* itself appeared taxonomically misplaced and much like *Eubacterium eligens* should better be placed in *Lachnospira* (Bryant and Small, 1956) according to the *Eubacteriales* 16S rRNA gene and GBDP trees (Supplementary Data Sheet 1). The scarcely recorded phenotypic features (Supplementary Table 1) do not preclude this taxonomic solution, which is also more conservative than establishing a new genus for *Lactobacillus rogosae*. Due to the unavailability of the type strain mentioned in the approved list of bacterial names (Skerman et al., 1980) this species, however, does not currently seem to be represented by an extant type strain (Tindall, 2014). While the genome sequence of *Lactobacillus rogosae* was obtained from ATCC 27753, this deposit does not currently appear in the ATCC online catalog. Although our results confirm previous ones based on the 16S rRNA gene (Tindall, 2014), further steps are necessary to clarify the status of the type strain, and we here can only tentatively suggest the name “*Lachnospira rogosae*” for *Lactobacillus rogosae*.

*B. xylanolyticus* (Scholten-Koerselman et al., 1986) was shown as sister group of *Hungatella effluvii* (Kaur et al., 2014) in the *Eubacteriales* GBDP tree with strong support (Supplementary Data Sheet 1), whereas the *Eubacteriales* 16S rRNA gene placed both in a moderately supported clade. Interestingly, flagella were reported for *B. xylanolyticus* (Scholten-Koerselman et al., 1986) but, as we will reiterate below, flagella are unlikely in *Bacteroidetes*, whereas *Hungatella* is motile. It should be noted, however, that the report was apparently not based on a flagellum-specific staining. The last emendation of *Bacteroides* indicated that the genus is non-motile (Shah and Collins, 1989). The 16S rRNA gene clade also included a set of apparently misplaced *Clostridium* species some of which showed high 16S rRNA gene similarities to *B. xylanolyticus* indicating that a DDH value should be obtained to determine species boundaries (Meier-Kolthoff et al., 2013b). While genome sequences were available for all of these *Clostridium* species that were validly published before *B. xylanolyticus*, none of them yielded a dDDH value ≥70% (Supplementary Table 1). *B. xylanolyticus* can safely be placed in *Hungatella* in this respect, which is not in disagreement with the scarcely recorded phenotype (Supplementary Table 1) and also more conservative than establishing a new genus. The nomenclature of the *Clostridium* species misplaced in the same clade, the phenotypically heterogeneous *Clostridium* group XIVa (Collins et al., 1994a), is beyond the scope of the current study, however.

### Non-*Bacteroidetes* Taxa

The outgroup taxa, which were selected in accordance with a previous study (Hahnke R. L. et al., 2016), were phylogenetically located at the expected positions in the newly inferred GBDP tree. For instance, the monophyly of the “*Bacteroidota-Rhodothermota-Balneolota-Chlorobiota* superphylum” could be confirmed (Figure 1). Despite the relatively low size of these taxa, a couple of taxonomic discrepancies were found.

Within the phylum *Nitrospirae*, the dDDH value (Table 3) between *Thermodesulfovibrio yellowstonii* (Henry et al., 1994) and *T. islandicus* (Sonne-Hansen and Ahring, 1999) indicated that *T. islandicus* is later heterotypic synonym of the former.

Because of the position of *Victivallis* (Zoetendal et al., 2003) *Lentisphaerae* (Cho et al., 2004), the phyla *Lentisphaeria* (Cho J. C. et al., 2011) and *Fibrobacteres* (Garrity and Holt, 2001) appeared as non-monophyletic but support against their monophyly was poor (Figure 2). The 16S rRNA gene trees, the ULT and additional supermatrix analyses supported the monophyly of these taxa (Supplementary Data Sheet 1), hence no taxonomic consequences were aimed at because in this respect the GBDP tree simply seemed to be unresolved.

Within the phylum *Deinococcus-Thermus, Deinococcales* (Rainey et al., 1997) appeared as paraphyletic in the GBDP tree because of the position of *Truepera* (Albuquerque et al., 2005) though support to this effect is not high (Figure 2). However, none of the 16S rRNA gene trees supported the monophyly of *Deinococcales* either (Supplementary Data Sheet 1). The ULT showed *Truepera* also branching first within the phylum *Deinococcus-Thermus* with strong support under ML. The current classification already separates *Truepera* in a family of its own (Albuquerque et al., 2005) from *Deinococccus*. Phylogenetically it seems safer to also place *Truepera* into an order of its own, *Trueperales* ord. nov.

Within the phylum *Rhodothermaeota, Rhodothermaceae* (Ludwig et al., 2011a; Hahnke R. L. et al., 2016) appeared as non-monophyletic in the GBDP tree because *Longibacter salinarum* (Xia J. et al., 2016) and *Longimonas halophila* (Xia et al., 2015) were located in a maximally supported clade containing *Salinibacter ruber* (Antón et al., 2002), *Salinivenus iranica* (Makhdoumi-Kakhki et al., 2012; Munoz et al., 2016), and *Salisaeta longa* (Vaisman and Oren, 2009) of *Salinibacteraceae* (Figure 2, Supplementary Data Sheet 1). *Longimonas* and *Longibacter* were not included in the phylogenetic study when *Salinibacteraceae* was proposed (Munoz et al., 2016). *Longibacter* and *Longimonas* differ from the other three genera by their facultatively anaerobic lifestyle and their lack of oxidase activity (Supplementary Table 1). However, the position of *Longimonas* indicates that its character states are plesiomorphic within the clade and hence these two characters cannot be used to separate the five genera into two families. It is thus proposed that *Longibacter* and *Longimonas* be included in *Salinibacteraceae*.

Within the class *Spartobacteria* (Sangwan et al., 2004), to date *Terrimicrobium* (Qiu Y.-L. et al., 2014) was not yet assigned to a family or order. Given its relatively isolated position in the trees with a considerable genomic divergence from its sister group (Figure 2, Supplementary Data Sheet 1), *Terrimicrobium* would best be assigned to a family of its own in *Chthoniobacterales*. However, neither *Spartobacteria* nor its supposed type order *Chthoniobacterales* are validly published names. As type strains of the type species of the type genus of *Chthoniobacterales, Chthoniobacter flavus*, were apparently not deposited in two culture collections in two distinct countries, there does not seem to be an easy remedy (Parker et al., 2015). Thus *Terrimicrobium*, whose name is validly published, is best placed into a family of its own in an order of its own in a class of its own within an accordingly emended phylum *Verrucomicrobia* (Hedlund, 2011) to provide names for higher taxa within this clade with a higher probability of obtaining standing in nomenclature.

### Class *Saprospiria*

The class *Saprospiria* is a relatively small, aerobic group within the phylum *Bacteroidetes* that was only recently recognized (Hahnke R. L. et al., 2016) as a separate group branching first within the phylum, as confirmed here albeit with limited branch support, whereas the monophyly of the class was strongly confirmed (Figure 2; Supplementary Data Sheet 1). While its single order is at present taxonomically subdivided into three families, this arrangement was only partially in agreement with the phylogenomic analysis.

*Haliscomenobacteraceae* (Hahnke R. L. et al., 2016) appeared polyphyletic in the GBDP tree (Figure 2) because *Lewinella nigricans* (Sly et al., 1998; Khan et al., 2007a) of *Lewinellaceae* was placed as sister group of *Phaeodactylibacter xiamenensis* (Chen Z. et al., 2014) of *Haliscomenobacteraceae* with moderate to high support, to the exclusion of the remaining *Lewinella* species. *Haliscomenobacteraceae* and *Lewinellaceae* were recently proposed by splitting *Saprospiraceae*, supported by phylogenomic and 16S rRNA gene sequence analyses and phenotypic features (Hahnke R. L. et al., 2016). Given the position of *Saprospiraceae sensu stricto* in the CCT (Supplementary Data Sheet 1) it was assumed that lack of motility within the order is a synapomorphy of *Haliscomenobacter, Phaeodactylibacter*, and *Portibacter*, hence they were placed in the same family but separate from *Lewinella* (*Lewinellaceae*); support against the monophyly of *Haliscomenobacteraceae* was low. The inclusion of additional *Lewinella* genome sequences, such as the one of the non-motile *L. nigricans*, in the present study partially increased backbone support and yielded a topology that rather indicates that motility is a homoplastic character within the order. Thus, it appears preferable to merge *Haliscomenobacteraceae* and *Lewinellaceae*. The phylogenetic situation is indeed confusing within the order because, unusually, the ULT and URT yielded moderate to strong support for a distinct arrangement including a monophyletic *Haliscomenobacteraceae* and a monophyletic *Lewinella* (Supplementary Data Sheet 1). Additional supermatrix analyses confirmed the GBDP topology but partially low partition bootstrap support indicated that within these two families large sets of genes may have had distinct evolutionary histories. We did not detect any hints of contamination in the genome of *L. nigricans* NBRC 102662^T^, while the SH test did not indicate a significant conflict between the 16S rRNA gene and the supermatrix topology (Supplementary Table 1).

Given the genomic divergence of *Lewinella, L. nigricans* may well be placed in a genus of its own, yielding a classification that would agree with all conflicting tree topologies. Originally, *L. nigricans, L. persica* and the type species of *Lewinella, L. cohaerens*, were reclassified from *Herpetosiphon* (currently assigned to the phylum *Chloroflexi*) because they formed a well-defined and well-supported clade that was only distantly related to the type species of *Herpetosiphon* (Sly et al., 1998). The phenotype of *Haliscomenobacteraceae* and *Lewinellacae* is quite uniform with the exception of motility in *Lewinella* (Supplementary Table 1) but this may be a homoplastic character, in agreement with the findings reported in Table 1. We thus propose to assign *L. nigricans* to a new genus of its own, *Flavilitoribacter* gen, nov., which is also in line with its higher genome size compared to the other *Lewinella* species (Figure 2). It should be noted that the remaining genus *Lewinella* is still genomically quite heterogeneous regarding *L. cohaerens* but further taxonomic consequences do not appear to be absolutely necessary.

### Class *Cytophagia*

The class *Cytophagia* is a largely aerobic subgroup of the phylum *Bacteroidetes*, which here was strongly supported as monophyletic and formed a moderately supported clade together with *Chitinophagia* and *Sphingobacteriia* (Figure 3). Its single order *Cytophagales* displayed a small numbers of discrepancies between the classification and the phylogenomic results at the level of families and genera.

Within *Cytophagales, Cytophagaceae* (Stanier, 1940) appeared as non-monophyletic in the GBDP tree (Figure 3) because *Flexibacter flexilis* (Soriano, 1945; Hahnke R. L. et al., 2016) was placed as sister group of *Mooreia alkaloidigena* (Choi E. J. et al., 2013) of *Mooreiaceae*. Support for this arrangement was low but so was the evidence for the monophyly of *Cytophagaceae* in all inferred trees (Supplementary Data Sheet 1). In contrast, the clade in the CCT corresponding to the clade ranging from *Siphonobacter* to *Spirosoma* in Figure 3 was strongly supported. This group shows a tendency toward larger genomes and higher G+C content values compared to *Cytophaga* and *Sporocytophaga*. It would thus appear reasonable to split *Cytophagaceae* into a total of three families. Whereas, *Flexibacter flexilis* should be placed in a family of its own, an earlier taxonomic concept already proposed the family *Spirosomaceae* to contain *Spirosoma, Flectobacillus*, and *Runella* (Larkin and Borrall, 1978), which all belong to that clade as shown in Figure 3. We thus suggest to reuse the validly published name *Spirosomaceae* as the family for these three genera, in addition to those proposed after 1978 but phylogenetically located within the same clade.

*Flammeovirgaceae* (Yoon J.-H. et al., 2011) appeared as paraphyletic in the GBDP tree (Figure 3) because *Chryseolinea* (Kim J.-J. et al., 2013)*, Ohtaekwangia* (Yoon J.et al., 2011), and *Thermoflexibacter* (Hahnke R. L. et al., 2016) which were not yet assigned to a family, *Ekhidna lutea* (Alain et al., 2010) of *Cytophagaceae*, all *Bernardetiaceae* (Hahnke R. L. et al., 2016) and all *Cyclobacteriaceae* (Nedashkovskaya and Ludwig, 2011) were phylogenetically placed within *Flammeovirgaceae*. The overall genomic divergence of these taxa argues against including all of them in a single family, which would also lack branch support in the tree. In line with the dissection of *Cytophagaceae* proposed above it would thus appear reasonable to split *Flammeovirgaceae*. Given the uncertain position of *Thermoflexibacter* (Hahnke R. L. et al., 2016), which has as yet not been assigned to a family, as sister group of *Bernardetiaceae* (Hahnke R. L. et al., 2016), and the comparatively long genomic distance separating it from that group, *Thermoflexibacter* is best be assigned to a family of its own, which is not contradicted by the scarcely known phenotypic features (Supplementary Table 1). *Cesiribacter* (Srinivas et al., 2011) and *Nafulsella* (Zhang L. et al., 2013) were placed in a strongly supported clade with uncertain affiliations to other clades, indicating the two genera should best be placed in a separate family. *Fulvivirga* (Nedashkovskaya et al., 2007a) formed a clade together with *Chryseolinea* (Kim J.-J. et al., 2013) and *Ohtaekwangia* (Yoon J.et al., 2011), hence a new family can be proposed to accommodate these genera (see also Supplementary Table 1, Supplementary Data Sheet 1). As in the GBDP tree, the 16S rRNA gene trees and the ULT (Supplementary Data Sheet 1) showed *Ekhidna lutea* in a clade together with *Marinoscillum* (Seo et al., 2009) and *Reichenbachiella* (Nedashkovskaya et al., 2005f; Cha et al., 2011), which suggests the classification of these three genera into a new family, which is not precluded by their phenotype (Supplementary Table 1). *Roseivirga* (Nedashkovskaya et al., 2005a,e, 2008a; Selvaratnam et al., 2016) formed a strongly supported clade together with *Fabibacter* (Lau et al., 2006a), with which it was even intermixed as discussed below. The two genera are best placed in a separate family, *Roseivirgaceae* fam. nov. (Supplementary Data Sheet 1).

*Roseivirga* (Nedashkovskaya et al., 2005a,e, 2008a; Selvaratnam et al., 2016) appeared as polyphyletic in the GBDP tree (Figure 3) because *R. spongicola* was placed within a paraphyletic *Fabibacter* (Lau et al., 2006a). In the study that proposed both *Fabibacter* and *R. spongicola* (Lau et al., 2006a), a 16S rRNA gene tree showed *F. halotolerans* placed together with two environmental isolates as sister group of a *Roseivirga* clade that included *R. spongicola* but support for this arrangement was moderate and apparently only obtained in a neighbor-joining analysis. The ULT and additional supermatrix analyses confirmed the GBDP topology instead (Supplementary Data Sheet 1); the URT also but with low support. The SH test indicated a significant conflict between the 16S rRNA gene and the supermatrix topology but the corresponding 16S rRNA gene tree did not show a monophyletic *Roseivirga* either (Supplementary Table 1). *Fabibacter* and the closely related genus *Fabivirga* (Tang M. et al., 2016) show phenotypic characteristics similar to those of *Roseivirga* except for the presence of flexirubin-like pigments in some *Roseivirga* species (Supplementary Table 1). A single binary character cannot properly separate two taxa, however, because it cannot yield an apomorphy for both (Hennig, 1965; Wiley and Lieberman, 2011; Montero-Calasanz et al., 2017). Moreover, the GBDP topology also indicates that production of flexirubins is homoplastic in the *Fabibacter*-*Roseivirga* clade (Supplementary Data Sheet 1), in line with the findings reported above (Table 1). The CCT and UCT (Supplementary Data Sheet 1) do not allow for an unambiguous placement of all species of the two genera, even though it is obvious that the type species of *Fabibacter, F. halotolerans*, is placed within the highly supported clade that also includes *Fabivirga*. For this reason, we propose to assign *Fabibacter* and *Fabivirga* into *Roseivirga*, which has priority, to create a genus that is unambiguously monophyletic in all examined datasets.

*Marivirga* (Nedashkovskaya et al., 2010c; Lin et al., 2015) formed the sister group of *Cyclobacteriaceae* with strong support but was phylogenetically distant from this family and thus may best also be placed into a family of its own, a solution neither contradicted by analyses of rRNA genes (Supplementary Data Sheet 1) nor by the phenotype (Supplementary Table 1). To obtain well-supported families, the only alternative to splitting *Flammeovirgaceae* into that many families is to place most of its genera in *Cyclobacteriaceae* thus create a family covering the genera from *Nafulsella* to *Algoriphagus* in Figure 3. However, low support in the CCT argues against this solution (Supplementary Data Sheet 1). The phylogenomic analysis indeed shows a series of relatively short branches descending from the root of the *Cytophagales* subtree to form an overall not well-supported backbone, which is better in agreement with splitting the order into more families than suggested in the literature.

### Class *Sphingobacteriia*

The class *Sphingobacteriia* here formed a strongly supported clade and appeared as the sister group of the class *Chitinophagia* with weak support (Figure 4). The largely aerobic *Sphingobacteriia* showed some disagreement between the classification and the phylogenomic tree but these were restricted to the single, relatively species-rich genus *Pedobacter*.

Indeed, *Pedobacter* (Steyn et al., 1998; Vanparys et al., 2005; Gallego et al., 2006; Hwang et al., 2006; Zhou et al., 2012; Farfán et al., 2014; Kook et al., 2014; Du et al., 2015) appeared as paraphyletic in the GBDP tree because several species, *P. arcticus* (Zhou et al., 2012), *P. bauzanensis* (Zhang et al., 2010b)*, P. glucosidilyticus* (Luo et al., 2010; Zhou et al., 2012; Hahnke R. L. et al., 2016)*, P. luteus* (Oh et al., 2013), *P. oryza* (Jeon et al., 2009), *P. psychrophilus* (Švec et al., 2017), and *P. ruber* (Margesin and Zhang, 2013) were placed apart from the clade containing the type species, *P. heparinus* (Steyn et al., 1998; Zhou et al., 2012) with high support (Figure 4). The ULT also strongly supported the paraphyly of *Pedobacter*; the URT showed the same albeit with lower support (Supplementary Data Sheet 1). In the CCT, *Pedobacter* also appeared as paraphyletic; here *P. composti* (Lee H.-G. et al., 2009)*, P. huanghensis* (Qiu X. et al., 2014)*, P. luteus, P. oryza*, and *P. ruber* formed an independent and well-supported clade, whereas *P. tournemirensis* (Urios et al., 2013) was placed as sister group of *Arcticibacter* (Prasad et al., 2013) with high support. Both clades appeared more closely related to *Mucilaginibacter* (Pankratov et al., 2007; Urai et al., 2008; Baik et al., 2010; Chen X. Y. et al., 2014) than to *P. heparinus*. Moreover, *P. bauzanensis* appeared as sister group of the clade comprising *Olivibacter* (Ntougias et al., 2007) and *Pseudosphingobacterium* (Vaz-Moreira et al., 2007) with high support. The UCT did not resolve the monophyly of *Pedobacter* (Supplementary Data Sheet 1). This observation is not in conflict with literature statements, since all emendations of the genus, much like its original description (Steyn et al., 1998), used a taxon sampling far too restricted to properly demonstrate whether or not *Pedobacter* is monophyletic. As detailed in Supplementary Table 1, there are no obvious phenotypic differences between *P. tournemirensis* and *Arcticibacter*. Hence, it is proposed that *P. tournemirensis* be assigned to *Arcticibacter*. In fact, obtaining monophyletic genera by merging all genera with which *Pedobacter* is intermixed would almost amount to placing all *Sphingobacteriaceae* in a single genus, which is unwise given the genomic (Figure 4) and phenotypic (Supplementary Table 1) divergence of the group. For this reason, we propose a new genus for accommodating *P. composti, P. huanghensis, P. luteus, P. oryza*, and *P. ruber* and another one to accommodate *P. bauzanensis*. We are aware of the fact that this does not solve all known taxonomic problems in *Pedobacter* because *P. arcticus* (Zhou et al., 2012), *P. glucosidilyticus* (Luo et al., 2010), and *P. psychrophilus* (Švec et al., 2017) are more closely related to *Pseudopedobacter* (Cao et al., 2014) than to *P. heparinus*. However, an accordingly revised genus lacks support even in the CCT (Supplementary Data Sheet 1). In addition, in the 16S rRNA gene tree, *Nubsella zeaxanthinifaciens* (Asker et al., 2008b) and *Pelobium manganitolerans* (Xia X. et al., 2016) were placed within the main *Pedobacter* clade that included *P. heparinus*. However, support for this clade was low. Since genome sequences for *N. zeaxanthinifaciens* and *P. manganitolerans* were not available at the time of writing, we here refrain from considering further taxonomic consequences.

### Class *Chitinophagia*

The only recently proposed class *Chitinophagia* (Munoz et al., 2016) here formed a strongly supported clade and appeared as the sister group of the class *Sphingobacteriia* with weak support (Figure 4). The largely aerobic *Chitinophagia* showed few discrepancies between the classification and the phylogenomic tree, which were restricted to the level of the genera.

The GBDP and rRNA gene trees (Figure 4, Supplementary Data Sheet 1) show that *Crenotalea* (Hanada et al., 2014) and *Thermoflavifilum* (Anders et al., 2014) form a clade which stands out as its genetic divergence is lower than that of adjacent clades, including closely related ones corresponding to individual genera, as exemplified by *Chinitophaga*. The dDDH value between the two species, which were proposed independently at about the same time to belong to two distinct genera, amounted to 37.8%, which is quite high for species assigned to distinct genera. Because *Crenotalea* and *Thermoflavifilum* also show almost identical phenotypic characteristics, including chemotaxonomic markers such as fatty acids (Supplementary Table 1), it is proposed that *Crenotalea* be included in *Thermoflavifilum*, which has priority.

*Sediminibacterium* (Qu and Yuan, 2008; Kim Y.-J. et al., 2013) appeared as paraphyletic in the GBDP tree (Figure 4) because the clade comprising *Hydrobacter penzbergensis* (Eder et al., 2015) and *Vibrionimonas magnilacihabitans* (Albert et al., 2014) was placed as sister group of *S. ginsengisoli* (Kim Y.-J. et al., 2013) with high support. Whereas, the ULT showed a monophyletic *Sediminibacterium* with strong support, additional supermatrix analyses confirmed the GBDP topology with respect to a paraphyletic *Sediminibacterium* (Supplementary Data Sheet 1). The SH test indicated a significant conflict between the 16S rRNA gene and the supermatrix topology (Supplementary Table 1). In the CCT, *H. penzbergensis* and *V. magnilacihabitans* were placed within *Sediminibacterium*, along with *Asinibacterium lactis* (Lee D.-G. et al., 2013), which formed their sister group with high support. When *A. lactis* and *H. penzbergensis* were proposed, only the type species of *Sediminibacterium* was considered for the phylogenetic analyses of 16S rRNA gene sequences, which also yielded low support for the interrelationships between these genera, hence monophyly of *Sediminibacterium* could not be guaranteed further. When *H. penzbergensis* was proposed, *V. magnilacihabitans* was not considered; as shown in Table 3, *H. penzbergensis* is a later heterotypic synonym of *V. magnilacihabitans*. These two genera as well as *Asinibacterium* (Lee D.-G. et al., 2013) and even *Hydrotalea* (Kämpfer et al., 2011b; Albuquerque et al., 2012) and *Parasediminibacterium* (Kang et al., 2016) display phenotypic features similar to those of *Sediminibacterium*, the only known differences being presence or absence of motility, which may well be homoplastic given the findings reported above (Table 1), and a negative response for oxidase and catalase activities reported for *Asinibacterium* (Supplementary Table 1). Consequently, it is proposed that *Asinibacterium lactis* and *Vibrionimonas magnilacihabitans* be classified within *Sediminibacterium*.

### Class *Bacteroidia*

The mainly anaerobic *Bacteroidia* formed a strongly supported clade and appeared as the sister group of the class *Flavobacteriia* with weak support (Figure 4). *Bacteroidia* showed several discrepancies between the classification and the phylogenomic tree, which affected orders, families, or genera.

*Marinilabiliales* (Wu et al., 2016) and *Bacteroidales* (Krieg, 2011b; Pikuta et al., 2017) appeared as paraphyletic in the GBDP tree (Figure 5) for a variety of reasons even if one disregards the completely misplaced *Bacteroides* species discussed above and the placement of *Cytophaga xylanolytica*, which is treated below. For instance, *Balneicella halophila* (Fadhlaoui et al., 2016) of *Balneicellaceae* within *Bacteroidales* was phylogenetically placed within *Marinilabiliales*. When *Balneicella* was proposed, it appeared as neighbor to *Marinifilum* which at that time was assigned to *Bacteroidales* instead of *Marinilabiliales*. As detailed in Supplementary Table 1, according to the respective taxon descriptions *Balneicellaceae* can only be differentiated from *Marinilabiliales* by the tolerance of the former toward oxygen (aerobic or facultatively anaerobic vs. strictly anaerobic) and a distinct major menaquinone (MK-7 vs. MK-6) but the real taxonomic value of these differences is difficult to judge from the taxonomic literature because it was not indicated which state of which character is apomorphic. As implied by the results shown in Table 1 and the GBDP topology, MK-7 is plesiomorphic in *Bacteroidetes*, hence MK-6 is simply an autapomorphy of *Balneicellaceae*; equivalent logic prohibits using the oxygen relationship to separate the two taxa. Moreover, a variety of taxa assigned to *Marinilabiliales* were described as facultatively anaerobic instead of strictly anaerobic (Supplementary Data Sheet 1). *Marinilabiliales* appeared monophyletic neither in the URT nor in the ULT (Supplementary Data Sheet 1). While one could taxonomically assign *Balneicellaceae* to *Marinilabiliales, Marinilabiliales*, and *Bacteroidales* also appeared as phylogenetically intermixed with respect to the position of *Butyricimonas, Gabonibacter, Odoribacter, Rikenellaceae*, and *Williamwhitmaniaceae* (Figure 5, Supplementary Data Sheet 1). Bootstrap support was low in the 16S rRNA tree that was used for proposing *Marinilabiales* (Wu et al., 2016). Likewise, within the GBDP tree, the support for the backbone of the *Bacteroidia* subtree was low except for *Bacteroidales sensu stricto*. In this respect, the phylogenetically safest solution is to not retain *Marinilabiliales* and to place all *Bacteroidia* families within *Bacteroidales*. This also provides a taxonomically conservative solution for *Lentimicrobiaceae* (Sun et al., 2016), which was as yet not assigned to any order or class and can now be included in *Bacteroidales*.

*Rikenellaceae* (Krieg et al., 2010b) appeared as paraphyletic in the GBDP and 16S rRNA gene trees (Figure 5, Supplementary Data Sheet 1) because *Williamwhitmania taraxaci* (Pikuta et al., 2017) of *Williamwhitmaniaceae* was placed as a sister group of *Acetobacteroides hydrogenigenes* (Su et al., 2014) of *Rikenellaceae* with strong support. When *Williamwhitmania* was proposed, *Acetobacteroides* was not considered in the phylogenetic analysis. *Rikenellaceae* and *Williamwhitmania* share many of their phenotypic properties except for the cellular motility of *Williamwhitmania* (Supplementary Table 1). Motility is incompletely reported for these genera; if all except *Williamwhitmania* were non-motile, being non-motile would be plesiomorphic and could not be used to justify a taxon. According to the results reported above (Table 1) the character may also be homoplastic. The clade comprising both *Williamwhitmania* and *Rikenellaceae* shows lower support than its descendant clades, hence it is phylogenetically preferable to place *Acetobacteroides* in *Williamwhitmaniaceae*.

*Marinifilaceae* (Iino et al., 2014) was recently emended (Ormerod et al., 2016) to include *Odoribacter* (Hardham et al., 2008) and *Butyricimonas* (Sakamoto et al., 2009b, 2014), previously classified within *Porphyromonadaceae*. Concurrently, another study (Munoz et al., 2016) proposed the new family *Odoribacteraceae* within *Bacteroidales* to contain *Odoribacter* and *Butyricimonas*, a family that was emended later on (Hahnke R. L. et al., 2016). According to our analysis, *Butyricimonas synergistica* (Sakamoto et al., 2009b, 2014; Hahnke R. L. et al., 2016), *B. virosa* (Sakamoto et al., 2009b, 2014; Hahnke R. L. et al., 2016), *Odoribacter laneus* (Nagai et al., 2010; Hahnke R. L. et al., 2016), and *O. splanchnicu*s (Hardham et al., 2008; Hahnke R. L. et al., 2016) form a well-supported group together with *Gabonibacter massiliensis* (Mourembou et al., 2016) set apart from *Marinifilaceae*, which was strongly supported as sister group of *Balneicellaceae* (Figure 5). *Marinifilaceae* also appeared as paraphyletic in the UCT and ULT (Supplementary Data Sheet 1). Therefore, our results corroborate the earlier proposal (Munoz et al., 2016) and do not support the alternative taxonomic framework proposed later on (Ormerod et al., 2016), which may have been caused by insufficient taxon sampling. *Gabonibacter massiliensis*, which is currently taxonomically placed in *Porphyromonadaceae*, presents phenotypic features similar to those of *Odoribacteraceae* with the exception of being motile (Supplementary Table 1). Motility is incompletely reported for these genera, however, and, given the phylogenetic position of *Gabonibacter*, non-motility would be plesiomorphic in the clade if all other genera were non-motile. Yet a single character with two states should indeed never be used to separate two taxa (Hennig, 1965; Wiley and Lieberman, 2011; Montero-Calasanz et al., 2017). It would not be surprising either if being motile was a homoplastic character in this group (Table 1). We thus propose to place *Gabonibacter* in *Odoribacteraceae*.

*Labilibacter* (Lu et al., 2017) appeared as paraphyletic in the GBDP and rRNA gene trees (Figure 5, Supplementary Data Sheet 1) because *Saccharicrinis* (Liu et al., 2014b; Yang S.-H. et al., 2014) formed the sister group of *L. marinus* (Lu et al., 2017) with high support. While originally placed in *Saccharicrinis* as *S. marinus* (Liu Q.-Q. et al., 2015), it had been reclassified in *Labilibacter* in a study in which a 16S rRNA gene tree showed it to form a clade together with the type species of *Labilibacter*, in contrast to our phylogenetic analyses. Some phenotypic differences such as temperature range for growth and fatty acids (Supplementary Table 1) were proposed as suggestive of separating the two genera (Lu et al., 2017) but it was not clarified which character states were apomorphies of which taxon. The known polar-lipid spectra are basically identical between the two genera. While the URT showed the same topology as the previously published 16S rRNA gene analyses, additional supermatrix analyses unambiguously confirmed *L. marinus* as more closely related to *Saccharicrinis* than to *L. aurantiacus* (Supplementary Data Sheet 1). The SH test indicated a significant conflict between the 16S rRNA gene and the supermatrix topology (Supplementary Table 1). In the case of *L. marinus*, this simply implies the synonym *S. marinus* should be preferred. However, since here the 16S rRNA gene is in significant conflict with genome-scale data, which is highly unusual, we also propose to include the entire genus *Labilibacter* in *Saccharicrinis* in order to allow 16S rRNA gene trees to agree with the taxonomic classification.

*Cytophaga xylanolytica* (Haack and Breznak, 1993) of *Cytophagaceae* was phylogenetically placed within a paraphyletic *Marinilabiliaceae* where it formed a relatively isolated lineage (Figure 5, Supplementary Data Sheet 1), which also made *Cytophaga* (Winogradsky, 1929; Nakagawa and Yamasato, 1996) appear as paraphyletic. *C. xylanolytica* was originally proposed on basis of phenotypic characteristics without taking into account the 16S rRNA gene sequence as phylogenetic marker (only few “signature nucleotides” were examined). Later 16S rRNA gene analyses indicated that the species should better not be placed in *Cytophaga* (Nakagawa, 2011b). In a comparison with other currently accepted taxa *C. xylanolytica* displays phenotypic characteristics similar to those of *Marinilabiliaceae* (Supplementary Table 1) but apparently does not phylogenetically belong to any of its known genera. Thus, it is proposed that *C. xylanolytica* be assigned to a new genus, *Breznakibacter* gen. nov., within *Marinilabiliaceae*.

*Dysgonomonadaceae* (Ormerod et al., 2016) appeared as polyphyletic in the GBDP and rRNA gene trees (Figure 5, Supplementary Data Sheet 1) because *Fermentimonas* (Hahnke S. et al., 2016) and *Petrimonas* (Grabowski et al., 2005), both classified in *Porphyromonadaceae*, were placed as a sister group of *Proteiniphilum* (Chen and Dong, 2005; Hahnke S. et al., 2016) of *Dysgonomonadaceae* with maximum support. When the name *Dysgonomonadaceae* was proposed, *Petrimonas* was not considered, while *Fermentimonas* was proposed independently at about the same time as *Dysgonomonadaceae*. Our suggestion to transfer *Fermentimonas* (Hahnke S. et al., 2016) and *Petrimonas* (Grabowski et al., 2005) to *Dysgonomonadaceae* (Ormerod et al., 2016) is not contradicted by the phenotype (Supplementary Table 1). To date the name *Dysgonomonadaceae* does not seem to have been validated, which may be caused by a formally incomplete description (Parker et al., 2015), which we attempt to fix below.

*Muribaculum* (Lagkouvardos et al., 2016) has as yet not been formally assigned to a family even though its authors suggested the not validly published name “*Muribaculaceae*.” Given its position as sister group of *Barnesiellaceae* in the GBDP tree (Figure 5) and its position within *Barnesiallaceae* in the UCT, ULT, and URT (Supplementary Data Sheet 1) we propose to place it in the family *Barnesiellaceae*. While the UCT and URT even showed a paraphyletic *Coprobacter* (Shkoporov et al., 2013) with relatively strong support due to the position of *Muribaculum*, additional supermatrix analyses confirmed the GBDP topology, and the SH tests did not indicate a significant conflict between the 16S rRNA gene and the supermatrix topology. However, not establishing a new family for *Muribaculum* is the taxonomically more conservative solution in either case. To date the name *Barnesiellaceae* does not seem to have been validated, which may be caused by a formally incomplete description (Parker et al., 2015), which we attempt to fix below.

*Prevotellaceae* (Krieg, 2010) appeared as polyphyletic in the GBDP tree (Figure 5) because *Prevotella zoogleoformans* (Weinberg et al., 1937; Shah and Collins, 1990; Moore et al., 1994) was placed within *Bacteroides* of *Bacteroidaceae*. In the CCT and the UCT (Supplementary Data Sheet 1) *P. heparinolytica* (Okuda et al., 1985; Shah and Collins, 1990) formed the sister group of *P. zoogleoformans* while the clade comprising both was placed as sister group to *B. helcogenes* (Benno et al., 1983; Hahnke R. L. et al., 2016)*. P. heparinolytica* and *P. zoogleoformans* were removed from *Bacteroides* in an earlier study (Shah and Collins, 1990) based on their morphological, physiological, and biochemical characteristics but without taking into account 16S rRNA gene sequences. Whereas, this had been noted as problematic early on (Olsen and Shah, 2001), the later taxonomic literature was undecided on whether placing the two species in *Bacteroides* (Willems and Collins, 1995) or *Prevotella* (Olsen and Shah, 2008). Phylogenomic analyses rather clearly indicate that *Prevotella heparinolytica* and *P. zoogleoformans* should better retain their earlier names *Bacteroides heparinolyticus* and *B. zoogleoformans*.

Even aside from these two *Prevotella* species and the four completely misplaced *Bacteroides* species treated above, *Bacteroides* (Castellani and Chalmers, 1919; Shah and Collins, 1989) appeared as non-monophyletic in the GBDP tree (Figure 5) because *Phocaeicola abscessus* (Al Masalma et al., 2009) was placed within *Bacteroides*. In the original description of *Phocaeicola abscessus*, the type strain of the species was grouped with three environmental clones, along with *Prevotella* species, but supported by a low bootstrap value, as sister group of a couple of *Bacteroides* species. However, the type species of *Bacteroides, B. fragilis*, was not included in the phylogenetic analysis. According to our results, *B. barnesiae, B. caecicola, B. caecigallinarum, B. chinchillae, B. coprocola B. coprophilus, B. dorei, B. gallinaceum, B. massiliensis, B. paurosaccharolyticus, B. plebeius, B. salanitronis, B. sartorii, B. vulgatus* are phylogenetically more closely related to *Phocaeicola* than to *B. fragilis* (Supplementary Data Sheet 1). We did not find a consistent phenotypic difference between *Phocaeicola* and these *Bacteroides* species except for cell motility in the former (Supplementary Table 1). While cells of *Phocaeicola* were reported to produce flagella, we would interpret the structure shown in the original picture, which was apparently not based on a flagellum-specific staining, rather as exopolysaccharides, which, in contrast to flagella, are well-known from *Bacteroidetes* (Krieg et al., 2010a). Moreover, even if *Phocaeicola* was motile, given the tree topology motility would be an autapomorphy of *Phocaeicola*, whereas being non-motile would be plesiomorphic and hence could not be used to justify the current separation of *Phocaeicola* from *Bacteroides* (Hennig, 1965; Wiley and Lieberman, 2011; Montero-Calasanz et al., 2017). It can neither be ruled out that motility was a homoplastic character in this group (Table 1). Regarding the overall genomic divergence of *Bacteroides*, particularly compared to other genera of *Bacteroidetes* (Figure 1), it does not make much sense to merge *Bacteroides* with *Phocaeicola*. Thus, we propose to assign the *Bacteroides* species listed above to *Phocaeicola*, which in turn should be included in *Bacteroidaceae*. The UCT, ULT, and URT do not fully resolve the position of *Phocaeicola* but show this genus together with the deviating *Bacteroides* species as more closely related to *Prevotellaceae* than to *B. fragilis* (Supplementary Data Sheet 1). Because additional supermatrix analyses confirmed the GBDP topology regarding the relationship between *Phocaeicola* and *Bacteroidaceae* and SH tests did not indicate a significant conflict between supermatrix and 16S rRNA gene trees (Supplementary Table 1), placing the genus into this family appears appropriate.

*Hallella seregens* (Moore and Moore, 1994; Hahnke R. L. et al., 2016) is known as a later heterotypic synonym of *Prevotella dentalis* (Willems and Collins, 1995; Hahnke R. L. et al., 2016). Accordingly, the two type strains displayed a >79% dDDH similarity (Table 3).

### Class *Flavobacteriia*

The mainly aerobic *Flavobacteriia* were shown as monophyletic with strong support and appeared as the sister group of the class *Bacteroidia* with weak support (Figure 4). *Flavobacteriia*, which are at present not subdivided into distinct orders, showed a variety discrepancies between the classification and the phylogenomic tree, mainly with regard to genus boundaries but also with respect to one family.

In fact, *Flavobacteriaceae* (Reichenbach, 1989; Bernardet et al., 1996, 2002) appeared as paraphyletic in the GBDP tree, ULT, and URT (Figures 6–8, Supplementary Data Sheet 1) because *Ichthyobacterium* of *Ichthyobacteriaceae* (Takano et al., 2016) was placed as sister group to one of the two major clades into which *Flavobacteriaceae* appeared to be split. The overall genomic divergence also supports splitting the species-rich and genus-rich family *Flavobacteriaceae* by assigning the clade that contains genera ranging from *Ornithobacterium* to *Chryseobacterium* in Figure 6 to a separate family. Whereas, the remaining *Flavobacteriaceae* are frequently motile, this distinct clade is almost entirely non-motile; this alone would not be an argument for the separation (Table 1) but other phenotypic features do not provide evidence against the split (Supplementary Table 1). We thus propose *Weeksellaceae*, fam. nov., to accommodate those *Flavobacteriaceae* that did not form a clade together with the type genus, *Flavobacterium*, to the exclusion of *Ichthyobacterium*. The clade corresponding to *Weeksellaceae* obtained strong support in the CCT and even in the UCT (Supplementary Data Sheet 1); only *Spongiimonas* (Yoon J. et al., 2013) is apparently more difficult to assign to a family within *Flavobacteriales* with 16S rRNA gene data; it is strongly supported as belonging to *Weeksellaceae* fam. nov. only under MP and thus tentatively also assigned to the family.

*Chryseobacterium* (Vandamme et al., 1994; Kämpfer et al., 2009; Wu et al., 2013; Montero-Calasanz et al., 2014; Chen et al., 2015; Hahnke R. L. et al., 2016) appeared as paraphyletic in the GBDP tree (Figure 6) because *Soonwooa buanensis* (Joung et al., 2010) was placed within it. *S. buanensis* and *S. purpurea* (Sirra et al., 2017) were also clustered within *Chryseobacterium* in the 16S rRNA gene tree but with low support (Supplementary Data Sheet 1). When *Soonwooa* was proposed (Joung et al., 2010), many *Chryseobacterium* species were omitted from the 16S rRNA gene tree, which may have caused the two genera to appear as separate. Based on our comprehensive analysis we conclude that *Soonwooa buanensis* and *S. purpurea* should be classified in *Chryseobacterium. Soonwooa* (Joung et al., 2010; Sirra et al., 2017) and *Chryseobacterium* (Vandamme et al., 1994; Kämpfer et al., 2009; Wu et al., 2013; Montero-Calasanz et al., 2014; Chen et al., 2015; Hahnke R. L. et al., 2016) cannot be differentiated based on their phenotypic characteristics (Supplementary Table 1) either. We are aware of the fact that even with this modification the taxonomic classification of *Chryseobacterium* remains unsatisfactory because its relationships to the most closely related genera are not fully resolved (Figure 6). However, the comprehensive 16S rRNA gene trees are even less resolved (Supplementary Data Sheet 1), which would preclude robust assignment of species after splitting *Chryseobacterium* into more narrowly defined genera. As more genome sequences become available, it may be possible to restore some genera that have been incorporated into *Chryseobacterium*, such as *Epilithonimonas* (O'Sullivan et al., 2006), *Kaistella* (Kim et al., 2004), *Planobacterium* (Peng et al., 2009), and *Sejongia* (Yi et al., 2005).

As for the species boundaries in this clade (Figure 6), based on the dDDH values (Table 3) *Chryseobacterium greenlandense* (Loveland-Curtze et al., 2010; Hahnke R. L. et al., 2016) and *Elizabethkingia endophytica* (Kämpfer et al., 2015b) should better be classified as *Chryseobacterium aquaticum* subsp. g*reenlandense* comb. nov., *Elizabethkingia anophelis* subsp. *endophytica* comb. nov., respectively.

*Flavobacterium* (Bergey et al., 1923; Bernardet et al., 1996; Dong et al., 2013; Kang et al., 2013; Kuo et al., 2013) appeared as paraphyletic in the GBDP tree (Figure 7). *Flexibacter aurantiacus* (Lewin, 1969), which was placed as sister group of *Flavobacterium johnsoniae* (Bernardet et al., 1996; Kim et al., 2012; Chen et al., 2013a) had already been proposed as heterotypic synonym of *F. johnsoniae* in an earlier study (Bernardet et al., 1996). According to the dDDH value (Table 3) *F. aurantiacus* is best assigned to a subspecies of *F. johnsoniae*. Moreover, *Myroides* (Vancanneyt et al., 1996; Yan et al., 2012) was placed within *Flavobacterium*, as sister group of the clade comprising *F. marinum* (Song et al., 2013) and *F. ummariense* (Lata et al., 2012). The ULT confirmed *Flavobacterium* as paraphyletic (Supplementary Data Sheet 1). In the CCT, *F. anatoliense* (Kacagan et al., 2013)*, F. ceti* (Vela et al., 2007; Lata et al., 2012; Kacagan et al., 2013), and *F. cloacae* (Liu et al., 2017) were also more closely related to *Myroides* than to the type species, *F. aquatile*. When the species *F. anatoliense, F. marinum*, and *F. ummariense* were proposed, *Myroides* was not considered in the phylogenetic analysis. In the original description of *F. ceti* and *F. cloacae*, several *Myroides* species were included in the phylogenetic analysis, but here *Flavobacterium* already appeared paraphyletic. More recently published 16S rRNA gene analyses show the same pattern but with low support (Hahnke et al., 2015). *Flavobacterium* (Bernardet and Bowman, 2006) and *Myroides* (Hugo et al., 2006) do not display consistent phenotypic differences from each other (Supplementary Table 1). For instance, it is not surprising that motility is a homoplastic character in this group (Table 1). While *Myroides* was originally described as non-motile (Vancanneyt et al., 1996) the last emendation took into account that *M. profundi* is motile by gliding (Yan et al., 2012). Motility is known to be variable in *Flavobacterium* (Bernardet et al., 2002). Merging *Myroides* and *Flavobacterium* does not seem to be advisable given the considerable overall genomic divergence of the group (Figure 7). Therefore, we propose the reclassification of *F. anatoliense, F. ceti, F. cloacae, F. marinum*, and *F. ummariense* into an emended genus *Myroides*, which is taxonomically more conservative than placing these species into a new genus.

As for the species boundaries in this clade, the dDDH values (Table 3) indicated that *Myroides xuanwuensis* (Zhang Z.-D. et al., 2014) should better be classified as *M. odoratimimus* subsp. x*uanwuensis* comb. nov. Moreover, *Flavobacterium spartansii* (Loch and Faisal, 2014b) appeared as later heterotypic synonym of *F. tructae* (Zamora et al., 2014).

*Muricauda* (Bruns et al., 2001; Yoon et al., 2005; Hwang et al., 2009) appeared as paraphyletic in the GBDP tree (Figure 7) because *Flagellimonas* (Bae et al., 2007; Yoon and Oh, 2012) and *Spongiibacterium* (Yoon and Oh, 2012; Gao X. et al., 2015) were placed as sister group to *M. pacifica* (Zhang et al., 2015a) with moderate support and within *Muricauda* with maximum support. The 16S rRNA gene tree in the original description of *M. pacifica* was already unresolved regarding *Muricauda* monophyly, much like the UCT, ULT and URT (Supplementary Data Sheet 1). Phenotypic differences between *Spongiibacterium flavum, S. pacificum, Flagellimonas eckloniae*, and *Muricauda* are not pronounced (Supplementary Table 1). Although *F. eckloniae* (Bae et al., 2007) was originally described as motile by means of a single flagellum, in contrast to the other genera, this conclusion was not based on a direct inspection of the flagellum through a specific staining. Given the general lack of flagella in *Bacteroidetes* and the published micrograph we interpret the structure formed by *F. eckloniae* as an appendage. In the light of these results, we propose to include *Flagellimonas* and *Spongiibacterium* in *Muricauda*, which has priority. This solution appears to be preferable compared to a recent proposal that only transferred *Spongiibacterium* to *Flagellimonas* (Choi et al., 2018). Additional supermatrix analyses indeed confirmed that *Flagellimonas* and *Spongiibacterium* are nested within *Muricauda*, whereas *Croceitalea* (Lee et al., 2008; Yoon and Oh, 2012; Su et al., 2017) and *Croceivirga* (Hu et al., 2017) form independent lineages (Supplementary Data Sheet 1). Whereas, the SH tests indicated a significant conflict between the best supermatrix and best 16S rRNA gene ML trees, the latter also showed *Spongiibacterium* and *Flagellimonas* nested within *Muricauda* (Supplementary Table 1).

*Maribacter* (Nedashkovskaya et al., 2004a, 2010a; Barbeyron et al., 2008; Lo et al., 2013; Weerawongwiwat et al., 2013; Hu et al., 2015; Jackson et al., 2015) appeared as paraphyletic in GBDP tree (Figure 7) because *Maribacter polysiphoniae* (Nedashkovskaya et al., 2007d) and *Maribacter vaceletii* (Jackson et al., 2015) were placed apart from the main *Maribacter* clade that included the type species, *M. sedimenticola*. In the CCT, *Maripseudobacter aurantiacus* (Chen et al., 2017) and *Pibocella ponti* (Nedashkovskaya et al., 2005b) appeared nested within *Maribacter*. But low support even in the CCT precluded identifying better genus boundaries, hence the taxonomy of this genus will need to be revisited once more genome sequences become available.

*Aequorivita* (Bowman and Nichols, 2002; Park et al., 2009; Hahnke R. L. et al., 2016) appeared as paraphyletic in the GBDP and rRNA gene trees (Figure 8, Supplementary Data Sheet 1) because *Vitellibacter aquimaris* (Thevarajoo et al., 2016) was placed as sister group of *A. vladivostokensis* (Hahnke R. L. et al., 2016)*. V. aquimaris* was proposed at about the same time as the reclassification of the entire genus *Vitellibacter* in *Aequorivita* in an earlier study (Hahnke R. L. et al., 2016). We did not detect any pronounced phenotypic differences between *V. aquimaris* and *Aequorivita* (Supplementary Table 1) while the dDDH similarity between the two strains (39.9%) did not indicate that *V. aquimaris* and *A. vladivostokensis* belong to the same species. Consequently, it is proposed that *V. aquimaris* be classified in *Aequorivita*.

Their high dDDH similarity indicates that *Nonlabens sediminis* (Yi and Chun, 2012) is a later heterotypic synonym of *N. tegetincola* (Lau et al., 2005). Moreover, according to the dDDH value (Table 3), *Mesoflavibacter sabulilitoris* (Park et al., 2014) should better be classified as *Mesoflavibacter zeaxanthinifaciens* subsp. *sabulilitoris* comb. nov.

*Mesoflavibacter* (Asker et al., 2007) appeared as paraphyletic in GBDP tree (Figure 8) because the type species of the genus, *M. zeaxanthinifaciens* (Asker et al., 2007), along with *M*. *sabulilitoris* (Park et al., 2014) were more closely related to *Olleya* (Nichols et al., 2005) than to *M. aestuarii* (Lee J. H. et al., 2014). When *M. aestuarii* and *M*. *sabulilitoris* were proposed, *Mesoflavibacter* appeared as monophyletic and as sister group to *Olleya* and *Lacinutrix*, but bootstrap support was low. Similarly, the ULT and URT do not resolve the monophyly of *Mesoflavibacter* (Supplementary Data Sheet 1). Genomically, *M. aestuarii* appears to be quite distant from *M. zeaxanthinifaciens*, and the GBDP topology would imply to place species from four genera into a single one to keep *M. aestuarii* and *M. zeaxanthinifaciens* in the same monophyletic genus. Thus, it is proposed to place *M. aestuarii* into a genus of its own.

*Bizionia* (Nedashkovskaya et al., 2005d; Li et al., 2015; Shakeela et al., 2015b) appeared as polyphyletic in the GBDP tree (Figure 8) because *B. echini* (Nedashkovskaya et al., 2010b) and *B. argentinensis* (Bercovich et al., 2008) were obviously more closely related to *Hanstruepera neustonica* (Hameed et al., 2015) than to the type species *B. paragorgiae* (Nedashkovskaya et al., 2005d). In the 16S rRNA gene tree (Supplementary Data Sheet 1), these two deviating *Bizionia* species formed a well-supported clade together with *B. algoritergicola* (Bowman and Nichols, 2005)*, B. hallyeonensis* (Yoon J.-H. et al., 2013)*, B. myxarmorum* (Bowman and Nichols, 2005)*, B. psychrotolerans* (Song et al., 2014), and *B. sediminis* (Zhang et al., 2017). Whereas, this clade was usually well-supported, support for the monophyly of the entire genus *Bizionia* was already lacking in the published 16S rRNA gene trees used for describing the most recent species. Obtaining monophyletic genera by merging all intermixed genera with *Bizionia* would amount to a huge number of changes and also seems inappropriate given the genomic (Figure 8) and phenotypic (Supplementary Table 1) divergence of the group. It would also be unwise to place the aberrant *Bizionia* species in *Hanstruepera* because further genera may be placed more closely to either group (Supplementary Data Sheet 1). Therefore, a new genus is proposed to accommodate *B. algoritergicola, B. argentinensis, B. echini, B. hallyeonensis, B. myxarmorum, B. psychrotolerans*, and *B. sediminis*. It should be noted that this may not solve all taxonomic problems in the genus because the phylogenetic placement of *B. arctica* (Li et al., 2015) is uncertain relative to the well-supported clade of remaining species that includes *B. paragorgiae* (Supplementary Data Sheet 1). However, alternative placements are uncertain as well, hence we refrain from further taxonomic consequences until the genome sequence of *B. arctica* becomes available.

*Lacinutrix* (Bowman and Nichols, 2005; Nedashkovskaya et al., 2016) appeared polyphyletic in the GBDP tree (Figure 8) because *L. himadriensis* (Srinivas et al., 2013) was more closely related to *Oceanihabitans sediminis* than to *L. algicola* (Nedashkovskaya et al., 2008b), *L. mariniflava* (Nedashkovskaya et al., 2008b), *L. jangbogonensis* (Lee Y. M. et al., 2014), and *L. venerupis* (Lasa et al., 2015), which formed a well-supported clade. While a genome sequence for the type strain of the type species, *L. copepodicola* (Bowman and Nichols, 2005), was not available at the time of writing, the CCT confidently placed it as sister group of *L. himadriensis*. The phylogenetically separate species *L. algicola, L. mariniflava, L. jangbogonensis*, and *L. venerupis* display similar phenotypic features (Supplementary Table 1) and thus can well be placed in a separate genus. *L. iliipiscaria* (Nedashkovskaya et al., 2016) may also belong to that group even though support for this arrangement was low. *L. iliipiscaria* had been reclassified from *Flavirhabdus* (Shakeela et al., 2015b) into *Lacinutrix* based on a poorly resolved 16S rRNA gene tree. For this reason, *Flavirhabdus* may be the appropriate genus name for the five deviating *Lacinutrix* species but this conclusion must be postponed until the genome sequence of the key species *L. iliipiscaria* becomes available.

*Formosa* (Ivanova et al., 2004; Nedashkovskaya et al., 2006b; Shakeela et al., 2015b) appeared as paraphyletic in the GBDP tree (Figure 8) because *Formosa spongicola* (Yoon and Oh, 2011) was confidently placed as sister group of *Xanthomarina gelatinilytica* (Vaidya et al., 2015). When *X. gelatinilytica* was proposed, a 16S rRNA gene tree was presented in which it appeared as the sister group of an unresolved clade comprising *Formosa* and some of the included *Bizionia* species. The ULT and additional supermatrix analyses confirmed the GBDP topology instead, while the CCT, UCT, and URT were unresolved (Supplementary Data Sheet 1). The SH test indicated a significant conflict between the 16S rRNA gene and the supermatrix topology. *F. spongicola* and *Xanthomarina* (Yoon and Oh, 2011; Vaidya et al., 2015) show similar phenotypic characteristics with the exception of motility in *F. spongicola* (Supplementary Table 1). However, as emphasized elsewhere (Hennig, 1965; Wiley and Lieberman, 2011; Montero-Calasanz et al., 2017) a single character with two states is insufficient to properly separate two taxa because it cannot provide an apomorphy for both. Moreover, motility could be a homoplastic character in this clade (Table 1). We thus propose to include *F. spongicola* in *Xanthomarina*.

*Algibacter* (Nedashkovskaya et al., 2004b, 2007c; Park et al., 2013d; Shakeela et al., 2015b) appeared as non-monophyletic in the GBDP and rRNA gene trees (Figure 8, Supplementary Data Sheet 1) since distinct *Algibacter* species were grouped with genera such as *Arenitalea* (Zhang X.-Y. et al., 2013), *Confluentibacter* (Park et al., 2016; Han et al., 2017b), *Flaviramulus* (Einen and Øvreås, 2006; Zhang Y. et al., 2013), *Flavivirga* (Yi et al., 2012; Shakeela et al., 2015b), and *Tamlana* (Lee, 2007; Jeong et al., 2013b) rather than with each other. *Flaviramulus* and *Tamlana* neither appeared as monophyletic. However, due to the low support of the branches, particularly in the comprehensive 16S rRNA gene trees (Supplementary Data Sheet 1) it would be difficult to infer taxonomic conclusions at the moment. Additional supermatrix analyses did not provide more resolution either. We suppose that a more satisfying classification of these intermixed genera can only be obtained once more genome sequences become available.

## Discussion

### Causes of Conflict Between Phylogenomic Analyses and Taxonomic Classification

Most of the taxa of *Bacteroidetes* already appeared monophyletic in this study and not in need of a taxonomic revision, which may reflect recent taxonomic proposals already based on at least multi-gene if not phylogenomic datasets (Hahnke R. L. et al., 2016; Munoz et al., 2016; Ormerod et al., 2016). Despite recent reclassifications at the phylum level (Horino et al., 2014; Verbarg et al., 2014; Ben Hania et al., 2016), some species taxonomically assigned to *Bacteroidetes* were still encountered in this study that are phylogenetically placed in a distinct phylum. The problematic classification of *Bacteroides* species and other Gram-negative anaerobic rods that were described prior to the availability of 16S rRNA gene sequencing has been noted early on (Olsen and Shah, 2001). With the exception of *B. pectinophilus*, we here were able to propose better taxonomic solutions for the four remaining strongly deviating *Bacteroides* species, which are not the first ones to be transferred to *Firmicutes* (Gharbia et al., 2012). However, it should be noted that according to 16S rRNA gene sequences four *Flavobacterium* species, *F. acidificum* (Steinhaus, 1941), *F. devorans* (Bergey et al., 1923), *F. thermophilum* (Loginova and Egorova, 1978), and *F. oceanosedimentum* (Carty and Litchfield, 1978) also do not seem to belong to *Bacteroidetes* phylogenetically (Supplementary Data Sheet 1) albeit taxonomically. As they lacked a genome sequence at the time of writing, we considered them beyond the scope of the current study. Much like the four deviant *Bacteroides* species, these *Flavobacterium* species are comparatively old and their taxonomic proposals were not accompanied by phylogenetic analyses.

Within *Bacteroidetes*, lack of a phylogenetic analysis in the original description also accounted for taxa misplaced such as *Cytophaga xylanolytica* (Haack and Breznak, 1993) as well as *Prevotella heparinolytica* and *P. zoogleoformans* (Shah and Collins, 1990). However, most of the taxonomic discrepancies observed within the phylum appeared to be caused by low resolution of the 16S rRNA genes used to propose the respective taxa. This held for the genera *Algibacter, Bizionia, Fabibacter, Formosa, Lacinutrix, Maribacter, Mesoflavibacter, Muricauda*, and *Roseivirga* as well as the families or orders *Cytophagaceae, Deinococcales, Flavobacteriaceae* and *Marinilabiliales*. Calculating branch support in an appropriate manner is obviously a necessary, but not a sufficient prerequisite, for safely generating monophyletic taxa. Additionally, taxa must also be chosen from trees so as to correspond to highly supported clades (Vences et al., 2013).

However, actually non-monophyletic taxa may easily appear monophyletic when species or strains of relevance are omitted from phylogenetic analysis. The second most important cause of non-monophyly in *Bacteroidetes* detected in the current study was incomplete taxon sampling, which affected *Aequorivita* vs. *Vitellibacter, Bacteroides* vs. *Phocaeicola, Crenotalea* vs. *Thermoflavifilum, Flavobacterium* vs. *Myroides, Soonwooa* vs. *Chryseobacterium*, the recent emendation of *Marinifilaceae* (Ormerod et al., 2016) and the monophyly of *Pedobacter, Salinibacteraceae*, and *Williamwhitmaniaceae*. Regarding *Aequorivita* vs. *Vitellibacter, Crenotalea* vs. *Thermoflavifilum*, and the composition of *Salinibacteraceae* conflicting taxonomic views were published at about the same time, respectively, hence the discrepancies were difficult to avoid.

More worrying are observations of conflicting topologies between 16S rRNA gene and phylogenomic analyses with significant support. This study identified five cases in which the supermatrix ML topology was significantly (α = 0.01) worse than the best ML topology inferred from the 16S rRNA gene in an SH test (Supplementary Table 1). Three of these cases affected taxonomic proposals made in the past, namely regarding the monophyly of *Formosa*, the monophyly of *Labilibacter* and *Saccharicrinis*, and the monophyly of *Sediminibacterium*. Insufficient taxon sampling and misspecified models are potential causes for the probably incorrect 16S rRNA gene phylogenies but we cannot rule out that in these cases the 16S rRNA gene truly conflicts with the organism tree. While conflicts regarding the monophyly of taxa between 16S rRNA gene and supermatrix analyses were not detected in a previous investigation that used exactly the same methods (Hahnke R. L. et al., 2016), the considerably enlarged genome sampling of the present study made it more likely to observe at least some discrepancies of this kind. An analogous results was recently obtained for the phylum *Actinobacteria* (Nouioui et al., 2018).

Compared to the overall number of taxa and the more common causes of taxonomic conflicts as listed above, real conflict between the 16S rRNA gene and entire genomes (and thus between UCT and CCT) indeed appears to be rare. As taxonomic problems caused by insufficient taxon sampling are probably much more common, we believe analyzing comprehensive sets of 16S rRNA gene sequences to be necessary unless all type strains are covered by genome sequences. In this context, using a backbone constraint is a valuable means for integrating information from analyses of more genes but fewer organisms into comprehensible sampled single-gene data (Liu X.-Z. et al., 2015; Hahnke R. L. et al., 2016; Nouioui et al., 2018). For instance, the CCT allowed us to safely place type species lacking genome sequences. The CCT was also helpful to detect instances where taxonomic conclusions would have been premature.

Whole genome-based methods such as GBDP were developed (Henz et al., 2005; Auch et al., 2006; Meier-Kolthoff et al., 2014a) for providing insights into the microbial tree of life (Patil and McHardy, 2013; Meier-Kolthoff et al., 2014a,b; Liu Y. et al., 2015; Garrido-Sanz et al., 2016; Lagkouvardos et al., 2016; Peeters et al., 2016; Montero-Calasanz et al., 2017; Mukherjee et al., 2017; Nouioui et al., 2018), for elucidating evolutionary relationships of viruses and eukaryotes (Riley et al., 2016; Meier-Kolthoff and Göker, 2017) and for yielding robust branch-support values (Hahnke R. L. et al., 2016; Nouioui et al., 2018). In general, distance methods for tree reconstruction still represent the most promising approach for accurately building phylogenies with a huge number of tips (Desper and Gascuel, 2004, 2006; Lefort et al., 2015). In the case of the GBDP/FastME approach the time-consuming step is the calculation of the intergenomic distances but this can be done incrementally since the pairwise distances are calculated independently of each other.

Encouragingly, the taxonomic conclusions drawn from the GBDP tree in the present study, too, were confirmed by corresponding supermatrix analyses in all of the cases investigated. The main approach of this study was to assess whether conflict was evident between trees inferred from distinct data and, if so, to conduct additional analyses. This approach appeared to be a robust procedure but the reliability of phylogenomic results might also depend on the kind of statistical resampling applied. Use of genome-scale data often yields more strongly resolved trees but this may also increase incongruities between distinct analyses (Jeffroy et al., 2006; Klenk and Göker, 2010). Topological incongruities between analyses of single genes attributed to horizontal gene transfer have even been used to argue against the concept of hierarchical classification itself (Bapteste and Boucher, 2009; Klenk and Göker, 2010). However, a strong hierarchical signal is indicated by the observation that the addition of more genes, up to virtually all available ones, increases support in phylogenomic trees (Breider et al., 2014). Studies that use only a limited number of genes rely on assumptions about the relative suitability of the selected genes compared to other genes (Lienau and DeSalle, 2009; Klenk and Göker, 2010) and can hardly be called genome-scale. Whole-genome methods, such as GBDP, yield truly genome-based phylogenies instead although overestimating phylogenetic confidence from genome-scale data must be avoided (Taylor and Piel, 2004). A reduction of the incongruities between trees and thereby more realistic phylogenomic support values can be obtained by bootstrapping entire genes instead of single alignment positions (Siddall, 2010; Hahnke R. L. et al., 2016; Simon et al., 2017; Nouioui et al., 2018), GBDP pseudo-bootstrapping in conjunction with the greedy-with-trimming algorithm (Meier-Kolthoff et al., 2014a) is akin to partition bootstrap.

Moreover, in this study partition bootstrapping was applied throughout in the supermatrix analyses. The effect of replacing ordinary bootstrapping by partition bootstrapping is illustrated by the subset of the data that was analyzed to determine *Lewinella* monophyly (Supplementary Data Sheet 1). Partition bootstrapping yielded only low support for the monophyly of the genus, 67% (47%) from the core-gene supermatrix and 23% (76%) from the full supermatrix under ML (MP). This is not in significant disagreement with the GBDP tree, which showed some support, but not particularly high support, for a paraphyletic *Lewinella*. Ordinary bootstrapping resulted in 100% (73%) support for *Lewinella* monophyly from the core-gene supermatrix and 100% (100%) from the full supermatrix under ML (MP). In contrast, there were twice as many single genes which displayed a support of ≥95% against *Lewinella* monophyly than single genes with a support ≥95% for *Lewinella* monophyly (Supplementary Data Sheet 1). While the controversial phylogenetic placement of the genome sequence of *L. nigricans* NBRC 102662^T^ may be due to contamination, its contigs did not display significant differences regarding the proportion of genes pointing to either monophyly or paraphyly of *Lewinella* (Supplementary Table 1).

As expected (Siddall, 2010; Simon et al., 2017), the ordinary bootstrap thus failed entirely to provide a realistic picture of the support and counter-support from all available genomic data for *Lewinella* monophyly. There are two main approaches to interpret such a result. An assumption that is implicitly made in all studies that only use a selection of the available genes (often presented as “conserved” or “housekeeping” genes) to form a supermatrix and analyse it using ordinary bootstrapping is that conflicting evidence from a minority of these genes and from the entirely neglected genes can be dismissed. We regard this assumption as careless, particularly when no attempt is made to actually prove that the conflicting evidence is caused by evolutionary phenomena such as horizontal gene transfer (Bapteste and Boucher, 2009) or artifacts of phylogenetic inference such as long-branch attraction (Bergsten, 2005). Alternatively, one can apply the partition bootstrap to a supermatrix (Siddall, 2010; Simon et al., 2017) or use equivalent methods such as GBDP pseudo-bootstrapping combined with appropriate distance formulas (Meier-Kolthoff et al., 2014a) as an attempt to obtain branch-support values that provide a better indication of whether or not the majority of the genes supports a certain clade.

In phylogenetic systematics, the purpose of taxonomic classification is to summarize the phylogeny of the organisms under consideration (Wiley and Lieberman, 2011). Formally, this is equivalent to selecting clades from a rooted phylogenetic tree, ideally well-supported clades (Vences et al., 2013), so as to assign taxa to these clades (in a way that avoids assigning more than a single taxon of the same rank to the same clade and avoids assigning a taxon of a rank higher than the one assigned to a certain clade to a subclade of this clade). While rampant horizontal gene transfer has been used to argue against a microbial tree of life and thus against hierarchical classification (Bapteste and Boucher, 2009), our earlier review had concluded that a strong phylogenetic signal is present despite horizontal gene transfer (Klenk and Göker, 2010), in line with an increase of branch support when more genes are added to a dataset (Breider et al., 2014). Our review failed to mention, however, that a situation as in *Lewinella*, where some branches are evidently supported by the majority of genes whereas others are not, may be unsatisfactory from a purely phylogenetic viewpoint but actually be advantageous for classification because it eases the selection of clades for assigning a taxon. In this respect, horizontal gene transfer does not only fail to impede hierarchical classification but may actually be beneficial for it. Similar observations were made regarding the classification of bacterial and archaeal viruses (Meier-Kolthoff and Göker, 2017).

### Genomic and Phenotypic Features as Taxonomic Markers

As for the analysis of individual characters, the use of “diagnostic” features in microbial taxonomy as support for taxa is a practice that clashes with the principles of phylogenetic systematics (Montero-Calasanz et al., 2017) which emphasize apomorphies as evidence for the monophyly of a group (Hennig, 1965; Wiley and Lieberman, 2011). The pitfalls of relying on “diagnostic” character states do not originate from homoplasy, which is a distinct problem, but from the fact that plesiomorphic (ancient) states may well be diagnostic but for a paraphyletic group (Nouioui et al., 2018). Reptiles may be the best known example of a paraphyletic group, as they are perfectly diagnosed by plesiomorphic features (Wiley and Lieberman, 2011). Among other consequences, a single feature with two character states cannot be used to justify the separation of two distinct taxa because only the apomorphic character state can argue for the monophyly of one of the two taxa (Hennig, 1965; Wiley and Lieberman, 2011; Montero-Calasanz et al., 2017). The use of “diagnostic” character states in microbial taxonomy without caring about whether or not these states are apomorphic may either be regarded as an oversimplification or as historically originating from the phenetic school of taxonomic classification rather than the phylogenetic one (Klenk and Göker, 2010). In fact, the term “polyphasic taxonomy,” which stands for the integration of genetic and phenotypic data (Vandamme et al., 1996; Gillis et al., 2005; Kämpfer and Glaeser, 2012), was originally introduced in conjunction with phenetic methodology (Colwell, 1970).

Current microbial taxonomy remains inconsistent regarding its principles as long as phylogenetic methods such as maximum likelihood, maximum parsimony, or neighbor joining, as opposed to clustering techniques (Felsenstein, 2004), are applied to sequence data whereas phenetic reasoning (Sneath and Sokal, 1963) is applied to phenotypic data. For these reasons, there are two distinct kinds of causes for the discrepancies between the phylogenomic trees and the traditional classification, which was at least partially based on phenotypic characters. The characters themselves may be in conflict with the phylogenomic trees, and the taxonomic interpretation of these characters may be insufficient (Montero-Calasanz et al., 2017; Nouioui et al., 2018). In order to distinguish between these two options, it is of course necessary to examine the characters as they were used in the taxonomic literature. For such a historical assessment it makes no sense to analyse new, modified characters derived from the earlier ones by, e.g., taking their genetic background into account, as this genetic background was not used for establishing the traditional classification in the first place.

In fact, we did not find any of the terms “apomorphic,” “apomorphy,” “plesiomorphic,” and “plesiomorphy” in any of the 1,744 screened literature references on *Bacteroidetes* taxonomy within the database of the DSMZ Prokaryotic Nomenclature Up-To-Date service. However, given a tree topology and the distribution of the states of a character that does not display too much homoplasy, it is not difficult to determine which states are apomorphic and which are plesiomorphic. For instance, it is encouraging that the number of isoprene residues of the major menaquinones showed such high agreement with the phylogenomic tree (Table 1). While the considerable uniformity of the classes of *Bacteroidetes* regarding menaquinones comes as no surprise and is partially already reflected in their protologs, in other cases it appears necessary to accordingly emend their descriptions as proposed below. The example also nicely illustrates that there is always at least one character state, even in a key character, that must not be used to justify a group: if possessing MK-7 as major menaquinone is plesiomorphic as indicated in Table 2 and the GBDP tree, the set of strains that can be diagnosed by MK-7 do not need to form a monophylum. Had phenetic principles be applied to interpret this key character, the MK-7 taxa may have been grouped into a single class.

Likewise, it is reasonable to postulate that the ancestor of *Bacteroidetes* was aerobic and that, accordingly, an anaerobic life style is an apomorphy of specific subgroups of the phylum. Indeed, the oxygen condition also displayed a strong fit to the phylogenomic tree. A previous study using a 16S rRNA gene tree but phenotypic features collected from recent taxonomic descriptions published in the *International Journal of Systematic and Evolutionary Microbiology* across phyla (Barberán et al., 2017) observed that cell shape had a stronger phylogenetic signal than physiological features such as temperature and pH optimum and even relationship to oxygen. In the present study, using a genome-scale tree of *Bacteroidetes*, relationship to oxygen showed a considerably stronger phylogenetic signal than cell width and cell length (Table 1). Within *Bacteroidetes*, as exemplified by the class *Bacteroidia* and by *Capnocytophaga*, the switch from strictly aerobic to strictly anaerobic was apparently mediated by facultatively aerobic or facultatively anaerobic life styles (Supplementary Data Sheet 1). It should be noted, however, that the full range of physiological capabilities regarding oxygen may not be obvious from the original description of a species. For instance, *Flavobacterium resistens* was originally described as aerobic but later recognized as facultatively anaerobic (Kim et al., 2012).

Incomplete information from the taxonomic literature may also affect menaquinones as usually only the major menaquinone is reported instead of the full spectrum, including percentage occurrences of individual compounds (The same restriction applies to fatty acids but the analysis of their phylogenetic conservation in *Bacteroidetes* is beyond the scope of the present study). Menaquinones were reported as absent in *Bacteroides luti* (Hatamoto et al., 2014). If this is not an artifact, it further illustrates the difficulty to define taxa in terms of even seemingly conserved characters.

The relatively low fit of the occurrence of gliding motility and flexirubins to the tree (Table 1) is more difficult to explain. Biosynthesis of flexirubin-type pigments (Reichenbach et al., 1974, 1980) is encoded in a single large gene cluster in *Flavobacterium johnsoniae* UW101 and two distinct gene clusters in *Chitinophaga pinensis* DSM 2588 (McBride et al., 2009; Schöner et al., 2014). Similarly, gliding motility is based on the interplay of a variety of largely known genes and their products (McBride and Zhu, 2013; Nan et al., 2014; Nan, 2017). Both characters can thus be assumed to be rather complex. According to Dollo's law, complex features arise only once in evolution but may be lost several times (Farris, 1977), hence a group of organisms displaying a complex feature should be monophyletic or paraphyletic in a tree, but not polyphyletic (Nouioui et al., 2018). Thus, Dollo's law alone could account for homoplasy in a complex character. However, ancestral character-state reconstructions under maximum parsimony with TNT and the same settings as used in Table 1 did not indicate a considerably higher number of losses than gains in the case of gliding and flexirubins unless gains are weighted at least eight times more heavily than losses (Supplementary Data Sheet 1). Similarly, a Dollo scenario had been postulated regarding anoxygenic photosynthesis in *Rhodobacteraceae*, which was presumed to be originally present and lost several times, but recent data indicate that entire photosynthesis operons had been horizontally transferred several times during the evolution of the family (Brinkmann et al., 2018). Such vertical transfer of complex characters may also be possible in *Bacteroidetes*.

While examining the genomic constitution regarding biosynthesis of flexirubin-like pigments or gliding motility is beyond the scope of the present study, a potential alternative explanation is that many strains do not glide under the usual cultivation conditions even though they have the genomic potential to do so. Moreover, reports on presence or absence of flexirubins could lack accuracy because the frequently performed KOH test is easy to apply but lacks specificity and sensitivity in some cases (Bernardet et al., 2002). A variety of effects may thus contribute to the relatively low phylogenetic conservation of the distribution of gliding motility and flexirubins, which in turn may help explaining the observed discrepancies between the current taxonomic classification and the phylogenomic results.

Bacterial G+C content, bacterial genome size and menaquinones appeared to be strongly phylogenetically conserved in *Bacteroidetes* (Supplementary Data Sheet 1, Tables 1, 2). It is for these reasons that the descriptions of the classes of *Bacteroidetes* are proposed to be emended regarding menaquinones and that genome sizes have been added to the description of the reclassified and emended species as shown below. The significant correlation between genome size and G+C content that was found in previous studies (Almpanis et al., 2018; Nouioui et al., 2018) is not unexpected because symbiotic bacteria tend to have smaller genomes and to be richer in A+T content (Rocha and Danchin, 2002; Mann and Chen, 2010), whereas positive selection (Hildebrand et al., 2010) and G+C-biased gene conversion (Lassalle et al., 2015) can increase the G+C content. Although exceptions from the rule that reduced genomes have a low G+C content are known (McCutcheon et al., 2009), genome size could thus be regarded as non-independent of G+C content, which would cast some doubt on it use as a taxonomic marker. Yet the overall correlation was considerably reduced in strength after accounting for the impact of the phylogeny (Nouioui et al., 2018) and may in fact be restricted to G+C reduction effects in symbiotic bacteria with dramatically reduced genome sizes. Such groups appear to be lacking in *Bacteroidetes* (Supplementary Data Sheet 1). As in *Actinobacteria*, both G+C content and genome size thus appear to be of use as taxonomic markers in *Bacteroidetes*.

As in the case of our earlier studies (Hahnke R. L. et al., 2016; Nouioui et al., 2018), many species descriptions were found to be inaccurate or too imprecise now that it has been shown that within-species deviation in G+C content is at most 1% (Meier-Kolthoff et al., 2014c). It is good practice to strengthen species descriptions in this way because such values not only assist in detecting strains that do not belong to the same species but also show significant correlation to phylogenetic trees. In contrast, in most cases it is premature to redefine genera and higher taxa of *Bacteroidetes* in this way since additional type-strain genome sequences would be needed before this issue could be addressed. However, some genera were comprehensively sampled genomically in this study, which allows for providing respective emendations below.

## Conclusions and Outlook

The results of this comparative phylogenomic study provide a further improved framework for the classification of the phylum *Bacteroidetes*. The improved taxonomic classification provides a sound basis for future studies on these bacteria, not least on those of clinical, piscicultural, and ecological interest. While it may be regarded as a truism that genome-based taxonomy must be based on genomic data, it should not be overlooked that this actually rules out the application of arbitrary and non-representative gene selections, and this study reiterates the pitfalls inherent in combining such gene selections with ordinary bootstrapping to obtain branch support. Similarly, discrepancies with aspects of the taxonomic classification based on the 16S rRNA gene were apparently mainly caused by overestimated or disregarded branch support, even though some exceptions appear to exist in which the 16S rRNA gene is in real conflict with genome-scale phylogenies. While not as tightly bound to the phylogeny as the G+C content, the relatively high correspondence between genome size and phylogenomic trees analyses yields “grist to the taxonomic mill.” Even more encouraging is the strong agreement between prominent phenotypic characters of *Bacteroidetes* and truly genome-scale phylogenies, provided these characters are interpreted in terms of the principles of phylogenetic systematics, which have so successfully been applied outside of microbial taxonomy. Future phylogenomic studies should try to link such key phenotypic features, their genomic basis and their evolutionary relationships, and make an attempt to clarify the evolutionary relationships between the richly sampled groups that could as yet not be resolved. Success in *Bacteroidetes* may help to revitalize prokaryotic systematics as a fundamental scientific discipline in other parts of the bacterial tree of life.

### Taxonomic Consequences: New Classes

**Description of** ***Terrimicrobia*, class. nov**.

Ter.ri.mi.cro'bi.a (N.L. neut. n. *Terrimicrobium*, type genus of the type order of the class; *-ia*, ending to denote a class; N.L. fem. pl. n. *Terrimicrobia*, the class of the order *Terrimicrobiales*).

The description is the same as for the order *Terrimicrobiales* ord. nov., the currently sole order in the class. The type order of the class is *Terrimicrobiales*. The order had not previously been assigned to a class; phylogenetic analyses of genome and 16S rRNA gene sequences indicate it is best placed in a class of its own.

### Taxonomic Consequences: New Orders

**Description of** ***Terrimicrobiales*, ord. nov**.

Ter.ri.mi.cro.bi.a'les (N.L. neut. n. *Terrimicrobium*, type genus of the order; -*ales*, ending to denote an order; N.L. fem. pl. n. *Terrimicrobiales*, the *Terrimicrobium* order).

The description is the same as for the family *Terrimicrobiaceae* fam. nov., the sole family in the order. The type genus of the order is *Terrimicrobium*. The family had not previously been assigned to an order; phylogenetic analyses of genome and 16S rRNA gene sequences indicate it is best placed in an order of its own.

**Description of** ***Trueperales*, ord. nov**.

True.pe.ra'les (N.L. fem. n. *Truepera*, type genus of the order; -*ales*, ending to denote an order; N.L. fem. pl. n. *Trueperales*, the *Truepera* order).

The description is the same as for the family *Trueperaceae* (Albuquerque et al., 2005), the sole family in the order. The type genus of the order is *Truepera*. The order has been separated from *Deinococcales* based on phylogenetic analyses of genome and 16S rRNA gene sequences.

### Taxonomic Consequences: New Families

**Description of** ***Barnesiellaceae*, fam. nov**.

Bar.ne.si.el.la'ce.ae (N.L. fem. dim. n. *Barnesiella*, type genus of the family; *-aceae*, ending to denote a family; N.L. fem. pl. n. *Barnesiellaceae*, the *Barnesiella* family*)*.

Cells are Gram-negative, non-spore-forming, rod-shaped and non-motile. Strictly anaerobic. Major fatty acids include iso-C_15:0_ and anteiso-C_15:0_. The G+C content as calculated from genome sequences is around 37.8–50.1% while the range provided in the literature is 38.5–52 mol%. The family contains the genera *Barnesiella* (the type genus), *Coprobacter*, and *Muribaculum*. The name *Barnesiellaceae* was suggested earlier on (Ormerod et al., 2016) but has never been validated.

**Description of** ***Cesiribacteraceae*, fam. nov**.

Ce.si.ri.bac.te.ra'ce.ae (N.L. masc. n. *Cesiribacter*, type genus of the family; -*aceae*, ending to denote a family; N.L. fem. pl. n. *Cesiribacteraceae*, the *Cesiribacter* family).

Cells are Gram-negative, rod-shaped, non-motile or motile by gliding. Strictly aerobic, oxidase, and catalase positive. The major menaquinone is MK-4 or MK-7. The major named polar lipids are phosphatidylethanolamine and diphosphatidylglycerol. The major fatty acids include iso-C_15:0_, anteiso-C_15:0_, iso-C_15:1_ G, iso-C_16:1_ G, C_13:1_ and/or iso-C_17:0_ 3-OH, and summed feature 3 (iso-C_15:0_ 2-OH and/or C_16:1_ ω7c). The G+C content calculated from genome sequences is around 45.7–54.6%; the range provided in the literature is 45.4–50.9 mol%. The family currently comprises the genera *Cesiribacter* (the type genus) and *Nafulsella*. It has been separated from other families based on phylogenetic analyses of genome and 16S rRNA gene sequences.

**Description of** ***Dysgonomonadaceae*, fam. nov**.

Dys.go.no.mo.na.da'ce.ae (N.L. fem. n. *Dysgonomonas*, type genus of the family; -*aceae*, ending to denote a family; N.L. fem. pl. n. *Dysgonomonadaceae*, the *Dysgonomonas* family).

Cells are Gram-negative, fermentative, strictly or facultatively anaerobic, non-spore-forming, coccobacilli to rod-shaped bacteria. Motility is variable. Catalase and oxidase are variable. The major menaquinone is MK-8. The major fatty acids include anteiso-C_15:0_ and either iso-C_15:0_, C_15:0_, iso-C_14:0_, iso-C_16:0_ 3-OH, iso-C_17:0_ 3-OH, or C_17:0_ 2-OH. The G+C content as calculated from genome sequences is around 36.7–39.6%; the range provided in the literature is 38.0–48.2 mol%. The family currently comprises the genera *Dysgonomonas* (the type genus), *Fermentimonas, Petrimonas*, and *Proteiniphilum*. The name *Dysgonomonadaceae* was suggested earlier on Ormerod et al. (2016) but has never been validated.

**Description of** ***Flexibacteraceae*, fam. nov**.

Fle.xi.bac.te.ra'ce.ae (N.L. masc. n. *Flexibacter*, type genus of the family; *-aceae*, ending to denote a family; N.L. fem. pl. n. *Flexibacteraceae*, the *Flexibacter* family).

The description is as for *Flexibacter* (Soriano, 1945; Hahnke R. L. et al., 2016) which is the type and currently sole genus of the family. It has been separated from other families based on phylogenetic analyses of genome and 16S rRNA gene sequences.

**Description of** ***Fulvivirgaceae*, fam. nov**.

Ful.vi.vir.ga'ce.ae (N.L. fem. n. *Fulvivirga*, type genus of the family; -*aceae*, ending to denote a family; N.L. fem. pl. n. *Fulvivirgaceae*, the *Fulvivirga* family).

Gram-negative, non-spore forming, non-flagellated or motile by gliding, rod shaped bacteria. Strictly aerobic and chemoorganotrophic metabolism. Catalase and oxidase positive. The major named polar lipid is phosphatidylethanolamine. The major menaquinone is MK-7. Major fatty acids are iso-C_15:0_ and either C_16:1_ ω5c, iso-C_17:0_ 3-OH or summed feature 3 (iso-C_15:0_ 2-OH and/or C_16:1_ ω7c). The G+C content calculated from genome sequences is around 42.4–51.2%; the range provided in the literature is 42.8–59.9 mol%. The family currently comprises the genera *Chryseolinea, Fulvivirga* (the type genus), and *Ohtaekwangia*. It has been separated from other families based on phylogenetic analyses of genome and 16S rRNA gene sequences.

**Description of** ***Marivirgaceae*, fam. nov**.

Ma.ri.vir.ga'ce.ae (N.L. fem. n. *Marivirga*, type genus of the family; -*aceae*, ending to denote a family; N.L. fem. pl. n. *Marivirgaceae*, the *Marivirga* family).

The description is as that for *Marivirga* (Nedashkovskaya et al., 2010c) which is the type and currently sole genus of the family. It has been separated from other families based on phylogenetic analyses of genome and 16S rRNA gene sequences.

**Description of** ***Reichenbachiellaceae*, fam. nov**.

Re.i.chen.ba.chi.el.la'ce.ae (N.L. fem. dim. n. *Reichenbachiella*, type genus of the family; *-aceae*, ending to denote a family; N.L. fem. pl. n. *Reichenbachiellaceae*, the *Reichenbachiella* family).

Gram-negative, non-spore-forming bacteria. Cells are flexible thread-like rods or rod-shaped. Motile by gliding. Aerobic and chemoorganotrophic. Oxidase and catalase positives. Synthesis of carotenoid and flexirubin-type pigments variable. The major menaquinone is MK-7. The major fatty acids include iso-C_15:0_ and/or summed feature 3 (iso-C_15:0_ 2-OH and/or C_16:1_ ω7c). The G+C content of genome sequences is around 39.8–45.4%; the range provided in the literature is 37.2–44.5 mol%. The family currently comprises the genera *Ekhidna, Marinoscillum, Reichenbachiella* (the type genus). It has been separated from other families based on phylogenetic analyses of genome and 16S rRNA gene sequences.

**Description of** ***Roseivirgaceae*, fam. nov**.

Ro.se.i.vir.ga'ce.ae (N.L. fem. n. *Roseivirg*a, type genus of the family; -*aceae*, ending to denote a family; N.L. fem. pl. n. *Roseivirgaceae*, the *Roseivirga* family).

The description is as for *Roseivirga* (Nedashkovskaya et al., 2005a,e, 2008a; Selvaratnam et al., 2016) which is the type and currently sole genus of the family. It has been separated from other families based on phylogenetic analyses of genome and 16S rRNA gene sequences.

**Description of** ***Terrimicrobiaceae*, fam. nov**.

Ter.ri.mi.cro.bi.a'ce.ae (N.L. neut. n. *Terrimicrobium*, type genus of the family; -*aceae*, ending to denote a family; N.L. fem. pl. n. *Terrimicrobiaceae*, the *Terrimicrobium* family*)*.

The description is as for *Terrimicrobium* (Qiu Y.-L. et al., 2014) which is the type and currently sole genus of the family. It has been separated from other families based on phylogenetic analyses of genome and 16S rRNA gene sequences.

**Description of** ***Thermoflexibacteraceae*, fam. nov**.

Ther.mo.fle.xi.bac.te.ra'ce.ae (N.L. masc. n. *Thermoflexibacter*, type genus of the family; -aceae, ending to denote a family; N.L. fem. pl. n. *Thermoflexibacteraceae*, the *Thermoflexibacter* family).

The description is as for *Thermoflexibacter* (Hahnke R. L. et al., 2016) which is the type and currently sole genus of the family. It has been separated from other families based on phylogenetic analyses of genome and 16S rRNA gene sequences.

**Description of** ***Weeksellaceae*, fam. nov**.

Week.sel.la'ce.ae (N.L. fem. dim. n. *Weeksella*, type genus of the family; -*aceae*, ending to denote a family; N.L. fem. pl. n. *Weeksellaceae*, the *Weeksella* family).

Gram-negative, non-spore-forming bacteria. Cells are coccoid, rod-shaped or pleomorphic. Aerobic, microaerobic or facultatively anaerobic chemoorganotrophs. Fermentative metabolism is also possible. Oxidase and catalase activities variable. Non-flagellated cells. Synthesis of carotenoid or flexirubin-type pigments variable. Mostly, the major named polar lipid is phosphatidylethanolamine. The major menaquinones are MK-6 and MK-7. The major fatty acids include iso-C_15:0_ and iso-C_17:0_ 3-OH. The G+C content as calculated from genome sequences is around 29.2–45.1%; 29.0–45.6 mol% is provided in the literature. The family currently comprises the genera *Algoriella, Apibacter, Bergeyella, Chishuiella, Chryseobacterium, Cloacibacterium, Cruoricaptor, Elizabethkingia, Empedobacter, Moheibacter, Ornithobacterium, Riemerella, Wautersiella*, and *Weeksella* (the type genus) after including *Soonwooa* in *Chryseobacterium*. The family has been separated from other families based on phylogenetic analyses of genome and 16S rRNA gene sequences. *Spongiimonas* is also tentatively assigned to this family.

### Taxonomic Consequences: New Genera

**Description of** ***Albibacterium*, gen. nov**.

Al.bi.bac.te'ri.um (L. adj. *albus*, white; L. neut. n. *bacterium*, rod; N.L. neut. n. *Albibacterium*, white rod).

The description is as that for *Albibacterium bauzanensis*, comb. nov., which is the type species. The genus has been separated from *Pedobacter* based on phylogenetic analyses of genome and 16S rRNA gene sequences.

**Description of** ***Algorimicrobium*, gen. nov**.

Al.go.ri.mi.cro'bi.um (L. masc. n. algor/-oris, cold; N.L. neut. n. *microbium*, a microbe; N.L. neut. n. *Algorimicrobium*, a cold microbe).

Gram-negative, rod-shaped cells, non-motile by gliding. Strictly aerobic and chemoorganotrophic metabolism. Oxidase and catalase variable. The major menaquinone is MK-6. The major fatty acids include iso-C_15:0_, and either anteiso-C_15:0_, iso-C_15:1_ G or iso-C_17:0_ 3-OH. The genomic G+C content provided in literature is around 33.4–45 mol%. The type species is *Algorimicrobium algoritergicola*, comb. nov.

**Description of** ***Breznakibacter*, gen. nov**.

Brez.na.ki.bac'ter (N.L. masc. n. *bacter*, a rod; N.L. masc. n. *Breznakibacter*, a rod named after John A. Breznak, an American microbiologist, professor at Michigan State University, East Lansing, Michigan, in recognition of his important contributions to insect gut microbiology).

The description is as for *Breznakibacter xylanolyticus*, comb. nov., which is the type species. The genus has been separated from *Cytophaga* based on phylogenetic analyses of genome and 16S rRNA gene sequences.

**Description of** ***Daejeonella*, gen. nov**.

Dae.je.o.nel'la (N.L. fem. dim. n. *Daejeonella*, derived from Daejeon, a city in South Korea where the type species was isolated).

Gram-negative, non-spore-forming bacteria. Cells are rod-shaped, non-motile or motile by gliding. Strictly aerobic. Oxidase and catalase positive. The major menaquinone is MK-7. The major fatty acids include iso-C_15:0_, anteiso-C_15:0_, iso-C_17:0_ 3-OH and summed feature 3 (iso-C_15:0_ 2-OH and/or C_16:1_ ω7c). The G+C content of genome sequences is around 37.8–50.0. The range in the literature is 37.7–48.5 mol%. The type species is *Daejeonella composti*, comb. nov.

**Description of** ***Flavilitoribacter*, gen. nov**.

Fla.vi.li.to.ri.bac'ter (L. adj. *flavus*, yellow; L. n. *litus*, -*oris*, sand beach; N.L. masc. n. *bacter*, a rod; N.L. masc. n. *Flavilitoribacter*, a yellow, rod-shaped bacterium from sand beach).

The description is as for *Flavilitoribacter nigricans*, comb. nov., which is the type species. The genus has been separated from *Lewinella* based on phylogenetic analyses of genome and 16S rRNA gene sequences.

**Description of** ***Aurantibacter*, gen. nov**.

Au.ran.ti.bac'ter (N.L. adj. *aurantus*, orange-colored; N.L. masc. n. *bacter*, rod; N.L. masc. n. *Aurantibacter*, orange rod).

The description is as for *Aurantibacter aestuarii* comb. nov., which is the type species. The genus has been separated from *Mesoflavibacter* based on phylogenetic analyses of genome and 16S rRNA gene sequences.

### Taxonomic Consequences: New Combinations for Species

**Description of** ***Aequorivita aquimaris*, comb. nov**.

A. a.qui.ma'ris (L. n. *aqua*, water; L. gen. n. *maris*, of the sea; N.L. gen. n. *aquimaris*, of the water of the sea).

Basonym: *Vitellibacter aquimaris* Thevarajoo et al. 2016.

The description is as for *Vitellibacter aquimaris* (Thevarajoo et al., 2016). The type strain is D-24 = DSM 101732 = KCTC 42708.

**Description of** ***Albibacterium*** *bauzanense*, comb. nov.

A. bau.za.nen'se (N.L. neut. adj. *bauzanense*, referring to Bauzanum, the medieval Latin name for Bozen/Bolzano, a city in South Tyrol, Italy, where the species was first found).

Basonym: *Pedobacter bauzanensis* Zhang et al. 2010

The description is as for *Pedobacter bauzanensis* (Zhang et al., 2010b). The type strain is BZ42 = CIP 110134 = DSM 22554.

**Description of** ***Algorimicrobium algoritergicola*, comb. nov**.

A. al.go.ri.ter.gi'co.la (L. n. *algor*, the cold; L. n. *tergum*, outer covering or surface; L. suff. -*cola*, the dweller, inhabitant; N.L. neut. n. *algoritergicola*, the inhabitant of a cold surface or covering).

Basonym: *Bizionia algoritergicola* Bowman and Nichols 2005

The description is as for *Bizionia algoritergicola* (Bowman and Nichols, 2005). The type strain is APA-1 = ACAM 1056 = CIP 108533.

**Description of** ***Algorimicrobium argentinense*, comb. nov**.

A. ar.gen.ti.nen'se (N.L. neut. adj. *argentinense*, pertaining to Argentina, the country associated with the scientific station in the vicinity of which the strain was isolated).

Basonym: *Bizionia argentinensis* Bercovich et al. 2008

The description is as for *Bizionia argentinensis* (Bercovich et al., 2008). The type strain is JUB59 = CCM-A-29 1259 = DSM 19628.

**Description of** ***Algorimicrobium echini*, comb. nov**.

A. e.chi'ni (L. gen. n. *echini*, of/from a sea urchin).

Basonym: *Bizionia echini* Nedashkovskaya et al. 2010

The description is as for *Bizionia echini* (Nedashkovskaya et al., 2010b). The type strain is KMM 6177 = DSM 23925 = KCTC 22015.

**Description of** ***Algorimicrobium hallyeonense*, comb. nov**.

A. hal.ly.e.o.nen'se (N.L. neut. adj. *hallyeonense*, pertaining to Hallyeo Marine National Park, the location of Tongyoung, where the type strain was isolated).

Basonym: *Bizionia hallyeonensis* Yoon et al. 2013

The description is as for *Bizionia hallyeonensis* (Yoon J.-H. et al., 2013). The type strain is T-y7 = CCUG 62110 = KCTC 23881.

**Description of** ***Algorimicrobium myxarmorum*, comb. nov**.

A. myx.ar.mo'rum (Gr. n. *myxa*, slime; L. gen. pl. n. *armorum*, defensive armor; N.L. gen. pl. n. *myxarmorum*, of armor slime, i.e., of the slime on the carapace of a crustacean host).

Basonym: *Bizionia myxarmorum* Bowman and Nichols 2005

The description is as for *Bizionia myxarmorum* (Bowman and Nichols, 2005). The type strain is ADA-4 = ACAM 1058 = CIP 108535.

**Description of** ***Algorimicrobium psychrotolerans*, comb. no*v*.**

A. psy.chro.to'le.rans (Gr. adj. *psychros*, cold; L. part. adj. *tolerans*, tolerating; N.L. part. adj. *psychrotolerans*, tolerating cold temperature).

Basonym: *Bizionia psychrotolerans* Song et al. 2015

The description is as for *Bizionia psychrotolerans* (Song et al., 2014). The type strain is PB-M7 = JCM 19924 = KCCM 43042.

**Description of** ***Algorimicrobium sediminis*, comb. nov**.

A. se.di'mi.nis (L. gen. n. *sediminis*, of sediment).

Basonym: *Bizionia sediminis* Zhang et al. 2017

The description is as for *Bizionia sediminis* (Zhang et al., 2017). The type strain is P131 = KCTC 42587 = MCCC 1H00124.

**Description of** ***Arcticibacter tournemirensis*, comb. nov**.

A. tour.ne.mi.ren'sis (N.L. masc. adj. *tournemirensis*, of or belonging to Tournemire, a village in Aveyron, France).

Basonym: *Pedobacter tournemirensis* Urios et al. 2013

The description is as for *Pedobacter tournemirensis* (Urios et al., 2013). The type strain is TF5-37.2-LB10 = CIP 110085 = DSM 23085.

**Description of** ***Breznakibacter xylanolyticus*, comb. nov**.

B. xy.la.no.ly'ti.cus (N.L. n. *xylanum*, xylan (a xylose-containing heteropolysaccharides in plant cell walls); N.L. masc. adj. *lyticus*, (from Gr. fem. adj. *lutikê*) able to loosen, able to dissolve; N.L. masc. adj. *xylanolyticus*, xylan-dissolving).

Basonym: *Cytophaga xylanolytica* Haack and Breznak 1993

The description is as for *Cytophaga xylanolytica* (Haack and Breznak, 1993) with the following modification. The G+C content of the type-strain genome is 46.6%, its approximate size 4.37 Mbp, its GenBank deposit SAMN05660438. The type strain is XM3 = ATCC 51429 = DSM 6779.

**Description of** ***Chryseobacterium buanense*, comb. nov**.

C. bu.a.nen'se (N.L. neut. adj. *buanense*, pertaining to Buan beach, where the type strain was isolated).

Basonym: *Soonwooa buanensis* Joung et al. 2010

The description is as for *Soonwooa buanensis* (Joung et al., 2010) with the following modification. The G+C content of the type-strain genome is 34.8%, its approximate size 3.33 Mbp, its GenBank deposit SAMN05660477. The type strain is HM0024 = DSM 26167 = CECT 7503.

**Description of** ***Daejeonella composti*, comb. nov**.

D. com.pos'ti (N.L. gen. n. *composti*, of compost).

Basonym: *Pedobacter composti* Kim et al. 2010

The description is as for *Pedobacter composti* (Lee H.-G. et al., 2009). The type strain is TR6-06 = KCTC 12638 = LMG 23490.

**Description of** ***Daejeonella huanghensis*, comb. nov**.

D. huang.hen'sis (N.L. fem. adj. *huanghensis*, named after the Chinese Arctic Huanghe Station, the site where the type strain was isolated).

Basonym: *Pedobacter huanghensis* Qiu et al. 2014

The description is as for *Pedobacter huanghensis* (Qiu X. et al., 2014). The type strain is M1-27 = CCTCC AB 2012936 = LMG 28205.

**Description of** ***Daejeonella lutea*, comb. nov**.

D. lu'te.a (L. fem. adj. *lutea*, orange-yellow, referring to the colony color on R2A agar).

Basonym: *Pedobacter luteus* Oh et al. 2013

The description is as for *Pedobacter luteus* (Oh et al., 2013) with the following modification. The G+C content of the type-strain genome is 42.3%, its approximate size 4.03 Mbp, its GenBank deposit SAMN05661099. The type strain is N7d-4 = DSM 22385 = KCTC 22699.

**Description of** ***Daejeonella oryzae*, comb. nov**.

D. o.ry'zae (L. gen. n. *oryzae*, of rice, referring to the rice paddy fields where the type strain was isolated).

Basonym: *Pedobacter oryzae* Jeon et al. 2009

The description is as for *Pedobacter oryzae* (Jeon et al., 2009). The type strain is N7 = DSM 19973 = KACC 12821.

**Description of** ***Daejeonella rubra*, comb. nov**.

D. rub'ra (L. fem. adj. rubra, red colored, referring to the pigmentation of the bacterium).

Basonym: *Pedobacter ruber* Margesin and Zhang 2013

The description is as for *Pedobacter ruber* (Margesin and Zhang, 2013). The type strain is W1 = DSM 24536 = LMG 26240.

**Description of** ***Ezakiella coagulans*, comb. nov**.

E. co.a'gu.lans (N.L. v. *coagulo*, to curdle or coagulate; N.L. part. adj. *coagulans*, curdling, coagulating).

Basonym: *Bacteroides coagulans* Eggerth and Gagnon 1933 (Approved Lists 1980)

The description is as for *Bacteroides coagulans* (Eggerth and Gagnon, 1932) with the following addition. The G+C content of the type-strain genome is 35.6%, its approximate size 1.81 Mbp, its IMG deposit 2756170280. The type strain is ATCC 29798 = DSM 20705 = JCM 12528.

**Description of** ***Flavilitoribacter nigricans*, comb. nov**.

F. ni'gri.cans (L. part. adj. *nigricans*, blackening).

Basonym: *Lewinella nigricans* Khan et al. 2007

The description is as for *Lewinella nigricans* (Khan et al., 2007a) with the following modification. The G+C content of the type-strain genome is 51.0%, its approximate size 11.33 Mbp, its GenBank deposit SAMN07775960. The type strain is SS-2 = ATCC 23147 = DSM 23189.

**Description of** ***Hungatella xylanolytica*, comb. nov**.

H. xy.la.no.ly'ti.ca (N.L. neut. n. *xylanum*, xylan; N.L. adj. *lyticus*/-*a*, (from Gr. adj. *lutikos/*-ê*/*-*on*), able to loosen, able to dissolve; N.L. fem. adj. *xylanolytica*, xylan-dissolving).

Basonym: *Bacteroides xylanolyticus* Scholten-Koerselman et al. 1988

The description is as for *Bacteroides xylanolyticus* (Scholten-Koerselman et al., 1986) with the following restriction. The G+C content of the type-strain genome is 41.8%, its approximate size 5.63 Mbp, its IMG deposit 2739367553. The type strain is X5-1 = CCUG 48289 = DSM 3808.

**Description of** ***Lachnospira eligens*, comb. nov**.

L. e'li.gens (L. part. adj. *eligens*, making a proper selection, selecting, referring to its generally poor growth without fermentable carbohydrate).

Basonym: *Eubacterium eligens* Holdeman and Moore 1974 (Approved Lists 1980)

The description is as for *Eubacterium eligens* (Holdeman and Moore, 1974) with the following modification. The G+C content of the type-strain genome is 37.6%, its approximate size 2.83 Mbp, its GenBank deposit SAMN02603083. The type strain is VPI C15-48 = ATCC 27750 = DSM 3376.

**Description of** ***Aurantibacter aestuarii*, comb. nov**.

M. aes.tu.a'ri.i (L. gen. n. *aestuarii*, of the tidal flat, from where the organism was isolated).

Basonym: *Mesoflavibacter aestuarii* Lee et al. 2014

The description is as for *Mesoflavibacter aestuarii* (Lee J. H. et al., 2014). The type strain is KYW614 = JCM 19524 = KCTC 32269.

**Description of** ***Muricauda koreensis*, nom. nov**.

M. ko.re.en'sis (L. fem. adj. *koreensis*, pertaining to Korea, the country in which the type strain was isolated).

Basonym: *Flagellimonas aquimarina* Choi et al. 2018 (The name *Muricauda aquimarina* has already been validly published, hence a new epithet must be chosen to avoid homonyms).

The description is as for *Flagellimonas aquimarina* (Choi et al., 2018). The type strain is ECD12 = KCTC 52351 = JCM 32292.

**Description of** ***Muricauda eckloniae*, comb. nov**.

M. eck.lo'ni.ae (N.L. fem. n. *Ecklonia*, scientific genus name of the marine alga from which the bacterium was isolated; N.L. gen. n. *eckloniae*, of *Ecklonia*).

Basonym: *Flagellimonas eckloniae* Bae et al. 2007

The description is as for *Flagellimonas eckloniae* (Bae et al., 2007) with the following addition. The G+C content of the type-strain genome is 37.8%, its approximate size 4.13 Mbp, its GenBank deposit SAMN03571530. The type strain is DOKDO 007 = JCM 13831 = KCCM 42307.

**Description of** ***Muricauda flava*, comb. nov**.

M. fla'va (L. fem. adj. *flava*, yellow, the color of colonies or pigment).

Basonym: *Spongiibacterium flavum* Yoon and Oh 2012

The description is as for *Spongiibacterium flavum* (Yoon and Oh, 2012). The type strain is A11 = DSM 22638 = KCTC 22665.

**Description of** ***Muricauda pacifica*, comb. nov**.

M. pa.ci'fi.ca (L. fem. adj. *pacifica*, peaceful, pertaining to the Pacific Ocean).

Basonym: *Spongiibacterium pacificum* Gao et al. 2015

The description is as for *Spongiibacterium pacificum* (Gao X. et al., 2015) with the following modification. The G+C content of the type-strain genome is 38.4%, its approximate size 4.44 Mbp, its IMG deposit 2728369489. The type strain is SW169 = DSM 25885 = JCM 18379.

**Description of** ***Myroides anatoliensis*, comb. nov**.

M. a.na.to.li.en'sis (N.L. masc. adj. *anatoliensis*, of or belonging to Anatolia).

Basonym: *Flavobacterium anatoliense* Kacagan et al. 2013

The description is as for *Flavobacterium anatoliense* (Kacagan et al., 2013). The type strain is MK3 = LMG 26441 = NCCB 100384.

**Description of** ***Myroides aquimaris*, nom. nov**.

M. a.qui.ma'ris (L. n. *aqua*, water; L. gen. n. *maris*, of the sea; N.L. gen. n. *aquimaris*, of the water of the sea).

Basonym: *Flavobacterium marinum* Song et al. 2013 (The name *Myroides marinus* has already been validly published (Cho S.-H. et al., 2011), hence a new epithet must be chosen to avoid homonyms).

The description is as for *Flavobacterium marinum* (Song et al., 2013). The type strain is SW105 = CGMCC 1.10825 = JCM 18132.

**Description of** ***Myroides ceti*, comb. nov**.

M. ce'ti (L. n. *cetus*, whale; L. gen. n. *ceti*, of a whale).

Basonym: *Flavobacterium ceti* Kacagan et al. 2013

The description is as for *Flavobacterium ceti* (Kacagan et al., 2013). The type strain is 454-2 = CCUG 52969 = CECT 7184.

**Description of** ***Myroides cloacae*, comb. nov**.

M. clo.a'cae (L. gen. n. *cloacae*, of a sewer).

Basonym: *Flavobacterium cloacae* Liu et al. 2017

The description is as for *Flavobacterium cloacae* (Liu et al., 2017). The type strain is wh15 = CCTCC AB 2015420 = JCM 31173.

**Description of** ***Myroides ummariensis*, comb. nov**.

M. um.ma.ri.en'sis (N.L. masc. adj. *ummariensis*, pertaining to Ummari, the hexachlorocyclohexane-contaminated site from where the type strain was isolated).

Basonym: *Flavobacterium ummariense* Lata et al. 2012

The description is as for *Flavobacterium ummariense* (Lata et al., 2012) with the following modification. The G+C content of the type-strain genome is 34.7%, its approximate size 3.49 Mbp, its GenBank deposit SAMN05421741. The type strain is DS-12 = CCM 7847 = MTCC 10766.

**Description of** ***Phocaeicola barnesiae*, comb. nov**.

P. bar.ne'si.ae (N.L. gen. n. *barnesiae*, of Barnes, named after Ella M. Barnes, a British microbiologist, who has contributed much to our knowledge of intestinal bacteriology and anaerobic bacteriology in general).

Basonym: *Bacteroides barnesiae* Lan et al. 2006

The description is as for *Bacteroides barnesiae* (Lan et al., 2006). The type strain is BL2 = CCUG 54636 = DSM 18169.

**Description of** ***Phocaeicola caecicola*, comb. nov**.

P. cae.ci'co.la (N.L. n. *caecum*, (from L. *caecum* intestinum caecum) caecum; L. suff. *-cola*, (from L. n. *incola*) dweller, inhabitant; N.L. masc. n. *caecicola*, caecum-dweller).

Basonym: *Bacteroides caecicola* Irisawa et al. 2016

The description is as for *Bacteroides caecicola* (Irisawa et al., 2016). The type strain is C13EG70 = InaCC B449 = NBRC 110958.

**Description of** ***Phocaeicola caecigallinarum*, comb. nov**.

P. cae.ci.gal.li.na'rum (N.L. n. *caecum*, caecum; L. gen. pl. n. *gallinarum*, of hens; N.L. gen. pl. n. *caecigallinarum*, of/from caecum of hens).

Basonym: *Bacteroides caecigallinarum* Saputra et al. 2015

The description is as for *Bacteroides caecigallinarum* (Saputra et al., 2015). The type strain is C13EG111 = InaCC B455 = NBRC 110959.

**Description of** ***Phocaeicola chinchillae*, comb. nov**.

P. chin.chil'lae (N.L. n. C*hinchilla*, the zoological genus name of the chinchilla; N.L. gen. n. *chinchillae*, of a chinchilla).

Basonym: *Bacteroides chinchillae* Kitahara et al. 2011

The description is as for *Bacteroides chinchillae* (Kitahara et al., 2011). The type strain is ST170 = CCUG 59335 = JCM 16497.

**Description of** ***Phocaeicola coprocola***, **comb. nov**.

P. co.pro'co.la (Gr. n. *kopros*, feces; L. suff. *-cola*, inhabitant of; N.L. masc. n. *coprocola*, inhabitant of feces).

Basonym: *Bacteroides coprocola* Kitahara et al. 2005

The description is as for *Bacteroides coprocola* (Kitahara et al., 2005). The type strain is M16 = DSM 17136 = JCM 12979.

**Description of** ***Phocaeicola coprophilus*, comb. nov**.

P. co.pro'phi.lus (Gr. n. *copros*, dung, feces; Gr. masc. adj. *philos*, friendly to; N.L. masc. adj. *coprophilus*, loving feces).

Basonym: *Bacteroides coprophilus* Hahnke et al. 2016

The description is as for *Bacteroides coprophilus* (Hahnke R. L. et al., 2016). The type strain is CB42 = DSM 18228 = JCM 13818.

**Description of** ***Phocaeicola dorei*, comb. nov**.

P. do.re'i (N.L. gen. masc. n. *dorei*, of Doré, in honor of the French microbiologist Joel Doré, in recognition of his many contributions to intestinal (gut) microbiology).

Basonym: *Bacteroides dorei* Hahnke et al. 2016

The description is as for *Bacteroides dorei* (Hahnke R. L. et al., 2016). The type strain is CCUG 53892 = DSM 17855 = JCM 13471.

**Description of** ***Phocaeicola gallinaceus*, comb. nov**.

P. gal.li.na'ce.us (L. masc. adj. *gallinaceus*, of or pertaining to a domestic fowl or poultry).

Basonym: *Bacteroides gallinaceum* Irisawa et al. 2016

The description is as for *Bacteroides gallinaceum* (Irisawa et al., 2016). The type strain is C13EG186 = InaCC B455 = NBRC 110963.

**Description of** ***Phocaeicola massiliensis*, comb. nov**.

P. mas.si.li.en'sis (L. masc. adj. *massiliensis*, of Massilia, the ancient Greek and Roman name for Marseille, France, where the type strain was isolated).

Basonym: *Bacteroides massiliensis* Hahnke et al. 2016

The description is as for *Bacteroides massiliensis* (Hahnke R. L. et al., 2016). The type strain is B84634 = CIP 107942 = DSM 17679.

**Description of** ***Phocaeicola paurosaccharolyticus*, comb. nov**.

P. pau.ro.sac.cha.ro.ly'ti.cus (Gr. adj. *pauros*, little or a few; Gr. n. *sakchâr*, sugar; Gr. adj. *lutikos*, dissolving; N.L. masc. adj. *paurosaccharolyticus*, dissolving a few kinds of sugars).

Basonym: *Bacteroides paurosaccharolyticus* Ueki et al. 2011

The description is as for *Bacteroides paurosaccharolyticus* (Ueki et al., 2011) with the following modification. The G+C content of the type-strain genome is 37.1%, its approximate size 3.43 Mbp, its GenBank deposit SAMD00009976. The type strain is WK042 = DSM 21004 = JCM 15092.

**Description of** ***Phocaeicola plebeius*, comb. nov**.

P. ple.bei'us (L. masc. adj. *plebeius*, common, of low class).

Basonym: *Bacteroides plebeius* Kitahara et al. 2005

The description is as for *Bacteroides plebeius* (Kitahara et al., 2005). The type strain is M12 = DSM 17135 = JCM 12973.

**Description of** ***Phocaeicola salanitronis*, comb. nov**. P. sa.la.ni.tro'nis (N.L. gen. masc. n. *salanitronis*, of Salanitro, named after Joseph P. Salanitro, an American microbiologist, who has contributed much to our knowledge of intestinal bacteriology in chicken and anaerobic bacteriology in general).

Basonym: *Bacteroides salanitronis* Lan et al. 2006

The description is as for *Bacteroides salanitronis* (Lan et al., 2006). The type strain is BL78 = DSM 18170 = JCM 13657.

**Description of** ***Phocaeicola sartorii*, comb. nov**.

P. sar.to'ri.i (N.L. gen. n. *sartorii*, in honor of Balfour Sartor, Professor of Medicine, Microbiology and Immunology at the University of North Carolina in Chapel Hill, for his outstanding contribution to the understanding of microbial ecology in inflammatory bowel diseases).

Basonym: *Bacteroides sartorii* Sakamoto and Ohkuma 2012

The description is as for *Bacteroides sartorii* (Sakamoto and Ohkuma, 2012a) with the following restriction. The G+C content of the type-strain genome is 43.8%, its approximate size 5.29 Mbp, its GenBank deposit SAMD00010076. The type strain is A-C2-0 = CCUG 57211 = DSM 21941.

**Description of** ***Phocaeicola vulgatus*, comb. nov**.

P. vul.ga'tus (L. masc. adj. *vulgatus*, (from L. v. *vulgo*) common (referring to the frequent occurrence of the species in fecal flora).

Basonym: *Bacteroides vulgatus* Hahnke et al. 2016

The description is as for *Bacteroides vulgatus* (Hahnke R. L. et al., 2016). The type strain is ATCC 8482 = DSM 1447 = JCM 5826.

**Description of** ***Roseivirga halotolerans*, comb. nov**.

R. ha.lo.to'le.rans (Gr. masc. n. *hals*, salt; L. part. adj. *toleran*s, tolerating; N.L. part. adj. *halotolerans*, salt-tolerating).

Basonym: *Fabibacter halotolerans* Lau et al. 2006

The description is as for *Fabibacter halotolerans* (Lau et al., 2006a). The type strain is UST030701-097 = CIP 109252 = JCM 13334.

**Description of** ***Roseivirga misakiensis*, comb. nov**.

R. mi.sa.ki.en'sis (N.L. fem. adj. *misakiensis*, pertaining to Misaki, the city of which the type strain was isolated).

Basonym: *Fabibacter misakiensis* Wong et al. 2015

The description is as for *Fabibacter misakiensis* (Wong et al., 2015). The type strain is SK-8 = DSM 102187 = NBRC 110216.

**Description of** ***Roseivirga pacifica*, comb. nov**.

R. pa.ci'fi.ca (L. fem. adj. *pacifica*, pacific referring to the Pacific Ocean, from which the type strain was isolated).

Basonym: *Fabibacter pacificus* Huo et al. 2013

The description is as for *Fabibacter pacificus* (Huo et al., 2013) with the following modification. The G+C content of the type-strain genome is 42.0%, its approximate size 4.66 Mbp, its GenBank deposit SAMN05216290. The type strain is DY53 = DSM 100771 = JCM 18885.

**Description of** ***Roseivirga thermotolerans*, comb. nov**.

R. ther.mo.to'le.rans (Gr. adj. *thermos*, hot; L. part. adj. *tolerans*, tolerating; N.L. part. adj. *thermotolerans*, tolerating heat).

Basonym: *Fabivirga thermotolerans* Tang et al. 2016

The description is as for *Fabivirga thermotolerans* (Tang M. et al., 2016) with the following modification. The type strain is A-4 = CGMCC 1.15111 = KCTC 42507.

**Description of** ***Saccharicrinis aurantiacus*, comb. nov**.

S. au.ran.ti'a.cus (N.L. masc. adj. *aurantiacus*, orange-colored).

Basonym: *Labilibacter aurantiacus* Lu et al. 2017

The description is as for *Labilibacter aurantiacus* (Lu et al., 2017). The type strain is HQYD1 = KCTC 42583 = MCCC 1K02304.

**Description of** ***Sediminibacterium lacti*s, comb. nov**.

S. lac'tis (L. gen. n. *lactis*, of milk, isolated from milk of a donkey).

Basonym: *Asinibacterium lactis* Lee et al. 2013

The description is as for *Asinibacterium lactis* (Lee D.-G. et al., 2013). The type strain is LCJ02 = JCM 18484 = KCCM 90108.

**Description of** ***Sediminibacterium magnilacihabitans*, comb. nov**.

S. ma.gni.la.ci.ha'bi.tans (L. adj. *magnus*, great; L. n. *lacus*, lake; L. n. *habitans*, a dweller, inhabitant; N.L. masc. n. *magnilacihabitans*, great lake dweller, referring to the lake, Lake Michigan, one of the Great Lakes, from which the type species was first isolated).

Basonym: *Vibrionimonas magnilacihabitans* Albert et al. 2014

The description is as for *Vibrionimonas magnilacihabitans* (Albert et al., 2014). The type strain is MU-2 = DSM 22423 = NRRL B-59231.

**Description of** ***Thermoflavifilum thermophilum*, comb. nov**.

T. ther.mo'phi.lum (Gr. adj. *thermos*, hot; N.L. adj. *philus*/*-a*/*-um*, (from Gr. adj. *philos/-ê/-on*) friend, loving; N.L. neut. adj. *thermophilum*, heat-loving).

Basonym: *Crenotalea thermophila* Hanada et al. 2014

The description is as for *Crenotalea thermophila* (Hanada et al., 2014). The type strain is STH-1-Y1 = DSM 14807 = JCM 11541.

**Description of** ***Xanthomarina spongicola*, comb. nov**.

X. spon.gi'co.la (L. fem. n. *spongia*, sponge; L. suff. -*cola*, (from L. n. *incola*) inhabitant; N.L. fem. n. *spongicola*, inhabitant of sponges).

Basonym: *Formosa spongicola* Yoon and Oh 2011

The description is as for *Formosa spongicola* (Yoon and Oh, 2011) with the following modification. The G+C content of the type-strain genome is 31.2%, its approximate size 3.16 Mbp, its GenBank deposit SAMN05660201. The type strain is A2 = DSM 22637 = KCTC 22662.

### Taxonomic Consequences: New Combinations for Subspecies

**Description of** ***Chryseobacterium aquaticum* subsp**. ***greenlandense*, comb. nov., change in rank**

C. a.qua'ti.cum subsp. green.lan.den'se (N.L. neut. adj. *greenlandense*, of Greenland, where the novel species was isolated from).

Basonym: *Chryseobacterium greenlandense* Loveland-Curtze et al. 2010 emend. Hahnke et al. 2016

The description is as for *Chryseobacterium greenlandense* (Hahnke R. L. et al., 2016). The type strain is UMB34 = CIP 110007 = NRRL B-59357.

**Description of** ***Elizabethkingia anophelis* subsp**. ***endophytica*, comb. nov., change in rank**

E. a.no.phe'lis subsp. en.do.phy'ti.ca (Gr. pref. e*ndo*, within; Gr. n. *phyton*, plant; L. fem. suff. *-ica*, adjectival suffix used with the sense of belonging to; N.L. fem. adj. *endophytica*, within plant, endophytic, pertaining to the original isolation from plant tissue).

Basonym: *Elizabethkingia endophytica* Kämpfer et al. 2015

The description is as for *Elizabethkingia endophytica* (Kämpfer et al., 2015b) with the following addition. The G+C content of the type-strain genome is 35.5%, its approximate size 4.18 Mbp, its GenBank deposit SAMN05255122. The type strain is JM-87 = CIP 110885 = DSM 29660.

**Description of** ***Flavobacterium johnsoniae* subsp**. ***aurantiacum*, comb. nov., change in rank**

F. john.so'ni.ae subsp. au.ran.ti.a'cum (N.L. neut. adj. *aurantiacum*, orange-colored).

Basonym: *Flexibacter aurantiacus* Lewin 1969

The description is as for *Flexibacter aurantiacus* (Lewin, 1969) with the following modification. The G+C content of the type-strain genome is 33.9%, its approximate size 5.46 Mbp, its GenBank deposit SAMN05444388. The type strain is ATCC 23107 = DSM 6792 = LMG 3987 = NBRC 15970.

**Description of** ***Mesoflavibacter zeaxanthinifaciens* subsp**. ***sabulilitoris*, comb. nov., change in rank**

M. ze.a.xan.thi.ni.fa'ci.ens subsp. sa.bu.li.li'to.ris (L. n. *sabulum*, sand; L. n. *litus, -oris* the seashore, coast; N.L. gen. n. *sabulilitoris*, of sand of seashore, from which the type strain was isolated).

Basonym: *Mesoflavibacter sabulilitoris* Park et al. 2014

The description is as for *Mesoflavibacter sabulilitoris* (Park et al., 2014). The type strain is GJMS-9 = CECT 8597 = KCTC 42117.

**Description of** ***Myroides odoratimimus* subsp. x*uanwuensis*, comb. nov., change in rank**

M. o.do.ra.ti'mi.mus subsp. xu.an.wu.en'sis (N.L. masc. adj. *xuanwuensis*, referring to Xuanwu district, Nanjing, Jiangsu Province, PR China, where the organism was first isolated).

Basonym: *Myroides xuanwuensis* Zhang et al. 2014

The description is as for *Myroides xuanwuensis* (Zhang Z.-D. et al., 2014) with the following modification. The G+C content of the type-strain genome is 34.0%, its approximate size 4.02 Mbp, its GenBank deposit SAMN05444275. The type strain is TH-19 = DSM 27251 = JCM 19200.

### Taxonomic Consequences: Emendations of Classes

**Emended description of** ***Bacteroidia* Krieg 2012**

The description is as before (Krieg, 2011a) with the following addition. Depending on the subgroup, the major menaquinone is either mostly MK-7 or mostly MK-10.

**Emended description of** ***Chitinophagia* Munoz et al. 2017**

The description is as before (Munoz et al., 2016) with the following addition. The major menaquinone is usually MK-7.

**Emended description of** ***Cytophagia* Nakagawa 2012**

The description is as before (Nakagawa, 2011a) with the following addition. The major menaquinone is usually MK-7.

**Emended description of** ***Sphingobacteriia* Kämpfer 2012**

The description is as before (Kämpfer, 2011) with the following addition. The major menaquinone is usually MK-7.

**Emended description of** ***Verrucomicrobia* Hedlund 2012**

The description is as before (Hedlund, 2011) with the following modification. The phylum contains the classes *Opitutae, Terrimicrobia* class. nov. and *Verrucomicrobiae*.

### Taxonomic Consequences: Emendations of Orders

**Emended description of** ***Bacteroidales* Krieg 2012 emend. Pikuta et al. 2017**

The description is as before (Pikuta et al., 2017) with the following modification. The order contains the families *Bacteroidaceae, Balneicellaceae, Dysgonomonadaceae, Lentimicrobiaceae, Marinifilaceae, Marinilabiliaceae, Paludibacteraceae, Porphyromonadaceae, Prevotellaceae, Prolixibacteraceae, Rikenellaceae, Salinivirgaceae, Tannerellaceae*, and *Williamwhitmaniaceae*. The type genus is *Bacteroides*.

**Emended description of** ***Cytophagales* Leadbetter 1974**

The description is as before (Leadbetter, 1974) with the following modification. The order consists of the families *Bernardetiaceae, Catalimonadaceae, Cyclobacteriaceae, Cytophagaceae, Flammeovirgaceae, Hymenobacteraceae, Microscillaceae, Mooreiaceae, Persicobacteraceae, Spirosomaceae*, and *Thermonemataceae* as well as the newly assigned families *Cesiribacteraceae* fam. nov., *Flexibacteraceae* fam. nov., *Fulvivirgaceae* fam. nov., *Marivirgaceae* fam. nov., *Reichenbachiellaceae* fam. nov., *Roseivirgaceae* fam. nov., and *Thermoflexibacteraceae* fam. nov. The type genus is *Cytophaga*.

**Emended description of** ***Flavobacteriales* Bernardet 2012**

The description is as before (Bernardet, 2011b) with the following modification. The order contains the families *Crocinitomicaceae, Cryomorphaceae, Flavobacteriaceae, Ichthyobacteriaceae, Schleiferiaceae*, and *Weeksellaceae* fam. nov. *Blattabacteriaceae*, from which no culture has been isolated, is tentatively also assigned to this order.

**Emended description of** ***Saprospirales* Hahnke et al. 2017**

The description is as before (Hahnke R. L. et al., 2016) with the following modification. The order contains the families *Lewinellaceae* and *Saprospiraceae* after including *Haliscomenobacteraceae* in *Lewinellaceae*. The type genus is *Saprospira*.

### Taxonomic Consequences: Emendations of Families

**Emended description of** ***Bacteroidaceae* Pribram 1933**

The description is as before (Pribram, 1933) with the following modification after inclusion of *Phocaeicola*. Cells are coccobacilli or rod-shaped and motility is variable.

**Emended description of** ***Cytophagaceae* Stanier 1940**

The description is as before (Stanier, 1940) with the following modification. The family contains the genera *Cytophaga* (the type genus), *Rhodocytophaga* and *Sporocytophaga*.

**Emended description of** ***Flavobacteriaceae* Reichenbach et al. 1992 emend. Bernardet et al. 2002**

The description is as before (Bernardet et al., 2002) with the following modification. The family contains the genera *Algorimicrobium* gen. nov. and *Aurantibacter* gen. nov., in addition to the previously included genera with the exception of *Algoriella, Apibacter, Bergeyella, Chishuiella, Chryseobacterium, Cloacibacterium, Cruoricaptor, Elizabethkingia, Empedobacter, Moheibacter, Ornithobacterium, Riemerella, Wautersiella*, and *Weeksella* which were assigned to *Weeksellaceae*.

**Emended description of** ***Lewinellaceae* Hahnke et al. 2016**

The description is as before (Hahnke R. L. et al., 2016) with the following modification that reflect the inclusion of additional genera. Cells are non-motile or motile by gliding. The family contains the genera *Lewinella* (the type genus), *Flavilitoribacter* gen. nov., *Haliscomenobacter, Phaeodactylibacter* and *Portibacter*.

**Emended description of** ***Marinilabiliaceae* Ludwig et al. 2012**

The description is as before (Ludwig et al., 2011b) with the following modification. This family houses *Breznakibacter*, gen. nov., in addition to the previously included genera *Alkaliflexus, Alkalitalea, Anaerophaga, Carboxylicivirga, Geofilum, Mangroviflexus, Marinilabilia, Natronoflexus, Saccharicrinis*, and *Thermophagus*.

**Emended description of** ***Odoribacteraceae* Munoz et al. 2016 emend. Hahnke et al. 2016**

The description is as before (Hahnke R. L. et al., 2016) with the following modification after inclusion of the genus *Gabonibacter*. Coccobacilli or fusiform cells variable in motility.

**Emended description of** ***Rhodothermaceae* Ludwig et al. 2012 emend. Hahnke et al. 2016**

The description is as before (Hahnke R. L. et al., 2016) with the following modification. The genera *Longibacter* and *Longimonas* have been removed based on the phylogenetic analysis of 16S rRNA gene and genome sequences.

**Emended description of** ***Rikenellaceae* Krieg et al. 2012**

The description is as before (Krieg et al., 2010b) with the following modification. The genus *Acetobacteroides* has been removed based on the phylogenetic analysis of 16S rRNA gene and genome sequences.

**Emended description of** ***Salinibacteraceae* Munoz et al. 2016 emend. Hahnke et al. 2016**

The description is as before (Hahnke R. L. et al., 2016) with the following modification after inclusion of *Longibacter* and *Longimonas*. Cells are aerobic or facultatively anaerobic. Oxidase reaction is variable. Major polar lipids are diphosphatidylglycerol, diphosphatidylcholin and phosphatidylethanolamine. The major fatty acids include iso-C_15:0_, iso-C_15:0_ 2-OH/C_16:1_ ω7c, C_18:1_ ω7c and summed feature 3 (C_16:1_ ω6c and/or C_16:1_ ω7c). The G+C content as calculated from genome sequences is around 59.3–66.1%; the range provided in the literature is 58.1–67.0 mol%.

**Emended description of** ***Schleiferiaceae* Albuquerque et al. 2011**

The description is as before (Albuquerque et al., 2011) with the following modification after inclusion of *Owenweeksia*. Motility and oxidase activity are variable. Some strains are halophilic and require Na^2+^, Mg^2+^, sea-salts, and either yeast extract or peptone for growth.

**Emended description of** ***Sphingobacteriaceae* Steyn et al. 1998**

The description is as before (Steyn et al., 1998) with the following modification. The family contains the genera *Anseongella, Arcticibacter, Mucilaginibacter, Nubsella, Olivibacter, Parapedobacter, Pedobacter, Pelobium, Pseudopedobacter, Pseudosphingobacterium, Solitalea*, and *Sphingobacterium*, in addition to the newly proposed genera *Albibacterium* gen. nov. and *Daejeonella* gen. nov.

**Emended description of** ***Spirosomaceae* Larkin and Borrall 1978**

The description is as before (Larkin and Borrall, 1978) with the following modifications to account for the genera that have been described in the meantime within the same clade. Cells are Gram-negative, aerobic or facultatively anaerobic, non-spore-forming. Motility is variable. Rods with various degrees of curvature, sometimes resulting in rings, coils, and undulating filaments. Colonies contain a pink or yellow, non-water-soluble pigment. The major menaquinone is MK-7. The major polar lipid is phosphatidylethanolamine. The G+C content as calculated from genome sequences is around 35.1–56.4%; the range provided in the literature is 34.0–64.4 mol%. The family comprises the genera *Arcicella, Arcticibacterium Arsenicibacter, Dyadobacter, Emticicia, Fibrella, Fibrisoma, Flectobacillus, Fluviimonas, Huanghella, Jiulongibacter, Lacihabitans, Larkinella, Leadbetterella, Nibrella, Persicitalea, Pseudarcicella, Ravibacter, Rhabdobacter, Rudanella, Runella, Siphonobacter, Spirosoma* (the type genus), *Taeseokella*, and *Telluribacter*.

**Emended description of** ***Williamwhitmaniaceae* Pikuta et al. 2017**

The description is as before (Pikuta et al., 2017) with the following modification after inclusion of *Acetobacteroides*. Cells are non-motile or motile by gliding.

### Taxonomic Consequences: Emendations of Genera

**Emended description of** ***Algoriella* Yang et al. 2016**

The description is as before (Yang et al., 2015) with the following modification. The genomic G+C content is c. 36–41%, the genome size is c. 3.0–3.5 Mbp.

**Emended description of** ***Alistipes* Rautio et al. 2003**

The description is as before (Rautio et al., 2003) with the following modification. The genomic G+C content is c. 51–61%, the genome size is c. 2.5–3.7 Mbp.

**Emended description of** ***Barnesiella* Sakamoto et al. 2007 emend. Morotomi et al. 2008**

The description is as before (Morotomi et al., 2008) with the following modification. The genomic G+C content is c. 41–55%, the genome size is c. 2.8–3.7 Mbp.

**Emended description of** ***Capnocytophaga* Leadbetter et al. 1982**

The description is as before (Leadbetter et al., 1979) with the following modification. The genomic G+C content is c. 32–47%, the genome size is c. 2.1–3.3 Mbp.

**Emended description of** ***Cellulophaga* Johansen et al. 1999**

The description is as before (Johansen et al., 1999) with the following modification. The genomic G+C content is c. 30–38%, the genome size is c. 3.3–5.1 Mbp.

**Emended description of** ***Cyclobacterium* Raj and Maloy 1990 emend. Shin and Kahng 2017**

The description is as before (Shin and Kahng, 2017) with the following modification. The genomic G+C content is c. 36–51%, the genome size is c. 5.4–6.5 Mbp.

**Emended description of** ***Dyadobacter* Chelius and Triplett 2000 emend. Reddy and Garcia-Pichel 2005**

The description is as before (Reddy and Garcia-Pichel, 2005) with the following modification. The genomic G+C content is c. 42–55%, the genome size is c. 5.1–9.7 Mbp.

**Emended description of** ***Dysgonomonas* Hofstad et al. 2000**

The description is as before (Hofstad et al., 2000) with the following modification. The genomic G+C content is c. 35–44%, the genome size is c. 3.7–5.4 Mbp.

**Emended description of** ***Ekhidna* Alain et al. 2010**

The description is as before (Alain et al., 2010) with the following modification. The genomic G+C content is c. 35–40%, the genome size is c. 4.0–4.5 Mbp.

**Emended description of** ***Formosa* Ivanova et al. 2004 emend. Shakeela et al. 2015**

The description is as before (Shakeela et al., 2015b) with the following modification. The genomic G+C content is c. 31–40%, the genome size is c. 2.9–4.6 Mbp.

**Emended description of** ***Hydrotalea* Kämpfer et al. 2011 emend. Albuquerque et al. 2012**

The description is as before (Albuquerque et al., 2012) with the following modification. The genomic G+C content is c. 32–40%, the genome size is c. 3.0–3.7 Mbp.

**Emended description of** ***Joostella* Quan et al. 2008 emend. Hameed et al. 2014**

The description is as before (Hameed et al., 2014) with the following modification. The genomic G+C content is c. 31–36%, the genome size is c. 4.3–4.8 Mbp.

**Emended description of** ***Lentimicrobium* Sun et al. 2016**

The description is as before (Sun et al., 2016) with the following modification. The genomic G+C content is c. 47–52%, the genome size is c. 4.3–4.8 Mbp.

**Emended description of** ***Mangrovibacterium* Huang et al. 2014**

The description is as before (Huang et al., 2014) with the following modification. The genomic G+C content is c. 41–50%, the genome size is c. 4.4–6.1 Mbp.

**Emended description of** ***Myroides* Vancanneyt et al. 1996 emend. Yan et al. 2012**

The description is as before (Yan et al., 2012) with the following modification after inclusion of some *Flavobacterium* species. Cells are non-motile or motile by gliding. Aerobic or facultatively anaerobic. The temperature range for growth is 6–45°C.

**Emended description of** ***Paludibacter* Ueki et al. 2006**

The description is as before (Ueki et al., 2006) with the following modification. The genomic G+C content is c. 37–45%, the genome size is c. 3.4–3.9 Mbp.

**Emended description of** ***Phocaeicola* Al Masalma et al. 2009**

The description is as before (Al Masalma et al., 2009) with the following modification. Cells do not possess flagella. Exopolysaccharides may be produced.

**Emended description of** ***Proteiniphilum* Chen and Dong 2005 emend. Hahnke et al. 2016**

The description is as before (Hahnke R. L. et al., 2016) with the following modification. The genomic G+C content is c. 41–48%, the genome size is c. 4.2–5.0 Mbp.

**Emended description of** ***Pseudarcicella* Kämpfer et al. 2012**

The description is as before (Kämpfer et al., 2012a) with the following modification. The genomic G+C content is c. 62–67%, the genome size is c. 5.9–6.4 Mbp.

**Emended description of** ***Pseudosphingobacterium* Vaz-Moreira et al. 2007**

The description is as before (Vaz-Moreira et al., 2007) with the following modification. The genomic G+C content is c. 36–41%, the genome size is c. 6.5–7.0 Mbp.

**Emended description of** ***Pustulibacterium* Wang et al. 2013**

The description is as before (Wang G. et al., 2013) with the following modification. The genomic G+C content is c. 35–40%, the genome size is c. 4.0–4.5 Mbp.

**Emended description of** ***Rikenella* Collins et al. 1985**

The description is as before (Collins et al., 1985) with the following modification. The genomic G+C content is c. 55–60%, the genome size is c. 2.6–3.1 Mbp.

**Emended description of** ***Robiginitalea* Cho and Giovannoni 2004 emend. Manh et al. 2008**

The description is as before (Manh et al., 2008) with the following modification. The genomic G+C content is c. 48–58%, the genome size is c. 3.0–3.8 Mbp.

**Emended description of** ***Runella* Larkin and Williams 1978**

The description is as before (Larkin and Williams, 1978) with the following modification. The genomic G+C content is c. 38–49%, the genome size is c. 6.3–7.8 Mbp.

**Emended description of** ***Saccharicrinis* Yang et al. 2014 emend. Liu et al. 2014**

The description is as before (Liu et al., 2014b) with the following modification. The genomic G+C content is c. 35–43%, the genome size is c. 3.4–6.2 Mbp.

**Emended description of** ***Sediminitomix* Khan et al. 2007**

The description is as before (Khan et al., 2007b) with the following modification. The genomic G+C content is c. 36–41%, the genome size is c. 6.4–6.9 Mbp.

**Emended description of** ***Spongiibacterium* Yoon and Oh 2012 emend. Gao et al. 2015**

The description is as before (Gao X. et al., 2015) with the following modification. The genomic G+C content is c. 39–46%, the genome size is c. 3.6–4.7 Mbp.

**Emended description of** ***Verrucomicrobium* Schlesner 1988**

The description is as before (Schlesner, 1987) with the following modification. The genomic G+C content is c. 56–61%, the genome size is c. 8.0–8.5 Mbp.

**Emended description of** ***Xanthomarina* Vaidya et al. 2015**

The description is as before (Vaidya et al., 2015) with the following modification after inclusion of *Formosa spongicola*. The motility is variable.

### Taxonomic Consequences: Emendations of Species

**Emended description of** ***Acetobacteroides hydrogenigenes* Su et al. 2014**

The description is as before (Su et al., 2014) with the following modification. The G+C content of the type-strain genome is 45.7%, its approximate size 3.86 Mbp, its IMG deposit 2731639222.

**Emended description of** ***Algibacter aquaticus* Wong et al. 2017**

The description is as before (Wong et al., 2017) with the following modification. The G+C content of the type-strain genome is 30.6%, its approximate size 4.64 Mbp, its GenBank deposit SAMN05001254.

**Emended description of** ***Algibacter lectus* Nedashkovskaya et al. 2004**

The description is as before (Nedashkovskaya et al., 2004b) with the following restriction. The G+C content of the type-strain genome is 33.3%, its approximate size 4.81 Mbp, its GenBank deposit SAMN04489722.

**Emended description of** ***Algibacter pectinivorans* (Yi et al. 2011) Park et al. 2013**

The description is as before (Park et al., 2013c) with the following modification. The G+C content of the type-strain genome is 33.4%, its approximate size 3.64 Mbp, its GenBank deposit SAMN04487987.

**Emended description of** ***Algoriella xinjiangensis* Yang et al. 2016**

The description is as before (Yang et al., 2015) with the following modification. The G+C content of the type-strain genome is 30.9%, its approximate size 3.27 Mbp, its GenBank deposit SAMN05421738.

**Emended description of** ***Algoriphagus antarcticus* Van Trappen et al. 2004**

The description is as before (Van Trappen et al., 2004a) with the following restriction. The G+C content of the type-strain genome is 40.4%, its approximate size 5.92 Mbp, its IMG deposit 2739367658.

**Emended description of** ***Algoriphagus aquaeductus* Rau et al. 2012**

The description is as before (Rau et al., 2012) with the following modification. The G+C content of the type-strain genome is 44.2%, its approximate size 4.90 Mbp, its IMG deposit 2728369468.

**Emended description of** ***Algoriphagus chordae* Nedashkovskaya et al. 2004**

The description is as before (Nedashkovskaya et al., 2004d) with the following restriction. The G+C content of the type-strain genome is 40.6%, its approximate size 5.18 Mbp, its GenBank deposit SAMN03080604.

**Emended description of** ***Algoriphagus faecimaris* Li et al. 2011**

The description is as before (Li et al., 2011) with the following modification. The G+C content of the type-strain genome is 41.4%, its approximate size 4.65 Mbp, its GenBank deposit SAMN04488104.

**Emended description of** ***Algoriphagus halophilus* (Yi and Chun 2004) Nedashkovskaya et al. 2004**

The description is as before (Nedashkovskaya et al., 2004d) with the following modification. The G+C content of the type-strain genome is 39.4%, its approximate size 4.98 Mbp, its GenBank deposit SAMN05444394.

**Emended description of** ***Algoriphagus hitonicola* Copa-Patiño et al. 2008 emend. Kim et al. 2014**

The description is as before (Kim H. et al., 2014) with the following modification. The G+C content of the type-strain genome is 42.1%, its approximate size 4.32 Mbp, its GenBank deposit SAMN04487988.

**Emended description of** ***Algoriphagus marinus* Han et al. 2017**

The description is as before (Han et al., 2017a) with the following modification. The G+C content of the type-strain genome is 39.5%, its GenBank deposit SAMN06187575.

**Emended description of** ***Algoriphagus ornithinivorans* (Yi and Chun 2004) Nedashkovskaya et al. 2007**

The description is as before (Nedashkovskaya et al., 2007b) with the following modification. The G+C content of the type-strain genome is 39.5%, its approximate size 4.09 Mbp, its GenBank deposit SAMN04488519.

**Emended description of** ***Algoriphagus ratkowskyi* Bowman et al. 2003 emend. Shahina et al. 2014**

The description is as before (Shahina et al., 2014) with the following modification. The G+C content of the type-strain genome is 39.4%, its approximate size 4.99 Mbp, its GenBank deposit SAMN03080613.

**Emended description of** ***Algoriphagus winogradskyi* Nedashkovskaya et al. 2004**

The description is as before (Nedashkovskaya et al., 2004d) with the following restriction. The G+C content of the type-strain genome is 40.1%, its approximate size 4.98 Mbp, its IMG deposit 2724679815.

**Emended description of** ***Alkalitalea saponilacus* Zhao and Chen 2012**

The description is as before (Zhao and Chen, 2012) with the following restriction. The G+C content of the type-strain genome is 39.4%, its approximate size 4.55 Mbp, its GenBank deposit SAMN03080601.

**Emended description of** ***Anaerophaga thermohalophila* Denger et al. 2002**

The description is as before (Denger et al., 2002) with the following restriction. The G+C content of the type-strain genome is 41.3%, its approximate size 4.32 Mbp, its GenBank deposit SAMN02471583.

**Emended description of** ***Aquimarina amphilecti* Kennedy et al. 2014**

The description is as before (Kennedy et al., 2014) with the following modification. The G+C content of the type-strain genome is 32.0%, its approximate size 5.31 Mbp, its GenBank deposit SAMN04487910.

**Emended description of** ***Aquimarina intermedia* Nedashkovskaya et al. 2006**

The description is as before (Nedashkovskaya et al., 2006c) with the following modification. The G+C content of the type-strain genome is 36.1%, its approximate size 3.49 Mbp, its GenBank deposit PRJNA335181.

**Emended description of** ***Aquimarina latercula* (Lewin 1969) Nedashkovskaya et al. 2006**

The description is as before (Nedashkovskaya et al., 2006c) with the following modification. The G+C content of the type-strain genome is 32.2%, its approximate size 6.24 Mbp, its GenBank deposit SAMN02440808.

**Emended description of** ***Aquimarina muelleri* Nedashkovskaya et al. 2005 emend. Yu et al. 2013**

The description is as before (Yu et al., 2013) with the following modification. The G+C content of the type-strain genome is 31.3%, its approximate size 4.90 Mbp, its GenBank deposit SAMN02441476.

**Emended description of** ***Arcicella aurantiaca* Sheu et al. 2010 emend. Chen et al. 2013**

The description is as before (Chen et al., 2013b) with the following modification. The G+C content of the type-strain genome is 35.1%, its approximate size 5.92 Mbp, its GenBank deposit SAMN03080612.

**Emended description of** ***Arenibacter hampyeongensis* Jeong et al. 2013**

The description is as before (Jeong et al., 2013a) with the following modification. The G+C content of the type-strain genome is 38.9%, its approximate size 5.43 Mbp, its GenBank deposit SAMN08100003.

**Emended description of** ***Arenitalea lutea* Zhang et al. 2013**

The description is as before (Zhang X.-Y. et al., 2013) with the following modification. The G+C content of the type-strain genome is 33.3%, its approximate size 3.38 Mbp, its GenBank deposit SAMN02470927.

**Emended description of** ***Aureicoccus marinus* Park et al. 2013**

The description is as before (Park et al., 2013g) with the following modification. The G+C content of the type-strain genome is 45.8%, its approximate size 3.03 Mbp, its GenBank deposit SAMN06075358.

**Emended description of** ***Aureitalea marina* Park et al. 2012**

The description is as before (Park et al., 2012) with the following modification. The G+C content of the type-strain genome is 45.6%, its approximate size 3.07 Mbp, its GenBank deposit SAMN06074325.

**Emended description of** ***Bacteroides acidifaciens* Miyamoto and Itoh 2000**

The description is as before (Miyamoto and Itoh, 2000) with the following restriction. The G+C content of the type-strain genome is 43.0%, its approximate size 4.85 Mbp, its GenBank deposit SAMD00004211.

**Emended description of** ***Bacteroides faecichinchillae* Kitahara et al. 2012**

The description is as before (Kitahara et al., 2012) with the following modification. The G+C content of the type-strain genome is 38.7%, its approximate size 4.78 Mbp, its GenBank deposit SAMD00004031.

**Emended description of** ***Bacteroides faecis* Kim et al. 2010**

The description is as before (Kim M.-S. et al., 2010) with the following restriction. The G+C content of the type-strain genome is 42.4%, its approximate size 6.11 Mbp, its GenBank deposit SAMN02470209.

**Emended description of** ***Bacteroides luti* Hatamoto et al. 2014**

The description is as before (Hatamoto et al., 2014) with the following modification. The G+C content of the type-strain genome is 36.8%, its approximate size 4.12 Mbp, its GenBank deposit SAMN05444405.

**Emended description of** ***Bacteroides reticulotermitis* Sakamoto and Ohkuma 2013**

The description is as before (Sakamoto and Ohkuma, 2013a) with the following modification. The G+C content of the type-strain genome is 43.3%, its approximate size 5.37 Mbp, its GenBank deposit SAMD00011359.

**Emended description of** ***Bacteroides rodentium* Kitahara et al. 2011**

The description is as before (Kitahara et al., 2011) with the following modification. The G+C content of the type-strain genome is 47.1%, its approximate size 4.88 Mbp, its GenBank deposit SAMD00002732.

**Emended description of** ***Bacteroides stercorirosoris* Kitahara et al. 2012**

The description is as before (Kitahara et al., 2012) with the following modification. The G+C content of the type-strain genome is 44.6%, its approximate size 6.27 Mbp, its GenBank deposit SAMN05444350.

**Emended description of** ***Bacteroides suis* Benno et al. 1983**

The description is as before (Benno et al., 1983) with the following restriction. The G+C content of the type-strain genome is 45.7%, its approximate size 3.41 Mbp, its GenBank deposit SAMD00013505.

**Emended description of** ***Bacteroides tectus* Love et al. 1986**

The description is as before (Love et al., 1986) with the following modification. The G+C content of the type-strain genome is 46.2%, its approximate size 3.38 Mbp, its GenBank deposit SAMD00016596.

**Emended description of** ***Belliella buryatensis* Kozyreva et al. 2016**

The description is as before (Kozyreva et al., 2016) with the following addition. The G+C content of the type-strain genome is 38.3%, its approximate size 3.88 Mbp, its GenBank deposit SAMN06295967.

**Emended description of** ***Belliella pelovolcani* Arun et al. 2009**

The description is as before (Arun et al., 2009) with the following restriction. The G+C content of the type-strain genome is 38.6%, its approximate size 4.11 Mbp, its GenBank deposit SAMN05421761.

**Emended description of** ***Bizionia paragorgiae* Nedashkovskaya et al. 2005**

The description is as before (Nedashkovskaya et al., 2005d) with the following modification. The G+C content of the type-strain genome is 35.3%, its approximate size 3.14 Mbp, its GenBank deposit SAMN04487990.

**Emended description of** ***Capnocytophaga leadbetteri* Frandsen et al. 2008**

The description is as before (Frandsen et al., 2008) with the following modification. The G+C content of the type-strain genome is 39.8%, its approximate size 2.64 Mbp, its IMG deposit 2734482253.

**Emended description of** ***Cecembia rubra* Duan et al. 2015**

The description is as before (Duan et al., 2015) with the following modification. The G+C content of the type-strain genome is 39.5%, its approximate size 4.75 Mbp, its IMG deposit 2728369475.

**Emended description of** ***Cellulophaga baltica* Johansen et al. 1999**

The description is as before (Johansen et al., 1999) with the following modification. The G+C content of the type-strain genome is 34.5%, its approximate size 4.71 Mbp, its GenBank deposit SAMN04487992.

**Emended description of** ***Cephaloticoccus primus* Lin et al. 2016**

The description is as before (Lin et al., 2016) with the following modification. The G+C content of the type-strain genome is 63.0%, its approximate size 2.36 Mbp, its GenBank deposit SAMN04456986.

**Emended description of** ***Cesiribacter andamanensis* Srinivas et al. 2011**

The description is as before (Srinivas et al., 2011) with the following modification. The G+C content of the type-strain genome is 54.6%, its approximate size 4.76 Mbp, its GenBank deposit SAMN02469440.

**Emended description of** ***Chitinophaga arvensicola* (Oyaizu et al. 1983) Kämpfer et al. 2006 emend. Pankratov et al. 2006**

The description is as before (Pankratov et al., 2006) with the following restriction. The G+C content of the type-strain genome is 46.6%, its approximate size 8.32 Mbp, its GenBank deposit SAMN04488122.

**Emended description of** ***Chitinophaga dinghuensis* Lv et al. 2015**

The description is as before (Lv et al., 2015) with the following modification. The G+C content of the type-strain genome is 44.7%, its approximate size 7.12 Mbp, its IMG deposit 2731639224.

**Emended description of** ***Chitinophaga eiseniae* Yasir et al. 2011**

The description is as before (Yasir et al., 2011) with the following modification. The G+C content of the type-strain genome is 50.4%, its approximate size 8.55 Mbp, its GenBank deposit SAMN04488128.

**Emended description of** ***Chitinophaga ginsengisoli* Lee et al. 2007**

The description is as before (Lee et al., 2007) with the following modification. The G+C content of the type-strain genome is 45.5%, its approximate size 8.50 Mbp, its IMG deposit 2728369473.

**Emended description of** ***Chitinophaga jiangningensis* Wang et al. 2014**

The description is as before (Wang et al., 2014) with the following modification. The G+C content of the type-strain genome is 47.4%, its approximate size 7.18 Mbp, its GenBank deposit SAMN05444266.

**Emended description of** ***Chitinophaga rupis* Lee et al. 2009**

The description is as before (Lee D. W. et al., 2009) with the following modification. The G+C content of the type-strain genome is 47.5%, its approximate size 8.39 Mbp, its GenBank deposit SAMN04488505.

**Emended description of** ***Chitinophaga skermanii* Kämpfer et al. 2006**

The description is as before (Kämpfer et al., 2006) with the following modification. The G+C content of the type-strain genome is 41.8%, its approximate size 6.11 Mbp, its GenBank deposit SAMN05660647.

**Emended description of** ***Chlamydia caviae* (Everett et al. 1999) Sachse et al. 2015**

The description is as before (Sachse et al., 2015) with the following addition. The G+C content of the type-strain genome is 39.2%, its approximate size 1.18 Mbp, its GenBank deposit SAMN02603993.

**Emended description of** ***Chlamydia pecorum* Fukushi and Hirai 1992**

The description is as before (Fukushi and Hirai, 1992) with the following modification. The G+C content of the type-strain genome is 41.1%, its approximate size 1.11 Mbp, its GenBank deposit SAMN02603383.

**Emended description of** ***Chlamydia psittaci* (Lillie 1930) Page 1968**

The description is as before (Page, 1968) with the following addition. The G+C content of the type-strain genome is 39.0%, its approximate size 1.18 Mbp, its GenBank deposit SAMN02603382.

**Emended description of** ***Chlamydia trachomatis* (Busacca 1935) Rake 1957 emend. Everett et al. 1999**

The description is as before (Everett et al., 1999) with the following addition. The G+C content of the type-strain genome is 41.3%, its approximate size 1.05 Mbp, its GenBank deposit SAMN02603498.

**Emended description of** ***Chlorobaculum limnaeum* Imhoff 2003**

The description is as before (Imhoff, 2003) with the following addition. The G+C content of the type-strain genome is 56.4%, its approximate size 2.80 Mbp, its GenBank deposit SAMN05793943.

**Emended description of** ***Chryseobacterium arachidiradicis* Kämpfer et al. 2015**

The description is as before (Kämpfer et al., 2015a) with the following addition. The G+C content of the type-strain genome is 36.2%, its approximate size 3.65 Mbp, its IMG deposit 2728369265.

**Emended description of** ***Chryseobacterium arachidis* Kämpfer et al. 2014**

The description is as before (Kämpfer et al., 2014b) with the following addition. The G+C content of the type-strain genome is 35.6%, its approximate size 4.95 Mbp, its GenBank deposit SAMN05443633.

**Emended description of** ***Chryseobacterium arthrosphaerae* Kämpfer et al. 2010 emend. Jeong et al. 2017**

The description is as before (Jeong et al., 2017) with the following addition. The G+C content of the type-strain genome is 38.3%, its approximate size 5.05 Mbp, its GenBank deposit SAMN05366337.

**Emended description of** ***Chryseobacterium balustinum* (Harrison 1929) Vandamme et al. 1994**

The description is as before (Vandamme et al., 1994) with the following addition. The G+C content of the type-strain genome is 33.6%, its approximate size 4.86 Mbp, its GenBank deposit SAMN05421800.

**Emended description of** ***Chryseobacterium chaponense* Kämpfer et al. 2011**

The description is as before (Kämpfer et al., 2011a) with the following addition. The G+C content of the type-strain genome is 35.3%, its approximate size 3.04 Mbp, its GenBank deposit SAMN05421789.

**Emended description of** ***Chryseobacterium contaminans* Kämpfer et al. 2014**

The description is as before (Kämpfer et al., 2014c) with the following addition. The G+C content of the type-strain genome is 35.9%, its approximate size 4.70 Mbp, its GenBank deposit SAMN05366335.

**Emended description of** ***Chryseobacterium culicis* Kämpfer et al. 2010**

The description is as before (Kämpfer et al., 2010a) with the following addition. The G+C content of the type-strain genome is 37.3%, its approximate size 4.90 Mbp, its GenBank deposit SAMN05421593.

**Emended description of** ***Chryseobacterium defluvii* Kämpfer et al. 2003 emend. Montero-Calasanz et al. 2013**

The description is as before (Montero-Calasanz et al., 2013) with the following addition. The G+C content of the type-strain genome is 36.6%, its approximate size 3.71 Mbp, its GenBank deposit SAMN05421836.

**Emended description of** ***Chryseobacterium gambrini* Herzog et al. 2008 emend. Montero-Calasanz et al. 2014**

The description is as before (Montero-Calasanz et al., 2014) with the following modification. The G+C content of the type-strain genome is 36.1%, its approximate size 4.84 Mbp, its GenBank deposit SAMN05421785.

**Emended description of** ***Chryseobacterium geocarposphaerae* Kämpfer et al. 2014**

The description is as before (Kämpfer et al., 2014b) with the following addition. The G+C content of the type-strain genome is 35.3%, its approximate size 4.11 Mbp, its IMG deposit 2734482070.

**Emended description of** ***Chryseobacterium hungaricum* Szoboszlay et al. 2008**

The description is as before (Szoboszlay et al., 2008) with the following modification. The G+C content of the type-strain genome is 36.5%, its approximate size 3.96 Mbp, its GenBank deposit SAMN05421825.

**Emended description of** ***Chryseobacterium indoltheticum* (Campbell and Williams 1951) Vandamme et al. 1994 emend. Wu et al. 2013**

The description is as before (Wu et al., 2013) with the following addition. The G+C content of the type-strain genome is 34.2%, its approximate size 4.21 Mbp, its GenBank deposit SAMN05421682.

**Emended description of** ***Chryseobacterium jejuense* Weon et al. 2008 emend. Montero-Calasanz et al. 2014**

The description is as before (Montero-Calasanz et al., 2014) with the following modification. The G+C content of the type-strain genome is 35.3%, its approximate size 5.17 Mbp, its GenBank deposit SAMN05421542.

**Emended description of** ***Chryseobacterium joostei* Hugo et al. 2003 emend. Montero-Calasanz et al. 2014**

The description is as before (Montero-Calasanz et al., 2014) with the following modification. The G+C content of the type-strain genome is 35.4%, its approximate size 5.27 Mbp, its GenBank deposit SAMN05421768.

**Emended description of** ***Chryseobacterium lactis* Holmes et al. 2013**

The description is as before (Holmes et al., 2013) with the following modification. The G+C content of the type-strain genome is 36.1%, its approximate size 5.59 Mbp, its GenBank deposit SAMN08324239.

**Emended description of** ***Chryseobacterium limigenitum* Kämpfer et al. 2015**

The description is as before (Kämpfer et al., 2015e) with the following addition. The G+C content of the type-strain genome is 34.4%, its approximate size 4.70 Mbp, its GenBank deposit SAMN05216324.

**Emended description of** ***Chryseobacterium molle* Herzog et al. 2008**

The description is as before (Herzog et al., 2008) with the following modification. The G+C content of the type-strain genome is 37.5%, its approximate size 3.70 Mbp, its GenBank deposit SAMN05444371.

**Emended description of** ***Chryseobacterium oranimense* Hantsis-Zacharov et al. 2008**

The description is as before (Hantsis-Zacharov et al., 2008) with the following addition. The G+C content of the type-strain genome is 37.9%, its approximate size 4.60 Mbp, its GenBank deposit SAMN05421866.

**Emended description of** ***Chryseobacterium pallidum* Herzog et al. 2008**

The description is as before (Herzog et al., 2008) with the following modification. The G+C content of the type-strain genome is 36.1%, its approximate size 3.83 Mbp, its GenBank deposit SAMN05421679.

**Emended description of** ***Chryseobacterium piscicola* Ilardi et al. 2009**

The description is as before (Ilardi et al., 2009) with the following modification. The G+C content of the type-strain genome is 33.8%, its approximate size 3.45 Mbp, its GenBank deposit SAMN05421796.

**Emended description of** ***Chryseobacterium polytrichastri* Chen et al. 2015**

The description is as before (Chen et al., 2015) with the following modification. The G+C content of the type-strain genome is 33.7%, its approximate size 5.14 Mbp, its GenBank deposit SAMN05444267.

**Emended description of** ***Chryseobacterium profundimaris* Xu et al. 2015**

The description is as before (Xu et al., 2015) with the following modification. The G+C content of the type-strain genome is 36.7%, its approximate size 4.33 Mbp, its IMG deposit 2724679817.

**Emended description of** ***Chryseobacterium rhizoplanae* Kämpfer et al. 2015**

The description is as before (Kämpfer et al., 2015d) with the following addition. The G+C content of the type-strain genome is 36.4%, its approximate size 5.18 Mbp, its IMG deposit 2724679795.

**Emended description of** ***Chryseobacterium scophthalmum* (Mudarris et al. 1994) Vandamme et al. 1994**

The description is as before (Vandamme et al., 1994) with the following restriction. The G+C content of the type-strain genome is 33.5%, its approximate size 4.47 Mbp, its GenBank deposit SAMN05421769.

**Emended description of** ***Chryseobacterium soldanellicola* Park et al. 2006**

The description is as before (Park M. S. et al., 2006) with the following modification. The G+C content of the type-strain genome is 35.4%, its approximate size 4.14 Mbp, its GenBank deposit SAMN05421664.

**Emended description of** ***Chryseobacterium taeanense* Park et al. 2006**

The description is as before (Park M. S. et al., 2006) with the following modification. The G+C content of the type-strain genome is 36.0%, its approximate size 3.94 Mbp, its GenBank deposit SAMN05421846.

**Emended description of** ***Chryseobacterium taichungense* Shen et al. 2005**

The description is as before (Shen et al., 2005) with the following addition. The G+C content of the type-strain genome is 36.5%, its approximate size 4.48 Mbp, its GenBank deposit SAMN05421856.

**Emended description of** ***Chryseobacterium taihuense* Wu et al. 2013**

The description is as before (Wu et al., 2013) with the following modification. The G+C content of the type-strain genome is 35.2%, its approximate size 3.40 Mbp, its GenBank deposit SAMN05216273.

**Emended description of** ***Chryseobacterium taklimakanense* (Peng et al. 2009) Holmes et al. 2013 emend. Kim et al. 2016**

The description is as before (Kim et al., 2016) with the following addition. The G+C content of the type-strain genome is 40.4%, its approximate size 2.76 Mbp, its GenBank deposit SAMEA4412677.

**Emended description of** ***Chryseobacterium treverense* Yassin et al. 2010**

The description is as before (Yassin et al., 2010) with the following addition. The G+C content of the type-strain genome is 39.5%, its approximate size 2.38 Mbp, its GenBank deposit SAMN05421638.

**Emended description of** ***Chryseobacterium ureilyticum* Herzog et al. 2008 emend. Montero-Calasanz et al. 2014**

The description is as before (Montero-Calasanz et al., 2014) with the following modification. The G+C content of the type-strain genome is 34.7%, its approximate size 5.18 Mbp, its GenBank deposit SAMN05421786.

**Emended description of** ***Chryseobacterium wanjuense* Weon et al. 2006 emend. Montero-Calasanz et al. 2013**

The description is as before (Montero-Calasanz et al., 2013) with the following modification. The G+C content of the type-strain genome is 36.0%, its approximate size 4.67 Mbp, its GenBank deposit SAMN05421841.

**Emended description of** ***Chryseobacterium zeae* Kämpfer et al. 2014**

The description is as before (Kämpfer et al., 2014b) with the following addition. The G+C content of the type-strain genome is 35.1%, its approximate size 4.08 Mbp, its GenBank deposit SAMN05444409.

**Emended description of** ***Chryseolinea serpens* Kim et al. 2013**

The description is as before (Kim J.-J. et al., 2013) with the following modification. The G+C content of the type-strain genome is 51.1%, its approximate size 8.36 Mbp, its GenBank deposit SAMN04488109.

**Emended description of** ***Cloacibacterium normanense* Allen et al. 2006**

The description is as before (Allen et al., 2006) with the following modification. The G+C content of the type-strain genome is 33.1%, its approximate size 2.74 Mbp, its GenBank deposit SAMN04489756.

**Emended description of** ***Deinococcus actinosclerus* Joo et al. 2016**

The description is as before (Joo et al., 2016) with the following modification. The G+C content of the type-strain genome is 70.6%, its approximate size 3.26 Mbp, its GenBank deposit SAMN04325383.

**Emended description of** ***Deinococcus aquatilis* Kämpfer et al. 2008**

The description is as before (Kämpfer et al., 2008) with the following addition. The G+C content of the type-strain genome is 62.1%, its approximate size 5.00 Mbp, its GenBank deposit SAMN02440995.

**Emended description of** ***Deinococcus deserti* de Groot et al. 2005**

The description is as before (de Groot et al., 2005) with the following modification. The G+C content of the type-strain genome is 63.0%, its approximate size 3.86 Mbp, its GenBank deposit SAMN02603250.

**Emended description of** ***Deinococcus ficus* Lai et al. 2006 emend. Kämpfer 2009**

The description is as before (Kämpfer, 2009) with the following addition. The G+C content of the type-strain genome is 70.0%, its approximate size 4.15 Mbp, its GenBank deposit SAMN02441464.

**Emended description of** ***Deinococcus gobiensis* Yuan et al. 2009**

The description is as before (Yuan et al., 2009) with the following modification. The G+C content of the type-strain genome is 69.2%, its approximate size 4.41 Mbp, its GenBank deposit SAMN02603039.

**Emended description of** ***Deinococcus hopiensis* Rainey and da Costa 2005**

The description is as before (Rainey et al., 2005) with the following modification. The G+C content of the type-strain genome is 64.9%, its approximate size 6.65 Mbp, its GenBank deposit SAMN00790413.

**Emended description of** ***Deinococcus maricopensis* Rainey and da Costa 2005**

The description is as before (Rainey et al., 2005) with the following modification. The G+C content of the type-strain genome is 69.8%, its approximate size 3.50 Mbp, its GenBank deposit SAMN00713579.

**Emended description of** ***Deinococcus marmoris* Hirsch et al. 2006**

The description is as before (Hirsch et al., 2004) with the following restriction. The G+C content of the type-strain genome is 64.4%, its approximate size 4.80 Mbp, its GenBank deposit SAMN02841164.

**Emended description of** ***Deinococcus misasensis* Asker et al. 2008**

The description is as before (Asker et al., 2008a) with the following modification. The G+C content of the type-strain genome is 55.2%, its approximate size 5.08 Mbp, its GenBank deposit SAMN02746038.

**Emended description of** ***Deinococcus phoenicis* Vaishampayan et al. 2014**

The description is as before (Vaishampayan et al., 2014) with the following addition. The G+C content of the type-strain genome is 69.0%, its approximate size 3.81 Mbp, its GenBank deposit SAMN02690678.

**Emended description of** ***Deinococcus pimensis* Rainey and da Costa 2005**

The description is as before (Rainey et al., 2005) with the following modification. The G+C content of the type-strain genome is 69.9%, its approximate size 4.68 Mbp, its GenBank deposit SAMN02584908.

**Emended description of** ***Deinococcus proteolyticus* (ex Kobatake et al. 1973) Brooks and Murray 1981**

The description is as before (Brooks and Murray, 1981) with the following modification. The G+C content of the type-strain genome is 66.2%, its approximate size 2.15 Mbp, its GenBank deposit SAMN00016729.

**Emended description of** ***Deinococcus puniceus* Lee et al. 2017**

The description is as before (Lee et al., 2015) with the following modification. The G+C content of the type-strain genome is 62.6%, its approximate size 2.97 Mbp, its GenBank deposit SAMN03285365.

**Emended description of** ***Deinococcus reticulitermitis* Chen et al. 2012**

The description is as before (Chen et al., 2012) with the following modification. The G+C content of the type-strain genome is 68.6%, its approximate size 3.50 Mbp, its GenBank deposit SAMN04488058.

**Emended description of** ***Deinococcus soli* Cha et al. 2016**

The description is as before (Cha et al., 2014) with the following modification. The G+C content of the type-strain genome is 70.2%, its approximate size 3.24 Mbp, its GenBank deposit SAMN03287573.

**Emended description of** ***Deinococcus xibeiensis* Wang et al. 2010**

The description is as before (Wang et al., 2010) with the following modification. The G+C content of the type-strain genome is 66.5%, its approximate size 3.29 Mbp, its GenBank deposit SAMN02470569.

**Emended description of** ***Dokdonia pacifica* Zhang et al. 2015**

The description is as before (Zhang et al., 2015b) with the following modification. The G+C content of the type-strain genome is 34.1%, its approximate size 5.52 Mbp, its IMG deposit 2724679821.

**Emended description of** ***Dyadobacter jiangsuensis* Wang et al. 2015**

The description is as before (Wang et al., 2015) with the following modification. The G+C content of the type-strain genome is 50.3%, its approximate size 8.27 Mbp, its IMG deposit 2728369267.

**Emended description of** ***Dyadobacter koreensis* Baik et al. 2007**

The description is as before (Baik et al., 2007a) with the following modification. The G+C content of the type-strain genome is 41.3%, its approximate size 7.34 Mbp, its GenBank deposit SAMN04487995.

**Emended description of** ***Dyadobacter psychrophilus* Zhang et al. 2010**

The description is as before (Zhang et al., 2010a) with the following modification. The G+C content of the type-strain genome is 45.1%, its approximate size 6.73 Mbp, its GenBank deposit SAMN05660293.

**Emended description of** ***Dyadobacter soli* Lee et al. 2010**

The description is as before (Lee M. et al., 2010) with the following modification. The G+C content of the type-strain genome is 50.5%, its approximate size 8.74 Mbp, its GenBank deposit SAMN04487996.

**Emended description of** ***Dyadobacter tibetensis* Shen et al. 2013**

The description is as before (Shen et al., 2013) with the following modification. The G+C content of the type-strain genome is 43.4%, its approximate size 5.31 Mbp, its GenBank deposit SAMN02951879.

**Emended description of** ***Dysgonomonas hofstadii* Lawson et al. 2010**

The description is as before (Lawson et al., 2010) with the following modification. The G+C content of the type-strain genome is 39.5%, its approximate size 5.04 Mbp, its GenBank deposit SAMD00016814.

**Emended description of** ***Dysgonomonas macrotermitis* Yang et al. 2014**

The description is as before (Yang Y. et al., 2014) with the following modification. The G+C content of the type-strain genome is 38.5%, its approximate size 4.66 Mbp, its GenBank deposit SAMN05444362.

**Emended description of** ***Ekhidna lutea* Alain et al. 2010**

The description is as before (Alain et al., 2010) with the following modification. The G+C content of the type-strain genome is 40.3%, its approximate size 4.23 Mbp, its GenBank deposit SAMN05421640.

**Emended description of** ***Fibrobacter intestinalis* Montgomery et al. 1988**

The description is as before (Montgomery et al., 1988) with the following modification. The G+C content of the type-strain genome is 47.8%, its approximate size 3.29 Mbp, its GenBank deposit SAMN02745108.

**Emended description of** ***Fibrobacter succinogenes* (Hungate 1950) Montgomery et al. 1988**

The description is as before (Montgomery et al., 1988) with the following restriction. The G+C content of the type-strain genome is 48.0%, its approximate size 3.84 Mbp, its GenBank deposit SAMN02603971.

**Emended description of** ***Filimonas lacunae* Shiratori et al. 2009 emend. Leandro et al. 2013**

The description is as before (Leandro et al., 2013) with the following modification. The G+C content of the type-strain genome is 44.0%, its approximate size 7.81 Mbp, its GenBank deposit SAMD00045010.

**Emended description of** ***Fimbriiglobus ruber* Kulichevskaya et al. 2017**

The description is as before (Kulichevskaya et al., 2017) with the following modification. The G+C content of the type-strain genome is 64.2%, its approximate size 12.36 Mbp, its GenBank deposit SAMN07187746.

**Emended description of** ***Flaviramulus basaltis* Einen and Øvreås 2006 emend. Zhang et al. 2013**

The description is as before (Zhang Y. et al., 2013) with the following restriction. The G+C content of the type-strain genome is 31.1%, its approximate size 4.19 Mbp, its GenBank deposit SAMN05428642.

**Emended description of** ***Flavisolibacter tropicus* Lee et al. 2016**

The description is as before (Lee et al., 2016b) with the following modification. The G+C content of the type-strain genome is 41.5%, its approximate size 5.94 Mbp, its GenBank deposit SAMN03287577.

**Emended description of** ***Flavobacterium anhuiense* Liu et al. 2008**

The description is as before (Liu et al., 2008) with the following modification. The G+C content of the type-strain genome is 34.4%, its approximate size 5.34 Mbp, its GenBank deposit SAMN02927916.

**Emended description of** ***Flavobacterium aquicola* Hatayama et al. 2016**

The description is as before (Hatayama et al., 2016) with the following modification. The G+C content of the type-strain genome is 34.2%, its approximate size 5.12 Mbp, its IMG deposit 2739367662.

**Emended description of** ***Flavobacterium araucananum* Kämpfer et al. 2012 emend. Loch and Faisal 2014**

The description is as before (Loch and Faisal, 2014b) with the following addition. The G+C content of the type-strain genome is 34.4%, its approximate size 6.01 Mbp, its GenBank deposit SAMN05444378.

**Emended description of** ***Flavobacterium branchiophilum* Wakabayashi et al. 1989 emend. Bernardet et al. 1996**

The description is as before (Bernardet et al., 1996) with the following modification. The G+C content of the type-strain genome is 32.8%, its approximate size 3.58 Mbp, its GenBank deposit SAMN06049065.

**Emended description of** ***Flavobacterium caeni* Liu et al. 2010 emend. Fujii et al. 2014**

The description is as before (Fujii et al., 2014) with the following restriction. The G+C content of the type-strain genome is 49.0%, its approximate size 3.69 Mbp, its GenBank deposit SAMN02927903.

**Emended description of** ***Flavobacterium cheniae* Qu et al. 2008 emend. Joung et al. 2013**

The description is as before (Joung et al., 2013) with the following modification. The G+C content of the type-strain genome is 32.5%, its approximate size 2.74 Mbp, its IMG deposit 2757320365.

**Emended description of** ***Flavobacterium chilense* Kämpfer et al. 2012**

The description is as before (Kämpfer et al., 2012b) with the following addition. The G+C content of the type-strain genome is 33.7%, its approximate size 5.92 Mbp, its GenBank deposit SAMN04506025.

**Emended description of** ***Flavobacterium commune* Ekwe and Kim 2018**

The description is as before (Ekwe and Kim, 2018) with the following modification. The G+C content of the type-strain genome is 34.4%, its approximate size 3.85 Mbp, its GenBank deposit SAMN05804760.

**Emended description of** ***Flavobacterium croceum* Park et al. 2006**

The description is as before (Park M. et al., 2006) with the following modification. The G+C content of the type-strain genome is 32.1%, its approximate size 2.95 Mbp, its IMG deposit 2734482249.

**Emended description of** ***Flavobacterium cucumis* Weon et al. 2007**

The description is as before (Weon et al., 2007) with the following modification. The G+C content of the type-strain genome is 33.1%, its approximate size 2.85 Mbp, its GenBank deposit SAMN05443547.

**Emended description of** ***Flavobacterium cutihirudinis* Glaeser et al. 2013**

The description is as before (Glaeser et al., 2013a) with the following addition. The G+C content of the type-strain genome is 33.9%, its approximate size 4.90 Mbp, its GenBank deposit SAMN05444268.

**Emended description of** ***Flavobacterium endophyticum* Gao et al. 2015**

The description is as before (Gao J. et al., 2015) with the following modification. The G+C content of the type-strain genome is 39.1%, its approximate size 3.74 Mbp, its IMG deposit 2731639186.

**Emended description of** ***Flavobacterium gillisiae* McCammon and Bowman 2000**

The description is as before (McCammon and Bowman, 2000) with the following modification. The G+C content of the type-strain genome is 34.3%, its approximate size 4.38 Mbp, its GenBank deposit SAMN05443667.

**Emended description of** ***Flavobacterium glaciei* Zhang et al. 2006**

The description is as before (Zhang et al., 2006) with the following modification. The G+C content of the type-strain genome is 33.4%, its approximate size 3.14 Mbp, its IMG deposit 2770939517.

**Emended description of** ***Flavobacterium glycines* Madhaiyan et al. 2010**

The description is as before (Madhaiyan et al., 2010a) with the following modification. The G+C content of the type-strain genome is 34.1%, its approximate size 3.96 Mbp, its GenBank deposit SAMN04570198.

**Emended description of** ***Flavobacterium haoranii* Zhang et al. 2010 emend. Sheu et al. 2013**

The description is as before (Sheu et al., 2013) with the following modification. The G+C content of the type-strain genome is 31.1%, its approximate size 2.85 Mbp, its GenBank deposit SAMN05444337.

**Emended description of** ***Flavobacterium lindanitolerans* Jit et al. 2008**

The description is as before (Jit et al., 2008) with the following modification. The G+C content of the type-strain genome is 37.9%, its approximate size 3.72 Mbp, its IMG deposit 2731639123.

**Emended description of** ***Flavobacterium micromati* Van Trappen et al. 2004**

The description is as before (Van Trappen et al., 2004b) with the following restriction. The G+C content of the type-strain genome is 33.1%, its approximate size 3.69 Mbp, its GenBank deposit SAMN05444372.

**Emended description of** ***Flavobacterium nitrogenifigens* Kämpfer et al. 2015**

The description is as before (Kämpfer et al., 2015c) with the following addition. The G+C content of the type-strain genome is 34.1%, its approximate size 5.43 Mbp, its IMG deposit 2724679792.

**Emended description of** ***Flavobacterium noncentrifugens* Zhu et al. 2013**

The description is as before (Zhu et al., 2013) with the following modification. The G+C content of the type-strain genome is 40.8%, its approximate size 4.03 Mbp, its GenBank deposit SAMN04487935.

**Emended description of** ***Flavobacterium saccharophilum* (Reichenbach 1989) Bernardet et al. 1996**

The description is as before (Bernardet et al., 1996) with the following restriction. The G+C content of the type-strain genome is 34.0%, its approximate size 5.21 Mbp, its GenBank deposit SAMN05444366.

**Emended description of** ***Flavobacterium segetis* Yi and Chun 2006**

The description is as before (Yi and Chun, 2006) with the following modification. The G+C content of the type-strain genome is 32.6%, its approximate size 3.46 Mbp, its GenBank deposit SAMN05444396.

**Emended description of** ***Flavobacterium sinopsychrotolerans* Xu et al. 2011**

The description is as before (Xu et al., 2011) with the following modification. The G+C content of the type-strain genome is 34.2%, its approximate size 3.52 Mbp, its GenBank deposit SAMN04487942.

**Emended description of** ***Flavobacterium spartansii* Loch and Faisal 2014**

The description is as before (Loch and Faisal, 2014a) with the following addition. The G+C content of the type-strain genome is 35.7%, its approximate size 5.35 Mbp, its GenBank deposit SAMN06049056.

**Emended description of** ***Flavobacterium succinicans* (Reichenbach 1989) Bernardet et al. 1996**

The description is as before (Bernardet et al., 1996) with the following restriction. The G+C content of the type-strain genome is 35.5%, its approximate size 3.66 Mbp, its GenBank deposit SAMN05444143.

**Emended description of** ***Flavobacterium terrae* Weon et al. 2007 emend. Sheu et al. 2013**

The description is as before (Sheu et al., 2013) with the following modification. The G+C content of the type-strain genome is 31.8%, its approximate size 3.16 Mbp, its GenBank deposit SAMN05444363.

**Emended description of** ***Flavobacterium urumqiense* Dong et al. 2012**

The description is as before (Dong et al., 2012) with the following modification. The G+C content of the type-strain genome is 33.5%, its approximate size 3.44 Mbp, its GenBank deposit SAMN04488130.

**Emended description of** ***Flavobacterium weaverense* Yi and Chun 2006**

The description is as before (Yi and Chun, 2006) with the following modification. The G+C content of the type-strain genome is 32.8%, its approximate size 3.40 Mbp, its GenBank deposit SAMN05443664.

**Emended description of** ***Flavobacterium xanthum* (ex Inoue and Komagata 1976) McCammon and Bowman 2000**

The description is as before (McCammon and Bowman, 2000) with the following modification. The G+C content of the type-strain genome is 34.4%, its approximate size 3.76 Mbp, its GenBank deposit SAMN05443669.

**Emended description of** ***Flavobacterium xueshanense* Dong et al. 2012**

The description is as before (Dong et al., 2012) with the following modification. The G+C content of the type-strain genome is 34.1%, its approximate size 3.47 Mbp, its GenBank deposit SAMN04488131.

**Emended description of** ***Flexibacter flexilis* Soriano 1945**

The description is as before (Soriano, 1945) with the following addition. The G+C content of the type-strain genome is 42.4%, its approximate size 4.25 Mbp, its GenBank deposit SAMN05421780.

**Emended description of** ***Fontibacter flavus* Kämpfer et al. 2010**

The description is as before (Kämpfer et al., 2010b) with the following modification. The G+C content of the type-strain genome is 39.8%, its approximate size 4.49 Mbp, its GenBank deposit SAMN05660746.

**Emended description of** ***Formosa algae* Ivanova et al. 2004 emend. Nedashkovskaya et al. 2006**

The description is as before (Nedashkovskaya et al., 2006b) with the following modification. The G+C content of the type-strain genome is 33.4%, its approximate size 4.29 Mbp, its GenBank deposit SAMN04202632.

**Emended description of** ***Fulvivirga imtechensis* Nupur et al. 2012**

The description is as before (Nupur et al., 2012) with the following modification. The G+C content of the type-strain genome is 42.4%, its approximate size 6.74 Mbp, its GenBank deposit SAMN02469986.

**Emended description of** ***Gelidibacter algens* Bowman et al. 1997**

The description is as before (Bowman et al., 1997) with the following restriction. The G+C content of the type-strain genome is 37.2%, its approximate size 4.45 Mbp, its GenBank deposit SAMN05660858.

**Emended description of** ***Gelidibacter sediminis* Zhang and Margesin 2015**

The description is as before (Zhang and Margesin, 2015) with the following modification. The G+C content of the type-strain genome is 36.9%, its approximate size 3.56 Mbp, its IMG deposit 2728369467.

**Emended description of** ***Gemmatimonas aurantiaca* Zhang et al. 2003 emend. Zeng et al. 2015**

The description is as before (Zeng et al., 2015) with the following modification. The G+C content of the type-strain genome is 64.3%, its approximate size 4.64 Mbp, its GenBank deposit SAMD00060909.

**Emended description of** ***Geofilum rhodophaeum* Mu et al. 2017**

The description is as before (Mu et al., 2017) with the following restriction. The G+C content of the type-strain genome is 48.9%, its approximate size 4.15 Mbp, its GenBank deposit SAMN06473345.

**Emended description of** ***Geofilum rubicundum* Miyazaki et al. 2012**

The description is as before (Miyazaki et al., 2012) with the following modification. The G+C content of the type-strain genome is 44.8%, its approximate size 4.92 Mbp, its GenBank deposit SAMD00000449.

**Emended description of** ***Gillisia mitskevichiae* Nedashkovskaya et al. 2005**

The description is as before (Nedashkovskaya et al., 2005c) with the following modification. The G+C content of the type-strain genome is 34.1%, its approximate size 3.59 Mbp, its GenBank deposit SAMN05444482.

**Emended description of** ***Gracilimonas mengyeensis* Wang et al. 2013**

The description is as before (Wang Y.-X. et al., 2013) with the following modification. The G+C content of the type-strain genome is 44.4%, its approximate size 4.67 Mbp, its IMG deposit 2724679790.

**Emended description of** ***Hanstruepera neustonica* Hameed et al. 2015**

The description is as before (Hameed et al., 2015) with the following modification. The G+C content of the type-strain genome is 35.4%, its approximate size 3.05 Mbp, its GenBank deposit SAMN08348338.

**Emended description of** ***Hydrotalea sandarakina* Albuquerque et al. 2012**

The description is as before (Albuquerque et al., 2012) with the following modification. The G+C content of the type-strain genome is 35.8%, its approximate size 3.26 Mbp, its GenBank deposit SAMN05660754.

**Emended description of** ***Hymenobacter arizonensis* Reddy and Garcia-Pichel 2013**

The description is as before (Reddy and Garcia-Pichel, 2013) with the following restriction. The G+C content of the type-strain genome is 59.3%, its approximate size 6.02 Mbp, its GenBank deposit SAMN04515668.

**Emended description of** ***Hymenobacter gelipurpurascens* Buczolits et al. 2006**

The description is as before (Buczolits et al., 2006) with the following restriction. The G+C content of the type-strain genome is 57.0%, its approximate size 5.06 Mbp, its IMG deposit 2728369218.

**Emended description of** ***Hymenobacter sedentarius* Lee et al. 2016**

The description is as before (Lee et al., 2016a) with the following modification. The G+C content of the type-strain genome is 61.0%, its approximate size 4.87 Mbp, its GenBank deposit SAMN04325376.

**Emended description of** ***Hyunsoonleella jejuensis* Yoon et al. 2010 emend. Park et al. 2013**

The description is as before (Park et al., 2013a) with the following modification. The G+C content of the type-strain genome is 34.6%, its approximate size 3.48 Mbp, its GenBank deposit SAMN05421824.

**Emended description of** ***Ichthyenterobacterium magnum* Shakeela et al. 2015**

The description is as before (Shakeela et al., 2015a) with the following modification. The G+C content of the type-strain genome is 31.4%, its approximate size 3.01 Mbp, its IMG deposit 2728369274.

**Emended description of** ***Kordia periserrulae* Choi et al. 2011**

The description is as before (Choi et al., 2011) with the following modification. The G+C content of the type-strain genome is 36.2%, its approximate size 4.73 Mbp, its IMG deposit 2734482288.

**Emended description of** ***Lacinutrix algicola* Nedashkovskaya et al. 2008**

The description is as before (Nedashkovskaya et al., 2008b) with the following modification. The G+C content of the type-strain genome is 31.4%, its approximate size 3.66 Mbp, its GenBank deposit SAMN04002512.

**Emended description of** ***Lacinutrix himadriensis* Srinivas et al. 2013**

The description is as before (Srinivas et al., 2013) with the following modification. The G+C content of the type-strain genome is 32.6%, its approximate size 4.17 Mbp, its GenBank deposit SAMN04002506.

**Emended description of** ***Lacinutrix mariniflava* Nedashkovskaya et al. 2008**

The description is as before (Nedashkovskaya et al., 2008b) with the following modification. The G+C content of the type-strain genome is 31.8%, its approximate size 3.97 Mbp, its GenBank deposit SAMN04002510.

**Emended description of** ***Lacinutrix venerupis* Lasa et al. 2016**

The description is as before (Lasa et al., 2015) with the following modification. The G+C content of the type-strain genome is 30.4%, its approximate size 3.12 Mbp, its IMG deposit 2734482143.

**Emended description of** ***Leeuwenhoekiella nanhaiensis* Liu et al. 2016**

The description is as before (Liu et al., 2016) with the following modification. The G+C content of the type-strain genome is 42.1%, its approximate size 4.37 Mbp, its GenBank deposit SAMN07515617.

**Emended description of** ***Leeuwenhoekiella palythoae* Nedashkovskaya et al. 2009**

The description is as before (Nedashkovskaya et al., 2009b) with the following modification. The G+C content of the type-strain genome is 39.9%, its approximate size 4.02 Mbp, its GenBank deposit SAMN04487999.

**Emended description of** ***Lentimicrobium saccharophilum* Sun et al. 2016**

The description is as before (Sun et al., 2016) with the following modification. The G+C content of the type-strain genome is 46.7%, its approximate size 4.51 Mbp, its GenBank deposit SAMD00034990.

**Emended description of** ***Lentisphaera araneosa* Cho et al. 2004**

The description is as before (Cho et al., 2004) with the following restriction. The G+C content of the type-strain genome is 41.0%, its GenBank deposit SAMN02436148.

**Emended description of** ***Leptospirillum ferriphilum* Coram and Rawlings 2002**

The description is as before (Coram and Rawlings, 2002) with the following restriction. The G+C content of the type-strain genome is 54.1%, its approximate size 2.41 Mbp, its GenBank deposit SAMN02894305.

**Emended description of** ***Lewinella marina* Khan et al. 2007**

The description is as before (Khan et al., 2007a) with the following modification. The G+C content of the type-strain genome is 62.0%, its approximate size 4.52 Mbp, its GenBank deposit SAMN07775809.

**Emended description of** ***Lishizhenia tianjinensis* Chen et al. 2009**

The description is as before (Chen L.-P. et al., 2009) with the following modification. The G+C content of the type-strain genome is 37.4%, its approximate size 3.57 Mbp, its GenBank deposit SAMN05216474.

**Emended description of** ***Longibacter salinarum* Xia et al. 2016**

The description is as before (Xia J. et al., 2016) with the following modification. The G+C content of the type-strain genome is 59.3%, its approximate size 4.41 Mbp, its GenBank deposit SAMN07768564.

**Emended description of** ***Longimonas halophila* Xia et al. 2015**

The description is as before (Xia et al., 2015) with the following modification. The G+C content of the type-strain genome is 60.5%, its approximate size 3.73 Mbp, its GenBank deposit SAMN07768561.

**Emended description of** ***Lutibacter agarilyticus* Park et al. 2013**

The description is as before (Park et al., 2013b) with the following restriction. The G+C content of the type-strain genome is 31.2%, its approximate size 4.10 Mbp, its IMG deposit 2724679779.

**Emended description of** ***Lutibacter flavus* Choi et al. 2013**

The description is as before (Choi A. et al., 2013) with the following modification. The G+C content of the type-strain genome is 30.0%, its approximate size 3.90 Mbp, its GenBank deposit SAMN04488111.

**Emended description of** ***Lutibacter maritimus* Park et al. 2010**

The description is as before (Park et al., 2010) with the following modification. The G+C content of the type-strain genome is 29.5%, its approximate size 3.48 Mbp, its GenBank deposit SAMN04488006.

**Emended description of** ***Mangrovibacterium diazotrophicum* Huang et al. 2014**

The description is as before (Huang et al., 2014) with the following modification. The G+C content of the type-strain genome is 44.6%, its approximate size 5.80 Mbp, its GenBank deposit SAMN05444343.

**Emended description of** ***Maribacter arcticus* Cho et al. 2008 emend. Weerawongwiwat et al. 2013**

The description is as before (Weerawongwiwat et al., 2013) with the following modification. The G+C content of the type-strain genome is 35.0%, its approximate size 4.20 Mbp, its GenBank deposit SAMN05660866.

**Emended description of** ***Maribacter orientalis* Nedashkovskaya et al. 2004**

The description is as before (Nedashkovskaya et al., 2004a) with the following modification. The G+C content of the type-strain genome is 35.1%, its approximate size 4.16 Mbp, its GenBank deposit SAMN04488008.

**Emended description of** ***Maribacter stanieri* Nedashkovskaya et al. 2010**

The description is as before (Nedashkovskaya et al., 2010a) with the following modification. The G+C content of the type-strain genome is 34.4%, its approximate size 4.46 Mbp, its GenBank deposit SAMN04488010.

**Emended description of** ***Maribacter ulvicola* Nedashkovskaya et al. 2004**

The description is as before (Nedashkovskaya et al., 2004a) with the following restriction. The G+C content of the type-strain genome is 35.2%, its approximate size 4.51 Mbp, its GenBank deposit SAMN05421797.

**Emended description of** ***Mariniblastus fucicola* Lage et al. 2017**

The description is as before (Lage et al., 2017) with the following modification. The G+C content of the type-strain genome is 53.4%, its approximate size 6.53 Mbp, its GenBank deposit SAMN03253105.

**Emended description of** ***Marinifilum flexuosum* Ruvira et al. 2013**

The description is as before (Ruvira et al., 2013) with the following restriction. The G+C content of the type-strain genome is 35.9%, its IMG deposit 2728369260.

**Emended description of** ***Marinifilum fragile* Na et al. 2009**

The description is as before (Na et al., 2009) with the following modification. The G+C content of the type-strain genome is 35.7%, its approximate size 4.72 Mbp, its GenBank deposit SAMD00000450.

**Emended description of** ***Marinoscillum furvescens* (ex Lewin 1969) Seo et al. 2009**

The description is as before (Seo et al., 2009) with the following modification. The G+C content of the type-strain genome is 45.4%, its approximate size 5.96 Mbp, its IMG deposit 2754412942.

**Emended description of** ***Marivirga sericea* (ex Lewin 1969) Nedashkovskaya et al. 2010**

The description is as before (Nedashkovskaya et al., 2010c) with the following modification. The G+C content of the type-strain genome is 36.0%, its approximate size 4.74 Mbp, its GenBank deposit SAMN05661096.

**Emended description of** ***Meiothermus ruber* (Loginova et al. 1984) Nobre et al. 1996**

The description is as before (Nobre et al., 1996) with the following restriction. The G+C content of the type-strain genome is 63.4%, its approximate size 3.10 Mbp, its GenBank deposit SAMN00002601.

**Emended description of** ***Meiothermus rufus* Albuquerque et al. 2010**

The description is as before (Albuquerque et al., 2009) with the following modification. The G+C content of the type-strain genome is 63.2%, its approximate size 2.75 Mbp, its GenBank deposit SAMN02441497.

**Emended description of** ***Meiothermus taiwanensis* Chen et al. 2002**

The description is as before (Chen et al., 2002) with the following modification. The G+C content of the type-strain genome is 63.5%, its approximate size 3.02 Mbp, its GenBank deposit SAMN02440751.

**Emended description of** ***Mesonia algae* Nedashkovskaya et al. 2003**

The description is as before (Nedashkovskaya et al., 2003) with the following restriction. The G+C content of the type-strain genome is 33.1%, its approximate size 3.09 Mbp, its GenBank deposit SAMN05660441.

**Emended description of** ***Mesonia phycicola* Kang and Lee 2010**

The description is as before (Kang and Lee, 2010) with the following modification. The G+C content of the type-strain genome is 31.4%, its approximate size 3.28 Mbp, its GenBank deposit SAMN04488096.

**Emended description of** ***Moheibacter sediminis* Zhang et al. 2014**

The description is as before (Zhang R.-G. et al., 2014) with the following modification. The G+C content of the type-strain genome is 35.8%, its approximate size 3.23 Mbp, its GenBank deposit SAMN06296427.

**Emended description of** ***Mongoliibacter ruber* Wang et al. 2016**

The description is as before (Wang et al., 2016) with the following modification. The G+C content of the type-strain genome is 39.3%, its approximate size 5.00 Mbp, its IMG deposit 2731639260.

**Emended description of** ***Mooreia alkaloidigena* Choi et al. 2013**

The description is as before (Choi E. J. et al., 2013) with the following modification. The G+C content of the type-strain genome is 56.6%, its approximate size 7.11 Mbp, its GenBank deposit SAMN05421823.

**Emended description of** ***Mucilaginibacter auburnensis* Kämpfer et al. 2014**

The description is as before (Kämpfer et al., 2014a) with the following addition. The G+C content of the type-strain genome is 41.9%, its approximate size 4.04 Mbp, its IMG deposit 2731639120.

**Emended description of** ***Mucilaginibacter frigoritolerans* Männistö et al. 2010**

The description is as before (Männistö et al., 2010) with the following modification. The G+C content of the type-strain genome is 39.2%, its approximate size 5.65 Mbp, its GenBank deposit SAMN02982930.

**Emended description of** ***Mucilaginibacter gossypii* Madhaiyan et al. 2010**

The description is as before (Madhaiyan et al., 2010b) with the following modification. The G+C content of the type-strain genome is 42.8%, its approximate size 7.11 Mbp, its GenBank deposit SAMN05192573.

**Emended description of** ***Mucilaginibacter gossypiicola* Madhaiyan et al. 2010**

The description is as before (Madhaiyan et al., 2010b) with the following modification. The G+C content of the type-strain genome is 42.5%, its approximate size 8.22 Mbp, its GenBank deposit SAMN05192574.

**Emended description of** ***Mucilaginibacter lappiensis* Männistö et al. 2010**

The description is as before (Männistö et al., 2010) with the following restriction. The G+C content of the type-strain genome is 41.7%, its approximate size 6.79 Mbp, its GenBank deposit SAMN05421821.

**Emended description of** ***Mucilaginibacter mallensis* Männistö et al. 2010**

The description is as before (Männistö et al., 2010) with the following modification. The G+C content of the type-strain genome is 41.3%, its approximate size 6.01 Mbp, its GenBank deposit SAMN05216490.

**Emended description of** ***Mucilaginibacter pedocola* Tang et al. 2016**

The description is as before (Tang J. et al., 2016) with the following modification. The G+C content of the type-strain genome is 46.1%, its approximate size 7.04 Mbp, its GenBank deposit SAMN05436731.

**Emended description of** ***Mucilaginibacter polytrichastri* Chen et al. 2014**

The description is as before (Chen X. Y. et al., 2014) with the following modification. The G+C content of the type-strain genome is 42.0%, its approximate size 5.81 Mbp, its GenBank deposit SAMN04487890.

**Emended description of** ***Mucilaginibacter yixingensis* Jing et al. 2016**

The description is as before (Jing et al., 2016) with the following modification. The G+C content of the type-strain genome is 45.8%, its approximate size 5.32 Mbp, its IMG deposit 2734482263.

**Emended description of** ***Muricauda pacifica* Zhang et al. 2015**

The description is as before (Zhang et al., 2015a) with the following modification. The G+C content of the type-strain genome is 39.7%, its approximate size 4.38 Mbp, its IMG deposit 2731639122.

**Emended description of** ***Myroides indicus* Ram et al. 2015**

The description is as before (Ram et al., 2015) with the following modification. The G+C content of the type-strain genome is 34.5%, its approximate size 3.06 Mbp, its IMG deposit 2739367659.

**Emended description of** ***Myroides odoratimimus* Vancanneyt et al. 1996**

The description is as before (Vancanneyt et al., 1996) with the following restriction. The G+C content of the type-strain genome is 33.9%, its approximate size 4.16 Mbp, its GenBank deposit SAMN02983007.

**Emended description of** ***Myroides phaeus* Yan et al. 2012**

The description is as before (Yan et al., 2012) with the following modification. The G+C content of the type-strain genome is 33.2%, its approximate size 2.97 Mbp, its GenBank deposit SAMN05421818.

**Emended description of** ***Niabella drilacis* Glaeser et al. 2013**

The description is as before (Glaeser et al., 2013b) with the following addition. The G+C content of the type-strain genome is 47.8%, its approximate size 6.09 Mbp, its GenBank deposit SAMN04487894.

**Emended description of** ***Nitrospira moscoviensis* Ehrich et al. 2001**

The description is as before (Ehrich et al., 1995) with the following modification. The G+C content of the type-strain genome is 62.0%, its approximate size 4.59 Mbp, its GenBank deposit SAMN03702441.

**Emended description of** ***Nonlabens agnitus* Yi and Chun 2012 emend. Kwon et al. 2014**

The description is as before (Kwon et al., 2014) with the following modification. The G+C content of the type-strain genome is 41.0%, its approximate size 3.24 Mbp, its GenBank deposit SAMN06074326.

**Emended description of** ***Nonlabens arenilitoris* Park et al. 2013**

The description is as before (Park et al., 2013e) with the following modification. The G+C content of the type-strain genome is 35.1%, its approximate size 3.32 Mbp, its GenBank deposit SAMN06075340.

**Emended description of** ***Nonlabens dokdonensis* (Yoon et al. 2006) Yi and Chun 2012**

The description is as before (Yi and Chun, 2012) with the following modification. The G+C content of the type-strain genome is 35.2%, its approximate size 3.84 Mbp, its GenBank deposit SAMN05660418.

**Emended description of** ***Nonlabens sediminis* (Khan et al. 2006) Yi and Chun 2012**

The description is as before (Yi and Chun, 2012) with the following modification. The G+C content of the type-strain genome is 35.5%, its approximate size 2.84 Mbp, its GenBank deposit SAMN06075339.

**Emended description of** ***Nonlabens tegetincola* Lau et al. 2005 emend. Lau et al. 2006**

The description is as before (Lau et al., 2006b) with the following modification. The G+C content of the type-strain genome is 35.7%, its approximate size 3.03 Mbp, its GenBank deposit SAMN06075350.

**Emended description of** ***Oceanihabitans sediminis* Zhang et al. 2016**

The description is as before (Zhang et al., 2016a) with the following modification. The G+C content of the type-strain genome is 33.1%, its approximate size 2.89 Mbp, its IMG deposit 2770939504.

**Emended description of** ***Oceanithermus profundus* Miroshnichenko et al. 2003**

The description is as before (Miroshnichenko et al., 2003) with the following modification. The G+C content of the type-strain genome is 69.8%, its approximate size 2.44 Mbp, its GenBank deposit SAMN00138957.

**Emended description of** ***Olleya aquimaris* Lee et al. 2010**

The description is as before (Lee S.-Y. et al., 2010) with the following modification. The G+C content of the type-strain genome is 32.2%, its approximate size 2.96 Mbp, its GenBank deposit SAMN05660851.

**Emended description of** ***Olleya marilimosa* Mancuso Nichols et al. 2005**

The description is as before (Nichols et al., 2005) with the following modification. The G+C content of the type-strain genome is 31.8%, its approximate size 3.34 Mbp, its GenBank deposit SAMN02584980.

**Emended description of** ***Olleya namhaensis* Lee et al. 2013**

The description is as before (Lee M.-H. et al., 2013) with the following modification. The G+C content of the type-strain genome is 32.7%, its approximate size 3.52 Mbp, its GenBank deposit SAMN05443431.

**Emended description of** ***Parabacteroides chartae* Tan et al. 2012**

The description is as before (Tan et al., 2012) with the following modification. The G+C content of the type-strain genome is 41.7%, its approximate size 3.91 Mbp, its GenBank deposit SAMN05660349.

**Emended description of** ***Parabacteroides chinchillae* Kitahara et al. 2013**

The description is as before (Kitahara et al., 2013) with the following modification. The G+C content of the type-strain genome is 40.4%, its approximate size 3.57 Mbp, its GenBank deposit SAMN05444001.

**Emended description of** ***Parachlamydia acanthamoebae* Everett et al. 1999**

The description is as before (Everett et al., 1999) with the following addition. The G+C content of the type-strain genome is 38.9%, its approximate size 3.00 Mbp, its GenBank deposit SAMD00000358.

**Emended description of** ***Parafilimonas terrae* Kim et al. 2014**

The description is as before (Kim S.-J. et al., 2014) with the following modification. The G+C content of the type-strain genome is 39.6%, its approximate size 4.99 Mbp, its GenBank deposit SAMN05444277.

**Emended description of** ***Parapedobacter composti* Kim et al. 2010**

The description is as before (Kim S.-J. et al., 2010) with the following modification. The G+C content of the type-strain genome is 50.0%, its approximate size 4.62 Mbp, its GenBank deposit SAMN05421747.

**Emended description of** ***Parapedobacter indicus* Kumar et al. 2015**

The description is as before (Kumar et al., 2015) with the following modification. The G+C content of the type-strain genome is 48.0%, its approximate size 6.16 Mbp, its IMG deposit 2728369513.

**Emended description of** ***Parapedobacter koreensis* Kim et al. 2007**

The description is as before (Kim et al., 2007) with the following modification. The G+C content of the type-strain genome is 48.2%, its approximate size 5.55 Mbp, its GenBank deposit SAMN05421740.

**Emended description of** ***Parapedobacter luteus* Kim et al. 2010**

The description is as before (Kim S.-J. et al., 2010) with the following modification. The G+C content of the type-strain genome is 49.3%, its approximate size 4.83 Mbp, its GenBank deposit SAMN05660226.

**Emended description of** ***Pedobacter africanus* Steyn et al. 1998**

The description is as before (Steyn et al., 1998) with the following restriction. The G+C content of the type-strain genome is 43.4%, its approximate size 5.72 Mbp, its GenBank deposit SAMN04488524.

**Emended description of** ***Pedobacter agri* Roh et al. 2008**

The description is as before (Roh et al., 2008) with the following modification. The G+C content of the type-strain genome is 38.0%, its approximate size 5.14 Mbp, its GenBank deposit SAMN02470139.

**Emended description of** ***Pedobacter alluvionis* Gordon et al. 2009**

The description is as before (Gordon et al., 2009) with the following modification. The G+C content of the type-strain genome is 38.4%, its approximate size 6.04 Mbp, its GenBank deposit SAMN05421794.

**Emended description of** ***Pedobacter arcticus* Zhou et al. 2012**

The description is as before (Zhou et al., 2012) with the following modification. The G+C content of the type-strain genome is 36.9%, its approximate size 4.23 Mbp, its GenBank deposit SAMN02469613.

**Emended description of** ***Pedobacter borealis* Gordon et al. 2009**

The description is as before (Gordon et al., 2009) with the following modification. The G+C content of the type-strain genome is 38.4%, its approximate size 5.55 Mbp, its GenBank deposit SAMN02597035.

**Emended description of** ***Pedobacter caeni* Vanparys et al. 2005**

The description is as before (Vanparys et al., 2005) with the following modification. The G+C content of the type-strain genome is 41.4%, its approximate size 7.83 Mbp, its GenBank deposit SAMN04488522.

**Emended description of** ***Pedobacter cryoconitis* Margesin et al. 2003**

The description is as before (Margesin et al., 2003) with the following modification. The G+C content of the type-strain genome is 39.4%, its approximate size 6.08 Mbp, its GenBank deposit SAMN05660812.

**Emended description of** ***Pedobacter duraquae* Muurholm et al. 2007**

The description is as before (Muurholm et al., 2007) with the following addition. The G+C content of the type-strain genome is 41.3%, its approximate size 5.29 Mbp, its IMG deposit 2728369744.

**Emended description of** ***Pedobacter hartonius* Muurholm et al. 2007**

The description is as before (Muurholm et al., 2007) with the following addition. The G+C content of the type-strain genome is 43.0%, its approximate size 5.19 Mbp, its GenBank deposit SAMN05443550.

**Emended description of** ***Pedobacter insulae* Yoon et al. 2007**

The description is as before (Yoon et al., 2007) with the following modification. The G+C content of the type-strain genome is 38.2%, its approximate size 4.39 Mbp, its GenBank deposit SAMN04489864.

**Emended description of** ***Pedobacter lusitanus* Covas et al. 2017**

The description is as before (Covas et al., 2017) with the following modification. The G+C content of the type-strain genome is 39.0%, its approximate size 5.99 Mbp, its GenBank deposit SAMN03291000.

**Emended description of** ***Pedobacter metabolipauper* Muurholm et al. 2007**

The description is as before (Muurholm et al., 2007) with the following addition. The G+C content of the type-strain genome is 40.6%, its approximate size 5.25 Mbp, its GenBank deposit SAMN04489865.

**Emended description of** ***Pedobacter nutrimenti* Derichs et al. 2014**

The description is as before (Derichs et al., 2014) with the following addition. The G+C content of the type-strain genome is 41.6%, its approximate size 5.72 Mbp, its GenBank deposit SAMN06297424.

**Emended description of** ***Pedobacter nyackensis* Gordon et al. 2009**

The description is as before (Gordon et al., 2009) with the following modification. The G+C content of the type-strain genome is 39.5%, its approximate size 6.08 Mbp, its GenBank deposit SAMN04488101.

**Emended description of** ***Pedobacter psychrophilus* Švec et al. 2017**

The description is as before (Švec et al., 2017) with the following modification. The G+C content of the type-strain genome is 32.5%, its GenBank deposit SAMN04622366.

**Emended description of** ***Pedobacter rhizosphaerae* Kwon et al. 2011**

The description is as before (Kwon et al., 2011) with the following modification. The G+C content of the type-strain genome is 39.3%, its approximate size 5.78 Mbp, its GenBank deposit SAMN04488023.

**Emended description of** ***Pedobacter soli* Kwon et al. 2011**

The description is as before (Kwon et al., 2011) with the following modification. The G+C content of the type-strain genome is 40.5%, its approximate size 6.00 Mbp, its GenBank deposit SAMN04488024.

**Emended description of** ***Pedobacter steynii* Muurholm et al. 2007**

The description is as before (Muurholm et al., 2007) with the following addition. The G+C content of the type-strain genome is 41.8%, its approximate size 8.06 Mbp, its GenBank deposit SAMN05421820.

**Emended description of** ***Pedobacter suwonensis* Kwon et al. 2007**

The description is as before (Kwon et al., 2007) with the following modification. The G+C content of the type-strain genome is 39.5%, its approximate size 5.80 Mbp, its GenBank deposit SAMN04488511.

**Emended description of** ***Pedobacter westerhofensis* Muurholm et al. 2007**

The description is as before (Muurholm et al., 2007) with the following addition. The G+C content of the type-strain genome is 43.2%, its approximate size 6.35 Mbp, its IMG deposit 2724679782.

**Emended description of** ***Pedobacter xixiisoli* Zeng et al. 2014**

The description is as before (Zeng et al., 2014) with the following modification. The G+C content of the type-strain genome is 37.7%, its approximate size 4.97 Mbp, its GenBank deposit SAMN06297358.

**Emended description of** ***Phocaeicola abscessus* Al Masalma et al. 2009**

The description is as before (Al Masalma et al., 2009) with the following addition. The G+C content of the type-strain genome is 47.3%, its approximate size 2.53 Mbp, its GenBank deposit SAMEA2272382.

**Emended description of** ***Polaribacter butkevichii* Nedashkovskaya et al. 2006 emend. Kim et al. 2013**

The description is as before (Kim B.-C. et al., 2013) with the following modification. The G+C content of the type-strain genome is 30.4%, its approximate size 3.98 Mbp, its GenBank deposit SAMN06133457.

**Emended description of** ***Polaribacter filamentus* Gosink et al. 1998**

The description is as before (Gosink et al., 1998) with the following restriction. The G+C content of the type-strain genome is 31.4%, its approximate size 4.28 Mbp, its GenBank deposit SAMN06074323.

**Emended description of** ***Polaribacter glomeratus* (McGuire et al. 1988) Gosink et al. 1998**

The description is as before (Gosink et al., 1998) with the following modification. The G+C content of the type-strain genome is 30.6%, its approximate size 4.00 Mbp, its GenBank deposit SAMN06133459.

**Emended description of** ***Polaribacter reichenbachii* Nedashkovskaya et al. 2013**

The description is as before (Nedashkovskaya et al., 2013) with the following modification. The G+C content of the type-strain genome is 29.5%, its approximate size 4.10 Mbp, its GenBank deposit SAMN04390149.

**Emended description of** ***Pontibacter indicus* Singh et al. 2014**

The description is as before (Singh et al., 2014) with the following modification. The G+C content of the type-strain genome is 51.9%, its approximate size 4.57 Mbp, its GenBank deposit SAMN05444128.

**Emended description of** ***Pontibacter ramchanderi* Singh et al. 2013**

The description is as before (Singh et al., 2013) with the following modification. The G+C content of the type-strain genome is 51.3%, its approximate size 4.43 Mbp, its GenBank deposit SAMN05444683.

**Emended description of** ***Pontibacter ummariensis* Mahato et al. 2016**

The description is as before (Mahato et al., 2016) with the following modification. The G+C content of the type-strain genome is 50.3%, its approximate size 5.97 Mbp, its IMG deposit 2728369279.

**Emended description of** ***Pontibacter virosus* Kohli et al. 2016**

The description is as before (Kohli et al., 2016) with the following modification. The G+C content of the type-strain genome is 50.1%, its approximate size 4.88 Mbp, its IMG deposit 2756170242.

**Emended description of** ***Porphyromonas cangingivalis* Collins et al. 1994**

The description is as before (Collins et al., 1994b) with the following modification. The G+C content of the type-strain genome is 47.6%, its approximate size 2.37 Mbp, its GenBank deposit SAMN02745205.

**Emended description of** ***Porphyromonas cansulci* Collins et al. 1994**

The description is as before (Collins et al., 1994b) with the following restriction. The G+C content of the type-strain genome is 45.4%, its approximate size 2.11 Mbp, its GenBank deposit SAMD00036672.

**Emended description of** ***Porphyromonas circumdentaria* Love et al. 1992**

The description is as before (Love et al., 1992) with the following restriction. The G+C content of the type-strain genome is 43.0%, its approximate size 2.03 Mbp, its GenBank deposit SAMN02745171.

**Emended description of** ***Porphyromonas crevioricanis* Hirasawa and Takada 1994 emend. Sakamoto and Ohkuma 2013**

The description is as before (Sakamoto and Ohkuma, 2013b) with the following restriction. The G+C content of the type-strain genome is 45.3%, its approximate size 2.04 Mbp, its GenBank deposit SAMN02745203.

**Emended description of** ***Porphyromonas gingivicanis* Hirasawa and Takada 1994**

The description is as before (Hirasawa and Takada, 1994) with the following modification. The G+C content of the type-strain genome is 42.7%, its approximate size 2.05 Mbp, its GenBank deposit SAMD00009315.

**Emended description of** ***Prevotella colorans* Buhl et al. 2016**

The description is as before (Buhl et al., 2016) with the following modification. The G+C content of the type-strain genome is 46.2%, its approximate size 2.94 Mbp, its IMG deposit 2756170230.

**Emended description of** ***Prevotella disiens* (Holdeman and Johnson 1977) Shah and Collins 1990**

The description is as before (Shah and Collins, 1990) with the following restriction. The G+C content of the type-strain genome is 39.6%, its approximate size 2.68 Mbp, its GenBank deposit SAMN02436748.

**Emended description of** ***Prevotella falsenii* Sakamoto et al. 2009**

The description is as before (Sakamoto et al., 2009a) with the following modification. The G+C content of the type-strain genome is 44.0%, its approximate size 2.80 Mbp, its GenBank deposit SAMD00004645.

**Emended description of** ***Prevotella oryzae* (Ueki et al. 2006) Sakamoto and Ohkuma 2012**

The description is as before (Sakamoto and Ohkuma, 2012b) with the following modification. The G+C content of the type-strain genome is 37.0%, its approximate size 3.29 Mbp, its GenBank deposit SAMN02849400.

**Emended description of** ***Prevotella oulorum* (Shah et al. 1985) Shah and Collins 1990**

The description is as before (Shah and Collins, 1990) with the following modification. The G+C content of the type-strain genome is 46.8%, its approximate size 2.83 Mbp, its GenBank deposit SAMN02745202.

**Emended description of** ***Prevotella ruminicola* (Bryant et al. 1958) Shah and Collins 1990 emend. Avguštin et al. 1997**

The description is as before (Avguštin et al., 1997) with the following restriction. The G+C content of the type-strain genome is 47.8%, its approximate size 3.55 Mbp, its GenBank deposit SAMN02745192.

**Emended description of** ***Prosthecobacter fusiformis* (ex Staley et al. 1976) Staley et al. 1980**

The description is as before (Staley et al., 1980) with the following addition. The G+C content of the type-strain genome is 56.7%, its approximate size 5.83 Mbp, its GenBank deposit SAMN02745162.

**Emended description of Proteiniphilum saccharofermentans Hahnke et al. 2016**

The description is as before (Hahnke S. et al., 2016) with the following modification. The G+C content of the type-strain genome is 43.6%, its approximate size 4.41 Mbp, its GenBank deposit SAMEA4373296.

**Emended description of** ***Pseudarcicella hirudinis* Kämpfer et al. 2012**

The description is as before (Kämpfer et al., 2012a) with the following modification. The G+C content of the type-strain genome is 38.4%, its approximate size 6.16 Mbp, its GenBank deposit SAMN04515674.

**Emended description of** ***Pseudozobellia thermophila* Nedashkovskaya et al. 2009**

The description is as before (Nedashkovskaya et al., 2009a) with the following modification. The G+C content of the type-strain genome is 47.1%, its approximate size 5.03 Mbp, its GenBank deposit SAMN04488513.

**Emended description of** ***Psychroflexus sediminis* Chen et al. 2009**

The description is as before (Chen Y.-G. et al., 2009) with the following modification. The G+C content of the type-strain genome is 38.5%, its approximate size 2.97 Mbp, its GenBank deposit SAMN04488027.

**Emended description of** ***Psychroserpens damuponensis* Lee et al. 2013**

The description is as before (Lee D.-H. et al., 2013) with the following modification. The G+C content of the type-strain genome is 32.5%, its approximate size 3.95 Mbp, its GenBank deposit SAMN02929384.

**Emended description of** ***Psychroserpens mesophilus* Kwon et al. 2006**

The description is as before (Kwon et al., 2006) with the following modification. The G+C content of the type-strain genome is 33.4%, its approximate size 3.68 Mbp, its GenBank deposit SAMN03160575.

**Emended description of** ***Pustulibacterium marinum* Wang et al. 2013**

The description is as before (Wang G. et al., 2013) with the following modification. The G+C content of the type-strain genome is 35.4%, its approximate size 4.21 Mbp, its GenBank deposit SAMN05216480.

**Emended description of** ***Reichenbachiella agariperforans* (Nedashkovskaya et al. 2003) Nedashkovskaya et al. 2005 emend. Cha et al. 2011**

The description is as before (Cha et al., 2011) with the following modification. The G+C content of the type-strain genome is 43.4%, its approximate size 5.03 Mbp, its GenBank deposit SAMN04488028.

**Emended description of** ***Reichenbachiella faecimaris* Cha et al. 2011**

The description is as before (Cha et al., 2011) with the following restriction. The G+C content of the type-strain genome is 39.8%, its approximate size 4.71 Mbp, its GenBank deposit SAMN04488029.

**Emended description of** ***Rhodopirellula lusitana* Bondoso et al. 2014**

The description is as before (Bondoso et al., 2014) with the following restriction. The G+C content of the type-strain genome is 55.5%, its approximate size 7.78 Mbp, its IMG deposit 2724679732.

**Emended description of** ***Rhodothermus profundi* Marteinsson et al. 2010**

The description is as before (Marteinsson et al., 2010) with the following modification. The G+C content of the type-strain genome is 59.1%, its approximate size 3.13 Mbp, its GenBank deposit SAMN04488087.

**Emended description of** ***Robiginitalea myxolifaciens* Manh et al. 2008**

The description is as before (Manh et al., 2008) with the following modification. The G+C content of the type-strain genome is 48.9%, its approximate size 3.20 Mbp, its GenBank deposit SAMN04490243.

**Emended description of** ***Roseimaritima ulvae* Bondoso et al. 2016**

The description is as before (Bondoso et al., 2015) with the following restriction. The G+C content of the type-strain genome is 59.1%, its approximate size 8.12 Mbp, its GenBank deposit SAMN03253104.

**Emended description of** ***Roseivirga seohaensis* (Yoon et al. 2005) Lau et al. 2006**

The description is as before (Lau et al., 2006a) with the following modification. The G+C content of the type-strain genome is 39.3%, its approximate size 4.16 Mbp, its GenBank deposit SAMN04423148.

**Emended description of** ***Rubripirellula obstinata* Bondoso et al. 2016**

The description is as before (Bondoso et al., 2015) with the following modification. The G+C content of the type-strain genome is 54.1%, its approximate size 6.58 Mbp, its GenBank deposit SAMN03252601.

**Emended description of** ***Rubrivirga marina* Park et al. 2013**

The description is as before (Park et al., 2013f) with the following modification. The G+C content of the type-strain genome is 72.5%, its approximate size 4.98 Mbp, its GenBank deposit SAMN06091685.

**Emended description of** ***Saccharicrinis carchari* Liu et al. 2014**

The description is as before (Liu et al., 2014b) with the following modification. The G+C content of the type-strain genome is 41.1%, its approximate size 4.64 Mbp, its IMG deposit 2724679709.

**Emended description of** ***Salegentibacter agarivorans* Nedashkovskaya et al. 2006**

The description is as before (Nedashkovskaya et al., 2006a) with the following modification. The G+C content of the type-strain genome is 36.8%, its approximate size 4.30 Mbp, its GenBank deposit SAMN04488033.

**Emended description of** ***Salegentibacter salegens* (Dobson et al. 1993) McCammon and Bowman 2000**

The description is as before (McCammon and Bowman, 2000) with the following modification. The G+C content of the type-strain genome is 37.1%, its approximate size 3.87 Mbp, its GenBank deposit SAMN05661042.

**Emended description of** ***Salinimicrobium sediminis* Subhash et al. 2014**

The description is as before (Subhash et al., 2014) with the following modification. The G+C content of the type-strain genome is 41.7%, its approximate size 3.48 Mbp, its GenBank deposit SAMN06296241.

**Emended description of** ***Sediminibacterium ginsengisoli* Kim et al. 2013**

The description is as before (Kim Y.-J. et al., 2013) with the following restriction. The G+C content of the type-strain genome is 46.3%, its approximate size 4.08 Mbp, its GenBank deposit SAMN04488132.

**Emended description of** ***Sediminibacterium salmoneum* Qu and Yuan 2008 emend. Kim et al. 2013**

The description is as before (Kim S.-J. et al., 2010) with the following modification. The G+C content of the type-strain genome is 39.3%, its approximate size 3.19 Mbp, its GenBank deposit SAMN02380457.

**Emended description of** ***Sediminitomix flava* Khan et al. 2007**

The description is as before (Khan et al., 2007b) with the following modification. The G+C content of the type-strain genome is 35.6%, its approximate size 6.60 Mbp, its GenBank deposit SAMN05444386.

**Emended description of** ***Simkania negevensis* Everett et al. 1999**

The description is as before (Everett et al., 1999) with the following addition. The G+C content of the type-strain genome is 41.6%, its approximate size 2.63 Mbp, its GenBank deposit SAMEA2272380.

**Emended description of** ***Sinomicrobium oceani* Xu et al. 2013**

The description is as before (Xu et al., 2013) with the following modification. The G+C content of the type-strain genome is 44.8%, its approximate size 5.02 Mbp, its GenBank deposit SAMN02927921.

**Emended description of** ***Sphingobacterium composti* Ten et al. 2007**

The description is as before (Ten et al., 2006) with the following modification. The G+C content of the type-strain genome is 46.8%, its approximate size 4.39 Mbp, its IMG deposit 2700988711.

**Emended description of** ***Sphingobacterium faecium* Takeuchi and Yokota 1993**

The description is as before (Takeuchi and Yokota, 1992) with the following restriction. The G+C content of the type-strain genome is 36.3%, its approximate size 5.30 Mbp, its IMG deposit 2734482254.

**Emended description of** ***Sphingobacterium gobiense* Zhao et al. 2014**

The description is as before (Zhao et al., 2014) with the following modification. The G+C content of the type-strain genome is 42.1%, its approximate size 4.60 Mbp, its GenBank deposit SAMN08612622.

**Emended description of** ***Sphingobacterium mizutaii* Yabuuchi et al. 1983 emend. Wauters et al. 2012**

The description is as before (Wauters et al., 2012) with the following restriction. The G+C content of the type-strain genome is 39.7%, its approximate size 4.63 Mbp, its GenBank deposit SAMN05192578.

**Emended description of** ***Sphingobacterium psychroaquaticum* Albert et al. 2013**

The description is as before (Albert et al., 2013) with the following addition. The G+C content of the type-strain genome is 41.5%, its approximate size 4.50 Mbp, its GenBank deposit SAMN05660862.

**Emended description of** ***Spirosoma aerolatum* Kim et al. 2015**

The description is as before (Kim et al., 2015) with the following modification. The G+C content of the type-strain genome is 48.3%, its approximate size 7.96 Mbp, its GenBank deposit SAMN06554014.

**Emended description of** ***Spirosoma fluviale* Hatayama and Kuno 2015**

The description is as before (Hatayama and Kuno, 2015) with the following modification. The G+C content of the type-strain genome is 50.2%, its approximate size 7.81 Mbp, its IMG deposit 2728369216.

**Emended description of** ***Spirosoma oryzae* Ahn et al. 2014**

The description is as before (Ahn et al., 2014) with the following modification. The G+C content of the type-strain genome is 54.1%, its approximate size 6.57 Mbp, its IMG deposit 2728369482.

**Emended description of** ***Spirosoma rigui* Baik et al. 2007**

The description is as before (Baik et al., 2007b) with the following modification. The G+C content of the type-strain genome is 54.4%, its approximate size 5.83 Mbp, its GenBank deposit SAMN06554015.

**Emended description of** ***Sunxiuqinia elliptica* Qu et al. 2011**

The description is as before (Qu et al., 2011) with the following modification. The G+C content of the type-strain genome is 41.7%, its approximate size 5.09 Mbp, its GenBank deposit SAMN05216283.

**Emended description of** ***Tamlana agarivorans* Yoon et al. 2008**

The description is as before (Yoon et al., 2008) with the following modification. The G+C content of the type-strain genome is 34.8%, its approximate size 3.83 Mbp, its GenBank deposit SAMN04876002.

**Emended description of** ***Tangfeifania diversioriginum* Liu et al. 2014**

The description is as before (Liu et al., 2014a) with the following modification. The G+C content of the type-strain genome is 41.8%, its approximate size 4.78 Mbp, its GenBank deposit SAMN05444280.

**Emended description of** ***Tenacibaculum jejuense* Oh et al. 2012**

The description is as before (Oh et al., 2012) with the following modification. The G+C content of the type-strain genome is 30.3%, its approximate size 4.61 Mbp, its GenBank deposit SAMEA104150437.

**Emended description of** ***Thermodesulfovibrio islandicus* Sonne-Hansen and Ahring 2000**

The description is as before (Sonne-Hansen and Ahring, 1999) with the following modification. The G+C content of the type-strain genome is 34.3%, its approximate size 2.04 Mbp, its GenBank deposit SAMN02440754.

**Emended description of** ***Thermodesulfovibrio yellowstonii* Henry et al. 1994**

The description is as before (Henry et al., 1994) with the following restriction. The G+C content of the type-strain genome is 34.1%, its approximate size 2.00 Mbp, its GenBank deposit SAMN02603929.

**Emended description of** ***Thermoflavifilum aggregans* Anders et al. 2014**

The description is as before (Anders et al., 2014) with the following modification. The G+C content of the type-strain genome is 46.0%, its approximate size 2.84 Mbp, its IMG deposit 2728369217.

**Emended description of** ***Thermoflexibacter ruber* (Lewin 1969) Hahnke et al. 2017**

The description is as before (Hahnke R. L. et al., 2016) with the following modification. The G+C content of the type-strain genome is 37.7%, its approximate size 5.49 Mbp, its GenBank deposit SAMN04488541.

**Emended description of** ***Thermus amyloliquefaciens* Yu et al. 2015**

The description is as before (Yu et al., 2015) with the following modification. The G+C content of the type-strain genome is 67.4%, its approximate size 2.16 Mbp, its GenBank deposit SAMN02745441.

**Emended description of** ***Thermus aquaticus* Brock and Freeze 1969**

The description is as before (Brock and Freeze, 1969) with the following restriction. The G+C content of the type-strain genome is 68.1%, its approximate size 2.22 Mbp, its GenBank deposit SAMN03951125.

**Emended description of** ***Thermus filiformis* Hudson et al. 1987**

The description is as before (Hudson et al., 1987) with the following modification. The G+C content of the type-strain genome is 69.0%, its approximate size 2.39 Mbp, its GenBank deposit SAMN02898028.

**Emended description of** ***Thermus igniterrae* Chung et al. 2000**

The description is as before (Chung et al., 2000) with the following modification. The G+C content of the type-strain genome is 68.8%, its approximate size 2.23 Mbp, its GenBank deposit SAMN02440410.

**Emended description of** ***Thermus oshimai* Williams et al. 1996**

The description is as before (Williams et al., 1996) with the following modification. The G+C content of the type-strain genome is 68.7%, its approximate size 2.26 Mbp, its GenBank deposit SAMN02441370.

**Emended description of** ***Thermus thermophilus* (ex Oshima and Imahori 1974) Manaia et al. 1995**

The description is as before (Manaia et al., 1995) with the following addition. The G+C content of the type-strain genome is 69.5%, its approximate size 2.12 Mbp, its GenBank deposit SAMD00061070.

**Emended description of** ***Ulvibacter litoralis* Nedashkovskaya et al. 2004**

The description is as before (Nedashkovskaya et al., 2004c) with the following restriction. The G+C content of the type-strain genome is 35.8%, its approximate size 3.82 Mbp, its GenBank deposit SAMN05421855.

**Emended description of** ***Verrucomicrobium spinosum* Schlesner 1988**

The description is as before (Schlesner, 1987) with the following modification. The G+C content of the type-strain genome is 60.3%, its approximate size 8.22 Mbp, its GenBank deposit SAMN02436175.

**Emended description of** ***Waddlia chondrophila* Rurangirwa et al. 1999**

The description is as before (Rurangirwa et al., 1999) with the following addition. The G+C content of the type-strain genome is 43.7%, its approximate size 2.13 Mbp, its GenBank deposit SAMN02603402.

**Emended description of** ***Wenyingzhuangia marina* Liu et al. 2014**

The description is as before (Liu Y. et al., 2014) with the following modification. The G+C content of the type-strain genome is 31.2%, its approximate size 3.24 Mbp, its GenBank deposit SAMN05444281.

**Emended description of** ***Winogradskyella jejuensis* Kim and Oh 2015**

The description is as before (Kim and Oh, 2012) with the following modification. The G+C content of the type-strain genome is 34.5%, its approximate size 3.03 Mbp, its GenBank deposit SAMN05444148.

**Emended description of** ***Winogradskyella sediminis* Zhang et al. 2016**

The description is as before (Zhang et al., 2016b) with the following modification. The G+C content of the type-strain genome is 33.6%, its approximate size 3.80 Mbp, its IMG deposit 2739367661.

**Emended description of** ***Winogradskyella thalassocola* Nedashkovskaya et al. 2005 emend. Nedashkovskaya et al. 2012**

The description is as before (Nedashkovskaya et al., 2012) with the following modification. The G+C content of the type-strain genome is 33.3%, its approximate size 4.57 Mbp, its GenBank deposit SAMN04489796.

**Emended description of** ***Xanthomarina gelatinilytica* Vaidya et al. 2015**

The description is as before (Vaidya et al., 2015) with the following modification. The G+C content of the type-strain genome is 35.4%, its approximate size 3.06 Mbp, its GenBank deposit SAMN01940371.

**Emended description of** ***Zhouia amylolytica* Liu et al. 2006**

The description is as before (Liu et al., 2006) with the following modification. The G+C content of the type-strain genome is 36.7%, its approximate size 3.89 Mbp, its GenBank deposit SAMN04487906.

**Emended description of** ***Zunongwangia mangrovi* Rameshkumar et al. 2014**

The description is as before (Rameshkumar et al., 2014) with the following modification. The G+C content of the type-strain genome is 35.9%, its approximate size 4.13 Mbp, its GenBank deposit SAMN04487907.

## Author Contributions

RH and MG selected the strains. BT and SG cultivated the strains. MG generated DNA. TW sequenced the genomes. NK annotated the genomes. JM-K and MG phylogenetically analyzed the data. MG-L and MG collected information from the taxonomic literature. MG-L, MG, and RH interpreted the results and wrote the manuscript. JM-K prepared the figures. All authors read and approved the final manuscript.

### Conflict of Interest Statement

The authors declare that the research was conducted in the absence of any commercial or financial relationships that could be construed as a potential conflict of interest.
