# Supplementary material for: Analysis of 1,000 Type-Strain Genomes Improves Taxonomic Classification of Bacteroidetes
Source: Front Microbiol. 2019 Sep 23;10:2083. doi: 10.3389/fmicb.2019.02083 (PMC6767994; doi:10.3389/fmicb.2019.02083)
Supplement: Supplementary file 1 [file Data_Sheet_1.PDF]

Analysis of 1,000 Type-Strain Genomes Improves Taxonomic Classification of Bacteroidetes

Supplementary File S2

List of Figures

1 Complete phylogenomic GBDP tree of the *Bacteroidetes* genome dataset 13
2 Phylogenomic GBDP tree of selected *Eubacteriales* 14
3 Backbone-constrained 16S rRNA gene ML tree (CCT) of *Bacteroidetes* . 30
4 Unconstrained 16S rRNA gene ML tree (UCT) of *Bacteroidetes* 46
5 Unconstrained 23S rRNA gene ML tree (ULT) of *Bacteroidetes* 54
6 Unconstrained 16S rRNA gene ML tree given GBDP tree sampling (URT) 62
7 Unconstrained 16S rRNA gene ML tree to resolve the placement of *Bacteroides coagulans* (in *Eubacteriales*) 66
8 Unconstrained 16S rRNA gene ML tree to resolve the placement of the three other orphan *Bacteroides* spp. (in *Eubacteriales*). 72
9 Supermatrix-based *Bacteroides* ML tree 73
10 Supermatrix-based *Croceitalea* ML tree 74
11 Supermatrix-based *Fibrobacteres* ML tree 75
12 Supermatrix-based *Flaviramulus* ML tree 76
13 Supermatrix-based *Formosa* ML tree 77
14 Supermatrix-based *Hydrobacter* ML tree 78
15 Supermatrix-based *Labilibacter* ML tree 79
16 Supermatrix-based *Muribaculum* ML tree 80
17 Supermatrix-based *Roseivirga* ML tree 81
18 Supermatrix-based *Lewinella* ML tree with partition bootstrap support . 82
19 Supermatrix-based *Lewinella* ML tree with standard bootstrap support . 83
20 ML bootstrap support for or against monopyly of *Lewinella* 84

Abbreviations

CCT constrained, comprehensive tree inferred with ML and MP using the bipartitions of the GBDP tree with ≥95% support as backbone constraint

GBDP Genome BLAST Distance Phylogeny

ML Maximum Likelihood

UCT unconstrained, comprehensive 16S rRNA gene tree

ULT unconstrained 23S (i.e., large subunit) rRNA gene tree

URT unconstrained 16S rRNA gene tree reduced to genome-sequenced strains

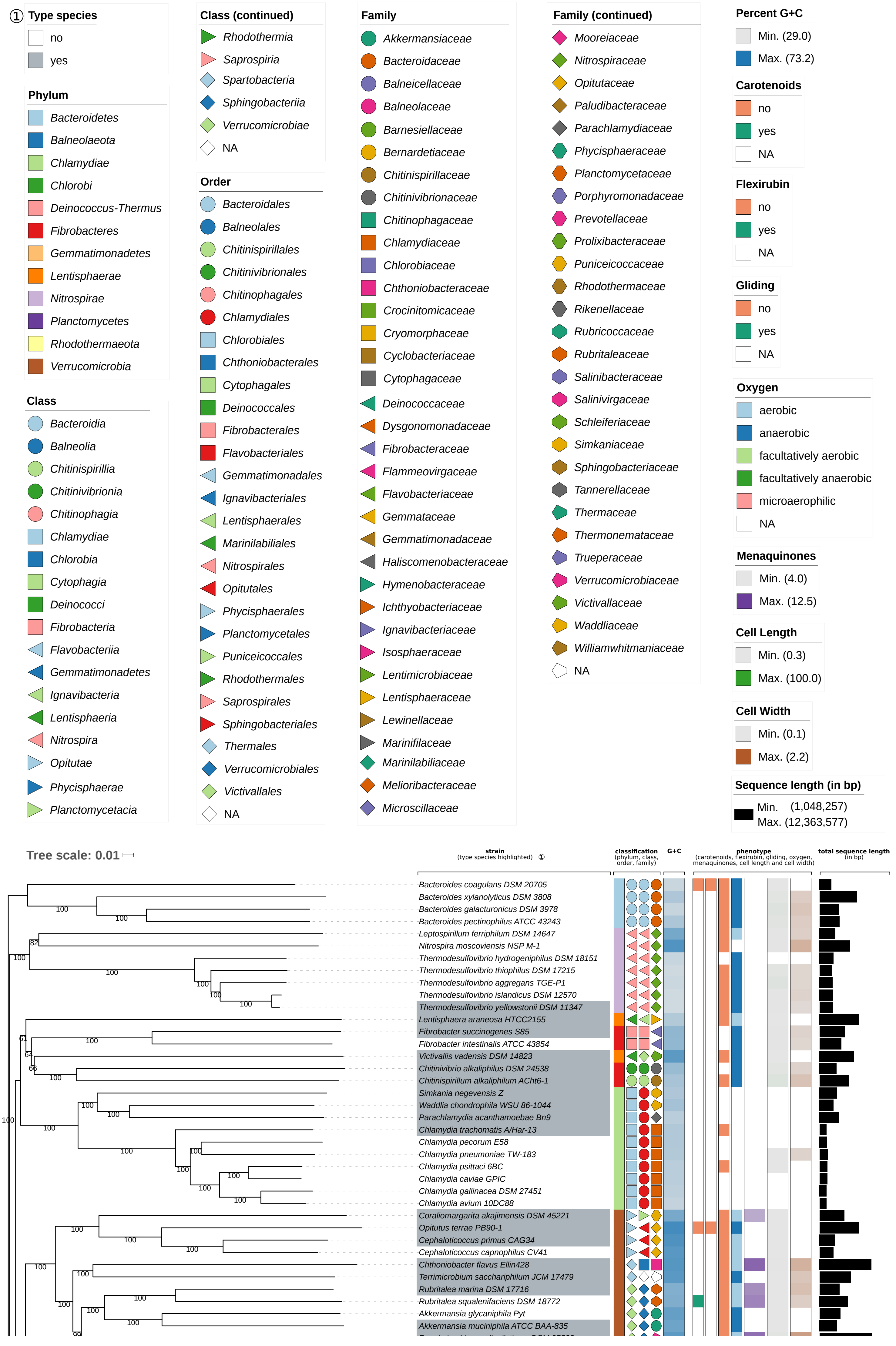

① Type species
no
yes
Phylum
Bacteroidetes
Balneolaeota
Chlamydiae
Chlorobi
Deinococcus-Thermus
Fibrobacteres
Gemmatimonadetes
Lentisphaerae
Nitrospirae
Planctomycetes
Rhodothermaeota
Verrucomicrobia
Class
Bacteroidia
Balneolia
Chitinispirillia
Chitinivibrionia
Chitinophagia
Chlamydiae
Chlorobia
Cytophagia
Deinococci
Fibrobacteria
Flavobacteriia
Gemmatimonadetes
Ignavibacteria
Lentisphaeria
Nitrospira
Opitutae
Phycisphaerae
Planctomycetacia
Class (continued)
Rhodothermia
Saprospiria
Spartobacteria
Sphingobacteriia
Verrucomicrobiae
NA
Order
Bacteroidales
Balneolales
Chitinispirillales
Chitinivibrionales
Chitinophagales
Chlamydiales
Chlorobiales
Chthoniobacterales
Cytophagales
Deinococcales
Fibrobacterales
Flavobacteriales
Gemmatimonadales
Ignavibacteriales
Lentisphaerales
Marinilabiliales
Nitrospirales
Opitutales
Phycisphaerales
Planctomycetales
Puniceicoccales
Rhodothermales
Saprospirales
Sphingobacteriales
Thermales
Verrucomicrobiales
Victivallales
NA
Family
Akkermansiaceae
Bacteroidaceae
Balneicellaceae
Balneolaceae
Barnesiellaceae
Bernardetiaceae
Chitinispirillaceae
Chitinivibrionaceae
Chitinophagaceae
Chlamydiaceae
Chlorobiaceae
Chthoniobacteraceae
Crocinitomicaceae
Cryomorphaceae
Cyclobacteriaceae
Cytophagaceae
Deinococcaceae
Dysgonomonadaceae
Fibrobacteraceae
Flammeovirgaceae
Flavobacteriaceae
Gemmataceae
Gemmatimonadaceae
Haliscomenobacteraceae
Hymenobacteraceae
Ichthyobacteriaceae
Ignavibacteriaceae
Isosphaeraceae
Lentimicrobiaceae
Lentisphaeraceae
Lewinellaceae
Marinifilaceae
Marinilabiliaceae
Melioribacteraceae
Microscillaceae
Family (continued)
Mooreiaceae
Nitrospiraceae
Opitutaceae
Paludibacteraceae
Parachlamydiaceae
Phycisphaeraceae
Planctomycetaceae
Porphyromonadaceae
Prevotellaceae
Prolixibacteraceae
Puniceicoccaceae
Rhodothermaceae
Rikenellaceae
Rubricoccaceae
Rubritaleaceae
Salinibacteraceae
Salinivirgaceae
Schleiferiaceae
Simkaniaceae
Sphingobacteriaceae
Tannerellaceae
Thermaceae
Thermonemataceae
Trueperaceae
Verrucomicrobiaceae
Victivallaceae
Waddliaceae
Williamwhitmaniaceae
NA
Percent G+C
Min. (29.0)
Max. (73.2)
Carotenoids
no
yes
NA
Flexirubin
no
yes
NA
Gliding
no
yes
NA
Oxygen
aerobic
anaerobic
facultatively aerobic
facultatively anaerobic
microaerophilic
NA
Menaquinones
Min. (4.0)
Max. (12.5)
Cell Length
Min. (0.3)
Max. (100.0)
Cell Width
Min. (0.1)
Max. (2.2)
Sequence length (in bp)
Min. (1,048,257)
Max. (12,363,577)
Tree scale: 0.01
strain (type species highlighted) ①
classification (phylum, class, order, family)
G+C
phenotype (carotenoids, flexirubin, gliding, oxygen, menaquinones, cell length and cell width)
total sequence length (in bp)
Bacteroides coagulans DSM 20705
Bacteroides xylanolyticus DSM 3808
Bacteroides galacturonicus DSM 3978
Bacteroides pectinophilus ATCC 43243
Leptospirillum ferriphilum DSM 14647
Nitrospira moscoviensis NSP M-1
Thermodesulfovibrio hydrogeniphilus DSM 18151
Thermodesulfovibrio thiophilus DSM 17215
Thermodesulfovibrio aggregans TGE-P1
Thermodesulfovibrio islandicus DSM 12570
Thermodesulfovibrio yellowstonii DSM 11347
Lentisphaera araneosa HTCC2155
Fibrobacter succinogenes S85
Fibrobacter intestinalis ATCC 43854
Victivallis vadensis DSM 14823
Chitinivibrio alkaliphilus DSM 24538
Chitinispirillum alkaliphilum ACht6-1
Simkania negevensis Z
Waddlia chondrophila WSU 86-1044
Parachlamydia acanthamoebae Bn9
Chlamydia trachomatis A/Har-13
Chlamydia pecorum E58
Chlamydia pneumoniae TW-183
Chlamydia psittaci 6BC
Chlamydia caviae GPIC
Chlamydia gallinacea DSM 27451
Chlamydia avium 10DC88
Coraliomargarita akajimensis DSM 45221
Opitutus terrae PB90-1
Cephaloticoccus primus CAG34
Cephaloticoccus capnophilus CV41
Chthoniobacter flavus Ellin428
Terrimicrobium sacchariphilum JCM 17479
Rubritalea marina DSM 17716
Rubritalea squalenifaciens DSM 18772
Akkermansia glycaniphila Pyt
Akkermansia muciniphila ATCC BAA-835

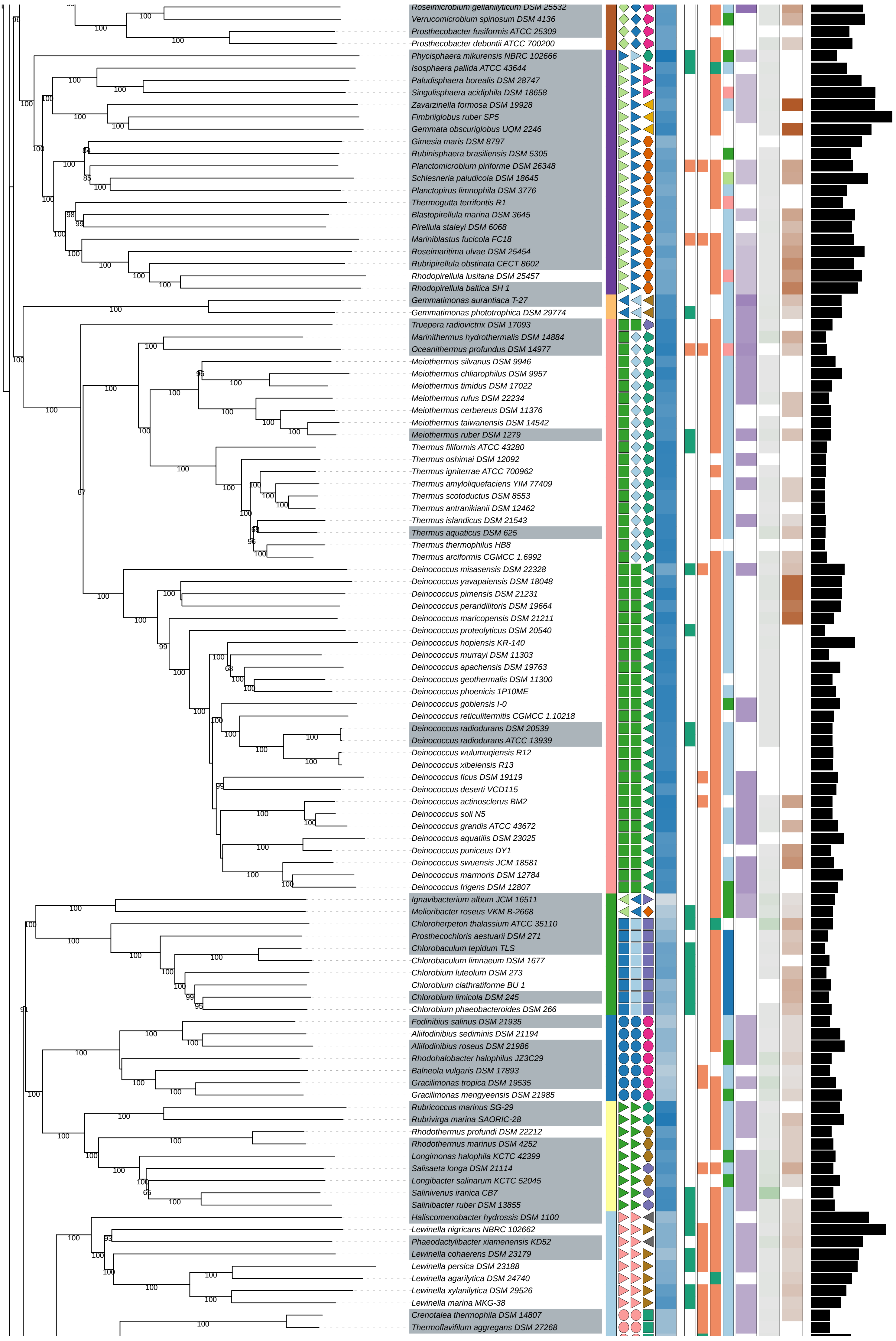
Roseimicrobium gellanilyticum DSM 25532
Verrucomicrobium spinosum DSM 4136
Prosthecobacter fusiformis ATCC 25309
Prosthecobacter debontii ATCC 700200
Phycisphaera mikurensis NBRC 102666
Isosphaera pallida ATCC 43644
Paludisphaera borealis DSM 28747
Singulisphaera acidiphila DSM 18658
Zavarzinella formosa DSM 19928
Fimbriiglobus ruber SP5
Gemmata obscuriglobus UQM 2246
Gimesia maris DSM 8797
Rubinisphaera brasiliensis DSM 5305
Planctomicrobium piriforme DSM 26348
Schlesneria paludicola DSM 18645
Planctopirus limnophila DSM 3776
Thermogutta terrifontis R1
Blastopirellula marina DSM 3645
Pirellula staleyi DSM 6068
Mariniblastus fucicola FC18
Roseimaritima ulvae DSM 25454
Rubripirellula obstinata CECT 8602
Rhodopirellula lusitana DSM 25457
Rhodopirellula baltica SH 1
Gemmatimonas aurantiaca T-27
Gemmatimonas phototrophica DSM 29774
Truepera radiovictrix DSM 17093
Marinithermus hydrothermalis DSM 14884
Oceanithermus profundus DSM 14977
Meiothermus silvanus DSM 9946
Meiothermus chliarophilus DSM 9957
Meiothermus timidus DSM 17022
Meiothermus rufus DSM 22234
Meiothermus cerbereus DSM 11376
Meiothermus taiwanensis DSM 14542
Meiothermus ruber DSM 1279
Thermus filiformis ATCC 43280
Thermus oshimai DSM 12092
Thermus igniterrae ATCC 700962
Thermus amyloliquefaciens YIM 77409
Thermus scotoductus DSM 8553
Thermus antranikianii DSM 12462
Thermus islandicus DSM 21543
Thermus aquaticus DSM 625
Thermus thermophilus HB8
Thermus arciformis CGMCC 1.6992
Deinococcus misasensis DSM 22328
Deinococcus yavapaiensis DSM 18048
Deinococcus pimensis DSM 21231
Deinococcus peraridilitoris DSM 19664
Deinococcus maricopensis DSM 21211
Deinococcus proteolyticus DSM 20540
Deinococcus hopiensis KR-140
Deinococcus murrayi DSM 11303
Deinococcus apachensis DSM 19763
Deinococcus geothermalis DSM 11300
Deinococcus phoenicis 1P10ME
Deinococcus gobiensis I-0
Deinococcus reticulitermitis CGMCC 1.10218
Deinococcus radiodurans DSM 20539
Deinococcus radiodurans ATCC 13939
Deinococcus wulumuqiensis R12
Deinococcus xibeiensis R13
Deinococcus ficus DSM 19119
Deinococcus deserti VCD115
Deinococcus actinosclerus BM2
Deinococcus soli N5
Deinococcus grandis ATCC 43672
Deinococcus aquatilis DSM 23025
Deinococcus puniceus DY1
Deinococcus swuensis JCM 18581
Deinococcus marmoris DSM 12784
Deinococcus frigens DSM 12807
Ignavibacterium album JCM 16511
Melioribacter roseus VKM B-2668
Chloroherpeton thalassium ATCC 35110
Prosthecochloris aestuarii DSM 271
Chlorobaculum tepidum TLS
Chlorobaculum limnaeum DSM 1677
Chlorobium luteolum DSM 273
Chlorobium clathratiforme BU 1
Chlorobium limicola DSM 245
Chlorobium phaeobacteroides DSM 266
Fodinibius salinus DSM 21935
Aliifodinibius sediminis DSM 21194
Aliifodinibius roseus DSM 21986
Rhodohalobacter halophilus JZ3C29
Balneola vulgaris DSM 17893
Gracilimonas tropica DSM 19535
Gracilimonas mengyeensis DSM 21985
Rubricoccus marinus SG-29
Rubrivirga marina SAORIC-28
Rhodothermus profundi DSM 22212
Rhodothermus marinus DSM 4252
Longimonas halophila KCTC 42399
Salisaeta longa DSM 21114
Longibacter salinarum KCTC 52045
Salinivenus iranica CB7
Salinibacter ruber DSM 13855
Haliscomenobacter hydrossis DSM 1100
Lewinella nigricans NBRC 102662
Phaeodactylibacter xiamenensis KD52
Lewinella cohaerens DSM 23179
Lewinella persica DSM 23188
Lewinella agarilytica DSM 24740
Lewinella xylanilytica DSM 29526
Lewinella marina MKG-38
Crenotalea thermophila DSM 14807
Thermoflavifilum aggregans DSM 27268

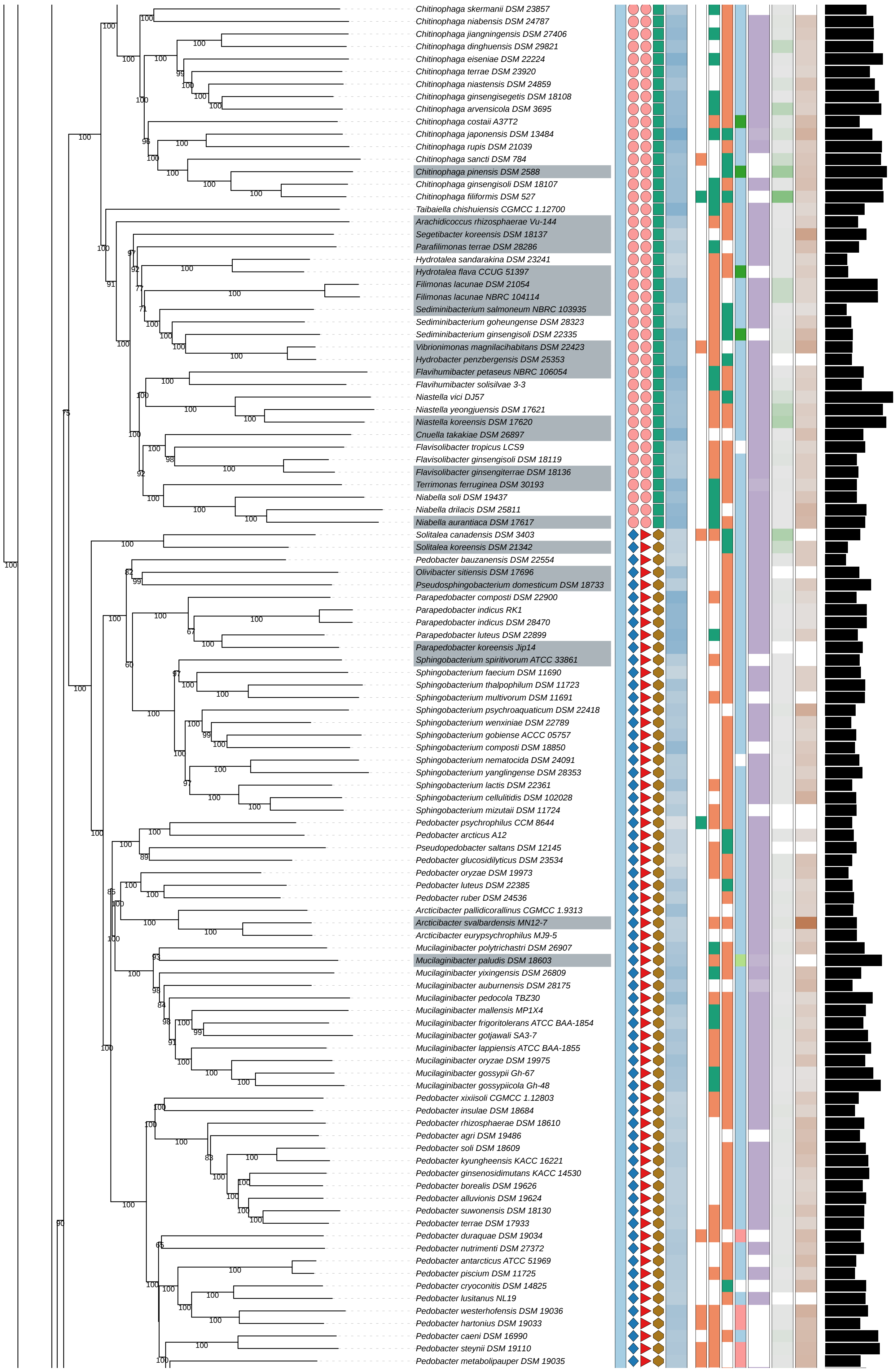
Chitinophaga skermanii DSM 23857
Chitinophaga niabensis DSM 24787
Chitinophaga jiangningensis DSM 27406
Chitinophaga dinghuensis DSM 29821
Chitinophaga eiseniae DSM 22224
Chitinophaga terrae DSM 23920
Chitinophaga niastensis DSM 24859
Chitinophaga ginsengisegetis DSM 18108
Chitinophaga arvensicola DSM 3695
Chitinophaga costaii A37T2
Chitinophaga japonensis DSM 13484
Chitinophaga rupis DSM 21039
Chitinophaga sancti DSM 784
Chitinophaga pinensis DSM 2588
Chitinophaga ginsengisoli DSM 18107
Chitinophaga filiformis DSM 527
Taibaiella chishuiensis CGMCC 1.12700
Arachidicoccus rhizosphaerae Vu-144
Segetibacter koreensis DSM 18137
Parafilimonas terrae DSM 28286
Hydrotalea sandarakina DSM 23241
Hydrotalea flava CCUG 51397
Filimonas lacunae DSM 21054
Filimonas lacunae NBRC 104114
Sediminibacterium salmoneum NBRC 103935
Sediminibacterium goheungense DSM 28323
Sediminibacterium ginsengisoli DSM 22335
Vibrionimonas magnilacihabitans DSM 22423
Hydrobacter penzbergensis DSM 25353
Flavihumibacter petaseus NBRC 106054
Flavihumibacter solisilvae 3-3
Niastella vici DJ57
Niastella yeongjuensis DSM 17621
Niastella koreensis DSM 17620
Cnuella takakiae DSM 26897
Flavisolibacter tropicus LCS9
Flavisolibacter ginsengisoli DSM 18119
Flavisolibacter ginsengiterrae DSM 18136
Terrimonas ferruginea DSM 30193
Niabella soli DSM 19437
Niabella drilacis DSM 25811
Niabella aurantiaca DSM 17617
Solitalea canadensis DSM 3403
Solitalea koreensis DSM 21342
Pedobacter bauzanensis DSM 22554
Olivibacter sitiensis DSM 17696
Pseudosphingobacterium domesticum DSM 18733
Parapedobacter composti DSM 22900
Parapedobacter indicus RK1
Parapedobacter indicus DSM 28470
Parapedobacter luteus DSM 22899
Parapedobacter koreensis Jip14
Sphingobacterium spiritivorum ATCC 33861
Sphingobacterium faecium DSM 11690
Sphingobacterium thalpophilum DSM 11723
Sphingobacterium multivorum DSM 11691
Sphingobacterium psychroaquaticum DSM 22418
Sphingobacterium wenxiniae DSM 22789
Sphingobacterium gobiense ACCC 05757
Sphingobacterium composti DSM 18850
Sphingobacterium nematocida DSM 24091
Sphingobacterium yanglingense DSM 28353
Sphingobacterium lactis DSM 22361
Sphingobacterium cellulitidis DSM 102028
Sphingobacterium mizutaii DSM 11724
Pedobacter psychrophilus CCM 8644
Pedobacter arcticus A12
Pseudopedobacter saltans DSM 12145
Pedobacter glucosidilyticus DSM 23534
Pedobacter oryzae DSM 19973
Pedobacter luteus DSM 22385
Pedobacter ruber DSM 24536
Arcticibacter pallidicorallinus CGMCC 1.9313
Arcticibacter svalbardensis MN12-7
Arcticibacter eurypsychrophilus MJ9-5
Mucilaginibacter polytrichastri DSM 26907
Mucilaginibacter paludis DSM 18603
Mucilaginibacter yixingensis DSM 26809
Mucilaginibacter auburnensis DSM 28175
Mucilaginibacter pedocola TBZ30
Mucilaginibacter mallensis MP1X4
Mucilaginibacter frigoritolerans ATCC BAA-1854
Mucilaginibacter gotjawali SA3-7
Mucilaginibacter lappiensis ATCC BAA-1855
Mucilaginibacter oryzae DSM 19975
Mucilaginibacter gossypii Gh-67
Mucilaginibacter gossypiicola Gh-48
Pedobacter xixiisoli CGMCC 1.12803
Pedobacter insulae DSM 18684
Pedobacter rhizosphaerae DSM 18610
Pedobacter agri DSM 19486
Pedobacter soli DSM 18609
Pedobacter kyungheensis KACC 16221
Pedobacter ginsenosidimutans KACC 14530
Pedobacter borealis DSM 19626
Pedobacter alluvionis DSM 19624
Pedobacter suwonensis DSM 18130
Pedobacter terrae DSM 17933
Pedobacter duraquae DSM 19034
Pedobacter nutrimenti DSM 27372
Pedobacter antarcticus ATCC 51969
Pedobacter piscium DSM 11725
Pedobacter cryoconitis DSM 14825
Pedobacter lusitanus NL19
Pedobacter westerhofensis DSM 19036
Pedobacter hartonius DSM 19033
Pedobacter caeni DSM 16990
Pedobacter steynii DSM 19110
Pedobacter metabolipauper DSM 19035

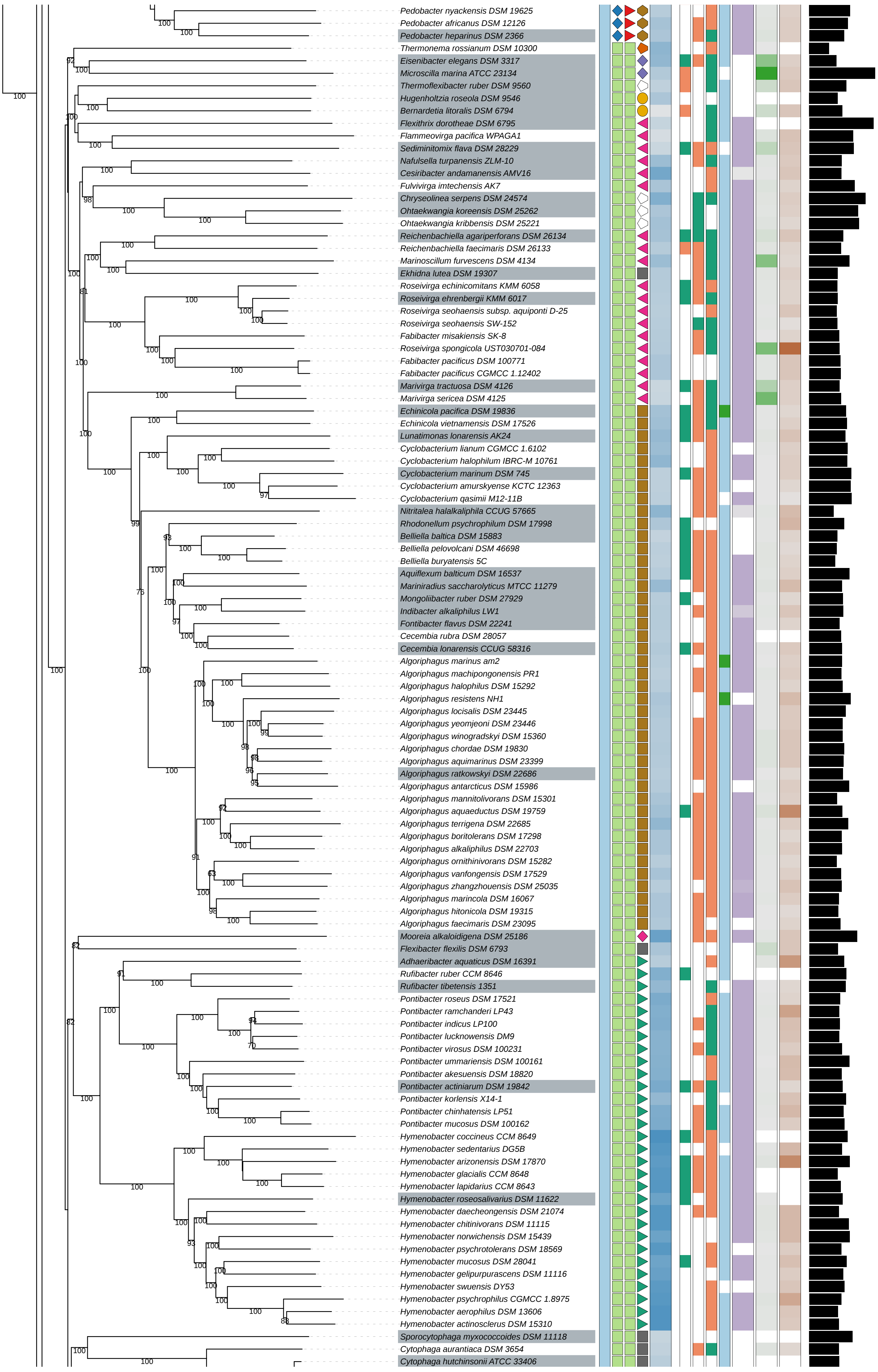
Pedobacter nyackensis DSM 19625
Pedobacter africanus DSM 12126
Pedobacter heparinus DSM 2366
Thermonema rossianum DSM 10300
Eisenibacter elegans DSM 3317
Microscilla marina ATCC 23134
Thermoflexibacter ruber DSM 9560
Hugenholtzia roseola DSM 9546
Bernardetia litoralis DSM 6794
Flexithrix dorotheae DSM 6795
Flammeovirga pacifica WPAGA1
Sediminitomix flava DSM 28229
Nafulsella turpanensis ZLM-10
Cesiribacter andamanensis AMV16
Fulvivirga imtechensis AK7
Chryseolinea serpens DSM 24574
Ohtaekwangia koreensis DSM 25262
Ohtaekwangia kribbensis DSM 25221
Reichenbachiella agariperforans DSM 26134
Reichenbachiella faecimaris DSM 26133
Marinoscillum furvescens DSM 4134
Ekhidna lutea DSM 19307
Roseivirga echinicomitans KMM 6058
Roseivirga ehrenbergii KMM 6017
Roseivirga seohaensis subsp. aquiponti D-25
Roseivirga seohaensis SW-152
Fabibacter misakiensis SK-8
Roseivirga spongicola UST030701-084
Fabibacter pacificus DSM 100771
Fabibacter pacificus CGMCC 1.12402
Marivirga tractuosa DSM 4126
Marivirga sericea DSM 4125
Echinicola pacifica DSM 19836
Echinicola vietnamensis DSM 17526
Lunatimonas lonarensis AK24
Cyclobacterium lianum CGMCC 1.6102
Cyclobacterium halophilum IBRC-M 10761
Cyclobacterium marinum DSM 745
Cyclobacterium amurskyense KCTC 12363
Cyclobacterium qasimii M12-11B
Nitritalea halalkaliphila CCUG 57665
Rhodonellum psychrophilum DSM 17998
Belliella baltica DSM 15883
Belliella pelovolcani DSM 46698
Belliella buryatensis 5C
Aquiflexum balticum DSM 16537
Mariniradius saccharolyticus MTCC 11279
Mongoliibacter ruber DSM 27929
Indibacter alkaliphilus LW1
Fontibacter flavus DSM 22241
Cecembia rubra DSM 28057
Cecembia lonarensis CCUG 58316
Algoriphagus marinus am2
Algoriphagus machipongonensis PR1
Algoriphagus halophilus DSM 15292
Algoriphagus resistens NH1
Algoriphagus locisalis DSM 23445
Algoriphagus yeomjeoni DSM 23446
Algoriphagus winogradskyi DSM 15360
Algoriphagus chordae DSM 19830
Algoriphagus aquimarinus DSM 23399
Algoriphagus ratkowskyi DSM 22686
Algoriphagus antarcticus DSM 15986
Algoriphagus mannitolivorans DSM 15301
Algoriphagus aquaeductus DSM 19759
Algoriphagus terrigena DSM 22685
Algoriphagus boritolerans DSM 17298
Algoriphagus alkaliphilus DSM 22703
Algoriphagus ornithinivorans DSM 15282
Algoriphagus vanfongensis DSM 17529
Algoriphagus zhangzhouensis DSM 25035
Algoriphagus marincola DSM 16067
Algoriphagus hitonicola DSM 19315
Algoriphagus faecimaris DSM 23095
Mooreia alkaloidigena DSM 25186
Flexibacter flexilis DSM 6793
Adhaeribacter aquaticus DSM 16391
Rufibacter ruber CCM 8646
Rufibacter tibetensis 1351
Pontibacter roseus DSM 17521
Pontibacter ramchanderi LP43
Pontibacter indicus LP100
Pontibacter lucknowensis DM9
Pontibacter virosus DSM 100231
Pontibacter ummariensis DSM 100161
Pontibacter akesuensis DSM 18820
Pontibacter actiniarum DSM 19842
Pontibacter korlensis X14-1
Pontibacter chinhatensis LP51
Pontibacter mucosus DSM 100162
Hymenobacter coccineus CCM 8649
Hymenobacter sedentarius DG5B
Hymenobacter arizonensis DSM 17870
Hymenobacter glacialis CCM 8648
Hymenobacter lapidarius CCM 8643
Hymenobacter roseosalivarius DSM 11622
Hymenobacter daecheongensis DSM 21074
Hymenobacter chitinivorans DSM 11115
Hymenobacter norwichensis DSM 15439
Hymenobacter psychrotolerans DSM 18569
Hymenobacter mucosus DSM 28041
Hymenobacter gelipurpurascens DSM 11116
Hymenobacter swuensis DY53
Hymenobacter psychrophilus CGMCC 1.8975
Hymenobacter aerophilus DSM 13606
Hymenobacter actinosclerus DSM 15310
Sporocytophaga myxococcoides DSM 11118
Cytophaga aurantiaca DSM 3654
Cytophaga hutchinsonii ATCC 33406

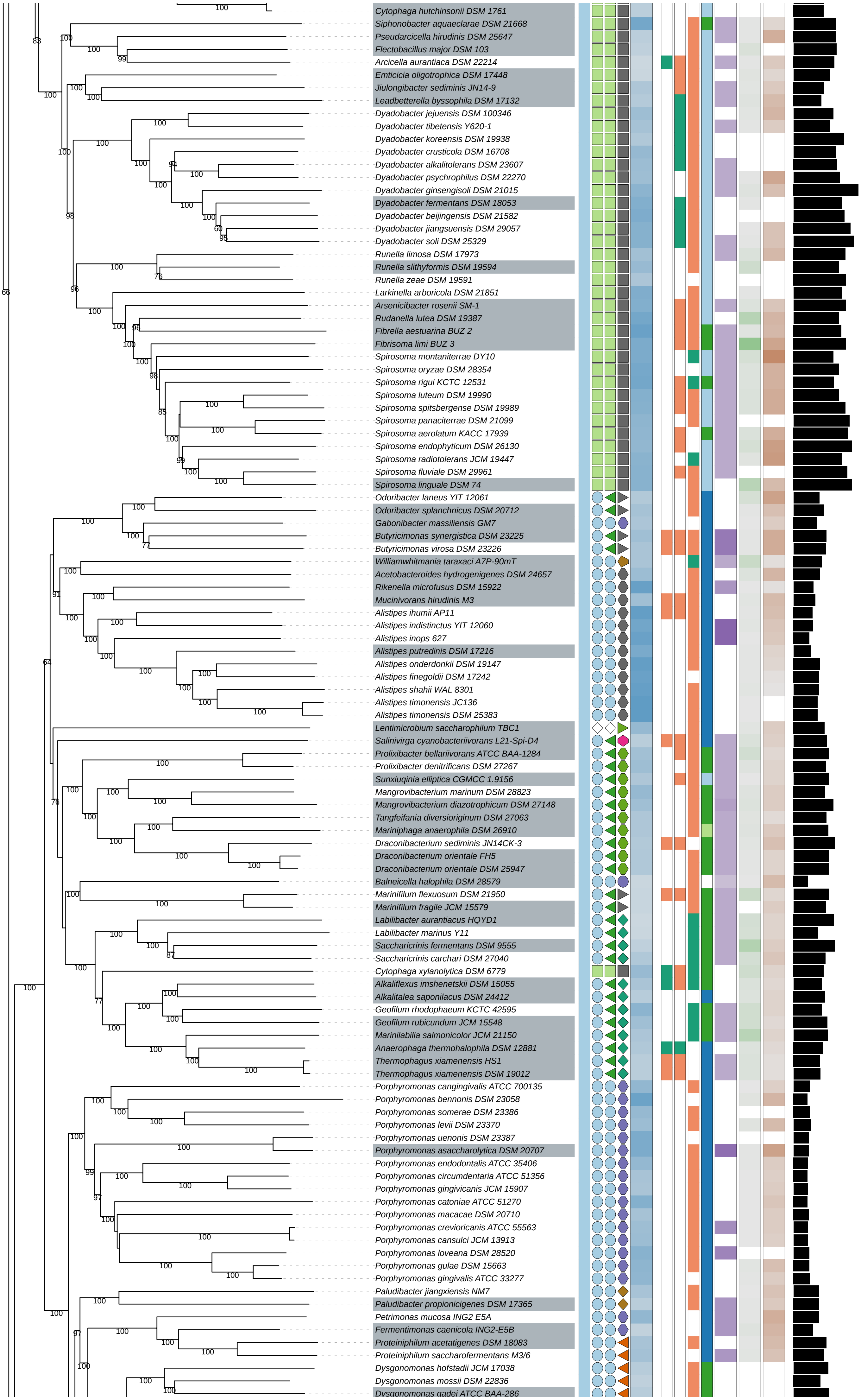

Cytophaga hutchinsonii DSM 1761
Siphonobacter aquaeclarae DSM 21668
Pseudarcicella hirudinis DSM 25647
Flectobacillus major DSM 103
Arcicella aurantiaca DSM 22214
Emticicia oligotrophica DSM 17448
Jiulongibacter sediminis JN14-9
Leadbetterella byssophila DSM 17132
Dyadobacter jejuensis DSM 100346
Dyadobacter tibetensis Y620-1
Dyadobacter koreensis DSM 19938
Dyadobacter crusticola DSM 16708
Dyadobacter alkalitolerans DSM 23607
Dyadobacter psychrophilus DSM 22270
Dyadobacter ginsengisoli DSM 21015
Dyadobacter fermentans DSM 18053
Dyadobacter beijingensis DSM 21582
Dyadobacter jiangsuensis DSM 29057
Dyadobacter soli DSM 25329
Runella limosa DSM 17973
Runella slithyformis DSM 19594
Runella zeae DSM 19591
Larkinella arboricola DSM 21851
Arsenicibacter rosenii SM-1
Rudanella lutea DSM 19387
Fibrella aestuarina BUZ 2
Fibrisoma limi BUZ 3
Spirosoma montaniterrae DY10
Spirosoma oryzae DSM 28354
Spirosoma rigui KCTC 12531
Spirosoma luteum DSM 19990
Spirosoma spitsbergense DSM 19989
Spirosoma panaciterrae DSM 21099
Spirosoma aerolatum KACC 17939
Spirosoma endophyticum DSM 26130
Spirosoma radiotolerans JCM 19447
Spirosoma fluviale DSM 29961
Spirosoma linguale DSM 74
Odoribacter laneus YIT 12061
Odoribacter splanchnicus DSM 20712
Gabonibacter massiliensis GM7
Butyricimonas synergistica DSM 23225
Butyricimonas virosa DSM 23226
Williamwhitmania taraxaci A7P-90mT
Acetobacteroides hydrogenigenes DSM 24657
Rikenella microfusus DSM 15922
Mucinivorans hirudinis M3
Alistipes ihumii AP11
Alistipes indistinctus YIT 12060
Alistipes inops 627
Alistipes putredinis DSM 17216
Alistipes onderdonkii DSM 19147
Alistipes finegoldii DSM 17242
Alistipes shahii WAL 8301
Alistipes timonensis JC136
Alistipes timonensis DSM 25383
Lentimicrobium saccharophilum TBC1
Salinivirga cyanobacteriivorans L21-Spi-D4
Prolixibacter bellariivorans ATCC BAA-1284
Prolixibacter denitrificans DSM 27267
Sunxiuqinia elliptica CGMCC 1.9156
Mangrovibacterium marinum DSM 28823
Mangrovibacterium diazotrophicum DSM 27148
Tangfeifania diversioriginum DSM 27063
Mariniphaga anaerophila DSM 26910
Draconibacterium sediminis JN14CK-3
Draconibacterium orientale FH5
Draconibacterium orientale DSM 25947
Balneicella halophila DSM 28579
Marinifilum flexuosum DSM 21950
Marinifilum fragile JCM 15579
Labilibacter aurantiacus HQYD1
Labilibacter marinus Y11
Saccharicrinis fermentans DSM 9555
Saccharicrinis carchari DSM 27040
Cytophaga xylanolytica DSM 6779
Alkaliflexus imshenetskii DSM 15055
Alkalitalea saponilacus DSM 24412
Geofilum rhodophaeum KCTC 42595
Geofilum rubicundum JCM 15548
Marinilabilia salmonicolor JCM 21150
Anaerophaga thermohalophila DSM 12881
Thermophagus xiamenensis HS1
Thermophagus xiamenensis DSM 19012
Porphyromonas cangingivalis ATCC 700135
Porphyromonas bennonis DSM 23058
Porphyromonas somerae DSM 23386
Porphyromonas levii DSM 23370
Porphyromonas uenonis DSM 23387
Porphyromonas asaccharolytica DSM 20707
Porphyromonas endodontalis ATCC 35406
Porphyromonas circumdentaria ATCC 51356
Porphyromonas gingivicanis JCM 15907
Porphyromonas catoniae ATCC 51270
Porphyromonas macacae DSM 20710
Porphyromonas crevioricanis ATCC 55563
Porphyromonas cansulci JCM 13913
Porphyromonas loveana DSM 28520
Porphyromonas gulae DSM 15663
Porphyromonas gingivalis ATCC 33277
Paludibacter jiangxiensis NM7
Paludibacter propionicigenes DSM 17365
Petrimonas mucosa ING2 E5A
Fermentimonas caenicola ING2-E5B
Proteiniphilum acetatigenes DSM 18083
Proteiniphilum saccharofermentans M3/6
Dysgonomonas hofstadii JCM 17038
Dysgonomonas mossii DSM 22836
Dysgonomonas gadei ATCC BAA-286

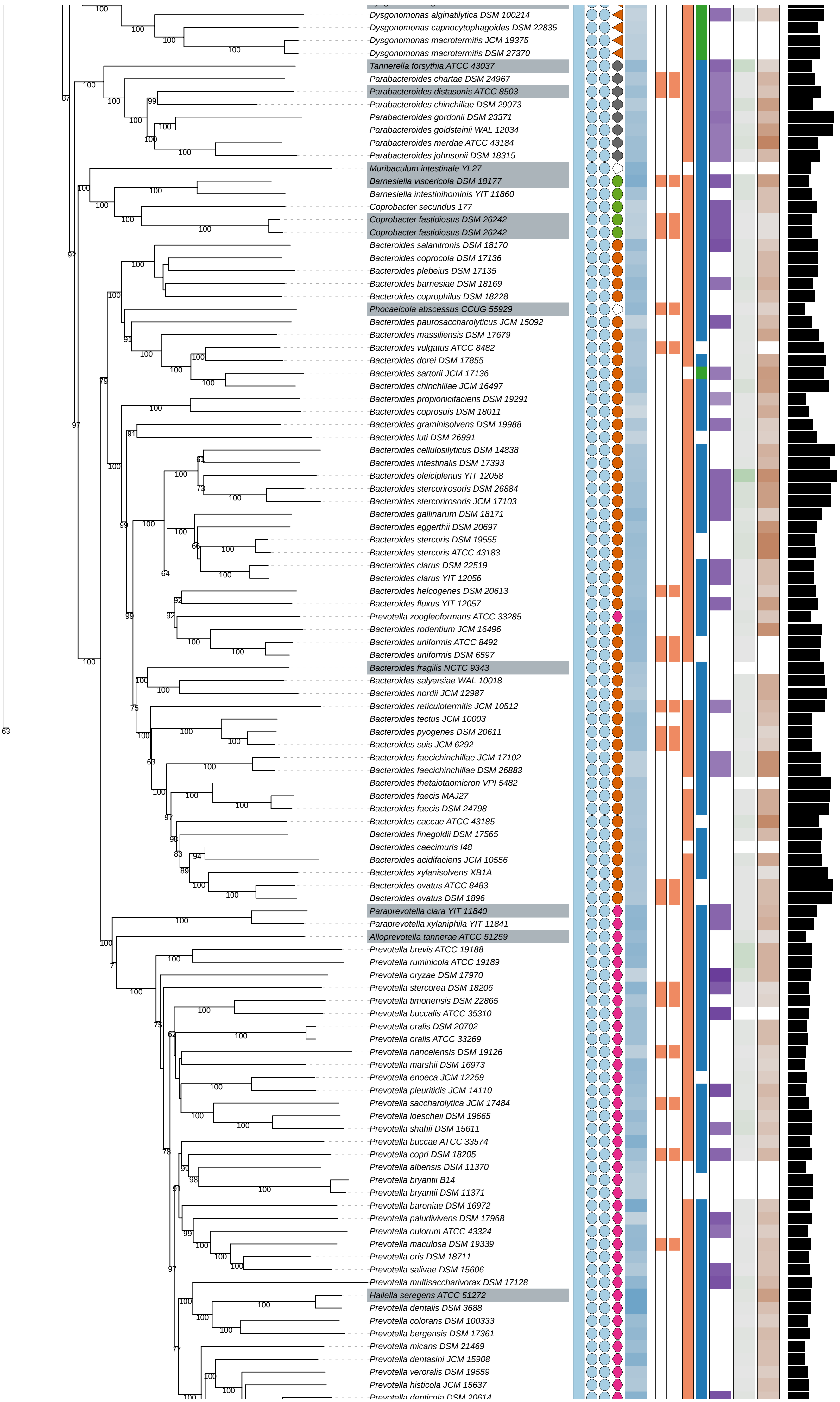

Dysgonomonas alginatilytica DSM 100214
Dysgonomonas capnocytophagoides DSM 22835
Dysgonomonas macrotermitis JCM 19375
Dysgonomonas macrotermitis DSM 27370
Tannerella forsythia ATCC 43037
Parabacteroides chartae DSM 24967
Parabacteroides distasonis ATCC 8503
Parabacteroides chinchillae DSM 29073
Parabacteroides gordonii DSM 23371
Parabacteroides goldsteinii WAL 12034
Parabacteroides merdae ATCC 43184
Parabacteroides johnsonii DSM 18315
Muribaculum intestinale YL27
Barnesiella viscericola DSM 18177
Barnesiella intestinihominis YIT 11860
Coprobacter secundus 177
Coprobacter fastidiosus DSM 26242
Coprobacter fastidiosus DSM 26242
Bacteroides salanitronis DSM 18170
Bacteroides coprocola DSM 17136
Bacteroides plebeius DSM 17135
Bacteroides barnesiae DSM 18169
Bacteroides coprophilus DSM 18228
Phocaeicola abscessus CCUG 55929
Bacteroides paurosaccharolyticus JCM 15092
Bacteroides massiliensis DSM 17679
Bacteroides vulgatus ATCC 8482
Bacteroides dorei DSM 17855
Bacteroides sartorii JCM 17136
Bacteroides chinchillae JCM 16497
Bacteroides propionicifaciens DSM 19291
Bacteroides coprosuis DSM 18011
Bacteroides graminisolvens DSM 19988
Bacteroides luti DSM 26991
Bacteroides cellulosilyticus DSM 14838
Bacteroides intestinalis DSM 17393
Bacteroides oleiciplenus YIT 12058
Bacteroides stercorirosoris DSM 26884
Bacteroides stercorirosoris JCM 17103
Bacteroides gallinarum DSM 18171
Bacteroides eggerthii DSM 20697
Bacteroides stercoris DSM 19555
Bacteroides stercoris ATCC 43183
Bacteroides clarus DSM 22519
Bacteroides clarus YIT 12056
Bacteroides helcogenes DSM 20613
Bacteroides fluxus YIT 12057
Prevotella zoogleoformans ATCC 33285
Bacteroides rodentium JCM 16496
Bacteroides uniformis ATCC 8492
Bacteroides uniformis DSM 6597
Bacteroides fragilis NCTC 9343
Bacteroides salyersiae WAL 10018
Bacteroides nordii JCM 12987
Bacteroides reticulotermitis JCM 10512
Bacteroides tectus JCM 10003
Bacteroides pyogenes DSM 20611
Bacteroides suis JCM 6292
Bacteroides faecichinchillae JCM 17102
Bacteroides faecichinchillae DSM 26883
Bacteroides thetaiotaomicron VPI 5482
Bacteroides faecis MAJ27
Bacteroides faecis DSM 24798
Bacteroides caccae ATCC 43185
Bacteroides finegoldii DSM 17565
Bacteroides caecimuris I48
Bacteroides acidifaciens JCM 10556
Bacteroides xylanisolvens XB1A
Bacteroides ovatus ATCC 8483
Bacteroides ovatus DSM 1896
Paraprevotella clara YIT 11840
Paraprevotella xylaniphila YIT 11841
Alloprevotella tannerae ATCC 51259
Prevotella brevis ATCC 19188
Prevotella ruminicola ATCC 19189
Prevotella oryzae DSM 17970
Prevotella stercorea DSM 18206
Prevotella timonensis DSM 22865
Prevotella buccalis ATCC 35310
Prevotella oralis DSM 20702
Prevotella oralis ATCC 33269
Prevotella nanceiensis DSM 19126
Prevotella marshii DSM 16973
Prevotella enoeca JCM 12259
Prevotella pleuritidis JCM 14110
Prevotella saccharolytica JCM 17484
Prevotella loescheii DSM 19665
Prevotella shahii DSM 15611
Prevotella buccae ATCC 33574
Prevotella copri DSM 18205
Prevotella albensis DSM 11370
Prevotella bryantii B14
Prevotella bryantii DSM 11371
Prevotella baroniae DSM 16972
Prevotella paludivivens DSM 17968
Prevotella oulorum ATCC 43324
Prevotella maculosa DSM 19339
Prevotella oris DSM 18711
Prevotella salivae DSM 15606
Prevotella multisaccharivorax DSM 17128
Hallella seregens ATCC 51272
Prevotella dentalis DSM 3688
Prevotella colorans DSM 100333
Prevotella bergensis DSM 17361
Prevotella micans DSM 21469
Prevotella dentasini JCM 15908
Prevotella veroralis DSM 19559
Prevotella histicola JCM 15637
Prevotella denticola DSM 20614

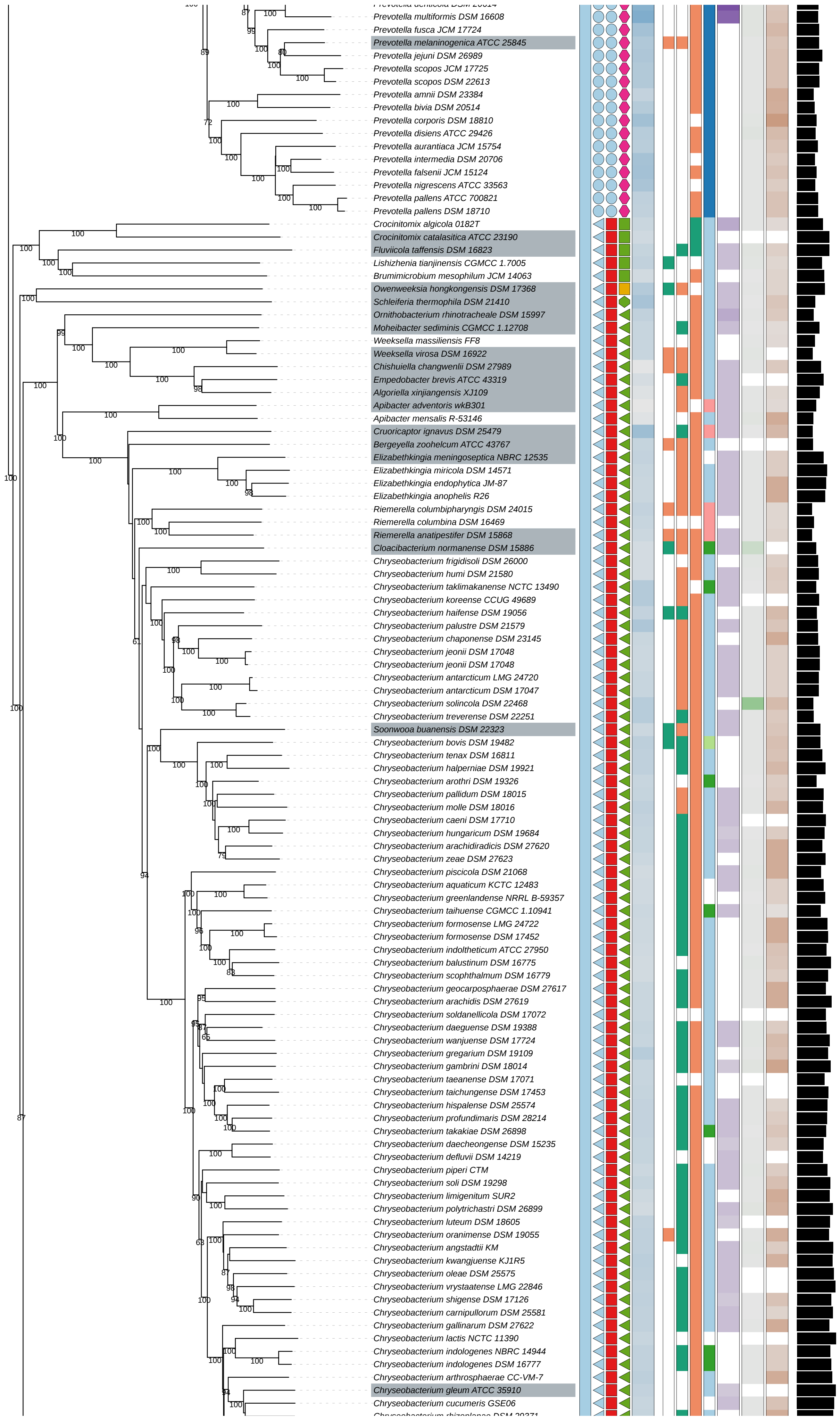

Prevotella multiformis DSM 16608
Prevotella fusca JCM 17724
Prevotella melaninogenica ATCC 25845
Prevotella jejuni DSM 26989
Prevotella scopos JCM 17725
Prevotella scopos DSM 22613
Prevotella amnii DSM 23384
Prevotella bivia DSM 20514
Prevotella corporis DSM 18810
Prevotella disiens ATCC 29426
Prevotella aurantiaca JCM 15754
Prevotella intermedia DSM 20706
Prevotella falsenii JCM 15124
Prevotella nigrescens ATCC 33563
Prevotella pallens ATCC 700821
Prevotella pallens DSM 18710
Crocinitomix algicola 0182T
Crocinitomix catalasitica ATCC 23190
Fluviicola taffensis DSM 16823
Lishizhenia tianjinensis CGMCC 1.7005
Brumimicrobium mesophilum JCM 14063
Owenweeksia hongkongensis DSM 17368
Schleiferia thermophila DSM 21410
Ornithobacterium rhinotracheale DSM 15997
Moheibacter sediminis CGMCC 1.12708
Weeksella massiliensis FF8
Weeksella virosa DSM 16922
Chishuiella changwenlii DSM 27989
Empedobacter brevis ATCC 43319
Algoriella xinjiangensis XJ109
Apibacter adventoris wkB301
Apibacter mensalis R-53146
Cruoricaptor ignavus DSM 25479
Bergeyella zoohelcum ATCC 43767
Elizabethkingia meningoseptica NBRC 12535
Elizabethkingia miricola DSM 14571
Elizabethkingia endophytica JM-87
Elizabethkingia anophelis R26
Riemerella columbipharyngis DSM 24015
Riemerella columbina DSM 16469
Riemerella anatipestifer DSM 15868
Cloacibacterium normanense DSM 15886
Chryseobacterium frigidisoli DSM 26000
Chryseobacterium humi DSM 21580
Chryseobacterium taklimakanense NCTC 13490
Chryseobacterium koreense CCUG 49689
Chryseobacterium haifense DSM 19056
Chryseobacterium palustre DSM 21579
Chryseobacterium chaponense DSM 23145
Chryseobacterium jeonii DSM 17048
Chryseobacterium jeonii DSM 17048
Chryseobacterium antarcticum LMG 24720
Chryseobacterium antarcticum DSM 17047
Chryseobacterium solincola DSM 22468
Chryseobacterium treverense DSM 22251
Soonwooa buanensis DSM 22323
Chryseobacterium bovis DSM 19482
Chryseobacterium tenax DSM 16811
Chryseobacterium halperniae DSM 19921
Chryseobacterium arothri DSM 19326
Chryseobacterium pallidum DSM 18015
Chryseobacterium molle DSM 18016
Chryseobacterium caeni DSM 17710
Chryseobacterium hungaricum DSM 19684
Chryseobacterium arachidiradicis DSM 27620
Chryseobacterium zeae DSM 27623
Chryseobacterium piscicola DSM 21068
Chryseobacterium aquaticum KCTC 12483
Chryseobacterium greenlandense NRRL B-59357
Chryseobacterium taihuense CGMCC 1.10941
Chryseobacterium formosense LMG 24722
Chryseobacterium formosense DSM 17452
Chryseobacterium indoltheticum ATCC 27950
Chryseobacterium balustinum DSM 16775
Chryseobacterium scophthalmum DSM 16779
Chryseobacterium geocarposphaerae DSM 27617
Chryseobacterium arachidis DSM 27619
Chryseobacterium soldanellicola DSM 17072
Chryseobacterium daeguense DSM 19388
Chryseobacterium wanjuense DSM 17724
Chryseobacterium gregarium DSM 19109
Chryseobacterium gambrini DSM 18014
Chryseobacterium taeanense DSM 17071
Chryseobacterium taichungense DSM 17453
Chryseobacterium hispalense DSM 25574
Chryseobacterium profundimaris DSM 28214
Chryseobacterium takakiae DSM 26898
Chryseobacterium daecheongense DSM 15235
Chryseobacterium defluvii DSM 14219
Chryseobacterium piperi CTM
Chryseobacterium soli DSM 19298
Chryseobacterium limigenitum SUR2
Chryseobacterium polytrichastri DSM 26899
Chryseobacterium luteum DSM 18605
Chryseobacterium oranimense DSM 19055
Chryseobacterium angstadtii KM
Chryseobacterium kwangjuense KJ1R5
Chryseobacterium oleae DSM 25575
Chryseobacterium vrystaatense LMG 22846
Chryseobacterium shigense DSM 17126
Chryseobacterium carnipullorum DSM 25581
Chryseobacterium gallinarum DSM 27622
Chryseobacterium lactis NCTC 11390
Chryseobacterium indologenes NBRC 14944
Chryseobacterium indologenes DSM 16777
Chryseobacterium arthrosphaerae CC-VM-7
Chryseobacterium gleum ATCC 35910
Chryseobacterium cucumeris GSE06

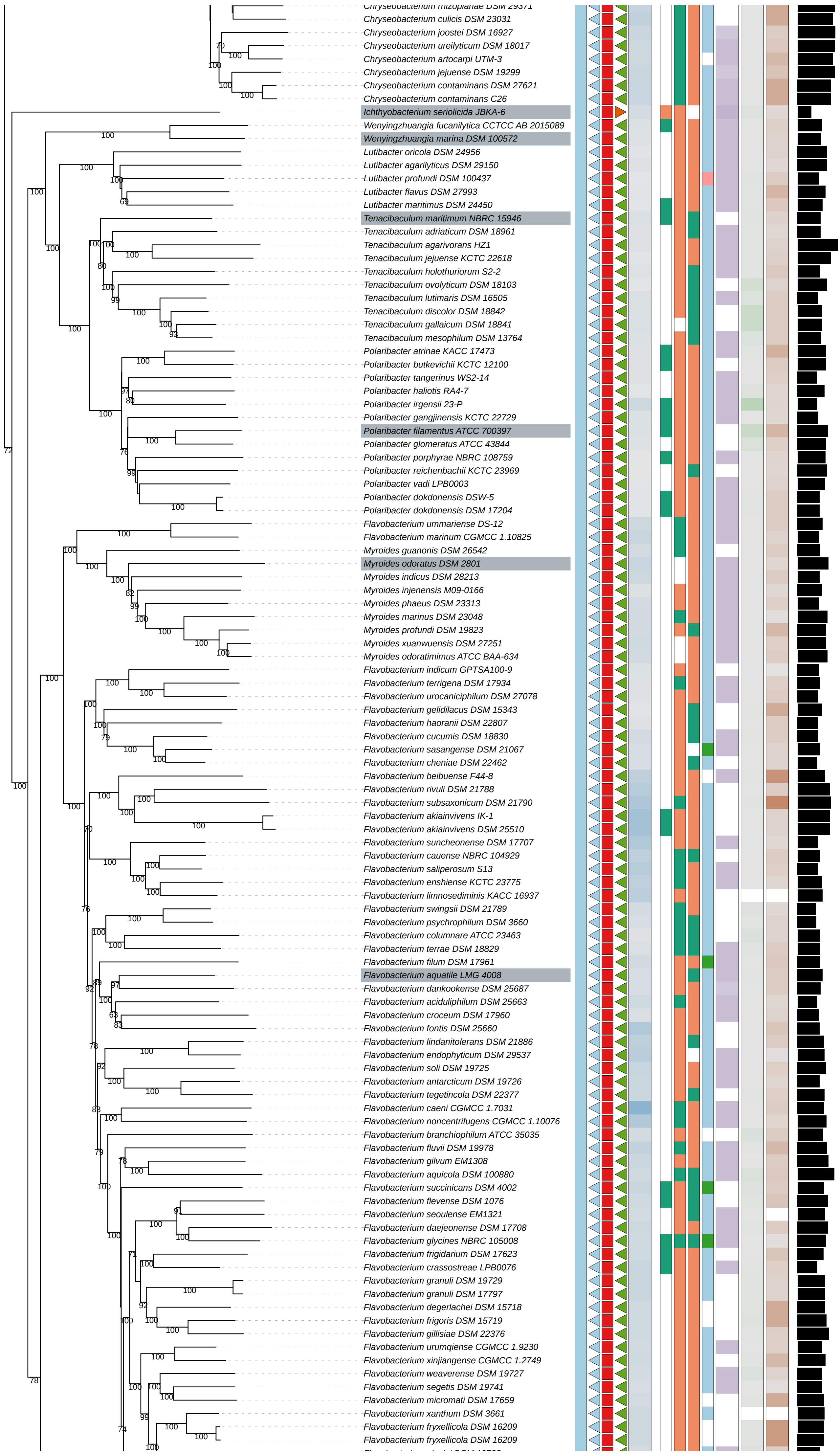
Chryseobacterium culicis DSM 23031
Chryseobacterium joostei DSM 16927
Chryseobacterium ureilyticum DSM 18017
Chryseobacterium artocarpi UTM-3
Chryseobacterium jejuense DSM 19299
Chryseobacterium contaminans DSM 27621
Chryseobacterium contaminans C26
Ichthyobacterium seriolicida JBKA-6
Wenyingzhuangia fucanilytica CCTCC AB 2015089
Wenyingzhuangia marina DSM 100572
Lutibacter oricola DSM 24956
Lutibacter agarilyticus DSM 29150
Lutibacter profundi DSM 100437
Lutibacter flavus DSM 27993
Lutibacter maritimus DSM 24450
Tenacibaculum maritimum NBRC 15946
Tenacibaculum adriaticum DSM 18961
Tenacibaculum agarivorans HZ1
Tenacibaculum jejuense KCTC 22618
Tenacibaculum holothuriorum S2-2
Tenacibaculum ovolyticum DSM 18103
Tenacibaculum lutimaris DSM 16505
Tenacibaculum discolor DSM 18842
Tenacibaculum gallaicum DSM 18841
Tenacibaculum mesophilum DSM 13764
Polaribacter atrinae KACC 17473
Polaribacter butkevichii KCTC 12100
Polaribacter tangerinus WS2-14
Polaribacter haliotis RA4-7
Polaribacter irgensii 23-P
Polaribacter gangjinensis KCTC 22729
Polaribacter filamentus ATCC 700397
Polaribacter glomeratus ATCC 43844
Polaribacter porphyrae NBRC 108759
Polaribacter reichenbachii KCTC 23969
Polaribacter vadi LPB0003
Polaribacter dokdonensis DSW-5
Polaribacter dokdonensis DSM 17204
Flavobacterium ummariense DS-12
Flavobacterium marinum CGMCC 1.10825
Myroides guanonis DSM 26542
Myroides odoratus DSM 2801
Myroides indicus DSM 28213
Myroides injenensis M09-0166
Myroides phaeus DSM 23313
Myroides marinus DSM 23048
Myroides profundi DSM 19823
Myroides xuanwuensis DSM 27251
Myroides odoratimimus ATCC BAA-634
Flavobacterium indicum GPTSA100-9
Flavobacterium terrigena DSM 17934
Flavobacterium urocaniciphilum DSM 27078
Flavobacterium gelidilacus DSM 15343
Flavobacterium haoranii DSM 22807
Flavobacterium cucumis DSM 18830
Flavobacterium sasangense DSM 21067
Flavobacterium cheniae DSM 22462
Flavobacterium beibuense F44-8
Flavobacterium rivuli DSM 21788
Flavobacterium subsaxonicum DSM 21790
Flavobacterium akiainvivens IK-1
Flavobacterium akiainvivens DSM 25510
Flavobacterium suncheonense DSM 17707
Flavobacterium cauense NBRC 104929
Flavobacterium saliperosum S13
Flavobacterium enshiense KCTC 23775
Flavobacterium limnosediminis KACC 16937
Flavobacterium swingsii DSM 21789
Flavobacterium psychrophilum DSM 3660
Flavobacterium columnare ATCC 23463
Flavobacterium terrae DSM 18829
Flavobacterium filum DSM 17961
Flavobacterium aquatile LMG 4008
Flavobacterium dankookense DSM 25687
Flavobacterium aciduliphilum DSM 25663
Flavobacterium croceum DSM 17960
Flavobacterium fontis DSM 25660
Flavobacterium lindanitolerans DSM 21886
Flavobacterium endophyticum DSM 29537
Flavobacterium soli DSM 19725
Flavobacterium antarcticum DSM 19726
Flavobacterium tegetincola DSM 22377
Flavobacterium caeni CGMCC 1.7031
Flavobacterium noncentrifugens CGMCC 1.10076
Flavobacterium branchiophilum ATCC 35035
Flavobacterium fluvii DSM 19978
Flavobacterium gilvum EM1308
Flavobacterium aquicola DSM 100880
Flavobacterium succinicans DSM 4002
Flavobacterium flevense DSM 1076
Flavobacterium seoulense EM1321
Flavobacterium daejeonense DSM 17708
Flavobacterium glycines NBRC 105008
Flavobacterium frigidarium DSM 17623
Flavobacterium crassostreae LPB0076
Flavobacterium granuli DSM 19729
Flavobacterium granuli DSM 17797
Flavobacterium degerlachei DSM 15718
Flavobacterium frigoris DSM 15719
Flavobacterium gillisiae DSM 22376
Flavobacterium urumqiense CGMCC 1.9230
Flavobacterium xinjiangense CGMCC 1.2749
Flavobacterium weaverense DSM 19727
Flavobacterium segetis DSM 19741
Flavobacterium micromati DSM 17659
Flavobacterium xanthum DSM 3661
Flavobacterium fryxellicola DSM 16209
Flavobacterium fryxellicola DSM 16209

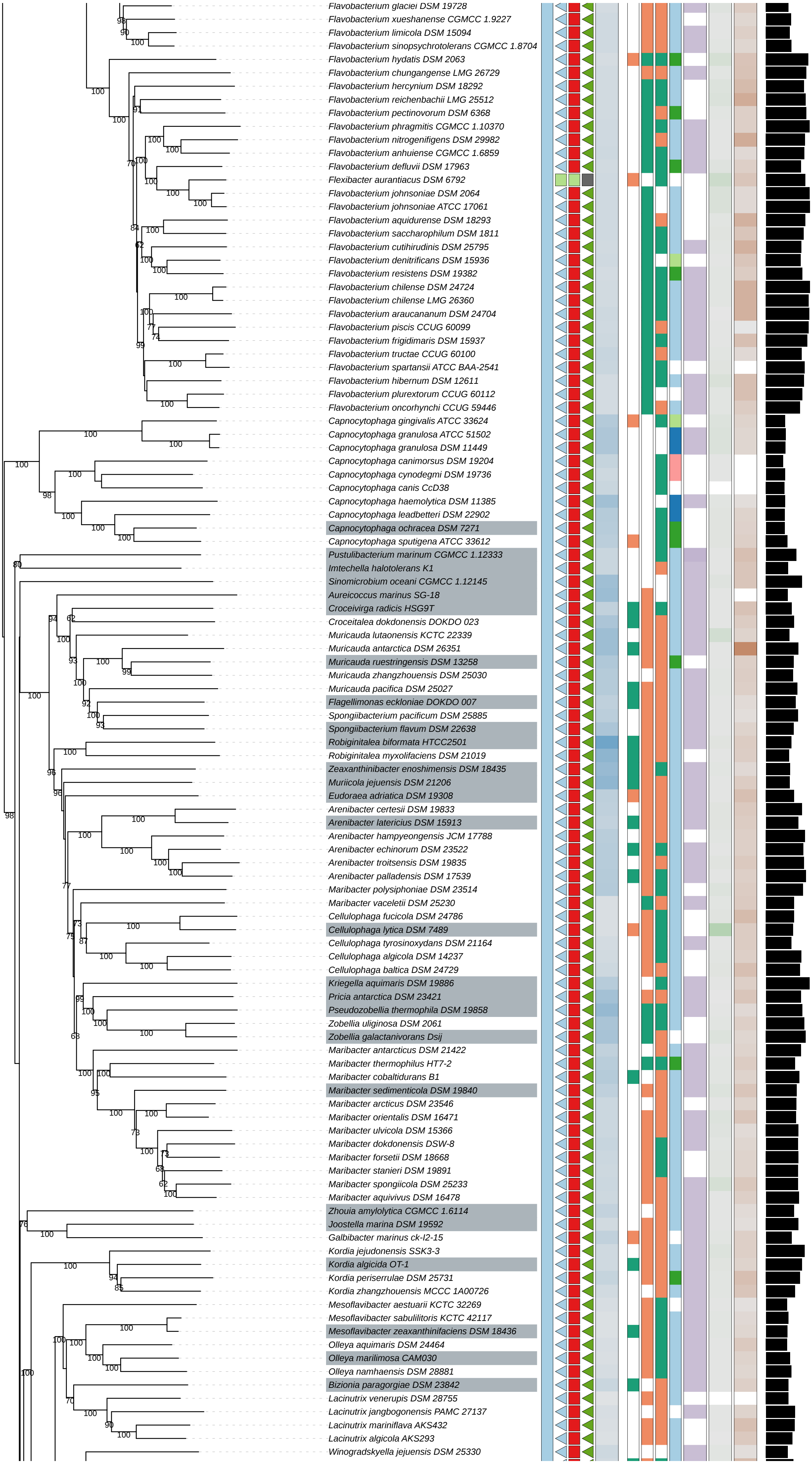

Flavobacterium glaciei DSM 19728
Flavobacterium xueshanense CGMCC 1.9227
Flavobacterium limicola DSM 15094
Flavobacterium sinopsychrotolerans CGMCC 1.8704
Flavobacterium hydatis DSM 2063
Flavobacterium chungangense LMG 26729
Flavobacterium hercynium DSM 18292
Flavobacterium reichenbachii LMG 25512
Flavobacterium pectinovorum DSM 6368
Flavobacterium phragmitis CGMCC 1.10370
Flavobacterium nitrogenifigens DSM 29982
Flavobacterium anhuiense CGMCC 1.6859
Flavobacterium defluvii DSM 17963
Flexibacter aurantiacus DSM 6792
Flavobacterium johnsoniae DSM 2064
Flavobacterium johnsoniae ATCC 17061
Flavobacterium aquidurense DSM 18293
Flavobacterium saccharophilum DSM 1811
Flavobacterium cutihirudinis DSM 25795
Flavobacterium denitrificans DSM 15936
Flavobacterium resistens DSM 19382
Flavobacterium chilense DSM 24724
Flavobacterium chilense LMG 26360
Flavobacterium araucananum DSM 24704
Flavobacterium piscis CCUG 60099
Flavobacterium frigidimaris DSM 15937
Flavobacterium tructae CCUG 60100
Flavobacterium spartansii ATCC BAA-2541
Flavobacterium hibernum DSM 12611
Flavobacterium plurextorum CCUG 60112
Flavobacterium oncorhynchi CCUG 59446
Capnocytophaga gingivalis ATCC 33624
Capnocytophaga granulosa ATCC 51502
Capnocytophaga granulosa DSM 11449
Capnocytophaga canimorsus DSM 19204
Capnocytophaga cynodegmi DSM 19736
Capnocytophaga canis CcD38
Capnocytophaga haemolytica DSM 11385
Capnocytophaga leadbetteri DSM 22902
Capnocytophaga ochracea DSM 7271
Capnocytophaga sputigena ATCC 33612
Pustulibacterium marinum CGMCC 1.12333
Imtechella halotolerans K1
Sinomicrobium oceani CGMCC 1.12145
Aureicoccus marinus SG-18
Croceivirga radicis HSG9T
Croceitalea dokdonensis DOKDO 023
Muricauda lutaonensis KCTC 22339
Muricauda antarctica DSM 26351
Muricauda ruestringensis DSM 13258
Muricauda zhangzhouensis DSM 25030
Muricauda pacifica DSM 25027
Flagellimonas eckloniae DOKDO 007
Spongiibacterium pacificum DSM 25885
Spongiibacterium flavum DSM 22638
Robiginitalea biformata HTCC2501
Robiginitalea myxolifaciens DSM 21019
Zeaxanthinibacter enoshimensis DSM 18435
Muriicola jejuensis DSM 21206
Eudoraea adriatica DSM 19308
Arenibacter certesii DSM 19833
Arenibacter latericius DSM 15913
Arenibacter hampyeongensis JCM 17788
Arenibacter echinorum DSM 23522
Arenibacter troitsensis DSM 19835
Arenibacter palladensis DSM 17539
Maribacter polysiphoniae DSM 23514
Maribacter vaceletii DSM 25230
Cellulophaga fucicola DSM 24786
Cellulophaga lytica DSM 7489
Cellulophaga tyrosinoxydans DSM 21164
Cellulophaga algicola DSM 14237
Cellulophaga baltica DSM 24729
Kriegella aquimaris DSM 19886
Pricia antarctica DSM 23421
Pseudozobellia thermophila DSM 19858
Zobellia uliginosa DSM 2061
Zobellia galactanivorans Dsij
Maribacter antarcticus DSM 21422
Maribacter thermophilus HT7-2
Maribacter cobaltidurans B1
Maribacter sedimenticola DSM 19840
Maribacter arcticus DSM 23546
Maribacter orientalis DSM 16471
Maribacter ulvicola DSM 15366
Maribacter dokdonensis DSW-8
Maribacter forsetii DSM 18668
Maribacter stanieri DSM 19891
Maribacter spongiicola DSM 25233
Maribacter aquivivus DSM 16478
Zhouia amylolytica CGMCC 1.6114
Joostella marina DSM 19592
Galbibacter marinus ck-I2-15
Kordia jejudonensis SSK3-3
Kordia algicida OT-1
Kordia periserrulae DSM 25731
Kordia zhangzhouensis MCCC 1A00726
Mesoflavibacter aestuarii KCTC 32269
Mesoflavibacter sabulilitoris KCTC 42117
Mesoflavibacter zeaxanthinifaciens DSM 18436
Olleya aquimaris DSM 24464
Olleya marilimosa CAM030
Olleya namhaensis DSM 28881
Bizionia paragorgiae DSM 23842
Lacinutrix venerupis DSM 28755
Lacinutrix jangbogonensis PAMC 27137
Lacinutrix mariniflava AKS432
Lacinutrix algicola AKS293
Winogradskyella jejuensis DSM 25330

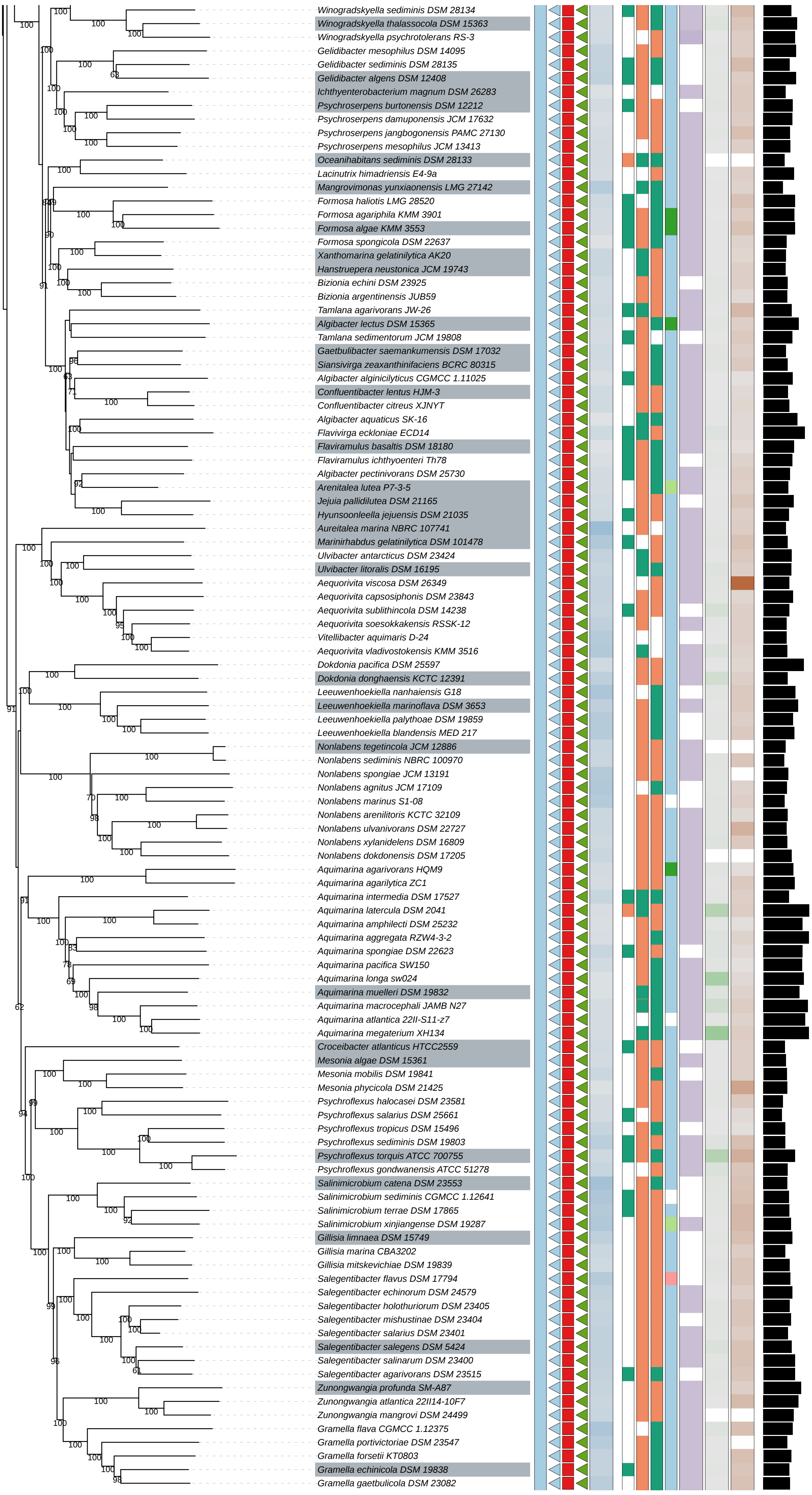
Winogradskyella sediminis DSM 28134
Winogradskyella thalassocola DSM 15363
Winogradskyella psychrotolerans RS-3
Gelidibacter mesophilus DSM 14095
Gelidibacter sediminis DSM 28135
Gelidibacter algens DSM 12408
Ichthyenterobacterium magnum DSM 26283
Psychroserpens burtonensis DSM 12212
Psychroserpens damuponensis JCM 17632
Psychroserpens jangbogonensis PAMC 27130
Psychroserpens mesophilus JCM 13413
Oceanihabitans sediminis DSM 28133
Lacinutrix himadriensis E4-9a
Mangrovimonas yunxiaonensis LMG 27142
Formosa haliotis LMG 28520
Formosa agariphila KMM 3901
Formosa algae KMM 3553
Formosa spongicola DSM 22637
Xanthomarina gelatinilytica AK20
Hanstruepera neustonica JCM 19743
Bizionia echini DSM 23925
Bizionia argentinensis JUB59
Tamlana agarivorans JW-26
Algibacter lectus DSM 15365
Tamlana sedimentorum JCM 19808
Gaetbulibacter saemankumensis DSM 17032
Siansivirga zeaxanthinifaciens BCRC 80315
Algibacter alginicilyticus CGMCC 1.11025
Confluentibacter lentus HJM-3
Confluentibacter citreus XJNYT
Algibacter aquaticus SK-16
Flavivirga eckloniae ECD14
Flaviramulus basaltis DSM 18180
Flaviramulus ichthyoenteri Th78
Algibacter pectinivorans DSM 25730
Arenitalea lutea P7-3-5
Jejuia pallidilutea DSM 21165
Hyunsoonleella jejuensis DSM 21035
Aureitalea marina NBRC 107741
Marinirhabdus gelatinilytica DSM 101478
Ulvibacter antarcticus DSM 23424
Ulvibacter litoralis DSM 16195
Aequorivita viscosa DSM 26349
Aequorivita capsosiphonis DSM 23843
Aequorivita sublithincola DSM 14238
Aequorivita soesokkakensis RSSK-12
Vitellibacter aquimaris D-24
Aequorivita vladivostokensis KMM 3516
Dokdonia pacifica DSM 25597
Dokdonia donghaensis KCTC 12391
Leeuwenhoekiella nanhaiensis G18
Leeuwenhoekiella marinoflava DSM 3653
Leeuwenhoekiella palythoae DSM 19859
Leeuwenhoekiella blandensis MED 217
Nonlabens tegetincola JCM 12886
Nonlabens sediminis NBRC 100970
Nonlabens spongiae JCM 13191
Nonlabens agnitus JCM 17109
Nonlabens marinus S1-08
Nonlabens arenilitoris KCTC 32109
Nonlabens ulvanivorans DSM 22727
Nonlabens xylanidelens DSM 16809
Nonlabens dokdonensis DSM 17205
Aquimarina agarivorans HQM9
Aquimarina agarilytica ZC1
Aquimarina intermedia DSM 17527
Aquimarina latercula DSM 2041
Aquimarina amphilecti DSM 25232
Aquimarina aggregata RZW4-3-2
Aquimarina spongiae DSM 22623
Aquimarina pacifica SW150
Aquimarina longa sw024
Aquimarina muelleri DSM 19832
Aquimarina macrocephali JAMB N27
Aquimarina atlantica 22II-S11-z7
Aquimarina megaterium XH134
Croceibacter atlanticus HTCC2559
Mesonia algae DSM 15361
Mesonia mobilis DSM 19841
Mesonia phycicola DSM 21425
Psychroflexus halocasei DSM 23581
Psychroflexus salarius DSM 25661
Psychroflexus tropicus DSM 15496
Psychroflexus sediminis DSM 19803
Psychroflexus torquis ATCC 700755
Psychroflexus gondwanensis ATCC 51278
Salinimicrobium catena DSM 23553
Salinimicrobium sediminis CGMCC 1.12641
Salinimicrobium terrae DSM 17865
Salinimicrobium xinjiangense DSM 19287
Gillisia limnaea DSM 15749
Gillisia marina CBA3202
Gillisia mitskevichiae DSM 19839
Salegentibacter flavus DSM 17794
Salegentibacter echinorum DSM 24579
Salegentibacter holothuriorum DSM 23405
Salegentibacter mishustinae DSM 23404
Salegentibacter salarius DSM 23401
Salegentibacter salegens DSM 5424
Salegentibacter salinarum DSM 23400
Salegentibacter agarivorans DSM 23515
Zunongwangia profunda SM-A87
Zunongwangia atlantica 22II14-10F7
Zunongwangia mangrovi DSM 24499
Gramella flava CGMCC 1.12375
Gramella portivictoriae DSM 23547
Gramella forsetii KT0803
Gramella echinicola DSM 19838
Gramella gaetbulicola DSM 23082

Figure 1: The figure shows the complete, uncollapsed GBDP tree of the *Bacteroidetes* genome dataset, which had to be distributed across Figures 1-8 in the main manuscript. Tree inferred with FastME from GBDP distances calculated from whole proteomes. The branches are scaled in terms of log-transformed intergenomic distances (GBDP formula d_5). The numbers above branches are GBDP pseudo-bootstrap support values from 100 replications. Tip colors indicate type species, colors to the right of the tips indicate, from left to right, phylum (1), class (2), order (3) and family (4). The blue gradient scale (5) indicates the exact G+C content as calculated from the genome sequences. The block labelled as "phenotype" displays phenotypic information (6-12), whereas genome size (13) is displayed at the right-hand side. See the embedded legend for details.

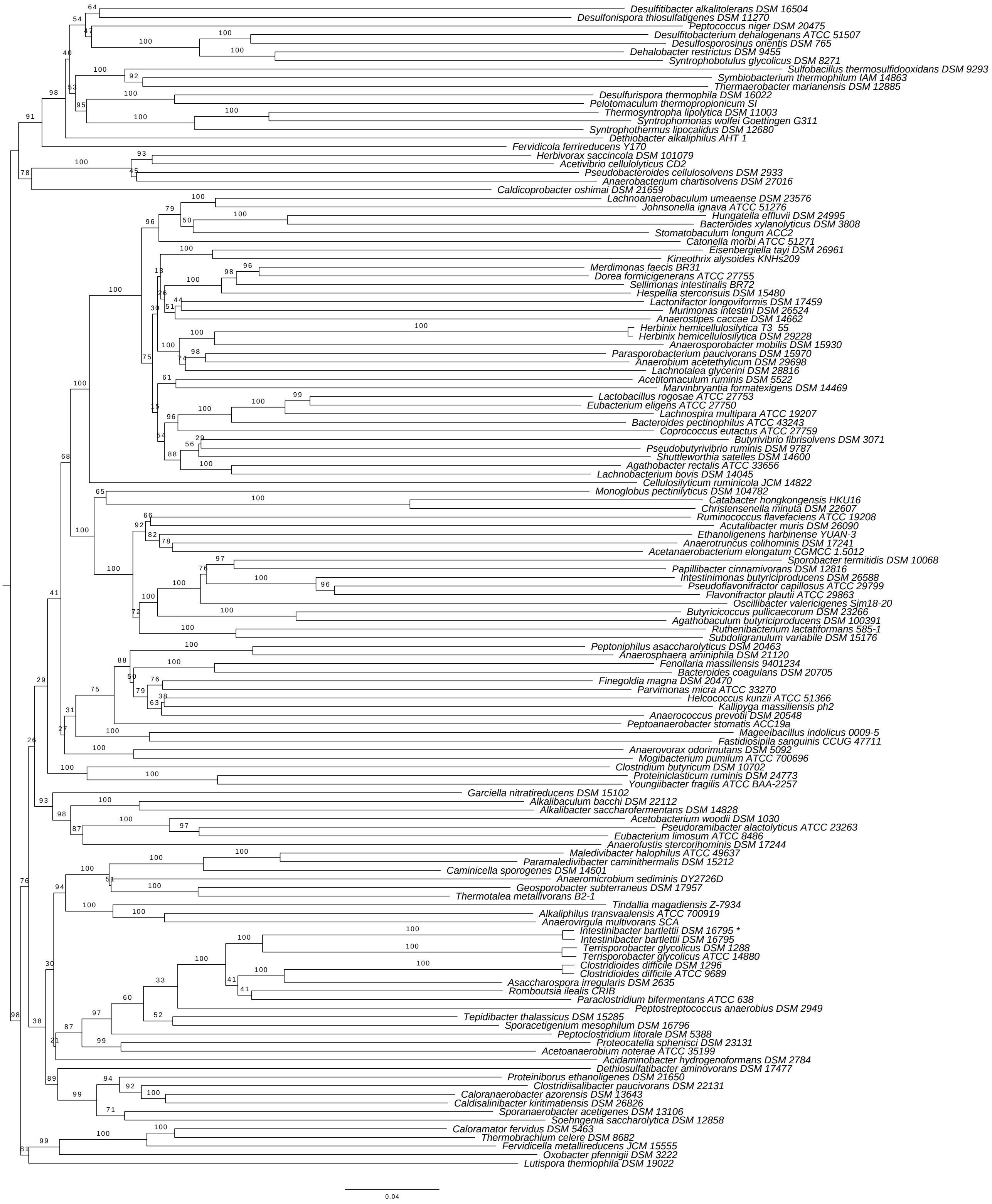

Figure 2: Phylogenomic tree of selected *Eubacteriales* inferred with FastME from GBDP distances calculated from whole proteomes. The branches are scaled in terms of log-transformed intergenomic distances (GBDP formula d_5). The numbers above branches are GBDP pseudo-bootstrap support values from 100 replications.

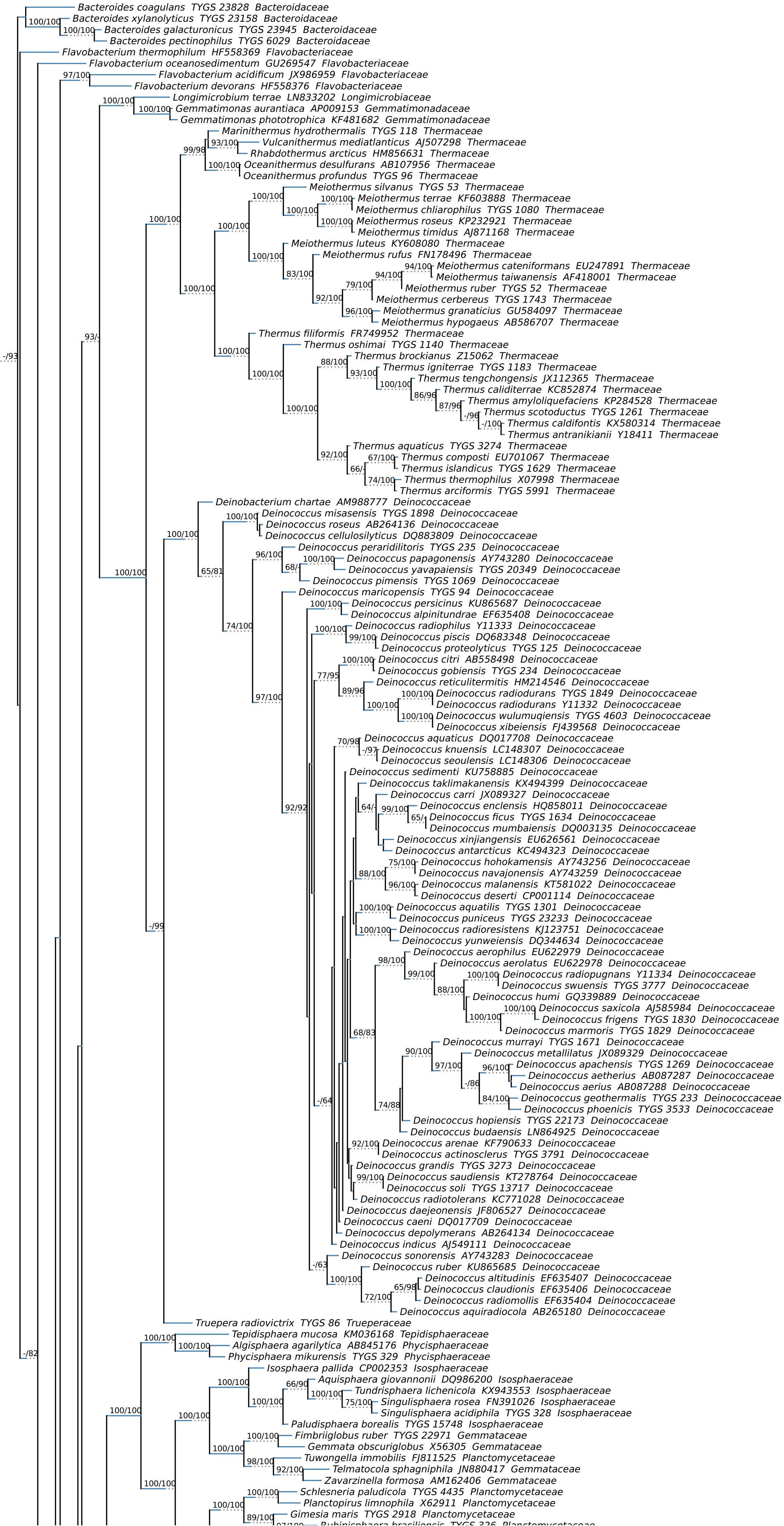
Bacteroides coagulans TYGS 23828 Bacteroidaceae
Bacteroides xylanolyticus TYGS 23158 Bacteroidaceae
Bacteroides galacturonicus TYGS 23945 Bacteroidaceae
Bacteroides pectinophilus TYGS 6029 Bacteroidaceae
Flavobacterium thermophilum HF558369 Flavobacteriaceae
Flavobacterium oceanosedimentum GU269547 Flavobacteriaceae
Flavobacterium acidificum JX986959 Flavobacteriaceae
Flavobacterium devorans HF558376 Flavobacteriaceae
Longimicrobium terrae LN833202 Longimicrobiaceae
Gemmatimonas aurantiaca AP009153 Gemmatimonadaceae
Gemmatimonas phototrophica KF481682 Gemmatimonadaceae
Marinithermus hydrothermalis TYGS 118 Thermaceae
Vulcanithermus mediatlanticus AJ507298 Thermaceae
Rhabdothermus arcticus HM856631 Thermaceae
Oceanithermus desulfurans AB107956 Thermaceae
Oceanithermus profundus TYGS 96 Thermaceae
Meiothermus silvanus TYGS 53 Thermaceae
Meiothermus terrae KF603888 Thermaceae
Meiothermus chliarophilus TYGS 1080 Thermaceae
Meiothermus roseus KP232921 Thermaceae
Meiothermus timidus AJ871168 Thermaceae
Meiothermus luteus KY608080 Thermaceae
Meiothermus rufus FN178496 Thermaceae
Meiothermus cateniformans EU247891 Thermaceae
Meiothermus taiwanensis AF418001 Thermaceae
Meiothermus ruber TYGS 52 Thermaceae
Meiothermus cerbereus TYGS 1743 Thermaceae
Meiothermus granaticius GU584097 Thermaceae
Meiothermus hypogaeus AB586707 Thermaceae
Thermus filiformis FR749952 Thermaceae
Thermus oshimai TYGS 1140 Thermaceae
Thermus brockianus Z15062 Thermaceae
Thermus igniterrae TYGS 1183 Thermaceae
Thermus tengchongensis JX112365 Thermaceae
Thermus caliditerrae KC852874 Thermaceae
Thermus amyloliquefaciens KP284528 Thermaceae
Thermus scotoductus TYGS 1261 Thermaceae
Thermus caldifontis KX580314 Thermaceae
Thermus antranikianii Y18411 Thermaceae
Thermus aquaticus TYGS 3274 Thermaceae
Thermus composti EU701067 Thermaceae
Thermus islandicus TYGS 1629 Thermaceae
Thermus thermophilus X07998 Thermaceae
Thermus arciformis TYGS 5991 Thermaceae
Deinobacterium chartae AM988777 Deinococcaceae
Deinococcus misasensis TYGS 1898 Deinococcaceae
Deinococcus roseus AB264136 Deinococcaceae
Deinococcus cellulosilyticus DQ883809 Deinococcaceae
Deinococcus peraridilitoris TYGS 235 Deinococcaceae
Deinococcus papagonensis AY743280 Deinococcaceae
Deinococcus yavapaiensis TYGS 20349 Deinococcaceae
Deinococcus pimensis TYGS 1069 Deinococcaceae
Deinococcus maricopensis TYGS 94 Deinococcaceae
Deinococcus persicinus KU865687 Deinococcaceae
Deinococcus alpinitundrae EF635408 Deinococcaceae
Deinococcus radiophilus Y11333 Deinococcaceae
Deinococcus piscis DQ683348 Deinococcaceae
Deinococcus proteolyticus TYGS 125 Deinococcaceae
Deinococcus citri AB558498 Deinococcaceae
Deinococcus gobiensis TYGS 234 Deinococcaceae
Deinococcus reticulitermitis HM214546 Deinococcaceae
Deinococcus radiodurans TYGS 1849 Deinococcaceae
Deinococcus radiodurans Y11332 Deinococcaceae
Deinococcus wulumuqiensis TYGS 4603 Deinococcaceae
Deinococcus xibeiensis FJ439568 Deinococcaceae
Deinococcus aquaticus DQ017708 Deinococcaceae
Deinococcus knuensis LC148307 Deinococcaceae
Deinococcus seoulensis LC148306 Deinococcaceae
Deinococcus sedimenti KU758885 Deinococcaceae
Deinococcus taklimakanensis KX494399 Deinococcaceae
Deinococcus carri JX089327 Deinococcaceae
Deinococcus enclensis HQ858011 Deinococcaceae
Deinococcus ficus TYGS 1634 Deinococcaceae
Deinococcus mumbaiensis DQ003135 Deinococcaceae
Deinococcus xinjiangensis EU626561 Deinococcaceae
Deinococcus antarcticus KC494323 Deinococcaceae
Deinococcus hohokamensis AY743256 Deinococcaceae
Deinococcus navajonensis AY743259 Deinococcaceae
Deinococcus malanensis KT581022 Deinococcaceae
Deinococcus deserti CP001114 Deinococcaceae
Deinococcus aquatilis TYGS 1301 Deinococcaceae
Deinococcus puniceus TYGS 23233 Deinococcaceae
Deinococcus radioresistens KJ123751 Deinococcaceae
Deinococcus yunweiensis DQ344634 Deinococcaceae
Deinococcus aerophilus EU622979 Deinococcaceae
Deinococcus aerolatus EU622978 Deinococcaceae
Deinococcus radiopugnans Y11334 Deinococcaceae
Deinococcus swuensis TYGS 3777 Deinococcaceae
Deinococcus humi GQ339889 Deinococcaceae
Deinococcus saxicola AJ585984 Deinococcaceae
Deinococcus frigens TYGS 1830 Deinococcaceae
Deinococcus marmoris TYGS 1829 Deinococcaceae
Deinococcus murrayi TYGS 1671 Deinococcaceae
Deinococcus metallilatus JX089329 Deinococcaceae
Deinococcus apachensis TYGS 1269 Deinococcaceae
Deinococcus aetherius AB087287 Deinococcaceae
Deinococcus aerius AB087288 Deinococcaceae
Deinococcus geothermalis TYGS 233 Deinococcaceae
Deinococcus phoenicis TYGS 3533 Deinococcaceae
Deinococcus hopiensis TYGS 22173 Deinococcaceae
Deinococcus budaensis LN864925 Deinococcaceae
Deinococcus arenae KF790633 Deinococcaceae
Deinococcus actinosclerus TYGS 3791 Deinococcaceae
Deinococcus grandis TYGS 3273 Deinococcaceae
Deinococcus saudiensis KT278764 Deinococcaceae
Deinococcus soli TYGS 13717 Deinococcaceae
Deinococcus radiotolerans KC771028 Deinococcaceae
Deinococcus daejeonensis JF806527 Deinococcaceae
Deinococcus caeni DQ017709 Deinococcaceae
Deinococcus depolymerans AB264134 Deinococcaceae
Deinococcus indicus AJ549111 Deinococcaceae
Deinococcus sonorensis AY743283 Deinococcaceae
Deinococcus ruber KU865685 Deinococcaceae
Deinococcus altitudinis EF635407 Deinococcaceae
Deinococcus claudionis EF635406 Deinococcaceae
Deinococcus radiomollis EF635404 Deinococcaceae
Deinococcus aquiradiocola AB265180 Deinococcaceae
Truepera radiovictrix TYGS 86 Trueperaceae
Tepidisphaera mucosa KM036168 Tepidisphaeraceae
Algisphaera agarilytica AB845176 Phycisphaeraceae
Phycisphaera mikurensis TYGS 329 Phycisphaeraceae
Isosphaera pallida CP002353 Isosphaeraceae
Aquisphaera giovannonii DQ986200 Isosphaeraceae
Tundrisphaera lichenicola KX943553 Isosphaeraceae
Singulisphaera rosea FN391026 Isosphaeraceae
Singulisphaera acidiphila TYGS 328 Isosphaeraceae
Paludisphaera borealis TYGS 15748 Isosphaeraceae
Fimbriiglobus ruber TYGS 22971 Gemmataceae
Gemmata obscuriglobus X56305 Gemmataceae
Tuwongella immobilis FJ811525 Planctomycetaceae
Telmatocola sphagniphila JN880417 Gemmataceae
Zavarzinella formosa AM162406 Gemmataceae
Schlesneria paludicola TYGS 4435 Planctomycetaceae
Planctopirus limnophila X62911 Planctomycetaceae
Gimesia maris TYGS 2918 Planctomycetaceae

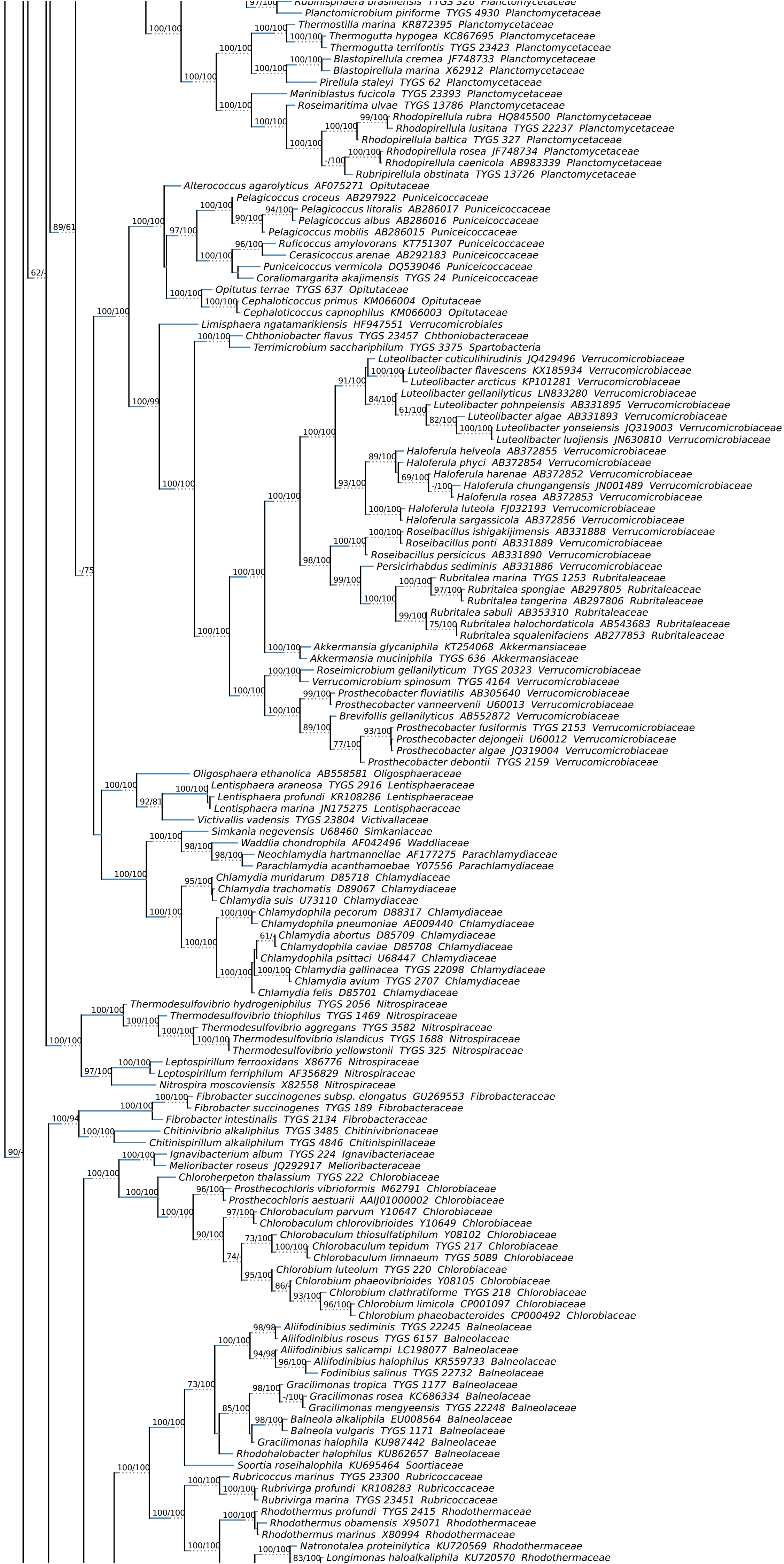

Planctomicrobium piriforme TYGS 4930 Planctomycetaceae
Thermostilla marina KR872395 Planctomycetaceae
Thermogutta hypogea KC867695 Planctomycetaceae
Thermogutta terrifontis TYGS 23423 Planctomycetaceae
Blastopirellula cremea JF748733 Planctomycetaceae
Blastopirellula marina X62912 Planctomycetaceae
Pirellula staleyi TYGS 62 Planctomycetaceae
Mariniblastus fucicola TYGS 23393 Planctomycetaceae
Roseimaritima ulvae TYGS 13786 Planctomycetaceae
Rhodopirellula rubra HQ845500 Planctomycetaceae
Rhodopirellula lusitana TYGS 22237 Planctomycetaceae
Rhodopirellula baltica TYGS 327 Planctomycetaceae
Rhodopirellula rosea JF748734 Planctomycetaceae
Rhodopirellula caenicola AB983339 Planctomycetaceae
Rubripirellula obstinata TYGS 13726 Planctomycetaceae
Alterococcus agarolyticus AF075271 Opitutaceae
Pelagicoccus croceus AB297922 Puniceicoccaceae
Pelagicoccus litoralis AB286017 Puniceicoccaceae
Pelagicoccus albus AB286016 Puniceicoccaceae
Pelagicoccus mobilis AB286015 Puniceicoccaceae
Ruficoccus amylovorans KT751307 Puniceicoccaceae
Cerasicoccus arenae AB292183 Puniceicoccaceae
Puniceicoccus vermicola DQ539046 Puniceicoccaceae
Coraliomargarita akajimensis TYGS 24 Puniceicoccaceae
Opitutus terrae TYGS 637 Opitutaceae
Cephaloticoccus primus KM066004 Opitutaceae
Cephaloticoccus capnophilus KM066003 Opitutaceae
Limisphaera ngatamarikiensis HF947551 Verrucomicrobiales
Chthoniobacter flavus TYGS 23457 Chthoniobacteraceae
Terrimicrobium sacchariphilum TYGS 3375 Spartobacteria
Luteolibacter cuticulihirudinis JQ429496 Verrucomicrobiaceae
Luteolibacter flavescens KX185934 Verrucomicrobiaceae
Luteolibacter arcticus KP101281 Verrucomicrobiaceae
Luteolibacter gellanilyticus LN833280 Verrucomicrobiaceae
Luteolibacter pohnpeiensis AB331895 Verrucomicrobiaceae
Luteolibacter algae AB331893 Verrucomicrobiaceae
Luteolibacter yonseiensis JQ319003 Verrucomicrobiaceae
Luteolibacter luojiensis JN630810 Verrucomicrobiaceae
Haloferula helveola AB372855 Verrucomicrobiaceae
Haloferula phyci AB372854 Verrucomicrobiaceae
Haloferula harenae AB372852 Verrucomicrobiaceae
Haloferula chungangensis JN001489 Verrucomicrobiaceae
Haloferula rosea AB372853 Verrucomicrobiaceae
Haloferula luteola FJ032193 Verrucomicrobiaceae
Haloferula sargassicola AB372856 Verrucomicrobiaceae
Roseibacillus ishigakijimensis AB331888 Verrucomicrobiaceae
Roseibacillus ponti AB331889 Verrucomicrobiaceae
Roseibacillus persicicus AB331890 Verrucomicrobiaceae
Persicirhabdus sediminis AB331886 Verrucomicrobiaceae
Rubritalea marina TYGS 1253 Rubritaleaceae
Rubritalea spongiae AB297805 Rubritaleaceae
Rubritalea tangerina AB297806 Rubritaleaceae
Rubritalea sabuli AB353310 Rubritaleaceae
Rubritalea halochordaticola AB543683 Rubritaleaceae
Rubritalea squalenifaciens AB277853 Rubritaleaceae
Akkermansia glycaniphila KT254068 Akkermansiaceae
Akkermansia muciniphila TYGS 636 Akkermansiaceae
Roseimicrobium gellanilyticum TYGS 20323 Verrucomicrobiaceae
Verrucomicrobium spinosum TYGS 4164 Verrucomicrobiaceae
Prosthecobacter fluviatilis AB305640 Verrucomicrobiaceae
Prosthecobacter vanneervenii U60013 Verrucomicrobiaceae
Brevifollis gellanilyticus AB552872 Verrucomicrobiaceae
Prosthecobacter fusiformis TYGS 2153 Verrucomicrobiaceae
Prosthecobacter dejongeii U60012 Verrucomicrobiaceae
Prosthecobacter algae JQ319004 Verrucomicrobiaceae
Prosthecobacter debontii TYGS 2159 Verrucomicrobiaceae
Oligosphaera ethanolica AB558581 Oligosphaeraceae
Lentisphaera araneosa TYGS 2916 Lentisphaeraceae
Lentisphaera profundi KR108286 Lentisphaeraceae
Lentisphaera marina JN175275 Lentisphaeraceae
Victivallis vadensis TYGS 23804 Victivallaceae
Simkania negevensis U68460 Simkaniaceae
Waddlia chondrophila AF042496 Waddliaceae
Neochlamydia hartmannellae AF177275 Parachlamydiaceae
Parachlamydia acanthamoebae Y07556 Parachlamydiaceae
Chlamydia muridarum D85718 Chlamydiaceae
Chlamydia trachomatis D89067 Chlamydiaceae
Chlamydia suis U73110 Chlamydiaceae
Chlamydophila pecorum D88317 Chlamydiaceae
Chlamydophila pneumoniae AE009440 Chlamydiaceae
Chlamydia abortus D85709 Chlamydiaceae
Chlamydophila caviae D85708 Chlamydiaceae
Chlamydophila psittaci U68447 Chlamydiaceae
Chlamydia gallinacea TYGS 22098 Chlamydiaceae
Chlamydia avium TYGS 2707 Chlamydiaceae
Chlamydia felis D85701 Chlamydiaceae
Thermodesulfovibrio hydrogeniphilus TYGS 2056 Nitrospiraceae
Thermodesulfovibrio thiophilus TYGS 1469 Nitrospiraceae
Thermodesulfovibrio aggregans TYGS 3582 Nitrospiraceae
Thermodesulfovibrio islandicus TYGS 1688 Nitrospiraceae
Thermodesulfovibrio yellowstonii TYGS 325 Nitrospiraceae
Leptospirillum ferrooxidans X86776 Nitrospiraceae
Leptospirillum ferriphilum AF356829 Nitrospiraceae
Nitrospira moscoviensis X82558 Nitrospiraceae
Fibrobacter succinogenes subsp. elongatus GU269553 Fibrobacteraceae
Fibrobacter succinogenes TYGS 189 Fibrobacteraceae
Fibrobacter intestinalis TYGS 2134 Fibrobacteraceae
Chitinivibrio alkaliphilus TYGS 3485 Chitinivibrionaceae
Chitinispirillum alkaliphilum TYGS 4846 Chitinispirillaceae
Ignavibacterium album TYGS 224 Ignavibacteriaceae
Melioribacter roseus JQ292917 Melioribacteraceae
Chloroherpeton thalassium TYGS 222 Chlorobiaceae
Prosthecochloris vibrioformis M62791 Chlorobiaceae
Prosthecochloris aestuarii AAIJ01000002 Chlorobiaceae
Chlorobaculum parvum Y10647 Chlorobiaceae
Chlorobaculum chlorovibrioides Y10649 Chlorobiaceae
Chlorobaculum thiosulfatiphilum Y08102 Chlorobiaceae
Chlorobaculum tepidum TYGS 217 Chlorobiaceae
Chlorobaculum limnaeum TYGS 5089 Chlorobiaceae
Chlorobium luteolum TYGS 220 Chlorobiaceae
Chlorobium phaeovibrioides Y08105 Chlorobiaceae
Chlorobium clathratiforme TYGS 218 Chlorobiaceae
Chlorobium limicola CP001097 Chlorobiaceae
Chlorobium phaeobacteroides CP000492 Chlorobiaceae
Aliifodinibius sediminis TYGS 22245 Balneolaceae
Aliifodinibius roseus TYGS 6157 Balneolaceae
Aliifodinibius salicampi LC198077 Balneolaceae
Aliifodinibius halophilus KR559733 Balneolaceae
Fodinibius salinus TYGS 22732 Balneolaceae
Gracilimonas tropica TYGS 1177 Balneolaceae
Gracilimonas rosea KC686334 Balneolaceae
Gracilimonas mengyeensis TYGS 22248 Balneolaceae
Balneola alkaliphila EU008564 Balneolaceae
Balneola vulgaris TYGS 1171 Balneolaceae
Gracilimonas halophila KU987442 Balneolaceae
Rhodohalobacter halophilus KU862657 Balneolaceae
Soortia roseihalophila KU695464 Soortiaceae
Rubricoccus marinus TYGS 23300 Rubricoccaceae
Rubrivirga profundi KR108283 Rubricoccaceae
Rubrivirga marina TYGS 23451 Rubricoccaceae
Rhodothermus profundi TYGS 2415 Rhodothermaceae
Rhodothermus obamensis X95071 Rhodothermaceae
Rhodothermus marinus X80994 Rhodothermaceae
Natronotalea proteinilytica KU720569 Rhodothermaceae
Longimonas haloalkaliphila KU720570 Rhodothermaceae

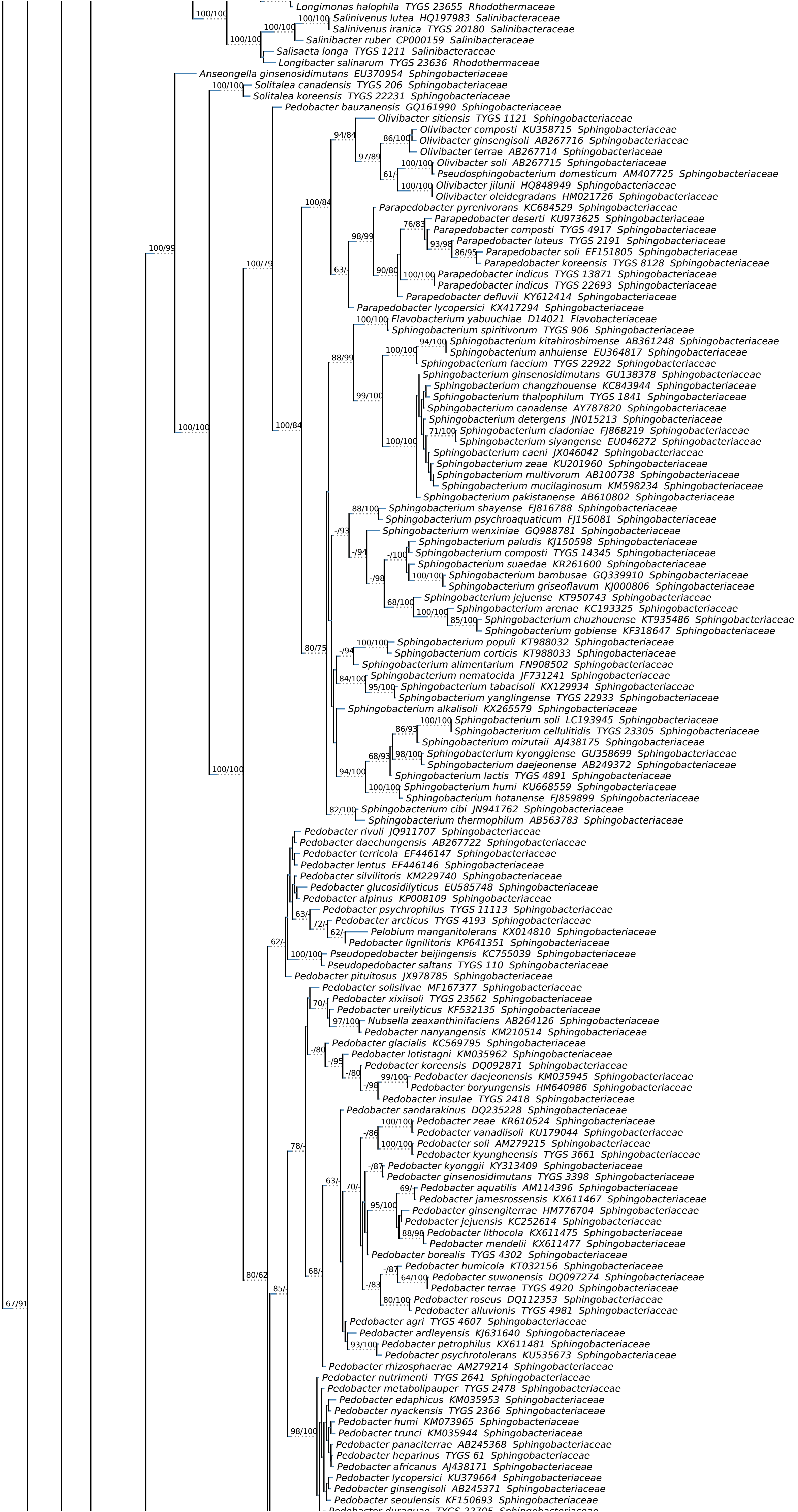

Longimonas halophila TYGS 23655 Rhodothermaceae
Salinivenus lutea HQ197983 Salinibacteraceae
Salinivenus iranica TYGS 20180 Salinibacteraceae
Salinibacter ruber CP000159 Salinibacteraceae
Salisaeta longa TYGS 1211 Salinibacteraceae
Longibacter salinarum TYGS 23636 Rhodothermaceae
Anseongella ginsenosidimutans EU370954 Sphingobacteriaceae
Solitalea canadensis TYGS 206 Sphingobacteriaceae
Solitalea koreensis TYGS 22231 Sphingobacteriaceae
Pedobacter bauzanensis GQ161990 Sphingobacteriaceae
Olivibacter sitiensis TYGS 1121 Sphingobacteriaceae
Olivibacter composti KU358715 Sphingobacteriaceae
Olivibacter ginsengisoli AB267716 Sphingobacteriaceae
Olivibacter terrae AB267714 Sphingobacteriaceae
Olivibacter soli AB267715 Sphingobacteriaceae
Pseudosphingobacterium domesticum AM407725 Sphingobacteriaceae
Olivibacter jilunii HQ848949 Sphingobacteriaceae
Olivibacter oleidegradans HM021726 Sphingobacteriaceae
Parapedobacter pyrenivorans KC684529 Sphingobacteriaceae
Parapedobacter deserti KU973625 Sphingobacteriaceae
Parapedobacter composti TYGS 4917 Sphingobacteriaceae
Parapedobacter luteus TYGS 2191 Sphingobacteriaceae
Parapedobacter soli EF151805 Sphingobacteriaceae
Parapedobacter koreensis TYGS 8128 Sphingobacteriaceae
Parapedobacter indicus TYGS 13871 Sphingobacteriaceae
Parapedobacter indicus TYGS 22693 Sphingobacteriaceae
Parapedobacter defluvii KY612414 Sphingobacteriaceae
Parapedobacter lycopersici KX417294 Sphingobacteriaceae
Flavobacterium yabuuchiae D14021 Flavobacteriaceae
Sphingobacterium spiritivorum TYGS 906 Sphingobacteriaceae
Sphingobacterium kitahiroshimense AB361248 Sphingobacteriaceae
Sphingobacterium anhuiense EU364817 Sphingobacteriaceae
Sphingobacterium faecium TYGS 22922 Sphingobacteriaceae
Sphingobacterium ginsenosidimutans GU138378 Sphingobacteriaceae
Sphingobacterium changzhouense KC843944 Sphingobacteriaceae
Sphingobacterium thalpophilum TYGS 1841 Sphingobacteriaceae
Sphingobacterium canadense AY787820 Sphingobacteriaceae
Sphingobacterium detergens JN015213 Sphingobacteriaceae
Sphingobacterium cladoniae FJ868219 Sphingobacteriaceae
Sphingobacterium siyangense EU046272 Sphingobacteriaceae
Sphingobacterium caeni JX046042 Sphingobacteriaceae
Sphingobacterium zeae KU201960 Sphingobacteriaceae
Sphingobacterium multivorum AB100738 Sphingobacteriaceae
Sphingobacterium mucilaginosum KM598234 Sphingobacteriaceae
Sphingobacterium pakistanense AB610802 Sphingobacteriaceae
Sphingobacterium shayense FJ816788 Sphingobacteriaceae
Sphingobacterium psychroaquaticum FJ156081 Sphingobacteriaceae
Sphingobacterium wenxiniae GQ988781 Sphingobacteriaceae
Sphingobacterium paludis KJ150598 Sphingobacteriaceae
Sphingobacterium composti TYGS 14345 Sphingobacteriaceae
Sphingobacterium suaedae KR261600 Sphingobacteriaceae
Sphingobacterium bambusae GQ339910 Sphingobacteriaceae
Sphingobacterium griseoflavum KJ000806 Sphingobacteriaceae
Sphingobacterium jejuense KT950743 Sphingobacteriaceae
Sphingobacterium arenae KC193325 Sphingobacteriaceae
Sphingobacterium chuzhouense KT935486 Sphingobacteriaceae
Sphingobacterium gobiense KF318647 Sphingobacteriaceae
Sphingobacterium populi KT988032 Sphingobacteriaceae
Sphingobacterium corticis KT988033 Sphingobacteriaceae
Sphingobacterium alimentarium FN908502 Sphingobacteriaceae
Sphingobacterium nematocida JF731241 Sphingobacteriaceae
Sphingobacterium tabacisoli KX129934 Sphingobacteriaceae
Sphingobacterium yanglingense TYGS 22933 Sphingobacteriaceae
Sphingobacterium alkalisoli KX265579 Sphingobacteriaceae
Sphingobacterium soli LC193945 Sphingobacteriaceae
Sphingobacterium cellulitidis TYGS 23305 Sphingobacteriaceae
Sphingobacterium mizutaii AJ438175 Sphingobacteriaceae
Sphingobacterium kyonggiense GU358699 Sphingobacteriaceae
Sphingobacterium daejeonense AB249372 Sphingobacteriaceae
Sphingobacterium lactis TYGS 4891 Sphingobacteriaceae
Sphingobacterium humi KU668559 Sphingobacteriaceae
Sphingobacterium hotanense FJ859899 Sphingobacteriaceae
Sphingobacterium cibi JN941762 Sphingobacteriaceae
Sphingobacterium thermophilum AB563783 Sphingobacteriaceae
Pedobacter rivuli JQ911707 Sphingobacteriaceae
Pedobacter daechungensis AB267722 Sphingobacteriaceae
Pedobacter terricola EF446147 Sphingobacteriaceae
Pedobacter lentus EF446146 Sphingobacteriaceae
Pedobacter silvilitoris KM229740 Sphingobacteriaceae
Pedobacter glucosidilyticus EU585748 Sphingobacteriaceae
Pedobacter alpinus KP008109 Sphingobacteriaceae
Pedobacter psychrophilus TYGS 11113 Sphingobacteriaceae
Pedobacter arcticus TYGS 4193 Sphingobacteriaceae
Pelobium manganitolerans KX014810 Sphingobacteriaceae
Pedobacter lignilitoris KP641351 Sphingobacteriaceae
Pseudopedobacter beijingensis KC755039 Sphingobacteriaceae
Pseudopedobacter saltans TYGS 110 Sphingobacteriaceae
Pedobacter pituitosus JX978785 Sphingobacteriaceae
Pedobacter solisilvae MF167377 Sphingobacteriaceae
Pedobacter xixiisoli TYGS 23562 Sphingobacteriaceae
Pedobacter ureilyticus KF532135 Sphingobacteriaceae
Nubsella zeaxanthinifaciens AB264126 Sphingobacteriaceae
Pedobacter nanyangensis KM210514 Sphingobacteriaceae
Pedobacter glacialis KC569795 Sphingobacteriaceae
Pedobacter lotistagni KM035962 Sphingobacteriaceae
Pedobacter koreensis DQ092871 Sphingobacteriaceae
Pedobacter daejeonensis KM035945 Sphingobacteriaceae
Pedobacter boryungensis HM640986 Sphingobacteriaceae
Pedobacter insulae TYGS 2418 Sphingobacteriaceae
Pedobacter sandarakinus DQ235228 Sphingobacteriaceae
Pedobacter zeae KR610524 Sphingobacteriaceae
Pedobacter vanadiisoli KU179044 Sphingobacteriaceae
Pedobacter soli AM279215 Sphingobacteriaceae
Pedobacter kyungheensis TYGS 3661 Sphingobacteriaceae
Pedobacter kyonggii KY313409 Sphingobacteriaceae
Pedobacter ginsenosidimutans TYGS 3398 Sphingobacteriaceae
Pedobacter aquatilis AM114396 Sphingobacteriaceae
Pedobacter jamesrossensis KX611467 Sphingobacteriaceae
Pedobacter ginsengiterrae HM776704 Sphingobacteriaceae
Pedobacter jejuensis KC252614 Sphingobacteriaceae
Pedobacter lithocola KX611475 Sphingobacteriaceae
Pedobacter mendelii KX611477 Sphingobacteriaceae
Pedobacter borealis TYGS 4302 Sphingobacteriaceae
Pedobacter humicola KT032156 Sphingobacteriaceae
Pedobacter suwonensis DQ097274 Sphingobacteriaceae
Pedobacter terrae TYGS 4920 Sphingobacteriaceae
Pedobacter roseus DQ112353 Sphingobacteriaceae
Pedobacter alluvionis TYGS 4981 Sphingobacteriaceae
Pedobacter agri TYGS 4607 Sphingobacteriaceae
Pedobacter ardleyensis KJ631640 Sphingobacteriaceae
Pedobacter petrophilus KX611481 Sphingobacteriaceae
Pedobacter psychrotolerans KU535673 Sphingobacteriaceae
Pedobacter rhizosphaerae AM279214 Sphingobacteriaceae
Pedobacter nutrimenti TYGS 2641 Sphingobacteriaceae
Pedobacter metabolipauper TYGS 2478 Sphingobacteriaceae
Pedobacter edaphicus KM035953 Sphingobacteriaceae
Pedobacter nyackensis TYGS 2366 Sphingobacteriaceae
Pedobacter humi KM073965 Sphingobacteriaceae
Pedobacter trunci KM035944 Sphingobacteriaceae
Pedobacter panaciterrae AB245368 Sphingobacteriaceae
Pedobacter heparinus TYGS 61 Sphingobacteriaceae
Pedobacter africanus AJ438171 Sphingobacteriaceae
Pedobacter lycopersici KU379664 Sphingobacteriaceae
Pedobacter ginsengisoli AB245371 Sphingobacteriaceae
Pedobacter seoulensis KF150693 Sphingobacteriaceae

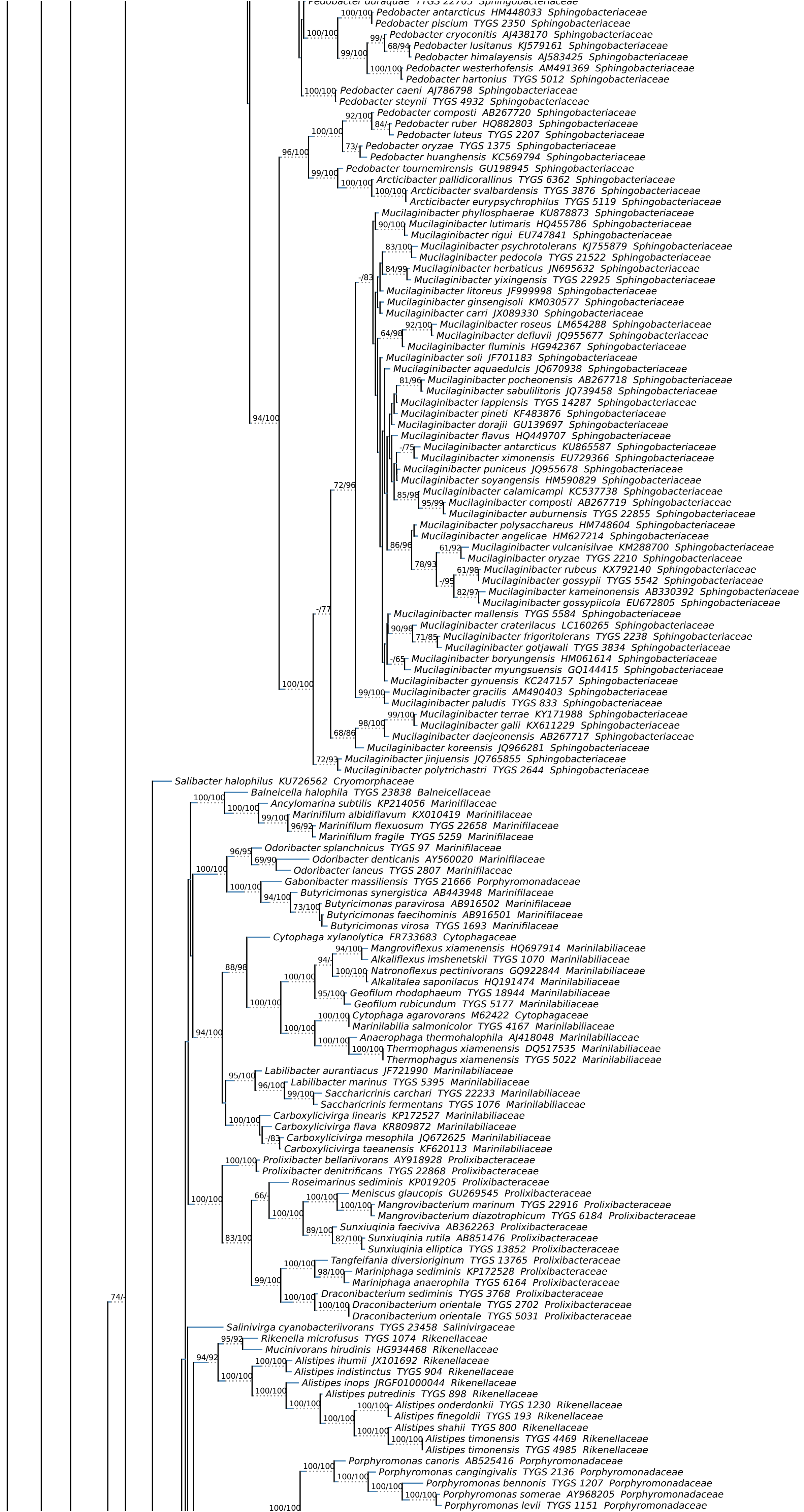
Pedobacter duraquae TYGS 22705 Sphingobacteriaceae
Pedobacter antarcticus HM448033 Sphingobacteriaceae
Pedobacter piscium TYGS 2350 Sphingobacteriaceae
Pedobacter cryoconitis AJ438170 Sphingobacteriaceae
Pedobacter lusitanus KJ579161 Sphingobacteriaceae
Pedobacter himalayensis AJ583425 Sphingobacteriaceae
Pedobacter westerhofensis AM491369 Sphingobacteriaceae
Pedobacter hartonius TYGS 5012 Sphingobacteriaceae
Pedobacter caeni AJ786798 Sphingobacteriaceae
Pedobacter steynii TYGS 4932 Sphingobacteriaceae
Pedobacter composti AB267720 Sphingobacteriaceae
Pedobacter ruber HQ882803 Sphingobacteriaceae
Pedobacter luteus TYGS 2207 Sphingobacteriaceae
Pedobacter oryzae TYGS 1375 Sphingobacteriaceae
Pedobacter huanghensis KC569794 Sphingobacteriaceae
Pedobacter tournemirensis GU198945 Sphingobacteriaceae
Arcticibacter pallidicorallinus TYGS 6362 Sphingobacteriaceae
Arcticibacter svalbardensis TYGS 3876 Sphingobacteriaceae
Arcticibacter eurypsychrophilus TYGS 5119 Sphingobacteriaceae
Mucilaginibacter phyllosphaerae KU878873 Sphingobacteriaceae
Mucilaginibacter lutimaris HQ455786 Sphingobacteriaceae
Mucilaginibacter rigui EU747841 Sphingobacteriaceae
Mucilaginibacter psychrotolerans KJ755879 Sphingobacteriaceae
Mucilaginibacter pedocola TYGS 21522 Sphingobacteriaceae
Mucilaginibacter herbaticus JN695632 Sphingobacteriaceae
Mucilaginibacter yixingensis TYGS 22925 Sphingobacteriaceae
Mucilaginibacter litoreus JF999998 Sphingobacteriaceae
Mucilaginibacter ginsengisoli KM030577 Sphingobacteriaceae
Mucilaginibacter carri JX089330 Sphingobacteriaceae
Mucilaginibacter roseus LM654288 Sphingobacteriaceae
Mucilaginibacter defluvii JQ955677 Sphingobacteriaceae
Mucilaginibacter fluminis HG942367 Sphingobacteriaceae
Mucilaginibacter soli JF701183 Sphingobacteriaceae
Mucilaginibacter aquaedulcis JQ670938 Sphingobacteriaceae
Mucilaginibacter pocheonensis AB267718 Sphingobacteriaceae
Mucilaginibacter sabulilitoris JQ739458 Sphingobacteriaceae
Mucilaginibacter lappiensis TYGS 14287 Sphingobacteriaceae
Mucilaginibacter pineti KF483876 Sphingobacteriaceae
Mucilaginibacter dorajii GU139697 Sphingobacteriaceae
Mucilaginibacter flavus HQ449707 Sphingobacteriaceae
Mucilaginibacter antarcticus KU865587 Sphingobacteriaceae
Mucilaginibacter ximonensis EU729366 Sphingobacteriaceae
Mucilaginibacter puniceus JQ955678 Sphingobacteriaceae
Mucilaginibacter soyangensis HM590829 Sphingobacteriaceae
Mucilaginibacter calamicampi KC537738 Sphingobacteriaceae
Mucilaginibacter composti AB267719 Sphingobacteriaceae
Mucilaginibacter auburnensis TYGS 22855 Sphingobacteriaceae
Mucilaginibacter polysacchareus HM748604 Sphingobacteriaceae
Mucilaginibacter angelicae HM627214 Sphingobacteriaceae
Mucilaginibacter vulcanisilvae KM288700 Sphingobacteriaceae
Mucilaginibacter oryzae TYGS 2210 Sphingobacteriaceae
Mucilaginibacter rubeus KX792140 Sphingobacteriaceae
Mucilaginibacter gossypii TYGS 5542 Sphingobacteriaceae
Mucilaginibacter kameinonensis AB330392 Sphingobacteriaceae
Mucilaginibacter gossypiicola EU672805 Sphingobacteriaceae
Mucilaginibacter mallensis TYGS 5584 Sphingobacteriaceae
Mucilaginibacter craterilacus LC160265 Sphingobacteriaceae
Mucilaginibacter frigoritolerans TYGS 2238 Sphingobacteriaceae
Mucilaginibacter gotjawali TYGS 3834 Sphingobacteriaceae
Mucilaginibacter boryungensis HM061614 Sphingobacteriaceae
Mucilaginibacter myungsuensis GQ144415 Sphingobacteriaceae
Mucilaginibacter gynuensis KC247157 Sphingobacteriaceae
Mucilaginibacter gracilis AM490403 Sphingobacteriaceae
Mucilaginibacter paludis TYGS 833 Sphingobacteriaceae
Mucilaginibacter terrae KY171988 Sphingobacteriaceae
Mucilaginibacter galii KX611229 Sphingobacteriaceae
Mucilaginibacter daejeonensis AB267717 Sphingobacteriaceae
Mucilaginibacter koreensis JQ966281 Sphingobacteriaceae
Mucilaginibacter jinjuensis JQ765855 Sphingobacteriaceae
Mucilaginibacter polytrichastri TYGS 2644 Sphingobacteriaceae
Salibacter halophilus KU726562 Cryomorphaceae
Balneicella halophila TYGS 23838 Balneicellaceae
Ancylomarina subtilis KP214056 Marinifilaceae
Marinifilum albidiflavum KX010419 Marinifilaceae
Marinifilum flexuosum TYGS 22658 Marinifilaceae
Marinifilum fragile TYGS 5259 Marinifilaceae
Odoribacter splanchnicus TYGS 97 Marinifilaceae
Odoribacter denticanis AY560020 Marinifilaceae
Odoribacter laneus TYGS 2807 Marinifilaceae
Gabonibacter massiliensis TYGS 21666 Porphyromonadaceae
Butyricimonas synergistica AB443948 Marinifilaceae
Butyricimonas paravirosa AB916502 Marinifilaceae
Butyricimonas faecihominis AB916501 Marinifilaceae
Butyricimonas virosa TYGS 1693 Marinifilaceae
Cytophaga xylanolytica FR733683 Cytophagaceae
Mangroviflexus xiamenensis HQ697914 Marinilabiliaceae
Alkaliflexus imshenetskii TYGS 1070 Marinilabiliaceae
Natronoflexus pectinivorans GQ922844 Marinilabiliaceae
Alkalitalea saponilacus HQ191474 Marinilabiliaceae
Geofilum rhodophaeum TYGS 18944 Marinilabiliaceae
Geofilum rubicundum TYGS 5177 Marinilabiliaceae
Cytophaga agarovorans M62422 Cytophagaceae
Marinilabilia salmonicolor TYGS 4167 Marinilabiliaceae
Anaerophaga thermohalophila AJ418048 Marinilabiliaceae
Thermophagus xiamenensis DQ517535 Marinilabiliaceae
Thermophagus xiamenensis TYGS 5022 Marinilabiliaceae
Labilibacter aurantiacus JF721990 Marinilabiliaceae
Labilibacter marinus TYGS 5395 Marinilabiliaceae
Saccharicrinis carchari TYGS 22233 Marinilabiliaceae
Saccharicrinis fermentans TYGS 1076 Marinilabiliaceae
Carboxylicivirga linearis KP172527 Marinilabiliaceae
Carboxylicivirga flava KR809872 Marinilabiliaceae
Carboxylicivirga mesophila JQ672625 Marinilabiliaceae
Carboxylicivirga taeanensis KF620113 Marinilabiliaceae
Prolixibacter bellariivorans AY918928 Prolixibacteraceae
Prolixibacter denitrificans TYGS 22868 Prolixibacteraceae
Roseimarinus sediminis KP019205 Prolixibacteraceae
Meniscus glaucopis GU269545 Prolixibacteraceae
Mangrovibacterium marinum TYGS 22916 Prolixibacteraceae
Mangrovibacterium diazotrophicum TYGS 6184 Prolixibacteraceae
Sunxiuqinia faeciviva AB362263 Prolixibacteraceae
Sunxiuqinia rutila AB851476 Prolixibacteraceae
Sunxiuqinia elliptica TYGS 13852 Prolixibacteraceae
Tangfeifania diversioriginum TYGS 13765 Prolixibacteraceae
Mariniphaga sediminis KP172528 Prolixibacteraceae
Mariniphaga anaerophila TYGS 6164 Prolixibacteraceae
Draconibacterium sediminis TYGS 3768 Prolixibacteraceae
Draconibacterium orientale TYGS 2702 Prolixibacteraceae
Draconibacterium orientale TYGS 5031 Prolixibacteraceae
Salinivirga cyanobacteriivorans TYGS 23458 Salinivirgaceae
Rikenella microfusus TYGS 1074 Rikenellaceae
Mucinivorans hirudinis HG934468 Rikenellaceae
Alistipes ihumii JX101692 Rikenellaceae
Alistipes indistinctus TYGS 904 Rikenellaceae
Alistipes inops JRGF01000044 Rikenellaceae
Alistipes putredinis TYGS 898 Rikenellaceae
Alistipes onderdonkii TYGS 1230 Rikenellaceae
Alistipes finegoldii TYGS 193 Rikenellaceae
Alistipes shahii TYGS 800 Rikenellaceae
Alistipes timonensis TYGS 4469 Rikenellaceae
Alistipes timonensis TYGS 4985 Rikenellaceae
Porphyromonas canoris AB525416 Porphyromonadaceae
Porphyromonas cangingivalis TYGS 2136 Porphyromonadaceae
Porphyromonas bennonis TYGS 1207 Porphyromonadaceae
Porphyromonas somerae AY968205 Porphyromonadaceae
Porphyromonas levii TYGS 1151 Porphyromonadaceae

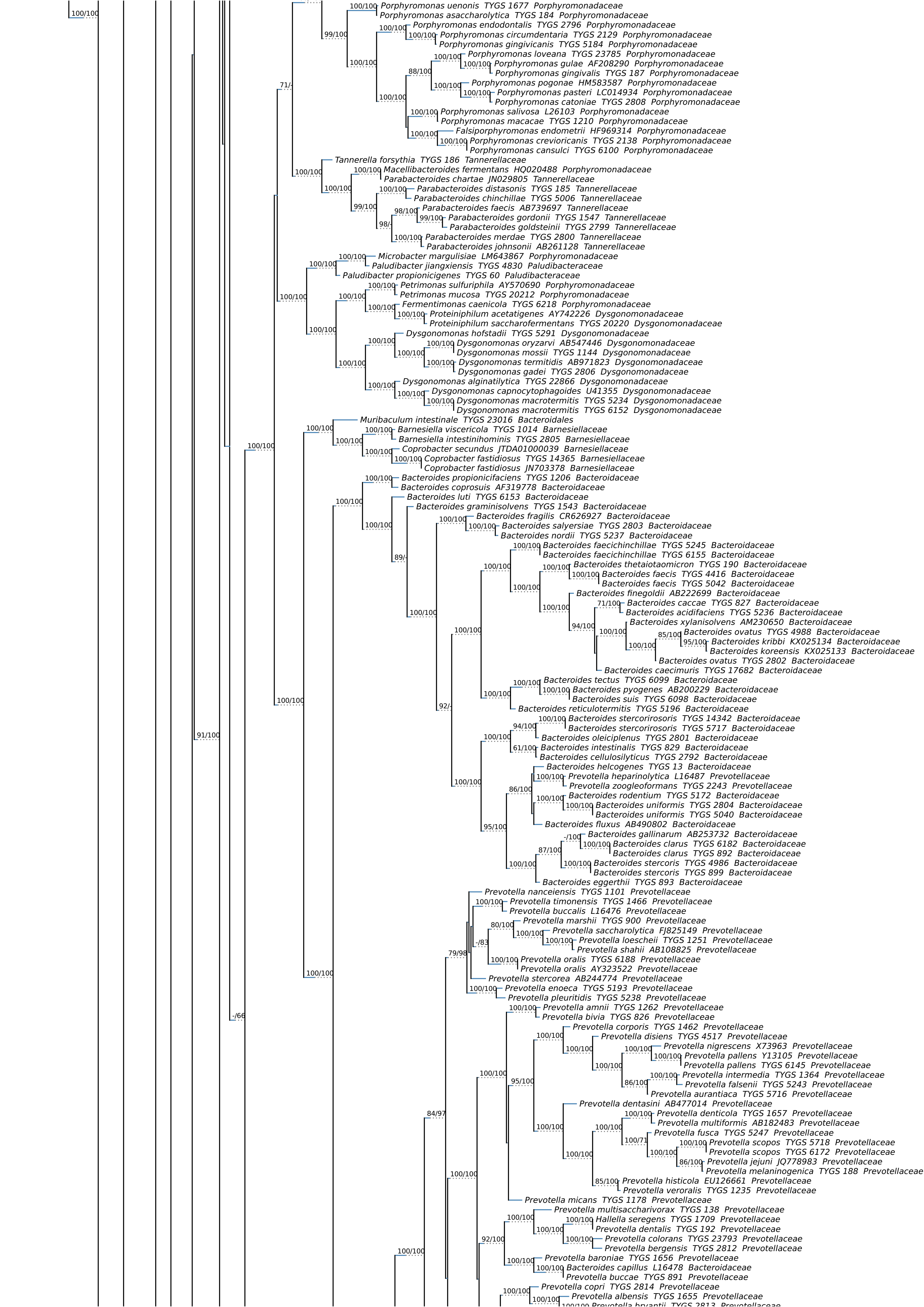
Porphyromonas uenonis TYGS 1677 Porphyromonadaceae
Porphyromonas asaccharolytica TYGS 184 Porphyromonadaceae
Porphyromonas endodontalis TYGS 2796 Porphyromonadaceae
Porphyromonas circumdentaria TYGS 2129 Porphyromonadaceae
Porphyromonas gingivicanis TYGS 5184 Porphyromonadaceae
Porphyromonas loveana TYGS 23785 Porphyromonadaceae
Porphyromonas gulae AF208290 Porphyromonadaceae
Porphyromonas gingivalis TYGS 187 Porphyromonadaceae
Porphyromonas pogonae HM583587 Porphyromonadaceae
Porphyromonas pasteri LC014934 Porphyromonadaceae
Porphyromonas catoniae TYGS 2808 Porphyromonadaceae
Porphyromonas salivosa L26103 Porphyromonadaceae
Porphyromonas macacae TYGS 1210 Porphyromonadaceae
Falsiporphyromonas endometrii HF969314 Porphyromonadaceae
Porphyromonas crevioricanis TYGS 2138 Porphyromonadaceae
Porphyromonas cansulci TYGS 6100 Porphyromonadaceae
Tannerella forsythia TYGS 186 Tannerellaceae
Macellibacteroides fermentans HQ020488 Porphyromonadaceae
Parabacteroides chartae JN029805 Tannerellaceae
Parabacteroides distasonis TYGS 185 Tannerellaceae
Parabacteroides chinchillae TYGS 5006 Tannerellaceae
Parabacteroides faecis AB739697 Tannerellaceae
Parabacteroides gordonii TYGS 1547 Tannerellaceae
Parabacteroides goldsteinii TYGS 2799 Tannerellaceae
Parabacteroides merdae TYGS 2800 Tannerellaceae
Parabacteroides johnsonii AB261128 Tannerellaceae
Microbacter margulisiae LM643867 Porphyromonadaceae
Paludibacter jiangxiensis TYGS 4830 Paludibacteraceae
Paludibacter propionicigenes TYGS 60 Paludibacteraceae
Petrimonas sulfuriphila AY570690 Porphyromonadaceae
Petrimonas mucosa TYGS 20212 Porphyromonadaceae
Fermentimonas caenicola TYGS 6218 Porphyromonadaceae
Proteiniphilum acetatigenes AY742226 Dysgonomonadaceae
Proteiniphilum saccharofermentans TYGS 20220 Dysgonomonadaceae
Dysgonomonas hofstadii TYGS 5291 Dysgonomonadaceae
Dysgonomonas oryzarvi AB547446 Dysgonomonadaceae
Dysgonomonas mossii TYGS 1144 Dysgonomonadaceae
Dysgonomonas termitidis AB971823 Dysgonomonadaceae
Dysgonomonas gadei TYGS 2806 Dysgonomonadaceae
Dysgonomonas alginatilytica TYGS 22866 Dysgonomonadaceae
Dysgonomonas capnocytophagoides U41355 Dysgonomonadaceae
Dysgonomonas macrotermitis TYGS 5234 Dysgonomonadaceae
Dysgonomonas macrotermitis TYGS 6152 Dysgonomonadaceae
Muribaculum intestinale TYGS 23016 Bacteroidales
Barnesiella viscericola TYGS 1014 Barnesiellaceae
Barnesiella intestinihominis TYGS 2805 Barnesiellaceae
Coprobacter secundus JTDA01000039 Barnesiellaceae
Coprobacter fastidiosus TYGS 14365 Barnesiellaceae
Coprobacter fastidiosus JN703378 Barnesiellaceae
Bacteroides propionicifaciens TYGS 1206 Bacteroidaceae
Bacteroides coprosuis AF319778 Bacteroidaceae
Bacteroides luti TYGS 6153 Bacteroidaceae
Bacteroides graminisolvens TYGS 1543 Bacteroidaceae
Bacteroides fragilis CR626927 Bacteroidaceae
Bacteroides salyersiae TYGS 2803 Bacteroidaceae
Bacteroides nordii TYGS 5237 Bacteroidaceae
Bacteroides faecichinchillae TYGS 5245 Bacteroidaceae
Bacteroides faecichinchillae TYGS 6155 Bacteroidaceae
Bacteroides thetaiotaomicron TYGS 190 Bacteroidaceae
Bacteroides faecis TYGS 4416 Bacteroidaceae
Bacteroides faecis TYGS 5042 Bacteroidaceae
Bacteroides finegoldii AB222699 Bacteroidaceae
Bacteroides caccae TYGS 827 Bacteroidaceae
Bacteroides acidifaciens TYGS 5236 Bacteroidaceae
Bacteroides xylanisolvens AM230650 Bacteroidaceae
Bacteroides ovatus TYGS 4988 Bacteroidaceae
Bacteroides kribbi KX025134 Bacteroidaceae
Bacteroides koreensis KX025133 Bacteroidaceae
Bacteroides ovatus TYGS 2802 Bacteroidaceae
Bacteroides caecimuris TYGS 17682 Bacteroidaceae
Bacteroides tectus TYGS 6099 Bacteroidaceae
Bacteroides pyogenes AB200229 Bacteroidaceae
Bacteroides suis TYGS 6098 Bacteroidaceae
Bacteroides reticulotermitis TYGS 5196 Bacteroidaceae
Bacteroides stercorirosoris TYGS 14342 Bacteroidaceae
Bacteroides stercorirosoris TYGS 5717 Bacteroidaceae
Bacteroides oleiciplenus TYGS 2801 Bacteroidaceae
Bacteroides intestinalis TYGS 829 Bacteroidaceae
Bacteroides cellulosilyticus TYGS 2792 Bacteroidaceae
Bacteroides helcogenes TYGS 13 Bacteroidaceae
Prevotella heparinolytica L16487 Prevotellaceae
Prevotella zoogleoformans TYGS 2243 Prevotellaceae
Bacteroides rodentium TYGS 5172 Bacteroidaceae
Bacteroides uniformis TYGS 2804 Bacteroidaceae
Bacteroides uniformis TYGS 5040 Bacteroidaceae
Bacteroides fluxus AB490802 Bacteroidaceae
Bacteroides gallinarum AB253732 Bacteroidaceae
Bacteroides clarus TYGS 6182 Bacteroidaceae
Bacteroides clarus TYGS 892 Bacteroidaceae
Bacteroides stercoris TYGS 4986 Bacteroidaceae
Bacteroides stercoris TYGS 899 Bacteroidaceae
Bacteroides eggerthii TYGS 893 Bacteroidaceae
Prevotella nanceiensis TYGS 1101 Prevotellaceae
Prevotella timonensis TYGS 1466 Prevotellaceae
Prevotella buccalis L16476 Prevotellaceae
Prevotella marshii TYGS 900 Prevotellaceae
Prevotella saccharolytica FJ825149 Prevotellaceae
Prevotella loescheii TYGS 1251 Prevotellaceae
Prevotella shahii AB108825 Prevotellaceae
Prevotella oralis TYGS 6188 Prevotellaceae
Prevotella oralis AY323522 Prevotellaceae
Prevotella stercorea AB244774 Prevotellaceae
Prevotella enoeca TYGS 5193 Prevotellaceae
Prevotella pleuritidis TYGS 5238 Prevotellaceae
Prevotella amnii TYGS 1262 Prevotellaceae
Prevotella bivia TYGS 826 Prevotellaceae
Prevotella corporis TYGS 1462 Prevotellaceae
Prevotella disiens TYGS 4517 Prevotellaceae
Prevotella nigrescens X73963 Prevotellaceae
Prevotella pallens Y13105 Prevotellaceae
Prevotella pallens TYGS 6145 Prevotellaceae
Prevotella intermedia TYGS 1364 Prevotellaceae
Prevotella falsenii TYGS 5243 Prevotellaceae
Prevotella aurantiaca TYGS 5716 Prevotellaceae
Prevotella dentasini AB477014 Prevotellaceae
Prevotella denticola TYGS 1657 Prevotellaceae
Prevotella multiformis AB182483 Prevotellaceae
Prevotella fusca TYGS 5247 Prevotellaceae
Prevotella scopos TYGS 5718 Prevotellaceae
Prevotella scopos TYGS 6172 Prevotellaceae
Prevotella jejuni JQ778983 Prevotellaceae
Prevotella melaninogenica TYGS 188 Prevotellaceae
Prevotella histicola EU126661 Prevotellaceae
Prevotella veroralis TYGS 1235 Prevotellaceae
Prevotella micans TYGS 1178 Prevotellaceae
Prevotella multisaccharivorax TYGS 138 Prevotellaceae
Hallella seregens TYGS 1709 Prevotellaceae
Prevotella dentalis TYGS 192 Prevotellaceae
Prevotella colorans TYGS 23793 Prevotellaceae
Prevotella bergensis TYGS 2812 Prevotellaceae
Prevotella baroniae TYGS 1656 Prevotellaceae
Bacteroides capillus L16478 Bacteroidaceae
Prevotella buccae TYGS 891 Prevotellaceae
Prevotella copri TYGS 2814 Prevotellaceae
Prevotella albensis TYGS 1655 Prevotellaceae

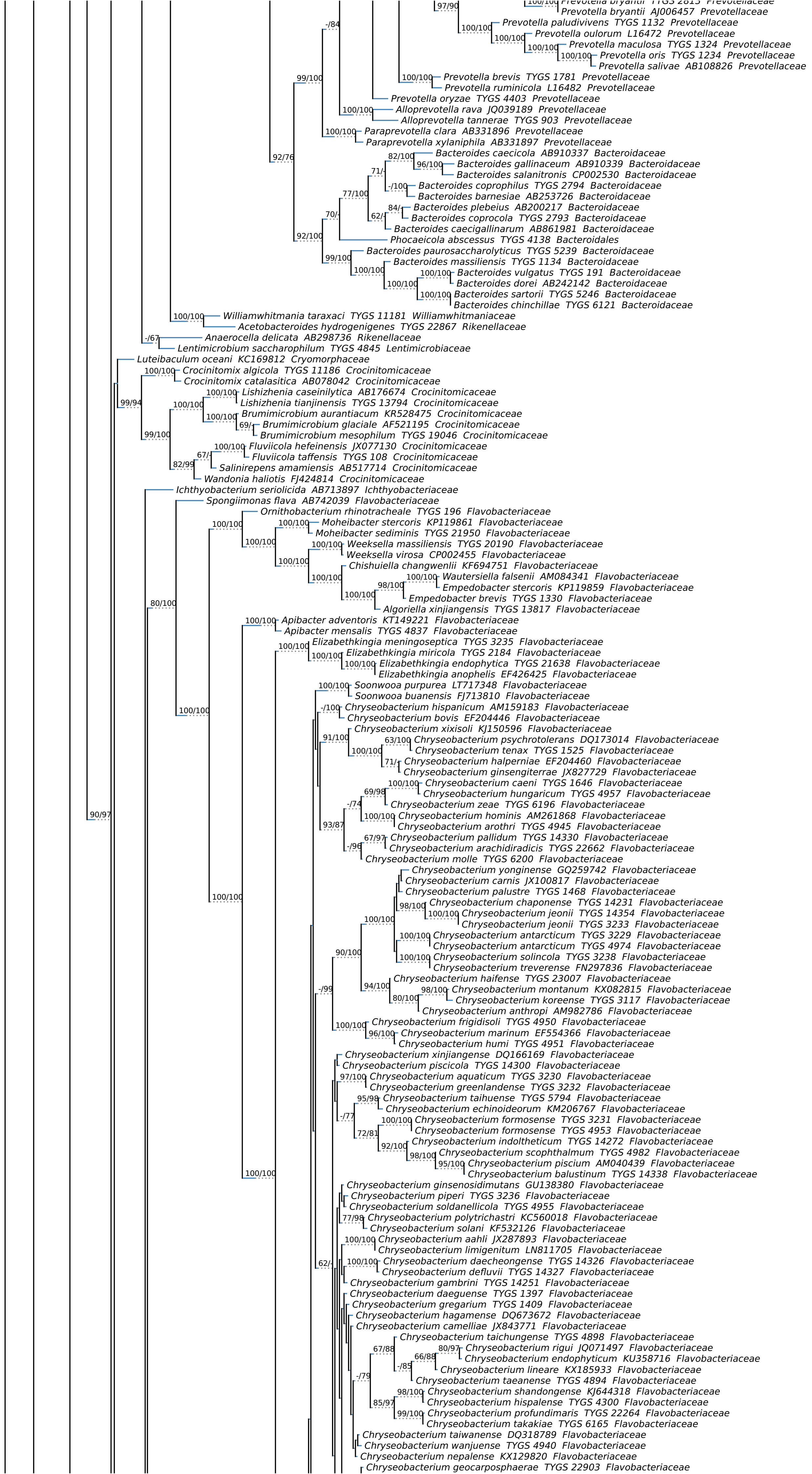
Prevotella bryantii AJ006457 Prevotellaceae
Prevotella paludivivens TYGS 1132 Prevotellaceae
Prevotella oulorum L16472 Prevotellaceae
Prevotella maculosa TYGS 1324 Prevotellaceae
Prevotella oris TYGS 1234 Prevotellaceae
Prevotella salivae AB108826 Prevotellaceae
Prevotella brevis TYGS 1781 Prevotellaceae
Prevotella ruminicola L16482 Prevotellaceae
Prevotella oryzae TYGS 4403 Prevotellaceae
Alloprevotella rava JQ039189 Prevotellaceae
Alloprevotella tannerae TYGS 903 Prevotellaceae
Paraprevotella clara AB331896 Prevotellaceae
Paraprevotella xylaniphila AB331897 Prevotellaceae
Bacteroides caecicola AB910337 Bacteroidaceae
Bacteroides gallinaceum AB910339 Bacteroidaceae
Bacteroides salanitronis CP002530 Bacteroidaceae
Bacteroides coprophilus TYGS 2794 Bacteroidaceae
Bacteroides barnesiae AB253726 Bacteroidaceae
Bacteroides plebeius AB200217 Bacteroidaceae
Bacteroides coprocola TYGS 2793 Bacteroidaceae
Bacteroides caecigallinarum AB861981 Bacteroidaceae
Phocaeicola abscessus TYGS 4138 Bacteroidales
Bacteroides paurosaccharolyticus TYGS 5239 Bacteroidaceae
Bacteroides massiliensis TYGS 1134 Bacteroidaceae
Bacteroides vulgatus TYGS 191 Bacteroidaceae
Bacteroides dorei AB242142 Bacteroidaceae
Bacteroides sartorii TYGS 5246 Bacteroidaceae
Bacteroides chinchillae TYGS 6121 Bacteroidaceae
Williamwhitmania taraxaci TYGS 11181 Williamwhitmaniaceae
Acetobacteroides hydrogenigenes TYGS 22867 Rikenellaceae
Anaerocella delicata AB298736 Rikenellaceae
Lentimicrobium saccharophilum TYGS 4845 Lentimicrobiaceae
Luteibaculum oceani KC169812 Cryomorphaceae
Crocinitomix algicola TYGS 11186 Crocinitomicaceae
Crocinitomix catalasitica AB078042 Crocinitomicaceae
Lishizhenia caseinilytica AB176674 Crocinitomicaceae
Lishizhenia tianjinensis TYGS 13794 Crocinitomicaceae
Brumimicrobium aurantiacum KR528475 Crocinitomicaceae
Brumimicrobium glaciale AF521195 Crocinitomicaceae
Brumimicrobium mesophilum TYGS 19046 Crocinitomicaceae
Fluviicola hefeinensis JX077130 Crocinitomicaceae
Fluviicola taffensis TYGS 108 Crocinitomicaceae
Salinirepens amamiensis AB517714 Crocinitomicaceae
Wandonia haliotis FJ424814 Crocinitomicaceae
Ichthyobacterium seriolicida AB713897 Ichthyobacteriaceae
Spongiimonas flava AB742039 Flavobacteriaceae
Ornithobacterium rhinotracheale TYGS 196 Flavobacteriaceae
Moheibacter stercoris KP119861 Flavobacteriaceae
Moheibacter sediminis TYGS 21950 Flavobacteriaceae
Weeksella massiliensis TYGS 20190 Flavobacteriaceae
Weeksella virosa CP002455 Flavobacteriaceae
Chishuiella changwenlii KF694751 Flavobacteriaceae
Wautersiella falsenii AM084341 Flavobacteriaceae
Empedobacter stercoris KP119859 Flavobacteriaceae
Empedobacter brevis TYGS 1330 Flavobacteriaceae
Algoriella xinjiangensis TYGS 13817 Flavobacteriaceae
Apibacter adventoris KT149221 Flavobacteriaceae
Apibacter mensalis TYGS 4837 Flavobacteriaceae
Elizabethkingia meningoseptica TYGS 3235 Flavobacteriaceae
Elizabethkingia miricola TYGS 2184 Flavobacteriaceae
Elizabethkingia endophytica TYGS 21638 Flavobacteriaceae
Elizabethkingia anophelis EF426425 Flavobacteriaceae
Soonwooa purpurea LT717348 Flavobacteriaceae
Soonwooa buanensis FJ713810 Flavobacteriaceae
Chryseobacterium hispanicum AM159183 Flavobacteriaceae
Chryseobacterium bovis EF204446 Flavobacteriaceae
Chryseobacterium xixisoli KJ150596 Flavobacteriaceae
Chryseobacterium psychrotolerans DQ173014 Flavobacteriaceae
Chryseobacterium tenax TYGS 1525 Flavobacteriaceae
Chryseobacterium halperniae EF204460 Flavobacteriaceae
Chryseobacterium ginsengiterrae JX827729 Flavobacteriaceae
Chryseobacterium caeni TYGS 1646 Flavobacteriaceae
Chryseobacterium hungaricum TYGS 4957 Flavobacteriaceae
Chryseobacterium zeae TYGS 6196 Flavobacteriaceae
Chryseobacterium hominis AM261868 Flavobacteriaceae
Chryseobacterium arothri TYGS 4945 Flavobacteriaceae
Chryseobacterium pallidum TYGS 14330 Flavobacteriaceae
Chryseobacterium arachidiradicis TYGS 22662 Flavobacteriaceae
Chryseobacterium molle TYGS 6200 Flavobacteriaceae
Chryseobacterium yonginense GQ259742 Flavobacteriaceae
Chryseobacterium carnis JX100817 Flavobacteriaceae
Chryseobacterium palustre TYGS 1468 Flavobacteriaceae
Chryseobacterium chaponense TYGS 14231 Flavobacteriaceae
Chryseobacterium jeonii TYGS 14354 Flavobacteriaceae
Chryseobacterium jeonii TYGS 3233 Flavobacteriaceae
Chryseobacterium antarcticum TYGS 3229 Flavobacteriaceae
Chryseobacterium antarcticum TYGS 4974 Flavobacteriaceae
Chryseobacterium solincola TYGS 3238 Flavobacteriaceae
Chryseobacterium treverense FN297836 Flavobacteriaceae
Chryseobacterium haifense TYGS 23007 Flavobacteriaceae
Chryseobacterium montanum KX082815 Flavobacteriaceae
Chryseobacterium koreense TYGS 3117 Flavobacteriaceae
Chryseobacterium anthropi AM982786 Flavobacteriaceae
Chryseobacterium frigidisoli TYGS 4950 Flavobacteriaceae
Chryseobacterium marinum EF554366 Flavobacteriaceae
Chryseobacterium humi TYGS 4951 Flavobacteriaceae
Chryseobacterium xinjiangense DQ166169 Flavobacteriaceae
Chryseobacterium piscicola TYGS 14300 Flavobacteriaceae
Chryseobacterium aquaticum TYGS 3230 Flavobacteriaceae
Chryseobacterium greenlandense TYGS 3232 Flavobacteriaceae
Chryseobacterium taihuense TYGS 5794 Flavobacteriaceae
Chryseobacterium echinoideorum KM206767 Flavobacteriaceae
Chryseobacterium formosense TYGS 3231 Flavobacteriaceae
Chryseobacterium formosense TYGS 4953 Flavobacteriaceae
Chryseobacterium indoltheticum TYGS 14272 Flavobacteriaceae
Chryseobacterium scophthalmum TYGS 4982 Flavobacteriaceae
Chryseobacterium piscium AM040439 Flavobacteriaceae
Chryseobacterium balustinum TYGS 14338 Flavobacteriaceae
Chryseobacterium ginsenosidimutans GU138380 Flavobacteriaceae
Chryseobacterium piperi TYGS 3236 Flavobacteriaceae
Chryseobacterium soldanellicola TYGS 4955 Flavobacteriaceae
Chryseobacterium polytrichastri KC560018 Flavobacteriaceae
Chryseobacterium solani KF532126 Flavobacteriaceae
Chryseobacterium aahli JX287893 Flavobacteriaceae
Chryseobacterium limigenitum LN811705 Flavobacteriaceae
Chryseobacterium daecheongense TYGS 14326 Flavobacteriaceae
Chryseobacterium defluvii TYGS 14327 Flavobacteriaceae
Chryseobacterium gambrini TYGS 14251 Flavobacteriaceae
Chryseobacterium daeguense TYGS 1397 Flavobacteriaceae
Chryseobacterium gregarium TYGS 1409 Flavobacteriaceae
Chryseobacterium hagamense DQ673672 Flavobacteriaceae
Chryseobacterium camelliae JX843771 Flavobacteriaceae
Chryseobacterium taichungense TYGS 4898 Flavobacteriaceae
Chryseobacterium rigui JQ071497 Flavobacteriaceae
Chryseobacterium endophyticum KU358716 Flavobacteriaceae
Chryseobacterium lineare KX185933 Flavobacteriaceae
Chryseobacterium taeanense TYGS 4894 Flavobacteriaceae
Chryseobacterium shandongense KJ644318 Flavobacteriaceae
Chryseobacterium hispalense TYGS 4300 Flavobacteriaceae
Chryseobacterium profundimaris TYGS 22264 Flavobacteriaceae
Chryseobacterium takakiae TYGS 6165 Flavobacteriaceae
Chryseobacterium taiwanense DQ318789 Flavobacteriaceae
Chryseobacterium wanjuense TYGS 4940 Flavobacteriaceae
Chryseobacterium nepalense KX129820 Flavobacteriaceae
Chryseobacterium geocarposphaerae TYGS 22903 Flavobacteriaceae

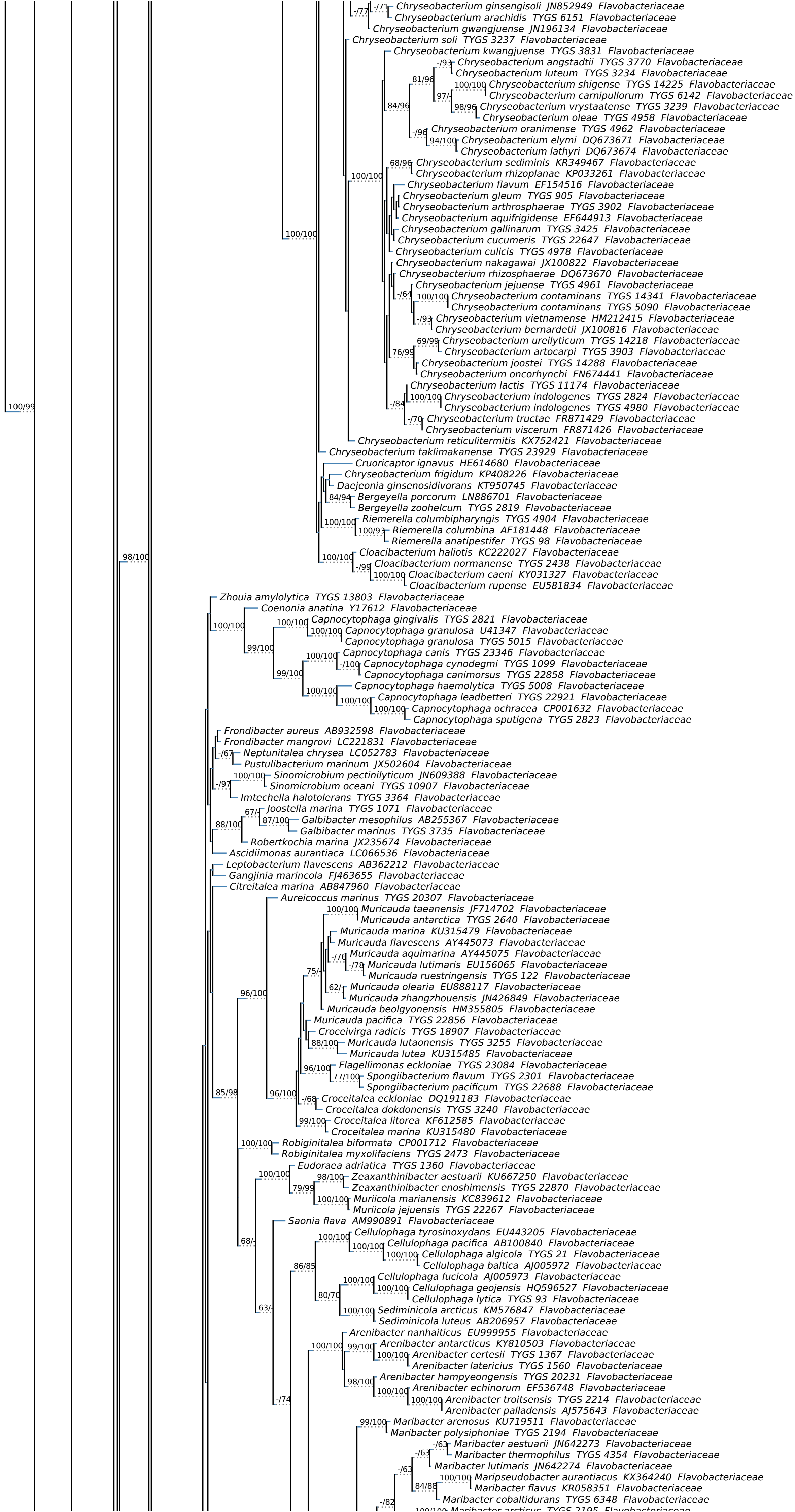
Chryseobacterium ginsengisoli JN852949 Flavobacteriaceae
Chryseobacterium arachidis TYGS 6151 Flavobacteriaceae
Chryseobacterium gwangjuense JN196134 Flavobacteriaceae
Chryseobacterium soli TYGS 3237 Flavobacteriaceae
Chryseobacterium kwangjuense TYGS 3831 Flavobacteriaceae
Chryseobacterium angstadtii TYGS 3770 Flavobacteriaceae
Chryseobacterium luteum TYGS 3234 Flavobacteriaceae
Chryseobacterium shigense TYGS 14225 Flavobacteriaceae
Chryseobacterium carnipullorum TYGS 6142 Flavobacteriaceae
Chryseobacterium vrystaatense TYGS 3239 Flavobacteriaceae
Chryseobacterium oleae TYGS 4958 Flavobacteriaceae
Chryseobacterium oranimense TYGS 4962 Flavobacteriaceae
Chryseobacterium elymi DQ673671 Flavobacteriaceae
Chryseobacterium lathyri DQ673674 Flavobacteriaceae
Chryseobacterium sediminis KR349467 Flavobacteriaceae
Chryseobacterium rhizoplanae KP033261 Flavobacteriaceae
Chryseobacterium flavum EF154516 Flavobacteriaceae
Chryseobacterium gleum TYGS 905 Flavobacteriaceae
Chryseobacterium arthrosphaerae TYGS 3902 Flavobacteriaceae
Chryseobacterium aquifrigidense EF644913 Flavobacteriaceae
Chryseobacterium gallinarum TYGS 3425 Flavobacteriaceae
Chryseobacterium cucumeris TYGS 22647 Flavobacteriaceae
Chryseobacterium culicis TYGS 4978 Flavobacteriaceae
Chryseobacterium nakagawai JX100822 Flavobacteriaceae
Chryseobacterium rhizosphaerae DQ673670 Flavobacteriaceae
Chryseobacterium jejuense TYGS 4961 Flavobacteriaceae
Chryseobacterium contaminans TYGS 14341 Flavobacteriaceae
Chryseobacterium contaminans TYGS 5090 Flavobacteriaceae
Chryseobacterium vietnamense HM212415 Flavobacteriaceae
Chryseobacterium bernardetii JX100816 Flavobacteriaceae
Chryseobacterium ureilyticum TYGS 14218 Flavobacteriaceae
Chryseobacterium artocarpi TYGS 3903 Flavobacteriaceae
Chryseobacterium joostei TYGS 14288 Flavobacteriaceae
Chryseobacterium oncorhynchi FN674441 Flavobacteriaceae
Chryseobacterium lactis TYGS 11174 Flavobacteriaceae
Chryseobacterium indologenes TYGS 2824 Flavobacteriaceae
Chryseobacterium indologenes TYGS 4980 Flavobacteriaceae
Chryseobacterium tructae FR871429 Flavobacteriaceae
Chryseobacterium viscerum FR871426 Flavobacteriaceae
Chryseobacterium reticulitermitis KX752421 Flavobacteriaceae
Chryseobacterium taklimakanense TYGS 23929 Flavobacteriaceae
Cruoricaptor ignavus HE614680 Flavobacteriaceae
Chryseobacterium frigidum KP408226 Flavobacteriaceae
Daejeonia ginsenosidivorans KT950745 Flavobacteriaceae
Bergeyella porcorum LN886701 Flavobacteriaceae
Bergeyella zoohelcum TYGS 2819 Flavobacteriaceae
Riemerella columbipharyngis TYGS 4904 Flavobacteriaceae
Riemerella columbina AF181448 Flavobacteriaceae
Riemerella anatipestifer TYGS 98 Flavobacteriaceae
Cloacibacterium haliotis KC222027 Flavobacteriaceae
Cloacibacterium normanense TYGS 2438 Flavobacteriaceae
Cloacibacterium caeni KY031327 Flavobacteriaceae
Cloacibacterium rupense EU581834 Flavobacteriaceae
Zhouia amylolytica TYGS 13803 Flavobacteriaceae
Coenonia anatina Y17612 Flavobacteriaceae
Capnocytophaga gingivalis TYGS 2821 Flavobacteriaceae
Capnocytophaga granulosa U41347 Flavobacteriaceae
Capnocytophaga granulosa TYGS 5015 Flavobacteriaceae
Capnocytophaga canis TYGS 23346 Flavobacteriaceae
Capnocytophaga cynodegmi TYGS 1099 Flavobacteriaceae
Capnocytophaga canimorsus TYGS 22858 Flavobacteriaceae
Capnocytophaga haemolytica TYGS 5008 Flavobacteriaceae
Capnocytophaga leadbetteri TYGS 22921 Flavobacteriaceae
Capnocytophaga ochracea CP001632 Flavobacteriaceae
Capnocytophaga sputigena TYGS 2823 Flavobacteriaceae
Frondibacter aureus AB932598 Flavobacteriaceae
Frondibacter mangrovi LC221831 Flavobacteriaceae
Neptunitalea chrysea LC052783 Flavobacteriaceae
Pustulibacterium marinum JX502604 Flavobacteriaceae
Sinomicrobium pectinilyticum JN609388 Flavobacteriaceae
Sinomicrobium oceani TYGS 10907 Flavobacteriaceae
Imtechella halotolerans TYGS 3364 Flavobacteriaceae
Joostella marina TYGS 1071 Flavobacteriaceae
Galbibacter mesophilus AB255367 Flavobacteriaceae
Galbibacter marinus TYGS 3735 Flavobacteriaceae
Robertkochia marina JX235674 Flavobacteriaceae
Ascidiimonas aurantiaca LC066536 Flavobacteriaceae
Leptobacterium flavescens AB362212 Flavobacteriaceae
Gangjinia marincola FJ463655 Flavobacteriaceae
Citreitalea marina AB847960 Flavobacteriaceae
Aureicoccus marinus TYGS 20307 Flavobacteriaceae
Muricauda taeanensis JF714702 Flavobacteriaceae
Muricauda antarctica TYGS 2640 Flavobacteriaceae
Muricauda marina KU315479 Flavobacteriaceae
Muricauda flavescens AY445073 Flavobacteriaceae
Muricauda aquimarina AY445075 Flavobacteriaceae
Muricauda lutimaris EU156065 Flavobacteriaceae
Muricauda ruestringensis TYGS 122 Flavobacteriaceae
Muricauda olearia EU888117 Flavobacteriaceae
Muricauda zhangzhouensis JN426849 Flavobacteriaceae
Muricauda beolgyonensis HM355805 Flavobacteriaceae
Muricauda pacifica TYGS 22856 Flavobacteriaceae
Croceivirga radicis TYGS 18907 Flavobacteriaceae
Muricauda lutaonensis TYGS 3255 Flavobacteriaceae
Muricauda lutea KU315485 Flavobacteriaceae
Flagellimonas eckloniae TYGS 23084 Flavobacteriaceae
Spongiibacterium flavum TYGS 2301 Flavobacteriaceae
Spongiibacterium pacificum TYGS 22688 Flavobacteriaceae
Croceitalea eckloniae DQ191183 Flavobacteriaceae
Croceitalea dokdonensis TYGS 3240 Flavobacteriaceae
Croceitalea litorea KF612585 Flavobacteriaceae
Croceitalea marina KU315480 Flavobacteriaceae
Robiginitalea biformata CP001712 Flavobacteriaceae
Robiginitalea myxolifaciens TYGS 2473 Flavobacteriaceae
Eudoraea adriatica TYGS 1360 Flavobacteriaceae
Zeaxanthinibacter aestuarii KU667250 Flavobacteriaceae
Zeaxanthinibacter enoshimensis TYGS 22870 Flavobacteriaceae
Muriicola marianensis KC839612 Flavobacteriaceae
Muriicola jejuensis TYGS 22267 Flavobacteriaceae
Saonia flava AM990891 Flavobacteriaceae
Cellulophaga tyrosinoxydans EU443205 Flavobacteriaceae
Cellulophaga pacifica AB100840 Flavobacteriaceae
Cellulophaga algicola TYGS 21 Flavobacteriaceae
Cellulophaga baltica AJ005972 Flavobacteriaceae
Cellulophaga fucicola AJ005973 Flavobacteriaceae
Cellulophaga geojensis HQ596527 Flavobacteriaceae
Cellulophaga lytica TYGS 93 Flavobacteriaceae
Sediminicola arcticus KM576847 Flavobacteriaceae
Sediminicola luteus AB206957 Flavobacteriaceae
Arenibacter nanhaiticus EU999955 Flavobacteriaceae
Arenibacter antarcticus KY810503 Flavobacteriaceae
Arenibacter certesii TYGS 1367 Flavobacteriaceae
Arenibacter latericius TYGS 1560 Flavobacteriaceae
Arenibacter hampyeongensis TYGS 20231 Flavobacteriaceae
Arenibacter echinorum EF536748 Flavobacteriaceae
Arenibacter troitsensis TYGS 2214 Flavobacteriaceae
Arenibacter palladensis AJ575643 Flavobacteriaceae
Maribacter arenosus KU719511 Flavobacteriaceae
Maribacter polysiphoniae TYGS 2194 Flavobacteriaceae
Maribacter aestuarii JN642273 Flavobacteriaceae
Maribacter thermophilus TYGS 4354 Flavobacteriaceae
Maribacter lutimaris JN642274 Flavobacteriaceae
Maripseudobacter aurantiacus KX364240 Flavobacteriaceae
Maribacter flavus KR058351 Flavobacteriaceae
Maribacter cobaltidurans TYGS 6348 Flavobacteriaceae

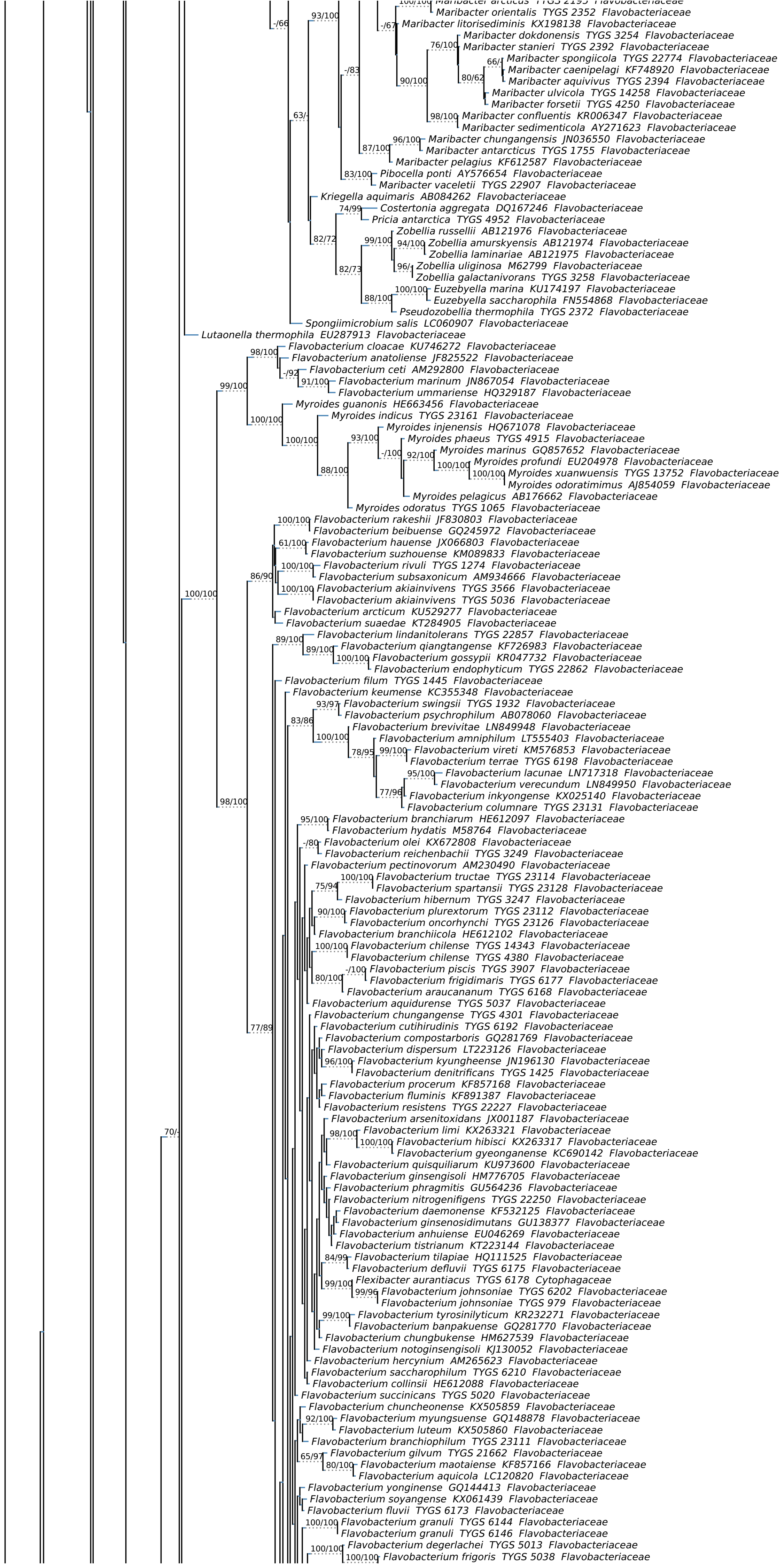
Maribacter orientalis TYGS 2352 Flavobacteriaceae
Maribacter litorisediminis KX198138 Flavobacteriaceae
Maribacter dokdonensis TYGS 3254 Flavobacteriaceae
Maribacter stanieri TYGS 2392 Flavobacteriaceae
Maribacter spongiicola TYGS 22774 Flavobacteriaceae
Maribacter caenipelagi KF748920 Flavobacteriaceae
Maribacter aquivivus TYGS 2394 Flavobacteriaceae
Maribacter ulvicola TYGS 14258 Flavobacteriaceae
Maribacter forsetii TYGS 4250 Flavobacteriaceae
Maribacter confluentis KR006347 Flavobacteriaceae
Maribacter sedimenticola AY271623 Flavobacteriaceae
Maribacter chungangensis JN036550 Flavobacteriaceae
Maribacter antarcticus TYGS 1755 Flavobacteriaceae
Maribacter pelagius KF612587 Flavobacteriaceae
Pibocella ponti AY576654 Flavobacteriaceae
Maribacter vaceletii TYGS 22907 Flavobacteriaceae
Kriegella aquimaris AB084262 Flavobacteriaceae
Costertonia aggregata DQ167246 Flavobacteriaceae
Pricia antarctica TYGS 4952 Flavobacteriaceae
Zobellia russellii AB121976 Flavobacteriaceae
Zobellia amurskyensis AB121974 Flavobacteriaceae
Zobellia laminariae AB121975 Flavobacteriaceae
Zobellia uliginosa M62799 Flavobacteriaceae
Zobellia galactanivorans TYGS 3258 Flavobacteriaceae
Euzebyella marina KU174197 Flavobacteriaceae
Euzebyella saccharophila FN554868 Flavobacteriaceae
Pseudozobellia thermophila TYGS 2372 Flavobacteriaceae
Spongiimicrobium salis LC060907 Flavobacteriaceae
Lutaonella thermophila EU287913 Flavobacteriaceae
Flavobacterium cloacae KU746272 Flavobacteriaceae
Flavobacterium anatoliense JF825522 Flavobacteriaceae
Flavobacterium ceti AM292800 Flavobacteriaceae
Flavobacterium marinum JN867054 Flavobacteriaceae
Flavobacterium ummariense HQ329187 Flavobacteriaceae
Myroides guanonis HE663456 Flavobacteriaceae
Myroides indicus TYGS 23161 Flavobacteriaceae
Myroides injenensis HQ671078 Flavobacteriaceae
Myroides phaeus TYGS 4915 Flavobacteriaceae
Myroides marinus GQ857652 Flavobacteriaceae
Myroides profundi EU204978 Flavobacteriaceae
Myroides xuanwuensis TYGS 13752 Flavobacteriaceae
Myroides odoratimimus AJ854059 Flavobacteriaceae
Myroides pelagicus AB176662 Flavobacteriaceae
Myroides odoratus TYGS 1065 Flavobacteriaceae
Flavobacterium rakeshii JF830803 Flavobacteriaceae
Flavobacterium beibuense GQ245972 Flavobacteriaceae
Flavobacterium hauense JX066803 Flavobacteriaceae
Flavobacterium suzhouense KM089833 Flavobacteriaceae
Flavobacterium rivuli TYGS 1274 Flavobacteriaceae
Flavobacterium subsaxonicum AM934666 Flavobacteriaceae
Flavobacterium akiainvivens TYGS 3566 Flavobacteriaceae
Flavobacterium akiainvivens TYGS 5036 Flavobacteriaceae
Flavobacterium arcticum KU529277 Flavobacteriaceae
Flavobacterium suaedae KT284905 Flavobacteriaceae
Flavobacterium lindanitolerans TYGS 22857 Flavobacteriaceae
Flavobacterium qiangtangense KF726983 Flavobacteriaceae
Flavobacterium gossypii KR047732 Flavobacteriaceae
Flavobacterium endophyticum TYGS 22862 Flavobacteriaceae
Flavobacterium filum TYGS 1445 Flavobacteriaceae
Flavobacterium keumense KC355348 Flavobacteriaceae
Flavobacterium swingsii TYGS 1932 Flavobacteriaceae
Flavobacterium psychrophilum AB078060 Flavobacteriaceae
Flavobacterium brevivitae LN849948 Flavobacteriaceae
Flavobacterium amniphilum LT555403 Flavobacteriaceae
Flavobacterium vireti KM576853 Flavobacteriaceae
Flavobacterium terrae TYGS 6198 Flavobacteriaceae
Flavobacterium lacunae LN717318 Flavobacteriaceae
Flavobacterium verecundum LN849950 Flavobacteriaceae
Flavobacterium inkyongense KX025140 Flavobacteriaceae
Flavobacterium columnare TYGS 23131 Flavobacteriaceae
Flavobacterium branchiarum HE612097 Flavobacteriaceae
Flavobacterium hydatis M58764 Flavobacteriaceae
Flavobacterium olei KX672808 Flavobacteriaceae
Flavobacterium reichenbachii TYGS 3249 Flavobacteriaceae
Flavobacterium pectinovorum AM230490 Flavobacteriaceae
Flavobacterium tructae TYGS 23114 Flavobacteriaceae
Flavobacterium spartansii TYGS 23128 Flavobacteriaceae
Flavobacterium hibernum TYGS 3247 Flavobacteriaceae
Flavobacterium plurextorum TYGS 23112 Flavobacteriaceae
Flavobacterium oncorhynchi TYGS 23126 Flavobacteriaceae
Flavobacterium branchiicola HE612102 Flavobacteriaceae
Flavobacterium chilense TYGS 14343 Flavobacteriaceae
Flavobacterium chilense TYGS 4380 Flavobacteriaceae
Flavobacterium piscis TYGS 3907 Flavobacteriaceae
Flavobacterium frigidimaris TYGS 6177 Flavobacteriaceae
Flavobacterium araucananum TYGS 6168 Flavobacteriaceae
Flavobacterium aquidurense TYGS 5037 Flavobacteriaceae
Flavobacterium chungangense TYGS 4301 Flavobacteriaceae
Flavobacterium cutihirudinis TYGS 6192 Flavobacteriaceae
Flavobacterium compostarboris GQ281769 Flavobacteriaceae
Flavobacterium dispersum LT223126 Flavobacteriaceae
Flavobacterium kyungheense JN196130 Flavobacteriaceae
Flavobacterium denitrificans TYGS 1425 Flavobacteriaceae
Flavobacterium procerum KF857168 Flavobacteriaceae
Flavobacterium fluminis KF891387 Flavobacteriaceae
Flavobacterium resistens TYGS 22227 Flavobacteriaceae
Flavobacterium arsenitoxidans JX001187 Flavobacteriaceae
Flavobacterium limi KX263321 Flavobacteriaceae
Flavobacterium hibisci KX263317 Flavobacteriaceae
Flavobacterium gyeonganense KC690142 Flavobacteriaceae
Flavobacterium quisquiliarum KU973600 Flavobacteriaceae
Flavobacterium ginsengisoli HM776705 Flavobacteriaceae
Flavobacterium phragmitis GU564236 Flavobacteriaceae
Flavobacterium nitrogenifigens TYGS 22250 Flavobacteriaceae
Flavobacterium daemonense KF532125 Flavobacteriaceae
Flavobacterium ginsenosidimutans GU138377 Flavobacteriaceae
Flavobacterium anhuiense EU046269 Flavobacteriaceae
Flavobacterium tistrianum KT223144 Flavobacteriaceae
Flavobacterium tilapiae HQ111525 Flavobacteriaceae
Flavobacterium defluvii TYGS 6175 Flavobacteriaceae
Flexibacter aurantiacus TYGS 6178 Cytophagaceae
Flavobacterium johnsoniae TYGS 6202 Flavobacteriaceae
Flavobacterium johnsoniae TYGS 979 Flavobacteriaceae
Flavobacterium tyrosinilyticum KR232271 Flavobacteriaceae
Flavobacterium banpakuense GQ281770 Flavobacteriaceae
Flavobacterium chungbukense HM627539 Flavobacteriaceae
Flavobacterium notoginsengisoli KJ130052 Flavobacteriaceae
Flavobacterium hercynium AM265623 Flavobacteriaceae
Flavobacterium saccharophilum TYGS 6210 Flavobacteriaceae
Flavobacterium collinsii HE612088 Flavobacteriaceae
Flavobacterium succinicans TYGS 5020 Flavobacteriaceae
Flavobacterium chuncheonense KX505859 Flavobacteriaceae
Flavobacterium myungsuense GQ148878 Flavobacteriaceae
Flavobacterium luteum KX505860 Flavobacteriaceae
Flavobacterium branchiophilum TYGS 23111 Flavobacteriaceae
Flavobacterium gilvum TYGS 21662 Flavobacteriaceae
Flavobacterium maotaiense KF857166 Flavobacteriaceae
Flavobacterium aquicola LC120820 Flavobacteriaceae
Flavobacterium yonginense GQ144413 Flavobacteriaceae
Flavobacterium soyangense KX061439 Flavobacteriaceae
Flavobacterium fluvii TYGS 6173 Flavobacteriaceae
Flavobacterium granuli TYGS 6144 Flavobacteriaceae
Flavobacterium granuli TYGS 6146 Flavobacteriaceae
Flavobacterium degerlachei TYGS 5013 Flavobacteriaceae
Flavobacterium frigoris TYGS 5038 Flavobacteriaceae

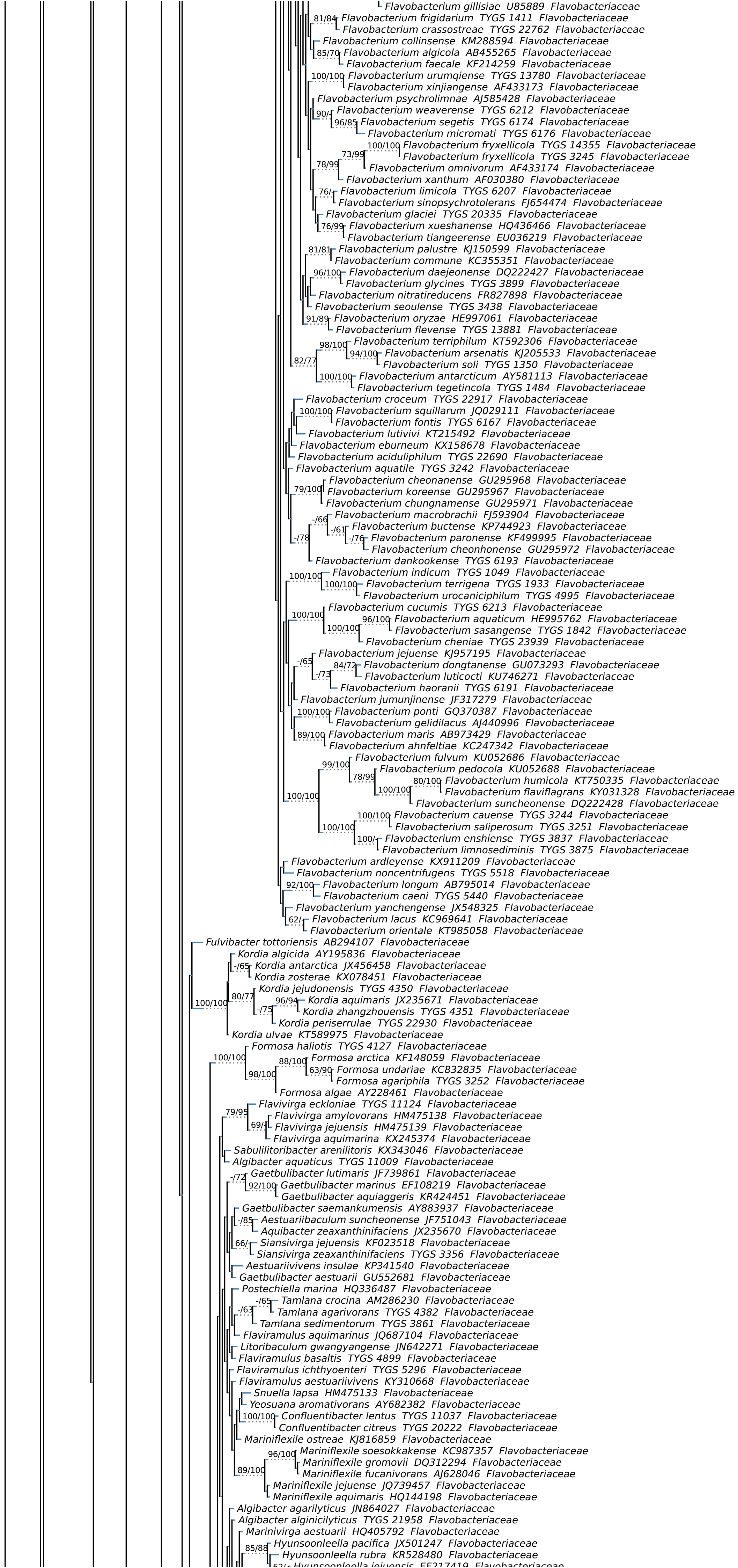

Flavobacterium gillisiae U85889 Flavobacteriaceae
81/84 Flavobacterium frigidarium TYGS 1411 Flavobacteriaceae
Flavobacterium crassostreae TYGS 22762 Flavobacteriaceae
Flavobacterium collinsense KM288594 Flavobacteriaceae
85/70 Flavobacterium algicola AB455265 Flavobacteriaceae
Flavobacterium faecale KF214259 Flavobacteriaceae
100/100 Flavobacterium urumqiense TYGS 13780 Flavobacteriaceae
Flavobacterium xinjiangense AF433173 Flavobacteriaceae
Flavobacterium psychrolimnae AJ585428 Flavobacteriaceae
90/- Flavobacterium weaverense TYGS 6212 Flavobacteriaceae
96/85 Flavobacterium segetis TYGS 6174 Flavobacteriaceae
Flavobacterium micromati TYGS 6176 Flavobacteriaceae
100/100 Flavobacterium fryxellicola TYGS 14355 Flavobacteriaceae
73/99 Flavobacterium fryxellicola TYGS 3245 Flavobacteriaceae
78/99 Flavobacterium omnivorum AF433174 Flavobacteriaceae
Flavobacterium xanthum AF030380 Flavobacteriaceae
76/- Flavobacterium limicola TYGS 6207 Flavobacteriaceae
Flavobacterium sinopsychrotolerans FJ654474 Flavobacteriaceae
Flavobacterium glaciei TYGS 20335 Flavobacteriaceae
76/99 Flavobacterium xueshanense HQ436466 Flavobacteriaceae
Flavobacterium tiangeerense EU036219 Flavobacteriaceae
81/81 Flavobacterium palustre KJ150599 Flavobacteriaceae
Flavobacterium commune KC355351 Flavobacteriaceae
96/100 Flavobacterium daejeonense DQ222427 Flavobacteriaceae
Flavobacterium glycines TYGS 3899 Flavobacteriaceae
Flavobacterium nitratireducens FR827898 Flavobacteriaceae
Flavobacterium seoulense TYGS 3438 Flavobacteriaceae
91/89 Flavobacterium oryzae HE997061 Flavobacteriaceae
Flavobacterium flevense TYGS 13881 Flavobacteriaceae
98/100 Flavobacterium terriphilum KT592306 Flavobacteriaceae
94/100 Flavobacterium arsenatis KJ205533 Flavobacteriaceae
82/77 Flavobacterium soli TYGS 1350 Flavobacteriaceae
100/100 Flavobacterium antarcticum AY581113 Flavobacteriaceae
Flavobacterium tegetincola TYGS 1484 Flavobacteriaceae
Flavobacterium croceum TYGS 22917 Flavobacteriaceae
100/100 Flavobacterium squillarum JQ029111 Flavobacteriaceae
Flavobacterium fontis TYGS 6167 Flavobacteriaceae
Flavobacterium lutivivi KT215492 Flavobacteriaceae
Flavobacterium eburneum KX158678 Flavobacteriaceae
Flavobacterium aciduliphilum TYGS 22690 Flavobacteriaceae
Flavobacterium aquatile TYGS 3242 Flavobacteriaceae
Flavobacterium cheonanense GU295968 Flavobacteriaceae
79/100 Flavobacterium koreense GU295967 Flavobacteriaceae
Flavobacterium chungnamense GU295971 Flavobacteriaceae
-/66 Flavobacterium macrobrachii FJ593904 Flavobacteriaceae
-/61 Flavobacterium buctense KP744923 Flavobacteriaceae
-/78 -/76 Flavobacterium paronense KF499995 Flavobacteriaceae
Flavobacterium cheonhonense GU295972 Flavobacteriaceae
Flavobacterium dankookense TYGS 6193 Flavobacteriaceae
100/100 Flavobacterium indicum TYGS 1049 Flavobacteriaceae
100/100 Flavobacterium terrigena TYGS 1933 Flavobacteriaceae
Flavobacterium urocaniciphilum TYGS 4995 Flavobacteriaceae
Flavobacterium cucumis TYGS 6213 Flavobacteriaceae
100/100 96/100 Flavobacterium aquaticum HE995762 Flavobacteriaceae
100/100 Flavobacterium sasangense TYGS 1842 Flavobacteriaceae
Flavobacterium cheniae TYGS 23939 Flavobacteriaceae
-/65 Flavobacterium jejuense KJ957195 Flavobacteriaceae
84/72 Flavobacterium dongtanense GU073293 Flavobacteriaceae
-/73 Flavobacterium luticocti KU746271 Flavobacteriaceae
Flavobacterium haoranii TYGS 6191 Flavobacteriaceae
Flavobacterium jumunjinense JF317279 Flavobacteriaceae
100/100 Flavobacterium ponti GQ370387 Flavobacteriaceae
Flavobacterium gelidilacus AJ440996 Flavobacteriaceae
89/100 Flavobacterium maris AB973429 Flavobacteriaceae
Flavobacterium ahnfeltiae KC247342 Flavobacteriaceae
Flavobacterium fulvum KU052686 Flavobacteriaceae
99/100 Flavobacterium pedocola KU052688 Flavobacteriaceae
78/99 80/100 Flavobacterium humicola KT750335 Flavobacteriaceae
100/100 100/100 Flavobacterium flaviflagrans KY031328 Flavobacteriaceae
Flavobacterium suncheonense DQ222428 Flavobacteriaceae
100/100 Flavobacterium cauense TYGS 3244 Flavobacteriaceae
100/100 Flavobacterium saliperosum TYGS 3251 Flavobacteriaceae
100/- Flavobacterium enshiense TYGS 3837 Flavobacteriaceae
Flavobacterium limnosediminis TYGS 3875 Flavobacteriaceae
Flavobacterium ardleyense KX911209 Flavobacteriaceae
Flavobacterium noncentrifugens TYGS 5518 Flavobacteriaceae
92/100 Flavobacterium longum AB795014 Flavobacteriaceae
Flavobacterium caeni TYGS 5440 Flavobacteriaceae
Flavobacterium yanchengense JX548325 Flavobacteriaceae
62/- Flavobacterium lacus KC969641 Flavobacteriaceae
Flavobacterium orientale KT985058 Flavobacteriaceae
Fulvibacter tottoriensis AB294107 Flavobacteriaceae
Kordia algicida AY195836 Flavobacteriaceae
-/65 Kordia antarctica JX456458 Flavobacteriaceae
Kordia zosterae KX078451 Flavobacteriaceae
Kordia jejudonensis TYGS 4350 Flavobacteriaceae
80/77 96/94 Kordia aquimaris JX235671 Flavobacteriaceae
100/100 -/75 Kordia zhangzhouensis TYGS 4351 Flavobacteriaceae
Kordia periserrulae TYGS 22930 Flavobacteriaceae
Kordia ulvae KT589975 Flavobacteriaceae
Formosa haliotis TYGS 4127 Flavobacteriaceae
100/100 88/100 Formosa arctica KF148059 Flavobacteriaceae
63/90 Formosa undariae KC832835 Flavobacteriaceae
98/100 Formosa agariphila TYGS 3252 Flavobacteriaceae
Formosa algae AY228461 Flavobacteriaceae
Flavivirga eckloniae TYGS 11124 Flavobacteriaceae
79/95 Flavivirga amylovorans HM475138 Flavobacteriaceae
69/- Flavivirga jejuensis HM475139 Flavobacteriaceae
Flavivirga aquimarina KX245374 Flavobacteriaceae
Sabulilitoribacter arenilitoris KX343046 Flavobacteriaceae
Algibacter aquaticus TYGS 11009 Flavobacteriaceae
-/72 Gaetbulibacter lutimaris JF739861 Flavobacteriaceae
92/100 Gaetbulibacter marinus EF108219 Flavobacteriaceae
Gaetbulibacter aquiaggeris KR424451 Flavobacteriaceae
Gaetbulibacter saemankumensis AY883937 Flavobacteriaceae
-/85 Aestuariibaculum suncheonense JF751043 Flavobacteriaceae
Aquibacter zeaxanthinifaciens JX235670 Flavobacteriaceae
66/- Siansivirga jejuensis KF023518 Flavobacteriaceae
Siansivirga zeaxanthinifaciens TYGS 3356 Flavobacteriaceae
Aestuariivivens insulae KP341540 Flavobacteriaceae
Gaetbulibacter aestuarii GU552681 Flavobacteriaceae
Postechiella marina HQ336487 Flavobacteriaceae
-/65 Tamlana crocina AM286230 Flavobacteriaceae
-/63 Tamlana agarivorans TYGS 4382 Flavobacteriaceae
Tamlana sedimentorum TYGS 3861 Flavobacteriaceae
Flaviramulus aquimarinus JQ687104 Flavobacteriaceae
Litoribaculum gwangyangense JN642271 Flavobacteriaceae
Flaviramulus basaltis TYGS 4899 Flavobacteriaceae
Flaviramulus ichthyoenteri TYGS 5296 Flavobacteriaceae
Flaviramulus aestuariivivens KY310668 Flavobacteriaceae
Snuella lapsa HM475133 Flavobacteriaceae
Yeosuana aromativorans AY682382 Flavobacteriaceae
100/100 Confluentibacter lentus TYGS 11037 Flavobacteriaceae
Confluentibacter citreus TYGS 20222 Flavobacteriaceae
Mariniflexile ostreae KJ816859 Flavobacteriaceae
96/100 Mariniflexile soesokkakense KC987357 Flavobacteriaceae
Mariniflexile gromovii DQ312294 Flavobacteriaceae
89/100 Mariniflexile fucanivorans AJ628046 Flavobacteriaceae
Mariniflexile jejuense JQ739457 Flavobacteriaceae
Mariniflexile aquimaris HQ144198 Flavobacteriaceae
Algibacter agarilyticus JN864027 Flavobacteriaceae
Algibacter alginicilyticus TYGS 21958 Flavobacteriaceae
Marinivirga aestuarii HQ405792 Flavobacteriaceae
85/88 Hyunsoonleella pacifica JX501247 Flavobacteriaceae
Hyunsoonleella rubra KR528480 Flavobacteriaceae

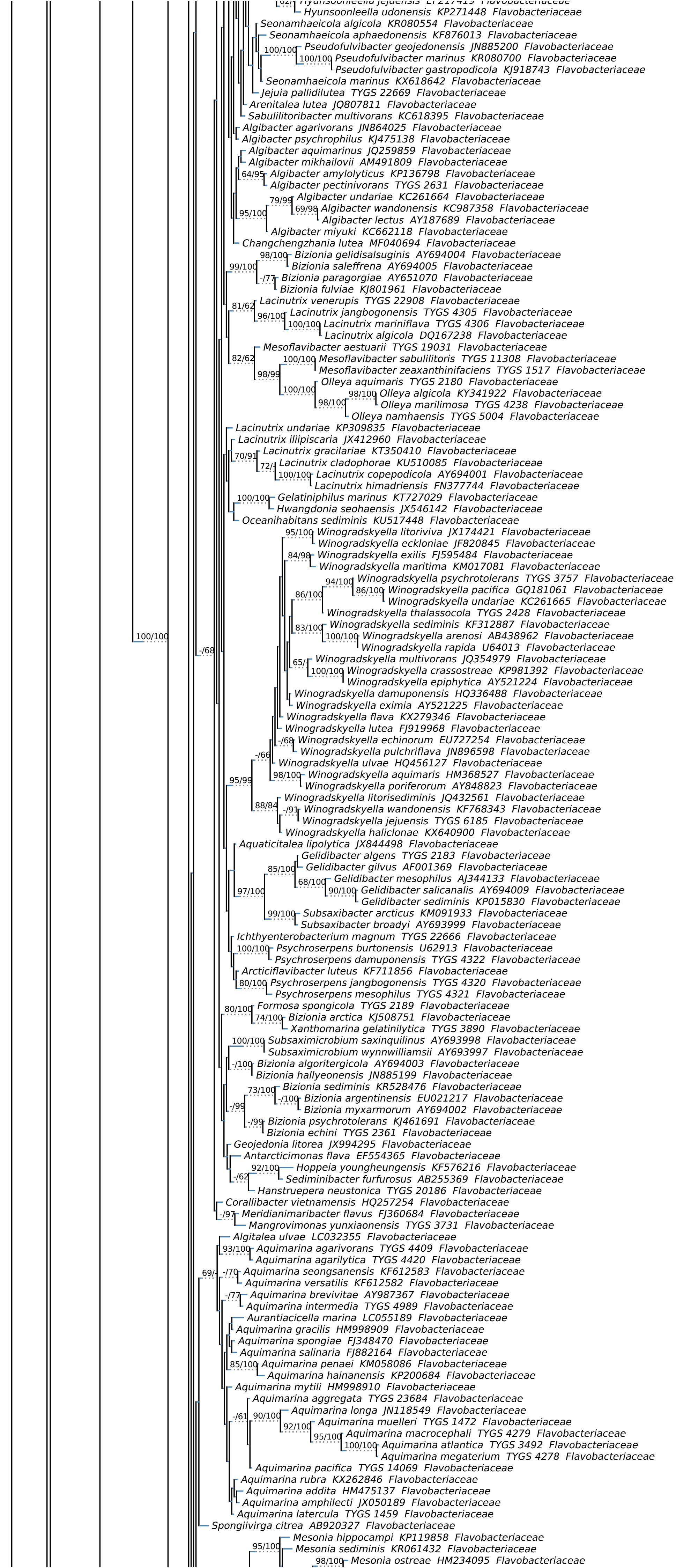
Hyunsoonleella udonensis KP271448 Flavobacteriaceae
Seonamhaeicola algicola KR080554 Flavobacteriaceae
Seonamhaeicola aphaedonensis KF876013 Flavobacteriaceae
Pseudofulvibacter geojedonensis JN885200 Flavobacteriaceae
Pseudofulvibacter marinus KR080700 Flavobacteriaceae
Pseudofulvibacter gastropodicola KJ918743 Flavobacteriaceae
Seonamhaeicola marinus KX618642 Flavobacteriaceae
Jejuia pallidilutea TYGS 22669 Flavobacteriaceae
Arenitalea lutea JQ807811 Flavobacteriaceae
Sabulilitoribacter multivorans KC618395 Flavobacteriaceae
Algibacter agarivorans JN864025 Flavobacteriaceae
Algibacter psychrophilus KJ475138 Flavobacteriaceae
Algibacter aquimarinus JQ259859 Flavobacteriaceae
Algibacter mikhailovii AM491809 Flavobacteriaceae
Algibacter amylolyticus KP136798 Flavobacteriaceae
Algibacter pectinivorans TYGS 2631 Flavobacteriaceae
Algibacter undariae KC261664 Flavobacteriaceae
Algibacter wandonensis KC987358 Flavobacteriaceae
Algibacter lectus AY187689 Flavobacteriaceae
Algibacter miyuki KC662118 Flavobacteriaceae
Changchengzhania lutea MF040694 Flavobacteriaceae
Bizionia gelidisalsuginis AY694004 Flavobacteriaceae
Bizionia saleffrena AY694005 Flavobacteriaceae
Bizionia paragorgiae AY651070 Flavobacteriaceae
Bizionia fulviae KJ801961 Flavobacteriaceae
Lacinutrix venerupis TYGS 22908 Flavobacteriaceae
Lacinutrix jangbogonensis TYGS 4305 Flavobacteriaceae
Lacinutrix mariniflava TYGS 4306 Flavobacteriaceae
Lacinutrix algicola DQ167238 Flavobacteriaceae
Mesoflavibacter aestuarii TYGS 19031 Flavobacteriaceae
Mesoflavibacter sabulilitoris TYGS 11308 Flavobacteriaceae
Mesoflavibacter zeaxanthinifaciens TYGS 1517 Flavobacteriaceae
Olleya aquimaris TYGS 2180 Flavobacteriaceae
Olleya algicola KY341922 Flavobacteriaceae
Olleya marilimosa TYGS 4238 Flavobacteriaceae
Olleya namhaensis TYGS 5004 Flavobacteriaceae
Lacinutrix undariae KP309835 Flavobacteriaceae
Lacinutrix iliipiscaria JX412960 Flavobacteriaceae
Lacinutrix gracilariae KT350410 Flavobacteriaceae
Lacinutrix cladophorae KU510085 Flavobacteriaceae
Lacinutrix copepodicola AY694001 Flavobacteriaceae
Lacinutrix himadriensis FN377744 Flavobacteriaceae
Gelatiniphilus marinus KT727029 Flavobacteriaceae
Hwangdonia seohaensis JX546142 Flavobacteriaceae
Oceanihabitans sediminis KU517448 Flavobacteriaceae
Winogradskyella litoriviva JX174421 Flavobacteriaceae
Winogradskyella eckloniae JF820845 Flavobacteriaceae
Winogradskyella exilis FJ595484 Flavobacteriaceae
Winogradskyella maritima KM017081 Flavobacteriaceae
Winogradskyella psychrotolerans TYGS 3757 Flavobacteriaceae
Winogradskyella pacifica GQ181061 Flavobacteriaceae
Winogradskyella undariae KC261665 Flavobacteriaceae
Winogradskyella thalassocola TYGS 2428 Flavobacteriaceae
Winogradskyella sediminis KF312887 Flavobacteriaceae
Winogradskyella arenosi AB438962 Flavobacteriaceae
Winogradskyella rapida U64013 Flavobacteriaceae
Winogradskyella multivorans JQ354979 Flavobacteriaceae
Winogradskyella crassostreae KP981392 Flavobacteriaceae
Winogradskyella epiphytica AY521224 Flavobacteriaceae
Winogradskyella damuponensis HQ336488 Flavobacteriaceae
Winogradskyella eximia AY521225 Flavobacteriaceae
Winogradskyella flava KX279346 Flavobacteriaceae
Winogradskyella lutea FJ919968 Flavobacteriaceae
Winogradskyella echinorum EU727254 Flavobacteriaceae
Winogradskyella pulchriflava JN896598 Flavobacteriaceae
Winogradskyella ulvae HQ456127 Flavobacteriaceae
Winogradskyella aquimaris HM368527 Flavobacteriaceae
Winogradskyella poriferorum AY848823 Flavobacteriaceae
Winogradskyella litorisediminis JQ432561 Flavobacteriaceae
Winogradskyella wandonensis KF768343 Flavobacteriaceae
Winogradskyella jejuensis TYGS 6185 Flavobacteriaceae
Winogradskyella haliclonae KX640900 Flavobacteriaceae
Aquaticitalea lipolytica JX844498 Flavobacteriaceae
Gelidibacter algens TYGS 2183 Flavobacteriaceae
Gelidibacter gilvus AF001369 Flavobacteriaceae
Gelidibacter mesophilus AJ344133 Flavobacteriaceae
Gelidibacter salicanalis AY694009 Flavobacteriaceae
Gelidibacter sediminis KP015830 Flavobacteriaceae
Subsaxibacter arcticus KM091933 Flavobacteriaceae
Subsaxibacter broadyi AY693999 Flavobacteriaceae
Ichthyenterobacterium magnum TYGS 22666 Flavobacteriaceae
Psychroserpens burtonensis U62913 Flavobacteriaceae
Psychroserpens damuponensis TYGS 4322 Flavobacteriaceae
Arcticiflavibacter luteus KF711856 Flavobacteriaceae
Psychroserpens jangbogonensis TYGS 4320 Flavobacteriaceae
Psychroserpens mesophilus TYGS 4321 Flavobacteriaceae
Formosa spongicola TYGS 2189 Flavobacteriaceae
Bizionia arctica KJ508751 Flavobacteriaceae
Xanthomarina gelatinilytica TYGS 3890 Flavobacteriaceae
Subsaximicrobium saxinquilinus AY693998 Flavobacteriaceae
Subsaximicrobium wynnwilliamsii AY693997 Flavobacteriaceae
Bizionia algoritergicola AY694003 Flavobacteriaceae
Bizionia hallyeonensis JN885199 Flavobacteriaceae
Bizionia sediminis KR528476 Flavobacteriaceae
Bizionia argentinensis EU021217 Flavobacteriaceae
Bizionia myxarmorum AY694002 Flavobacteriaceae
Bizionia psychrotolerans KJ461691 Flavobacteriaceae
Bizionia echini TYGS 2361 Flavobacteriaceae
Geojedonia litorea JX994295 Flavobacteriaceae
Antarcticimonas flava EF554365 Flavobacteriaceae
Hoppeia youngheungensis KF576216 Flavobacteriaceae
Sediminibacter furfurosus AB255369 Flavobacteriaceae
Hanstruepera neustonica TYGS 20186 Flavobacteriaceae
Corallibacter vietnamensis HQ257254 Flavobacteriaceae
Meridianimaribacter flavus FJ360684 Flavobacteriaceae
Mangrovimonas yunxiaonensis TYGS 3731 Flavobacteriaceae
Algitalea ulvae LC032355 Flavobacteriaceae
Aquimarina agarivorans TYGS 4409 Flavobacteriaceae
Aquimarina agarilytica TYGS 4420 Flavobacteriaceae
Aquimarina seongsanensis KF612583 Flavobacteriaceae
Aquimarina versatilis KF612582 Flavobacteriaceae
Aquimarina brevivitae AY987367 Flavobacteriaceae
Aquimarina intermedia TYGS 4989 Flavobacteriaceae
Aurantiacicella marina LC055189 Flavobacteriaceae
Aquimarina gracilis HM998909 Flavobacteriaceae
Aquimarina spongiae FJ348470 Flavobacteriaceae
Aquimarina salinaria FJ882164 Flavobacteriaceae
Aquimarina penaei KM058086 Flavobacteriaceae
Aquimarina hainanensis KP200684 Flavobacteriaceae
Aquimarina mytili HM998910 Flavobacteriaceae
Aquimarina aggregata TYGS 23684 Flavobacteriaceae
Aquimarina longa JN118549 Flavobacteriaceae
Aquimarina muelleri TYGS 1472 Flavobacteriaceae
Aquimarina macrocephali TYGS 4279 Flavobacteriaceae
Aquimarina atlantica TYGS 3492 Flavobacteriaceae
Aquimarina megaterium TYGS 4278 Flavobacteriaceae
Aquimarina pacifica TYGS 14069 Flavobacteriaceae
Aquimarina rubra KX262846 Flavobacteriaceae
Aquimarina addita HM475137 Flavobacteriaceae
Aquimarina amphilecti JX050189 Flavobacteriaceae
Aquimarina latercula TYGS 1459 Flavobacteriaceae
Spongiivirga citrea AB920327 Flavobacteriaceae
Mesonia hippocampi KP119858 Flavobacteriaceae
Mesonia sediminis KR061432 Flavobacteriaceae
Mesonia ostreae HM234095 Flavobacteriaceae

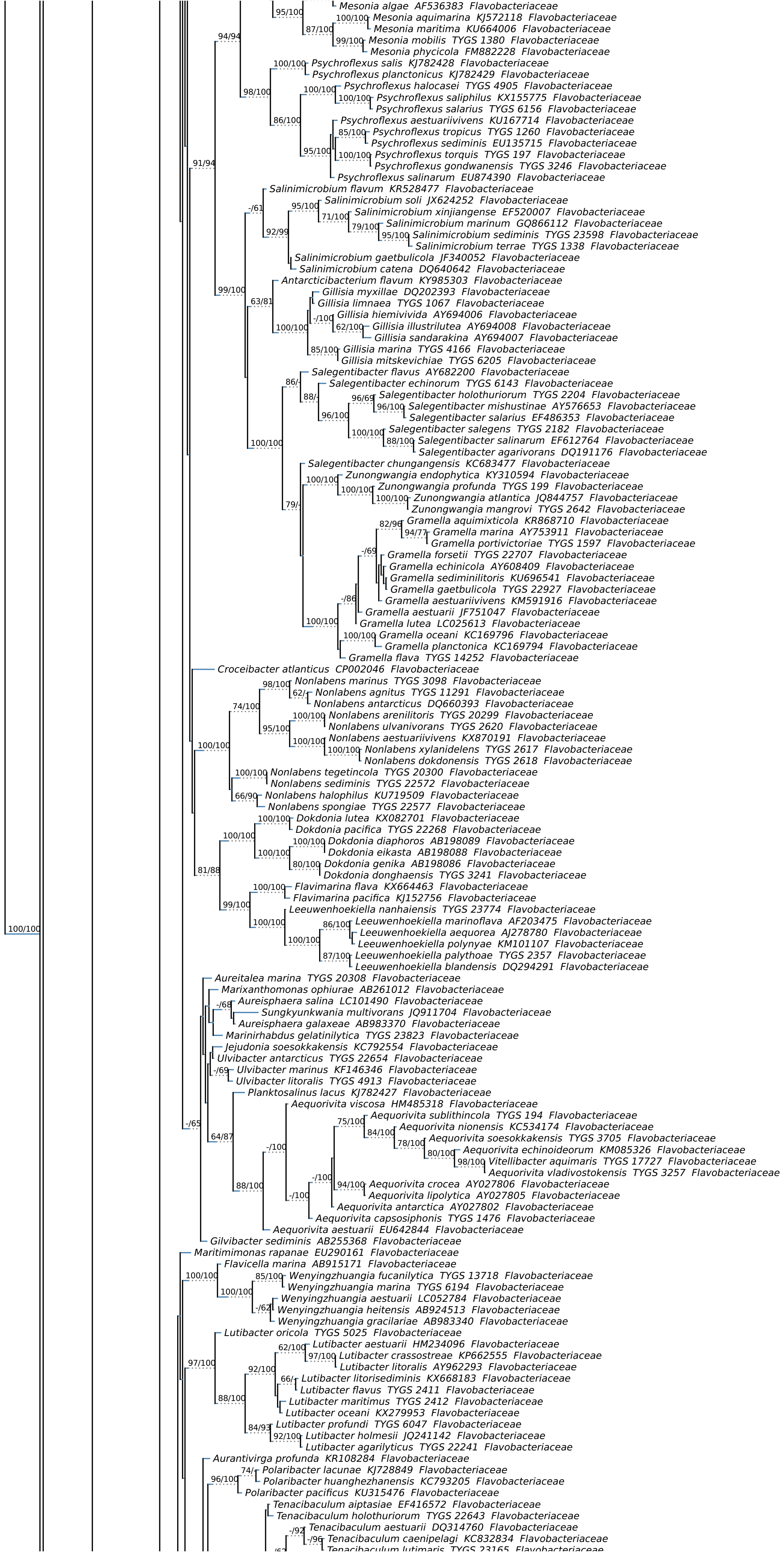

Mesonia algae AF536383 Flavobacteriaceae
Mesonia aquimarina KJ572118 Flavobacteriaceae
Mesonia maritima KU664006 Flavobacteriaceae
Mesonia mobilis TYGS 1380 Flavobacteriaceae
Mesonia phycicola FM882228 Flavobacteriaceae
Psychroflexus salis KJ782428 Flavobacteriaceae
Psychroflexus planctonicus KJ782429 Flavobacteriaceae
Psychroflexus halocasei TYGS 4905 Flavobacteriaceae
Psychroflexus saliphilus KX155775 Flavobacteriaceae
Psychroflexus salarius TYGS 6156 Flavobacteriaceae
Psychroflexus aestuariivivens KU167714 Flavobacteriaceae
Psychroflexus tropicus TYGS 1260 Flavobacteriaceae
Psychroflexus sediminis EU135715 Flavobacteriaceae
Psychroflexus torquis TYGS 197 Flavobacteriaceae
Psychroflexus gondwanensis TYGS 3246 Flavobacteriaceae
Psychroflexus salinarum EU874390 Flavobacteriaceae
Salinimicrobium flavum KR528477 Flavobacteriaceae
Salinimicrobium soli JX624252 Flavobacteriaceae
Salinimicrobium xinjiangense EF520007 Flavobacteriaceae
Salinimicrobium marinum GQ866112 Flavobacteriaceae
Salinimicrobium sediminis TYGS 23598 Flavobacteriaceae
Salinimicrobium terrae TYGS 1338 Flavobacteriaceae
Salinimicrobium gaetbulicola JF340052 Flavobacteriaceae
Salinimicrobium catena DQ640642 Flavobacteriaceae
Antarcticibacterium flavum KY985303 Flavobacteriaceae
Gillisia myxillae DQ202393 Flavobacteriaceae
Gillisia limnaea TYGS 1067 Flavobacteriaceae
Gillisia hiemivivida AY694006 Flavobacteriaceae
Gillisia illustrilutea AY694008 Flavobacteriaceae
Gillisia sandarakina AY694007 Flavobacteriaceae
Gillisia marina TYGS 4166 Flavobacteriaceae
Gillisia mitskevichiae TYGS 6205 Flavobacteriaceae
Salegentibacter flavus AY682200 Flavobacteriaceae
Salegentibacter echinorum TYGS 6143 Flavobacteriaceae
Salegentibacter holothuriorum TYGS 2204 Flavobacteriaceae
Salegentibacter mishustinae AY576653 Flavobacteriaceae
Salegentibacter salarius EF486353 Flavobacteriaceae
Salegentibacter salegens TYGS 2182 Flavobacteriaceae
Salegentibacter salinarum EF612764 Flavobacteriaceae
Salegentibacter agarivorans DQ191176 Flavobacteriaceae
Salegentibacter chungangensis KC683477 Flavobacteriaceae
Zunongwangia endophytica KY310594 Flavobacteriaceae
Zunongwangia profunda TYGS 199 Flavobacteriaceae
Zunongwangia atlantica JQ844757 Flavobacteriaceae
Zunongwangia mangrovi TYGS 2642 Flavobacteriaceae
Gramella aquimixticola KR868710 Flavobacteriaceae
Gramella marina AY753911 Flavobacteriaceae
Gramella portivictoriae TYGS 1597 Flavobacteriaceae
Gramella forsetii TYGS 22707 Flavobacteriaceae
Gramella echinicola AY608409 Flavobacteriaceae
Gramella sediminilitoris KU696541 Flavobacteriaceae
Gramella gaetbulicola TYGS 22927 Flavobacteriaceae
Gramella aestuariivivens KM591916 Flavobacteriaceae
Gramella aestuarii JF751047 Flavobacteriaceae
Gramella lutea LC025613 Flavobacteriaceae
Gramella oceani KC169796 Flavobacteriaceae
Gramella planctonica KC169794 Flavobacteriaceae
Gramella flava TYGS 14252 Flavobacteriaceae
Croceibacter atlanticus CP002046 Flavobacteriaceae
Nonlabens marinus TYGS 3098 Flavobacteriaceae
Nonlabens agnitus TYGS 11291 Flavobacteriaceae
Nonlabens antarcticus DQ660393 Flavobacteriaceae
Nonlabens arenilitoris TYGS 20299 Flavobacteriaceae
Nonlabens ulvanivorans TYGS 2620 Flavobacteriaceae
Nonlabens aestuariivivens KX870191 Flavobacteriaceae
Nonlabens xylanidelens TYGS 2617 Flavobacteriaceae
Nonlabens dokdonensis TYGS 2618 Flavobacteriaceae
Nonlabens tegetincola TYGS 20300 Flavobacteriaceae
Nonlabens sediminis TYGS 22572 Flavobacteriaceae
Nonlabens halophilus KU719509 Flavobacteriaceae
Nonlabens spongiae TYGS 22577 Flavobacteriaceae
Dokdonia lutea KX082701 Flavobacteriaceae
Dokdonia pacifica TYGS 22268 Flavobacteriaceae
Dokdonia diaphoros AB198089 Flavobacteriaceae
Dokdonia eikasta AB198088 Flavobacteriaceae
Dokdonia genika AB198086 Flavobacteriaceae
Dokdonia donghaensis TYGS 3241 Flavobacteriaceae
Flavimarina flava KX664463 Flavobacteriaceae
Flavimarina pacifica KJ152756 Flavobacteriaceae
Leeuwenhoekiella nanhaiensis TYGS 23774 Flavobacteriaceae
Leeuwenhoekiella marinoflava AF203475 Flavobacteriaceae
Leeuwenhoekiella aequorea AJ278780 Flavobacteriaceae
Leeuwenhoekiella polynyae KM101107 Flavobacteriaceae
Leeuwenhoekiella palythoae TYGS 2357 Flavobacteriaceae
Leeuwenhoekiella blandensis DQ294291 Flavobacteriaceae
Aureitalea marina TYGS 20308 Flavobacteriaceae
Marixanthomonas ophiurae AB261012 Flavobacteriaceae
Aureisphaera salina LC101490 Flavobacteriaceae
Sungkyunkwania multivorans JQ911704 Flavobacteriaceae
Aureisphaera galaxeae AB983370 Flavobacteriaceae
Marinirhabdus gelatinilytica TYGS 23823 Flavobacteriaceae
Jejudonia soesokkakensis KC792554 Flavobacteriaceae
Ulvibacter antarcticus TYGS 22654 Flavobacteriaceae
Ulvibacter marinus KF146346 Flavobacteriaceae
Ulvibacter litoralis TYGS 4913 Flavobacteriaceae
Planktosalinus lacus KJ782427 Flavobacteriaceae
Aequorivita viscosa HM485318 Flavobacteriaceae
Aequorivita sublithincola TYGS 194 Flavobacteriaceae
Aequorivita nionensis KC534174 Flavobacteriaceae
Aequorivita soesokkakensis TYGS 3705 Flavobacteriaceae
Aequorivita echinoideorum KM085326 Flavobacteriaceae
Vitellibacter aquimaris TYGS 17727 Flavobacteriaceae
Aequorivita vladivostokensis TYGS 3257 Flavobacteriaceae
Aequorivita crocea AY027806 Flavobacteriaceae
Aequorivita lipolytica AY027805 Flavobacteriaceae
Aequorivita antarctica AY027802 Flavobacteriaceae
Aequorivita capsosiphonis TYGS 1476 Flavobacteriaceae
Aequorivita aestuarii EU642844 Flavobacteriaceae
Gilvibacter sediminis AB255368 Flavobacteriaceae
Maritimimonas rapanae EU290161 Flavobacteriaceae
Flavicella marina AB915171 Flavobacteriaceae
Wenyingzhuangia fucanilytica TYGS 13718 Flavobacteriaceae
Wenyingzhuangia marina TYGS 6194 Flavobacteriaceae
Wenyingzhuangia aestuarii LC052784 Flavobacteriaceae
Wenyingzhuangia heitensis AB924513 Flavobacteriaceae
Wenyingzhuangia gracilariae AB983340 Flavobacteriaceae
Lutibacter oricola TYGS 5025 Flavobacteriaceae
Lutibacter aestuarii HM234096 Flavobacteriaceae
Lutibacter crassostreae KP662555 Flavobacteriaceae
Lutibacter litoralis AY962293 Flavobacteriaceae
Lutibacter litorisediminis KX668183 Flavobacteriaceae
Lutibacter flavus TYGS 2411 Flavobacteriaceae
Lutibacter maritimus TYGS 2412 Flavobacteriaceae
Lutibacter oceani KX279953 Flavobacteriaceae
Lutibacter profundi TYGS 6047 Flavobacteriaceae
Lutibacter holmesii JQ241142 Flavobacteriaceae
Lutibacter agarilyticus TYGS 22241 Flavobacteriaceae
Aurantivirga profunda KR108284 Flavobacteriaceae
Polaribacter lacunae KJ728849 Flavobacteriaceae
Polaribacter huanghezhanensis KC793205 Flavobacteriaceae
Polaribacter pacificus KU315476 Flavobacteriaceae
Tenacibaculum aiptasiae EF416572 Flavobacteriaceae
Tenacibaculum holothuriorum TYGS 22643 Flavobacteriaceae
Tenacibaculum aestuarii DQ314760 Flavobacteriaceae
Tenacibaculum caenipelagi KC832834 Flavobacteriaceae
Tenacibaculum lutimaris TYGS 23165 Flavobacteriaceae

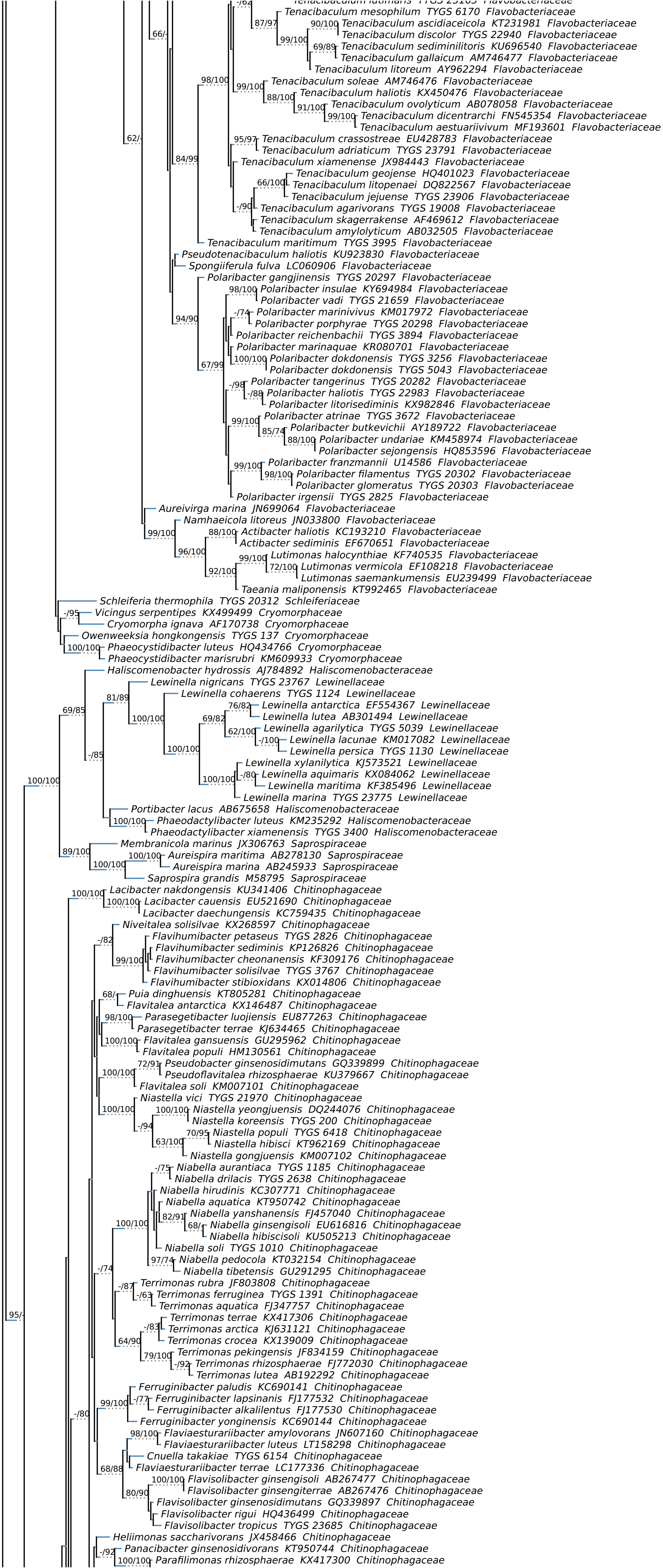
Tenacibaculum mesophilum TYGS 6170 Flavobacteriaceae
Tenacibaculum ascidiaceicola KT231981 Flavobacteriaceae
Tenacibaculum discolor TYGS 22940 Flavobacteriaceae
Tenacibaculum sediminilitoris KU696540 Flavobacteriaceae
Tenacibaculum gallaicum AM746477 Flavobacteriaceae
Tenacibaculum litoreum AY962294 Flavobacteriaceae
Tenacibaculum soleae AM746476 Flavobacteriaceae
Tenacibaculum haliotis KX450476 Flavobacteriaceae
Tenacibaculum ovolyticum AB078058 Flavobacteriaceae
Tenacibaculum dicentrarchi FN545354 Flavobacteriaceae
Tenacibaculum aestuariivivum MF193601 Flavobacteriaceae
Tenacibaculum crassostreae EU428783 Flavobacteriaceae
Tenacibaculum adriaticum TYGS 23791 Flavobacteriaceae
Tenacibaculum xiamenense JX984443 Flavobacteriaceae
Tenacibaculum geojense HQ401023 Flavobacteriaceae
Tenacibaculum litopenaei DQ822567 Flavobacteriaceae
Tenacibaculum jejuense TYGS 23906 Flavobacteriaceae
Tenacibaculum agarivorans TYGS 19008 Flavobacteriaceae
Tenacibaculum skagerrakense AF469612 Flavobacteriaceae
Tenacibaculum amylolyticum AB032505 Flavobacteriaceae
Tenacibaculum maritimum TYGS 3995 Flavobacteriaceae
Pseudotenacibaculum haliotis KU923830 Flavobacteriaceae
Spongiiferula fulva LC060906 Flavobacteriaceae
Polaribacter gangjinensis TYGS 20297 Flavobacteriaceae
Polaribacter insulae KY694984 Flavobacteriaceae
Polaribacter vadi TYGS 21659 Flavobacteriaceae
Polaribacter marinivivus KM017972 Flavobacteriaceae
Polaribacter porphyrae TYGS 20298 Flavobacteriaceae
Polaribacter reichenbachii TYGS 3894 Flavobacteriaceae
Polaribacter marinaquae KR080701 Flavobacteriaceae
Polaribacter dokdonensis TYGS 3256 Flavobacteriaceae
Polaribacter dokdonensis TYGS 5043 Flavobacteriaceae
Polaribacter tangerinus TYGS 20282 Flavobacteriaceae
Polaribacter haliotis TYGS 22983 Flavobacteriaceae
Polaribacter litorisediminis KX982846 Flavobacteriaceae
Polaribacter atrinae TYGS 3672 Flavobacteriaceae
Polaribacter butkevichii AY189722 Flavobacteriaceae
Polaribacter undariae KM458974 Flavobacteriaceae
Polaribacter sejongensis HQ853596 Flavobacteriaceae
Polaribacter franzmannii U14586 Flavobacteriaceae
Polaribacter filamentus TYGS 20302 Flavobacteriaceae
Polaribacter glomeratus TYGS 20303 Flavobacteriaceae
Polaribacter irgensii TYGS 2825 Flavobacteriaceae
Aureivirga marina JN699064 Flavobacteriaceae
Namhaeicola litoreus JN033800 Flavobacteriaceae
Actibacter haliotis KC193210 Flavobacteriaceae
Actibacter sediminis EF670651 Flavobacteriaceae
Lutimonas halocynthiae KF740535 Flavobacteriaceae
Lutimonas vermicola EF108218 Flavobacteriaceae
Lutimonas saemankumensis EU239499 Flavobacteriaceae
Taeania maliponensis KT992465 Flavobacteriaceae
Schleiferia thermophila TYGS 20312 Schleiferiaceae
Vicingus serpentipes KX499499 Cryomorphaceae
Cryomorpha ignava AF170738 Cryomorphaceae
Owenweeksia hongkongensis TYGS 137 Cryomorphaceae
Phaeocystidibacter luteus HQ434766 Cryomorphaceae
Phaeocystidibacter marisrubri KM609933 Cryomorphaceae
Haliscomenobacter hydrossis AJ784892 Haliscomenobacteraceae
Lewinella nigricans TYGS 23767 Lewinellaceae
Lewinella cohaerens TYGS 1124 Lewinellaceae
Lewinella antarctica EF554367 Lewinellaceae
Lewinella lutea AB301494 Lewinellaceae
Lewinella agarilytica TYGS 5039 Lewinellaceae
Lewinella lacunae KM017082 Lewinellaceae
Lewinella persica TYGS 1130 Lewinellaceae
Lewinella xylanilytica KJ573521 Lewinellaceae
Lewinella aquimaris KX084062 Lewinellaceae
Lewinella maritima KF385496 Lewinellaceae
Lewinella marina TYGS 23775 Lewinellaceae
Portibacter lacus AB675658 Haliscomenobacteraceae
Phaeodactylibacter luteus KM235292 Haliscomenobacteraceae
Phaeodactylibacter xiamenensis TYGS 3400 Haliscomenobacteraceae
Membranicola marinus JX306763 Saprospiraceae
Aureispira maritima AB278130 Saprospiraceae
Aureispira marina AB245933 Saprospiraceae
Saprospira grandis M58795 Saprospiraceae
Lacibacter nakdongensis KU341406 Chitinophagaceae
Lacibacter cauensis EU521690 Chitinophagaceae
Lacibacter daechungensis KC759435 Chitinophagaceae
Niveitalea solisilvae KX268597 Chitinophagaceae
Flavihumibacter petaseus TYGS 2826 Chitinophagaceae
Flavihumibacter sediminis KP126826 Chitinophagaceae
Flavihumibacter cheonanensis KF309176 Chitinophagaceae
Flavihumibacter solisilvae TYGS 3767 Chitinophagaceae
Flavihumibacter stibioxidans KX014806 Chitinophagaceae
Puia dinghuensis KT805281 Chitinophagaceae
Flavitalea antarctica KX146487 Chitinophagaceae
Parasegetibacter luojiensis EU877263 Chitinophagaceae
Parasegetibacter terrae KJ634465 Chitinophagaceae
Flavitalea gansuensis GU295962 Chitinophagaceae
Flavitalea populi HM130561 Chitinophagaceae
Pseudobacter ginsenosidimutans GQ339899 Chitinophagaceae
Pseudoflavitalea rhizosphaerae KU379667 Chitinophagaceae
Flavitalea soli KM007101 Chitinophagaceae
Niastella vici TYGS 21970 Chitinophagaceae
Niastella yeongjuensis DQ244076 Chitinophagaceae
Niastella koreensis TYGS 200 Chitinophagaceae
Niastella populi TYGS 6418 Chitinophagaceae
Niastella hibisci KT962169 Chitinophagaceae
Niastella gongjuensis KM007102 Chitinophagaceae
Niabella aurantiaca TYGS 1185 Chitinophagaceae
Niabella drilacis TYGS 2638 Chitinophagaceae
Niabella hirudinis KC307771 Chitinophagaceae
Niabella aquatica KT950742 Chitinophagaceae
Niabella yanshanensis FJ457040 Chitinophagaceae
Niabella ginsengisoli EU616816 Chitinophagaceae
Niabella hibiscisoli KU505213 Chitinophagaceae
Niabella soli TYGS 1010 Chitinophagaceae
Niabella pedocola KT032154 Chitinophagaceae
Niabella tibetensis GU291295 Chitinophagaceae
Terrimonas rubra JF803808 Chitinophagaceae
Terrimonas ferruginea TYGS 1391 Chitinophagaceae
Terrimonas aquatica FJ347757 Chitinophagaceae
Terrimonas terrae KX417306 Chitinophagaceae
Terrimonas arctica KJ631121 Chitinophagaceae
Terrimonas crocea KX139009 Chitinophagaceae
Terrimonas pekingensis JF834159 Chitinophagaceae
Terrimonas rhizosphaerae FJ772030 Chitinophagaceae
Terrimonas lutea AB192292 Chitinophagaceae
Ferruginibacter paludis KC690141 Chitinophagaceae
Ferruginibacter lapsinanis FJ177532 Chitinophagaceae
Ferruginibacter alkalilentus FJ177530 Chitinophagaceae
Ferruginibacter yonginensis KC690144 Chitinophagaceae
Flaviaesturariibacter amylovorans JN607160 Chitinophagaceae
Flaviaesturariibacter luteus LT158298 Chitinophagaceae
Cnuella takakiae TYGS 6154 Chitinophagaceae
Flaviaesturariibacter terrae LC177336 Chitinophagaceae
Flavisolibacter ginsengisoli AB267477 Chitinophagaceae
Flavisolibacter ginsengiterrae AB267476 Chitinophagaceae
Flavisolibacter ginsenosidimutans GQ339897 Chitinophagaceae
Flavisolibacter rigui HQ436499 Chitinophagaceae
Flavisolibacter tropicus TYGS 23685 Chitinophagaceae
Heliimonas saccharivorans JX458466 Chitinophagaceae
Panacibacter ginsenosidivorans KT950744 Chitinophagaceae
Parafilimonas rhizosphaerae KX417300 Chitinophagaceae

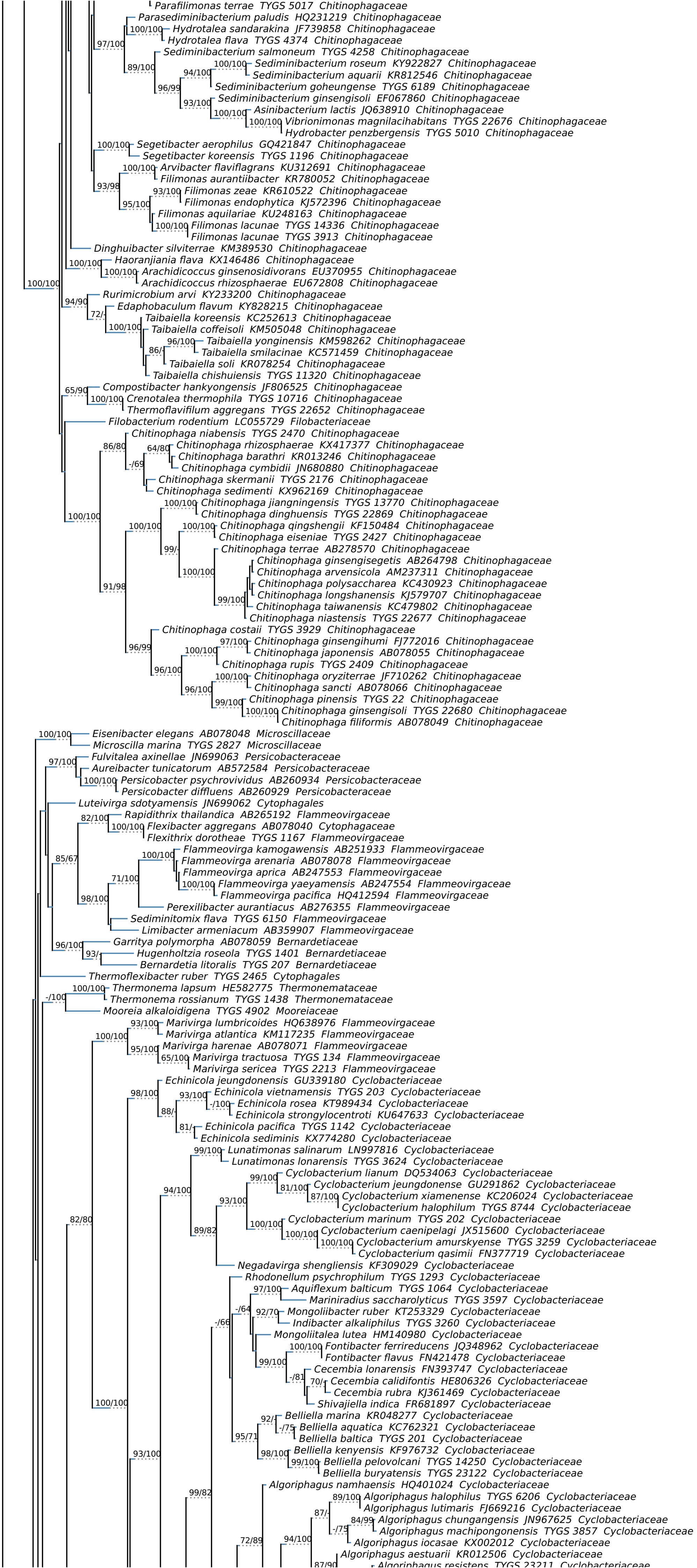
Parafilimonas terrae TYGS 5017 Chitinophagaceae
Parasediminibacterium paludis HQ231219 Chitinophagaceae
Hydrotalea sandarakina JF739858 Chitinophagaceae
Hydrotalea flava TYGS 4374 Chitinophagaceae
Sediminibacterium salmoneum TYGS 4258 Chitinophagaceae
Sediminibacterium roseum KY922827 Chitinophagaceae
Sediminibacterium aquarii KR812546 Chitinophagaceae
Sediminibacterium goheungense TYGS 6189 Chitinophagaceae
Sediminibacterium ginsengisoli EF067860 Chitinophagaceae
Asinibacterium lactis JQ638910 Chitinophagaceae
Vibrionimonas magnilacihabitans TYGS 22676 Chitinophagaceae
Hydrobacter penzbergensis TYGS 5010 Chitinophagaceae
Segetibacter aerophilus GQ421847 Chitinophagaceae
Segetibacter koreensis TYGS 1196 Chitinophagaceae
Arvibacter flaviflagrans KU312691 Chitinophagaceae
Filimonas aurantiibacter KR780052 Chitinophagaceae
Filimonas zeae KR610522 Chitinophagaceae
Filimonas endophytica KJ572396 Chitinophagaceae
Filimonas aquilariae KU248163 Chitinophagaceae
Filimonas lacunae TYGS 14336 Chitinophagaceae
Filimonas lacunae TYGS 3913 Chitinophagaceae
Dinghuibacter silviterrae KM389530 Chitinophagaceae
Haoranjiania flava KX146486 Chitinophagaceae
Arachidicoccus ginsenosidivorans EU370955 Chitinophagaceae
Arachidicoccus rhizosphaerae EU672808 Chitinophagaceae
Rurimicrobium arvi KY233200 Chitinophagaceae
Edaphobaculum flavum KY828215 Chitinophagaceae
Taibaiella koreensis KC252613 Chitinophagaceae
Taibaiella coffeisoli KM505048 Chitinophagaceae
Taibaiella yonginensis KM598262 Chitinophagaceae
Taibaiella smilacinae KC571459 Chitinophagaceae
Taibaiella soli KR078254 Chitinophagaceae
Taibaiella chishuiensis TYGS 11320 Chitinophagaceae
Compostibacter hankyongensis JF806525 Chitinophagaceae
Crenotalea thermophila TYGS 10716 Chitinophagaceae
Thermoflavifilum aggregans TYGS 22652 Chitinophagaceae
Filobacterium rodentium LC055729 Filobacteriaceae
Chitinophaga niabensis TYGS 2470 Chitinophagaceae
Chitinophaga rhizosphaerae KX417377 Chitinophagaceae
Chitinophaga barathri KR013246 Chitinophagaceae
Chitinophaga cymbidii JN680880 Chitinophagaceae
Chitinophaga skermanii TYGS 2176 Chitinophagaceae
Chitinophaga sedimenti KX962169 Chitinophagaceae
Chitinophaga jiangningensis TYGS 13770 Chitinophagaceae
Chitinophaga dinghuensis TYGS 22869 Chitinophagaceae
Chitinophaga qingshengii KF150484 Chitinophagaceae
Chitinophaga eiseniae TYGS 2427 Chitinophagaceae
Chitinophaga terrae AB278570 Chitinophagaceae
Chitinophaga ginsengisegetis AB264798 Chitinophagaceae
Chitinophaga arvensicola AM237311 Chitinophagaceae
Chitinophaga polysaccharea KC430923 Chitinophagaceae
Chitinophaga longshanensis KJ579707 Chitinophagaceae
Chitinophaga taiwanensis KC479802 Chitinophagaceae
Chitinophaga niastensis TYGS 22677 Chitinophagaceae
Chitinophaga costaii TYGS 3929 Chitinophagaceae
Chitinophaga ginsengihumi FJ772016 Chitinophagaceae
Chitinophaga japonensis AB078055 Chitinophagaceae
Chitinophaga rupis TYGS 2409 Chitinophagaceae
Chitinophaga oryziterrae JF710262 Chitinophagaceae
Chitinophaga sancti AB078066 Chitinophagaceae
Chitinophaga pinensis TYGS 22 Chitinophagaceae
Chitinophaga ginsengisoli TYGS 22680 Chitinophagaceae
Chitinophaga filiformis AB078049 Chitinophagaceae
Eisenibacter elegans AB078048 Microscillaceae
Microscilla marina TYGS 2827 Microscillaceae
Fulvitalea axinellae JN699063 Persicobacteraceae
Aureibacter tunicatorum AB572584 Persicobacteraceae
Persicobacter psychrovividus AB260934 Persicobacteraceae
Persicobacter diffluens AB260929 Persicobacteraceae
Luteivirga sdotyamensis JN699062 Cytophagales
Rapidithrix thailandica AB265192 Flammeovirgaceae
Flexibacter aggregans AB078040 Cytophagaceae
Flexithrix dorotheae TYGS 1167 Flammeovirgaceae
Flammeovirga kamogawensis AB251933 Flammeovirgaceae
Flammeovirga arenaria AB078078 Flammeovirgaceae
Flammeovirga aprica AB247553 Flammeovirgaceae
Flammeovirga yaeyamensis AB247554 Flammeovirgaceae
Flammeovirga pacifica HQ412594 Flammeovirgaceae
Perexilibacter aurantiacus AB276355 Flammeovirgaceae
Sediminitomix flava TYGS 6150 Flammeovirgaceae
Limibacter armeniacum AB359907 Flammeovirgaceae
Garritya polymorpha AB078059 Bernardetiaceae
Hugenholtzia roseola TYGS 1401 Bernardetiaceae
Bernardetia litoralis TYGS 207 Bernardetiaceae
Thermoflexibacter ruber TYGS 2465 Cytophagales
Thermonema lapsum HE582775 Thermonemataceae
Thermonema rossianum TYGS 1438 Thermonemataceae
Mooreia alkaloidigena TYGS 4902 Mooreiaceae
Marivirga lumbricoides HQ638976 Flammeovirgaceae
Marivirga atlantica KM117235 Flammeovirgaceae
Marivirga harenae AB078071 Flammeovirgaceae
Marivirga tractuosa TYGS 134 Flammeovirgaceae
Marivirga sericea TYGS 2213 Flammeovirgaceae
Echinicola jeungdonensis GU339180 Cyclobacteriaceae
Echinicola vietnamensis TYGS 203 Cyclobacteriaceae
Echinicola rosea KT989434 Cyclobacteriaceae
Echinicola strongylocentroti KU647633 Cyclobacteriaceae
Echinicola pacifica TYGS 1142 Cyclobacteriaceae
Echinicola sediminis KX774280 Cyclobacteriaceae
Lunatimonas salinarum LN997816 Cyclobacteriaceae
Lunatimonas lonarensis TYGS 3624 Cyclobacteriaceae
Cyclobacterium lianum DQ534063 Cyclobacteriaceae
Cyclobacterium jeungdonense GU291862 Cyclobacteriaceae
Cyclobacterium xiamenense KC206024 Cyclobacteriaceae
Cyclobacterium halophilum TYGS 8744 Cyclobacteriaceae
Cyclobacterium marinum TYGS 202 Cyclobacteriaceae
Cyclobacterium caenipelagi JX515600 Cyclobacteriaceae
Cyclobacterium amurskyense TYGS 3259 Cyclobacteriaceae
Cyclobacterium qasimii FN377719 Cyclobacteriaceae
Negadavirga shengliensis KF309029 Cyclobacteriaceae
Rhodonellum psychrophilum TYGS 1293 Cyclobacteriaceae
Aquiflexum balticum TYGS 1064 Cyclobacteriaceae
Mariniradius saccharolyticus TYGS 3597 Cyclobacteriaceae
Mongoliibacter ruber KT253329 Cyclobacteriaceae
Indibacter alkaliphilus TYGS 3260 Cyclobacteriaceae
Mongoliitalea lutea HM140980 Cyclobacteriaceae
Fontibacter ferrireducens JQ348962 Cyclobacteriaceae
Fontibacter flavus FN421478 Cyclobacteriaceae
Cecembia lonarensis FN393747 Cyclobacteriaceae
Cecembia calidifontis HE806326 Cyclobacteriaceae
Cecembia rubra KJ361469 Cyclobacteriaceae
Shivajiella indica FR681897 Cyclobacteriaceae
Belliella marina KR048277 Cyclobacteriaceae
Belliella aquatica KC762321 Cyclobacteriaceae
Belliella baltica TYGS 201 Cyclobacteriaceae
Belliella kenyensis KF976732 Cyclobacteriaceae
Belliella pelovolcani TYGS 14250 Cyclobacteriaceae
Belliella buryatensis TYGS 23122 Cyclobacteriaceae
Algoriphagus namhaensis HQ401024 Cyclobacteriaceae
Algoriphagus halophilus TYGS 6206 Cyclobacteriaceae
Algoriphagus lutimaris FJ669216 Cyclobacteriaceae
Algoriphagus chungangensis JN967625 Cyclobacteriaceae
Algoriphagus machipongonensis TYGS 3857 Cyclobacteriaceae
Algoriphagus iocasae KX002012 Cyclobacteriaceae
Algoriphagus aestuarii KR012506 Cyclobacteriaceae
Algoriphagus resistens TYGS 23211 Cyclobacteriaceae

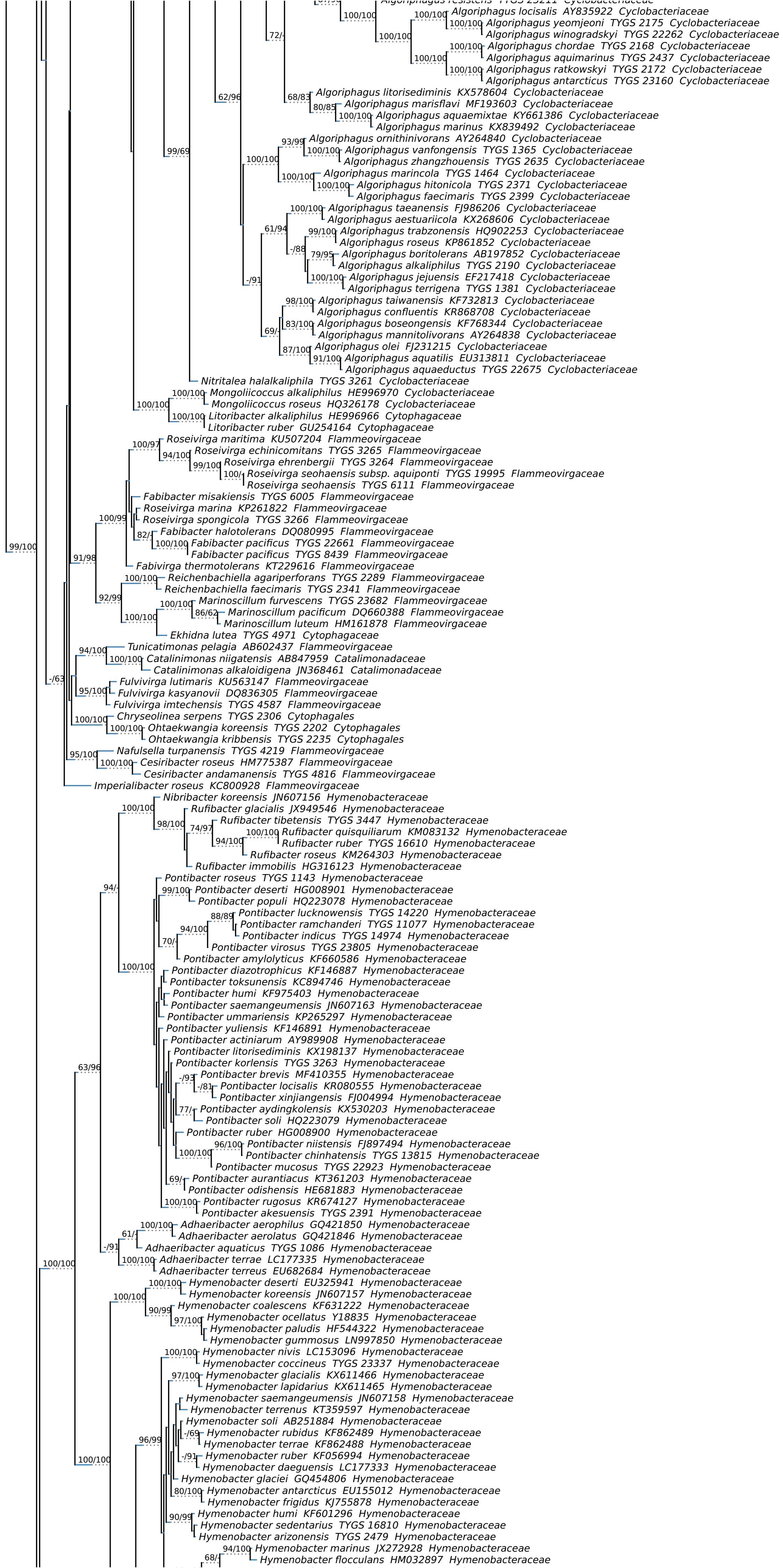
Algoriphagus locisalis AY835922 Cyclobacteriaceae
Algoriphagus yeomjeoni TYGS 2175 Cyclobacteriaceae
Algoriphagus winogradskyi TYGS 22262 Cyclobacteriaceae
Algoriphagus chordae TYGS 2168 Cyclobacteriaceae
Algoriphagus aquimarinus TYGS 2437 Cyclobacteriaceae
Algoriphagus ratkowskyi TYGS 2172 Cyclobacteriaceae
Algoriphagus antarcticus TYGS 23160 Cyclobacteriaceae
Algoriphagus litorisediminis KX578604 Cyclobacteriaceae
Algoriphagus marisflavi MF193603 Cyclobacteriaceae
Algoriphagus aquaemixtae KY661386 Cyclobacteriaceae
Algoriphagus marinus KX839492 Cyclobacteriaceae
Algoriphagus ornithinivorans AY264840 Cyclobacteriaceae
Algoriphagus vanfongensis TYGS 1365 Cyclobacteriaceae
Algoriphagus zhangzhouensis TYGS 2635 Cyclobacteriaceae
Algoriphagus marincola TYGS 1464 Cyclobacteriaceae
Algoriphagus hitonicola TYGS 2371 Cyclobacteriaceae
Algoriphagus faecimaris TYGS 2399 Cyclobacteriaceae
Algoriphagus taeanensis FJ986206 Cyclobacteriaceae
Algoriphagus aestuariicola KX268606 Cyclobacteriaceae
Algoriphagus trabzonensis HQ902253 Cyclobacteriaceae
Algoriphagus roseus KP861852 Cyclobacteriaceae
Algoriphagus boritolerans AB197852 Cyclobacteriaceae
Algoriphagus alkaliphilus TYGS 2190 Cyclobacteriaceae
Algoriphagus jejuensis EF217418 Cyclobacteriaceae
Algoriphagus terrigena TYGS 1381 Cyclobacteriaceae
Algoriphagus taiwanensis KF732813 Cyclobacteriaceae
Algoriphagus confluentis KR868708 Cyclobacteriaceae
Algoriphagus boseongensis KF768344 Cyclobacteriaceae
Algoriphagus mannitolivorans AY264838 Cyclobacteriaceae
Algoriphagus olei FJ231215 Cyclobacteriaceae
Algoriphagus aquatilis EU313811 Cyclobacteriaceae
Algoriphagus aquaeductus TYGS 22675 Cyclobacteriaceae
Nitritalea halalkaliphila TYGS 3261 Cyclobacteriaceae
Mongoliicoccus alkaliphilus HE996970 Cyclobacteriaceae
Mongoliicoccus roseus HQ326178 Cyclobacteriaceae
Litoribacter alkaliphilus HE996966 Cytophagaceae
Litoribacter ruber GU254164 Cytophagaceae
Roseivirga maritima KU507204 Flammeovirgaceae
Roseivirga echinicomitans TYGS 3265 Flammeovirgaceae
Roseivirga ehrenbergii TYGS 3264 Flammeovirgaceae
Roseivirga seohaensis subsp. aquiponti TYGS 19995 Flammeovirgaceae
Roseivirga seohaensis TYGS 6111 Flammeovirgaceae
Fabibacter misakiensis TYGS 6005 Flammeovirgaceae
Roseivirga marina KP261822 Flammeovirgaceae
Roseivirga spongicola TYGS 3266 Flammeovirgaceae
Fabibacter halotolerans DQ080995 Flammeovirgaceae
Fabibacter pacificus TYGS 22661 Flammeovirgaceae
Fabibacter pacificus TYGS 8439 Flammeovirgaceae
Fabivirga thermotolerans KT229616 Flammeovirgaceae
Reichenbachiella agariperforans TYGS 2289 Flammeovirgaceae
Reichenbachiella faecimaris TYGS 2341 Flammeovirgaceae
Marinoscillum furvescens TYGS 23682 Flammeovirgaceae
Marinoscillum pacificum DQ660388 Flammeovirgaceae
Marinoscillum luteum HM161878 Flammeovirgaceae
Ekhidna lutea TYGS 4971 Cytophagaceae
Tunicatimonas pelagia AB602437 Flammeovirgaceae
Catalinimonas niigatensis AB847959 Catalimonadaceae
Catalinimonas alkaloidigena JN368461 Catalimonadaceae
Fulvivirga lutimaris KU563147 Flammeovirgaceae
Fulvivirga kasyanovii DQ836305 Flammeovirgaceae
Fulvivirga imtechensis TYGS 4587 Flammeovirgaceae
Chryseolinea serpens TYGS 2306 Cytophagales
Ohtaekwangia koreensis TYGS 2202 Cytophagales
Ohtaekwangia kribbensis TYGS 2235 Cytophagales
Nafulsella turpanensis TYGS 4219 Flammeovirgaceae
Cesiribacter roseus HM775387 Flammeovirgaceae
Cesiribacter andamanensis TYGS 4816 Flammeovirgaceae
Imperialibacter roseus KC800928 Flammeovirgaceae
Nibribacter koreensis JN607156 Hymenobacteraceae
Rufibacter glacialis JX949546 Hymenobacteraceae
Rufibacter tibetensis TYGS 3447 Hymenobacteraceae
Rufibacter quisquiliarum KM083132 Hymenobacteraceae
Rufibacter ruber TYGS 16610 Hymenobacteraceae
Rufibacter roseus KM264303 Hymenobacteraceae
Rufibacter immobilis HG316123 Hymenobacteraceae
Pontibacter roseus TYGS 1143 Hymenobacteraceae
Pontibacter deserti HG008901 Hymenobacteraceae
Pontibacter populi HQ223078 Hymenobacteraceae
Pontibacter lucknowensis TYGS 14220 Hymenobacteraceae
Pontibacter ramchanderi TYGS 11077 Hymenobacteraceae
Pontibacter indicus TYGS 14974 Hymenobacteraceae
Pontibacter virosus TYGS 23805 Hymenobacteraceae
Pontibacter amylolyticus KF660586 Hymenobacteraceae
Pontibacter diazotrophicus KF146887 Hymenobacteraceae
Pontibacter toksunensis KC894746 Hymenobacteraceae
Pontibacter humi KF975403 Hymenobacteraceae
Pontibacter saemangeumensis JN607163 Hymenobacteraceae
Pontibacter ummariensis KP265297 Hymenobacteraceae
Pontibacter yuliensis KF146891 Hymenobacteraceae
Pontibacter actiniarum AY989908 Hymenobacteraceae
Pontibacter litorisediminis KX198137 Hymenobacteraceae
Pontibacter korlensis TYGS 3263 Hymenobacteraceae
Pontibacter brevis MF410355 Hymenobacteraceae
Pontibacter locisalis KR080555 Hymenobacteraceae
Pontibacter xinjiangensis FJ004994 Hymenobacteraceae
Pontibacter aydingkolensis KX530203 Hymenobacteraceae
Pontibacter soli HQ223079 Hymenobacteraceae
Pontibacter ruber HG008900 Hymenobacteraceae
Pontibacter niistensis FJ897494 Hymenobacteraceae
Pontibacter chinhatensis TYGS 13815 Hymenobacteraceae
Pontibacter mucosus TYGS 22923 Hymenobacteraceae
Pontibacter aurantiacus KT361203 Hymenobacteraceae
Pontibacter odishensis HE681883 Hymenobacteraceae
Pontibacter rugosus KR674127 Hymenobacteraceae
Pontibacter akesuensis TYGS 2391 Hymenobacteraceae
Adhaeribacter aerophilus GQ421850 Hymenobacteraceae
Adhaeribacter aerolatus GQ421846 Hymenobacteraceae
Adhaeribacter aquaticus TYGS 1086 Hymenobacteraceae
Adhaeribacter terrae LC177335 Hymenobacteraceae
Adhaeribacter terreus EU682684 Hymenobacteraceae
Hymenobacter deserti EU325941 Hymenobacteraceae
Hymenobacter koreensis JN607157 Hymenobacteraceae
Hymenobacter coalescens KF631222 Hymenobacteraceae
Hymenobacter ocellatus Y18835 Hymenobacteraceae
Hymenobacter paludis HF544322 Hymenobacteraceae
Hymenobacter gummosus LN997850 Hymenobacteraceae
Hymenobacter nivis LC153096 Hymenobacteraceae
Hymenobacter coccineus TYGS 23337 Hymenobacteraceae
Hymenobacter glacialis KX611466 Hymenobacteraceae
Hymenobacter lapidarius KX611465 Hymenobacteraceae
Hymenobacter saemangeumensis JN607158 Hymenobacteraceae
Hymenobacter terrenus KT359597 Hymenobacteraceae
Hymenobacter soli AB251884 Hymenobacteraceae
Hymenobacter rubidus KF862489 Hymenobacteraceae
Hymenobacter terrae KF862488 Hymenobacteraceae
Hymenobacter ruber KF056994 Hymenobacteraceae
Hymenobacter daeguensis LC177333 Hymenobacteraceae
Hymenobacter glaciei GQ454806 Hymenobacteraceae
Hymenobacter antarcticus EU155012 Hymenobacteraceae
Hymenobacter frigidus KJ755878 Hymenobacteraceae
Hymenobacter humi KF601296 Hymenobacteraceae
Hymenobacter sedentarius TYGS 16810 Hymenobacteraceae
Hymenobacter arizonensis TYGS 2479 Hymenobacteraceae
Hymenobacter marinus JX272928 Hymenobacteraceae
Hymenobacter flocculans HM032897 Hymenobacteraceae

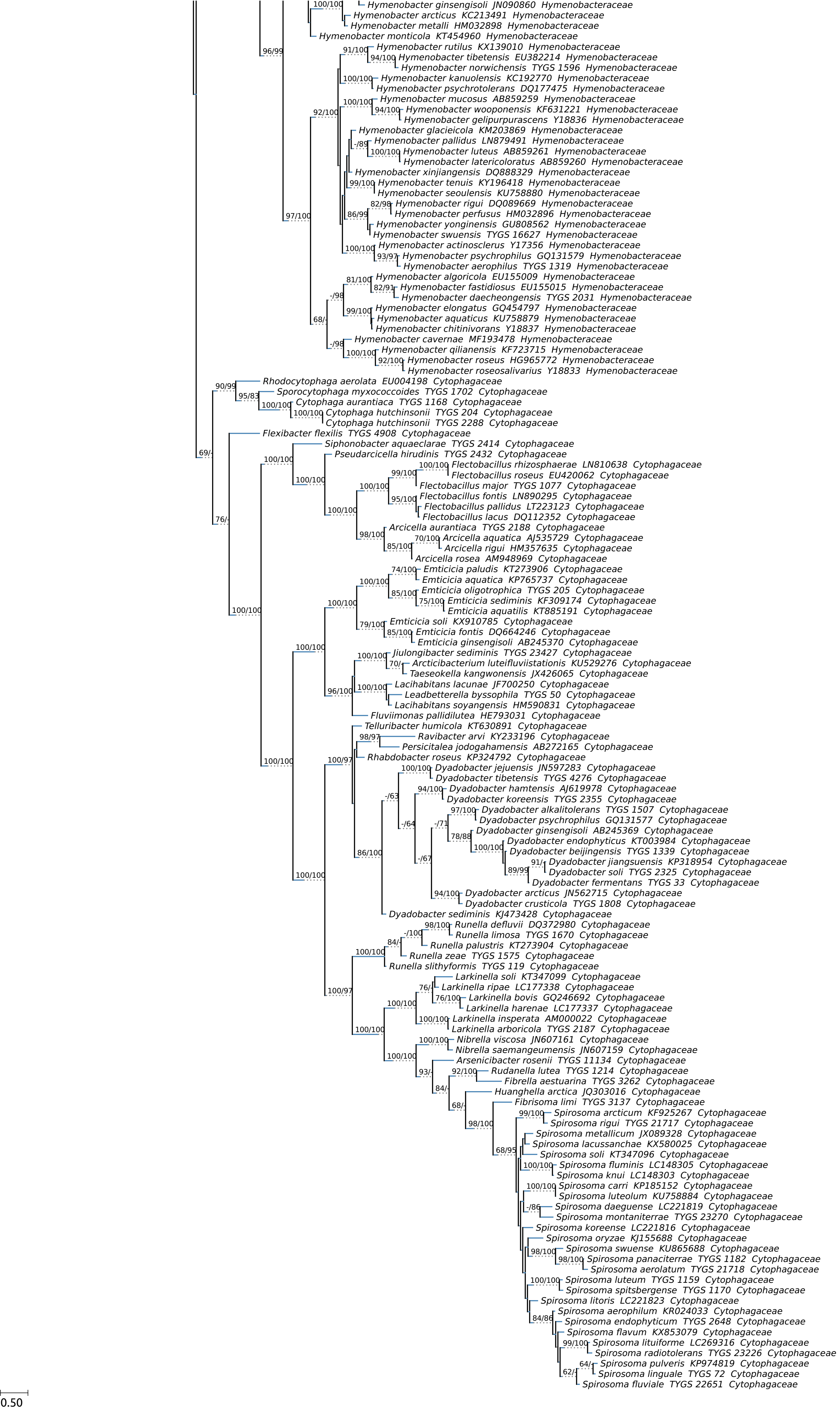
Hymenobacter ginsengisoli JN090860 Hymenobacteraceae
Hymenobacter arcticus KC213491 Hymenobacteraceae
Hymenobacter metalli HM032898 Hymenobacteraceae
Hymenobacter monticola KT454960 Hymenobacteraceae
Hymenobacter rutilus KX139010 Hymenobacteraceae
Hymenobacter tibetensis EU382214 Hymenobacteraceae
Hymenobacter norwichensis TYGS 1596 Hymenobacteraceae
Hymenobacter kanuolensis KC192770 Hymenobacteraceae
Hymenobacter psychrotolerans DQ177475 Hymenobacteraceae
Hymenobacter mucosus AB859259 Hymenobacteraceae
Hymenobacter wooponensis KF631221 Hymenobacteraceae
Hymenobacter gelipurpurascens Y18836 Hymenobacteraceae
Hymenobacter glacieicola KM203869 Hymenobacteraceae
Hymenobacter pallidus LN879491 Hymenobacteraceae
Hymenobacter luteus AB859261 Hymenobacteraceae
Hymenobacter latericoloratus AB859260 Hymenobacteraceae
Hymenobacter xinjiangensis DQ888329 Hymenobacteraceae
Hymenobacter tenuis KY196418 Hymenobacteraceae
Hymenobacter seoulensis KU758880 Hymenobacteraceae
Hymenobacter rigui DQ089669 Hymenobacteraceae
Hymenobacter perfusus HM032896 Hymenobacteraceae
Hymenobacter yonginensis GU808562 Hymenobacteraceae
Hymenobacter swuensis TYGS 16627 Hymenobacteraceae
Hymenobacter actinosclerus Y17356 Hymenobacteraceae
Hymenobacter psychrophilus GQ131579 Hymenobacteraceae
Hymenobacter aerophilus TYGS 1319 Hymenobacteraceae
Hymenobacter algoricola EU155009 Hymenobacteraceae
Hymenobacter fastidiosus EU155015 Hymenobacteraceae
Hymenobacter daecheongensis TYGS 2031 Hymenobacteraceae
Hymenobacter elongatus GQ454797 Hymenobacteraceae
Hymenobacter aquaticus KU758879 Hymenobacteraceae
Hymenobacter chitinivorans Y18837 Hymenobacteraceae
Hymenobacter cavernae MF193478 Hymenobacteraceae
Hymenobacter qilianensis KF723715 Hymenobacteraceae
Hymenobacter roseus HG965772 Hymenobacteraceae
Hymenobacter roseosalivarius Y18833 Hymenobacteraceae
Rhodocytophaga aerolata EU004198 Cytophagaceae
Sporocytophaga myxococcoides TYGS 1702 Cytophagaceae
Cytophaga aurantiaca TYGS 1168 Cytophagaceae
Cytophaga hutchinsonii TYGS 204 Cytophagaceae
Cytophaga hutchinsonii TYGS 2288 Cytophagaceae
Flexibacter flexilis TYGS 4908 Cytophagaceae
Siphonobacter aquaeclarae TYGS 2414 Cytophagaceae
Pseudarcicella hirudinis TYGS 2432 Cytophagaceae
Flectobacillus rhizosphaerae LN810638 Cytophagaceae
Flectobacillus roseus EU420062 Cytophagaceae
Flectobacillus major TYGS 1077 Cytophagaceae
Flectobacillus fontis LN890295 Cytophagaceae
Flectobacillus pallidus LT223123 Cytophagaceae
Flectobacillus lacus DQ112352 Cytophagaceae
Arcicella aurantiaca TYGS 2188 Cytophagaceae
Arcicella aquatica AJ535729 Cytophagaceae
Arcicella rigui HM357635 Cytophagaceae
Arcicella rosea AM948969 Cytophagaceae
Emticicia paludis KT273906 Cytophagaceae
Emticicia aquatica KP765737 Cytophagaceae
Emticicia oligotrophica TYGS 205 Cytophagaceae
Emticicia sediminis KF309174 Cytophagaceae
Emticicia aquatilis KT885191 Cytophagaceae
Emticicia soli KX910785 Cytophagaceae
Emticicia fontis DQ664246 Cytophagaceae
Emticicia ginsengisoli AB245370 Cytophagaceae
Jiulongibacter sediminis TYGS 23427 Cytophagaceae
Arcticibacterium luteifluviistationis KU529276 Cytophagaceae
Taeseokella kangwonensis JX426065 Cytophagaceae
Lacihabitans lacunae JF700250 Cytophagaceae
Leadbetterella byssophila TYGS 50 Cytophagaceae
Lacihabitans soyangensis HM590831 Cytophagaceae
Fluviimonas pallidilutea HE793031 Cytophagaceae
Telluribacter humicola KT630891 Cytophagaceae
Ravibacter arvi KY233196 Cytophagaceae
Persicitalea jodogahamensis AB272165 Cytophagaceae
Rhabdobacter roseus KP324792 Cytophagaceae
Dyadobacter jejuensis JN597283 Cytophagaceae
Dyadobacter tibetensis TYGS 4276 Cytophagaceae
Dyadobacter hamtensis AJ619978 Cytophagaceae
Dyadobacter koreensis TYGS 2355 Cytophagaceae
Dyadobacter alkalitolerans TYGS 1507 Cytophagaceae
Dyadobacter psychrophilus GQ131577 Cytophagaceae
Dyadobacter ginsengisoli AB245369 Cytophagaceae
Dyadobacter endophyticus KT003984 Cytophagaceae
Dyadobacter beijingensis TYGS 1339 Cytophagaceae
Dyadobacter jiangsuensis KP318954 Cytophagaceae
Dyadobacter soli TYGS 2325 Cytophagaceae
Dyadobacter fermentans TYGS 33 Cytophagaceae
Dyadobacter arcticus JN562715 Cytophagaceae
Dyadobacter crusticola TYGS 1808 Cytophagaceae
Dyadobacter sediminis KJ473428 Cytophagaceae
Runella defluvii DQ372980 Cytophagaceae
Runella limosa TYGS 1670 Cytophagaceae
Runella palustris KT273904 Cytophagaceae
Runella zeae TYGS 1575 Cytophagaceae
Runella slithyformis TYGS 119 Cytophagaceae
Larkinella soli KT347099 Cytophagaceae
Larkinella ripae LC177338 Cytophagaceae
Larkinella bovis GQ246692 Cytophagaceae
Larkinella harenae LC177337 Cytophagaceae
Larkinella insperata AM000022 Cytophagaceae
Larkinella arboricola TYGS 2187 Cytophagaceae
Nibrella viscosa JN607161 Cytophagaceae
Nibrella saemangeumensis JN607159 Cytophagaceae
Arsenicibacter rosenii TYGS 11134 Cytophagaceae
Rudanella lutea TYGS 1214 Cytophagaceae
Fibrella aestuarina TYGS 3262 Cytophagaceae
Huanghella arctica JQ303016 Cytophagaceae
Fibrisoma limi TYGS 3137 Cytophagaceae
Spirosoma arcticum KF925267 Cytophagaceae
Spirosoma rigui TYGS 21717 Cytophagaceae
Spirosoma metallicum JX089328 Cytophagaceae
Spirosoma lacussanchae KX580025 Cytophagaceae
Spirosoma soli KT347096 Cytophagaceae
Spirosoma fluminis LC148305 Cytophagaceae
Spirosoma knui LC148303 Cytophagaceae
Spirosoma carri KP185152 Cytophagaceae
Spirosoma luteolum KU758884 Cytophagaceae
Spirosoma daeguense LC221819 Cytophagaceae
Spirosoma montaniterrae TYGS 23270 Cytophagaceae
Spirosoma koreense LC221816 Cytophagaceae
Spirosoma oryzae KJ155688 Cytophagaceae
Spirosoma swuense KU865688 Cytophagaceae
Spirosoma panaciterrae TYGS 1182 Cytophagaceae
Spirosoma aerolatum TYGS 21718 Cytophagaceae
Spirosoma luteum TYGS 1159 Cytophagaceae
Spirosoma spitsbergense TYGS 1170 Cytophagaceae
Spirosoma litoris LC221823 Cytophagaceae
Spirosoma aerophilum KR024033 Cytophagaceae
Spirosoma endophyticum TYGS 2648 Cytophagaceae
Spirosoma flavum KX853079 Cytophagaceae
Spirosoma lituiforme LC269316 Cytophagaceae
Spirosoma radiotolerans TYGS 23226 Cytophagaceae
Spirosoma pulveris KP974819 Cytophagaceae
Spirosoma linguale TYGS 72 Cytophagaceae
Spirosoma fluviale TYGS 22651 Cytophagaceae
0.50

Figure 3: Backbone-constrained comprehensive 16S rRNA gene ML tree (CCT) of *Bacteroidetes* inferred under the GTR+CAT model. The branches are scaled in terms of the expected number of substitutions per site. The numbers above the branches are support values when larger than 60% from ML (left) and MP (right) bootstrapping. Dotted parts of branches are filled in to allow proper placement of bootstrap values and are not part of the actual branch length. Numbers preceeded by the term 'TYGS' in labels refer to the genome IDs as found in Supplementary Table S1 (first sheet). Each tip label ends with the family of the respective taxon.

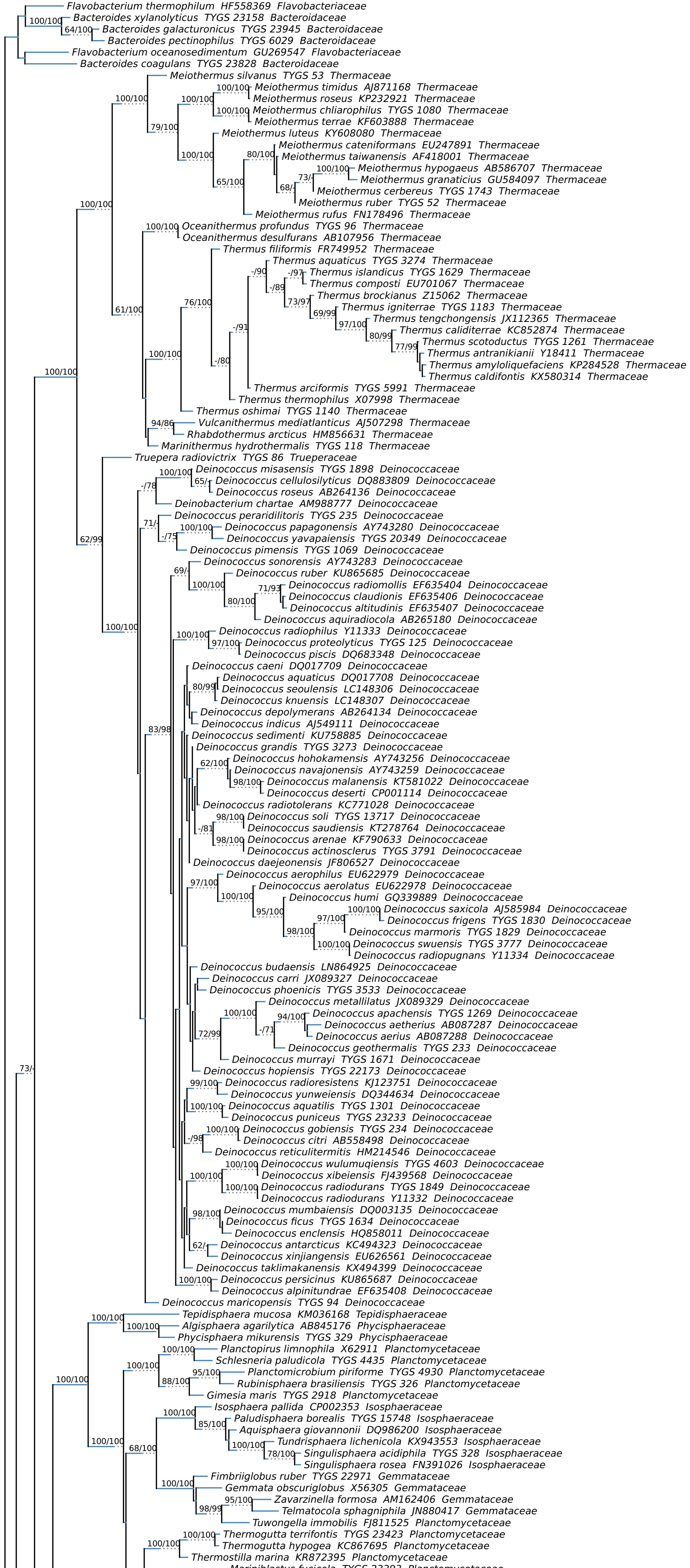
Flavobacterium thermophilum HF558369 Flavobacteriaceae
Bacteroides xylanolyticus TYGS 23158 Bacteroidaceae
Bacteroides galacturonicus TYGS 23945 Bacteroidaceae
Bacteroides pectinophilus TYGS 6029 Bacteroidaceae
Flavobacterium oceanosedimentum GU269547 Flavobacteriaceae
Bacteroides coagulans TYGS 23828 Bacteroidaceae
Meiothermus silvanus TYGS 53 Thermaceae
Meiothermus timidus AJ871168 Thermaceae
Meiothermus roseus KP232921 Thermaceae
Meiothermus chliarophilus TYGS 1080 Thermaceae
Meiothermus terrae KF603888 Thermaceae
Meiothermus luteus KY608080 Thermaceae
Meiothermus cateniformans EU247891 Thermaceae
Meiothermus taiwanensis AF418001 Thermaceae
Meiothermus hypogaeus AB586707 Thermaceae
Meiothermus granaticius GU584097 Thermaceae
Meiothermus cerbereus TYGS 1743 Thermaceae
Meiothermus ruber TYGS 52 Thermaceae
Meiothermus rufus FN178496 Thermaceae
Oceanithermus profundus TYGS 96 Thermaceae
Oceanithermus desulfurans AB107956 Thermaceae
Thermus filiformis FR749952 Thermaceae
Thermus aquaticus TYGS 3274 Thermaceae
Thermus islandicus TYGS 1629 Thermaceae
Thermus composti EU701067 Thermaceae
Thermus brockianus Z15062 Thermaceae
Thermus igniterrae TYGS 1183 Thermaceae
Thermus tengchongensis JX112365 Thermaceae
Thermus caliditerrae KC852874 Thermaceae
Thermus scotoductus TYGS 1261 Thermaceae
Thermus antranikianii Y18411 Thermaceae
Thermus amyloliquefaciens KP284528 Thermaceae
Thermus caldifontis KX580314 Thermaceae
Thermus arciformis TYGS 5991 Thermaceae
Thermus thermophilus X07998 Thermaceae
Thermus oshimai TYGS 1140 Thermaceae
Vulcanithermus mediatlanticus AJ507298 Thermaceae
Rhabdothermus arcticus HM856631 Thermaceae
Marinithermus hydrothermalis TYGS 118 Thermaceae
Truepera radiovictrix TYGS 86 Trueperaceae
Deinococcus misasensis TYGS 1898 Deinococcaceae
Deinococcus cellulosilyticus DQ883809 Deinococcaceae
Deinococcus roseus AB264136 Deinococcaceae
Deinobacterium chartae AM988777 Deinococcaceae
Deinococcus perariditoris TYGS 235 Deinococcaceae
Deinococcus papagonensis AY743280 Deinococcaceae
Deinococcus yavapaiensis TYGS 20349 Deinococcaceae
Deinococcus pimensis TYGS 1069 Deinococcaceae
Deinococcus sonorensis AY743283 Deinococcaceae
Deinococcus ruber KU865685 Deinococcaceae
Deinococcus radiomollis EF635404 Deinococcaceae
Deinococcus claudionis EF635406 Deinococcaceae
Deinococcus altitudinis EF635407 Deinococcaceae
Deinococcus aquiradiocola AB265180 Deinococcaceae
Deinococcus radiophilus Y11333 Deinococcaceae
Deinococcus proteolyticus TYGS 125 Deinococcaceae
Deinococcus piscis DQ683348 Deinococcaceae
Deinococcus caeni DQ017709 Deinococcaceae
Deinococcus aquaticus DQ017708 Deinococcaceae
Deinococcus seoulensis LC148306 Deinococcaceae
Deinococcus knuensis LC148307 Deinococcaceae
Deinococcus depolymerans AB264134 Deinococcaceae
Deinococcus indicus AJ549111 Deinococcaceae
Deinococcus sedimenti KU758885 Deinococcaceae
Deinococcus grandis TYGS 3273 Deinococcaceae
Deinococcus hohokamensis AY743256 Deinococcaceae
Deinococcus navajonensis AY743259 Deinococcaceae
Deinococcus malanensis KT581022 Deinococcaceae
Deinococcus deserti CP001114 Deinococcaceae
Deinococcus radiotolerans KC771028 Deinococcaceae
Deinococcus soli TYGS 13717 Deinococcaceae
Deinococcus saudiensis KT278764 Deinococcaceae
Deinococcus arenae KF790633 Deinococcaceae
Deinococcus actinosclerus TYGS 3791 Deinococcaceae
Deinococcus daejeonensis JF806527 Deinococcaceae
Deinococcus aerophilus EU622979 Deinococcaceae
Deinococcus aerolatus EU622978 Deinococcaceae
Deinococcus humi GQ339889 Deinococcaceae
Deinococcus saxicola AJ585984 Deinococcaceae
Deinococcus frigens TYGS 1830 Deinococcaceae
Deinococcus marmoris TYGS 1829 Deinococcaceae
Deinococcus swuensis TYGS 3777 Deinococcaceae
Deinococcus radiopugnans Y11334 Deinococcaceae
Deinococcus budaensis LN864925 Deinococcaceae
Deinococcus carri JX089327 Deinococcaceae
Deinococcus phoenicis TYGS 3533 Deinococcaceae
Deinococcus metallilatus JX089329 Deinococcaceae
Deinococcus apachensis TYGS 1269 Deinococcaceae
Deinococcus aetherius AB087287 Deinococcaceae
Deinococcus aerius AB087288 Deinococcaceae
Deinococcus geothermalis TYGS 233 Deinococcaceae
Deinococcus murrayi TYGS 1671 Deinococcaceae
Deinococcus hopiensis TYGS 22173 Deinococcaceae
Deinococcus radioresistens KJ123751 Deinococcaceae
Deinococcus yunweiensis DQ344634 Deinococcaceae
Deinococcus aquatilis TYGS 1301 Deinococcaceae
Deinococcus puniceus TYGS 23233 Deinococcaceae
Deinococcus gobiensis TYGS 234 Deinococcaceae
Deinococcus citri AB558498 Deinococcaceae
Deinococcus reticulitermitis HM214546 Deinococcaceae
Deinococcus wulumuqiensis TYGS 4603 Deinococcaceae
Deinococcus xibeiensis FJ439568 Deinococcaceae
Deinococcus radiodurans TYGS 1849 Deinococcaceae
Deinococcus radiodurans Y11332 Deinococcaceae
Deinococcus mumbaiensis DQ003135 Deinococcaceae
Deinococcus ficus TYGS 1634 Deinococcaceae
Deinococcus enclensis HQ858011 Deinococcaceae
Deinococcus antarcticus KC494323 Deinococcaceae
Deinococcus xinjiangensis EU626561 Deinococcaceae
Deinococcus taklimakanensis KX494399 Deinococcaceae
Deinococcus persicinus KU865687 Deinococcaceae
Deinococcus alpinitundrae EF635408 Deinococcaceae
Deinococcus maricopensis TYGS 94 Deinococcaceae
Tepidisphaera mucosa KM036168 Tepidisphaeraceae
Algisphaera agarilytica AB845176 Phycisphaeraceae
Phycisphaera mikurensis TYGS 329 Phycisphaeraceae
Planctopirus limnophila X62911 Planctomycetaceae
Schlesneria paludicola TYGS 4435 Planctomycetaceae
Planctomicrobium piriforme TYGS 4930 Planctomycetaceae
Rubinisphaera brasiliensis TYGS 326 Planctomycetaceae
Gimesia maris TYGS 2918 Planctomycetaceae
Isosphaera pallida CP002353 Isosphaeraceae
Paludisphaera borealis TYGS 15748 Isosphaeraceae
Aquisphaera giovannonii DQ986200 Isosphaeraceae
Tundrisphaera lichenicola KX943553 Isosphaeraceae
Singulisphaera acidiphila TYGS 328 Isosphaeraceae
Singulisphaera rosea FN391026 Isosphaeraceae
Fimbriiglobus ruber TYGS 22971 Gemmataceae
Gemmata obscuriglobus X56305 Gemmataceae
Zavarzinella formosa AM162406 Gemmataceae
Telmatocola sphagniphila JN880417 Gemmataceae
Tuwongella immobilis FJ811525 Planctomycetaceae
Thermogutta terrifontis TYGS 23423 Planctomycetaceae
Thermogutta hypogea KC867695 Planctomycetaceae
Thermostilla marina KR872395 Planctomycetaceae

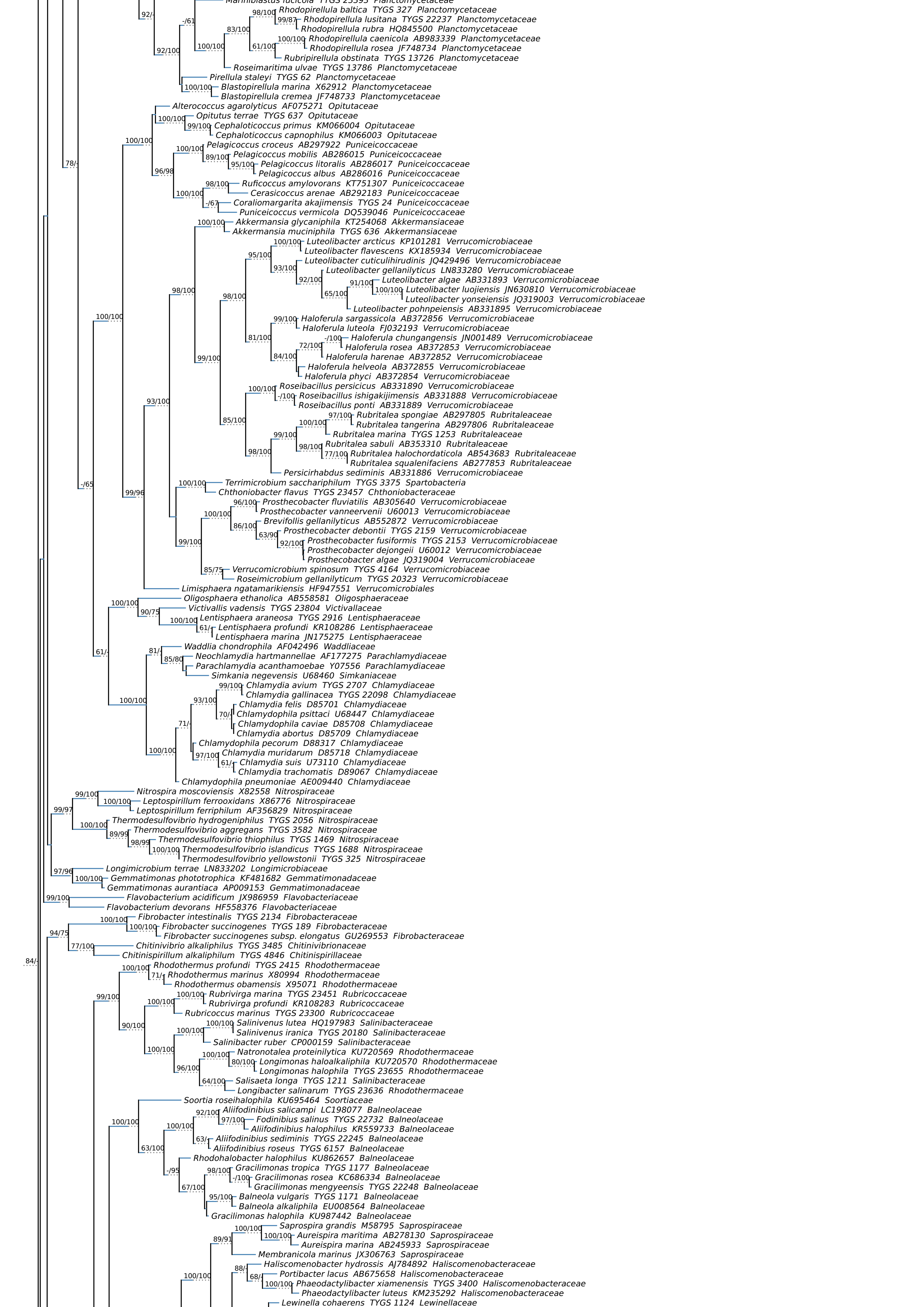
Rhodopirellula baltica TYGS 327 Planctomycetaceae
Rhodopirellula lusitana TYGS 22237 Planctomycetaceae
Rhodopirellula rubra HQ845500 Planctomycetaceae
Rhodopirellula caenicola AB983339 Planctomycetaceae
Rhodopirellula rosea JF748734 Planctomycetaceae
Rubripirellula obstinata TYGS 13726 Planctomycetaceae
Roseimaritima ulvae TYGS 13786 Planctomycetaceae
Pirellula staleyi TYGS 62 Planctomycetaceae
Blastopirellula marina X62912 Planctomycetaceae
Blastopirellula cremea JF748733 Planctomycetaceae
Alterococcus agarolyticus AF075271 Opitutaceae
Opitutus terrae TYGS 637 Opitutaceae
Cephaloticoccus primus KM066004 Opitutaceae
Cephaloticoccus capnophilus KM066003 Opitutaceae
Pelagicoccus croceus AB297922 Puniceicoccaceae
Pelagicoccus mobilis AB286015 Puniceicoccaceae
Pelagicoccus litoralis AB286017 Puniceicoccaceae
Pelagicoccus albus AB286016 Puniceicoccaceae
Ruficoccus amylovorans KT751307 Puniceicoccaceae
Cerasicoccus arenae AB292183 Puniceicoccaceae
Coraliomargarita akajimensis TYGS 24 Puniceicoccaceae
Puniceicoccus vermicola DQ539046 Puniceicoccaceae
Akkermansia glycaniphila KT254068 Akkermansiaceae
Akkermansia muciniphila TYGS 636 Akkermansiaceae
Luteolibacter arcticus KP101281 Verrucomicrobiaceae
Luteolibacter flavescens KX185934 Verrucomicrobiaceae
Luteolibacter cuticulihirudinis JQ429496 Verrucomicrobiaceae
Luteolibacter gellanilyticus LN833280 Verrucomicrobiaceae
Luteolibacter algae AB331893 Verrucomicrobiaceae
Luteolibacter luojiensis JN630810 Verrucomicrobiaceae
Luteolibacter yonseiensis JQ319003 Verrucomicrobiaceae
Luteolibacter pohnpeiensis AB331895 Verrucomicrobiaceae
Haloferula sargassicola AB372856 Verrucomicrobiaceae
Haloferula luteola FJ032193 Verrucomicrobiaceae
Haloferula chungangensis JN001489 Verrucomicrobiaceae
Haloferula rosea AB372853 Verrucomicrobiaceae
Haloferula harenae AB372852 Verrucomicrobiaceae
Haloferula helveola AB372855 Verrucomicrobiaceae
Haloferula phyci AB372854 Verrucomicrobiaceae
Roseibacillus persicicus AB331890 Verrucomicrobiaceae
Roseibacillus ishigakijimensis AB331888 Verrucomicrobiaceae
Roseibacillus ponti AB331889 Verrucomicrobiaceae
Rubritalea spongiae AB297805 Rubritaleaceae
Rubritalea tangerina AB297806 Rubritaleaceae
Rubritalea marina TYGS 1253 Rubritaleaceae
Rubritalea sabuli AB353310 Rubritaleaceae
Rubritalea halochordaticola AB543683 Rubritaleaceae
Rubritalea squalenifaciens AB277853 Rubritaleaceae
Persicirhabdus sediminis AB331886 Verrucomicrobiaceae
Terrimicrobium sacchariphilum TYGS 3375 Spartobacteria
Chthoniobacter flavus TYGS 23457 Chthoniobacteraceae
Prosthecobacter fluviatilis AB305640 Verrucomicrobiaceae
Prosthecobacter vanneervenii U60013 Verrucomicrobiaceae
Brevifollis gellanilyticus AB552872 Verrucomicrobiaceae
Prosthecobacter debontii TYGS 2159 Verrucomicrobiaceae
Prosthecobacter fusiformis TYGS 2153 Verrucomicrobiaceae
Prosthecobacter dejongeii U60012 Verrucomicrobiaceae
Prosthecobacter algae JQ319004 Verrucomicrobiaceae
Verrucomicrobium spinosum TYGS 4164 Verrucomicrobiaceae
Roseimicrobium gellanilyticum TYGS 20323 Verrucomicrobiaceae
Limisphaera ngatamarikiensis HF947551 Verrucomicrobiales
Oligosphaera ethanolica AB558581 Oligosphaeraceae
Victivallis vadensis TYGS 23804 Victivallaceae
Lentisphaera araneosa TYGS 2916 Lentisphaeraceae
Lentisphaera profundi KR108286 Lentisphaeraceae
Lentisphaera marina JN175275 Lentisphaeraceae
Waddlia chondrophila AF042496 Waddliaceae
Neochlamydia hartmannellae AF177275 Parachlamydiaceae
Parachlamydia acanthamoebae Y07556 Parachlamydiaceae
Simkania negevensis U68460 Simkaniaceae
Chlamydia avium TYGS 2707 Chlamydiaceae
Chlamydia gallinacea TYGS 22098 Chlamydiaceae
Chlamydia felis D85701 Chlamydiaceae
Chlamydophila psittaci U68447 Chlamydiaceae
Chlamydophila caviae D85708 Chlamydiaceae
Chlamydia abortus D85709 Chlamydiaceae
Chlamydophila pecorum D88317 Chlamydiaceae
Chlamydia muridarum D85718 Chlamydiaceae
Chlamydia suis U73110 Chlamydiaceae
Chlamydia trachomatis D89067 Chlamydiaceae
Chlamydophila pneumoniae AE009440 Chlamydiaceae
Nitrospira moscoviensis X82558 Nitrospiraceae
Leptospirillum ferrooxidans X86776 Nitrospiraceae
Leptospirillum ferriphilum AF356829 Nitrospiraceae
Thermodesulfovibrio hydrogeniphilus TYGS 2056 Nitrospiraceae
Thermodesulfovibrio aggregans TYGS 3582 Nitrospiraceae
Thermodesulfovibrio thiophilus TYGS 1469 Nitrospiraceae
Thermodesulfovibrio islandicus TYGS 1688 Nitrospiraceae
Thermodesulfovibrio yellowstonii TYGS 325 Nitrospiraceae
Longimicrobium terrae LN833202 Longimicrobiaceae
Gemmatimonas phototrophica KF481682 Gemmatimonadaceae
Gemmatimonas aurantiaca AP009153 Gemmatimonadaceae
Flavobacterium acidificum JX986959 Flavobacteriaceae
Flavobacterium devorans HF558376 Flavobacteriaceae
Fibrobacter intestinalis TYGS 2134 Fibrobacteraceae
Fibrobacter succinogenes TYGS 189 Fibrobacteraceae
Fibrobacter succinogenes subsp. elongatus GU269553 Fibrobacteraceae
Chitinivibrio alkaliphilus TYGS 3485 Chitinivibrionaceae
Chitinispirillum alkaliphilum TYGS 4846 Chitinispirillaceae
Rhodothermus profundi TYGS 2415 Rhodothermaceae
Rhodothermus marinus X80994 Rhodothermaceae
Rhodothermus obamensis X95071 Rhodothermaceae
Rubrivirga marina TYGS 23451 Rubricoccaceae
Rubrivirga profundi KR108283 Rubricoccaceae
Rubricoccus marinus TYGS 23300 Rubricoccaceae
Salinivenus lutea HQ197983 Salinibacteraceae
Salinivenus iranica TYGS 20180 Salinibacteraceae
Salinibacter ruber CP000159 Salinibacteraceae
Natronotalea proteinilytica KU720569 Rhodothermaceae
Longimonas haloalkaliphila KU720570 Rhodothermaceae
Longimonas halophila TYGS 23655 Rhodothermaceae
Salisaeta longa TYGS 1211 Salinibacteraceae
Longibacter salinarum TYGS 23636 Rhodothermaceae
Soortia roseihalophila KU695464 Soortiaceae
Aliifodinibius salicampi LC198077 Balneolaceae
Fodinibius salinus TYGS 22732 Balneolaceae
Aliifodinibius halophilus KR559733 Balneolaceae
Aliifodinibius sediminis TYGS 22245 Balneolaceae
Aliifodinibius roseus TYGS 6157 Balneolaceae
Rhodohalobacter halophilus KU862657 Balneolaceae
Gracilimonas tropica TYGS 1177 Balneolaceae
Gracilimonas rosea KC686334 Balneolaceae
Gracilimonas mengyeensis TYGS 22248 Balneolaceae
Balneola vulgaris TYGS 1171 Balneolaceae
Balneola alkaliphila EU008564 Balneolaceae
Gracilimonas halophila KU987442 Balneolaceae
Saprospira grandis M58795 Saprospiraceae
Aureispira maritima AB278130 Saprospiraceae
Aureispira marina AB245933 Saprospiraceae
Membranicola marinus JX306763 Saprospiraceae
Haliscomenobacter hydrossis AJ784892 Haliscomenobacteraceae
Portibacter lacus AB675658 Haliscomenobacteraceae
Phaeodactylibacter xiamenensis TYGS 3400 Haliscomenobacteraceae
Phaeodactylibacter luteus KM235292 Haliscomenobacteraceae
Lewinella cohaerens TYGS 1124 Lewinellaceae

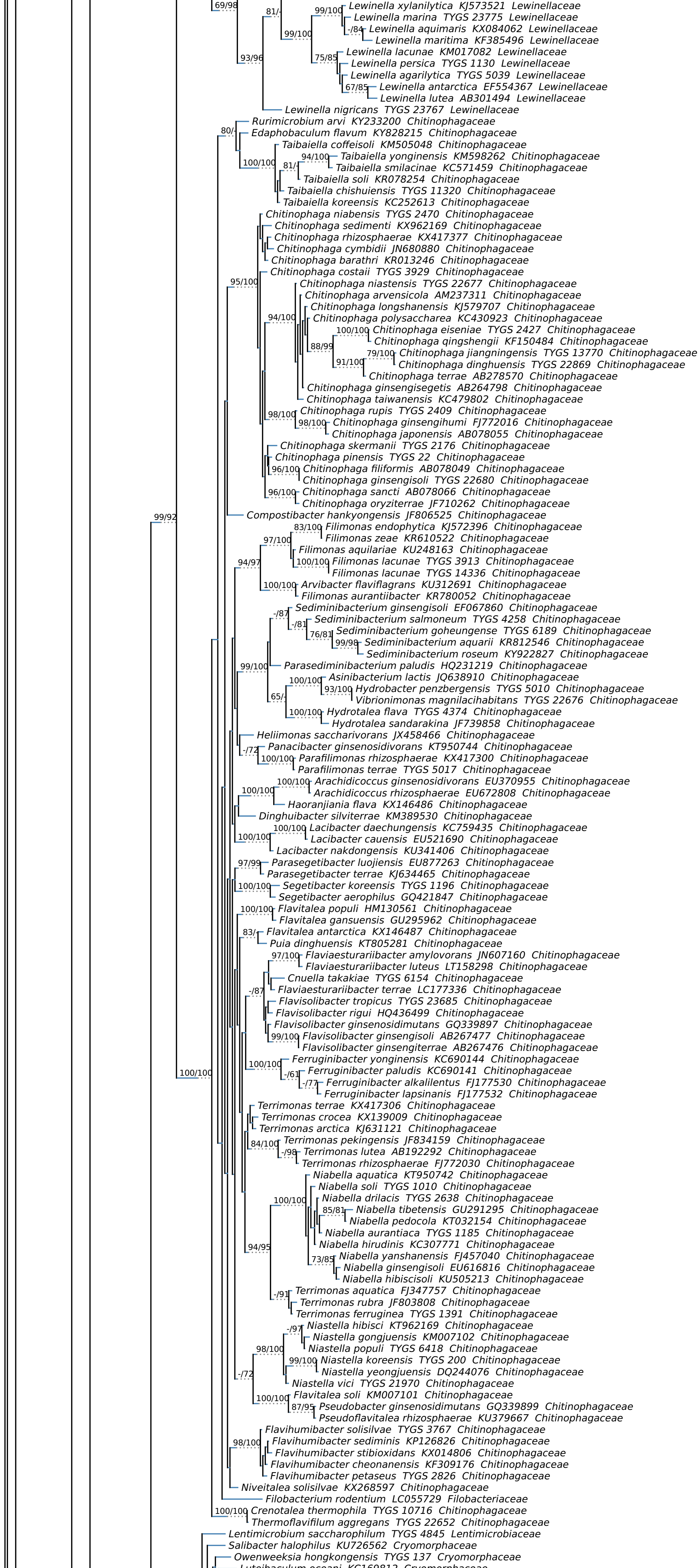
Lewinella xylanilytica KJ573521 Lewinellaceae
Lewinella marina TYGS 23775 Lewinellaceae
Lewinella aquimaris KX084062 Lewinellaceae
Lewinella maritima KF385496 Lewinellaceae
Lewinella lacunae KM017082 Lewinellaceae
Lewinella persica TYGS 1130 Lewinellaceae
Lewinella agarilytica TYGS 5039 Lewinellaceae
Lewinella antarctica EF554367 Lewinellaceae
Lewinella lutea AB301494 Lewinellaceae
Lewinella nigricans TYGS 23767 Lewinellaceae
Rurimicrobium arvi KY233200 Chitinophagaceae
Edaphobaculum flavum KY828215 Chitinophagaceae
Taibaiella coffeisoli KM505048 Chitinophagaceae
Taibaiella yonginensis KM598262 Chitinophagaceae
Taibaiella smilacinae KC571459 Chitinophagaceae
Taibaiella soli KR078254 Chitinophagaceae
Taibaiella chishuiensis TYGS 11320 Chitinophagaceae
Taibaiella koreensis KC252613 Chitinophagaceae
Chitinophaga niabensis TYGS 2470 Chitinophagaceae
Chitinophaga sedimenti KX962169 Chitinophagaceae
Chitinophaga rhizosphaerae KX417377 Chitinophagaceae
Chitinophaga cymbidii JN680880 Chitinophagaceae
Chitinophaga barathri KR013246 Chitinophagaceae
Chitinophaga costaii TYGS 3929 Chitinophagaceae
Chitinophaga niastensis TYGS 22677 Chitinophagaceae
Chitinophaga arvensicola AM237311 Chitinophagaceae
Chitinophaga longshanensis KJ579707 Chitinophagaceae
Chitinophaga polysaccharea KC430923 Chitinophagaceae
Chitinophaga eiseniae TYGS 2427 Chitinophagaceae
Chitinophaga qingshengii KF150484 Chitinophagaceae
Chitinophaga jiangningensis TYGS 13770 Chitinophagaceae
Chitinophaga dinghuensis TYGS 22869 Chitinophagaceae
Chitinophaga terrae AB278570 Chitinophagaceae
Chitinophaga ginsengisegetis AB264798 Chitinophagaceae
Chitinophaga taiwanensis KC479802 Chitinophagaceae
Chitinophaga rupis TYGS 2409 Chitinophagaceae
Chitinophaga ginsengihumi FJ772016 Chitinophagaceae
Chitinophaga japonensis AB078055 Chitinophagaceae
Chitinophaga skermanii TYGS 2176 Chitinophagaceae
Chitinophaga pinensis TYGS 22 Chitinophagaceae
Chitinophaga filiformis AB078049 Chitinophagaceae
Chitinophaga ginsengisoli TYGS 22680 Chitinophagaceae
Chitinophaga sancti AB078066 Chitinophagaceae
Chitinophaga oryziterrae JF710262 Chitinophagaceae
Compostibacter hankyongensis JF806525 Chitinophagaceae
Filimonas endophytica KJ572396 Chitinophagaceae
Filimonas zeae KR610522 Chitinophagaceae
Filimonas aquilariae KU248163 Chitinophagaceae
Filimonas lacunae TYGS 3913 Chitinophagaceae
Filimonas lacunae TYGS 14336 Chitinophagaceae
Arvibacter flaviflagrans KU312691 Chitinophagaceae
Filimonas aurantiibacter KR780052 Chitinophagaceae
Sediminibacterium ginsengisoli EF067860 Chitinophagaceae
Sediminibacterium salmoneum TYGS 4258 Chitinophagaceae
Sediminibacterium goheungense TYGS 6189 Chitinophagaceae
Sediminibacterium aquarii KR812546 Chitinophagaceae
Sediminibacterium roseum KY922827 Chitinophagaceae
Parasediminibacterium paludis HQ231219 Chitinophagaceae
Asinibacterium lactis JQ638910 Chitinophagaceae
Hydrobacter penzbergensis TYGS 5010 Chitinophagaceae
Vibrionimonas magnilacihabitans TYGS 22676 Chitinophagaceae
Hydrotalea flava TYGS 4374 Chitinophagaceae
Hydrotalea sandarakina JF739858 Chitinophagaceae
Heliimonas saccharivorans JX458466 Chitinophagaceae
Panacibacter ginsenosidivorans KT950744 Chitinophagaceae
Parafilimonas rhizosphaerae KX417300 Chitinophagaceae
Parafilimonas terrae TYGS 5017 Chitinophagaceae
Arachidicoccus ginsenosidivorans EU370955 Chitinophagaceae
Arachidicoccus rhizosphaerae EU672808 Chitinophagaceae
Haoranjiania flava KX146486 Chitinophagaceae
Dinghuibacter silviterrae KM389530 Chitinophagaceae
Lacibacter daechungensis KC759435 Chitinophagaceae
Lacibacter cauensis EU521690 Chitinophagaceae
Lacibacter nakdongensis KU341406 Chitinophagaceae
Parasegetibacter luojiensis EU877263 Chitinophagaceae
Parasegetibacter terrae KJ634465 Chitinophagaceae
Segetibacter koreensis TYGS 1196 Chitinophagaceae
Segetibacter aerophilus GQ421847 Chitinophagaceae
Flavitalea populi HM130561 Chitinophagaceae
Flavitalea gansuensis GU295962 Chitinophagaceae
Flavitalea antarctica KX146487 Chitinophagaceae
Puia dinghuensis KT805281 Chitinophagaceae
Flaviaesturariibacter amylovorans JN607160 Chitinophagaceae
Flaviaesturariibacter luteus LT158298 Chitinophagaceae
Cnuella takakiae TYGS 6154 Chitinophagaceae
Flaviaesturariibacter terrae LC177336 Chitinophagaceae
Flavisolibacter tropicus TYGS 23685 Chitinophagaceae
Flavisolibacter rigui HQ436499 Chitinophagaceae
Flavisolibacter ginsenosidimutans GQ339897 Chitinophagaceae
Flavisolibacter ginsengisoli AB267477 Chitinophagaceae
Flavisolibacter ginsengiterrae AB267476 Chitinophagaceae
Ferruginibacter yonginensis KC690144 Chitinophagaceae
Ferruginibacter paludis KC690141 Chitinophagaceae
Ferruginibacter alkalilentus FJ177530 Chitinophagaceae
Ferruginibacter lapsinanis FJ177532 Chitinophagaceae
Terrimonas terrae KX417306 Chitinophagaceae
Terrimonas crocea KX139009 Chitinophagaceae
Terrimonas arctica KJ631121 Chitinophagaceae
Terrimonas pekingensis JF834159 Chitinophagaceae
Terrimonas lutea AB192292 Chitinophagaceae
Terrimonas rhizosphaerae FJ772030 Chitinophagaceae
Niabella aquatica KT950742 Chitinophagaceae
Niabella soli TYGS 1010 Chitinophagaceae
Niabella drilacis TYGS 2638 Chitinophagaceae
Niabella tibetensis GU291295 Chitinophagaceae
Niabella pedocola KT032154 Chitinophagaceae
Niabella aurantiaca TYGS 1185 Chitinophagaceae
Niabella hirudinis KC307771 Chitinophagaceae
Niabella yanshanensis FJ457040 Chitinophagaceae
Niabella ginsengisoli EU616816 Chitinophagaceae
Niabella hibiscisoli KU505213 Chitinophagaceae
Terrimonas aquatica FJ347757 Chitinophagaceae
Terrimonas rubra JF803808 Chitinophagaceae
Terrimonas ferruginea TYGS 1391 Chitinophagaceae
Niastella hibisci KT962169 Chitinophagaceae
Niastella gongjuensis KM007102 Chitinophagaceae
Niastella populi TYGS 6418 Chitinophagaceae
Niastella koreensis TYGS 200 Chitinophagaceae
Niastella yeongjuensis DQ244076 Chitinophagaceae
Niastella vici TYGS 21970 Chitinophagaceae
Flavitalea soli KM007101 Chitinophagaceae
Pseudobacter ginsenosidimutans GQ339899 Chitinophagaceae
Pseudoflavitalea rhizosphaerae KU379667 Chitinophagaceae
Flavihumibacter solisilvae TYGS 3767 Chitinophagaceae
Flavihumibacter sediminis KP126826 Chitinophagaceae
Flavihumibacter stibioxidans KX014806 Chitinophagaceae
Flavihumibacter cheonanensis KF309176 Chitinophagaceae
Flavihumibacter petaseus TYGS 2826 Chitinophagaceae
Niveitalea solisilvae KX268597 Chitinophagaceae
Filobacterium rodentium LC055729 Filobacteriaceae
Crenotalea thermophila TYGS 10716 Chitinophagaceae
Thermoflavifilum aggregans TYGS 22652 Chitinophagaceae
Lentimicrobium saccharophilum TYGS 4845 Lentimicrobiaceae
Salibacter halophilus KU726562 Cryomorphaceae
Owenweeksia hongkongensis TYGS 137 Cryomorphaceae

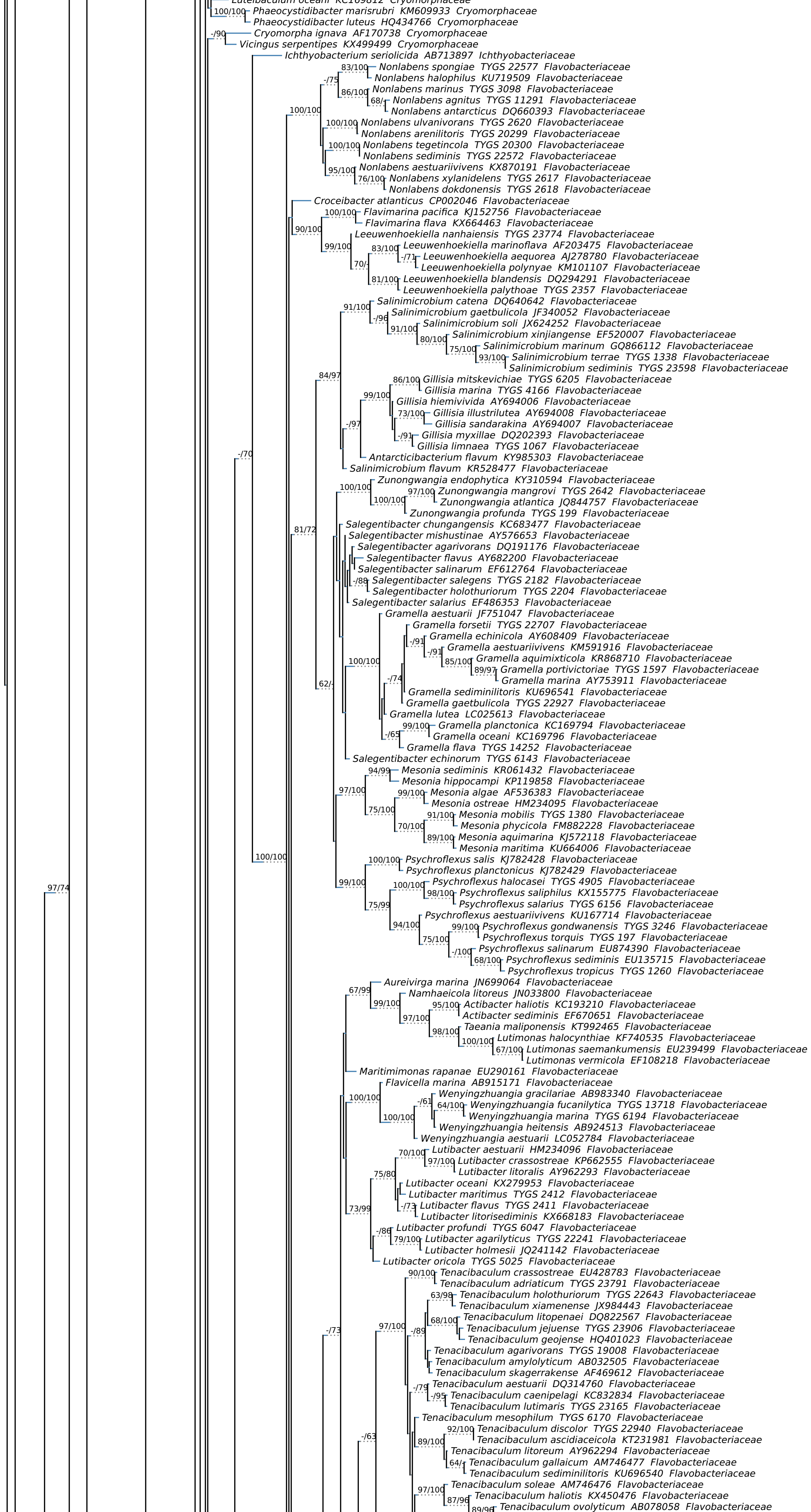
Luteibaculum oceani KC109812 Cryomorphaceae
Phaeocystidibacter marisrubri KM609933 Cryomorphaceae
Phaeocystidibacter luteus HQ434766 Cryomorphaceae
Cryomorpha ignava AF170738 Cryomorphaceae
Vicingus serpentipes KX499499 Cryomorphaceae
Ichthyobacterium seriolicida AB713897 Ichthyobacteriaceae
Nonlabens spongiae TYGS 22577 Flavobacteriaceae
Nonlabens halophilus KU719509 Flavobacteriaceae
Nonlabens marinus TYGS 3098 Flavobacteriaceae
Nonlabens agnitus TYGS 11291 Flavobacteriaceae
Nonlabens antarcticus DQ660393 Flavobacteriaceae
Nonlabens ulvanivorans TYGS 2620 Flavobacteriaceae
Nonlabens arenilitoris TYGS 20299 Flavobacteriaceae
Nonlabens tegetincola TYGS 20300 Flavobacteriaceae
Nonlabens sediminis TYGS 22572 Flavobacteriaceae
Nonlabens aestuariivivens KX870191 Flavobacteriaceae
Nonlabens xylanidelens TYGS 2617 Flavobacteriaceae
Nonlabens dokdonensis TYGS 2618 Flavobacteriaceae
Croceibacter atlanticus CP002046 Flavobacteriaceae
Flavimarina pacifica KJ152756 Flavobacteriaceae
Flavimarina flava KX664463 Flavobacteriaceae
Leeuwenhoekiella nanhaiensis TYGS 23774 Flavobacteriaceae
Leeuwenhoekiella marinoflava AF203475 Flavobacteriaceae
Leeuwenhoekiella aequorea AJ278780 Flavobacteriaceae
Leeuwenhoekiella polynyae KM101107 Flavobacteriaceae
Leeuwenhoekiella blandensis DQ294291 Flavobacteriaceae
Leeuwenhoekiella palythoae TYGS 2357 Flavobacteriaceae
Salinimicrobium catena DQ640642 Flavobacteriaceae
Salinimicrobium gaetbulicola JF340052 Flavobacteriaceae
Salinimicrobium soli JX624252 Flavobacteriaceae
Salinimicrobium xinjiangense EF520007 Flavobacteriaceae
Salinimicrobium marinum GQ866112 Flavobacteriaceae
Salinimicrobium terrae TYGS 1338 Flavobacteriaceae
Salinimicrobium sediminis TYGS 23598 Flavobacteriaceae
Gillisia mitskevichiae TYGS 6205 Flavobacteriaceae
Gillisia marina TYGS 4166 Flavobacteriaceae
Gillisia hiemivivida AY694006 Flavobacteriaceae
Gillisia illustrilutea AY694008 Flavobacteriaceae
Gillisia sandarakina AY694007 Flavobacteriaceae
Gillisia myxillae DQ202393 Flavobacteriaceae
Gillisia limnaea TYGS 1067 Flavobacteriaceae
Antarcticibacterium flavum KY985303 Flavobacteriaceae
Salinimicrobium flavum KR528477 Flavobacteriaceae
Zunongwangia endophytica KY310594 Flavobacteriaceae
Zunongwangia mangrovi TYGS 2642 Flavobacteriaceae
Zunongwangia atlantica JQ844757 Flavobacteriaceae
Zunongwangia profunda TYGS 199 Flavobacteriaceae
Salegentibacter chungangensis KC683477 Flavobacteriaceae
Salegentibacter mishustinae AY576653 Flavobacteriaceae
Salegentibacter agarivorans DQ191176 Flavobacteriaceae
Salegentibacter flavus AY682200 Flavobacteriaceae
Salegentibacter salinarum EF612764 Flavobacteriaceae
Salegentibacter salegens TYGS 2182 Flavobacteriaceae
Salegentibacter holothuriorum TYGS 2204 Flavobacteriaceae
Salegentibacter salarius EF486353 Flavobacteriaceae
Gramella aestuarii JF751047 Flavobacteriaceae
Gramella forsetii TYGS 22707 Flavobacteriaceae
Gramella echinicola AY608409 Flavobacteriaceae
Gramella aestuariivivens KM591916 Flavobacteriaceae
Gramella aquimixticola KR868710 Flavobacteriaceae
Gramella portivictoriae TYGS 1597 Flavobacteriaceae
Gramella marina AY753911 Flavobacteriaceae
Gramella sediminilitoris KU696541 Flavobacteriaceae
Gramella gaetbulicola TYGS 22927 Flavobacteriaceae
Gramella lutea LC025613 Flavobacteriaceae
Gramella planctonica KC169794 Flavobacteriaceae
Gramella oceani KC169796 Flavobacteriaceae
Gramella flava TYGS 14252 Flavobacteriaceae
Salegentibacter echinorum TYGS 6143 Flavobacteriaceae
Mesonia sediminis KR061432 Flavobacteriaceae
Mesonia hippocampi KP119858 Flavobacteriaceae
Mesonia algae AF536383 Flavobacteriaceae
Mesonia ostreae HM234095 Flavobacteriaceae
Mesonia mobilis TYGS 1380 Flavobacteriaceae
Mesonia phycicola FM882228 Flavobacteriaceae
Mesonia aquimarina KJ572118 Flavobacteriaceae
Mesonia maritima KU664006 Flavobacteriaceae
Psychroflexus salis KJ782428 Flavobacteriaceae
Psychroflexus planctonicus KJ782429 Flavobacteriaceae
Psychroflexus halocasei TYGS 4905 Flavobacteriaceae
Psychroflexus saliphilus KX155775 Flavobacteriaceae
Psychroflexus salarius TYGS 6156 Flavobacteriaceae
Psychroflexus aestuariivivens KU167714 Flavobacteriaceae
Psychroflexus gondwanensis TYGS 3246 Flavobacteriaceae
Psychroflexus torquis TYGS 197 Flavobacteriaceae
Psychroflexus salinarum EU874390 Flavobacteriaceae
Psychroflexus sediminis EU135715 Flavobacteriaceae
Psychroflexus tropicus TYGS 1260 Flavobacteriaceae
Aureivirga marina JN699064 Flavobacteriaceae
Namhaeicola litoreus JN033800 Flavobacteriaceae
Actibacter haliotis KC193210 Flavobacteriaceae
Actibacter sediminis EF670651 Flavobacteriaceae
Taeania maliponensis KT992465 Flavobacteriaceae
Lutimonas halocynthiae KF740535 Flavobacteriaceae
Lutimonas saemankumensis EU239499 Flavobacteriaceae
Lutimonas vermicola EF108218 Flavobacteriaceae
Maritimimonas rapanae EU290161 Flavobacteriaceae
Flavicella marina AB915171 Flavobacteriaceae
Wenyingzhuangia gracilariae AB983340 Flavobacteriaceae
Wenyingzhuangia fucanilytica TYGS 13718 Flavobacteriaceae
Wenyingzhuangia marina TYGS 6194 Flavobacteriaceae
Wenyingzhuangia heitensis AB924513 Flavobacteriaceae
Wenyingzhuangia aestuarii LC052784 Flavobacteriaceae
Lutibacter aestuarii HM234096 Flavobacteriaceae
Lutibacter crassostreae KP662555 Flavobacteriaceae
Lutibacter litoralis AY962293 Flavobacteriaceae
Lutibacter oceani KX279953 Flavobacteriaceae
Lutibacter maritimus TYGS 2412 Flavobacteriaceae
Lutibacter flavus TYGS 2411 Flavobacteriaceae
Lutibacter litorisediminis KX668183 Flavobacteriaceae
Lutibacter profundi TYGS 6047 Flavobacteriaceae
Lutibacter agarilyticus TYGS 22241 Flavobacteriaceae
Lutibacter holmesii JQ241142 Flavobacteriaceae
Lutibacter oricola TYGS 5025 Flavobacteriaceae
Tenacibaculum crassostreae EU428783 Flavobacteriaceae
Tenacibaculum adriaticum TYGS 23791 Flavobacteriaceae
Tenacibaculum holothuriorum TYGS 22643 Flavobacteriaceae
Tenacibaculum xiamenense JX984443 Flavobacteriaceae
Tenacibaculum litopenaei DQ822567 Flavobacteriaceae
Tenacibaculum jejuense TYGS 23906 Flavobacteriaceae
Tenacibaculum geojense HQ401023 Flavobacteriaceae
Tenacibaculum agarivorans TYGS 19008 Flavobacteriaceae
Tenacibaculum amylolyticum AB032505 Flavobacteriaceae
Tenacibaculum skagerrakense AF469612 Flavobacteriaceae
Tenacibaculum aestuarii DQ314760 Flavobacteriaceae
Tenacibaculum caenipelagi KC832834 Flavobacteriaceae
Tenacibaculum lutimaris TYGS 23165 Flavobacteriaceae
Tenacibaculum mesophilum TYGS 6170 Flavobacteriaceae
Tenacibaculum discolor TYGS 22940 Flavobacteriaceae
Tenacibaculum ascidiaceicola KT231981 Flavobacteriaceae
Tenacibaculum litoreum AY962294 Flavobacteriaceae
Tenacibaculum gallaicum AM746477 Flavobacteriaceae
Tenacibaculum sediminilitoris KU696540 Flavobacteriaceae
Tenacibaculum soleae AM746476 Flavobacteriaceae
Tenacibaculum haliotis KX450476 Flavobacteriaceae
Tenacibaculum ovolyticum AB078058 Flavobacteriaceae

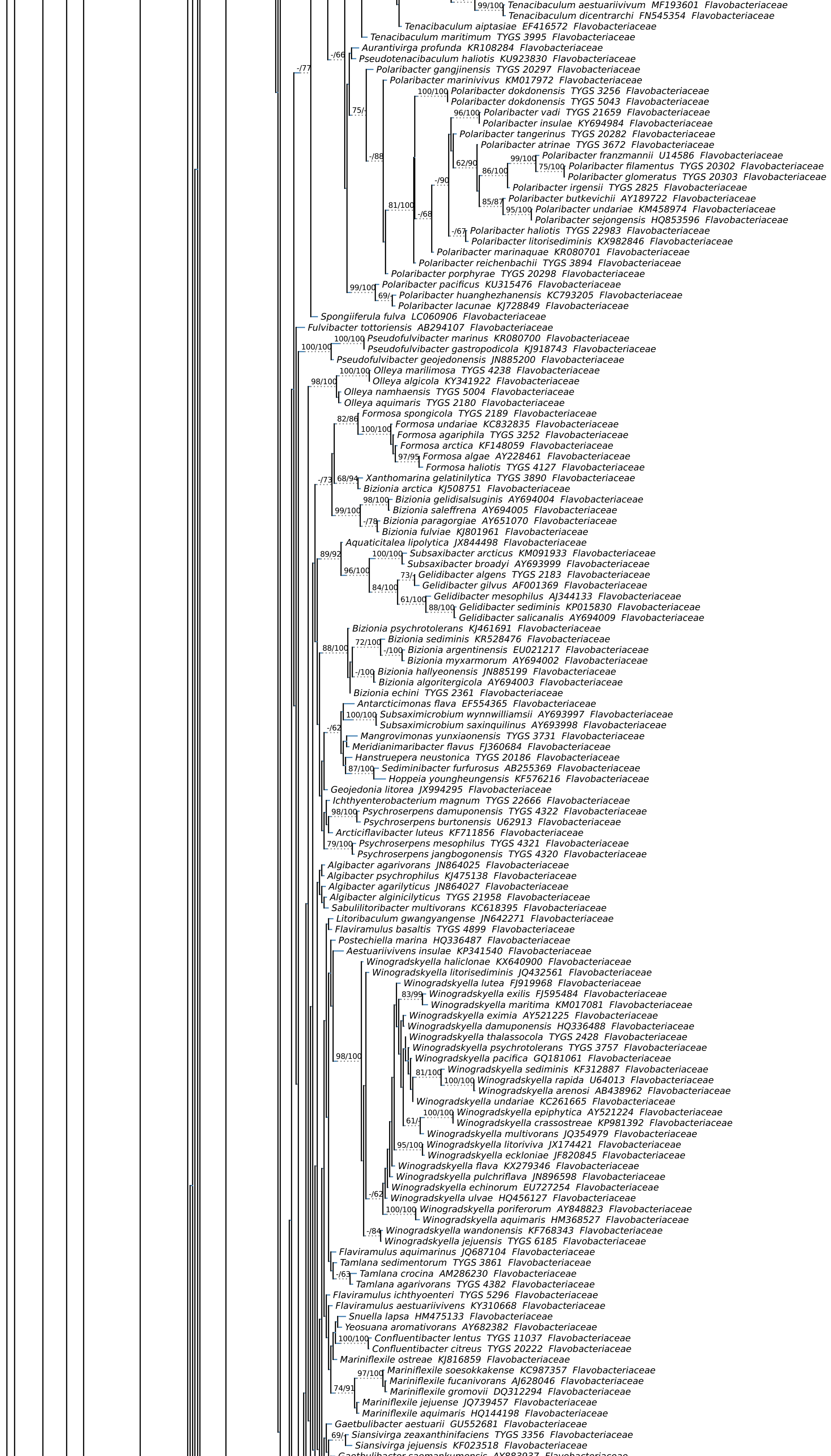

99/100 Tenacibaculum aestuariivivum MF193601 Flavobacteriaceae
Tenacibaculum dicentrarchi FN545354 Flavobacteriaceae
Tenacibaculum aiptasiae EF416572 Flavobacteriaceae
Tenacibaculum maritimum TYGS 3995 Flavobacteriaceae
Aurantivirga profunda KR108284 Flavobacteriaceae
-/66 Pseudotenacibaculum haliotis KU923830 Flavobacteriaceae
-/77
Polaribacter gangjinensis TYGS 20297 Flavobacteriaceae
Polaribacter marinivivus KM017972 Flavobacteriaceae
100/100 Polaribacter dokdonensis TYGS 3256 Flavobacteriaceae
Polaribacter dokdonensis TYGS 5043 Flavobacteriaceae
96/100 Polaribacter vadi TYGS 21659 Flavobacteriaceae
75/-
Polaribacter insulae KY694984 Flavobacteriaceae
Polaribacter tangerinus TYGS 20282 Flavobacteriaceae
Polaribacter atrinae TYGS 3672 Flavobacteriaceae
-/88
99/100 Polaribacter franzmannii U14586 Flavobacteriaceae
62/90
75/100 Polaribacter filamentus TYGS 20302 Flavobacteriaceae
86/100
Polaribacter glomeratus TYGS 20303 Flavobacteriaceae
-/90
Polaribacter irgensii TYGS 2825 Flavobacteriaceae
Polaribacter butkevichii AY189722 Flavobacteriaceae
85/87
81/100
95/100 Polaribacter undariae KM458974 Flavobacteriaceae
-/68
Polaribacter sejongensis HQ853596 Flavobacteriaceae
-/67 Polaribacter haliotis TYGS 22983 Flavobacteriaceae
Polaribacter litorisediminis KX982846 Flavobacteriaceae
Polaribacter marinaquae KR080701 Flavobacteriaceae
Polaribacter reichenbachii TYGS 3894 Flavobacteriaceae
Polaribacter porphyrae TYGS 20298 Flavobacteriaceae
99/100 Polaribacter pacificus KU315476 Flavobacteriaceae
69/- Polaribacter huanghezhanensis KC793205 Flavobacteriaceae
Polaribacter lacunae KJ728849 Flavobacteriaceae
Spongiiferula fulva LC060906 Flavobacteriaceae
Fulvibacter tottoriensis AB294107 Flavobacteriaceae
100/100 Pseudofulvibacter marinus KR080700 Flavobacteriaceae
100/100
Pseudofulvibacter gastropodicola KJ918743 Flavobacteriaceae
Pseudofulvibacter geojedonensis JN885200 Flavobacteriaceae
100/100 Olleya marilimosa TYGS 4238 Flavobacteriaceae
98/100
Olleya algicola KY341922 Flavobacteriaceae
Olleya namhaensis TYGS 5004 Flavobacteriaceae
Olleya aquimaris TYGS 2180 Flavobacteriaceae
Formosa spongicola TYGS 2189 Flavobacteriaceae
82/86
Formosa undariae KC832835 Flavobacteriaceae
100/100
Formosa agariphila TYGS 3252 Flavobacteriaceae
Formosa arctica KF148059 Flavobacteriaceae
97/95 Formosa algae AY228461 Flavobacteriaceae
Formosa haliotis TYGS 4127 Flavobacteriaceae
-/73 68/94 Xanthomarina gelatinilytica TYGS 3890 Flavobacteriaceae
Bizionia arctica KJ508751 Flavobacteriaceae
98/100 Bizionia gelidisalsuginis AY694004 Flavobacteriaceae
99/100
Bizionia saleffrena AY694005 Flavobacteriaceae
-/78 Bizionia paragorgiae AY651070 Flavobacteriaceae
Bizionia fulviae KJ801961 Flavobacteriaceae
Aquaticitalea lipolytica JX844498 Flavobacteriaceae
100/100 Subsaxibacter arcticus KM091933 Flavobacteriaceae
89/92
Subsaxibacter broadyi AY693999 Flavobacteriaceae
96/100
73/- Gelidibacter algens TYGS 2183 Flavobacteriaceae
Gelidibacter gilvus AF001369 Flavobacteriaceae
84/100
61/100 Gelidibacter mesophilus AJ344133 Flavobacteriaceae
88/100 Gelidibacter sediminis KP015830 Flavobacteriaceae
Gelidibacter salicanalis AY694009 Flavobacteriaceae
Bizionia psychrotolerans KJ461691 Flavobacteriaceae
72/100 Bizionia sediminis KR528476 Flavobacteriaceae
88/100
-/100 Bizionia argentinensis EU021217 Flavobacteriaceae
Bizionia myxarmorum AY694002 Flavobacteriaceae
-/100 Bizionia hallyeonensis JN885199 Flavobacteriaceae
Bizionia algoritergicola AY694003 Flavobacteriaceae
Bizionia echini TYGS 2361 Flavobacteriaceae
Antarcticimonas flava EF554365 Flavobacteriaceae
100/100 Subsaximicrobium wynnwilliamsii AY693997 Flavobacteriaceae
-/62
Subsaximicrobium saxinquilinus AY693998 Flavobacteriaceae
Mangrovimonas yunxiaonensis TYGS 3731 Flavobacteriaceae
Meridianimaribacter flavus FJ360684 Flavobacteriaceae
Hanstruepera neustonica TYGS 20186 Flavobacteriaceae
87/100 Sediminibacter furfurosus AB255369 Flavobacteriaceae
Hoppeia youngheungensis KF576216 Flavobacteriaceae
Geojedonia litorea JX994295 Flavobacteriaceae
Ichthyenterobacterium magnum TYGS 22666 Flavobacteriaceae
98/100 Psychroserpens damuponensis TYGS 4322 Flavobacteriaceae
Psychroserpens burtonensis U62913 Flavobacteriaceae
Arcticiflavibacter luteus KF711856 Flavobacteriaceae
79/100 Psychroserpens mesophilus TYGS 4321 Flavobacteriaceae
Psychroserpens jangbogonensis TYGS 4320 Flavobacteriaceae
Algibacter agarivorans JN864025 Flavobacteriaceae
Algibacter psychrophilus KJ475138 Flavobacteriaceae
Algibacter agarilyticus JN864027 Flavobacteriaceae
Algibacter alginicilyticus TYGS 21958 Flavobacteriaceae
Sabulilitoribacter multivorans KC618395 Flavobacteriaceae
Litoribaculum gwangyangense JN642271 Flavobacteriaceae
Flaviramulus basaltis TYGS 4899 Flavobacteriaceae
Postechiella marina HQ336487 Flavobacteriaceae
Aestuariivivens insulae KP341540 Flavobacteriaceae
Winogradskyella haliclonae KX640900 Flavobacteriaceae
Winogradskyella litorisediminis JQ432561 Flavobacteriaceae
Winogradskyella lutea FJ919968 Flavobacteriaceae
83/99 Winogradskyella exilis FJ595484 Flavobacteriaceae
Winogradskyella maritima KM017081 Flavobacteriaceae
Winogradskyella eximia AY521225 Flavobacteriaceae
Winogradskyella damuponensis HQ336488 Flavobacteriaceae
Winogradskyella thalassocola TYGS 2428 Flavobacteriaceae
Winogradskyella psychrotolerans TYGS 3757 Flavobacteriaceae
98/100
Winogradskyella pacifica GQ181061 Flavobacteriaceae
81/100 Winogradskyella sediminis KF312887 Flavobacteriaceae
100/100 Winogradskyella rapida U64013 Flavobacteriaceae
Winogradskyella arenosi AB438962 Flavobacteriaceae
Winogradskyella undariae KC261665 Flavobacteriaceae
100/100 Winogradskyella epiphytica AY521224 Flavobacteriaceae
61/- Winogradskyella crassostreae KP981392 Flavobacteriaceae
Winogradskyella multivorans JQ354979 Flavobacteriaceae
95/100 Winogradskyella litoriviva JX174421 Flavobacteriaceae
Winogradskyella eckloniae JF820845 Flavobacteriaceae
Winogradskyella flava KX279346 Flavobacteriaceae
Winogradskyella pulchriflava JN896598 Flavobacteriaceae
Winogradskyella echinorum EU727254 Flavobacteriaceae
-/62
Winogradskyella ulvae HQ456127 Flavobacteriaceae
100/100 Winogradskyella poriferorum AY848823 Flavobacteriaceae
Winogradskyella aquimaris HM368527 Flavobacteriaceae
-/84 Winogradskyella wandonensis KF768343 Flavobacteriaceae
Winogradskyella jejuensis TYGS 6185 Flavobacteriaceae
Flaviramulus aquimarinus JQ687104 Flavobacteriaceae
Tamlana sedimentorum TYGS 3861 Flavobacteriaceae
-/63 Tamlana crocina AM286230 Flavobacteriaceae
Tamlana agarivorans TYGS 4382 Flavobacteriaceae
Flaviramulus ichthyoenteri TYGS 5296 Flavobacteriaceae
Flaviramulus aestuariivivens KY310668 Flavobacteriaceae
Snuella lapsa HM475133 Flavobacteriaceae
Yeosuana aromativorans AY682382 Flavobacteriaceae
100/100 Confluentibacter lentus TYGS 11037 Flavobacteriaceae
Confluentibacter citreus TYGS 20222 Flavobacteriaceae
Mariniflexile ostreae KJ816859 Flavobacteriaceae
97/100 Mariniflexile soesokkakense KC987357 Flavobacteriaceae
Mariniflexile fucanivorans AJ628046 Flavobacteriaceae
74/91
Mariniflexile gromovii DQ312294 Flavobacteriaceae
Mariniflexile jejuense JQ739457 Flavobacteriaceae
Mariniflexile aquimaris HQ144198 Flavobacteriaceae
Gaetbulibacter aestuarii GU552681 Flavobacteriaceae
69/- Siansivirga zeaxanthinifaciens TYGS 3356 Flavobacteriaceae
Siansivirga jejuensis KF023518 Flavobacteriaceae

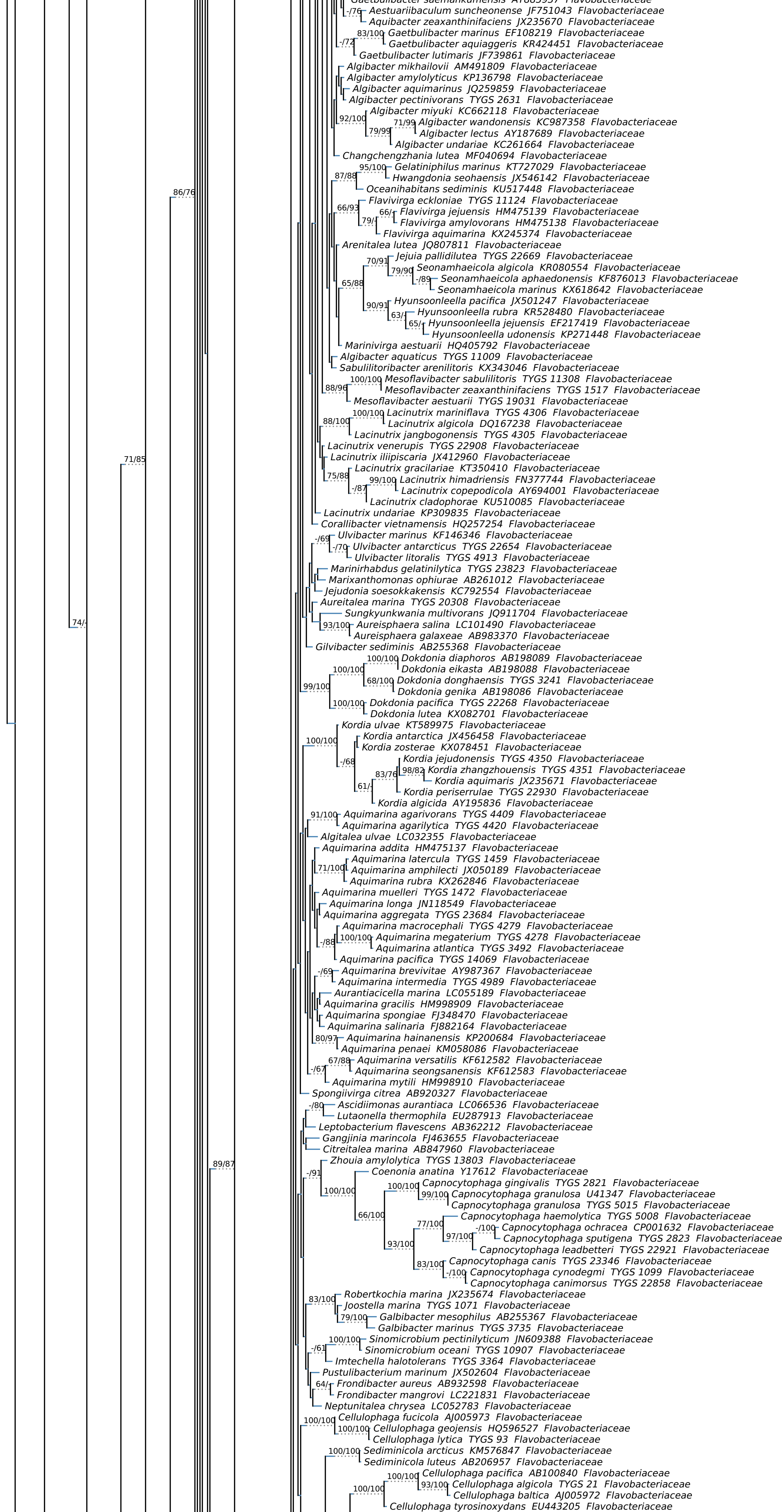
Aestuariibaculum suncheonense JF751043 Flavobacteriaceae
Aquibacter zeaxanthinifaciens JX235670 Flavobacteriaceae
Gaetbulibacter marinus EF108219 Flavobacteriaceae
Gaetbulibacter aquiaggeris KR424451 Flavobacteriaceae
Gaetbulibacter lutimaris JF739861 Flavobacteriaceae
Algibacter mikhailovii AM491809 Flavobacteriaceae
Algibacter amylolyticus KP136798 Flavobacteriaceae
Algibacter aquimarinus JQ259859 Flavobacteriaceae
Algibacter pectinivorans TYGS 2631 Flavobacteriaceae
Algibacter miyuki KC662118 Flavobacteriaceae
Algibacter wandonensis KC987358 Flavobacteriaceae
Algibacter lectus AY187689 Flavobacteriaceae
Algibacter undariae KC261664 Flavobacteriaceae
Changchengzhania lutea MF040694 Flavobacteriaceae
Gelatiniphilus marinus KT727029 Flavobacteriaceae
Hwangdonia seohaensis JX546142 Flavobacteriaceae
Oceanihabitans sediminis KU517448 Flavobacteriaceae
Flavivirga eckloniae TYGS 11124 Flavobacteriaceae
Flavivirga jejuensis HM475139 Flavobacteriaceae
Flavivirga amylovorans HM475138 Flavobacteriaceae
Flavivirga aquimarina KX245374 Flavobacteriaceae
Arenitalea lutea JQ807811 Flavobacteriaceae
Jejuia pallidilutea TYGS 22669 Flavobacteriaceae
Seonamhaeicola algicola KR080554 Flavobacteriaceae
Seonamhaeicola aphaedonensis KF876013 Flavobacteriaceae
Seonamhaeicola marinus KX618642 Flavobacteriaceae
Hyunsoonleella pacifica JX501247 Flavobacteriaceae
Hyunsoonleella rubra KR528480 Flavobacteriaceae
Hyunsoonleella jejuensis EF217419 Flavobacteriaceae
Hyunsoonleella udonensis KP271448 Flavobacteriaceae
Marinivirga aestuarii HQ405792 Flavobacteriaceae
Algibacter aquaticus TYGS 11009 Flavobacteriaceae
Sabulilitoribacter arenilitoris KX343046 Flavobacteriaceae
Mesoflavibacter sabulilitoris TYGS 11308 Flavobacteriaceae
Mesoflavibacter zeaxanthinifaciens TYGS 1517 Flavobacteriaceae
Mesoflavibacter aestuarii TYGS 19031 Flavobacteriaceae
Lacinutrix mariniflava TYGS 4306 Flavobacteriaceae
Lacinutrix algicola DQ167238 Flavobacteriaceae
Lacinutrix jangbogonensis TYGS 4305 Flavobacteriaceae
Lacinutrix venerupis TYGS 22908 Flavobacteriaceae
Lacinutrix iliipiscaria JX412960 Flavobacteriaceae
Lacinutrix gracilariae KT350410 Flavobacteriaceae
Lacinutrix himadriensis FN377744 Flavobacteriaceae
Lacinutrix copepodicola AY694001 Flavobacteriaceae
Lacinutrix cladophorae KU510085 Flavobacteriaceae
Lacinutrix undariae KP309835 Flavobacteriaceae
Corallibacter vietnamensis HQ257254 Flavobacteriaceae
Ulvibacter marinus KF146346 Flavobacteriaceae
Ulvibacter antarcticus TYGS 22654 Flavobacteriaceae
Ulvibacter litoralis TYGS 4913 Flavobacteriaceae
Marinirhabdus gelatinilytica TYGS 23823 Flavobacteriaceae
Marixanthomonas ophiurae AB261012 Flavobacteriaceae
Jejudonia soesokkakensis KC792554 Flavobacteriaceae
Aureitalea marina TYGS 20308 Flavobacteriaceae
Sungkyunkwania multivorans JQ911704 Flavobacteriaceae
Aureisphaera salina LC101490 Flavobacteriaceae
Aureisphaera galaxeae AB983370 Flavobacteriaceae
Gilvibacter sediminis AB255368 Flavobacteriaceae
Dokdonia diaphoros AB198089 Flavobacteriaceae
Dokdonia eikasta AB198088 Flavobacteriaceae
Dokdonia donghaensis TYGS 3241 Flavobacteriaceae
Dokdonia genika AB198086 Flavobacteriaceae
Dokdonia pacifica TYGS 22268 Flavobacteriaceae
Dokdonia lutea KX082701 Flavobacteriaceae
Kordia ulvae KT589975 Flavobacteriaceae
Kordia antarctica JX456458 Flavobacteriaceae
Kordia zosterae KX078451 Flavobacteriaceae
Kordia jejudonensis TYGS 4350 Flavobacteriaceae
Kordia zhangzhouensis TYGS 4351 Flavobacteriaceae
Kordia aquimaris JX235671 Flavobacteriaceae
Kordia periserrulae TYGS 22930 Flavobacteriaceae
Kordia algicida AY195836 Flavobacteriaceae
Aquimarina agarivorans TYGS 4409 Flavobacteriaceae
Aquimarina agarilytica TYGS 4420 Flavobacteriaceae
Algitalea ulvae LC032355 Flavobacteriaceae
Aquimarina addita HM475137 Flavobacteriaceae
Aquimarina latercula TYGS 1459 Flavobacteriaceae
Aquimarina amphilecti JX050189 Flavobacteriaceae
Aquimarina rubra KX262846 Flavobacteriaceae
Aquimarina muelleri TYGS 1472 Flavobacteriaceae
Aquimarina longa JN118549 Flavobacteriaceae
Aquimarina aggregata TYGS 23684 Flavobacteriaceae
Aquimarina macrocephali TYGS 4279 Flavobacteriaceae
Aquimarina megaterium TYGS 4278 Flavobacteriaceae
Aquimarina atlantica TYGS 3492 Flavobacteriaceae
Aquimarina pacifica TYGS 14069 Flavobacteriaceae
Aquimarina brevivitae AY987367 Flavobacteriaceae
Aquimarina intermedia TYGS 4989 Flavobacteriaceae
Aurantiacicella marina LC055189 Flavobacteriaceae
Aquimarina gracilis HM998909 Flavobacteriaceae
Aquimarina spongiae FJ348470 Flavobacteriaceae
Aquimarina salinaria FJ882164 Flavobacteriaceae
Aquimarina hainanensis KP200684 Flavobacteriaceae
Aquimarina penaei KM058086 Flavobacteriaceae
Aquimarina versatilis KF612582 Flavobacteriaceae
Aquimarina seongsanensis KF612583 Flavobacteriaceae
Aquimarina mytili HM998910 Flavobacteriaceae
Spongiivirga citrea AB920327 Flavobacteriaceae
Ascidiimonas aurantiaca LC066536 Flavobacteriaceae
Lutaonella thermophila EU287913 Flavobacteriaceae
Leptobacterium flavescens AB362212 Flavobacteriaceae
Gangjinia marincola FJ463655 Flavobacteriaceae
Citreitalea marina AB847960 Flavobacteriaceae
Zhouia amylolytica TYGS 13803 Flavobacteriaceae
Coenonia anatina Y17612 Flavobacteriaceae
Capnocytophaga gingivalis TYGS 2821 Flavobacteriaceae
Capnocytophaga granulosa U41347 Flavobacteriaceae
Capnocytophaga granulosa TYGS 5015 Flavobacteriaceae
Capnocytophaga haemolytica TYGS 5008 Flavobacteriaceae
Capnocytophaga ochracea CP001632 Flavobacteriaceae
Capnocytophaga sputigena TYGS 2823 Flavobacteriaceae
Capnocytophaga leadbetteri TYGS 22921 Flavobacteriaceae
Capnocytophaga canis TYGS 23346 Flavobacteriaceae
Capnocytophaga cynodegmi TYGS 1099 Flavobacteriaceae
Capnocytophaga canimorsus TYGS 22858 Flavobacteriaceae
Robertkochia marina JX235674 Flavobacteriaceae
Joostella marina TYGS 1071 Flavobacteriaceae
Galbibacter mesophilus AB255367 Flavobacteriaceae
Galbibacter marinus TYGS 3735 Flavobacteriaceae
Sinomicrobium pectinilyticum JN609388 Flavobacteriaceae
Sinomicrobium oceani TYGS 10907 Flavobacteriaceae
Imtechella halotolerans TYGS 3364 Flavobacteriaceae
Pustulibacterium marinum JX502604 Flavobacteriaceae
Frondibacter aureus AB932598 Flavobacteriaceae
Frondibacter mangrovi LC221831 Flavobacteriaceae
Neptunitalea chrysea LC052783 Flavobacteriaceae
Cellulophaga fucicola AJ005973 Flavobacteriaceae
Cellulophaga geojensis HQ596527 Flavobacteriaceae
Cellulophaga lytica TYGS 93 Flavobacteriaceae
Sediminicola arcticus KM576847 Flavobacteriaceae
Sediminicola luteus AB206957 Flavobacteriaceae
Cellulophaga pacifica AB100840 Flavobacteriaceae
Cellulophaga algicola TYGS 21 Flavobacteriaceae
Cellulophaga baltica AJ005972 Flavobacteriaceae
Cellulophaga tyrosinoxydans EU443205 Flavobacteriaceae

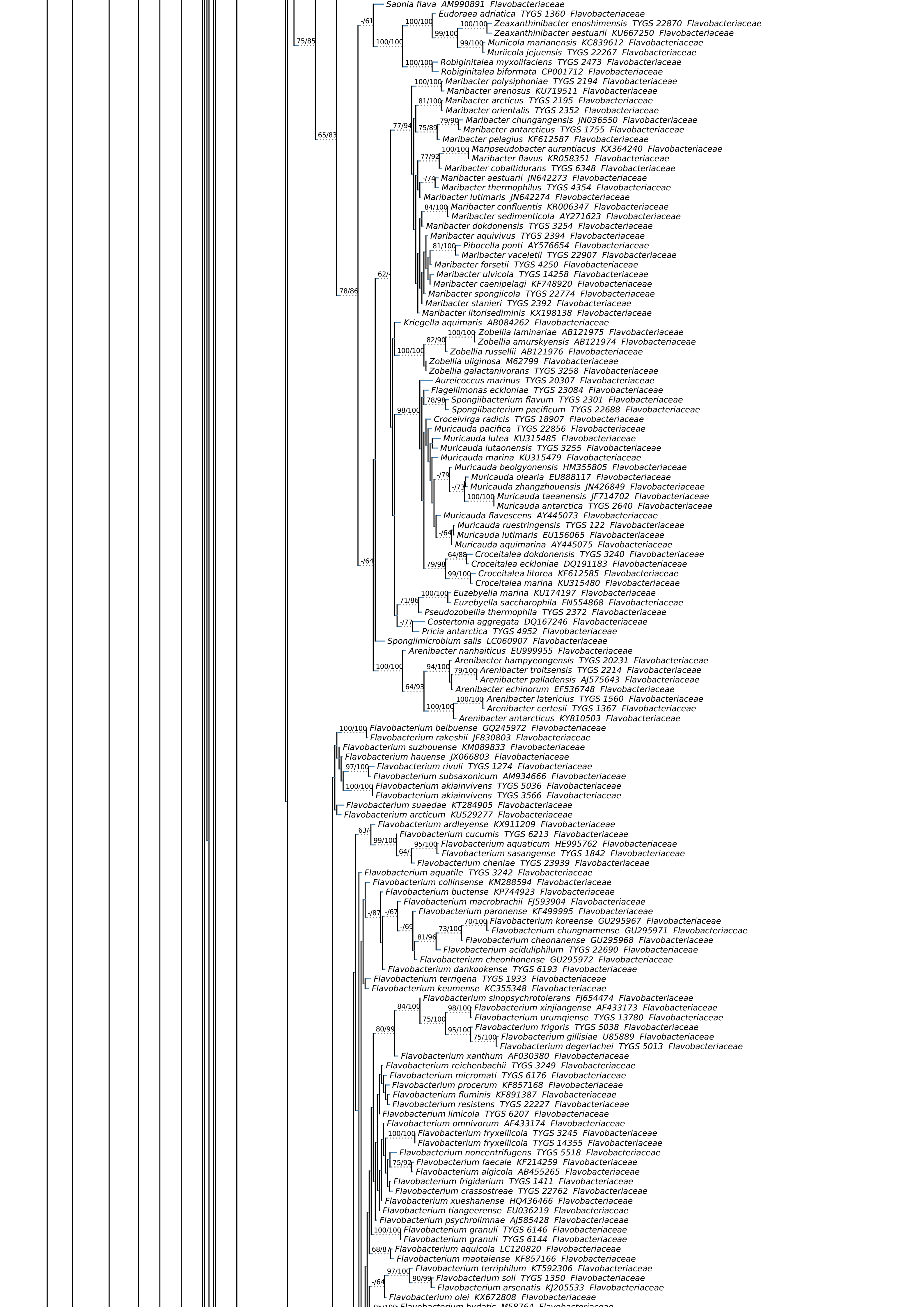
Saonia flava AM990891 Flavobacteriaceae
Eudoraea adriatica TYGS 1360 Flavobacteriaceae
Zeaxanthinibacter enoshimensis TYGS 22870 Flavobacteriaceae
Zeaxanthinibacter aestuarii KU667250 Flavobacteriaceae
Muriicola marianensis KC839612 Flavobacteriaceae
Muriicola jejuensis TYGS 22267 Flavobacteriaceae
Robiginitalea myxolifaciens TYGS 2473 Flavobacteriaceae
Robiginitalea biformata CP001712 Flavobacteriaceae
Maribacter polysiphoniae TYGS 2194 Flavobacteriaceae
Maribacter arenosus KU719511 Flavobacteriaceae
Maribacter arcticus TYGS 2195 Flavobacteriaceae
Maribacter orientalis TYGS 2352 Flavobacteriaceae
Maribacter chungangensis JN036550 Flavobacteriaceae
Maribacter antarcticus TYGS 1755 Flavobacteriaceae
Maribacter pelagius KF612587 Flavobacteriaceae
Maripseudobacter aurantiacus KX364240 Flavobacteriaceae
Maribacter flavus KR058351 Flavobacteriaceae
Maribacter cobaltidurans TYGS 6348 Flavobacteriaceae
Maribacter aestuarii JN642273 Flavobacteriaceae
Maribacter thermophilus TYGS 4354 Flavobacteriaceae
Maribacter lutimaris JN642274 Flavobacteriaceae
Maribacter confluentis KR006347 Flavobacteriaceae
Maribacter sedimenticola AY271623 Flavobacteriaceae
Maribacter dokdonensis TYGS 3254 Flavobacteriaceae
Maribacter aquivivus TYGS 2394 Flavobacteriaceae
Pibocella ponti AY576654 Flavobacteriaceae
Maribacter vaceletii TYGS 22907 Flavobacteriaceae
Maribacter forsetii TYGS 4250 Flavobacteriaceae
Maribacter ulvicola TYGS 14258 Flavobacteriaceae
Maribacter caenipelagi KF748920 Flavobacteriaceae
Maribacter spongiicola TYGS 22774 Flavobacteriaceae
Maribacter stanieri TYGS 2392 Flavobacteriaceae
Maribacter litorisediminis KX198138 Flavobacteriaceae
Kriegella aquimaris AB084262 Flavobacteriaceae
Zobellia laminariae AB121975 Flavobacteriaceae
Zobellia amurskyensis AB121974 Flavobacteriaceae
Zobellia russellii AB121976 Flavobacteriaceae
Zobellia uliginosa M62799 Flavobacteriaceae
Zobellia galactanivorans TYGS 3258 Flavobacteriaceae
Aureicoccus marinus TYGS 20307 Flavobacteriaceae
Flagellimonas eckloniae TYGS 23084 Flavobacteriaceae
Spongiibacterium flavum TYGS 2301 Flavobacteriaceae
Spongiibacterium pacificum TYGS 22688 Flavobacteriaceae
Croceivirga radicis TYGS 18907 Flavobacteriaceae
Muricauda pacifica TYGS 22856 Flavobacteriaceae
Muricauda lutea KU315485 Flavobacteriaceae
Muricauda lutaonensis TYGS 3255 Flavobacteriaceae
Muricauda marina KU315479 Flavobacteriaceae
Muricauda beolgyonensis HM355805 Flavobacteriaceae
Muricauda olearia EU888117 Flavobacteriaceae
Muricauda zhangzhouensis JN426849 Flavobacteriaceae
Muricauda taeanensis JF714702 Flavobacteriaceae
Muricauda antarctica TYGS 2640 Flavobacteriaceae
Muricauda flavescens AY445073 Flavobacteriaceae
Muricauda ruestringensis TYGS 122 Flavobacteriaceae
Muricauda lutimaris EU156065 Flavobacteriaceae
Muricauda aquimarina AY445075 Flavobacteriaceae
Croceitalea dokdonensis TYGS 3240 Flavobacteriaceae
Croceitalea eckloniae DQ191183 Flavobacteriaceae
Croceitalea litorea KF612585 Flavobacteriaceae
Croceitalea marina KU315480 Flavobacteriaceae
Euzebyella marina KU174197 Flavobacteriaceae
Euzebyella saccharophila FN554868 Flavobacteriaceae
Pseudozobellia thermophila TYGS 2372 Flavobacteriaceae
Costertonia aggregata DQ167246 Flavobacteriaceae
Pricia antarctica TYGS 4952 Flavobacteriaceae
Spongiimicrobium salis LC060907 Flavobacteriaceae
Arenibacter nanhaiticus EU999955 Flavobacteriaceae
Arenibacter hampyeongensis TYGS 20231 Flavobacteriaceae
Arenibacter troitsensis TYGS 2214 Flavobacteriaceae
Arenibacter palladensis AJ575643 Flavobacteriaceae
Arenibacter echinorum EF536748 Flavobacteriaceae
Arenibacter latericius TYGS 1560 Flavobacteriaceae
Arenibacter certesii TYGS 1367 Flavobacteriaceae
Arenibacter antarcticus KY810503 Flavobacteriaceae
Flavobacterium beibuense GQ245972 Flavobacteriaceae
Flavobacterium rakeshii JF830803 Flavobacteriaceae
Flavobacterium suzhouense KM089833 Flavobacteriaceae
Flavobacterium hauense JX066803 Flavobacteriaceae
Flavobacterium rivuli TYGS 1274 Flavobacteriaceae
Flavobacterium subsaxonicum AM934666 Flavobacteriaceae
Flavobacterium akiainvivens TYGS 5036 Flavobacteriaceae
Flavobacterium akiainvivens TYGS 3566 Flavobacteriaceae
Flavobacterium suaedae KT284905 Flavobacteriaceae
Flavobacterium arcticum KU529277 Flavobacteriaceae
Flavobacterium ardleyense KX911209 Flavobacteriaceae
Flavobacterium cucumis TYGS 6213 Flavobacteriaceae
Flavobacterium aquaticum HE995762 Flavobacteriaceae
Flavobacterium sasangense TYGS 1842 Flavobacteriaceae
Flavobacterium cheniae TYGS 23939 Flavobacteriaceae
Flavobacterium aquatile TYGS 3242 Flavobacteriaceae
Flavobacterium collinsense KM288594 Flavobacteriaceae
Flavobacterium buctense KP744923 Flavobacteriaceae
Flavobacterium macrobrachii FJ593904 Flavobacteriaceae
Flavobacterium paronense KF499995 Flavobacteriaceae
Flavobacterium koreense GU295967 Flavobacteriaceae
Flavobacterium chungnamense GU295971 Flavobacteriaceae
Flavobacterium cheonanense GU295968 Flavobacteriaceae
Flavobacterium aciduliphilum TYGS 22690 Flavobacteriaceae
Flavobacterium cheonhonense GU295972 Flavobacteriaceae
Flavobacterium dankookense TYGS 6193 Flavobacteriaceae
Flavobacterium terrigena TYGS 1933 Flavobacteriaceae
Flavobacterium keumense KC355348 Flavobacteriaceae
Flavobacterium sinopsychrotolerans FJ654474 Flavobacteriaceae
Flavobacterium xinjiangense AF433173 Flavobacteriaceae
Flavobacterium urumqiense TYGS 13780 Flavobacteriaceae
Flavobacterium frigoris TYGS 5038 Flavobacteriaceae
Flavobacterium gillisiae U85889 Flavobacteriaceae
Flavobacterium degerlachei TYGS 5013 Flavobacteriaceae
Flavobacterium xanthum AF030380 Flavobacteriaceae
Flavobacterium reichenbachii TYGS 3249 Flavobacteriaceae
Flavobacterium micromati TYGS 6176 Flavobacteriaceae
Flavobacterium procerum KF857168 Flavobacteriaceae
Flavobacterium fluminis KF891387 Flavobacteriaceae
Flavobacterium resistens TYGS 22227 Flavobacteriaceae
Flavobacterium limicola TYGS 6207 Flavobacteriaceae
Flavobacterium omnivorum AF433174 Flavobacteriaceae
Flavobacterium fryxellicola TYGS 3245 Flavobacteriaceae
Flavobacterium fryxellicola TYGS 14355 Flavobacteriaceae
Flavobacterium noncentrifugens TYGS 5518 Flavobacteriaceae
Flavobacterium faecale KF214259 Flavobacteriaceae
Flavobacterium algicola AB455265 Flavobacteriaceae
Flavobacterium frigidarium TYGS 1411 Flavobacteriaceae
Flavobacterium crassostreae TYGS 22762 Flavobacteriaceae
Flavobacterium xueshanense HQ436466 Flavobacteriaceae
Flavobacterium tiangeerense EU036219 Flavobacteriaceae
Flavobacterium psychrolimnae AJ585428 Flavobacteriaceae
Flavobacterium granuli TYGS 6146 Flavobacteriaceae
Flavobacterium granuli TYGS 6144 Flavobacteriaceae
Flavobacterium aquicola LC120820 Flavobacteriaceae
Flavobacterium maotaiense KF857166 Flavobacteriaceae
Flavobacterium terriphilum KT592306 Flavobacteriaceae
Flavobacterium soli TYGS 1350 Flavobacteriaceae
Flavobacterium arsenatis KJ205533 Flavobacteriaceae
Flavobacterium olei KX672808 Flavobacteriaceae

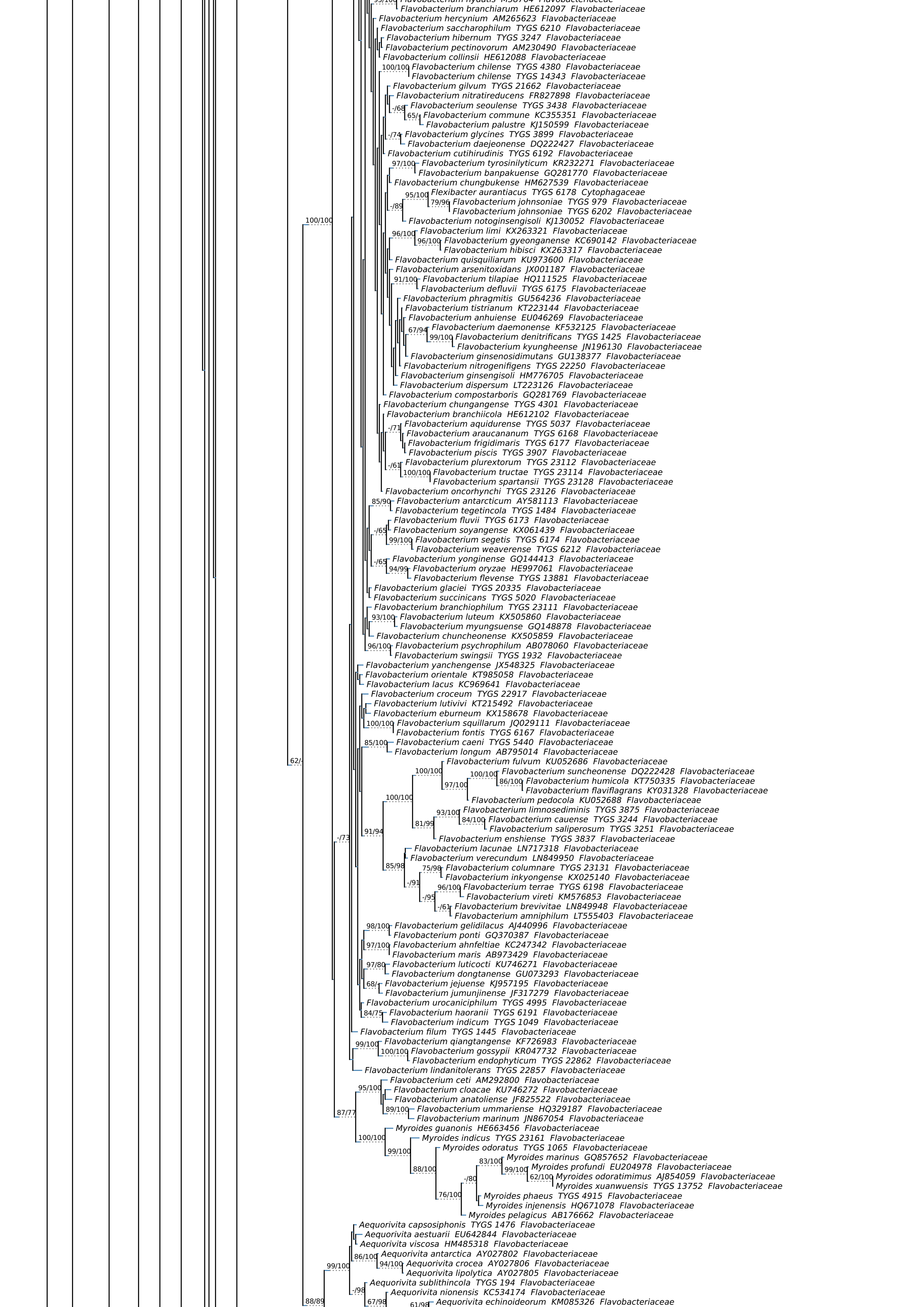
Flavobacterium branchiarum HE612097 Flavobacteriaceae
Flavobacterium hercynium AM265623 Flavobacteriaceae
Flavobacterium saccharophilum TYGS 6210 Flavobacteriaceae
Flavobacterium hibernum TYGS 3247 Flavobacteriaceae
Flavobacterium pectinovorum AM230490 Flavobacteriaceae
Flavobacterium collinsii HE612088 Flavobacteriaceae
100/100
Flavobacterium chilense TYGS 4380 Flavobacteriaceae
Flavobacterium chilense TYGS 14343 Flavobacteriaceae
Flavobacterium gilvum TYGS 21662 Flavobacteriaceae
Flavobacterium nitratireducens FR827898 Flavobacteriaceae
-/68
Flavobacterium seoulense TYGS 3438 Flavobacteriaceae
65/-
Flavobacterium commune KC355351 Flavobacteriaceae
Flavobacterium palustre KJ150599 Flavobacteriaceae
-/74
Flavobacterium glycines TYGS 3899 Flavobacteriaceae
Flavobacterium daejeonense DQ222427 Flavobacteriaceae
Flavobacterium cutihirudinis TYGS 6192 Flavobacteriaceae
97/100
Flavobacterium tyrosinilyticum KR232271 Flavobacteriaceae
Flavobacterium banpakuense GQ281770 Flavobacteriaceae
Flavobacterium chungbukense HM627539 Flavobacteriaceae
95/100
Flexibacter aurantiacus TYGS 6178 Cytophagaceae
-/89
79/96
Flavobacterium johnsoniae TYGS 979 Flavobacteriaceae
Flavobacterium johnsoniae TYGS 6202 Flavobacteriaceae
100/100
Flavobacterium notoginsengisoli KJ130052 Flavobacteriaceae
96/100
Flavobacterium limi KX263321 Flavobacteriaceae
96/100
Flavobacterium gyeonganense KC690142 Flavobacteriaceae
Flavobacterium hibisci KX263317 Flavobacteriaceae
Flavobacterium quisquiliarum KU973600 Flavobacteriaceae
Flavobacterium arsenitoxidans JX001187 Flavobacteriaceae
91/100
Flavobacterium tilapiae HQ111525 Flavobacteriaceae
Flavobacterium defluvii TYGS 6175 Flavobacteriaceae
Flavobacterium phragmitis GU564236 Flavobacteriaceae
Flavobacterium tistrianum KT223144 Flavobacteriaceae
Flavobacterium anhuiense EU046269 Flavobacteriaceae
67/94
Flavobacterium daemonense KF532125 Flavobacteriaceae
99/100
Flavobacterium denitrificans TYGS 1425 Flavobacteriaceae
Flavobacterium kyungheense JN196130 Flavobacteriaceae
Flavobacterium ginsenosidimutans GU138377 Flavobacteriaceae
Flavobacterium nitrogenifigens TYGS 22250 Flavobacteriaceae
Flavobacterium ginsengisoli HM776705 Flavobacteriaceae
Flavobacterium dispersum LT223126 Flavobacteriaceae
Flavobacterium compostarboris GQ281769 Flavobacteriaceae
Flavobacterium chungangense TYGS 4301 Flavobacteriaceae
Flavobacterium branchiicola HE612102 Flavobacteriaceae
-/71
Flavobacterium aquidurense TYGS 5037 Flavobacteriaceae
Flavobacterium araucananum TYGS 6168 Flavobacteriaceae
Flavobacterium frigidimaris TYGS 6177 Flavobacteriaceae
Flavobacterium piscis TYGS 3907 Flavobacteriaceae
-/61
Flavobacterium plurextorum TYGS 23112 Flavobacteriaceae
100/100
Flavobacterium tructae TYGS 23114 Flavobacteriaceae
Flavobacterium spartansii TYGS 23128 Flavobacteriaceae
Flavobacterium oncorhynchi TYGS 23126 Flavobacteriaceae
85/90
Flavobacterium antarcticum AY581113 Flavobacteriaceae
Flavobacterium tegetincola TYGS 1484 Flavobacteriaceae
Flavobacterium fluvii TYGS 6173 Flavobacteriaceae
-/65
Flavobacterium soyangense KX061439 Flavobacteriaceae
99/100
Flavobacterium segetis TYGS 6174 Flavobacteriaceae
Flavobacterium weaverense TYGS 6212 Flavobacteriaceae
-/65
Flavobacterium yonginense GQ144413 Flavobacteriaceae
94/99
Flavobacterium oryzae HE997061 Flavobacteriaceae
Flavobacterium flevense TYGS 13881 Flavobacteriaceae
Flavobacterium glaciei TYGS 20335 Flavobacteriaceae
Flavobacterium succinicans TYGS 5020 Flavobacteriaceae
Flavobacterium branchiophilum TYGS 23111 Flavobacteriaceae
93/100
Flavobacterium luteum KX505860 Flavobacteriaceae
Flavobacterium myungsuense GQ148878 Flavobacteriaceae
Flavobacterium chuncheonense KX505859 Flavobacteriaceae
96/100
Flavobacterium psychrophilum AB078060 Flavobacteriaceae
Flavobacterium swingsii TYGS 1932 Flavobacteriaceae
Flavobacterium yanchengense JX548325 Flavobacteriaceae
Flavobacterium orientale KT985058 Flavobacteriaceae
Flavobacterium lacus KC969641 Flavobacteriaceae
Flavobacterium croceum TYGS 22917 Flavobacteriaceae
Flavobacterium lutivivi KT215492 Flavobacteriaceae
Flavobacterium eburneum KX158678 Flavobacteriaceae
100/100
Flavobacterium squillarum JQ029111 Flavobacteriaceae
Flavobacterium fontis TYGS 6167 Flavobacteriaceae
85/100
Flavobacterium caeni TYGS 5440 Flavobacteriaceae
Flavobacterium longum AB795014 Flavobacteriaceae
62/-
Flavobacterium fulvum KU052686 Flavobacteriaceae
100/100
100/100
Flavobacterium suncheonense DQ222428 Flavobacteriaceae
86/100
Flavobacterium humicola KT750335 Flavobacteriaceae
97/100
Flavobacterium flaviflagrans KY031328 Flavobacteriaceae
100/100
Flavobacterium pedocola KU052688 Flavobacteriaceae
93/100
Flavobacterium limnosediminis TYGS 3875 Flavobacteriaceae
84/100
Flavobacterium cauense TYGS 3244 Flavobacteriaceae
81/99
Flavobacterium saliperosum TYGS 3251 Flavobacteriaceae
91/94
Flavobacterium enshiense TYGS 3837 Flavobacteriaceae
-/73
Flavobacterium lacunae LN717318 Flavobacteriaceae
Flavobacterium verecundum LN849950 Flavobacteriaceae
85/98
75/98
Flavobacterium columnare TYGS 23131 Flavobacteriaceae
Flavobacterium inkyongense KX025140 Flavobacteriaceae
-/91
96/100
Flavobacterium terrae TYGS 6198 Flavobacteriaceae
-/95
Flavobacterium vireti KM576853 Flavobacteriaceae
-/61
Flavobacterium brevivitae LN849948 Flavobacteriaceae
Flavobacterium amniphilum LT555403 Flavobacteriaceae
98/100
Flavobacterium gelidilacus AJ440996 Flavobacteriaceae
Flavobacterium ponti GQ370387 Flavobacteriaceae
97/100
Flavobacterium ahnfeltiae KC247342 Flavobacteriaceae
Flavobacterium maris AB973429 Flavobacteriaceae
97/80
Flavobacterium luticocti KU746271 Flavobacteriaceae
Flavobacterium dongtanense GU073293 Flavobacteriaceae
68/-
Flavobacterium jejuense KJ957195 Flavobacteriaceae
Flavobacterium jumunjinense JF317279 Flavobacteriaceae
Flavobacterium urocaniciphilum TYGS 4995 Flavobacteriaceae
84/75
Flavobacterium haoranii TYGS 6191 Flavobacteriaceae
Flavobacterium indicum TYGS 1049 Flavobacteriaceae
Flavobacterium filum TYGS 1445 Flavobacteriaceae
99/100
Flavobacterium qiangtangense KF726983 Flavobacteriaceae
100/100
Flavobacterium gossypii KR047732 Flavobacteriaceae
Flavobacterium endophyticum TYGS 22862 Flavobacteriaceae
Flavobacterium lindanitolerans TYGS 22857 Flavobacteriaceae
95/100
Flavobacterium ceti AM292800 Flavobacteriaceae
Flavobacterium cloacae KU746272 Flavobacteriaceae
Flavobacterium anatoliense JF825522 Flavobacteriaceae
87/77
89/100
Flavobacterium ummariense HQ329187 Flavobacteriaceae
Flavobacterium marinum JN867054 Flavobacteriaceae
Myroides guanonis HE663456 Flavobacteriaceae
100/100
Myroides indicus TYGS 23161 Flavobacteriaceae
99/100
Myroides odoratus TYGS 1065 Flavobacteriaceae
83/100
Myroides marinus GQ857652 Flavobacteriaceae
88/100
99/100
Myroides profundi EU204978 Flavobacteriaceae
-/80
62/100
Myroides odoratimimus AJ854059 Flavobacteriaceae
Myroides xuanwuensis TYGS 13752 Flavobacteriaceae
76/100
Myroides phaeus TYGS 4915 Flavobacteriaceae
Myroides injenensis HQ671078 Flavobacteriaceae
Myroides pelagicus AB176662 Flavobacteriaceae
Aequorivita capsosiphonis TYGS 1476 Flavobacteriaceae
Aequorivita aestuarii EU642844 Flavobacteriaceae
Aequorivita viscosa HM485318 Flavobacteriaceae
86/100
Aequorivita antarctica AY027802 Flavobacteriaceae
99/100
94/100
Aequorivita crocea AY027806 Flavobacteriaceae
Aequorivita lipolytica AY027805 Flavobacteriaceae
Aequorivita sublithincola TYGS 194 Flavobacteriaceae
-/98
Aequorivita nionensis KC534174 Flavobacteriaceae
88/89
67/98
61/98
Aequorivita echinoideorum KM085326 Flavobacteriaceae

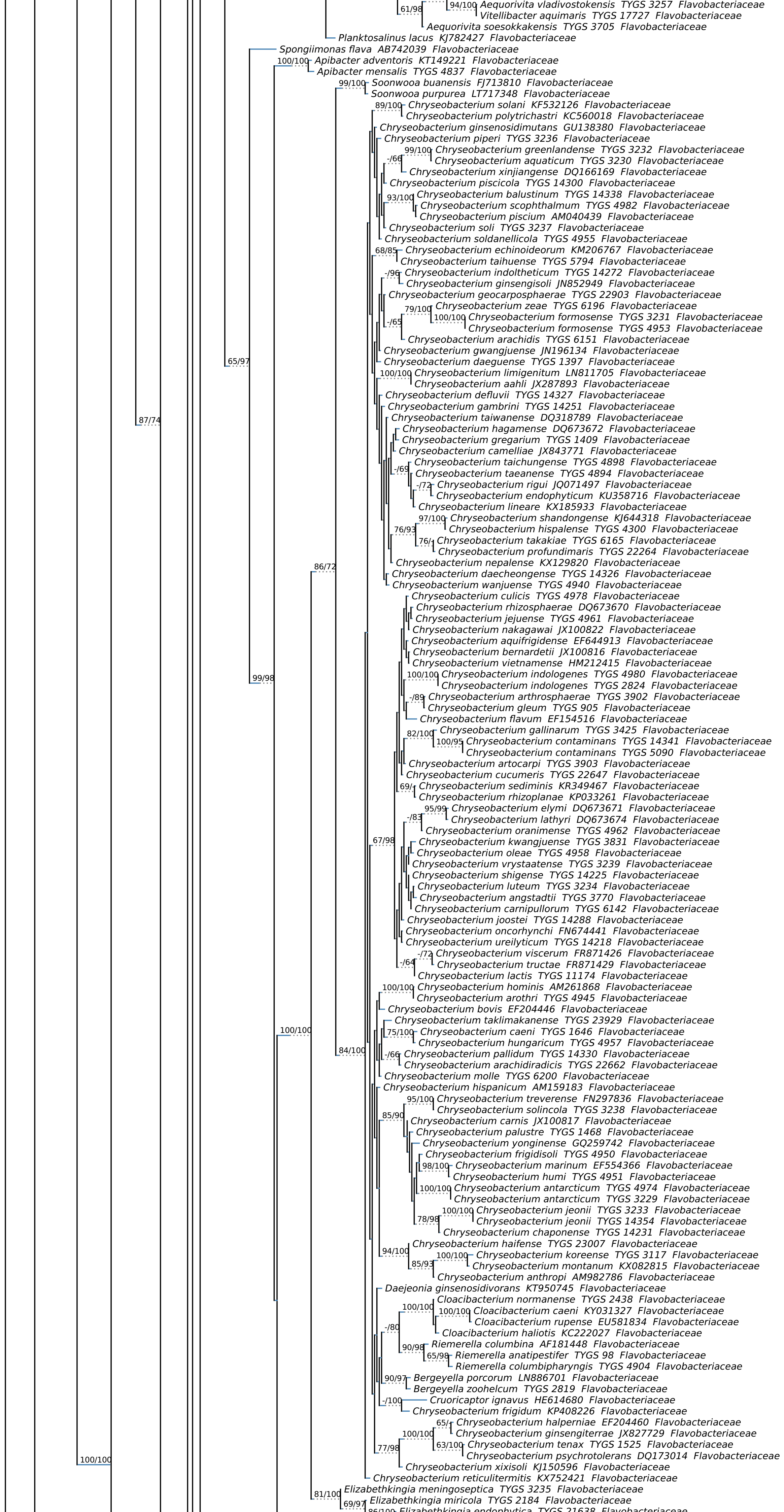

Aequorivita vladivostokensis TYGS 3257 Flavobacteriaceae
Vitellibacter aquimaris TYGS 17727 Flavobacteriaceae
Aequorivita soesokkakensis TYGS 3705 Flavobacteriaceae
Planktosalinus lacus KJ782427 Flavobacteriaceae
Spongiimonas flava AB742039 Flavobacteriaceae
Apibacter adventoris KT149221 Flavobacteriaceae
Apibacter mensalis TYGS 4837 Flavobacteriaceae
Soonwooa buanensis FJ713810 Flavobacteriaceae
Soonwooa purpurea LT717348 Flavobacteriaceae
Chryseobacterium solani KF532126 Flavobacteriaceae
Chryseobacterium polytrichastri KC560018 Flavobacteriaceae
Chryseobacterium ginsenosidimutans GU138380 Flavobacteriaceae
Chryseobacterium piperi TYGS 3236 Flavobacteriaceae
Chryseobacterium greenlandense TYGS 3232 Flavobacteriaceae
Chryseobacterium aquaticum TYGS 3230 Flavobacteriaceae
Chryseobacterium xinjiangense DQ166169 Flavobacteriaceae
Chryseobacterium piscicola TYGS 14300 Flavobacteriaceae
Chryseobacterium balustinum TYGS 14338 Flavobacteriaceae
Chryseobacterium scophthalmum TYGS 4982 Flavobacteriaceae
Chryseobacterium piscium AM040439 Flavobacteriaceae
Chryseobacterium soli TYGS 3237 Flavobacteriaceae
Chryseobacterium soldanellicola TYGS 4955 Flavobacteriaceae
Chryseobacterium echinoideorum KM206767 Flavobacteriaceae
Chryseobacterium taihuense TYGS 5794 Flavobacteriaceae
Chryseobacterium indoltheticum TYGS 14272 Flavobacteriaceae
Chryseobacterium ginsengisoli JN852949 Flavobacteriaceae
Chryseobacterium geocarposphaerae TYGS 22903 Flavobacteriaceae
Chryseobacterium zeae TYGS 6196 Flavobacteriaceae
Chryseobacterium formosense TYGS 3231 Flavobacteriaceae
Chryseobacterium formosense TYGS 4953 Flavobacteriaceae
Chryseobacterium arachidis TYGS 6151 Flavobacteriaceae
Chryseobacterium gwangjuense JN196134 Flavobacteriaceae
Chryseobacterium daeguense TYGS 1397 Flavobacteriaceae
Chryseobacterium limigenitum LN811705 Flavobacteriaceae
Chryseobacterium aahli JX287893 Flavobacteriaceae
Chryseobacterium defluvii TYGS 14327 Flavobacteriaceae
Chryseobacterium gambrini TYGS 14251 Flavobacteriaceae
Chryseobacterium taiwanense DQ318789 Flavobacteriaceae
Chryseobacterium hagamense DQ673672 Flavobacteriaceae
Chryseobacterium gregarium TYGS 1409 Flavobacteriaceae
Chryseobacterium camelliae JX843771 Flavobacteriaceae
Chryseobacterium taichungense TYGS 4898 Flavobacteriaceae
Chryseobacterium taeanense TYGS 4894 Flavobacteriaceae
Chryseobacterium rigui JQ071497 Flavobacteriaceae
Chryseobacterium endophyticum KU358716 Flavobacteriaceae
Chryseobacterium lineare KX185933 Flavobacteriaceae
Chryseobacterium shandongense KJ644318 Flavobacteriaceae
Chryseobacterium hispalense TYGS 4300 Flavobacteriaceae
Chryseobacterium takakiae TYGS 6165 Flavobacteriaceae
Chryseobacterium profundimaris TYGS 22264 Flavobacteriaceae
Chryseobacterium nepalense KX129820 Flavobacteriaceae
Chryseobacterium daecheongense TYGS 14326 Flavobacteriaceae
Chryseobacterium wanjuense TYGS 4940 Flavobacteriaceae
Chryseobacterium culicis TYGS 4978 Flavobacteriaceae
Chryseobacterium rhizosphaerae DQ673670 Flavobacteriaceae
Chryseobacterium jejuense TYGS 4961 Flavobacteriaceae
Chryseobacterium nakagawai JX100822 Flavobacteriaceae
Chryseobacterium aquifrigidense EF644913 Flavobacteriaceae
Chryseobacterium bernardetii JX100816 Flavobacteriaceae
Chryseobacterium vietnamense HM212415 Flavobacteriaceae
Chryseobacterium indologenes TYGS 4980 Flavobacteriaceae
Chryseobacterium indologenes TYGS 2824 Flavobacteriaceae
Chryseobacterium arthrosphaerae TYGS 3902 Flavobacteriaceae
Chryseobacterium gleum TYGS 905 Flavobacteriaceae
Chryseobacterium flavum EF154516 Flavobacteriaceae
Chryseobacterium gallinarum TYGS 3425 Flavobacteriaceae
Chryseobacterium contaminans TYGS 14341 Flavobacteriaceae
Chryseobacterium contaminans TYGS 5090 Flavobacteriaceae
Chryseobacterium artocarpi TYGS 3903 Flavobacteriaceae
Chryseobacterium cucumeris TYGS 22647 Flavobacteriaceae
Chryseobacterium sediminis KR349467 Flavobacteriaceae
Chryseobacterium rhizoplanae KP033261 Flavobacteriaceae
Chryseobacterium elymi DQ673671 Flavobacteriaceae
Chryseobacterium lathyri DQ673674 Flavobacteriaceae
Chryseobacterium oranimense TYGS 4962 Flavobacteriaceae
Chryseobacterium kwangjuense TYGS 3831 Flavobacteriaceae
Chryseobacterium oleae TYGS 4958 Flavobacteriaceae
Chryseobacterium vrystaatense TYGS 3239 Flavobacteriaceae
Chryseobacterium shigense TYGS 14225 Flavobacteriaceae
Chryseobacterium luteum TYGS 3234 Flavobacteriaceae
Chryseobacterium angstadtii TYGS 3770 Flavobacteriaceae
Chryseobacterium carnipullorum TYGS 6142 Flavobacteriaceae
Chryseobacterium joostei TYGS 14288 Flavobacteriaceae
Chryseobacterium oncorhynchi FN674441 Flavobacteriaceae
Chryseobacterium ureilyticum TYGS 14218 Flavobacteriaceae
Chryseobacterium viscerum FR871426 Flavobacteriaceae
Chryseobacterium tructae FR871429 Flavobacteriaceae
Chryseobacterium lactis TYGS 11174 Flavobacteriaceae
Chryseobacterium hominis AM261868 Flavobacteriaceae
Chryseobacterium arothri TYGS 4945 Flavobacteriaceae
Chryseobacterium bovis EF204446 Flavobacteriaceae
Chryseobacterium taklimakanense TYGS 23929 Flavobacteriaceae
Chryseobacterium caeni TYGS 1646 Flavobacteriaceae
Chryseobacterium hungaricum TYGS 4957 Flavobacteriaceae
Chryseobacterium pallidum TYGS 14330 Flavobacteriaceae
Chryseobacterium arachidiradicis TYGS 22662 Flavobacteriaceae
Chryseobacterium molle TYGS 6200 Flavobacteriaceae
Chryseobacterium hispanicum AM159183 Flavobacteriaceae
Chryseobacterium treverense FN297836 Flavobacteriaceae
Chryseobacterium solincola TYGS 3238 Flavobacteriaceae
Chryseobacterium carnis JX100817 Flavobacteriaceae
Chryseobacterium palustre TYGS 1468 Flavobacteriaceae
Chryseobacterium yonginense GQ259742 Flavobacteriaceae
Chryseobacterium frigidisoli TYGS 4950 Flavobacteriaceae
Chryseobacterium marinum EF554366 Flavobacteriaceae
Chryseobacterium humi TYGS 4951 Flavobacteriaceae
Chryseobacterium antarcticum TYGS 4974 Flavobacteriaceae
Chryseobacterium antarcticum TYGS 3229 Flavobacteriaceae
Chryseobacterium jeonii TYGS 3233 Flavobacteriaceae
Chryseobacterium jeonii TYGS 14354 Flavobacteriaceae
Chryseobacterium chaponense TYGS 14231 Flavobacteriaceae
Chryseobacterium haifense TYGS 23007 Flavobacteriaceae
Chryseobacterium koreense TYGS 3117 Flavobacteriaceae
Chryseobacterium montanum KX082815 Flavobacteriaceae
Chryseobacterium anthropi AM982786 Flavobacteriaceae
Daejeonia ginsenosidivorans KT950745 Flavobacteriaceae
Cloacibacterium normanense TYGS 2438 Flavobacteriaceae
Cloacibacterium caeni KY031327 Flavobacteriaceae
Cloacibacterium rupense EU581834 Flavobacteriaceae
Cloacibacterium haliotis KC222027 Flavobacteriaceae
Riemerella columbina AF181448 Flavobacteriaceae
Riemerella anatipestifer TYGS 98 Flavobacteriaceae
Riemerella columbipharyngis TYGS 4904 Flavobacteriaceae
Bergeyella porcorum LN886701 Flavobacteriaceae
Bergeyella zoohelcum TYGS 2819 Flavobacteriaceae
Cruoricaptor ignavus HE614680 Flavobacteriaceae
Chryseobacterium frigidum KP408226 Flavobacteriaceae
Chryseobacterium halperniae EF204460 Flavobacteriaceae
Chryseobacterium ginsengiterrae JX827729 Flavobacteriaceae
Chryseobacterium tenax TYGS 1525 Flavobacteriaceae
Chryseobacterium psychrotolerans DQ173014 Flavobacteriaceae
Chryseobacterium xixisoli KJ150596 Flavobacteriaceae
Chryseobacterium reticulitermitis KX752421 Flavobacteriaceae
Elizabethkingia meningoseptica TYGS 3235 Flavobacteriaceae
Elizabethkingia miricola TYGS 2184 Flavobacteriaceae
Elizabethkingia endophytica TYGS 21638 Flavobacteriaceae

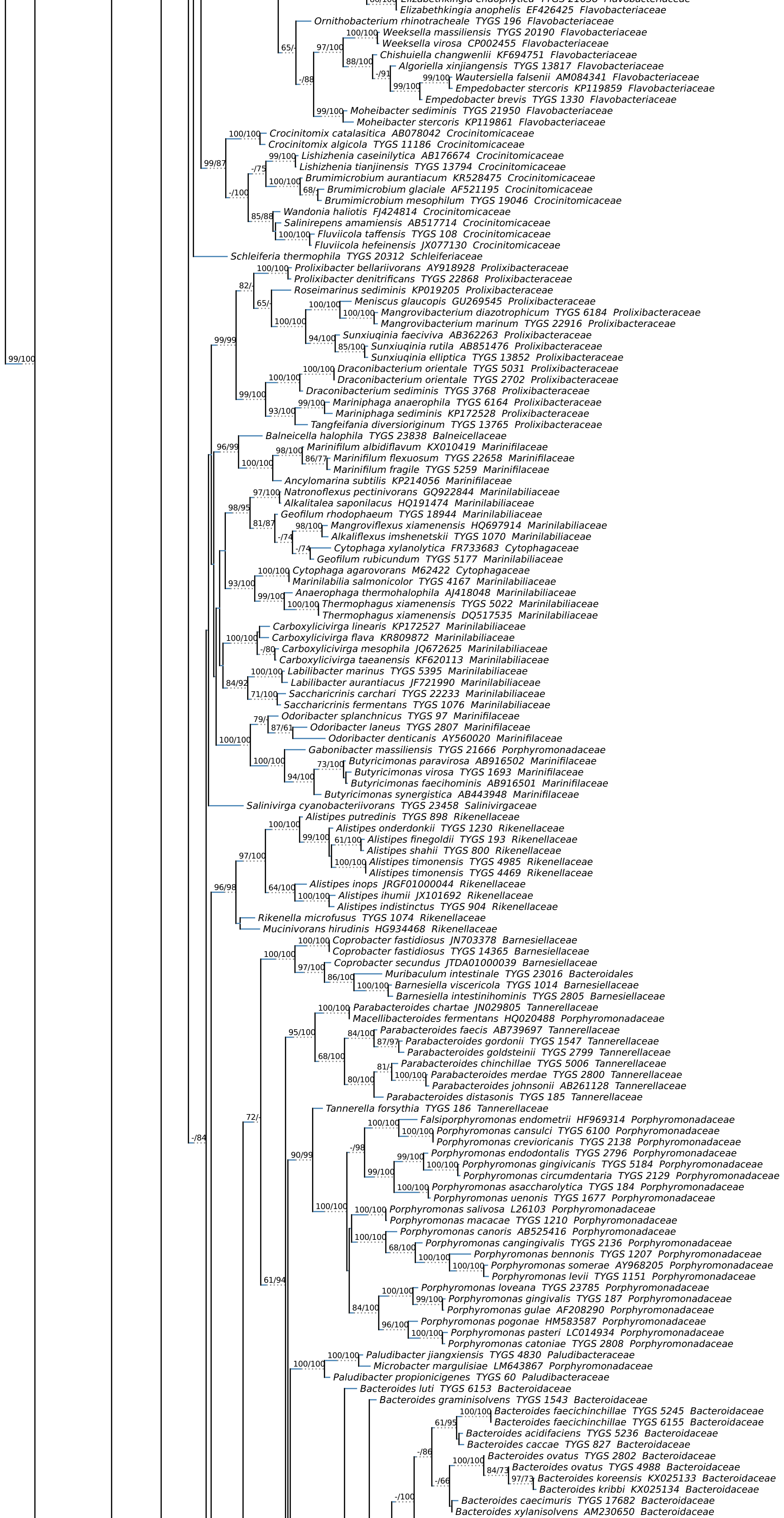
Elizabethkingia anophelis EF426425 Flavobacteriaceae
Ornithobacterium rhinotracheale TYGS 196 Flavobacteriaceae
Weeksella massiliensis TYGS 20190 Flavobacteriaceae
Weeksella virosa CP002455 Flavobacteriaceae
Chishuiella changwenlii KF694751 Flavobacteriaceae
Algoriella xinjiangensis TYGS 13817 Flavobacteriaceae
Wautersiella falsenii AM084341 Flavobacteriaceae
Empedobacter stercoris KP119859 Flavobacteriaceae
Empedobacter brevis TYGS 1330 Flavobacteriaceae
Moheibacter sediminis TYGS 21950 Flavobacteriaceae
Moheibacter stercoris KP119861 Flavobacteriaceae
Crocinitomix catalasitica AB078042 Crocinitomicaceae
Crocinitomix algicola TYGS 11186 Crocinitomicaceae
Lishizhenia caseinilytica AB176674 Crocinitomicaceae
Lishizhenia tianjinensis TYGS 13794 Crocinitomicaceae
Brumimicrobium aurantiacum KR528475 Crocinitomicaceae
Brumimicrobium glaciale AF521195 Crocinitomicaceae
Brumimicrobium mesophilum TYGS 19046 Crocinitomicaceae
Wandonia haliotis FJ424814 Crocinitomicaceae
Salinirepens amamiensis AB517714 Crocinitomicaceae
Fluviicola taffensis TYGS 108 Crocinitomicaceae
Fluviicola hefeinensis JX077130 Crocinitomicaceae
Schleiferia thermophila TYGS 20312 Schleiferiaceae
Prolixibacter bellariivorans AY918928 Prolixibacteraceae
Prolixibacter denitrificans TYGS 22868 Prolixibacteraceae
Roseimarinus sediminis KP019205 Prolixibacteraceae
Meniscus glaucopis GU269545 Prolixibacteraceae
Mangrovibacterium diazotrophicum TYGS 6184 Prolixibacteraceae
Mangrovibacterium marinum TYGS 22916 Prolixibacteraceae
Sunxiuqinia faeciviva AB362263 Prolixibacteraceae
Sunxiuqinia rutila AB851476 Prolixibacteraceae
Sunxiuqinia elliptica TYGS 13852 Prolixibacteraceae
Draconibacterium orientale TYGS 5031 Prolixibacteraceae
Draconibacterium orientale TYGS 2702 Prolixibacteraceae
Draconibacterium sediminis TYGS 3768 Prolixibacteraceae
Mariniphaga anaerophila TYGS 6164 Prolixibacteraceae
Mariniphaga sediminis KP172528 Prolixibacteraceae
Tangfeifania diversioriginum TYGS 13765 Prolixibacteraceae
Balneicella halophila TYGS 23838 Balneicellaceae
Marinifilum albidiflavum KX010419 Marinifilaceae
Marinifilum flexuosum TYGS 22658 Marinifilaceae
Marinifilum fragile TYGS 5259 Marinifilaceae
Ancylomarina subtilis KP214056 Marinifilaceae
Natronoflexus pectinivorans GQ922844 Marinilabiliaceae
Alkalitalea saponilacus HQ191474 Marinilabiliaceae
Geofilum rhodophaeum TYGS 18944 Marinilabiliaceae
Mangroviflexus xiamenensis HQ697914 Marinilabiliaceae
Alkaliflexus imshenetskii TYGS 1070 Marinilabiliaceae
Cytophaga xylanolytica FR733683 Cytophagaceae
Geofilum rubicundum TYGS 5177 Marinilabiliaceae
Cytophaga agarovorans M62422 Cytophagaceae
Marinilabilia salmonicolor TYGS 4167 Marinilabiliaceae
Anaerophaga thermohalophila AJ418048 Marinilabiliaceae
Thermophagus xiamenensis TYGS 5022 Marinilabiliaceae
Thermophagus xiamenensis DQ517535 Marinilabiliaceae
Carboxylicivirga linearis KP172527 Marinilabiliaceae
Carboxylicivirga flava KR809872 Marinilabiliaceae
Carboxylicivirga mesophila JQ672625 Marinilabiliaceae
Carboxylicivirga taeanensis KF620113 Marinilabiliaceae
Labilibacter marinus TYGS 5395 Marinilabiliaceae
Labilibacter aurantiacus JF721990 Marinilabiliaceae
Saccharicrinis carchari TYGS 22233 Marinilabiliaceae
Saccharicrinis fermentans TYGS 1076 Marinilabiliaceae
Odoribacter splanchnicus TYGS 97 Marinifilaceae
Odoribacter laneus TYGS 2807 Marinifilaceae
Odoribacter denticanis AY560020 Marinifilaceae
Gabonibacter massiliensis TYGS 21666 Porphyromonadaceae
Butyricimonas paravirosa AB916502 Marinifilaceae
Butyricimonas virosa TYGS 1693 Marinifilaceae
Butyricimonas faecihominis AB916501 Marinifilaceae
Butyricimonas synergistica AB443948 Marinifilaceae
Salinivirga cyanobacteriivorans TYGS 23458 Salinivirgaceae
Alistipes putredinis TYGS 898 Rikenellaceae
Alistipes onderdonkii TYGS 1230 Rikenellaceae
Alistipes finegoldii TYGS 193 Rikenellaceae
Alistipes shahii TYGS 800 Rikenellaceae
Alistipes timonensis TYGS 4985 Rikenellaceae
Alistipes timonensis TYGS 4469 Rikenellaceae
Alistipes inops JRGF01000044 Rikenellaceae
Alistipes ihumii JX101692 Rikenellaceae
Alistipes indistinctus TYGS 904 Rikenellaceae
Rikenella microfusus TYGS 1074 Rikenellaceae
Mucinivorans hirudinis HG934468 Rikenellaceae
Coprobacter fastidiosus JN703378 Barnesiellaceae
Coprobacter fastidiosus TYGS 14365 Barnesiellaceae
Coprobacter secundus JTDA01000039 Barnesiellaceae
Muribaculum intestinale TYGS 23016 Bacteroidales
Barnesiella viscericola TYGS 1014 Barnesiellaceae
Barnesiella intestinihominis TYGS 2805 Barnesiellaceae
Parabacteroides chartae JN029805 Tannerellaceae
Macellibacteroides fermentans HQ020488 Porphyromonadaceae
Parabacteroides faecis AB739697 Tannerellaceae
Parabacteroides gordonii TYGS 1547 Tannerellaceae
Parabacteroides goldsteinii TYGS 2799 Tannerellaceae
Parabacteroides chinchillae TYGS 5006 Tannerellaceae
Parabacteroides merdae TYGS 2800 Tannerellaceae
Parabacteroides johnsonii AB261128 Tannerellaceae
Parabacteroides distasonis TYGS 185 Tannerellaceae
Tannerella forsythia TYGS 186 Tannerellaceae
Falsiporphyromonas endometrii HF969314 Porphyromonadaceae
Porphyromonas cansulci TYGS 6100 Porphyromonadaceae
Porphyromonas creviorcanis TYGS 2138 Porphyromonadaceae
Porphyromonas endodontalis TYGS 2796 Porphyromonadaceae
Porphyromonas gingivicanis TYGS 5184 Porphyromonadaceae
Porphyromonas circumdentaria TYGS 2129 Porphyromonadaceae
Porphyromonas asaccharolytica TYGS 184 Porphyromonadaceae
Porphyromonas uenonis TYGS 1677 Porphyromonadaceae
Porphyromonas salivosa L26103 Porphyromonadaceae
Porphyromonas macacae TYGS 1210 Porphyromonadaceae
Porphyromonas canoris AB525416 Porphyromonadaceae
Porphyromonas cangingivalis TYGS 2136 Porphyromonadaceae
Porphyromonas bennonis TYGS 1207 Porphyromonadaceae
Porphyromonas somerae AY968205 Porphyromonadaceae
Porphyromonas levii TYGS 1151 Porphyromonadaceae
Porphyromonas loveana TYGS 23785 Porphyromonadaceae
Porphyromonas gingivalis TYGS 187 Porphyromonadaceae
Porphyromonas gulae AF208290 Porphyromonadaceae
Porphyromonas pogonae HM583587 Porphyromonadaceae
Porphyromonas pasteri LC014934 Porphyromonadaceae
Porphyromonas catoniae TYGS 2808 Porphyromonadaceae
Paludibacter jiangxiensis TYGS 4830 Paludibacteraceae
Microbacter margulisiae LM643867 Porphyromonadaceae
Paludibacter propionicigenes TYGS 60 Paludibacteraceae
Bacteroides luti TYGS 6153 Bacteroidaceae
Bacteroides graminisolvens TYGS 1543 Bacteroidaceae
Bacteroides faecichinchillae TYGS 5245 Bacteroidaceae
Bacteroides faecichinchillae TYGS 6155 Bacteroidaceae
Bacteroides acidifaciens TYGS 5236 Bacteroidaceae
Bacteroides caccae TYGS 827 Bacteroidaceae
Bacteroides ovatus TYGS 2802 Bacteroidaceae
Bacteroides ovatus TYGS 4988 Bacteroidaceae
Bacteroides koreensis KX025133 Bacteroidaceae
Bacteroides kribbi KX025134 Bacteroidaceae
Bacteroides caecimuris TYGS 17682 Bacteroidaceae
Bacteroides xylanisolvens AM230650 Bacteroidaceae

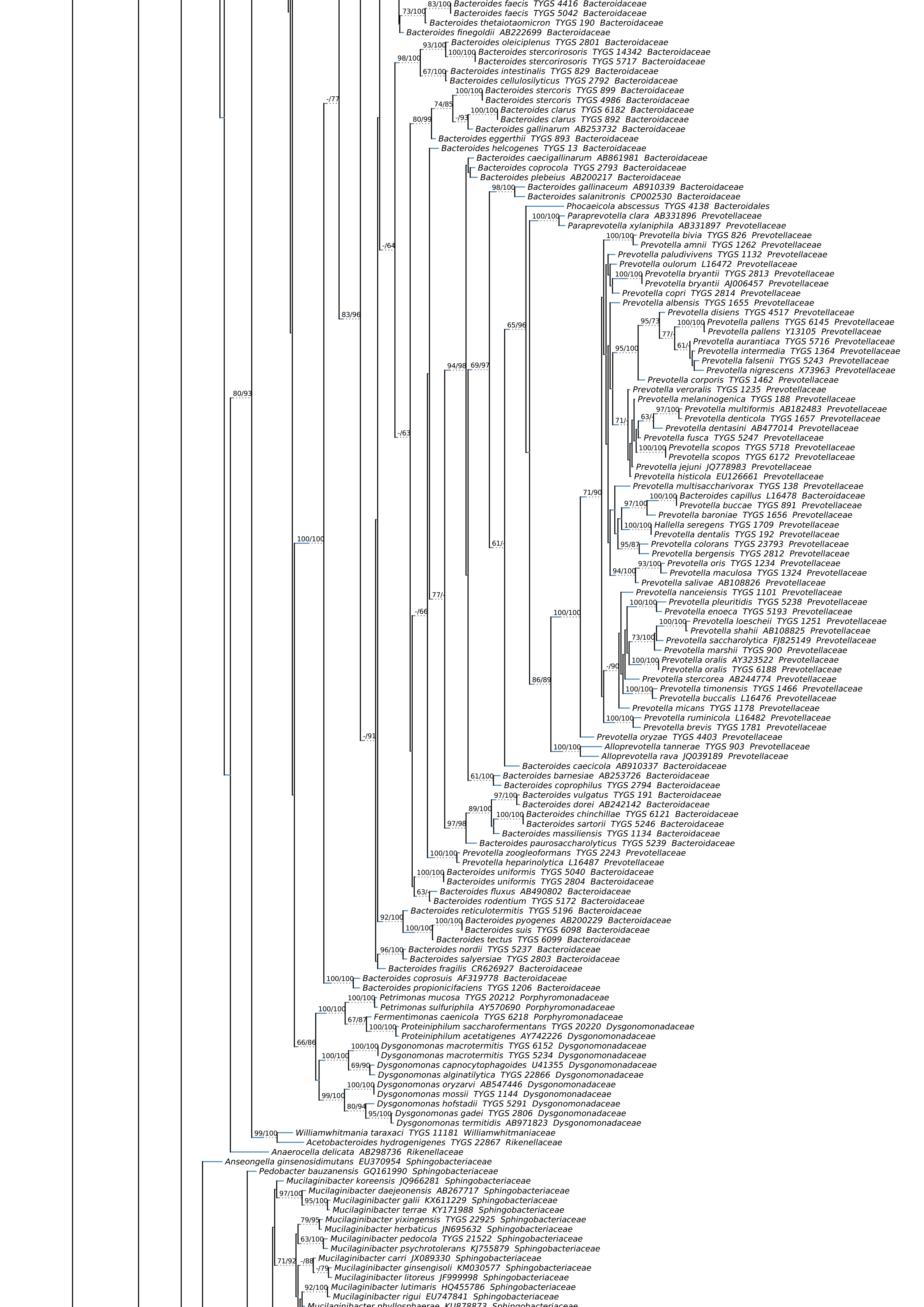
83/100 Bacteroides faecis TYGS 4416 Bacteroidaceae
73/100 Bacteroides faecis TYGS 5042 Bacteroidaceae
Bacteroides thetaiotaomicron TYGS 190 Bacteroidaceae
Bacteroides finegoldii AB222699 Bacteroidaceae
93/100 Bacteroides oleiciplenus TYGS 2801 Bacteroidaceae
100/100 Bacteroides stercorirosoris TYGS 14342 Bacteroidaceae
98/100 Bacteroides stercorirosoris TYGS 5717 Bacteroidaceae
67/100 Bacteroides intestinalis TYGS 829 Bacteroidaceae
Bacteroides cellulosilyticus TYGS 2792 Bacteroidaceae
100/100 Bacteroides stercoris TYGS 899 Bacteroidaceae
-/77
74/85 Bacteroides stercoris TYGS 4986 Bacteroidaceae
100/100 Bacteroides clarus TYGS 6182 Bacteroidaceae
80/99 -/93 Bacteroides clarus TYGS 892 Bacteroidaceae
Bacteroides gallinarum AB253732 Bacteroidaceae
Bacteroides eggerthii TYGS 893 Bacteroidaceae
Bacteroides helcogenes TYGS 13 Bacteroidaceae
Bacteroides caecigallinarum AB861981 Bacteroidaceae
Bacteroides coprocola TYGS 2793 Bacteroidaceae
Bacteroides plebeius AB200217 Bacteroidaceae
98/100 Bacteroides gallinaceum AB910339 Bacteroidaceae
Bacteroides salanitronis CP002530 Bacteroidaceae
Phocaeicola abscessus TYGS 4138 Bacteroidales
100/100 Paraprevotella clara AB331896 Prevotellaceae
Paraprevotella xylaniphila AB331897 Prevotellaceae
100/100 Prevotella bivia TYGS 826 Prevotellaceae
-/64 Prevotella amnii TYGS 1262 Prevotellaceae
Prevotella paludivivens TYGS 1132 Prevotellaceae
Prevotella oulorum L16472 Prevotellaceae
100/100 Prevotella bryantii TYGS 2813 Prevotellaceae
Prevotella bryantii AJ006457 Prevotellaceae
Prevotella copri TYGS 2814 Prevotellaceae
Prevotella albensis TYGS 1655 Prevotellaceae
83/96 Prevotella disiens TYGS 4517 Prevotellaceae
95/73 100/100 Prevotella pallens TYGS 6145 Prevotellaceae
65/96 77/- Prevotella pallens Y13105 Prevotellaceae
61/- Prevotella aurantiaca TYGS 5716 Prevotellaceae
95/100 Prevotella intermedia TYGS 1364 Prevotellaceae
Prevotella falsenii TYGS 5243 Prevotellaceae
94/98 69/97 Prevotella nigrescens X73963 Prevotellaceae
Prevotella corporis TYGS 1462 Prevotellaceae
80/93 Prevotella veroralis TYGS 1235 Prevotellaceae
Prevotella melaninogenica TYGS 188 Prevotellaceae
97/100 Prevotella multiformis AB182483 Prevotellaceae
63/- Prevotella denticola TYGS 1657 Prevotellaceae
71/- Prevotella dentasini AB477014 Prevotellaceae
-/63 Prevotella fusca TYGS 5247 Prevotellaceae
100/100 Prevotella scopos TYGS 5718 Prevotellaceae
Prevotella scopos TYGS 6172 Prevotellaceae
Prevotella jejuni JQ778983 Prevotellaceae
Prevotella histicola EU126661 Prevotellaceae
71/90 Prevotella multisaccharivorax TYGS 138 Prevotellaceae
100/100 Bacteroides capillus L16478 Bacteroidaceae
97/100 Prevotella buccae TYGS 891 Prevotellaceae
Prevotella baroniae TYGS 1656 Prevotellaceae
100/100 100/100 Hallella seregens TYGS 1709 Prevotellaceae
Prevotella dentalis TYGS 192 Prevotellaceae
61/- 95/87 Prevotella colorans TYGS 23793 Prevotellaceae
Prevotella bergensis TYGS 2812 Prevotellaceae
93/100 Prevotella oris TYGS 1234 Prevotellaceae
94/100 Prevotella maculosa TYGS 1324 Prevotellaceae
Prevotella salivae AB108826 Prevotellaceae
77/- Prevotella nanceiensis TYGS 1101 Prevotellaceae
100/100 Prevotella pleuritidis TYGS 5238 Prevotellaceae
-/66 100/100 Prevotella enoeca TYGS 5193 Prevotellaceae
100/100 Prevotella loescheii TYGS 1251 Prevotellaceae
Prevotella shahii AB108825 Prevotellaceae
73/100 Prevotella saccharolytica FJ825149 Prevotellaceae
Prevotella marshii TYGS 900 Prevotellaceae
100/100 Prevotella oralis AY323522 Prevotellaceae
-/90 Prevotella oralis TYGS 6188 Prevotellaceae
86/89 Prevotella stercorea AB244774 Prevotellaceae
100/100 Prevotella timonensis TYGS 1466 Prevotellaceae
Prevotella buccalis L16476 Prevotellaceae
Prevotella micans TYGS 1178 Prevotellaceae
100/100 Prevotella ruminicola L16482 Prevotellaceae
Prevotella brevis TYGS 1781 Prevotellaceae
-/91 Prevotella oryzae TYGS 4403 Prevotellaceae
100/100 Alloprevotella tannerae TYGS 903 Prevotellaceae
Alloprevotella rava JQ039189 Prevotellaceae
Bacteroides caecicola AB910337 Bacteroidaceae
61/100 Bacteroides barnesiae AB253726 Bacteroidaceae
Bacteroides coprophilus TYGS 2794 Bacteroidaceae
97/100 Bacteroides vulgatus TYGS 191 Bacteroidaceae
89/100 Bacteroides dorei AB242142 Bacteroidaceae
100/100 Bacteroides chinchillae TYGS 6121 Bacteroidaceae
97/98 Bacteroides sartorii TYGS 5246 Bacteroidaceae
Bacteroides massiliensis TYGS 1134 Bacteroidaceae
Bacteroides paurosaccharolyticus TYGS 5239 Bacteroidaceae
100/100 Prevotella zoogleoformans TYGS 2243 Prevotellaceae
Prevotella heparinolytica L16487 Prevotellaceae
100/100 Bacteroides uniformis TYGS 5040 Bacteroidaceae
Bacteroides uniformis TYGS 2804 Bacteroidaceae
63/- Bacteroides fluxus AB490802 Bacteroidaceae
Bacteroides rodentium TYGS 5172 Bacteroidaceae
Bacteroides reticulotermitis TYGS 5196 Bacteroidaceae
92/100 100/100 Bacteroides pyogenes AB200229 Bacteroidaceae
100/100 Bacteroides suis TYGS 6098 Bacteroidaceae
Bacteroides tectus TYGS 6099 Bacteroidaceae
96/100 Bacteroides nordii TYGS 5237 Bacteroidaceae
Bacteroides salyersiae TYGS 2803 Bacteroidaceae
Bacteroides fragilis CR626927 Bacteroidaceae
100/100 Bacteroides coprosuis AF319778 Bacteroidaceae
Bacteroides propionicifaciens TYGS 1206 Bacteroidaceae
100/100 Petrimonas mucosa TYGS 20212 Porphyromonadaceae
100/100 Petrimonas sulfuriphila AY570690 Porphyromonadaceae
67/87 Fermentimonas caenicola TYGS 6218 Porphyromonadaceae
100/100 Proteiniphilum saccharofermentans TYGS 20220 Dysgonomonadaceae
Proteiniphilum acetatigenes AY742226 Dysgonomonadaceae
66/86 100/100 Dysgonomonas macrotermitis TYGS 6152 Dysgonomonadaceae
100/100 Dysgonomonas macrotermitis TYGS 5234 Dysgonomonadaceae
69/90 Dysgonomonas capnocytophagoides U41355 Dysgonomonadaceae
Dysgonomonas alginatilytica TYGS 22866 Dysgonomonadaceae
100/100 Dysgonomonas oryzarvi AB547446 Dysgonomonadaceae
99/100 Dysgonomonas mossii TYGS 1144 Dysgonomonadaceae
80/94 Dysgonomonas hofstadii TYGS 5291 Dysgonomonadaceae
95/100 Dysgonomonas gadei TYGS 2806 Dysgonomonadaceae
Dysgonomonas termitidis AB971823 Dysgonomonadaceae
99/100 Williamwhitmania taraxaci TYGS 11181 Williamwhitmaniaceae
Acetobacteroides hydrogenigenes TYGS 22867 Rikenellaceae
Anaerocella delicata AB298736 Rikenellaceae
Anseongella ginsenosidimutans EU370954 Sphingobacteriaceae
Pedobacter bauzanensis GQ161990 Sphingobacteriaceae
Mucilaginibacter koreensis JQ966281 Sphingobacteriaceae
97/100 Mucilaginibacter daejeonensis AB267717 Sphingobacteriaceae
95/100 Mucilaginibacter galii KX611229 Sphingobacteriaceae
Mucilaginibacter terrae KY171988 Sphingobacteriaceae
79/95 Mucilaginibacter yixingensis TYGS 22925 Sphingobacteriaceae
Mucilaginibacter herbaticus JN695632 Sphingobacteriaceae
63/100 Mucilaginibacter pedocola TYGS 21522 Sphingobacteriaceae
Mucilaginibacter psychrotolerans KJ755879 Sphingobacteriaceae
71/92 -/88 Mucilaginibacter carri JX089330 Sphingobacteriaceae
-/79 Mucilaginibacter ginsengisoli KM030577 Sphingobacteriaceae
Mucilaginibacter litoreus JF999998 Sphingobacteriaceae
92/100 Mucilaginibacter lutimaris HQ455786 Sphingobacteriaceae
Mucilaginibacter rigui EU747841 Sphingobacteriaceae

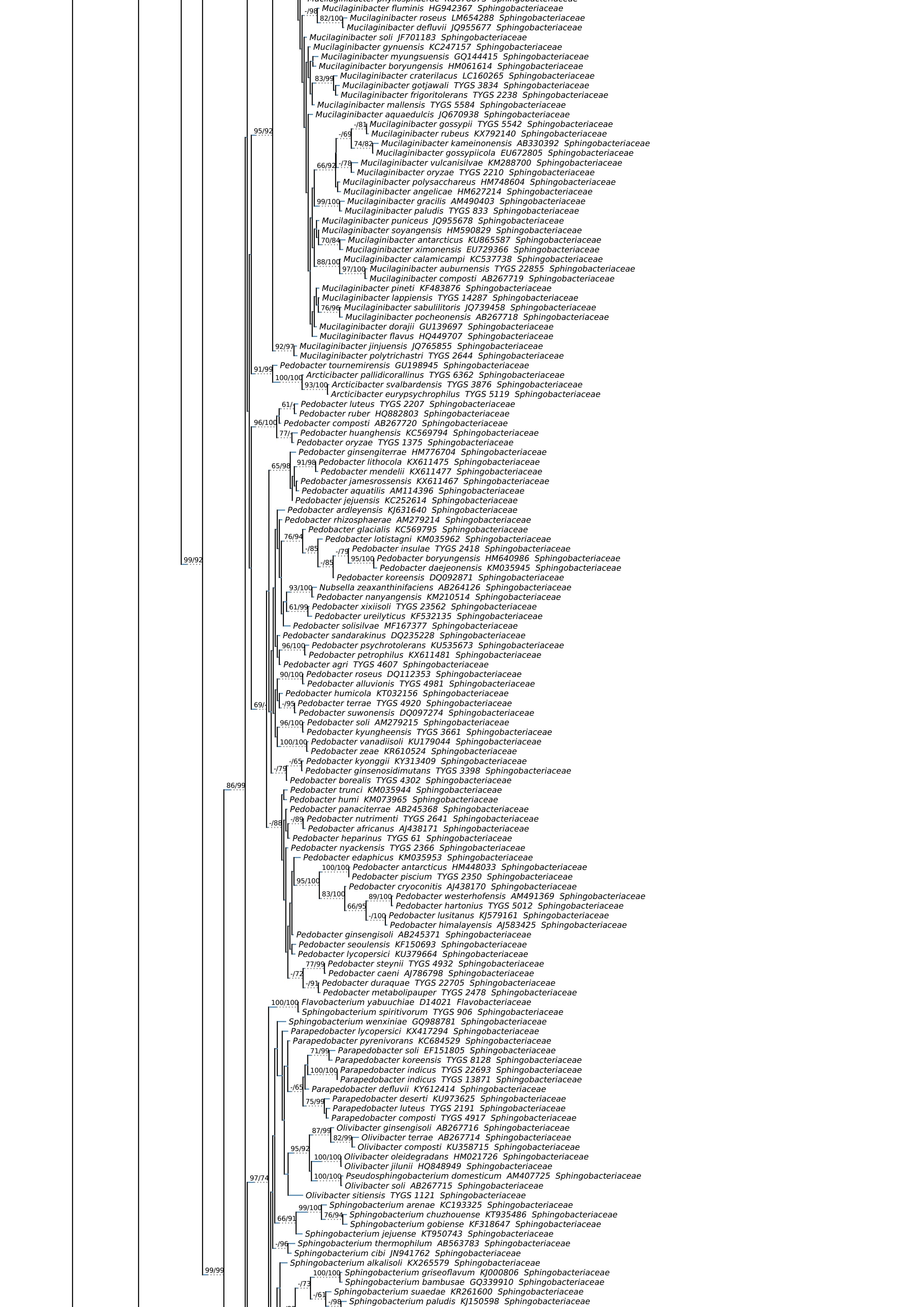
Mucilaginibacter fluminis HG942367 Sphingobacteriaceae
Mucilaginibacter roseus LM654288 Sphingobacteriaceae
Mucilaginibacter defluvii JQ955677 Sphingobacteriaceae
Mucilaginibacter soli JF701183 Sphingobacteriaceae
Mucilaginibacter gynuensis KC247157 Sphingobacteriaceae
Mucilaginibacter myungsuensis GQ144415 Sphingobacteriaceae
Mucilaginibacter boryungensis HM061614 Sphingobacteriaceae
Mucilaginibacter craterilacus LC160265 Sphingobacteriaceae
Mucilaginibacter gotjawali TYGS 3834 Sphingobacteriaceae
Mucilaginibacter frigoritolerans TYGS 2238 Sphingobacteriaceae
Mucilaginibacter mallensis TYGS 5584 Sphingobacteriaceae
Mucilaginibacter aquaedulcis JQ670938 Sphingobacteriaceae
Mucilaginibacter gossypii TYGS 5542 Sphingobacteriaceae
Mucilaginibacter rubeus KX792140 Sphingobacteriaceae
Mucilaginibacter kameinonensis AB330392 Sphingobacteriaceae
Mucilaginibacter gossypiicola EU672805 Sphingobacteriaceae
Mucilaginibacter vulcanisilvae KM288700 Sphingobacteriaceae
Mucilaginibacter oryzae TYGS 2210 Sphingobacteriaceae
Mucilaginibacter polysacchareus HM748604 Sphingobacteriaceae
Mucilaginibacter angelicae HM627214 Sphingobacteriaceae
Mucilaginibacter gracilis AM490403 Sphingobacteriaceae
Mucilaginibacter paludis TYGS 833 Sphingobacteriaceae
Mucilaginibacter puniceus JQ955678 Sphingobacteriaceae
Mucilaginibacter soyangensis HM590829 Sphingobacteriaceae
Mucilaginibacter antarcticus KU865587 Sphingobacteriaceae
Mucilaginibacter ximonensis EU729366 Sphingobacteriaceae
Mucilaginibacter calamicampi KC537738 Sphingobacteriaceae
Mucilaginibacter auburnensis TYGS 22855 Sphingobacteriaceae
Mucilaginibacter composti AB267719 Sphingobacteriaceae
Mucilaginibacter pineti KF483876 Sphingobacteriaceae
Mucilaginibacter lappiensis TYGS 14287 Sphingobacteriaceae
Mucilaginibacter sabulilitoris JQ739458 Sphingobacteriaceae
Mucilaginibacter pocheonensis AB267718 Sphingobacteriaceae
Mucilaginibacter dorajii GU139697 Sphingobacteriaceae
Mucilaginibacter flavus HQ449707 Sphingobacteriaceae
Mucilaginibacter jinjuensis JQ765855 Sphingobacteriaceae
Mucilaginibacter polytrichastri TYGS 2644 Sphingobacteriaceae
Pedobacter tournemirensis GU198945 Sphingobacteriaceae
Arcticibacter pallidicorallinus TYGS 6362 Sphingobacteriaceae
Arcticibacter svalbardensis TYGS 3876 Sphingobacteriaceae
Arcticibacter eurypsychrophilus TYGS 5119 Sphingobacteriaceae
Pedobacter luteus TYGS 2207 Sphingobacteriaceae
Pedobacter ruber HQ882803 Sphingobacteriaceae
Pedobacter composti AB267720 Sphingobacteriaceae
Pedobacter huanghensis KC569794 Sphingobacteriaceae
Pedobacter oryzae TYGS 1375 Sphingobacteriaceae
Pedobacter ginsengiterrae HM776704 Sphingobacteriaceae
Pedobacter lithocola KX611475 Sphingobacteriaceae
Pedobacter mendelii KX611477 Sphingobacteriaceae
Pedobacter jamesrossensis KX611467 Sphingobacteriaceae
Pedobacter aquatilis AM114396 Sphingobacteriaceae
Pedobacter jejuensis KC252614 Sphingobacteriaceae
Pedobacter ardleyensis KJ631640 Sphingobacteriaceae
Pedobacter rhizosphaerae AM279214 Sphingobacteriaceae
Pedobacter glacialis KC569795 Sphingobacteriaceae
Pedobacter lotistagni KM035962 Sphingobacteriaceae
Pedobacter insulae TYGS 2418 Sphingobacteriaceae
Pedobacter boryungensis HM640986 Sphingobacteriaceae
Pedobacter daejeonensis KM035945 Sphingobacteriaceae
Pedobacter koreensis DQ092871 Sphingobacteriaceae
Nubsella zeaxanthinifaciens AB264126 Sphingobacteriaceae
Pedobacter nanyangensis KM210514 Sphingobacteriaceae
Pedobacter xixiisoli TYGS 23562 Sphingobacteriaceae
Pedobacter ureilyticus KF532135 Sphingobacteriaceae
Pedobacter solisilvae MF167377 Sphingobacteriaceae
Pedobacter sandarakinus DQ235228 Sphingobacteriaceae
Pedobacter psychrotolerans KU535673 Sphingobacteriaceae
Pedobacter petrophilus KX611481 Sphingobacteriaceae
Pedobacter agri TYGS 4607 Sphingobacteriaceae
Pedobacter roseus DQ112353 Sphingobacteriaceae
Pedobacter alluvionis TYGS 4981 Sphingobacteriaceae
Pedobacter humicola KT032156 Sphingobacteriaceae
Pedobacter terrae TYGS 4920 Sphingobacteriaceae
Pedobacter suwonensis DQ097274 Sphingobacteriaceae
Pedobacter soli AM279215 Sphingobacteriaceae
Pedobacter kyungheensis TYGS 3661 Sphingobacteriaceae
Pedobacter vanadiisoli KU179044 Sphingobacteriaceae
Pedobacter zeae KR610524 Sphingobacteriaceae
Pedobacter kyonggii KY313409 Sphingobacteriaceae
Pedobacter ginsenosidimutans TYGS 3398 Sphingobacteriaceae
Pedobacter borealis TYGS 4302 Sphingobacteriaceae
Pedobacter trunci KM035944 Sphingobacteriaceae
Pedobacter humi KM073965 Sphingobacteriaceae
Pedobacter panaciterrae AB245368 Sphingobacteriaceae
Pedobacter nutrimenti TYGS 2641 Sphingobacteriaceae
Pedobacter africanus AJ438171 Sphingobacteriaceae
Pedobacter heparinus TYGS 61 Sphingobacteriaceae
Pedobacter nyackensis TYGS 2366 Sphingobacteriaceae
Pedobacter edaphicus KM035953 Sphingobacteriaceae
Pedobacter antarcticus HM448033 Sphingobacteriaceae
Pedobacter piscium TYGS 2350 Sphingobacteriaceae
Pedobacter cryoconitis AJ438170 Sphingobacteriaceae
Pedobacter westerhofensis AM491369 Sphingobacteriaceae
Pedobacter hartonius TYGS 5012 Sphingobacteriaceae
Pedobacter lusitanus KJ579161 Sphingobacteriaceae
Pedobacter himalayensis AJ583425 Sphingobacteriaceae
Pedobacter ginsengisoli AB245371 Sphingobacteriaceae
Pedobacter seoulensis KF150693 Sphingobacteriaceae
Pedobacter lycopersici KU379664 Sphingobacteriaceae
Pedobacter steynii TYGS 4932 Sphingobacteriaceae
Pedobacter caeni AJ786798 Sphingobacteriaceae
Pedobacter duraquae TYGS 22705 Sphingobacteriaceae
Pedobacter metabolipauper TYGS 2478 Sphingobacteriaceae
Flavobacterium yabuuchiae D14021 Flavobacteriaceae
Sphingobacterium spiritivorum TYGS 906 Sphingobacteriaceae
Sphingobacterium wenxiniae GQ988781 Sphingobacteriaceae
Parapedobacter lycopersici KX417294 Sphingobacteriaceae
Parapedobacter pyrenivorans KC684529 Sphingobacteriaceae
Parapedobacter soli EF151805 Sphingobacteriaceae
Parapedobacter koreensis TYGS 8128 Sphingobacteriaceae
Parapedobacter indicus TYGS 22693 Sphingobacteriaceae
Parapedobacter indicus TYGS 13871 Sphingobacteriaceae
Parapedobacter defluvii KY612414 Sphingobacteriaceae
Parapedobacter deserti KU973625 Sphingobacteriaceae
Parapedobacter luteus TYGS 2191 Sphingobacteriaceae
Parapedobacter composti TYGS 4917 Sphingobacteriaceae
Olivibacter ginsengisoli AB267716 Sphingobacteriaceae
Olivibacter terrae AB267714 Sphingobacteriaceae
Olivibacter composti KU358715 Sphingobacteriaceae
Olivibacter oleidegradans HM021726 Sphingobacteriaceae
Olivibacter jilunii HQ848949 Sphingobacteriaceae
Pseudosphingobacterium domesticum AM407725 Sphingobacteriaceae
Olivibacter soli AB267715 Sphingobacteriaceae
Olivibacter sitiensis TYGS 1121 Sphingobacteriaceae
Sphingobacterium arenae KC193325 Sphingobacteriaceae
Sphingobacterium chuzhouense KT935486 Sphingobacteriaceae
Sphingobacterium gobiense KF318647 Sphingobacteriaceae
Sphingobacterium jejuense KT950743 Sphingobacteriaceae
Sphingobacterium thermophilum AB563783 Sphingobacteriaceae
Sphingobacterium cibi JN941762 Sphingobacteriaceae
Sphingobacterium alkalisoli KX265579 Sphingobacteriaceae
Sphingobacterium griseoflavum KJ000806 Sphingobacteriaceae
Sphingobacterium bambusae GQ339910 Sphingobacteriaceae
Sphingobacterium suaedae KR261600 Sphingobacteriaceae
Sphingobacterium paludis KJ150598 Sphingobacteriaceae

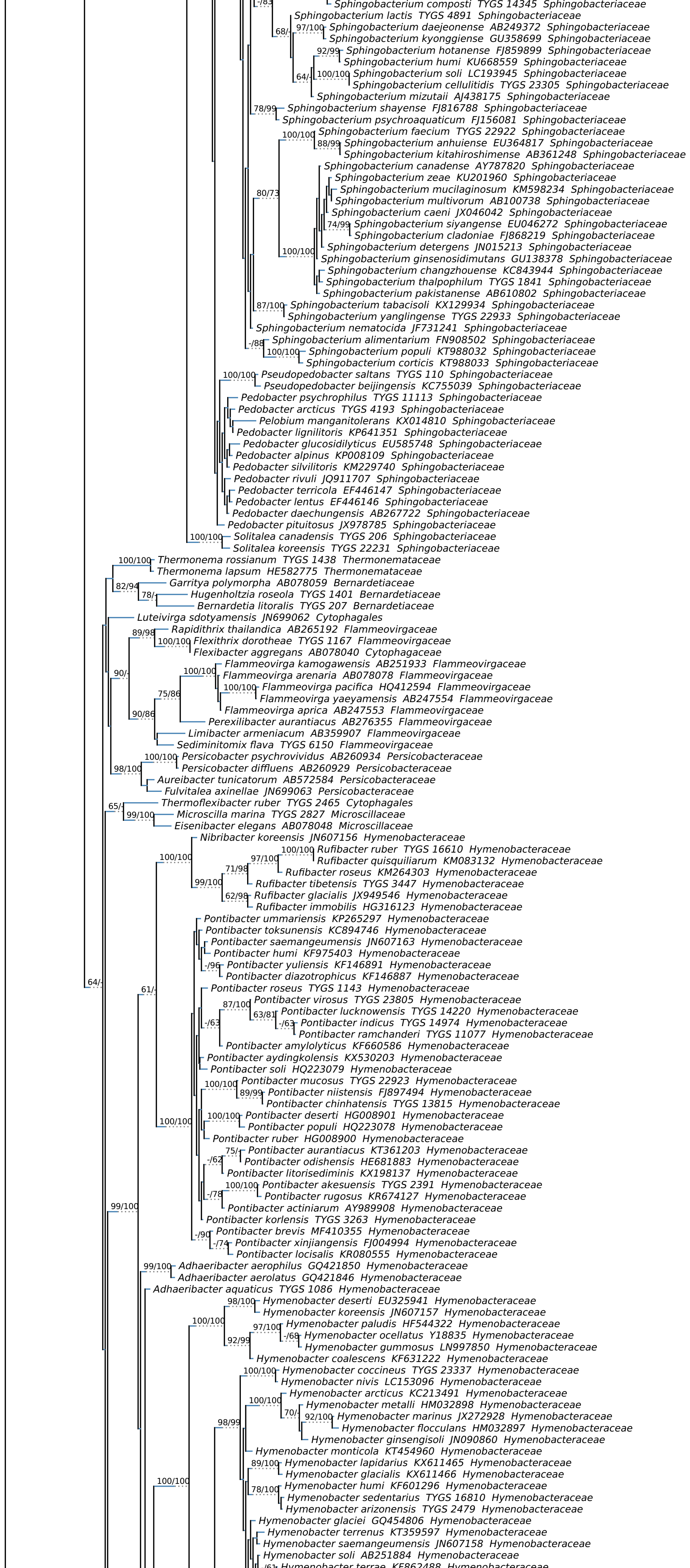
Sphingobacterium composti TYGS 14345 Sphingobacteriaceae
Sphingobacterium lactis TYGS 4891 Sphingobacteriaceae
Sphingobacterium daejeonense AB249372 Sphingobacteriaceae
Sphingobacterium kyonggiense GU358699 Sphingobacteriaceae
Sphingobacterium hotanense FJ859899 Sphingobacteriaceae
Sphingobacterium humi KU668559 Sphingobacteriaceae
Sphingobacterium soli LC193945 Sphingobacteriaceae
Sphingobacterium cellulitidis TYGS 23305 Sphingobacteriaceae
Sphingobacterium mizutaii AJ438175 Sphingobacteriaceae
Sphingobacterium shayense FJ816788 Sphingobacteriaceae
Sphingobacterium psychroaquaticum FJ156081 Sphingobacteriaceae
Sphingobacterium faecium TYGS 22922 Sphingobacteriaceae
Sphingobacterium anhuiense EU364817 Sphingobacteriaceae
Sphingobacterium kitahiroshimense AB361248 Sphingobacteriaceae
Sphingobacterium canadense AY787820 Sphingobacteriaceae
Sphingobacterium zeae KU201960 Sphingobacteriaceae
Sphingobacterium mucilaginosum KM598234 Sphingobacteriaceae
Sphingobacterium multivorum AB100738 Sphingobacteriaceae
Sphingobacterium caeni JX046042 Sphingobacteriaceae
Sphingobacterium siyangense EU046272 Sphingobacteriaceae
Sphingobacterium cladoniae FJ868219 Sphingobacteriaceae
Sphingobacterium detergens JN015213 Sphingobacteriaceae
Sphingobacterium ginsenosidimutans GU138378 Sphingobacteriaceae
Sphingobacterium changzhouense KC843944 Sphingobacteriaceae
Sphingobacterium thalpophilum TYGS 1841 Sphingobacteriaceae
Sphingobacterium pakistanense AB610802 Sphingobacteriaceae
Sphingobacterium tabacisoli KX129934 Sphingobacteriaceae
Sphingobacterium yanglingense TYGS 22933 Sphingobacteriaceae
Sphingobacterium nematocida JF731241 Sphingobacteriaceae
Sphingobacterium alimentarium FN908502 Sphingobacteriaceae
Sphingobacterium populi KT988032 Sphingobacteriaceae
Sphingobacterium corticis KT988033 Sphingobacteriaceae
Pseudopedobacter saltans TYGS 110 Sphingobacteriaceae
Pseudopedobacter beijingensis KC755039 Sphingobacteriaceae
Pedobacter psychrophilus TYGS 11113 Sphingobacteriaceae
Pedobacter arcticus TYGS 4193 Sphingobacteriaceae
Pelobium manganitolerans KX014810 Sphingobacteriaceae
Pedobacter lignilitoris KP641351 Sphingobacteriaceae
Pedobacter glucosidilyticus EU585748 Sphingobacteriaceae
Pedobacter alpinus KP008109 Sphingobacteriaceae
Pedobacter silvilitoris KM229740 Sphingobacteriaceae
Pedobacter rivuli JQ911707 Sphingobacteriaceae
Pedobacter terricola EF446147 Sphingobacteriaceae
Pedobacter lentus EF446146 Sphingobacteriaceae
Pedobacter daechungensis AB267722 Sphingobacteriaceae
Pedobacter pituitosus JX978785 Sphingobacteriaceae
Solitalea canadensis TYGS 206 Sphingobacteriaceae
Solitalea koreensis TYGS 22231 Sphingobacteriaceae
Thermonema rossianum TYGS 1438 Thermonemataceae
Thermonema lapsum HE582775 Thermonemataceae
Garritya polymorpha AB078059 Bernardetiaceae
Hugenholtzia roseola TYGS 1401 Bernardetiaceae
Bernardetia litoralis TYGS 207 Bernardetiaceae
Luteivirga sdotyamensis JN699062 Cytophagales
Rapidithrix thailandica AB265192 Flammeovirgaceae
Flexithrix dorotheae TYGS 1167 Flammeovirgaceae
Flexibacter aggregans AB078040 Cytophagaceae
Flammeovirga kamogawensis AB251933 Flammeovirgaceae
Flammeovirga arenaria AB078078 Flammeovirgaceae
Flammeovirga pacifica HQ412594 Flammeovirgaceae
Flammeovirga yaeyamensis AB247554 Flammeovirgaceae
Flammeovirga aprica AB247553 Flammeovirgaceae
Perexilibacter aurantiacus AB276355 Flammeovirgaceae
Limibacter armeniacum AB359907 Flammeovirgaceae
Sediminitomix flava TYGS 6150 Flammeovirgaceae
Persicobacter psychrovividus AB260934 Persicobacteraceae
Persicobacter diffluens AB260929 Persicobacteraceae
Aureibacter tunicatorum AB572584 Persicobacteraceae
Fulvitalea axinellae JN699063 Persicobacteraceae
Thermoflexibacter ruber TYGS 2465 Cytophagales
Microscilla marina TYGS 2827 Microscillaceae
Eisenibacter elegans AB078048 Microscillaceae
Nibribacter koreensis JN607156 Hymenobacteraceae
Rufibacter ruber TYGS 16610 Hymenobacteraceae
Rufibacter quisquiliarum KM083132 Hymenobacteraceae
Rufibacter roseus KM264303 Hymenobacteraceae
Rufibacter tibetensis TYGS 3447 Hymenobacteraceae
Rufibacter glacialis JX949546 Hymenobacteraceae
Rufibacter immobilis HG316123 Hymenobacteraceae
Pontibacter ummariensis KP265297 Hymenobacteraceae
Pontibacter toksunensis KC894746 Hymenobacteraceae
Pontibacter saemangeumensis JN607163 Hymenobacteraceae
Pontibacter humi KF975403 Hymenobacteraceae
Pontibacter yuliensis KF146891 Hymenobacteraceae
Pontibacter diazotrophicus KF146887 Hymenobacteraceae
Pontibacter roseus TYGS 1143 Hymenobacteraceae
Pontibacter virosus TYGS 23805 Hymenobacteraceae
Pontibacter lucknowensis TYGS 14220 Hymenobacteraceae
Pontibacter indicus TYGS 14974 Hymenobacteraceae
Pontibacter ramchanderi TYGS 11077 Hymenobacteraceae
Pontibacter amylolyticus KF660586 Hymenobacteraceae
Pontibacter aydingkolensis KX530203 Hymenobacteraceae
Pontibacter soli HQ223079 Hymenobacteraceae
Pontibacter mucosus TYGS 22923 Hymenobacteraceae
Pontibacter niistensis FJ897494 Hymenobacteraceae
Pontibacter chinhatensis TYGS 13815 Hymenobacteraceae
Pontibacter deserti HG008901 Hymenobacteraceae
Pontibacter populi HQ223078 Hymenobacteraceae
Pontibacter ruber HG008900 Hymenobacteraceae
Pontibacter aurantiacus KT361203 Hymenobacteraceae
Pontibacter odishensis HE681883 Hymenobacteraceae
Pontibacter litorisediminis KX198137 Hymenobacteraceae
Pontibacter akesuensis TYGS 2391 Hymenobacteraceae
Pontibacter rugosus KR674127 Hymenobacteraceae
Pontibacter actiniarum AY989908 Hymenobacteraceae
Pontibacter korlensis TYGS 3263 Hymenobacteraceae
Pontibacter brevis MF410355 Hymenobacteraceae
Pontibacter xinjiangensis FJ004994 Hymenobacteraceae
Pontibacter locisalis KR080555 Hymenobacteraceae
Adhaeribacter aerophilus GQ421850 Hymenobacteraceae
Adhaeribacter aerolatus GQ421846 Hymenobacteraceae
Adhaeribacter aquaticus TYGS 1086 Hymenobacteraceae
Hymenobacter deserti EU325941 Hymenobacteraceae
Hymenobacter koreensis JN607157 Hymenobacteraceae
Hymenobacter paludis HF544322 Hymenobacteraceae
Hymenobacter ocellatus Y18835 Hymenobacteraceae
Hymenobacter gummosus LN997850 Hymenobacteraceae
Hymenobacter coalescens KF631222 Hymenobacteraceae
Hymenobacter coccineus TYGS 23337 Hymenobacteraceae
Hymenobacter nivis LC153096 Hymenobacteraceae
Hymenobacter arcticus KC213491 Hymenobacteraceae
Hymenobacter metalli HM032898 Hymenobacteraceae
Hymenobacter marinus JX272928 Hymenobacteraceae
Hymenobacter flocculans HM032897 Hymenobacteraceae
Hymenobacter ginsengisoli JN090860 Hymenobacteraceae
Hymenobacter monticola KT454960 Hymenobacteraceae
Hymenobacter lapidarius KX611465 Hymenobacteraceae
Hymenobacter glacialis KX611466 Hymenobacteraceae
Hymenobacter humi KF601296 Hymenobacteraceae
Hymenobacter sedentarius TYGS 16810 Hymenobacteraceae
Hymenobacter arizonensis TYGS 2479 Hymenobacteraceae
Hymenobacter glaciei GQ454806 Hymenobacteraceae
Hymenobacter terrenus KT359597 Hymenobacteraceae
Hymenobacter saemangeumensis JN607158 Hymenobacteraceae
Hymenobacter soli AB251884 Hymenobacteraceae

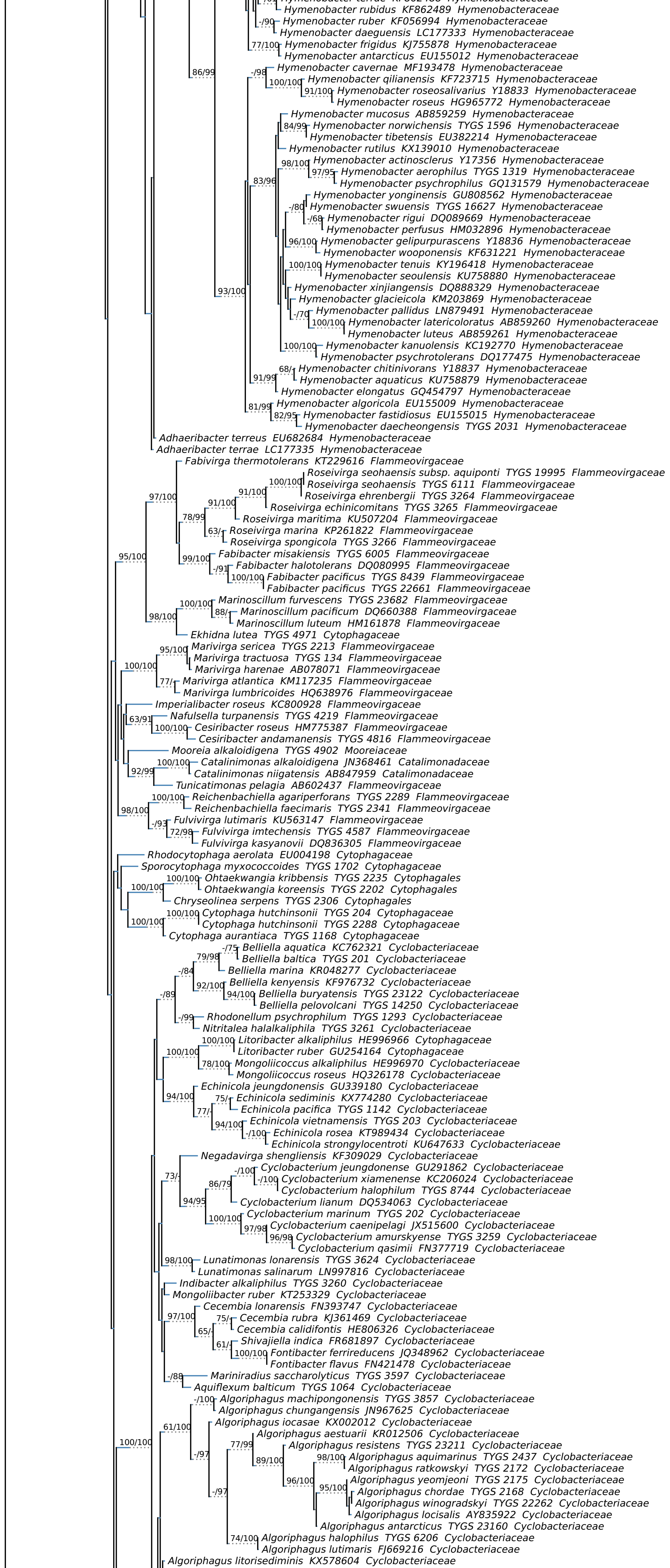
Hymenobacter rubidus KF862489 Hymenobacteraceae
Hymenobacter ruber KF056994 Hymenobacteraceae
Hymenobacter daeguensis LC177333 Hymenobacteraceae
Hymenobacter frigidus KJ755878 Hymenobacteraceae
Hymenobacter antarcticus EU155012 Hymenobacteraceae
Hymenobacter cavernae MF193478 Hymenobacteraceae
Hymenobacter qilianensis KF723715 Hymenobacteraceae
Hymenobacter roseosalivarius Y18833 Hymenobacteraceae
Hymenobacter roseus HG965772 Hymenobacteraceae
Hymenobacter mucosus AB859259 Hymenobacteraceae
Hymenobacter norwichensis TYGS 1596 Hymenobacteraceae
Hymenobacter tibetensis EU382214 Hymenobacteraceae
Hymenobacter rutilus KX139010 Hymenobacteraceae
Hymenobacter actinosclerus Y17356 Hymenobacteraceae
Hymenobacter aerophilus TYGS 1319 Hymenobacteraceae
Hymenobacter psychrophilus GQ131579 Hymenobacteraceae
Hymenobacter yonginensis GU808562 Hymenobacteraceae
Hymenobacter swuensis TYGS 16627 Hymenobacteraceae
Hymenobacter rigui DQ089669 Hymenobacteraceae
Hymenobacter perfusus HM032896 Hymenobacteraceae
Hymenobacter gelipurpurascens Y18836 Hymenobacteraceae
Hymenobacter wooponensis KF631221 Hymenobacteraceae
Hymenobacter tenuis KY196418 Hymenobacteraceae
Hymenobacter seoulensis KU758880 Hymenobacteraceae
Hymenobacter xinjiangensis DQ888329 Hymenobacteraceae
Hymenobacter glacieicola KM203869 Hymenobacteraceae
Hymenobacter pallidus LN879491 Hymenobacteraceae
Hymenobacter latericoloratus AB859260 Hymenobacteraceae
Hymenobacter luteus AB859261 Hymenobacteraceae
Hymenobacter kanuolensis KC192770 Hymenobacteraceae
Hymenobacter psychrotolerans DQ177475 Hymenobacteraceae
Hymenobacter chitinivorans Y18837 Hymenobacteraceae
Hymenobacter aquaticus KU758879 Hymenobacteraceae
Hymenobacter elongatus GQ454797 Hymenobacteraceae
Hymenobacter algoricola EU155009 Hymenobacteraceae
Hymenobacter fastidiosus EU155015 Hymenobacteraceae
Hymenobacter daecheongensis TYGS 2031 Hymenobacteraceae
Adhaeribacter terreus EU682684 Hymenobacteraceae
Adhaeribacter terrae LC177335 Hymenobacteraceae
Fabivirga thermotolerans KT229616 Flammeovirgaceae
Roseivirga seohaensis subsp. aquiponti TYGS 19995 Flammeovirgaceae
Roseivirga seohaensis TYGS 6111 Flammeovirgaceae
Roseivirga ehrenbergii TYGS 3264 Flammeovirgaceae
Roseivirga echinicomitans TYGS 3265 Flammeovirgaceae
Roseivirga maritima KU507204 Flammeovirgaceae
Roseivirga marina KP261822 Flammeovirgaceae
Roseivirga spongicola TYGS 3266 Flammeovirgaceae
Fabibacter misakiensis TYGS 6005 Flammeovirgaceae
Fabibacter halotolerans DQ080995 Flammeovirgaceae
Fabibacter pacificus TYGS 8439 Flammeovirgaceae
Fabibacter pacificus TYGS 22661 Flammeovirgaceae
Marinoscillum furvescens TYGS 23682 Flammeovirgaceae
Marinoscillum pacificum DQ660388 Flammeovirgaceae
Marinoscillum luteum HM161878 Flammeovirgaceae
Ekhidna lutea TYGS 4971 Cytophagaceae
Marivirga sericea TYGS 2213 Flammeovirgaceae
Marivirga tractuosa TYGS 134 Flammeovirgaceae
Marivirga harenae AB078071 Flammeovirgaceae
Marivirga atlantica KM117235 Flammeovirgaceae
Marivirga lumbricoides HQ638976 Flammeovirgaceae
Imperialibacter roseus KC800928 Flammeovirgaceae
Nafulsella turpanensis TYGS 4219 Flammeovirgaceae
Cesiribacter roseus HM775387 Flammeovirgaceae
Cesiribacter andamanensis TYGS 4816 Flammeovirgaceae
Mooreia alkaloidigena TYGS 4902 Mooreiaceae
Catalinimonas alkaloidigena JN368461 Catalimonadaceae
Catalinimonas niigatensis AB847959 Catalimonadaceae
Tunicatimonas pelagia AB602437 Flammeovirgaceae
Reichenbachiella agariperforans TYGS 2289 Flammeovirgaceae
Reichenbachiella faecimaris TYGS 2341 Flammeovirgaceae
Fulvivirga lutimaris KU563147 Flammeovirgaceae
Fulvivirga imtechensis TYGS 4587 Flammeovirgaceae
Fulvivirga kasyanovii DQ836305 Flammeovirgaceae
Rhodocytophaga aerolata EU004198 Cytophagaceae
Sporocytophaga myxococcoides TYGS 1702 Cytophagaceae
Ohtaekwangia kribbensis TYGS 2235 Cytophagales
Ohtaekwangia koreensis TYGS 2202 Cytophagales
Chryseolinea serpens TYGS 2306 Cytophagales
Cytophaga hutchinsonii TYGS 204 Cytophagaceae
Cytophaga hutchinsonii TYGS 2288 Cytophagaceae
Cytophaga aurantiaca TYGS 1168 Cytophagaceae
Belliella aquatica KC762321 Cyclobacteriaceae
Belliella baltica TYGS 201 Cyclobacteriaceae
Belliella marina KR048277 Cyclobacteriaceae
Belliella kenyensis KF976732 Cyclobacteriaceae
Belliella buryatensis TYGS 23122 Cyclobacteriaceae
Belliella pelovolcani TYGS 14250 Cyclobacteriaceae
Rhodonellum psychrophilum TYGS 1293 Cyclobacteriaceae
Nitritalea halalkaliphila TYGS 3261 Cyclobacteriaceae
Litoribacter alkaliphilus HE996966 Cytophagaceae
Litoribacter ruber GU254164 Cytophagaceae
Mongoliicoccus alkaliphilus HE996970 Cyclobacteriaceae
Mongoliicoccus roseus HQ326178 Cyclobacteriaceae
Echinicola jeungdonensis GU339180 Cyclobacteriaceae
Echinicola sediminis KX774280 Cyclobacteriaceae
Echinicola pacifica TYGS 1142 Cyclobacteriaceae
Echinicola vietnamensis TYGS 203 Cyclobacteriaceae
Echinicola rosea KT989434 Cyclobacteriaceae
Echinicola strongylocentroti KU647633 Cyclobacteriaceae
Negadavirga shengliensis KF309029 Cyclobacteriaceae
Cyclobacterium jeungdonense GU291862 Cyclobacteriaceae
Cyclobacterium xiamenense KC206024 Cyclobacteriaceae
Cyclobacterium halophilum TYGS 8744 Cyclobacteriaceae
Cyclobacterium lianum DQ534063 Cyclobacteriaceae
Cyclobacterium marinum TYGS 202 Cyclobacteriaceae
Cyclobacterium caenipelagi JX515600 Cyclobacteriaceae
Cyclobacterium amurskyense TYGS 3259 Cyclobacteriaceae
Cyclobacterium qasimii FN377719 Cyclobacteriaceae
Lunatimonas lonarensis TYGS 3624 Cyclobacteriaceae
Lunatimonas salinarum LN997816 Cyclobacteriaceae
Indibacter alkaliphilus TYGS 3260 Cyclobacteriaceae
Mongoliibacter ruber KT253329 Cyclobacteriaceae
Cecembia lonarensis FN393747 Cyclobacteriaceae
Cecembia rubra KJ361469 Cyclobacteriaceae
Cecembia calidifontis HE806326 Cyclobacteriaceae
Shivajiella indica FR681897 Cyclobacteriaceae
Fontibacter ferrireducens JQ348962 Cyclobacteriaceae
Fontibacter flavus FN421478 Cyclobacteriaceae
Mariniradius saccharolyticus TYGS 3597 Cyclobacteriaceae
Aquiflexum balticum TYGS 1064 Cyclobacteriaceae
Algoriphagus machipongonensis TYGS 3857 Cyclobacteriaceae
Algoriphagus chungangensis JN967625 Cyclobacteriaceae
Algoriphagus iocasae KX002012 Cyclobacteriaceae
Algoriphagus aestuarii KR012506 Cyclobacteriaceae
Algoriphagus resistens TYGS 23211 Cyclobacteriaceae
Algoriphagus aquimarinus TYGS 2437 Cyclobacteriaceae
Algoriphagus ratkowskyi TYGS 2172 Cyclobacteriaceae
Algoriphagus yeomjeoni TYGS 2175 Cyclobacteriaceae
Algoriphagus chordae TYGS 2168 Cyclobacteriaceae
Algoriphagus winogradskyi TYGS 22262 Cyclobacteriaceae
Algoriphagus locisalis AY835922 Cyclobacteriaceae
Algoriphagus antarcticus TYGS 23160 Cyclobacteriaceae
Algoriphagus halophilus TYGS 6206 Cyclobacteriaceae
Algoriphagus lutimaris FJ669216 Cyclobacteriaceae
Algoriphagus litorisediminis KX578604 Cyclobacteriaceae

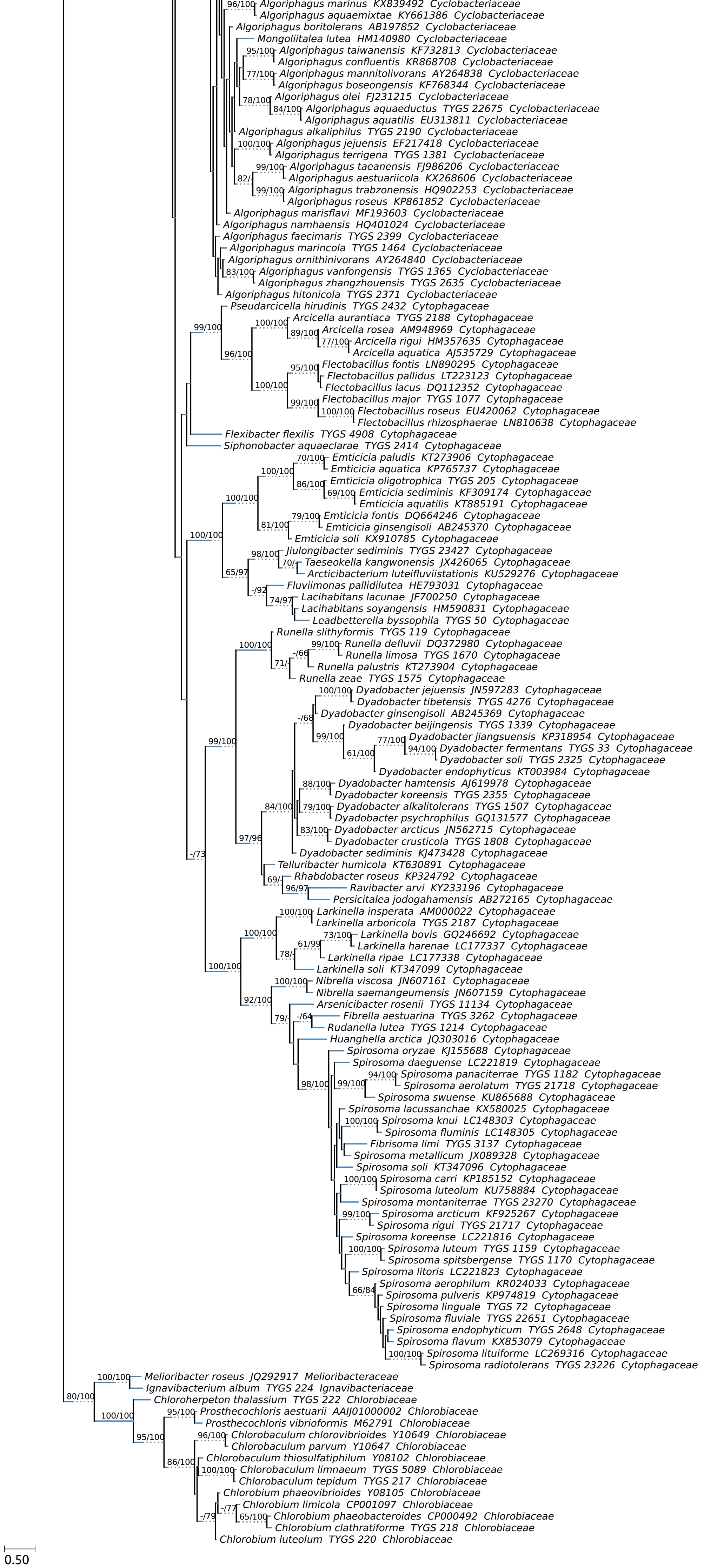
Algoriphagus marinus KX839492 Cyclobacteriaceae
Algoriphagus aquaemixtae KY661386 Cyclobacteriaceae
Algoriphagus boritolerans AB197852 Cyclobacteriaceae
Mongoliitalea lutea HM140980 Cyclobacteriaceae
Algoriphagus taiwanensis KF732813 Cyclobacteriaceae
Algoriphagus confluentis KR868708 Cyclobacteriaceae
Algoriphagus mannitolivorans AY264838 Cyclobacteriaceae
Algoriphagus boseongensis KF768344 Cyclobacteriaceae
Algoriphagus olei FJ231215 Cyclobacteriaceae
Algoriphagus aquaeductus TYGS 22675 Cyclobacteriaceae
Algoriphagus aquatilis EU313811 Cyclobacteriaceae
Algoriphagus alkaliphilus TYGS 2190 Cyclobacteriaceae
Algoriphagus jejuensis EF217418 Cyclobacteriaceae
Algoriphagus terrigena TYGS 1381 Cyclobacteriaceae
Algoriphagus taeanensis FJ986206 Cyclobacteriaceae
Algoriphagus aestuariicola KX268606 Cyclobacteriaceae
Algoriphagus trabzonensis HQ902253 Cyclobacteriaceae
Algoriphagus roseus KP861852 Cyclobacteriaceae
Algoriphagus marisflavi MF193603 Cyclobacteriaceae
Algoriphagus namhaensis HQ401024 Cyclobacteriaceae
Algoriphagus faecimaris TYGS 2399 Cyclobacteriaceae
Algoriphagus marincola TYGS 1464 Cyclobacteriaceae
Algoriphagus ornithinivorans AY264840 Cyclobacteriaceae
Algoriphagus vanfongensis TYGS 1365 Cyclobacteriaceae
Algoriphagus zhangzhouensis TYGS 2635 Cyclobacteriaceae
Algoriphagus hitonicola TYGS 2371 Cyclobacteriaceae
Pseudarcicella hirudinis TYGS 2432 Cytophagaceae
Arcicella aurantiaca TYGS 2188 Cytophagaceae
Arcicella rosea AM948969 Cytophagaceae
Arcicella rigui HM357635 Cytophagaceae
Arcicella aquatica AJ535729 Cytophagaceae
Flectobacillus fontis LN890295 Cytophagaceae
Flectobacillus pallidus LT223123 Cytophagaceae
Flectobacillus lacus DQ112352 Cytophagaceae
Flectobacillus major TYGS 1077 Cytophagaceae
Flectobacillus roseus EU420062 Cytophagaceae
Flectobacillus rhizosphaerae LN810638 Cytophagaceae
Flexibacter flexilis TYGS 4908 Cytophagaceae
Siphonobacter aquaeclarae TYGS 2414 Cytophagaceae
Emticicia paludis KT273906 Cytophagaceae
Emticicia aquatica KP765737 Cytophagaceae
Emticicia oligotrophica TYGS 205 Cytophagaceae
Emticicia sediminis KF309174 Cytophagaceae
Emticicia aquatilis KT885191 Cytophagaceae
Emticicia fontis DQ664246 Cytophagaceae
Emticicia ginsengisoli AB245370 Cytophagaceae
Emticicia soli KX910785 Cytophagaceae
Jiulongibacter sediminis TYGS 23427 Cytophagaceae
Taeseokella kangwonensis JX426065 Cytophagaceae
Arcticibacterium luteifluviistationis KU529276 Cytophagaceae
Fluviimonas pallidilutea HE793031 Cytophagaceae
Lacihabitans lacunae JF700250 Cytophagaceae
Lacihabitans soyangensis HM590831 Cytophagaceae
Leadbetterella byssophila TYGS 50 Cytophagaceae
Runella slithyformis TYGS 119 Cytophagaceae
Runella defluvii DQ372980 Cytophagaceae
Runella limosa TYGS 1670 Cytophagaceae
Runella palustris KT273904 Cytophagaceae
Runella zeae TYGS 1575 Cytophagaceae
Dyadobacter jejuensis JN597283 Cytophagaceae
Dyadobacter tibetensis TYGS 4276 Cytophagaceae
Dyadobacter ginsengisoli AB245369 Cytophagaceae
Dyadobacter beijingensis TYGS 1339 Cytophagaceae
Dyadobacter jiangsuensis KP318954 Cytophagaceae
Dyadobacter fermentans TYGS 33 Cytophagaceae
Dyadobacter soli TYGS 2325 Cytophagaceae
Dyadobacter endophyticus KT003984 Cytophagaceae
Dyadobacter hamtensis AJ619978 Cytophagaceae
Dyadobacter koreensis TYGS 2355 Cytophagaceae
Dyadobacter alkalitolerans TYGS 1507 Cytophagaceae
Dyadobacter psychrophilus GQ131577 Cytophagaceae
Dyadobacter arcticus JN562715 Cytophagaceae
Dyadobacter crusticola TYGS 1808 Cytophagaceae
Dyadobacter sediminis KJ473428 Cytophagaceae
Telluribacter humicola KT630891 Cytophagaceae
Rhabdobacter roseus KP324792 Cytophagaceae
Ravibacter arvi KY233196 Cytophagaceae
Persicitalea jodogahamensis AB272165 Cytophagaceae
Larkinella insperata AM000022 Cytophagaceae
Larkinella arboricola TYGS 2187 Cytophagaceae
Larkinella bovis GQ246692 Cytophagaceae
Larkinella harenae LC177337 Cytophagaceae
Larkinella ripae LC177338 Cytophagaceae
Larkinella soli KT347099 Cytophagaceae
Nibrella viscosa JN607161 Cytophagaceae
Nibrella saemangeumensis JN607159 Cytophagaceae
Arsenicibacter rosenii TYGS 11134 Cytophagaceae
Fibrella aestuarina TYGS 3262 Cytophagaceae
Rudanella lutea TYGS 1214 Cytophagaceae
Huanghella arctica JQ303016 Cytophagaceae
Spirosoma oryzae KJ155688 Cytophagaceae
Spirosoma daeguense LC221819 Cytophagaceae
Spirosoma panaciterrae TYGS 1182 Cytophagaceae
Spirosoma aerolatum TYGS 21718 Cytophagaceae
Spirosoma swuense KU865688 Cytophagaceae
Spirosoma lacussanchae KX580025 Cytophagaceae
Spirosoma knui LC148303 Cytophagaceae
Spirosoma fluminis LC148305 Cytophagaceae
Fibrisoma limi TYGS 3137 Cytophagaceae
Spirosoma metallicum JX089328 Cytophagaceae
Spirosoma soli KT347096 Cytophagaceae
Spirosoma carri KP185152 Cytophagaceae
Spirosoma luteolum KU758884 Cytophagaceae
Spirosoma montaniterrae TYGS 23270 Cytophagaceae
Spirosoma arcticum KF925267 Cytophagaceae
Spirosoma rigui TYGS 21717 Cytophagaceae
Spirosoma koreense LC221816 Cytophagaceae
Spirosoma luteum TYGS 1159 Cytophagaceae
Spirosoma spitsbergense TYGS 1170 Cytophagaceae
Spirosoma litoris LC221823 Cytophagaceae
Spirosoma aerophilum KR024033 Cytophagaceae
Spirosoma pulveris KP974819 Cytophagaceae
Spirosoma linguale TYGS 72 Cytophagaceae
Spirosoma fluviale TYGS 22651 Cytophagaceae
Spirosoma endophyticum TYGS 2648 Cytophagaceae
Spirosoma flavum KX853079 Cytophagaceae
Spirosoma lituiforme LC269316 Cytophagaceae
Spirosoma radiotolerans TYGS 23226 Cytophagaceae
Melioribacter roseus JQ292917 Melioribacteraceae
Ignavibacterium album TYGS 224 Ignavibacteriaceae
Chloroherpeton thalassium TYGS 222 Chlorobiaceae
Prosthecochloris aestuarii AAIJ01000002 Chlorobiaceae
Prosthecochloris vibrioformis M62791 Chlorobiaceae
Chlorobaculum chlorovibrioides Y10649 Chlorobiaceae
Chlorobaculum parvum Y10647 Chlorobiaceae
Chlorobaculum thiosulfatiphilum Y08102 Chlorobiaceae
Chlorobaculum limnaeum TYGS 5089 Chlorobiaceae
Chlorobaculum tepidum TYGS 217 Chlorobiaceae
Chlorobium phaeovibrioides Y08105 Chlorobiaceae
Chlorobium limicola CP001097 Chlorobiaceae
Chlorobium phaeobacteroides CP000492 Chlorobiaceae
Chlorobium clathratiforme TYGS 218 Chlorobiaceae
Chlorobium luteolum TYGS 220 Chlorobiaceae
0.50

Figure 4: Unconstrained comprehensive 16S rRNA gene ML tree (UCT) of *Bacteroidetes* inferred under the GTR+CAT model. The branches are scaled in terms of the expected number of substitutions per site. The numbers above the branches are support values when larger than 60% from ML (left) and MP (right) bootstrapping. Dotted parts of branches are filled in to allow proper placement of bootstrap values and are not part of the actual branch length. Numbers preceeded by the term 'TYGS' in labels refer to the genome IDs as found in Supplementary Table S1 (first sheet). Each tip label ends with the family of the respective taxon.

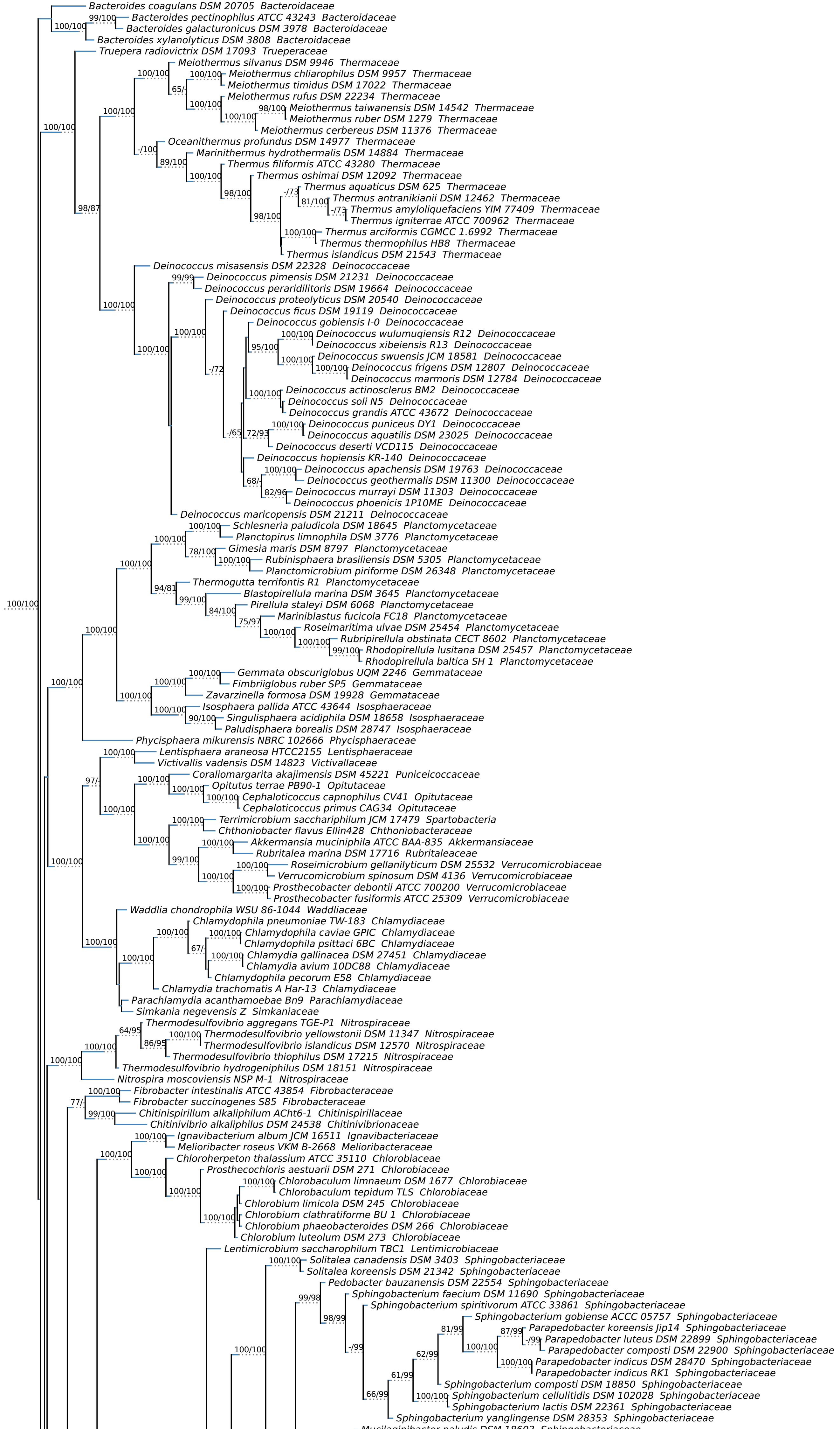

Bacteroides coagulans DSM 20705 Bacteroidaceae
Bacteroides pectinophilus ATCC 43243 Bacteroidaceae
Bacteroides galacturonicus DSM 3978 Bacteroidaceae
Bacteroides xylanolyticus DSM 3808 Bacteroidaceae
Truepera radiovictrix DSM 17093 Trueperaceae
Meiothermus silvanus DSM 9946 Thermaceae
Meiothermus chliarophilus DSM 9957 Thermaceae
Meiothermus timidus DSM 17022 Thermaceae
Meiothermus rufus DSM 22234 Thermaceae
Meiothermus taiwanensis DSM 14542 Thermaceae
Meiothermus ruber DSM 1279 Thermaceae
Meiothermus cerbereus DSM 11376 Thermaceae
Oceanithermus profundus DSM 14977 Thermaceae
Marinithermus hydrothermalis DSM 14884 Thermaceae
Thermus filiformis ATCC 43280 Thermaceae
Thermus oshimai DSM 12092 Thermaceae
Thermus aquaticus DSM 625 Thermaceae
Thermus antranikianii DSM 12462 Thermaceae
Thermus amyloliquefaciens YIM 77409 Thermaceae
Thermus igniterrae ATCC 700962 Thermaceae
Thermus arciformis CGMCC 1.6992 Thermaceae
Thermus thermophilus HB8 Thermaceae
Thermus islandicus DSM 21543 Thermaceae
Deinococcus misasensis DSM 22328 Deinococcaceae
Deinococcus pimensis DSM 21231 Deinococcaceae
Deinococcus peraridilitoris DSM 19664 Deinococcaceae
Deinococcus proteolyticus DSM 20540 Deinococcaceae
Deinococcus ficus DSM 19119 Deinococcaceae
Deinococcus gobiensis I-0 Deinococcaceae
Deinococcus wulumuqiensis R12 Deinococcaceae
Deinococcus xibeiensis R13 Deinococcaceae
Deinococcus swuensis JCM 18581 Deinococcaceae
Deinococcus frigens DSM 12807 Deinococcaceae
Deinococcus marmoris DSM 12784 Deinococcaceae
Deinococcus actinosclerus BM2 Deinococcaceae
Deinococcus soli N5 Deinococcaceae
Deinococcus grandis ATCC 43672 Deinococcaceae
Deinococcus puniceus DY1 Deinococcaceae
Deinococcus aquatilis DSM 23025 Deinococcaceae
Deinococcus deserti VCD115 Deinococcaceae
Deinococcus hopiensis KR-140 Deinococcaceae
Deinococcus apachensis DSM 19763 Deinococcaceae
Deinococcus geothermalis DSM 11300 Deinococcaceae
Deinococcus murrayi DSM 11303 Deinococcaceae
Deinococcus phoenicis 1P10ME Deinococcaceae
Deinococcus maricopensis DSM 21211 Deinococcaceae
Schlesneria paludicola DSM 18645 Planctomycetaceae
Planctopirus limnophila DSM 3776 Planctomycetaceae
Gimesia maris DSM 8797 Planctomycetaceae
Rubinisphaera brasiliensis DSM 5305 Planctomycetaceae
Planctomicrobium piriforme DSM 26348 Planctomycetaceae
Thermogutta terrifontis R1 Planctomycetaceae
Blastopirellula marina DSM 3645 Planctomycetaceae
Pirellula staleyi DSM 6068 Planctomycetaceae
Mariniblastus fucicola FC18 Planctomycetaceae
Roseimaritima ulvae DSM 25454 Planctomycetaceae
Rubripirellula obstinata CECT 8602 Planctomycetaceae
Rhodopirellula lusitana DSM 25457 Planctomycetaceae
Rhodopirellula baltica SH 1 Planctomycetaceae
Gemmata obscuriglobus UQM 2246 Gemmataceae
Fimbriiglobus ruber SP5 Gemmataceae
Zavarzinella formosa DSM 19928 Gemmataceae
Isosphaera pallida ATCC 43644 Isosphaeraceae
Singulisphaera acidiphila DSM 18658 Isosphaeraceae
Paludisphaera borealis DSM 28747 Isosphaeraceae
Phycisphaera mikurensis NBRC 102666 Phycisphaeraceae
Lentisphaera araneosa HTCC2155 Lentisphaeraceae
Victivallis vadensis DSM 14823 Victivallaceae
Coraliomargarita akajimensis DSM 45221 Puniceicoccaceae
Opitutus terrae PB90-1 Opitutaceae
Cephaloticoccus capnophilus CV41 Opitutaceae
Cephaloticoccus primus CAG34 Opitutaceae
Terrimicrobium sacchariphilum JCM 17479 Spartobacteria
Chthoniobacter flavus Ellin428 Chthoniobacteraceae
Akkermansia muciniphila ATCC BAA-835 Akkermansiaceae
Rubritalea marina DSM 17716 Rubritaleaceae
Roseimicrobium gellanilyticum DSM 25532 Verrucomicrobiaceae
Verrucomicrobium spinosum DSM 4136 Verrucomicrobiaceae
Prosthecobacter debontii ATCC 700200 Verrucomicrobiaceae
Prosthecobacter fusiformis ATCC 25309 Verrucomicrobiaceae
Waddlia chondrophila WSU 86-1044 Waddliaceae
Chlamydophila pneumoniae TW-183 Chlamydiaceae
Chlamydophila caviae GPIC Chlamydiaceae
Chlamydophila psittaci 6BC Chlamydiaceae
Chlamydia gallinacea DSM 27451 Chlamydiaceae
Chlamydia avium 10DC88 Chlamydiaceae
Chlamydophila pecorum E58 Chlamydiaceae
Chlamydia trachomatis A Har-13 Chlamydiaceae
Parachlamydia acanthamoebae Bn9 Parachlamydiaceae
Simkania negevensis Z Simkaniaceae
Thermodesulfovibrio aggregans TGE-P1 Nitrospiraceae
Thermodesulfovibrio yellowstonii DSM 11347 Nitrospiraceae
Thermodesulfovibrio islandicus DSM 12570 Nitrospiraceae
Thermodesulfovibrio thiophilus DSM 17215 Nitrospiraceae
Thermodesulfovibrio hydrogeniphilus DSM 18151 Nitrospiraceae
Nitrospira moscoviensis NSP M-1 Nitrospiraceae
Fibrobacter intestinalis ATCC 43854 Fibrobacteraceae
Fibrobacter succinogenes S85 Fibrobacteraceae
Chitinispirillum alkaliphilum ACht6-1 Chitinispirillaceae
Chitinivibrio alkaliphilus DSM 24538 Chitinivibrionaceae
Ignavibacterium album JCM 16511 Ignavibacteriaceae
Melioribacter roseus VKM B-2668 Melioribacteraceae
Chloroherpeton thalassium ATCC 35110 Chlorobiaceae
Prosthecochloris aestuarii DSM 271 Chlorobiaceae
Chlorobaculum limnaeum DSM 1677 Chlorobiaceae
Chlorobaculum tepidum TLS Chlorobiaceae
Chlorobium limicola DSM 245 Chlorobiaceae
Chlorobium clathratiforme BU 1 Chlorobiaceae
Chlorobium phaeobacteroides DSM 266 Chlorobiaceae
Chlorobium luteolum DSM 273 Chlorobiaceae
Lentimicrobium saccharophilum TBC1 Lentimicrobiaceae
Solitalea canadensis DSM 3403 Sphingobacteriaceae
Solitalea koreensis DSM 21342 Sphingobacteriaceae
Pedobacter bauzanensis DSM 22554 Sphingobacteriaceae
Sphingobacterium faecium DSM 11690 Sphingobacteriaceae
Sphingobacterium spiritivorum ATCC 33861 Sphingobacteriaceae
Sphingobacterium gobiense ACCC 05757 Sphingobacteriaceae
Parapedobacter koreensis Jip14 Sphingobacteriaceae
Parapedobacter luteus DSM 22899 Sphingobacteriaceae
Parapedobacter composti DSM 22900 Sphingobacteriaceae
Parapedobacter indicus DSM 28470 Sphingobacteriaceae
Parapedobacter indicus RK1 Sphingobacteriaceae
Sphingobacterium composti DSM 18850 Sphingobacteriaceae
Sphingobacterium cellulitidis DSM 102028 Sphingobacteriaceae
Sphingobacterium lactis DSM 22361 Sphingobacteriaceae
Sphingobacterium yanglingense DSM 28353 Sphingobacteriaceae

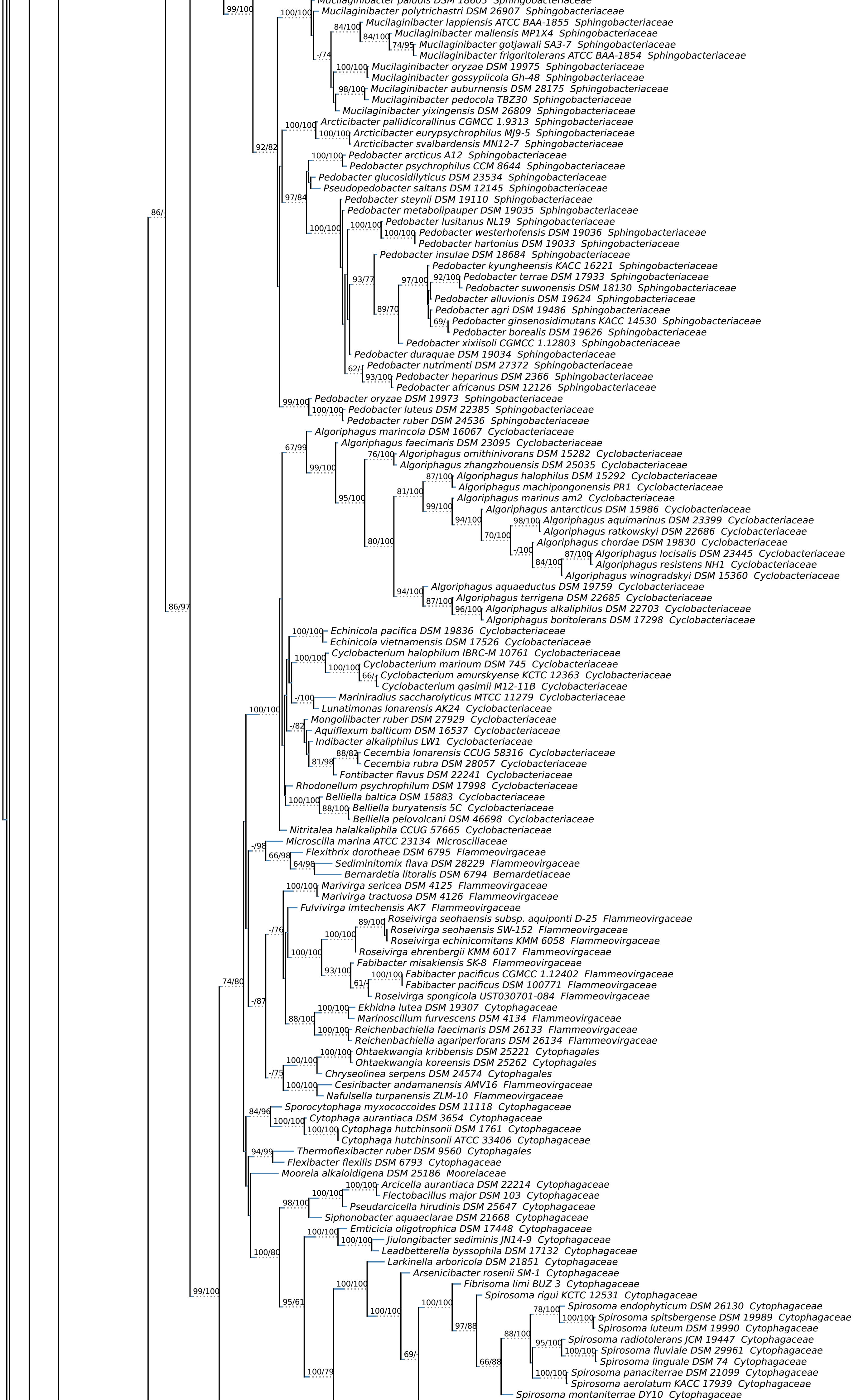
Mucilaginibacter paludis DSM 18603 Sphingobacteriaceae
Mucilaginibacter polytrichastri DSM 26907 Sphingobacteriaceae
Mucilaginibacter lappiensis ATCC BAA-1855 Sphingobacteriaceae
Mucilaginibacter mallensis MP1X4 Sphingobacteriaceae
Mucilaginibacter gotjawali SA3-7 Sphingobacteriaceae
Mucilaginibacter frigoritolerans ATCC BAA-1854 Sphingobacteriaceae
Mucilaginibacter oryzae DSM 19975 Sphingobacteriaceae
Mucilaginibacter gossypiicola Gh-48 Sphingobacteriaceae
Mucilaginibacter auburnensis DSM 28175 Sphingobacteriaceae
Mucilaginibacter pedocola TBZ30 Sphingobacteriaceae
Mucilaginibacter yixingensis DSM 26809 Sphingobacteriaceae
Arcticibacter pallidicorallinus CGMCC 1.9313 Sphingobacteriaceae
Arcticibacter eurypsychrophilus MJ9-5 Sphingobacteriaceae
Arcticibacter svalbardensis MN12-7 Sphingobacteriaceae
Pedobacter arcticus A12 Sphingobacteriaceae
Pedobacter psychrophilus CCM 8644 Sphingobacteriaceae
Pedobacter glucosidilyticus DSM 23534 Sphingobacteriaceae
Pseudopedobacter saltans DSM 12145 Sphingobacteriaceae
Pedobacter steynii DSM 19110 Sphingobacteriaceae
Pedobacter metabolipauper DSM 19035 Sphingobacteriaceae
Pedobacter lusitanus NL19 Sphingobacteriaceae
Pedobacter westerhofensis DSM 19036 Sphingobacteriaceae
Pedobacter hartonius DSM 19033 Sphingobacteriaceae
Pedobacter insulae DSM 18684 Sphingobacteriaceae
Pedobacter kyungheensis KACC 16221 Sphingobacteriaceae
Pedobacter terrae DSM 17933 Sphingobacteriaceae
Pedobacter suwonensis DSM 18130 Sphingobacteriaceae
Pedobacter alluvionis DSM 19624 Sphingobacteriaceae
Pedobacter agri DSM 19486 Sphingobacteriaceae
Pedobacter ginsenosidimutans KACC 14530 Sphingobacteriaceae
Pedobacter borealis DSM 19626 Sphingobacteriaceae
Pedobacter xixiisoli CGMCC 1.12803 Sphingobacteriaceae
Pedobacter duraquae DSM 19034 Sphingobacteriaceae
Pedobacter nutrimenti DSM 27372 Sphingobacteriaceae
Pedobacter heparinus DSM 2366 Sphingobacteriaceae
Pedobacter africanus DSM 12126 Sphingobacteriaceae
Pedobacter oryzae DSM 19973 Sphingobacteriaceae
Pedobacter luteus DSM 22385 Sphingobacteriaceae
Pedobacter ruber DSM 24536 Sphingobacteriaceae
Algoriphagus marincola DSM 16067 Cyclobacteriaceae
Algoriphagus faecimaris DSM 23095 Cyclobacteriaceae
Algoriphagus ornithinivorans DSM 15282 Cyclobacteriaceae
Algoriphagus zhangzhouensis DSM 25035 Cyclobacteriaceae
Algoriphagus halophilus DSM 15292 Cyclobacteriaceae
Algoriphagus machipongonensis PR1 Cyclobacteriaceae
Algoriphagus marinus am2 Cyclobacteriaceae
Algoriphagus antarcticus DSM 15986 Cyclobacteriaceae
Algoriphagus aquimarinus DSM 23399 Cyclobacteriaceae
Algoriphagus ratkowskyi DSM 22686 Cyclobacteriaceae
Algoriphagus chordae DSM 19830 Cyclobacteriaceae
Algoriphagus locisalis DSM 23445 Cyclobacteriaceae
Algoriphagus resistens NH1 Cyclobacteriaceae
Algoriphagus winogradskyi DSM 15360 Cyclobacteriaceae
Algoriphagus aquaeductus DSM 19759 Cyclobacteriaceae
Algoriphagus terrigena DSM 22685 Cyclobacteriaceae
Algoriphagus alkaliphilus DSM 22703 Cyclobacteriaceae
Algoriphagus boritolerans DSM 17298 Cyclobacteriaceae
Echinicola pacifica DSM 19836 Cyclobacteriaceae
Echinicola vietnamensis DSM 17526 Cyclobacteriaceae
Cyclobacterium halophilum IBRC-M 10761 Cyclobacteriaceae
Cyclobacterium marinum DSM 745 Cyclobacteriaceae
Cyclobacterium amurskyense KCTC 12363 Cyclobacteriaceae
Cyclobacterium qasimii M12-11B Cyclobacteriaceae
Mariniradius saccharolyticus MTCC 11279 Cyclobacteriaceae
Lunatimonas lonarensis AK24 Cyclobacteriaceae
Mongoliibacter ruber DSM 27929 Cyclobacteriaceae
Aquiflexum balticum DSM 16537 Cyclobacteriaceae
Indibacter alkaliphilus LW1 Cyclobacteriaceae
Cecembia lonarensis CCUG 58316 Cyclobacteriaceae
Cecembia rubra DSM 28057 Cyclobacteriaceae
Fontibacter flavus DSM 22241 Cyclobacteriaceae
Rhodonellum psychrophilum DSM 17998 Cyclobacteriaceae
Belliella baltica DSM 15883 Cyclobacteriaceae
Belliella buryatensis 5C Cyclobacteriaceae
Belliella pelovolcani DSM 46698 Cyclobacteriaceae
Nitritalea halalkaliphila CCUG 57665 Cyclobacteriaceae
Microscilla marina ATCC 23134 Microscillaceae
Flexithrix dorotheae DSM 6795 Flammeovirgaceae
Sediminitomix flava DSM 28229 Flammeovirgaceae
Bernardetia litoralis DSM 6794 Bernardetiaceae
Marivirga sericea DSM 4125 Flammeovirgaceae
Marivirga tractuosa DSM 4126 Flammeovirgaceae
Fulvivirga imtechensis AK7 Flammeovirgaceae
Roseivirga seohaensis subsp. aquiponti D-25 Flammeovirgaceae
Roseivirga seohaensis SW-152 Flammeovirgaceae
Roseivirga echinicomitans KMM 6058 Flammeovirgaceae
Roseivirga ehrenbergii KMM 6017 Flammeovirgaceae
Fabibacter misakiensis SK-8 Flammeovirgaceae
Fabibacter pacificus CGMCC 1.12402 Flammeovirgaceae
Fabibacter pacificus DSM 100771 Flammeovirgaceae
Roseivirga spongicola UST030701-084 Flammeovirgaceae
Ekhidna lutea DSM 19307 Cytophagaceae
Marinoscillum furvescens DSM 4134 Flammeovirgaceae
Reichenbachiella faecimaris DSM 26133 Flammeovirgaceae
Reichenbachiella agariperforans DSM 26134 Flammeovirgaceae
Ohtaekwangia kribbensis DSM 25221 Cytophagales
Ohtaekwangia koreensis DSM 25262 Cytophagales
Chryseolinea serpens DSM 24574 Cytophagales
Cesiribacter andamanensis AMV16 Flammeovirgaceae
Nafulsella turpanensis ZLM-10 Flammeovirgaceae
Sporocytophaga myxococcoides DSM 11118 Cytophagaceae
Cytophaga aurantiaca DSM 3654 Cytophagaceae
Cytophaga hutchinsonii DSM 1761 Cytophagaceae
Cytophaga hutchinsonii ATCC 33406 Cytophagaceae
Thermoflexibacter ruber DSM 9560 Cytophagales
Flexibacter flexilis DSM 6793 Cytophagaceae
Mooreia alkaloidigena DSM 25186 Mooreiaceae
Arcicella aurantiaca DSM 22214 Cytophagaceae
Flectobacillus major DSM 103 Cytophagaceae
Pseudarcicella hirudinis DSM 25647 Cytophagaceae
Siphonobacter aquaeclarae DSM 21668 Cytophagaceae
Emticicia oligotrophica DSM 17448 Cytophagaceae
Jiulongibacter sediminis JN14-9 Cytophagaceae
Leadbetterella byssophila DSM 17132 Cytophagaceae
Larkinella arboricola DSM 21851 Cytophagaceae
Arsenicibacter rosenii SM-1 Cytophagaceae
Fibrisoma limi BUZ 3 Cytophagaceae
Spirosoma rigui KCTC 12531 Cytophagaceae
Spirosoma endophyticum DSM 26130 Cytophagaceae
Spirosoma spitsbergense DSM 19989 Cytophagaceae
Spirosoma luteum DSM 19990 Cytophagaceae
Spirosoma radiotolerans JCM 19447 Cytophagaceae
Spirosoma fluviale DSM 29961 Cytophagaceae
Spirosoma linguale DSM 74 Cytophagaceae
Spirosoma panaciterrae DSM 21099 Cytophagaceae
Spirosoma aerolatum KACC 17939 Cytophagaceae
Spirosoma montaniterrae DY10 Cytophagaceae

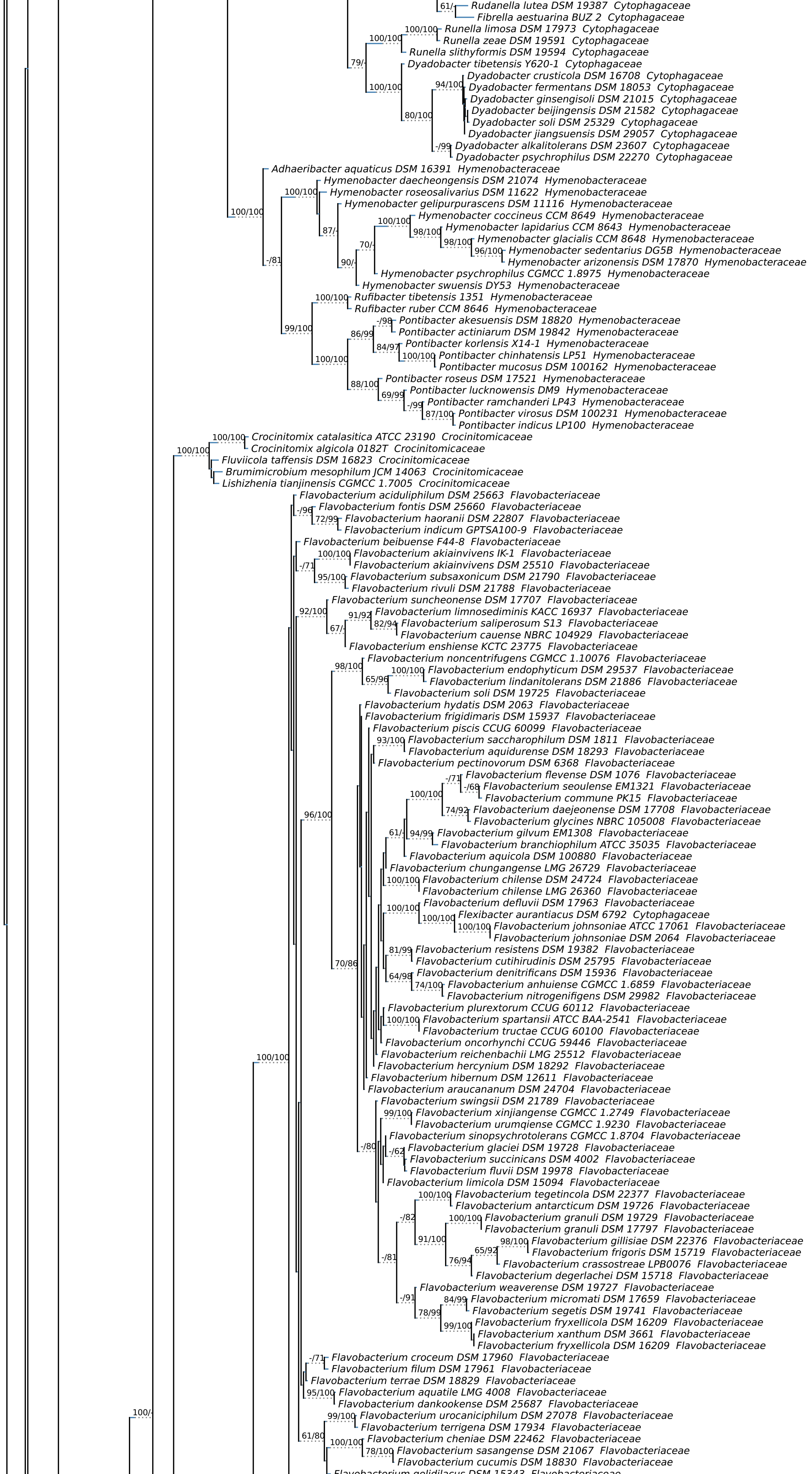
Rudanella lutea DSM 19387 Cytophagaceae
Fibrella aestuarina BUZ 2 Cytophagaceae
Runella limosa DSM 17973 Cytophagaceae
Runella zeae DSM 19591 Cytophagaceae
Runella slithyformis DSM 19594 Cytophagaceae
Dyadobacter tibetensis Y620-1 Cytophagaceae
Dyadobacter crusticola DSM 16708 Cytophagaceae
Dyadobacter fermentans DSM 18053 Cytophagaceae
Dyadobacter ginsengisoli DSM 21015 Cytophagaceae
Dyadobacter beijingensis DSM 21582 Cytophagaceae
Dyadobacter soli DSM 25329 Cytophagaceae
Dyadobacter jiangsuensis DSM 29057 Cytophagaceae
Dyadobacter alkalitolerans DSM 23607 Cytophagaceae
Dyadobacter psychrophilus DSM 22270 Cytophagaceae
Adhaeribacter aquaticus DSM 16391 Hymenobacteraceae
Hymenobacter daecheongensis DSM 21074 Hymenobacteraceae
Hymenobacter roseosalivarius DSM 11622 Hymenobacteraceae
Hymenobacter gelipurpurascens DSM 11116 Hymenobacteraceae
Hymenobacter coccineus CCM 8649 Hymenobacteraceae
Hymenobacter lapidarius CCM 8643 Hymenobacteraceae
Hymenobacter glacialis CCM 8648 Hymenobacteraceae
Hymenobacter sedentarius DG5B Hymenobacteraceae
Hymenobacter arizonensis DSM 17870 Hymenobacteraceae
Hymenobacter psychrophilus CGMCC 1.8975 Hymenobacteraceae
Hymenobacter swuensis DY53 Hymenobacteraceae
Rufibacter tibetensis 1351 Hymenobacteraceae
Rufibacter ruber CCM 8646 Hymenobacteraceae
Pontibacter akesuensis DSM 18820 Hymenobacteraceae
Pontibacter actiniarum DSM 19842 Hymenobacteraceae
Pontibacter korlensis X14-1 Hymenobacteraceae
Pontibacter chinhatensis LP51 Hymenobacteraceae
Pontibacter mucosus DSM 100162 Hymenobacteraceae
Pontibacter roseus DSM 17521 Hymenobacteraceae
Pontibacter lucknowensis DM9 Hymenobacteraceae
Pontibacter ramchanderi LP43 Hymenobacteraceae
Pontibacter virosus DSM 100231 Hymenobacteraceae
Pontibacter indicus LP100 Hymenobacteraceae
Crocinitomix catalasitica ATCC 23190 Crocinitomicaceae
Crocinitomix algicola 0182T Crocinitomicaceae
Fluviicola taffensis DSM 16823 Crocinitomicaceae
Brumimicrobium mesophilum JCM 14063 Crocinitomicaceae
Lishizhenia tianjinensis CGMCC 1.7005 Crocinitomicaceae
Flavobacterium aciduliphilum DSM 25663 Flavobacteriaceae
Flavobacterium fontis DSM 25660 Flavobacteriaceae
Flavobacterium haoranii DSM 22807 Flavobacteriaceae
Flavobacterium indicum GPTSA100-9 Flavobacteriaceae
Flavobacterium beibuense F44-8 Flavobacteriaceae
Flavobacterium akiainvivens IK-1 Flavobacteriaceae
Flavobacterium akiainvivens DSM 25510 Flavobacteriaceae
Flavobacterium subsaxonicum DSM 21790 Flavobacteriaceae
Flavobacterium rivuli DSM 21788 Flavobacteriaceae
Flavobacterium suncheonense DSM 17707 Flavobacteriaceae
Flavobacterium limnosediminis KACC 16937 Flavobacteriaceae
Flavobacterium saliperosum S13 Flavobacteriaceae
Flavobacterium cauense NBRC 104929 Flavobacteriaceae
Flavobacterium enshiense KCTC 23775 Flavobacteriaceae
Flavobacterium noncentrifugens CGMCC 1.10076 Flavobacteriaceae
Flavobacterium endophyticum DSM 29537 Flavobacteriaceae
Flavobacterium lindanitolerans DSM 21886 Flavobacteriaceae
Flavobacterium soli DSM 19725 Flavobacteriaceae
Flavobacterium hydatis DSM 2063 Flavobacteriaceae
Flavobacterium frigidimaris DSM 15937 Flavobacteriaceae
Flavobacterium piscis CCUG 60099 Flavobacteriaceae
Flavobacterium saccharophilum DSM 1811 Flavobacteriaceae
Flavobacterium aquidurense DSM 18293 Flavobacteriaceae
Flavobacterium pectinovorum DSM 6368 Flavobacteriaceae
Flavobacterium flevense DSM 1076 Flavobacteriaceae
Flavobacterium seoulense EM1321 Flavobacteriaceae
Flavobacterium commune PK15 Flavobacteriaceae
Flavobacterium daejeonense DSM 17708 Flavobacteriaceae
Flavobacterium glycines NBRC 105008 Flavobacteriaceae
Flavobacterium gilvum EM1308 Flavobacteriaceae
Flavobacterium branchiophilum ATCC 35035 Flavobacteriaceae
Flavobacterium aquicola DSM 100880 Flavobacteriaceae
Flavobacterium chungangense LMG 26729 Flavobacteriaceae
Flavobacterium chilense DSM 24724 Flavobacteriaceae
Flavobacterium chilense LMG 26360 Flavobacteriaceae
Flavobacterium defluvii DSM 17963 Flavobacteriaceae
Flexibacter aurantiacus DSM 6792 Cytophagaceae
Flavobacterium johnsoniae ATCC 17061 Flavobacteriaceae
Flavobacterium johnsoniae DSM 2064 Flavobacteriaceae
Flavobacterium resistens DSM 19382 Flavobacteriaceae
Flavobacterium cutihirudinis DSM 25795 Flavobacteriaceae
Flavobacterium denitrificans DSM 15936 Flavobacteriaceae
Flavobacterium anhuiense CGMCC 1.6859 Flavobacteriaceae
Flavobacterium nitrogenifigens DSM 29982 Flavobacteriaceae
Flavobacterium plurextorum CCUG 60112 Flavobacteriaceae
Flavobacterium spartansii ATCC BAA-2541 Flavobacteriaceae
Flavobacterium tructae CCUG 60100 Flavobacteriaceae
Flavobacterium oncorhynchi CCUG 59446 Flavobacteriaceae
Flavobacterium reichenbachii LMG 25512 Flavobacteriaceae
Flavobacterium hercynium DSM 18292 Flavobacteriaceae
Flavobacterium hibernum DSM 12611 Flavobacteriaceae
Flavobacterium araucananum DSM 24704 Flavobacteriaceae
Flavobacterium swingsii DSM 21789 Flavobacteriaceae
Flavobacterium xinjiangense CGMCC 1.2749 Flavobacteriaceae
Flavobacterium urumqiense CGMCC 1.9230 Flavobacteriaceae
Flavobacterium sinopsychrotolerans CGMCC 1.8704 Flavobacteriaceae
Flavobacterium glaciei DSM 19728 Flavobacteriaceae
Flavobacterium succinicans DSM 4002 Flavobacteriaceae
Flavobacterium fluvii DSM 19978 Flavobacteriaceae
Flavobacterium limicola DSM 15094 Flavobacteriaceae
Flavobacterium tegetincola DSM 22377 Flavobacteriaceae
Flavobacterium antarcticum DSM 19726 Flavobacteriaceae
Flavobacterium granuli DSM 19729 Flavobacteriaceae
Flavobacterium granuli DSM 17797 Flavobacteriaceae
Flavobacterium gillisiae DSM 22376 Flavobacteriaceae
Flavobacterium frigoris DSM 15719 Flavobacteriaceae
Flavobacterium crassostreae LPB0076 Flavobacteriaceae
Flavobacterium degerlachei DSM 15718 Flavobacteriaceae
Flavobacterium weaverense DSM 19727 Flavobacteriaceae
Flavobacterium micromati DSM 17659 Flavobacteriaceae
Flavobacterium segetis DSM 19741 Flavobacteriaceae
Flavobacterium fryxellicola DSM 16209 Flavobacteriaceae
Flavobacterium xanthum DSM 3661 Flavobacteriaceae
Flavobacterium fryxellicola DSM 16209 Flavobacteriaceae
Flavobacterium croceum DSM 17960 Flavobacteriaceae
Flavobacterium filum DSM 17961 Flavobacteriaceae
Flavobacterium terrae DSM 18829 Flavobacteriaceae
Flavobacterium aquatile LMG 4008 Flavobacteriaceae
Flavobacterium dankookense DSM 25687 Flavobacteriaceae
Flavobacterium urocaniciphilum DSM 27078 Flavobacteriaceae
Flavobacterium terrigena DSM 17934 Flavobacteriaceae
Flavobacterium cheniae DSM 22462 Flavobacteriaceae
Flavobacterium sasangense DSM 21067 Flavobacteriaceae
Flavobacterium cucumis DSM 18830 Flavobacteriaceae

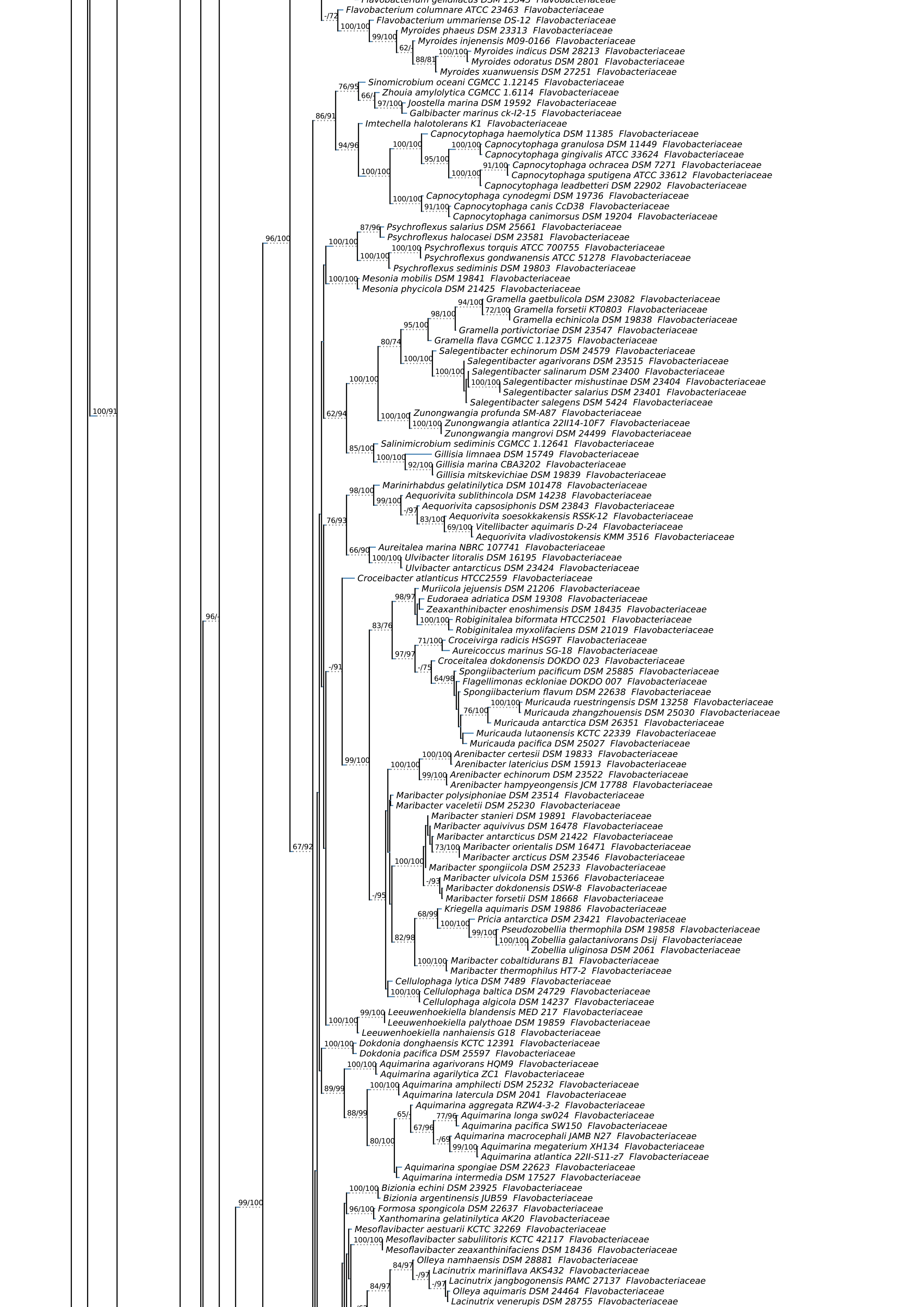
Flavobacterium columnare ATCC 23463 Flavobacteriaceae
Flavobacterium ummariense DS-12 Flavobacteriaceae
Myroides phaeus DSM 23313 Flavobacteriaceae
Myroides injenensis M09-0166 Flavobacteriaceae
Myroides indicus DSM 28213 Flavobacteriaceae
Myroides odoratus DSM 2801 Flavobacteriaceae
Myroides xuanwuensis DSM 27251 Flavobacteriaceae
Sinomicrobium oceani CGMCC 1.12145 Flavobacteriaceae
Zhouia amylolytica CGMCC 1.6114 Flavobacteriaceae
Joostella marina DSM 19592 Flavobacteriaceae
Galbibacter marinus ck-I2-15 Flavobacteriaceae
Imtechella halotolerans K1 Flavobacteriaceae
Capnocytophaga haemolytica DSM 11385 Flavobacteriaceae
Capnocytophaga granulosa DSM 11449 Flavobacteriaceae
Capnocytophaga gingivalis ATCC 33624 Flavobacteriaceae
Capnocytophaga ochracea DSM 7271 Flavobacteriaceae
Capnocytophaga sputigena ATCC 33612 Flavobacteriaceae
Capnocytophaga leadbetteri DSM 22902 Flavobacteriaceae
Capnocytophaga cynodegmi DSM 19736 Flavobacteriaceae
Capnocytophaga canis CcD38 Flavobacteriaceae
Capnocytophaga canimorsus DSM 19204 Flavobacteriaceae
Psychroflexus salarius DSM 25661 Flavobacteriaceae
Psychroflexus halocasei DSM 23581 Flavobacteriaceae
Psychroflexus torquis ATCC 700755 Flavobacteriaceae
Psychroflexus gondwanensis ATCC 51278 Flavobacteriaceae
Psychroflexus sediminis DSM 19803 Flavobacteriaceae
Mesonia mobilis DSM 19841 Flavobacteriaceae
Mesonia phycicola DSM 21425 Flavobacteriaceae
Gramella gaetbulicola DSM 23082 Flavobacteriaceae
Gramella forsetii KT0803 Flavobacteriaceae
Gramella echinicola DSM 19838 Flavobacteriaceae
Gramella portivictoriae DSM 23547 Flavobacteriaceae
Gramella flava CGMCC 1.12375 Flavobacteriaceae
Salegentibacter echinorum DSM 24579 Flavobacteriaceae
Salegentibacter agarivorans DSM 23515 Flavobacteriaceae
Salegentibacter salinarum DSM 23400 Flavobacteriaceae
Salegentibacter mishustinae DSM 23404 Flavobacteriaceae
Salegentibacter salarius DSM 23401 Flavobacteriaceae
Salegentibacter salegens DSM 5424 Flavobacteriaceae
Zunongwangia profunda SM-A87 Flavobacteriaceae
Zunongwangia atlantica 22II14-10F7 Flavobacteriaceae
Zunongwangia mangrovi DSM 24499 Flavobacteriaceae
Salinimicrobium sediminis CGMCC 1.12641 Flavobacteriaceae
Gillisia limnaea DSM 15749 Flavobacteriaceae
Gillisia marina CBA3202 Flavobacteriaceae
Gillisia mitskevichiae DSM 19839 Flavobacteriaceae
Marinirhabdus gelatinilytica DSM 101478 Flavobacteriaceae
Aequorivita sublithincola DSM 14238 Flavobacteriaceae
Aequorivita capsosiphonis DSM 23843 Flavobacteriaceae
Aequorivita soesokkakensis RSSK-12 Flavobacteriaceae
Vitellibacter aquimaris D-24 Flavobacteriaceae
Aequorivita vladivostokensis KMM 3516 Flavobacteriaceae
Aureitalea marina NBRC 107741 Flavobacteriaceae
Ulvibacter litoralis DSM 16195 Flavobacteriaceae
Ulvibacter antarcticus DSM 23424 Flavobacteriaceae
Croceibacter atlanticus HTCC2559 Flavobacteriaceae
Muriicola jejuensis DSM 21206 Flavobacteriaceae
Eudoraea adriatica DSM 19308 Flavobacteriaceae
Zeaxanthinibacter enoshimensis DSM 18435 Flavobacteriaceae
Robiginitalea biformata HTCC2501 Flavobacteriaceae
Robiginitalea myxolifaciens DSM 21019 Flavobacteriaceae
Croceivirga radicis HSG9T Flavobacteriaceae
Aureicoccus marinus SG-18 Flavobacteriaceae
Croceitalea dokdonensis DOKDO 023 Flavobacteriaceae
Spongiibacterium pacificum DSM 25885 Flavobacteriaceae
Flagellimonas eckloniae DOKDO 007 Flavobacteriaceae
Spongiibacterium flavum DSM 22638 Flavobacteriaceae
Muricauda ruestringensis DSM 13258 Flavobacteriaceae
Muricauda zhangzhouensis DSM 25030 Flavobacteriaceae
Muricauda antarctica DSM 26351 Flavobacteriaceae
Muricauda lutaonensis KCTC 22339 Flavobacteriaceae
Muricauda pacifica DSM 25027 Flavobacteriaceae
Arenibacter certesii DSM 19833 Flavobacteriaceae
Arenibacter latericius DSM 15913 Flavobacteriaceae
Arenibacter echinorum DSM 23522 Flavobacteriaceae
Arenibacter hampyeongensis JCM 17788 Flavobacteriaceae
Maribacter polysiphoniae DSM 23514 Flavobacteriaceae
Maribacter vaceletii DSM 25230 Flavobacteriaceae
Maribacter stanieri DSM 19891 Flavobacteriaceae
Maribacter aquivivus DSM 16478 Flavobacteriaceae
Maribacter antarcticus DSM 21422 Flavobacteriaceae
Maribacter orientalis DSM 16471 Flavobacteriaceae
Maribacter arcticus DSM 23546 Flavobacteriaceae
Maribacter spongiicola DSM 25233 Flavobacteriaceae
Maribacter ulvicola DSM 15366 Flavobacteriaceae
Maribacter dokdonensis DSW-8 Flavobacteriaceae
Maribacter forsetii DSM 18668 Flavobacteriaceae
Kriegella aquimaris DSM 19886 Flavobacteriaceae
Pricia antarctica DSM 23421 Flavobacteriaceae
Pseudozobellia thermophila DSM 19858 Flavobacteriaceae
Zobellia galactanivorans Dsij Flavobacteriaceae
Zobellia uliginosa DSM 2061 Flavobacteriaceae
Maribacter cobaltidurans B1 Flavobacteriaceae
Maribacter thermophilus HT7-2 Flavobacteriaceae
Cellulophaga lytica DSM 7489 Flavobacteriaceae
Cellulophaga baltica DSM 24729 Flavobacteriaceae
Cellulophaga algicola DSM 14237 Flavobacteriaceae
Leeuwenhoekiella blandensis MED 217 Flavobacteriaceae
Leeuwenhoekiella palythoae DSM 19859 Flavobacteriaceae
Leeuwenhoekiella nanhaiensis G18 Flavobacteriaceae
Dokdonia donghaensis KCTC 12391 Flavobacteriaceae
Dokdonia pacifica DSM 25597 Flavobacteriaceae
Aquimarina agarivorans HQM9 Flavobacteriaceae
Aquimarina agarilytica ZC1 Flavobacteriaceae
Aquimarina amphilecti DSM 25232 Flavobacteriaceae
Aquimarina latercula DSM 2041 Flavobacteriaceae
Aquimarina aggregata RZW4-3-2 Flavobacteriaceae
Aquimarina longa sw024 Flavobacteriaceae
Aquimarina pacifica SW150 Flavobacteriaceae
Aquimarina macrocephali JAMB N27 Flavobacteriaceae
Aquimarina megaterium XH134 Flavobacteriaceae
Aquimarina atlantica 22II-S11-z7 Flavobacteriaceae
Aquimarina spongiae DSM 22623 Flavobacteriaceae
Aquimarina intermedia DSM 17527 Flavobacteriaceae
Bizionia echini DSM 23925 Flavobacteriaceae
Bizionia argentinensis JUB59 Flavobacteriaceae
Formosa spongicola DSM 22637 Flavobacteriaceae
Xanthomarina gelatinilytica AK20 Flavobacteriaceae
Mesoflavibacter aestuarii KCTC 32269 Flavobacteriaceae
Mesoflavibacter sabulilitoris KCTC 42117 Flavobacteriaceae
Mesoflavibacter zeaxanthinifaciens DSM 18436 Flavobacteriaceae
Olleya namhaensis DSM 28881 Flavobacteriaceae
Lacinutrix mariniflava AKS432 Flavobacteriaceae
Lacinutrix jangbogonensis PAMC 27137 Flavobacteriaceae
Olleya aquimaris DSM 24464 Flavobacteriaceae
Lacinutrix venerupis DSM 28755 Flavobacteriaceae

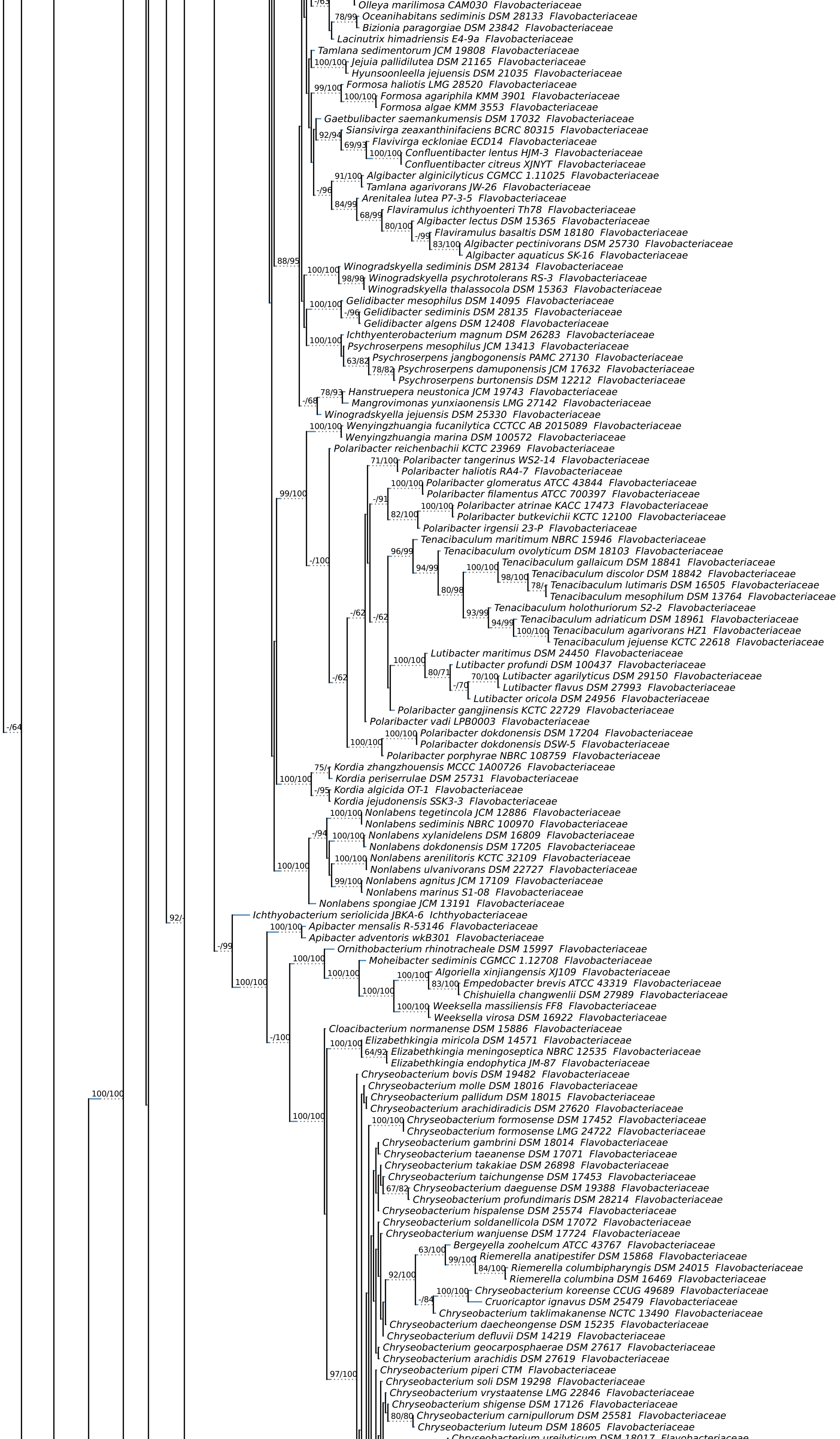

Olleya marilimosa CAM030 Flavobacteriaceae
Oceanihabitans sediminis DSM 28133 Flavobacteriaceae
Bizionia paragorgiae DSM 23842 Flavobacteriaceae
Lacinutrix himadriensis E4-9a Flavobacteriaceae
Tamlana sedimentorum JCM 19808 Flavobacteriaceae
Jejuia pallidilutea DSM 21165 Flavobacteriaceae
Hyunsoonleella jejuensis DSM 21035 Flavobacteriaceae
Formosa haliotis LMG 28520 Flavobacteriaceae
Formosa agariphila KMM 3901 Flavobacteriaceae
Formosa algae KMM 3553 Flavobacteriaceae
Gaetbulibacter saemankumensis DSM 17032 Flavobacteriaceae
Siansivirga zeaxanthinifaciens BCRC 80315 Flavobacteriaceae
Flavivirga eckloniae ECD14 Flavobacteriaceae
Confluentibacter lentus HJM-3 Flavobacteriaceae
Confluentibacter citreus XJNYT Flavobacteriaceae
Algibacter alginicilyticus CGMCC 1.11025 Flavobacteriaceae
Tamlana agarivorans JW-26 Flavobacteriaceae
Arenitalea lutea P7-3-5 Flavobacteriaceae
Flaviramulus ichthyoenteri Th78 Flavobacteriaceae
Algibacter lectus DSM 15365 Flavobacteriaceae
Flaviramulus basaltis DSM 18180 Flavobacteriaceae
Algibacter pectinivorans DSM 25730 Flavobacteriaceae
Algibacter aquaticus SK-16 Flavobacteriaceae
Winogradskyella sediminis DSM 28134 Flavobacteriaceae
Winogradskyella psychrotolerans RS-3 Flavobacteriaceae
Winogradskyella thalassocola DSM 15363 Flavobacteriaceae
Gelidibacter mesophilus DSM 14095 Flavobacteriaceae
Gelidibacter sediminis DSM 28135 Flavobacteriaceae
Gelidibacter algens DSM 12408 Flavobacteriaceae
Ichthyenterobacterium magnum DSM 26283 Flavobacteriaceae
Psychroserpens mesophilus JCM 13413 Flavobacteriaceae
Psychroserpens jangbogonensis PAMC 27130 Flavobacteriaceae
Psychroserpens damuponensis JCM 17632 Flavobacteriaceae
Psychroserpens burtonensis DSM 12212 Flavobacteriaceae
Hanstruepera neustonica JCM 19743 Flavobacteriaceae
Mangrovimonas yunxiaonensis LMG 27142 Flavobacteriaceae
Winogradskyella jejuensis DSM 25330 Flavobacteriaceae
Wenyingzhuangia fucanilytica CCTCC AB 2015089 Flavobacteriaceae
Wenyingzhuangia marina DSM 100572 Flavobacteriaceae
Polaribacter reichenbachii KCTC 23969 Flavobacteriaceae
Polaribacter tangerinus WS2-14 Flavobacteriaceae
Polaribacter haliotis RA4-7 Flavobacteriaceae
Polaribacter glomeratus ATCC 43844 Flavobacteriaceae
Polaribacter filamentus ATCC 700397 Flavobacteriaceae
Polaribacter atrinae KACC 17473 Flavobacteriaceae
Polaribacter butkevichii KCTC 12100 Flavobacteriaceae
Polaribacter irgensii 23-P Flavobacteriaceae
Tenacibaculum maritimum NBRC 15946 Flavobacteriaceae
Tenacibaculum ovolyticum DSM 18103 Flavobacteriaceae
Tenacibaculum gallaicum DSM 18841 Flavobacteriaceae
Tenacibaculum discolor DSM 18842 Flavobacteriaceae
Tenacibaculum lutimaris DSM 16505 Flavobacteriaceae
Tenacibaculum mesophilum DSM 13764 Flavobacteriaceae
Tenacibaculum holothuriorum S2-2 Flavobacteriaceae
Tenacibaculum adriaticum DSM 18961 Flavobacteriaceae
Tenacibaculum agarivorans HZ1 Flavobacteriaceae
Tenacibaculum jejuense KCTC 22618 Flavobacteriaceae
Lutibacter maritimus DSM 24450 Flavobacteriaceae
Lutibacter profundi DSM 100437 Flavobacteriaceae
Lutibacter agarilyticus DSM 29150 Flavobacteriaceae
Lutibacter flavus DSM 27993 Flavobacteriaceae
Lutibacter oricola DSM 24956 Flavobacteriaceae
Polaribacter gangjinensis KCTC 22729 Flavobacteriaceae
Polaribacter vadi LPB0003 Flavobacteriaceae
Polaribacter dokdonensis DSM 17204 Flavobacteriaceae
Polaribacter dokdonensis DSW-5 Flavobacteriaceae
Polaribacter porphyrae NBRC 108759 Flavobacteriaceae
Kordia zhangzhouensis MCCC 1A00726 Flavobacteriaceae
Kordia periserrulae DSM 25731 Flavobacteriaceae
Kordia algicida OT-1 Flavobacteriaceae
Kordia jejudonensis SSK3-3 Flavobacteriaceae
Nonlabens tegetincola JCM 12886 Flavobacteriaceae
Nonlabens sediminis NBRC 100970 Flavobacteriaceae
Nonlabens xylanidelens DSM 16809 Flavobacteriaceae
Nonlabens dokdonensis DSM 17205 Flavobacteriaceae
Nonlabens arenilitoris KCTC 32109 Flavobacteriaceae
Nonlabens ulvanivorans DSM 22727 Flavobacteriaceae
Nonlabens agnitus JCM 17109 Flavobacteriaceae
Nonlabens marinus S1-08 Flavobacteriaceae
Nonlabens spongiae JCM 13191 Flavobacteriaceae
Ichthyobacterium seriolicida JBKA-6 Ichthyobacteriaceae
Apibacter mensalis R-53146 Flavobacteriaceae
Apibacter adventoris wkB301 Flavobacteriaceae
Ornithobacterium rhinotracheale DSM 15997 Flavobacteriaceae
Moheibacter sediminis CGMCC 1.12708 Flavobacteriaceae
Algoriella xinjiangensis XJ109 Flavobacteriaceae
Empedobacter brevis ATCC 43319 Flavobacteriaceae
Chishuiella changwenlii DSM 27989 Flavobacteriaceae
Weeksella massiliensis FF8 Flavobacteriaceae
Weeksella virosa DSM 16922 Flavobacteriaceae
Cloacibacterium normanense DSM 15886 Flavobacteriaceae
Elizabethkingia miricola DSM 14571 Flavobacteriaceae
Elizabethkingia meningoseptica NBRC 12535 Flavobacteriaceae
Elizabethkingia endophytica JM-87 Flavobacteriaceae
Chryseobacterium bovis DSM 19482 Flavobacteriaceae
Chryseobacterium molle DSM 18016 Flavobacteriaceae
Chryseobacterium pallidum DSM 18015 Flavobacteriaceae
Chryseobacterium arachidiradicis DSM 27620 Flavobacteriaceae
Chryseobacterium formosense DSM 17452 Flavobacteriaceae
Chryseobacterium formosense LMG 24722 Flavobacteriaceae
Chryseobacterium gambrini DSM 18014 Flavobacteriaceae
Chryseobacterium taeanense DSM 17071 Flavobacteriaceae
Chryseobacterium takakiae DSM 26898 Flavobacteriaceae
Chryseobacterium taichungense DSM 17453 Flavobacteriaceae
Chryseobacterium daeguense DSM 19388 Flavobacteriaceae
Chryseobacterium profundimaris DSM 28214 Flavobacteriaceae
Chryseobacterium hispalense DSM 25574 Flavobacteriaceae
Chryseobacterium soldanellicola DSM 17072 Flavobacteriaceae
Chryseobacterium wanjuense DSM 17724 Flavobacteriaceae
Bergeyella zoohelcum ATCC 43767 Flavobacteriaceae
Riemerella anatipestifer DSM 15868 Flavobacteriaceae
Riemerella columbipharyngis DSM 24015 Flavobacteriaceae
Riemerella columbina DSM 16469 Flavobacteriaceae
Chryseobacterium koreense CCUG 49689 Flavobacteriaceae
Cruoricaptor ignavus DSM 25479 Flavobacteriaceae
Chryseobacterium taklimakanense NCTC 13490 Flavobacteriaceae
Chryseobacterium daecheongense DSM 15235 Flavobacteriaceae
Chryseobacterium defluvii DSM 14219 Flavobacteriaceae
Chryseobacterium geocarposphaerae DSM 27617 Flavobacteriaceae
Chryseobacterium arachidis DSM 27619 Flavobacteriaceae
Chryseobacterium piperi CTM Flavobacteriaceae
Chryseobacterium soli DSM 19298 Flavobacteriaceae
Chryseobacterium vrystaatense LMG 22846 Flavobacteriaceae
Chryseobacterium shigense DSM 17126 Flavobacteriaceae
Chryseobacterium carnipullorum DSM 25581 Flavobacteriaceae
Chryseobacterium luteum DSM 18605 Flavobacteriaceae
-/63
78/99
100/100
99/100
100/100
92/94
69/93
100/100
91/100
-/96
84/99
68/99
80/100
-/99
83/100
88/95
100/100
98/98
100/100
-/96
100/100
63/82
78/82
78/93
-/68
100/100
71/100
100/100
99/100
-/91
100/100
82/100
96/99
94/99
100/100
98/100
78/-
80/98
-/100
93/99
94/99
100/100
-/62
-/62
100/100
80/71
70/100
-/70
-/62
-/64
100/100
100/100
75/-
100/100
-/95
100/100
-/94
100/100
100/100
100/100
99/100
92/-
-/99
100/100
100/100
100/100
100/100
100/100
100/100
83/100
100/100
-/100
100/100
64/92
100/100
100/100
100/100
67/82
63/100
99/100
84/100
92/100
100/100
-/84
97/100
80/80

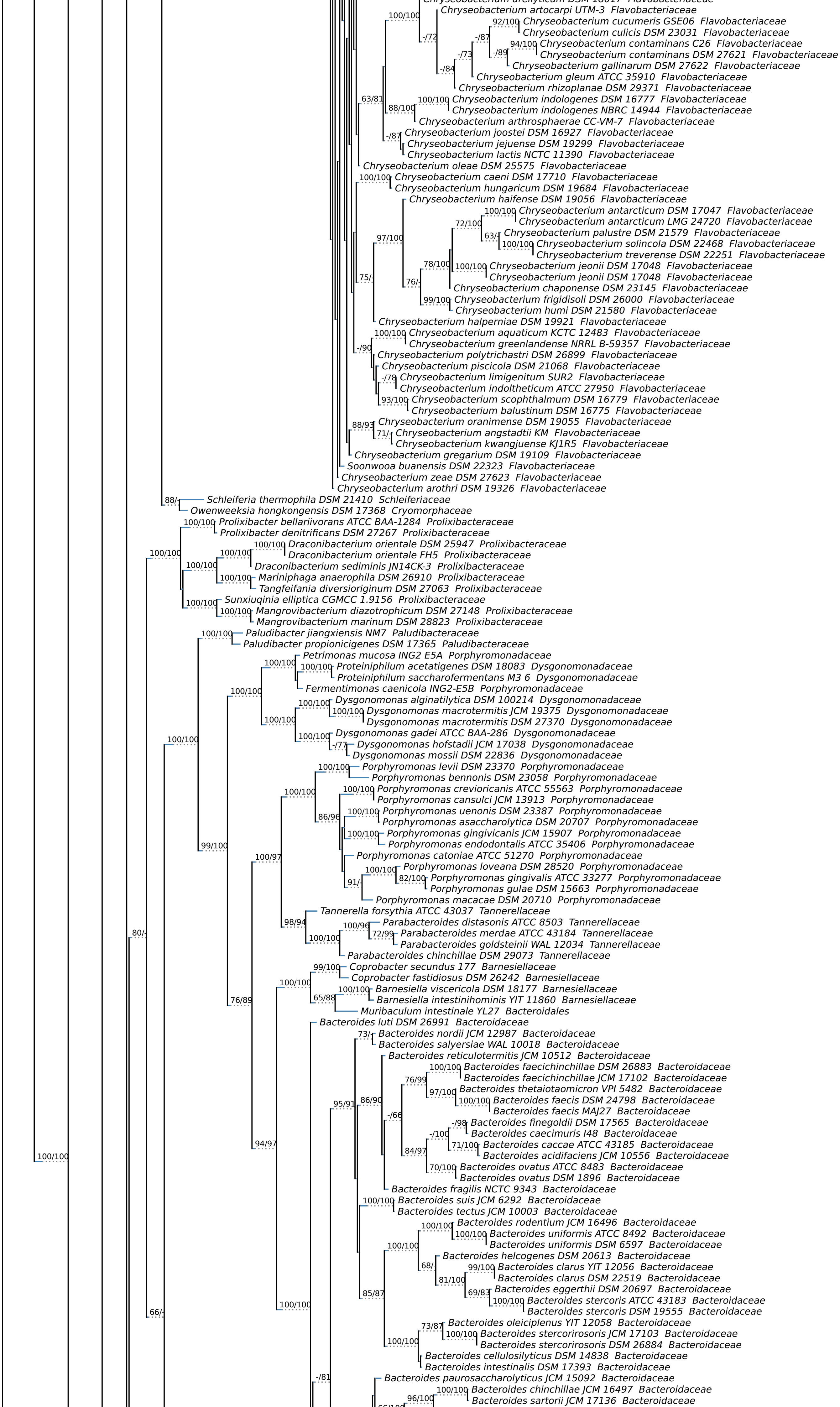
Chryseobacterium artocarpi UTM-3 Flavobacteriaceae
Chryseobacterium cucumeris GSE06 Flavobacteriaceae
Chryseobacterium culicis DSM 23031 Flavobacteriaceae
Chryseobacterium contaminans C26 Flavobacteriaceae
Chryseobacterium contaminans DSM 27621 Flavobacteriaceae
Chryseobacterium gallinarum DSM 27622 Flavobacteriaceae
Chryseobacterium gleum ATCC 35910 Flavobacteriaceae
Chryseobacterium rhizoplanae DSM 29371 Flavobacteriaceae
Chryseobacterium indologenes DSM 16777 Flavobacteriaceae
Chryseobacterium indologenes NBRC 14944 Flavobacteriaceae
Chryseobacterium arthrosphaerae CC-VM-7 Flavobacteriaceae
Chryseobacterium joostei DSM 16927 Flavobacteriaceae
Chryseobacterium jejuense DSM 19299 Flavobacteriaceae
Chryseobacterium lactis NCTC 11390 Flavobacteriaceae
Chryseobacterium oleae DSM 25575 Flavobacteriaceae
Chryseobacterium caeni DSM 17710 Flavobacteriaceae
Chryseobacterium hungaricum DSM 19684 Flavobacteriaceae
Chryseobacterium haifense DSM 19056 Flavobacteriaceae
Chryseobacterium antarcticum DSM 17047 Flavobacteriaceae
Chryseobacterium antarcticum LMG 24720 Flavobacteriaceae
Chryseobacterium palustre DSM 21579 Flavobacteriaceae
Chryseobacterium solincola DSM 22468 Flavobacteriaceae
Chryseobacterium treverense DSM 22251 Flavobacteriaceae
Chryseobacterium jeonii DSM 17048 Flavobacteriaceae
Chryseobacterium jeonii DSM 17048 Flavobacteriaceae
Chryseobacterium chaponense DSM 23145 Flavobacteriaceae
Chryseobacterium frigidisoli DSM 26000 Flavobacteriaceae
Chryseobacterium humi DSM 21580 Flavobacteriaceae
Chryseobacterium halperniae DSM 19921 Flavobacteriaceae
Chryseobacterium aquaticum KCTC 12483 Flavobacteriaceae
Chryseobacterium greenlandense NRRL B-59357 Flavobacteriaceae
Chryseobacterium polytrichastri DSM 26899 Flavobacteriaceae
Chryseobacterium piscicola DSM 21068 Flavobacteriaceae
Chryseobacterium limigenitum SUR2 Flavobacteriaceae
Chryseobacterium indoltheticum ATCC 27950 Flavobacteriaceae
Chryseobacterium scophthalmum DSM 16779 Flavobacteriaceae
Chryseobacterium balustinum DSM 16775 Flavobacteriaceae
Chryseobacterium oranimense DSM 19055 Flavobacteriaceae
Chryseobacterium angstadtii KM Flavobacteriaceae
Chryseobacterium kwangjuense KJ1R5 Flavobacteriaceae
Chryseobacterium gregarium DSM 19109 Flavobacteriaceae
Soonwooa buanensis DSM 22323 Flavobacteriaceae
Chryseobacterium zeae DSM 27623 Flavobacteriaceae
Chryseobacterium arothri DSM 19326 Flavobacteriaceae
Schleiferia thermophila DSM 21410 Schleiferiaceae
Owenweeksia hongkongensis DSM 17368 Cryomorphaceae
Prolixibacter bellariivorans ATCC BAA-1284 Prolixibacteraceae
Prolixibacter denitrificans DSM 27267 Prolixibacteraceae
Draconibacterium orientale DSM 25947 Prolixibacteraceae
Draconibacterium orientale FH5 Prolixibacteraceae
Draconibacterium sediminis JN14CK-3 Prolixibacteraceae
Mariniphaga anaerophila DSM 26910 Prolixibacteraceae
Tangfeifania diversioriginum DSM 27063 Prolixibacteraceae
Sunxiuqinia elliptica CGMCC 1.9156 Prolixibacteraceae
Mangrovibacterium diazotrophicum DSM 27148 Prolixibacteraceae
Mangrovibacterium marinum DSM 28823 Prolixibacteraceae
Paludibacter jiangxiensis NM7 Paludibacteraceae
Paludibacter propionicigenes DSM 17365 Paludibacteraceae
Petrimonas mucosa ING2 E5A Porphyromonadaceae
Proteiniphilum acetatigenes DSM 18083 Dysgonomonadaceae
Proteiniphilum saccharofermentans M3 6 Dysgonomonadaceae
Fermentimonas caenicola ING2-E5B Porphyromonadaceae
Dysgonomonas alginatilytica DSM 100214 Dysgonomonadaceae
Dysgonomonas macrotermitis JCM 19375 Dysgonomonadaceae
Dysgonomonas macrotermitis DSM 27370 Dysgonomonadaceae
Dysgonomonas gadei ATCC BAA-286 Dysgonomonadaceae
Dysgonomonas hofstadii JCM 17038 Dysgonomonadaceae
Dysgonomonas mossii DSM 22836 Dysgonomonadaceae
Porphyromonas levii DSM 23370 Porphyromonadaceae
Porphyromonas bennonis DSM 23058 Porphyromonadaceae
Porphyromonas crevioricanis ATCC 55563 Porphyromonadaceae
Porphyromonas cansulci JCM 13913 Porphyromonadaceae
Porphyromonas uenonis DSM 23387 Porphyromonadaceae
Porphyromonas asaccharolytica DSM 20707 Porphyromonadaceae
Porphyromonas gingivicanis JCM 15907 Porphyromonadaceae
Porphyromonas endodontalis ATCC 35406 Porphyromonadaceae
Porphyromonas catoniae ATCC 51270 Porphyromonadaceae
Porphyromonas loveana DSM 28520 Porphyromonadaceae
Porphyromonas gingivalis ATCC 33277 Porphyromonadaceae
Porphyromonas gulae DSM 15663 Porphyromonadaceae
Porphyromonas macacae DSM 20710 Porphyromonadaceae
Tannerella forsythia ATCC 43037 Tannerellaceae
Parabacteroides distasonis ATCC 8503 Tannerellaceae
Parabacteroides merdae ATCC 43184 Tannerellaceae
Parabacteroides goldsteinii WAL 12034 Tannerellaceae
Parabacteroides chinchillae DSM 29073 Tannerellaceae
Coprobacter secundus 177 Barnesiellaceae
Coprobacter fastidiosus DSM 26242 Barnesiellaceae
Barnesiella viscericola DSM 18177 Barnesiellaceae
Barnesiella intestinihominis YIT 11860 Barnesiellaceae
Muribaculum intestinale YL27 Bacteroidales
Bacteroides luti DSM 26991 Bacteroidaceae
Bacteroides nordii JCM 12987 Bacteroidaceae
Bacteroides salyersiae WAL 10018 Bacteroidaceae
Bacteroides reticulotermitis JCM 10512 Bacteroidaceae
Bacteroides faecichinchillae DSM 26883 Bacteroidaceae
Bacteroides faecichinchillae JCM 17102 Bacteroidaceae
Bacteroides thetaiotaomicron VPI 5482 Bacteroidaceae
Bacteroides faecis DSM 24798 Bacteroidaceae
Bacteroides faecis MAJ27 Bacteroidaceae
Bacteroides finegoldii DSM 17565 Bacteroidaceae
Bacteroides caecimuris I48 Bacteroidaceae
Bacteroides caccae ATCC 43185 Bacteroidaceae
Bacteroides acidifaciens JCM 10556 Bacteroidaceae
Bacteroides ovatus ATCC 8483 Bacteroidaceae
Bacteroides ovatus DSM 1896 Bacteroidaceae
Bacteroides fragilis NCTC 9343 Bacteroidaceae
Bacteroides suis JCM 6292 Bacteroidaceae
Bacteroides tectus JCM 10003 Bacteroidaceae
Bacteroides rodentium JCM 16496 Bacteroidaceae
Bacteroides uniformis ATCC 8492 Bacteroidaceae
Bacteroides uniformis DSM 6597 Bacteroidaceae
Bacteroides helcogenes DSM 20613 Bacteroidaceae
Bacteroides clarus YIT 12056 Bacteroidaceae
Bacteroides clarus DSM 22519 Bacteroidaceae
Bacteroides eggerthii DSM 20697 Bacteroidaceae
Bacteroides stercoris ATCC 43183 Bacteroidaceae
Bacteroides stercoris DSM 19555 Bacteroidaceae
Bacteroides oleiciplenus YIT 12058 Bacteroidaceae
Bacteroides stercorirosoris JCM 17103 Bacteroidaceae
Bacteroides stercorirosoris DSM 26884 Bacteroidaceae
Bacteroides cellulosilyticus DSM 14838 Bacteroidaceae
Bacteroides intestinalis DSM 17393 Bacteroidaceae
Bacteroides paurosaccharolyticus JCM 15092 Bacteroidaceae
Bacteroides chinchillae JCM 16497 Bacteroidaceae
Bacteroides sartorii JCM 17136 Bacteroidaceae

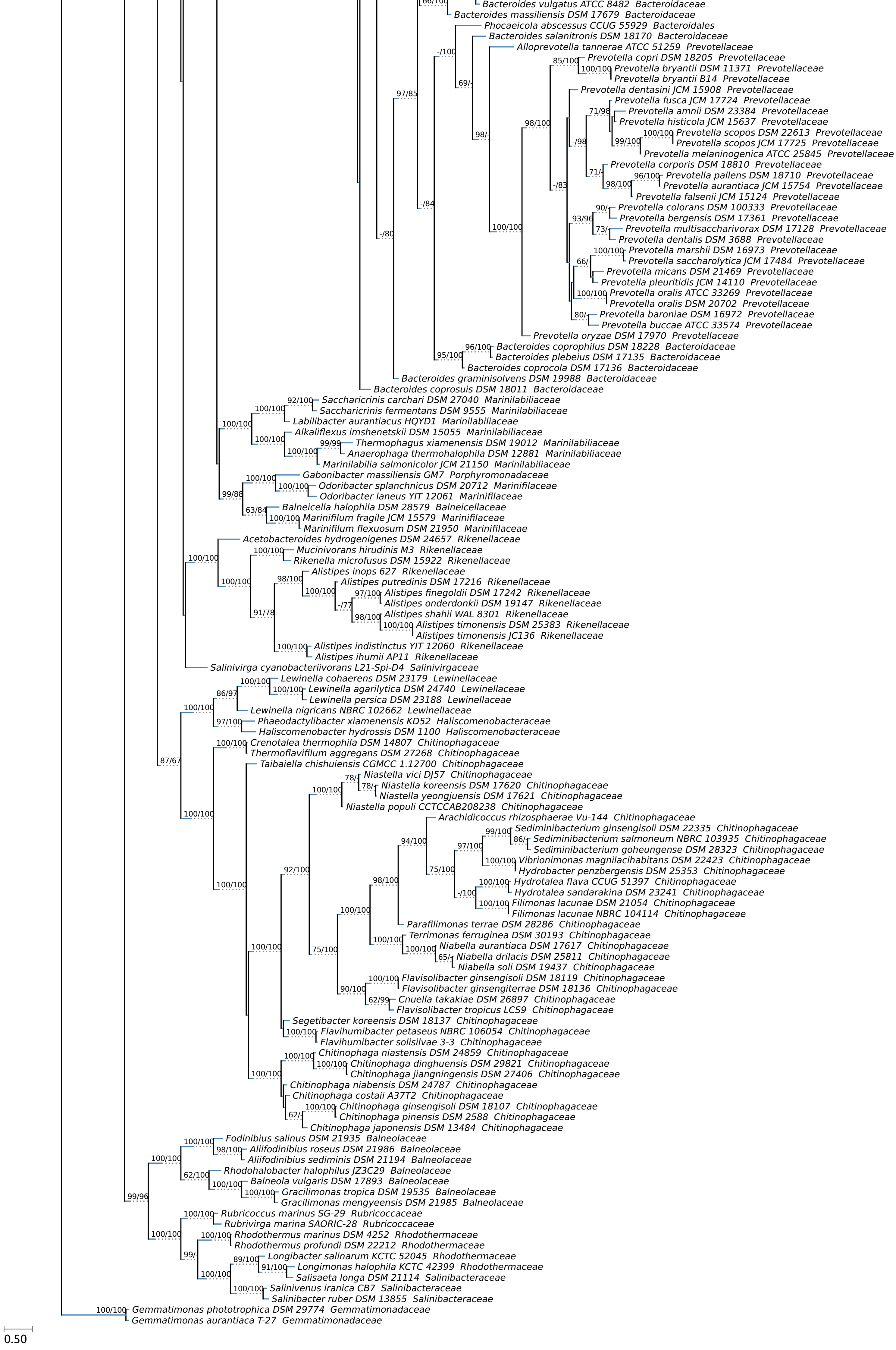
Bacteroides vulgatus ATCC 8482 Bacteroidaceae
Bacteroides massiliensis DSM 17679 Bacteroidaceae
Phocaeicola abscessus CCUG 55929 Bacteroidales
Bacteroides salanitronis DSM 18170 Bacteroidaceae
Alloprevotella tannerae ATCC 51259 Prevotellaceae
Prevotella copri DSM 18205 Prevotellaceae
Prevotella bryantii DSM 11371 Prevotellaceae
Prevotella bryantii B14 Prevotellaceae
Prevotella dentasini JCM 15908 Prevotellaceae
Prevotella fusca JCM 17724 Prevotellaceae
Prevotella amnii DSM 23384 Prevotellaceae
Prevotella histicola JCM 15637 Prevotellaceae
Prevotella scopos DSM 22613 Prevotellaceae
Prevotella scopos JCM 17725 Prevotellaceae
Prevotella melaninogenica ATCC 25845 Prevotellaceae
Prevotella corporis DSM 18810 Prevotellaceae
Prevotella pallens DSM 18710 Prevotellaceae
Prevotella aurantiaca JCM 15754 Prevotellaceae
Prevotella falsenii JCM 15124 Prevotellaceae
Prevotella colorans DSM 100333 Prevotellaceae
Prevotella bergensis DSM 17361 Prevotellaceae
Prevotella multisaccharivorax DSM 17128 Prevotellaceae
Prevotella dentalis DSM 3688 Prevotellaceae
Prevotella marshii DSM 16973 Prevotellaceae
Prevotella saccharolytica JCM 17484 Prevotellaceae
Prevotella micans DSM 21469 Prevotellaceae
Prevotella pleuritidis JCM 14110 Prevotellaceae
Prevotella oralis ATCC 33269 Prevotellaceae
Prevotella oralis DSM 20702 Prevotellaceae
Prevotella baroniae DSM 16972 Prevotellaceae
Prevotella buccae ATCC 33574 Prevotellaceae
Prevotella oryzae DSM 17970 Prevotellaceae
Bacteroides coprophilus DSM 18228 Bacteroidaceae
Bacteroides plebeius DSM 17135 Bacteroidaceae
Bacteroides coprocola DSM 17136 Bacteroidaceae
Bacteroides graminisolvens DSM 19988 Bacteroidaceae
Bacteroides coprosuis DSM 18011 Bacteroidaceae
Saccharicrinis carchari DSM 27040 Marinilabiliaceae
Saccharicrinis fermentans DSM 9555 Marinilabiliaceae
Labilibacter aurantiacus HQYD1 Marinilabiliaceae
Alkaliflexus imshenetskii DSM 15055 Marinilabiliaceae
Thermophagus xiamenensis DSM 19012 Marinilabiliaceae
Anaerophaga thermohalophila DSM 12881 Marinilabiliaceae
Marinilabilia salmonicolor JCM 21150 Marinilabiliaceae
Gabonibacter massiliensis GM7 Porphyromonadaceae
Odoribacter splanchnicus DSM 20712 Marinifilaceae
Odoribacter laneus YIT 12061 Marinifilaceae
Balneicella halophila DSM 28579 Balneicellaceae
Marinifilum fragile JCM 15579 Marinifilaceae
Marinifilum flexuosum DSM 21950 Marinifilaceae
Acetobacteroides hydrogenigenes DSM 24657 Rikenellaceae
Mucinivorans hirudinis M3 Rikenellaceae
Rikenella microfusus DSM 15922 Rikenellaceae
Alistipes inops 627 Rikenellaceae
Alistipes putredinis DSM 17216 Rikenellaceae
Alistipes finegoldii DSM 17242 Rikenellaceae
Alistipes onderdonkii DSM 19147 Rikenellaceae
Alistipes shahii WAL 8301 Rikenellaceae
Alistipes timonensis DSM 25383 Rikenellaceae
Alistipes timonensis JC136 Rikenellaceae
Alistipes indistinctus YIT 12060 Rikenellaceae
Alistipes ihumii AP11 Rikenellaceae
Salinivirga cyanobacteriivorans L21-Spi-D4 Salinivirgaceae
Lewinella cohaerens DSM 23179 Lewinellaceae
Lewinella agarilytica DSM 24740 Lewinellaceae
Lewinella persica DSM 23188 Lewinellaceae
Lewinella nigricans NBRC 102662 Lewinellaceae
Phaeodactylibacter xiamenensis KD52 Haliscomenobacteraceae
Haliscomenobacter hydrossis DSM 1100 Haliscomenobacteraceae
Crenotalea thermophila DSM 14807 Chitinophagaceae
Thermoflavifilum aggregans DSM 27268 Chitinophagaceae
Taibaiella chishuiensis CGMCC 1.12700 Chitinophagaceae
Niastella vici DJ57 Chitinophagaceae
Niastella koreensis DSM 17620 Chitinophagaceae
Niastella yeongjuensis DSM 17621 Chitinophagaceae
Niastella populi CCTCCAB208238 Chitinophagaceae
Arachidicoccus rhizosphaerae Vu-144 Chitinophagaceae
Sediminibacterium ginsengisoli DSM 22335 Chitinophagaceae
Sediminibacterium salmoneum NBRC 103935 Chitinophagaceae
Sediminibacterium goheungense DSM 28323 Chitinophagaceae
Vibrionimonas magnilacihabitans DSM 22423 Chitinophagaceae
Hydrobacter penzbergensis DSM 25353 Chitinophagaceae
Hydrotalea flava CCUG 51397 Chitinophagaceae
Hydrotalea sandarakina DSM 23241 Chitinophagaceae
Filimonas lacunae DSM 21054 Chitinophagaceae
Filimonas lacunae NBRC 104114 Chitinophagaceae
Parafilimonas terrae DSM 28286 Chitinophagaceae
Terrimonas ferruginea DSM 30193 Chitinophagaceae
Niabella aurantiaca DSM 17617 Chitinophagaceae
Niabella drilacis DSM 25811 Chitinophagaceae
Niabella soli DSM 19437 Chitinophagaceae
Flavisolibacter ginsengisoli DSM 18119 Chitinophagaceae
Flavisolibacter ginsengiterrae DSM 18136 Chitinophagaceae
Cnuella takakiae DSM 26897 Chitinophagaceae
Flavisolibacter tropicus LCS9 Chitinophagaceae
Segetibacter koreensis DSM 18137 Chitinophagaceae
Flavihumibacter petaseus NBRC 106054 Chitinophagaceae
Flavihumibacter solisilvae 3-3 Chitinophagaceae
Chitinophaga niastensis DSM 24859 Chitinophagaceae
Chitinophaga dinghuensis DSM 29821 Chitinophagaceae
Chitinophaga jiangningensis DSM 27406 Chitinophagaceae
Chitinophaga niabensis DSM 24787 Chitinophagaceae
Chitinophaga costaii A37T2 Chitinophagaceae
Chitinophaga ginsengisoli DSM 18107 Chitinophagaceae
Chitinophaga pinensis DSM 2588 Chitinophagaceae
Chitinophaga japonensis DSM 13484 Chitinophagaceae
Fodinibius salinus DSM 21935 Balneolaceae
Aliifodinibius roseus DSM 21986 Balneolaceae
Aliifodinibius sediminis DSM 21194 Balneolaceae
Rhodohalobacter halophilus JZ3C29 Balneolaceae
Balneola vulgaris DSM 17893 Balneolaceae
Gracilimonas tropica DSM 19535 Balneolaceae
Gracilimonas mengyeensis DSM 21985 Balneolaceae
Rubricoccus marinus SG-29 Rubricoccaceae
Rubrivirga marina SAORIC-28 Rubricoccaceae
Rhodothermus marinus DSM 4252 Rhodothermaceae
Rhodothermus profundi DSM 22212 Rhodothermaceae
Longibacter salinarum KCTC 52045 Rhodothermaceae
Longimonas halophila KCTC 42399 Rhodothermaceae
Salisaeta longa DSM 21114 Salinibacteraceae
Salinivenus iranica CB7 Salinibacteraceae
Salinibacter ruber DSM 13855 Salinibacteraceae
Gemmatimonas phototrophica DSM 29774 Gemmatimonadaceae
Gemmatimonas aurantiaca T-27 Gemmatimonadaceae
0.50

Figure 5: Unconstrained 23S rRNA gene ML tree (ULT) of *Bacteroidetes* inferred under the GTR+CAT model. The branches are scaled in terms of the expected number of substitutions per site. The numbers above the branches are support values when larger than 60% from ML (left) and MP (right) bootstrapping. Dotted parts of branches are filled in to allow proper placement of bootstrap values and are not part of the actual branch length. Each tip label ends with the family of the respective taxon.

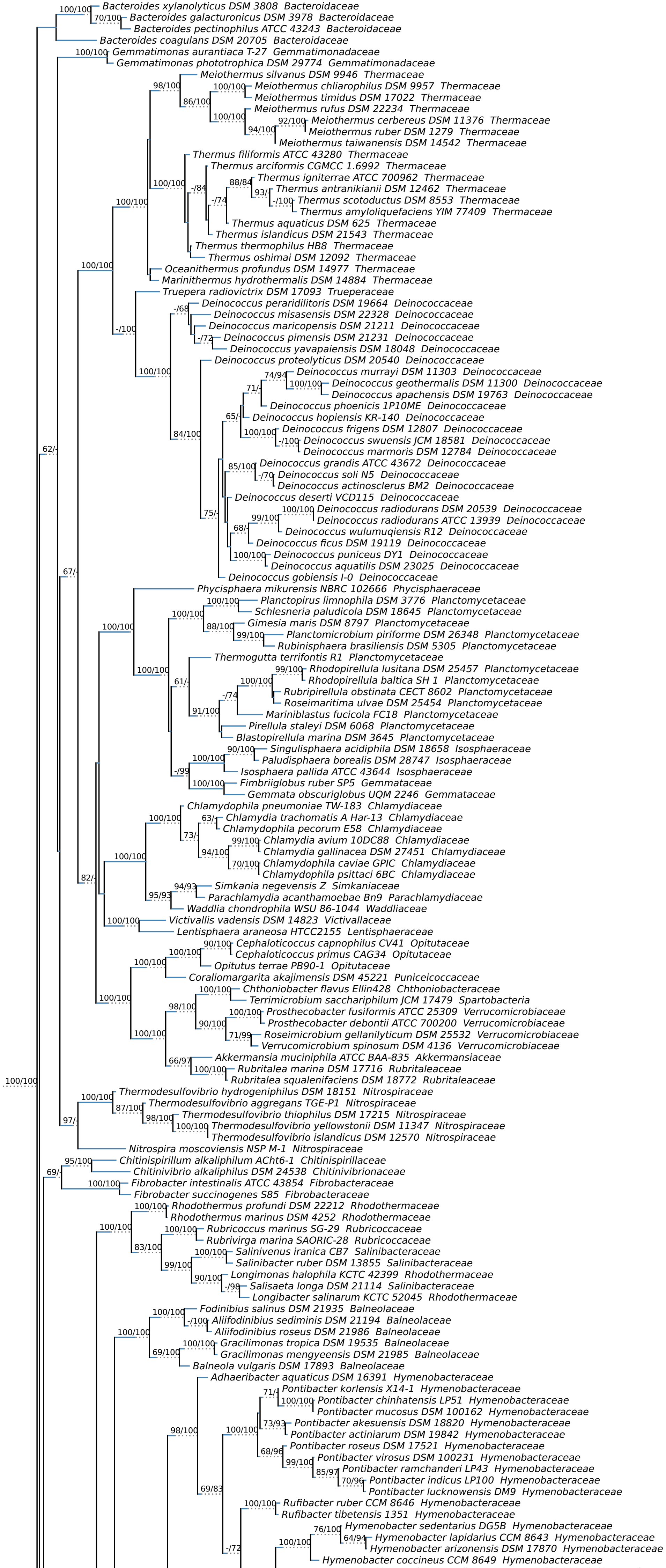
Bacteroides xylanolyticus DSM 3808 Bacteroidaceae
Bacteroides galacturonicus DSM 3978 Bacteroidaceae
Bacteroides pectinophilus ATCC 43243 Bacteroidaceae
Bacteroides coagulans DSM 20705 Bacteroidaceae
Gemmatimonas aurantiaca T-27 Gemmatimonadaceae
Gemmatimonas phototrophica DSM 29774 Gemmatimonadaceae
Meiothermus silvanus DSM 9946 Thermaceae
Meiothermus chliarophilus DSM 9957 Thermaceae
Meiothermus timidus DSM 17022 Thermaceae
Meiothermus rufus DSM 22234 Thermaceae
Meiothermus cerbereus DSM 11376 Thermaceae
Meiothermus ruber DSM 1279 Thermaceae
Meiothermus taiwanensis DSM 14542 Thermaceae
Thermus filiformis ATCC 43280 Thermaceae
Thermus arciformis CGMCC 1.6992 Thermaceae
Thermus igniterrae ATCC 700962 Thermaceae
Thermus antranikianii DSM 12462 Thermaceae
Thermus scotoductus DSM 8553 Thermaceae
Thermus amyloliquefaciens YIM 77409 Thermaceae
Thermus aquaticus DSM 625 Thermaceae
Thermus islandicus DSM 21543 Thermaceae
Thermus thermophilus HB8 Thermaceae
Thermus oshimai DSM 12092 Thermaceae
Oceanithermus profundus DSM 14977 Thermaceae
Marinithermus hydrothermalis DSM 14884 Thermaceae
Truepera radiovictrix DSM 17093 Trueperaceae
Deinococcus peraridilitoris DSM 19664 Deinococcaceae
Deinococcus misasensis DSM 22328 Deinococcaceae
Deinococcus maricopensis DSM 21211 Deinococcaceae
Deinococcus pimensis DSM 21231 Deinococcaceae
Deinococcus yavapaiensis DSM 18048 Deinococcaceae
Deinococcus proteolyticus DSM 20540 Deinococcaceae
Deinococcus murrayi DSM 11303 Deinococcaceae
Deinococcus geothermalis DSM 11300 Deinococcaceae
Deinococcus apachensis DSM 19763 Deinococcaceae
Deinococcus phoenicis 1P10ME Deinococcaceae
Deinococcus hopiensis KR-140 Deinococcaceae
Deinococcus frigens DSM 12807 Deinococcaceae
Deinococcus swuensis JCM 18581 Deinococcaceae
Deinococcus marmoris DSM 12784 Deinococcaceae
Deinococcus grandis ATCC 43672 Deinococcaceae
Deinococcus soli N5 Deinococcaceae
Deinococcus actinosclerus BM2 Deinococcaceae
Deinococcus deserti VCD115 Deinococcaceae
Deinococcus radiodurans DSM 20539 Deinococcaceae
Deinococcus radiodurans ATCC 13939 Deinococcaceae
Deinococcus wulumuqiensis R12 Deinococcaceae
Deinococcus ficus DSM 19119 Deinococcaceae
Deinococcus puniceus DY1 Deinococcaceae
Deinococcus aquatilis DSM 23025 Deinococcaceae
Deinococcus gobiensis I-0 Deinococcaceae
Phycisphaera mikurensis NBRC 102666 Phycisphaeraceae
Planctopirus limnophila DSM 3776 Planctomycetaceae
Schlesneria paludicola DSM 18645 Planctomycetaceae
Gimesia maris DSM 8797 Planctomycetaceae
Planctomicrobium piriforme DSM 26348 Planctomycetaceae
Rubinisphaera brasiliensis DSM 5305 Planctomycetaceae
Thermogutta terrifontis R1 Planctomycetaceae
Rhodopirellula lusitana DSM 25457 Planctomycetaceae
Rhodopirellula baltica SH 1 Planctomycetaceae
Rubripirellula obstinata CECT 8602 Planctomycetaceae
Roseimaritima ulvae DSM 25454 Planctomycetaceae
Mariniblastus fucicola FC18 Planctomycetaceae
Pirellula staleyi DSM 6068 Planctomycetaceae
Blastopirellula marina DSM 3645 Planctomycetaceae
Singulisphaera acidiphila DSM 18658 Isosphaeraceae
Paludisphaera borealis DSM 28747 Isosphaeraceae
Isosphaera pallida ATCC 43644 Isosphaeraceae
Fimbriiglobus ruber SP5 Gemmataceae
Gemmata obscuriglobus UQM 2246 Gemmataceae
Chlamydophila pneumoniae TW-183 Chlamydiaceae
Chlamydia trachomatis A Har-13 Chlamydiaceae
Chlamydophila pecorum E58 Chlamydiaceae
Chlamydia avium 10DC88 Chlamydiaceae
Chlamydia gallinacea DSM 27451 Chlamydiaceae
Chlamydophila caviae GPIC Chlamydiaceae
Chlamydophila psittaci 6BC Chlamydiaceae
Simkania negevensis Z Simkaniaceae
Parachlamydia acanthamoebae Bn9 Parachlamydiaceae
Waddlia chondrophila WSU 86-1044 Waddliaceae
Victivallis vadensis DSM 14823 Victivallaceae
Lentisphaera araneosa HTCC2155 Lentisphaeraceae
Cephaloticoccus capnophilus CV41 Opitutaceae
Cephaloticoccus primus CAG34 Opitutaceae
Opitutus terrae PB90-1 Opitutaceae
Coraliomargarita akajimensis DSM 45221 Puniceicoccaceae
Chthoniobacter flavus Ellin428 Chthoniobacteraceae
Terrimicrobium sacchariphilum JCM 17479 Spartobacteria
Prosthecobacter fusiformis ATCC 25309 Verrucomicrobiaceae
Prosthecobacter debontii ATCC 700200 Verrucomicrobiaceae
Roseimicrobium gellanilyticum DSM 25532 Verrucomicrobiaceae
Verrucomicrobium spinosum DSM 4136 Verrucomicrobiaceae
Akkermansia muciniphila ATCC BAA-835 Akkermansiaceae
Rubritalea marina DSM 17716 Rubritaleaceae
Rubritalea squalenifaciens DSM 18772 Rubritaleaceae
Thermodesulfovibrio hydrogeniphilus DSM 18151 Nitrospiraceae
Thermodesulfovibrio aggregans TGE-P1 Nitrospiraceae
Thermodesulfovibrio thiophilus DSM 17215 Nitrospiraceae
Thermodesulfovibrio yellowstonii DSM 11347 Nitrospiraceae
Thermodesulfovibrio islandicus DSM 12570 Nitrospiraceae
Nitrospira moscoviensis NSP M-1 Nitrospiraceae
Chitinispirillum alkaliphilum ACht6-1 Chitinispirillaceae
Chitinivibrio alkaliphilus DSM 24538 Chitinivibrionaceae
Fibrobacter intestinalis ATCC 43854 Fibrobacteraceae
Fibrobacter succinogenes S85 Fibrobacteraceae
Rhodothermus profundi DSM 22212 Rhodothermaceae
Rhodothermus marinus DSM 4252 Rhodothermaceae
Rubricoccus marinus SG-29 Rubricoccaceae
Rubrivirga marina SAORIC-28 Rubricoccaceae
Salinivenus iranica CB7 Salinibacteraceae
Salinibacter ruber DSM 13855 Salinibacteraceae
Longimonas halophila KCTC 42399 Rhodothermaceae
Salisaeta longa DSM 21114 Salinibacteraceae
Longibacter salinarum KCTC 52045 Rhodothermaceae
Fodinibius salinus DSM 21935 Balneolaceae
Aliifodinibius sediminis DSM 21194 Balneolaceae
Aliifodinibius roseus DSM 21986 Balneolaceae
Gracilimonas tropica DSM 19535 Balneolaceae
Gracilimonas mengyeensis DSM 21985 Balneolaceae
Balneola vulgaris DSM 17893 Balneolaceae
Adhaeribacter aquaticus DSM 16391 Hymenobacteraceae
Pontibacter korlensis X14-1 Hymenobacteraceae
Pontibacter chinhatensis LP51 Hymenobacteraceae
Pontibacter mucosus DSM 100162 Hymenobacteraceae
Pontibacter akesuensis DSM 18820 Hymenobacteraceae
Pontibacter actiniarum DSM 19842 Hymenobacteraceae
Pontibacter roseus DSM 17521 Hymenobacteraceae
Pontibacter virosus DSM 100231 Hymenobacteraceae
Pontibacter ramchanderi LP43 Hymenobacteraceae
Pontibacter indicus LP100 Hymenobacteraceae
Pontibacter lucknowensis DM9 Hymenobacteraceae
Rufibacter ruber CCM 8646 Hymenobacteraceae
Rufibacter tibetensis 1351 Hymenobacteraceae
Hymenobacter sedentarius DG5B Hymenobacteraceae
Hymenobacter lapidarius CCM 8643 Hymenobacteraceae
Hymenobacter arizonensis DSM 17870 Hymenobacteraceae
Hymenobacter coccineus CCM 8649 Hymenobacteraceae

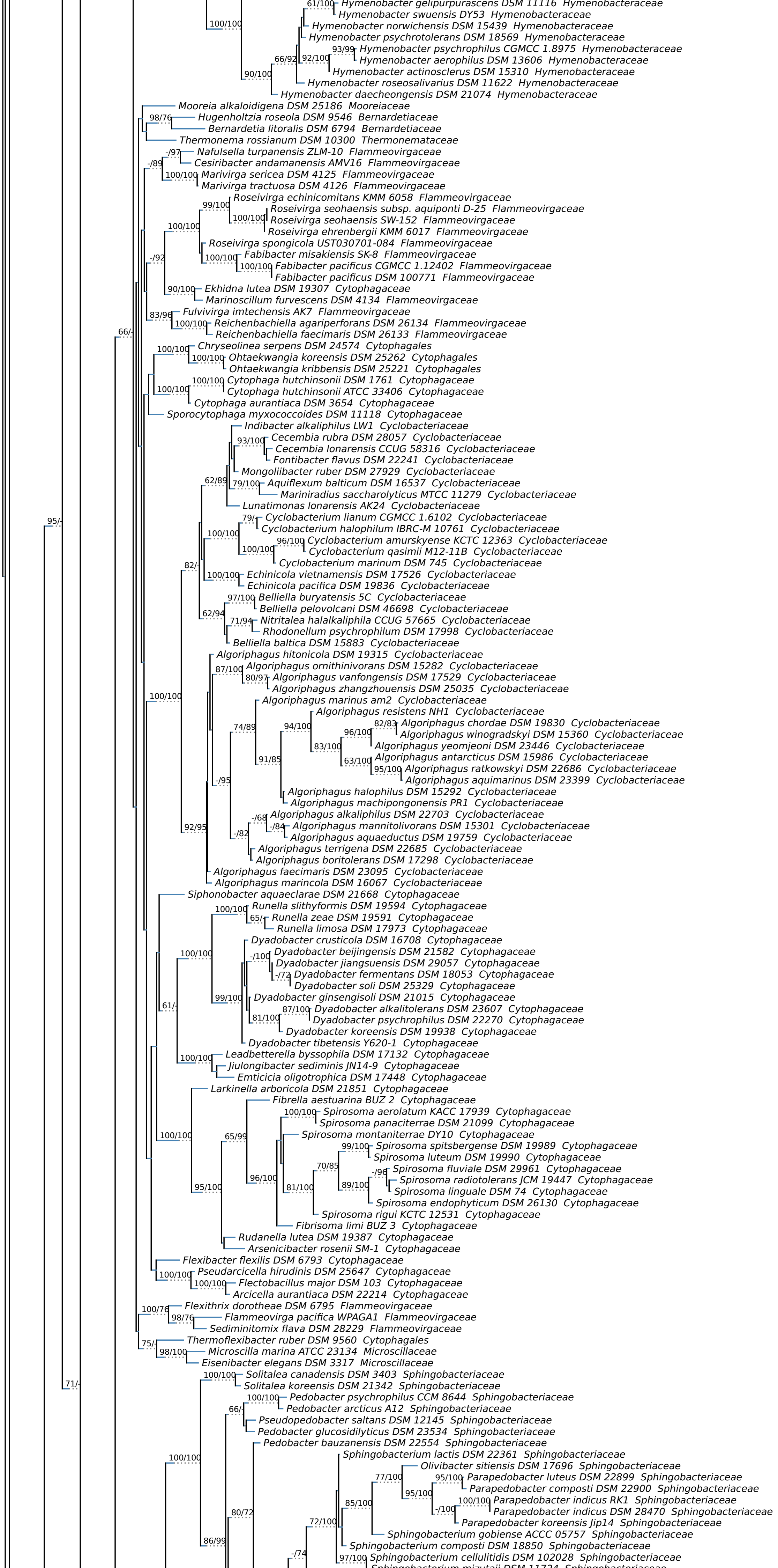

Hymenobacter gelipurpurascens DSM 11116 Hymenobacteraceae
Hymenobacter swuensis DY53 Hymenobacteraceae
Hymenobacter norwichensis DSM 15439 Hymenobacteraceae
Hymenobacter psychrotolerans DSM 18569 Hymenobacteraceae
Hymenobacter psychrophilus CGMCC 1.8975 Hymenobacteraceae
Hymenobacter aerophilus DSM 13606 Hymenobacteraceae
Hymenobacter actinosclerus DSM 15310 Hymenobacteraceae
Hymenobacter roseosalivarius DSM 11622 Hymenobacteraceae
Hymenobacter daecheongensis DSM 21074 Hymenobacteraceae
Mooreia alkaloidigena DSM 25186 Mooreiaceae
Hugenholtzia roseola DSM 9546 Bernardetiaceae
Bernardetia litoralis DSM 6794 Bernardetiaceae
Thermonema rossianum DSM 10300 Thermonemataceae
Nafulsella turpanensis ZLM-10 Flammeovirgaceae
Cesiribacter andamanensis AMV16 Flammeovirgaceae
Marivirga sericea DSM 4125 Flammeovirgaceae
Marivirga tractuosa DSM 4126 Flammeovirgaceae
Roseivirga echinicomitans KMM 6058 Flammeovirgaceae
Roseivirga seohaensis subsp. aquiponti D-25 Flammeovirgaceae
Roseivirga seohaensis SW-152 Flammeovirgaceae
Roseivirga ehrenbergii KMM 6017 Flammeovirgaceae
Roseivirga spongicola UST030701-084 Flammeovirgaceae
Fabibacter misakiensis SK-8 Flammeovirgaceae
Fabibacter pacificus CGMCC 1.12402 Flammeovirgaceae
Fabibacter pacificus DSM 100771 Flammeovirgaceae
Ekhidna lutea DSM 19307 Cytophagaceae
Marinoscillum furvescens DSM 4134 Flammeovirgaceae
Fulvivirga imtechensis AK7 Flammeovirgaceae
Reichenbachiella agariperforans DSM 26134 Flammeovirgaceae
Reichenbachiella faecimaris DSM 26133 Flammeovirgaceae
Chryseolinea serpens DSM 24574 Cytophagales
Ohtaekwangia koreensis DSM 25262 Cytophagales
Ohtaekwangia kribbensis DSM 25221 Cytophagales
Cytophaga hutchinsonii DSM 1761 Cytophagaceae
Cytophaga hutchinsonii ATCC 33406 Cytophagaceae
Cytophaga aurantiaca DSM 3654 Cytophagaceae
Sporocytophaga myxococcoides DSM 11118 Cytophagaceae
Indibacter alkaliphilus LW1 Cyclobacteriaceae
Cecembia rubra DSM 28057 Cyclobacteriaceae
Cecembia lonarensis CCUG 58316 Cyclobacteriaceae
Fontibacter flavus DSM 22241 Cyclobacteriaceae
Mongoliibacter ruber DSM 27929 Cyclobacteriaceae
Aquiflexum balticum DSM 16537 Cyclobacteriaceae
Mariniradius saccharolyticus MTCC 11279 Cyclobacteriaceae
Lunatimonas lonarensis AK24 Cyclobacteriaceae
Cyclobacterium lianum CGMCC 1.6102 Cyclobacteriaceae
Cyclobacterium halophilum IBRC-M 10761 Cyclobacteriaceae
Cyclobacterium amurskyense KCTC 12363 Cyclobacteriaceae
Cyclobacterium qasimii M12-11B Cyclobacteriaceae
Cyclobacterium marinum DSM 745 Cyclobacteriaceae
Echinicola vietnamensis DSM 17526 Cyclobacteriaceae
Echinicola pacifica DSM 19836 Cyclobacteriaceae
Belliella buryatensis 5C Cyclobacteriaceae
Belliella pelovolcani DSM 46698 Cyclobacteriaceae
Nitritalea halalkaliphila CCUG 57665 Cyclobacteriaceae
Rhodonellum psychrophilum DSM 17998 Cyclobacteriaceae
Belliella baltica DSM 15883 Cyclobacteriaceae
Algoriphagus hitonicola DSM 19315 Cyclobacteriaceae
Algoriphagus ornithinivorans DSM 15282 Cyclobacteriaceae
Algoriphagus vanfongensis DSM 17529 Cyclobacteriaceae
Algoriphagus zhangzhouensis DSM 25035 Cyclobacteriaceae
Algoriphagus marinus am2 Cyclobacteriaceae
Algoriphagus resistens NH1 Cyclobacteriaceae
Algoriphagus chordae DSM 19830 Cyclobacteriaceae
Algoriphagus winogradskyi DSM 15360 Cyclobacteriaceae
Algoriphagus yeomjeoni DSM 23446 Cyclobacteriaceae
Algoriphagus antarcticus DSM 15986 Cyclobacteriaceae
Algoriphagus ratkowskyi DSM 22686 Cyclobacteriaceae
Algoriphagus aquimarinus DSM 23399 Cyclobacteriaceae
Algoriphagus halophilus DSM 15292 Cyclobacteriaceae
Algoriphagus machipongonensis PR1 Cyclobacteriaceae
Algoriphagus alkaliphilus DSM 22703 Cyclobacteriaceae
Algoriphagus mannitolivorans DSM 15301 Cyclobacteriaceae
Algoriphagus aquaeductus DSM 19759 Cyclobacteriaceae
Algoriphagus terrigena DSM 22685 Cyclobacteriaceae
Algoriphagus boritolerans DSM 17298 Cyclobacteriaceae
Algoriphagus faecimaris DSM 23095 Cyclobacteriaceae
Algoriphagus marincola DSM 16067 Cyclobacteriaceae
Siphonobacter aquaeclarae DSM 21668 Cytophagaceae
Runella slithyformis DSM 19594 Cytophagaceae
Runella zeae DSM 19591 Cytophagaceae
Runella limosa DSM 17973 Cytophagaceae
Dyadobacter crusticola DSM 16708 Cytophagaceae
Dyadobacter beijingensis DSM 21582 Cytophagaceae
Dyadobacter jiangsuensis DSM 29057 Cytophagaceae
Dyadobacter fermentans DSM 18053 Cytophagaceae
Dyadobacter soli DSM 25329 Cytophagaceae
Dyadobacter ginsengisoli DSM 21015 Cytophagaceae
Dyadobacter alkalitolerans DSM 23607 Cytophagaceae
Dyadobacter psychrophilus DSM 22270 Cytophagaceae
Dyadobacter koreensis DSM 19938 Cytophagaceae
Dyadobacter tibetensis Y620-1 Cytophagaceae
Leadbetterella byssophila DSM 17132 Cytophagaceae
Jiulongibacter sediminis JN14-9 Cytophagaceae
Emticicia oligotrophica DSM 17448 Cytophagaceae
Larkinella arboricola DSM 21851 Cytophagaceae
Fibrella aestuarina BUZ 2 Cytophagaceae
Spirosoma aerolatum KACC 17939 Cytophagaceae
Spirosoma panaciterrae DSM 21099 Cytophagaceae
Spirosoma montaniterrae DY10 Cytophagaceae
Spirosoma spitsbergense DSM 19989 Cytophagaceae
Spirosoma luteum DSM 19990 Cytophagaceae
Spirosoma fluviale DSM 29961 Cytophagaceae
Spirosoma radiotolerans JCM 19447 Cytophagaceae
Spirosoma linguale DSM 74 Cytophagaceae
Spirosoma endophyticum DSM 26130 Cytophagaceae
Spirosoma rigui KCTC 12531 Cytophagaceae
Fibrisoma limi BUZ 3 Cytophagaceae
Rudanella lutea DSM 19387 Cytophagaceae
Arsenicibacter rosenii SM-1 Cytophagaceae
Flexibacter flexilis DSM 6793 Cytophagaceae
Pseudarcicella hirudinis DSM 25647 Cytophagaceae
Flectobacillus major DSM 103 Cytophagaceae
Arcicella aurantiaca DSM 22214 Cytophagaceae
Flexithrix dorotheae DSM 6795 Flammeovirgaceae
Flammeovirga pacifica WPAGA1 Flammeovirgaceae
Sediminitomix flava DSM 28229 Flammeovirgaceae
Thermoflexibacter ruber DSM 9560 Cytophagales
Microscilla marina ATCC 23134 Microscillaceae
Eisenibacter elegans DSM 3317 Microscillaceae
Solitalea canadensis DSM 3403 Sphingobacteriaceae
Solitalea koreensis DSM 21342 Sphingobacteriaceae
Pedobacter psychrophilus CCM 8644 Sphingobacteriaceae
Pedobacter arcticus A12 Sphingobacteriaceae
Pseudopedobacter saltans DSM 12145 Sphingobacteriaceae
Pedobacter glucosidilyticus DSM 23534 Sphingobacteriaceae
Pedobacter bauzanensis DSM 22554 Sphingobacteriaceae
Sphingobacterium lactis DSM 22361 Sphingobacteriaceae
Olivibacter sitiensis DSM 17696 Sphingobacteriaceae
Parapedobacter luteus DSM 22899 Sphingobacteriaceae
Parapedobacter composti DSM 22900 Sphingobacteriaceae
Parapedobacter indicus RK1 Sphingobacteriaceae
Parapedobacter indicus DSM 28470 Sphingobacteriaceae
Parapedobacter koreensis Jip14 Sphingobacteriaceae
Sphingobacterium gobiense ACCC 05757 Sphingobacteriaceae
Sphingobacterium composti DSM 18850 Sphingobacteriaceae
Sphingobacterium cellulitidis DSM 102028 Sphingobacteriaceae

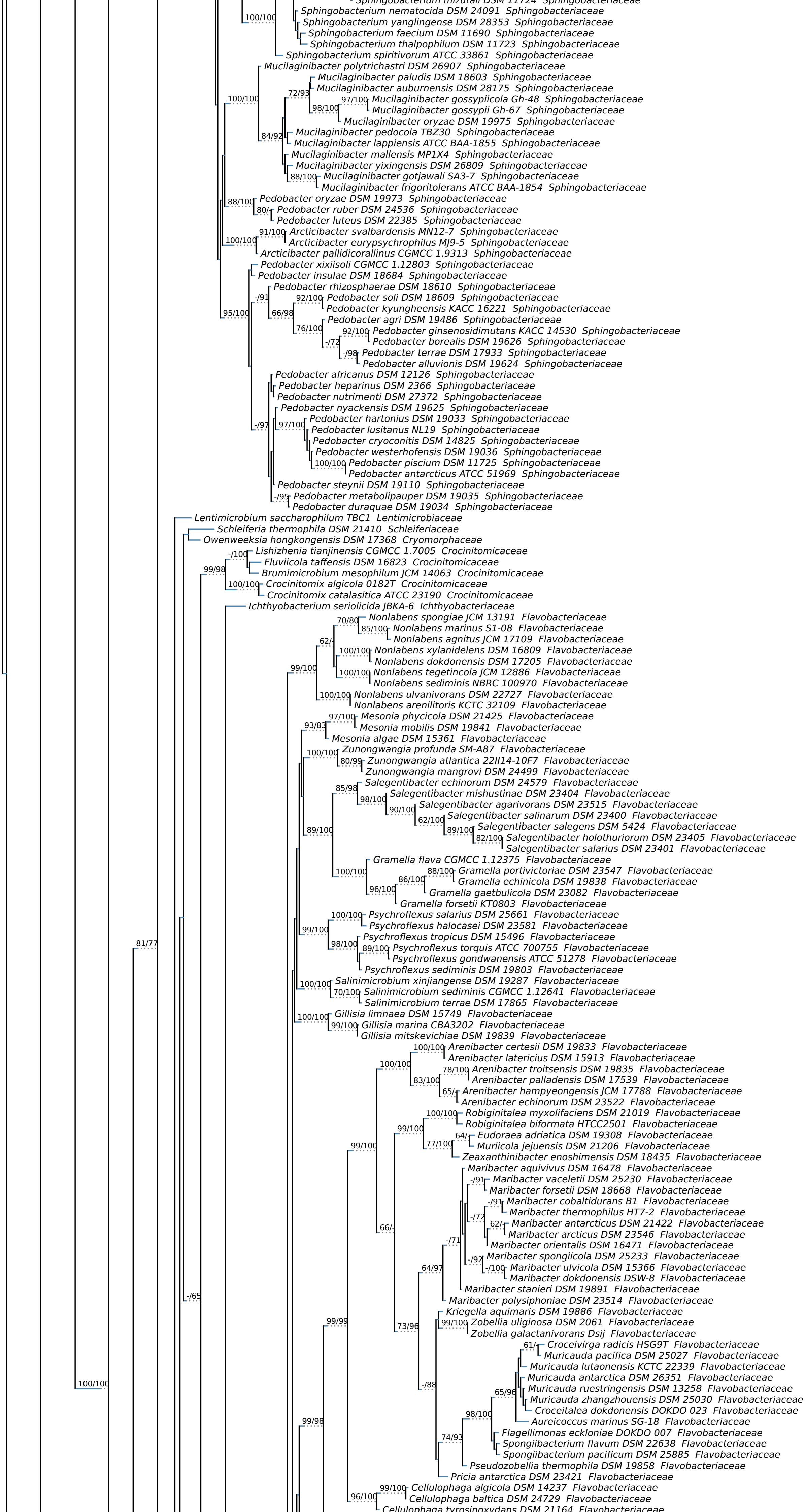
Sphingobacterium nematocida DSM 24091 Sphingobacteriaceae
Sphingobacterium yanglingense DSM 28353 Sphingobacteriaceae
Sphingobacterium faecium DSM 11690 Sphingobacteriaceae
Sphingobacterium thalpophilum DSM 11723 Sphingobacteriaceae
Sphingobacterium spiritivorum ATCC 33861 Sphingobacteriaceae
Mucilaginibacter polytrichastri DSM 26907 Sphingobacteriaceae
Mucilaginibacter paludis DSM 18603 Sphingobacteriaceae
Mucilaginibacter auburnensis DSM 28175 Sphingobacteriaceae
Mucilaginibacter gossypiicola Gh-48 Sphingobacteriaceae
Mucilaginibacter gossypii Gh-67 Sphingobacteriaceae
Mucilaginibacter oryzae DSM 19975 Sphingobacteriaceae
Mucilaginibacter pedocola TBZ30 Sphingobacteriaceae
Mucilaginibacter lappiensis ATCC BAA-1855 Sphingobacteriaceae
Mucilaginibacter mallensis MP1X4 Sphingobacteriaceae
Mucilaginibacter yixingensis DSM 26809 Sphingobacteriaceae
Mucilaginibacter gotjawali SA3-7 Sphingobacteriaceae
Mucilaginibacter frigoritolerans ATCC BAA-1854 Sphingobacteriaceae
Pedobacter oryzae DSM 19973 Sphingobacteriaceae
Pedobacter ruber DSM 24536 Sphingobacteriaceae
Pedobacter luteus DSM 22385 Sphingobacteriaceae
Arcticibacter svalbardensis MN12-7 Sphingobacteriaceae
Arcticibacter eurypsychrophilus MJ9-5 Sphingobacteriaceae
Arcticibacter pallidicorallinus CGMCC 1.9313 Sphingobacteriaceae
Pedobacter xixiisoli CGMCC 1.12803 Sphingobacteriaceae
Pedobacter insulae DSM 18684 Sphingobacteriaceae
Pedobacter rhizosphaerae DSM 18610 Sphingobacteriaceae
Pedobacter soli DSM 18609 Sphingobacteriaceae
Pedobacter kyungheensis KACC 16221 Sphingobacteriaceae
Pedobacter agri DSM 19486 Sphingobacteriaceae
Pedobacter ginsenosidimutans KACC 14530 Sphingobacteriaceae
Pedobacter borealis DSM 19626 Sphingobacteriaceae
Pedobacter terrae DSM 17933 Sphingobacteriaceae
Pedobacter alluvionis DSM 19624 Sphingobacteriaceae
Pedobacter africanus DSM 12126 Sphingobacteriaceae
Pedobacter heparinus DSM 2366 Sphingobacteriaceae
Pedobacter nutrimenti DSM 27372 Sphingobacteriaceae
Pedobacter nyackensis DSM 19625 Sphingobacteriaceae
Pedobacter hartonius DSM 19033 Sphingobacteriaceae
Pedobacter lusitanus NL19 Sphingobacteriaceae
Pedobacter cryoconitis DSM 14825 Sphingobacteriaceae
Pedobacter westerhofensis DSM 19036 Sphingobacteriaceae
Pedobacter piscium DSM 11725 Sphingobacteriaceae
Pedobacter antarcticus ATCC 51969 Sphingobacteriaceae
Pedobacter steynii DSM 19110 Sphingobacteriaceae
Pedobacter metabolipauper DSM 19035 Sphingobacteriaceae
Pedobacter duraquae DSM 19034 Sphingobacteriaceae
Lentimicrobium saccharophilum TBC1 Lentimicrobiaceae
Schleiferia thermophila DSM 21410 Schleiferiaceae
Owenweeksia hongkongensis DSM 17368 Cryomorphaceae
Lishizhenia tianjinensis CGMCC 1.7005 Crocinitomicaceae
Fluviicola taffensis DSM 16823 Crocinitomicaceae
Brumimicrobium mesophilum JCM 14063 Crocinitomicaceae
Crocinitomix algicola 0182T Crocinitomicaceae
Crocinitomix catalasitica ATCC 23190 Crocinitomicaceae
Ichthyobacterium seriolicida JBKA-6 Ichthyobacteriaceae
Nonlabens spongiae JCM 13191 Flavobacteriaceae
Nonlabens marinus S1-08 Flavobacteriaceae
Nonlabens agnitus JCM 17109 Flavobacteriaceae
Nonlabens xylanidelens DSM 16809 Flavobacteriaceae
Nonlabens dokdonensis DSM 17205 Flavobacteriaceae
Nonlabens tegetincola JCM 12886 Flavobacteriaceae
Nonlabens sediminis NBRC 100970 Flavobacteriaceae
Nonlabens ulvanivorans DSM 22727 Flavobacteriaceae
Nonlabens arenilitoris KCTC 32109 Flavobacteriaceae
Mesonia phycicola DSM 21425 Flavobacteriaceae
Mesonia mobilis DSM 19841 Flavobacteriaceae
Mesonia algae DSM 15361 Flavobacteriaceae
Zunongwangia profunda SM-A87 Flavobacteriaceae
Zunongwangia atlantica 22II14-10F7 Flavobacteriaceae
Zunongwangia mangrovi DSM 24499 Flavobacteriaceae
Salegentibacter echinorum DSM 24579 Flavobacteriaceae
Salegentibacter mishustinae DSM 23404 Flavobacteriaceae
Salegentibacter agarivorans DSM 23515 Flavobacteriaceae
Salegentibacter salinarum DSM 23400 Flavobacteriaceae
Salegentibacter salegens DSM 5424 Flavobacteriaceae
Salegentibacter holothuriorum DSM 23405 Flavobacteriaceae
Salegentibacter salarius DSM 23401 Flavobacteriaceae
Gramella flava CGMCC 1.12375 Flavobacteriaceae
Gramella portivictoriae DSM 23547 Flavobacteriaceae
Gramella echinicola DSM 19838 Flavobacteriaceae
Gramella gaetbulicola DSM 23082 Flavobacteriaceae
Gramella forsetii KT0803 Flavobacteriaceae
Psychroflexus salarius DSM 25661 Flavobacteriaceae
Psychroflexus halocasei DSM 23581 Flavobacteriaceae
Psychroflexus tropicus DSM 15496 Flavobacteriaceae
Psychroflexus torquis ATCC 700755 Flavobacteriaceae
Psychroflexus gondwanensis ATCC 51278 Flavobacteriaceae
Psychroflexus sediminis DSM 19803 Flavobacteriaceae
Salinimicrobium xinjiangense DSM 19287 Flavobacteriaceae
Salinimicrobium sediminis CGMCC 1.12641 Flavobacteriaceae
Salinimicrobium terrae DSM 17865 Flavobacteriaceae
Gillisia limnaea DSM 15749 Flavobacteriaceae
Gillisia marina CBA3202 Flavobacteriaceae
Gillisia mitskevichiae DSM 19839 Flavobacteriaceae
Arenibacter certesii DSM 19833 Flavobacteriaceae
Arenibacter latericius DSM 15913 Flavobacteriaceae
Arenibacter troitsensis DSM 19835 Flavobacteriaceae
Arenibacter palladensis DSM 17539 Flavobacteriaceae
Arenibacter hampyeongensis JCM 17788 Flavobacteriaceae
Arenibacter echinorum DSM 23522 Flavobacteriaceae
Robiginitalea myxolifaciens DSM 21019 Flavobacteriaceae
Robiginitalea biformata HTCC2501 Flavobacteriaceae
Eudoraea adriatica DSM 19308 Flavobacteriaceae
Muriicola jejuensis DSM 21206 Flavobacteriaceae
Zeaxanthinibacter enoshimensis DSM 18435 Flavobacteriaceae
Maribacter aquivivus DSM 16478 Flavobacteriaceae
Maribacter vaceletii DSM 25230 Flavobacteriaceae
Maribacter forsetii DSM 18668 Flavobacteriaceae
Maribacter cobaltidurans B1 Flavobacteriaceae
Maribacter thermophilus HT7-2 Flavobacteriaceae
Maribacter antarcticus DSM 21422 Flavobacteriaceae
Maribacter arcticus DSM 23546 Flavobacteriaceae
Maribacter orientalis DSM 16471 Flavobacteriaceae
Maribacter spongiicola DSM 25233 Flavobacteriaceae
Maribacter ulvicola DSM 15366 Flavobacteriaceae
Maribacter dokdonensis DSW-8 Flavobacteriaceae
Maribacter stanieri DSM 19891 Flavobacteriaceae
Maribacter polysiphoniae DSM 23514 Flavobacteriaceae
Kriegella aquimaris DSM 19886 Flavobacteriaceae
Zobellia uliginosa DSM 2061 Flavobacteriaceae
Zobellia galactanivorans Dsij Flavobacteriaceae
Croceivirga radicis HSG9T Flavobacteriaceae
Muricauda pacifica DSM 25027 Flavobacteriaceae
Muricauda lutaonensis KCTC 22339 Flavobacteriaceae
Muricauda antarctica DSM 26351 Flavobacteriaceae
Muricauda ruestringensis DSM 13258 Flavobacteriaceae
Muricauda zhangzhouensis DSM 25030 Flavobacteriaceae
Croceitalea dokdonensis DOKDO 023 Flavobacteriaceae
Aureicoccus marinus SG-18 Flavobacteriaceae
Flagellimonas eckloniae DOKDO 007 Flavobacteriaceae
Spongiibacterium flavum DSM 22638 Flavobacteriaceae
Spongiibacterium pacificum DSM 25885 Flavobacteriaceae
Pseudozobellia thermophila DSM 19858 Flavobacteriaceae
Pricia antarctica DSM 23421 Flavobacteriaceae
Cellulophaga algicola DSM 14237 Flavobacteriaceae
Cellulophaga baltica DSM 24729 Flavobacteriaceae
Cellulophaga tyrosinoxydans DSM 21164 Flavobacteriaceae

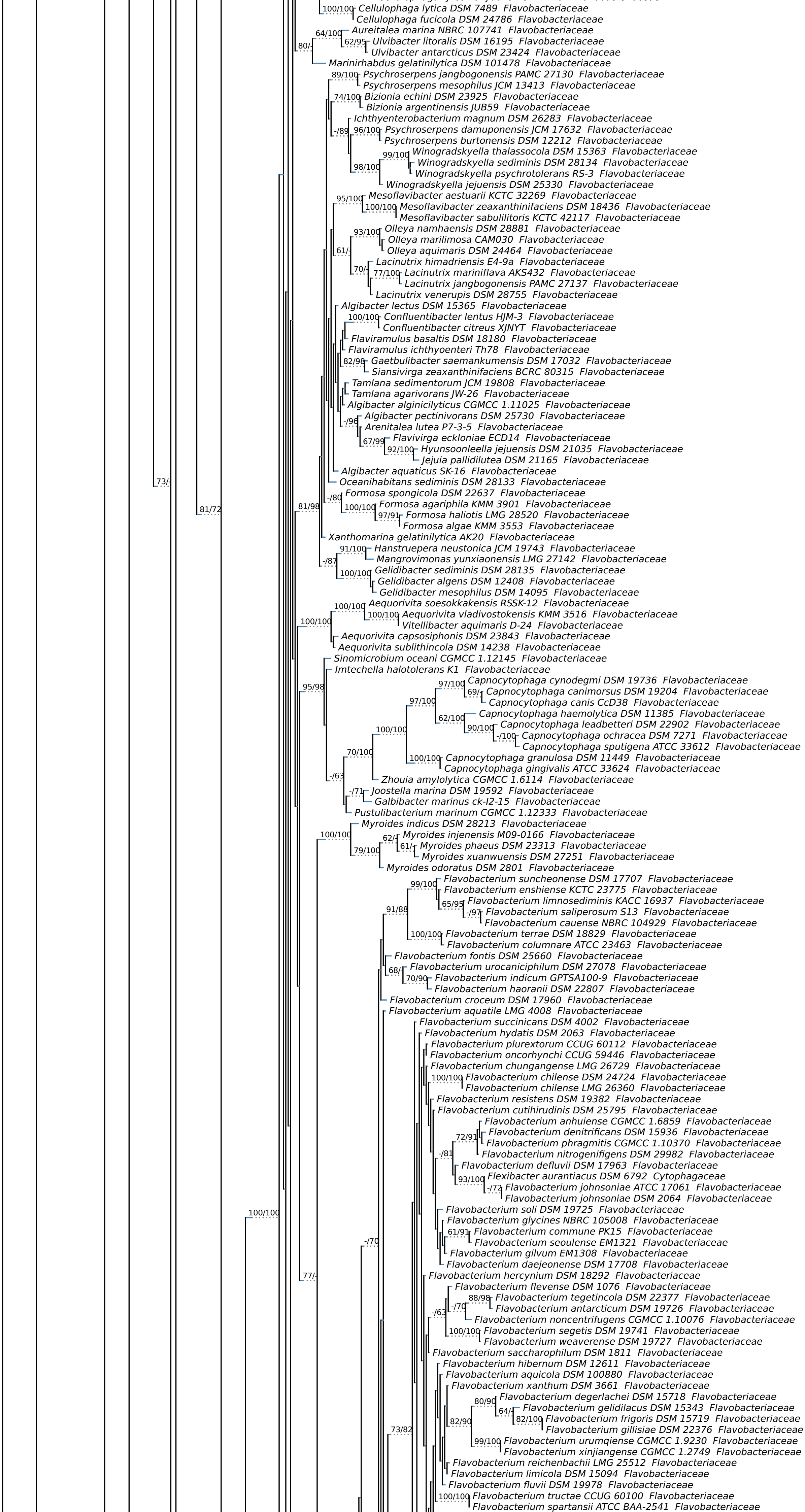
100/100 Cellulophaga lytica DSM 7489 Flavobacteriaceae
Cellulophaga fucicola DSM 24786 Flavobacteriaceae
64/100 Aureitalea marina NBRC 107741 Flavobacteriaceae
62/95 Ulvibacter litoralis DSM 16195 Flavobacteriaceae
80/- Ulvibacter antarcticus DSM 23424 Flavobacteriaceae
Marinirhabdus gelatinilytica DSM 101478 Flavobacteriaceae
89/100 Psychroserpens jangbogonensis PAMC 27130 Flavobacteriaceae
Psychroserpens mesophilus JCM 13413 Flavobacteriaceae
74/100 Bizionia echini DSM 23925 Flavobacteriaceae
Bizionia argentinensis JUB59 Flavobacteriaceae
Ichthyenterobacterium magnum DSM 26283 Flavobacteriaceae
-/89 96/100 Psychroserpens damuponensis JCM 17632 Flavobacteriaceae
Psychroserpens burtonensis DSM 12212 Flavobacteriaceae
99/100 Winogradskyella thalassocola DSM 15363 Flavobacteriaceae
Winogradskyella sediminis DSM 28134 Flavobacteriaceae
98/100 Winogradskyella psychrotolerans RS-3 Flavobacteriaceae
Winogradskyella jejuensis DSM 25330 Flavobacteriaceae
95/100 Mesoflavibacter aestuarii KCTC 32269 Flavobacteriaceae
100/100 Mesoflavibacter zeaxanthinifaciens DSM 18436 Flavobacteriaceae
Mesoflavibacter sabulilitoris KCTC 42117 Flavobacteriaceae
Olleya namhaensis DSM 28881 Flavobacteriaceae
93/100 Olleya marilimosa CAM030 Flavobacteriaceae
61/- Olleya aquimaris DSM 24464 Flavobacteriaceae
Lacinutrix himadriensis E4-9a Flavobacteriaceae
70/- 77/100 Lacinutrix mariniflava AKS432 Flavobacteriaceae
Lacinutrix jangbogonensis PAMC 27137 Flavobacteriaceae
Lacinutrix venerupis DSM 28755 Flavobacteriaceae
Algibacter lectus DSM 15365 Flavobacteriaceae
100/100 Confluentibacter lentus HJM-3 Flavobacteriaceae
Confluentibacter citreus XJNYT Flavobacteriaceae
Flaviramulus basaltis DSM 18180 Flavobacteriaceae
Flaviramulus ichthyoenteri Th78 Flavobacteriaceae
82/98 Gaetbulibacter saemankumensis DSM 17032 Flavobacteriaceae
Siansivirga zeaxanthinifaciens BCRC 80315 Flavobacteriaceae
Tamlana sedimentorum JCM 19808 Flavobacteriaceae
Tamlana agarivorans JW-26 Flavobacteriaceae
Algibacter alginicilyticus CGMCC 1.11025 Flavobacteriaceae
-/96 Algibacter pectinivorans DSM 25730 Flavobacteriaceae
Arenitalea lutea P7-3-5 Flavobacteriaceae
67/99 Flavivirga eckloniae ECD14 Flavobacteriaceae
92/100 Hyunsoonleella jejuensis DSM 21035 Flavobacteriaceae
Jejuia pallidilutea DSM 21165 Flavobacteriaceae
Algibacter aquaticus SK-16 Flavobacteriaceae
73/- Oceanihabitans sediminis DSM 28133 Flavobacteriaceae
-/80 Formosa spongicola DSM 22637 Flavobacteriaceae
81/72 81/98 100/100 Formosa agariphila KMM 3901 Flavobacteriaceae
97/91 Formosa haliotis LMG 28520 Flavobacteriaceae
Formosa algae KMM 3553 Flavobacteriaceae
Xanthomarina gelatinilytica AK20 Flavobacteriaceae
91/100 Hanstruepera neustonica JCM 19743 Flavobacteriaceae
-/87 Mangrovimonas yunxiaonensis LMG 27142 Flavobacteriaceae
Gelidibacter sediminis DSM 28135 Flavobacteriaceae
100/100 Gelidibacter algens DSM 12408 Flavobacteriaceae
Gelidibacter mesophilus DSM 14095 Flavobacteriaceae
100/100 Aequorivita soesokkakensis RSSK-12 Flavobacteriaceae
100/100 100/100 Aequorivita vladivostokensis KMM 3516 Flavobacteriaceae
Vitellibacter aquimaris D-24 Flavobacteriaceae
Aequorivita capsosiphonis DSM 23843 Flavobacteriaceae
Aequorivita sublithincola DSM 14238 Flavobacteriaceae
Sinomicrobium oceani CGMCC 1.12145 Flavobacteriaceae
Imtechella halotolerans K1 Flavobacteriaceae
97/100 Capnocytophaga cynodegmi DSM 19736 Flavobacteriaceae
95/98 69/- Capnocytophaga canimorsus DSM 19204 Flavobacteriaceae
97/100 Capnocytophaga canis CcD38 Flavobacteriaceae
62/100 Capnocytophaga haemolytica DSM 11385 Flavobacteriaceae
90/100 Capnocytophaga leadbetteri DSM 22902 Flavobacteriaceae
100/100 -/100 Capnocytophaga ochracea DSM 7271 Flavobacteriaceae
Capnocytophaga sputigena ATCC 33612 Flavobacteriaceae
70/100 100/100 Capnocytophaga granulosa DSM 11449 Flavobacteriaceae
Capnocytophaga gingivalis ATCC 33624 Flavobacteriaceae
-/63 Zhouia amylolytica CGMCC 1.6114 Flavobacteriaceae
-/71 Joostella marina DSM 19592 Flavobacteriaceae
Galbibacter marinus ck-I2-15 Flavobacteriaceae
Pustulibacterium marinum CGMCC 1.12333 Flavobacteriaceae
100/100 Myroides indicus DSM 28213 Flavobacteriaceae
62/- Myroides injenensis M09-0166 Flavobacteriaceae
61/- Myroides phaeus DSM 23313 Flavobacteriaceae
79/100 Myroides xuanwuensis DSM 27251 Flavobacteriaceae
Myroides odoratus DSM 2801 Flavobacteriaceae
99/100 Flavobacterium suncheonense DSM 17707 Flavobacteriaceae
Flavobacterium enshiense KCTC 23775 Flavobacteriaceae
65/95 Flavobacterium limnosediminis KACC 16937 Flavobacteriaceae
91/88 -/97 Flavobacterium saliperosum S13 Flavobacteriaceae
Flavobacterium cauense NBRC 104929 Flavobacteriaceae
100/100 Flavobacterium terrae DSM 18829 Flavobacteriaceae
Flavobacterium columnare ATCC 23463 Flavobacteriaceae
Flavobacterium fontis DSM 25660 Flavobacteriaceae
68/- Flavobacterium urocaniciphilum DSM 27078 Flavobacteriaceae
70/90 Flavobacterium indicum GPTSA100-9 Flavobacteriaceae
Flavobacterium haoranii DSM 22807 Flavobacteriaceae
Flavobacterium croceum DSM 17960 Flavobacteriaceae
Flavobacterium aquatile LMG 4008 Flavobacteriaceae
Flavobacterium succinicans DSM 4002 Flavobacteriaceae
Flavobacterium hydatis DSM 2063 Flavobacteriaceae
Flavobacterium plurextorum CCUG 60112 Flavobacteriaceae
Flavobacterium oncorhynchi CCUG 59446 Flavobacteriaceae
Flavobacterium chungangense LMG 26729 Flavobacteriaceae
100/100 Flavobacterium chilense DSM 24724 Flavobacteriaceae
Flavobacterium chilense LMG 26360 Flavobacteriaceae
Flavobacterium resistens DSM 19382 Flavobacteriaceae
Flavobacterium cutihirudinis DSM 25795 Flavobacteriaceae
Flavobacterium anhuiense CGMCC 1.6859 Flavobacteriaceae
Flavobacterium denitrificans DSM 15936 Flavobacteriaceae
72/91 Flavobacterium phragmitis CGMCC 1.10370 Flavobacteriaceae
-/81 Flavobacterium nitrogenifigens DSM 29982 Flavobacteriaceae
Flavobacterium defluvii DSM 17963 Flavobacteriaceae
93/100 Flexibacter aurantiacus DSM 6792 Cytophagaceae
-/72 Flavobacterium johnsoniae ATCC 17061 Flavobacteriaceae
Flavobacterium johnsoniae DSM 2064 Flavobacteriaceae
Flavobacterium soli DSM 19725 Flavobacteriaceae
100/100 Flavobacterium glycines NBRC 105008 Flavobacteriaceae
61/91 Flavobacterium commune PK15 Flavobacteriaceae
-/70 Flavobacterium seoulense EM1321 Flavobacteriaceae
Flavobacterium gilvum EM1308 Flavobacteriaceae
Flavobacterium daejeonense DSM 17708 Flavobacteriaceae
77/- Flavobacterium hercynium DSM 18292 Flavobacteriaceae
Flavobacterium flevense DSM 1076 Flavobacteriaceae
88/98 Flavobacterium tegetincola DSM 22377 Flavobacteriaceae
-/70 Flavobacterium antarcticum DSM 19726 Flavobacteriaceae
-/63 Flavobacterium noncentrifugens CGMCC 1.10076 Flavobacteriaceae
100/100 Flavobacterium segetis DSM 19741 Flavobacteriaceae
Flavobacterium weaverense DSM 19727 Flavobacteriaceae
Flavobacterium saccharophilum DSM 1811 Flavobacteriaceae
Flavobacterium hibernum DSM 12611 Flavobacteriaceae
Flavobacterium aquicola DSM 100880 Flavobacteriaceae
Flavobacterium xanthum DSM 3661 Flavobacteriaceae
80/90 Flavobacterium degerlachei DSM 15718 Flavobacteriaceae
64/- Flavobacterium gelidilacus DSM 15343 Flavobacteriaceae
82/90 82/100 Flavobacterium frigoris DSM 15719 Flavobacteriaceae
73/82 Flavobacterium gillisiae DSM 22376 Flavobacteriaceae
99/100 Flavobacterium urumqiense CGMCC 1.9230 Flavobacteriaceae
Flavobacterium xinjiangense CGMCC 1.2749 Flavobacteriaceae
Flavobacterium reichenbachii LMG 25512 Flavobacteriaceae
Flavobacterium limicola DSM 15094 Flavobacteriaceae
Flavobacterium fluvii DSM 19978 Flavobacteriaceae
100/100 Flavobacterium tructae CCUG 60100 Flavobacteriaceae
Flavobacterium spartansii ATCC BAA-2541 Flavobacteriaceae

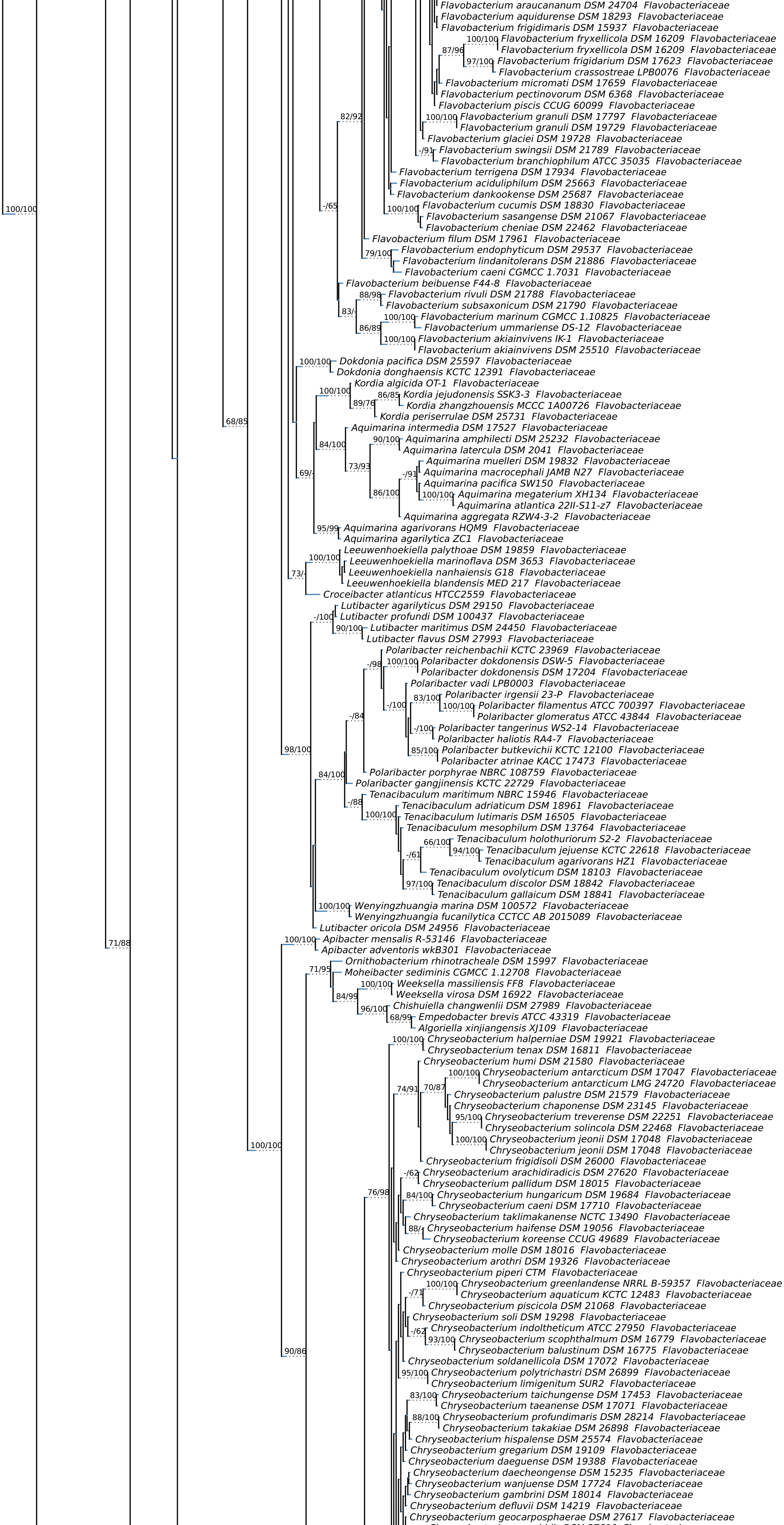
Flavobacterium araucananum DSM 24704 Flavobacteriaceae
Flavobacterium aquidurense DSM 18293 Flavobacteriaceae
Flavobacterium frigidimaris DSM 15937 Flavobacteriaceae
100/100
Flavobacterium fryxellicola DSM 16209 Flavobacteriaceae
87/96
Flavobacterium fryxellicola DSM 16209 Flavobacteriaceae
97/100
Flavobacterium frigidarium DSM 17623 Flavobacteriaceae
Flavobacterium crassostreae LPB0076 Flavobacteriaceae
Flavobacterium micromati DSM 17659 Flavobacteriaceae
Flavobacterium pectinovorum DSM 6368 Flavobacteriaceae
Flavobacterium piscis CCUG 60099 Flavobacteriaceae
82/92
100/100
Flavobacterium granuli DSM 17797 Flavobacteriaceae
Flavobacterium granuli DSM 19729 Flavobacteriaceae
Flavobacterium glaciei DSM 19728 Flavobacteriaceae
-/91
Flavobacterium swingsii DSM 21789 Flavobacteriaceae
Flavobacterium branchiophilum ATCC 35035 Flavobacteriaceae
Flavobacterium terrigena DSM 17934 Flavobacteriaceae
Flavobacterium aciduliphilum DSM 25663 Flavobacteriaceae
Flavobacterium dankookense DSM 25687 Flavobacteriaceae
100/100
-/65
Flavobacterium cucumis DSM 18830 Flavobacteriaceae
100/100
Flavobacterium sasangense DSM 21067 Flavobacteriaceae
Flavobacterium cheniae DSM 22462 Flavobacteriaceae
Flavobacterium filum DSM 17961 Flavobacteriaceae
79/100
Flavobacterium endophyticum DSM 29537 Flavobacteriaceae
Flavobacterium lindanitolerans DSM 21886 Flavobacteriaceae
Flavobacterium caeni CGMCC 1.7031 Flavobacteriaceae
Flavobacterium beibuense F44-8 Flavobacteriaceae
88/98
Flavobacterium rivuli DSM 21788 Flavobacteriaceae
Flavobacterium subsaxonicum DSM 21790 Flavobacteriaceae
83/-
100/100
Flavobacterium marinum CGMCC 1.10825 Flavobacteriaceae
86/89
Flavobacterium ummariense DS-12 Flavobacteriaceae
100/100
Flavobacterium akiainvivens IK-1 Flavobacteriaceae
Flavobacterium akiainvivens DSM 25510 Flavobacteriaceae
100/100
Dokdonia pacifica DSM 25597 Flavobacteriaceae
Dokdonia donghaensis KCTC 12391 Flavobacteriaceae
Kordia algicida OT-1 Flavobacteriaceae
100/100
86/85
Kordia jejudonensis SSK3-3 Flavobacteriaceae
89/76
Kordia zhangzhouensis MCCC 1A00726 Flavobacteriaceae
68/85
Kordia periserrulae DSM 25731 Flavobacteriaceae
Aquimarina intermedia DSM 17527 Flavobacteriaceae
90/100
Aquimarina amphilecti DSM 25232 Flavobacteriaceae
84/100
Aquimarina latercula DSM 2041 Flavobacteriaceae
Aquimarina muelleri DSM 19832 Flavobacteriaceae
73/93
Aquimarina macrocephali JAMB N27 Flavobacteriaceae
-/91
69/-
Aquimarina pacifica SW150 Flavobacteriaceae
100/100
Aquimarina megaterium XH134 Flavobacteriaceae
86/100
Aquimarina atlantica 22II-S11-z7 Flavobacteriaceae
Aquimarina aggregata RZW4-3-2 Flavobacteriaceae
95/99
Aquimarina agarivorans HQM9 Flavobacteriaceae
Aquimarina agarilytica ZC1 Flavobacteriaceae
100/100
Leeuwenhoekiella palythoae DSM 19859 Flavobacteriaceae
Leeuwenhoekiella marinoflava DSM 3653 Flavobacteriaceae
73/-
Leeuwenhoekiella nanhaiensis G18 Flavobacteriaceae
Leeuwenhoekiella blandensis MED 217 Flavobacteriaceae
Croceibacter atlanticus HTCC2559 Flavobacteriaceae
Lutibacter agarilyticus DSM 29150 Flavobacteriaceae
-/100
Lutibacter profundi DSM 100437 Flavobacteriaceae
90/100
Lutibacter maritimus DSM 24450 Flavobacteriaceae
Lutibacter flavus DSM 27993 Flavobacteriaceae
Polaribacter reichenbachii KCTC 23969 Flavobacteriaceae
-/98
100/100
Polaribacter dokdonensis DSW-5 Flavobacteriaceae
Polaribacter dokdonensis DSM 17204 Flavobacteriaceae
Polaribacter vadi LPB0003 Flavobacteriaceae
83/100
Polaribacter irgensii 23-P Flavobacteriaceae
-/100
100/100
Polaribacter filamentus ATCC 700397 Flavobacteriaceae
Polaribacter glomeratus ATCC 43844 Flavobacteriaceae
-/84
-/100
Polaribacter tangerinus WS2-14 Flavobacteriaceae
Polaribacter haliotis RA4-7 Flavobacteriaceae
98/100
85/100
Polaribacter butkevichii KCTC 12100 Flavobacteriaceae
Polaribacter atrinae KACC 17473 Flavobacteriaceae
Polaribacter porphyrae NBRC 108759 Flavobacteriaceae
84/100
Polaribacter gangjinensis KCTC 22729 Flavobacteriaceae
Tenacibaculum maritimum NBRC 15946 Flavobacteriaceae
-/88
Tenacibaculum adriaticum DSM 18961 Flavobacteriaceae
100/100
Tenacibaculum lutimaris DSM 16505 Flavobacteriaceae
Tenacibaculum mesophilum DSM 13764 Flavobacteriaceae
66/100
Tenacibaculum holothuriorum S2-2 Flavobacteriaceae
94/100
Tenacibaculum jejuense KCTC 22618 Flavobacteriaceae
-/61
Tenacibaculum agarivorans HZ1 Flavobacteriaceae
Tenacibaculum ovolyticum DSM 18103 Flavobacteriaceae
97/100
Tenacibaculum discolor DSM 18842 Flavobacteriaceae
Tenacibaculum gallaicum DSM 18841 Flavobacteriaceae
100/100
Wenyingzhuangia marina DSM 100572 Flavobacteriaceae
Wenyingzhuangia fucanilytica CCTCC AB 2015089 Flavobacteriaceae
Lutibacter oricola DSM 24956 Flavobacteriaceae
71/88
100/100
Apibacter mensalis R-53146 Flavobacteriaceae
Apibacter adventoris wkB301 Flavobacteriaceae
71/95
Ornithobacterium rhinotracheale DSM 15997 Flavobacteriaceae
Moheibacter sediminis CGMCC 1.12708 Flavobacteriaceae
100/100
Weeksella massiliensis FF8 Flavobacteriaceae
84/99
Weeksella virosa DSM 16922 Flavobacteriaceae
Chishuiella changwenlii DSM 27989 Flavobacteriaceae
96/100
68/99
Empedobacter brevis ATCC 43319 Flavobacteriaceae
Algoriella xinjiangensis XJ109 Flavobacteriaceae
100/100
Chryseobacterium halperniae DSM 19921 Flavobacteriaceae
Chryseobacterium tenax DSM 16811 Flavobacteriaceae
Chryseobacterium humi DSM 21580 Flavobacteriaceae
100/100
Chryseobacterium antarcticum DSM 17047 Flavobacteriaceae
74/91
70/87
Chryseobacterium antarcticum LMG 24720 Flavobacteriaceae
Chryseobacterium palustre DSM 21579 Flavobacteriaceae
Chryseobacterium chaponense DSM 23145 Flavobacteriaceae
95/100
Chryseobacterium treverense DSM 22251 Flavobacteriaceae
Chryseobacterium solincola DSM 22468 Flavobacteriaceae
100/100
100/100
Chryseobacterium jeonii DSM 17048 Flavobacteriaceae
Chryseobacterium jeonii DSM 17048 Flavobacteriaceae
Chryseobacterium frigidisoli DSM 26000 Flavobacteriaceae
-/62
Chryseobacterium arachidiradicis DSM 27620 Flavobacteriaceae
Chryseobacterium pallidum DSM 18015 Flavobacteriaceae
76/98
84/100
Chryseobacterium hungaricum DSM 19684 Flavobacteriaceae
Chryseobacterium caeni DSM 17710 Flavobacteriaceae
Chryseobacterium taklimakanense NCTC 13490 Flavobacteriaceae
88/-
Chryseobacterium haifense DSM 19056 Flavobacteriaceae
Chryseobacterium koreense CCUG 49689 Flavobacteriaceae
Chryseobacterium molle DSM 18016 Flavobacteriaceae
Chryseobacterium arothri DSM 19326 Flavobacteriaceae
Chryseobacterium piperi CTM Flavobacteriaceae
100/100
Chryseobacterium greenlandense NRRL B-59357 Flavobacteriaceae
-/71
Chryseobacterium aquaticum KCTC 12483 Flavobacteriaceae
Chryseobacterium piscicola DSM 21068 Flavobacteriaceae
Chryseobacterium soli DSM 19298 Flavobacteriaceae
-/62
Chryseobacterium indoltheticum ATCC 27950 Flavobacteriaceae
93/100
Chryseobacterium scophthalmum DSM 16779 Flavobacteriaceae
90/86
Chryseobacterium balustinum DSM 16775 Flavobacteriaceae
Chryseobacterium soldanellicola DSM 17072 Flavobacteriaceae
95/100
Chryseobacterium polytrichastri DSM 26899 Flavobacteriaceae
Chryseobacterium limigenitum SUR2 Flavobacteriaceae
83/100
Chryseobacterium taichungense DSM 17453 Flavobacteriaceae
Chryseobacterium taeanense DSM 17071 Flavobacteriaceae
88/100
Chryseobacterium profundimaris DSM 28214 Flavobacteriaceae
Chryseobacterium takakiae DSM 26898 Flavobacteriaceae
Chryseobacterium hispalense DSM 25574 Flavobacteriaceae
Chryseobacterium gregarium DSM 19109 Flavobacteriaceae
Chryseobacterium daeguense DSM 19388 Flavobacteriaceae
Chryseobacterium daecheongense DSM 15235 Flavobacteriaceae
Chryseobacterium wanjuense DSM 17724 Flavobacteriaceae
Chryseobacterium gambrini DSM 18014 Flavobacteriaceae
Chryseobacterium defluvii DSM 14219 Flavobacteriaceae
Chryseobacterium geocarposphaerae DSM 27617 Flavobacteriaceae

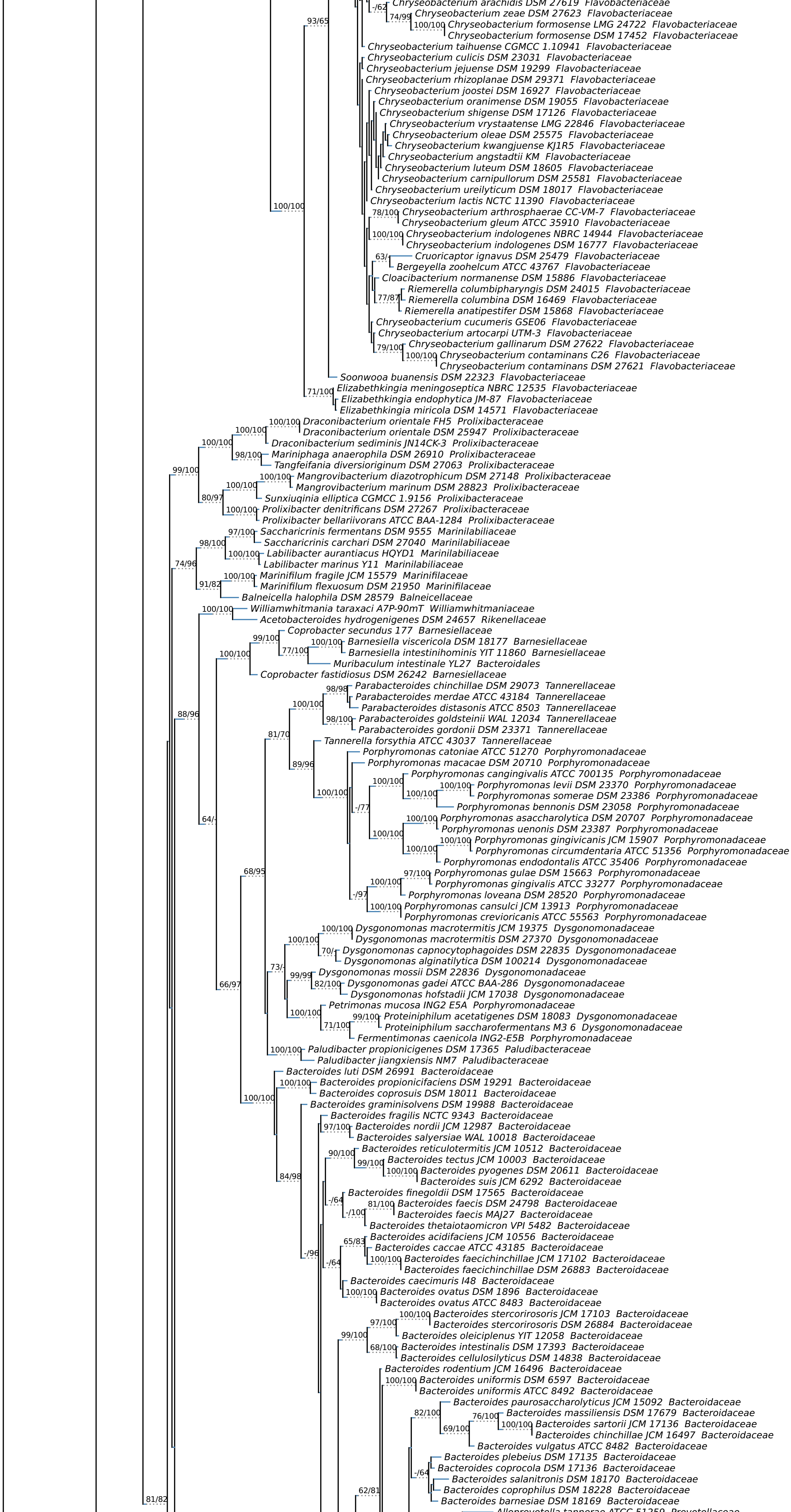
Chryseobacterium zeae DSM 27623 Flavobacteriaceae
Chryseobacterium formosense LMG 24722 Flavobacteriaceae
Chryseobacterium formosense DSM 17452 Flavobacteriaceae
Chryseobacterium taihuense CGMCC 1.10941 Flavobacteriaceae
Chryseobacterium culicis DSM 23031 Flavobacteriaceae
Chryseobacterium jejuense DSM 19299 Flavobacteriaceae
Chryseobacterium rhizoplanae DSM 29371 Flavobacteriaceae
Chryseobacterium joostei DSM 16927 Flavobacteriaceae
Chryseobacterium oranimense DSM 19055 Flavobacteriaceae
Chryseobacterium shigense DSM 17126 Flavobacteriaceae
Chryseobacterium vrystaatense LMG 22846 Flavobacteriaceae
Chryseobacterium oleae DSM 25575 Flavobacteriaceae
Chryseobacterium kwangjuense KJ1R5 Flavobacteriaceae
Chryseobacterium angstadtii KM Flavobacteriaceae
Chryseobacterium luteum DSM 18605 Flavobacteriaceae
Chryseobacterium carnipullorum DSM 25581 Flavobacteriaceae
Chryseobacterium ureilyticum DSM 18017 Flavobacteriaceae
Chryseobacterium lactis NCTC 11390 Flavobacteriaceae
Chryseobacterium arthrosphaerae CC-VM-7 Flavobacteriaceae
Chryseobacterium gleum ATCC 35910 Flavobacteriaceae
Chryseobacterium indologenes NBRC 14944 Flavobacteriaceae
Chryseobacterium indologenes DSM 16777 Flavobacteriaceae
Cruoricaptor ignavus DSM 25479 Flavobacteriaceae
Bergeyella zoohelcum ATCC 43767 Flavobacteriaceae
Cloacibacterium normanense DSM 15886 Flavobacteriaceae
Riemerella columbipharyngis DSM 24015 Flavobacteriaceae
Riemerella columbina DSM 16469 Flavobacteriaceae
Riemerella anatipestifer DSM 15868 Flavobacteriaceae
Chryseobacterium cucumeris GSE06 Flavobacteriaceae
Chryseobacterium artocarpi UTM-3 Flavobacteriaceae
Chryseobacterium gallinarum DSM 27622 Flavobacteriaceae
Chryseobacterium contaminans C26 Flavobacteriaceae
Chryseobacterium contaminans DSM 27621 Flavobacteriaceae
Soonwooa buanensis DSM 22323 Flavobacteriaceae
Elizabethkingia meningoseptica NBRC 12535 Flavobacteriaceae
Elizabethkingia endophytica JM-87 Flavobacteriaceae
Elizabethkingia miricola DSM 14571 Flavobacteriaceae
Draconibacterium orientale FH5 Prolixibacteraceae
Draconibacterium orientale DSM 25947 Prolixibacteraceae
Draconibacterium sediminis JN14CK-3 Prolixibacteraceae
Mariniphaga anaerophila DSM 26910 Prolixibacteraceae
Tangfeifania diversioriginum DSM 27063 Prolixibacteraceae
Mangrovibacterium diazotrophicum DSM 27148 Prolixibacteraceae
Mangrovibacterium marinum DSM 28823 Prolixibacteraceae
Sunxiuqinia elliptica CGMCC 1.9156 Prolixibacteraceae
Prolixibacter denitrificans DSM 27267 Prolixibacteraceae
Prolixibacter bellariivorans ATCC BAA-1284 Prolixibacteraceae
Saccharicrinis fermentans DSM 9555 Marinilabiliaceae
Saccharicrinis carchari DSM 27040 Marinilabiliaceae
Labilibacter aurantiacus HQYD1 Marinilabiliaceae
Labilibacter marinus Y11 Marinilabiliaceae
Marinifilum fragile JCM 15579 Marinifilaceae
Marinifilum flexuosum DSM 21950 Marinifilaceae
Balneicella halophila DSM 28579 Balneicellaceae
Williamwhitmania taraxaci A7P-90mT Williamwhitmaniaceae
Acetobacteroides hydrogenigenes DSM 24657 Rikenellaceae
Coprobacter secundus 177 Barnesiellaceae
Barnesiella viscericola DSM 18177 Barnesiellaceae
Barnesiella intestinihominis YIT 11860 Barnesiellaceae
Muribaculum intestinale YL27 Bacteroidales
Coprobacter fastidiosus DSM 26242 Barnesiellaceae
Parabacteroides chinchillae DSM 29073 Tannerellaceae
Parabacteroides merdae ATCC 43184 Tannerellaceae
Parabacteroides distasonis ATCC 8503 Tannerellaceae
Parabacteroides goldsteinii WAL 12034 Tannerellaceae
Parabacteroides gordonii DSM 23371 Tannerellaceae
Tannerella forsythia ATCC 43037 Tannerellaceae
Porphyromonas catoniae ATCC 51270 Porphyromonadaceae
Porphyromonas macacae DSM 20710 Porphyromonadaceae
Porphyromonas cangingivalis ATCC 700135 Porphyromonadaceae
Porphyromonas levii DSM 23370 Porphyromonadaceae
Porphyromonas somerae DSM 23386 Porphyromonadaceae
Porphyromonas bennonis DSM 23058 Porphyromonadaceae
Porphyromonas asaccharolytica DSM 20707 Porphyromonadaceae
Porphyromonas uenonis DSM 23387 Porphyromonadaceae
Porphyromonas gingivicanis JCM 15907 Porphyromonadaceae
Porphyromonas circumdentaria ATCC 51356 Porphyromonadaceae
Porphyromonas endodontalis ATCC 35406 Porphyromonadaceae
Porphyromonas gulae DSM 15663 Porphyromonadaceae
Porphyromonas gingivalis ATCC 33277 Porphyromonadaceae
Porphyromonas loveana DSM 28520 Porphyromonadaceae
Porphyromonas cansulci JCM 13913 Porphyromonadaceae
Porphyromonas crevioricanis ATCC 55563 Porphyromonadaceae
Dysgonomonas macrotermitis JCM 19375 Dysgonomonadaceae
Dysgonomonas macrotermitis DSM 27370 Dysgonomonadaceae
Dysgonomonas capnocytophagoides DSM 22835 Dysgonomonadaceae
Dysgonomonas alginatilytica DSM 100214 Dysgonomonadaceae
Dysgonomonas mossii DSM 22836 Dysgonomonadaceae
Dysgonomonas gadei ATCC BAA-286 Dysgonomonadaceae
Dysgonomonas hofstadii JCM 17038 Dysgonomonadaceae
Petrimonas mucosa ING2 E5A Porphyromonadaceae
Proteiniphilum acetatigenes DSM 18083 Dysgonomonadaceae
Proteiniphilum saccharofermentans M3 6 Dysgonomonadaceae
Fermentimonas caenicola ING2-E5B Porphyromonadaceae
Paludibacter propionicigenes DSM 17365 Paludibacteraceae
Paludibacter jiangxiensis NM7 Paludibacteraceae
Bacteroides luti DSM 26991 Bacteroidaceae
Bacteroides propionicifaciens DSM 19291 Bacteroidaceae
Bacteroides coprosuis DSM 18011 Bacteroidaceae
Bacteroides graminisolvens DSM 19988 Bacteroidaceae
Bacteroides fragilis NCTC 9343 Bacteroidaceae
Bacteroides nordii JCM 12987 Bacteroidaceae
Bacteroides salyersiae WAL 10018 Bacteroidaceae
Bacteroides reticulotermitis JCM 10512 Bacteroidaceae
Bacteroides tectus JCM 10003 Bacteroidaceae
Bacteroides pyogenes DSM 20611 Bacteroidaceae
Bacteroides suis JCM 6292 Bacteroidaceae
Bacteroides finegoldii DSM 17565 Bacteroidaceae
Bacteroides faecis DSM 24798 Bacteroidaceae
Bacteroides faecis MAJ27 Bacteroidaceae
Bacteroides thetaiotaomicron VPI 5482 Bacteroidaceae
Bacteroides acidifaciens JCM 10556 Bacteroidaceae
Bacteroides caccae ATCC 43185 Bacteroidaceae
Bacteroides faecichinchillae JCM 17102 Bacteroidaceae
Bacteroides faecichinchillae DSM 26883 Bacteroidaceae
Bacteroides caecimuris I48 Bacteroidaceae
Bacteroides ovatus DSM 1896 Bacteroidaceae
Bacteroides ovatus ATCC 8483 Bacteroidaceae
Bacteroides stercorirosoris JCM 17103 Bacteroidaceae
Bacteroides stercorirosoris DSM 26884 Bacteroidaceae
Bacteroides oleiciplenus YIT 12058 Bacteroidaceae
Bacteroides intestinalis DSM 17393 Bacteroidaceae
Bacteroides cellulosilyticus DSM 14838 Bacteroidaceae
Bacteroides rodentium JCM 16496 Bacteroidaceae
Bacteroides uniformis DSM 6597 Bacteroidaceae
Bacteroides uniformis ATCC 8492 Bacteroidaceae
Bacteroides paurosaccharolyticus JCM 15092 Bacteroidaceae
Bacteroides massiliensis DSM 17679 Bacteroidaceae
Bacteroides sartorii JCM 17136 Bacteroidaceae
Bacteroides chinchillae JCM 16497 Bacteroidaceae
Bacteroides vulgatus ATCC 8482 Bacteroidaceae
Bacteroides plebeius DSM 17135 Bacteroidaceae
Bacteroides coprocola DSM 17136 Bacteroidaceae
Bacteroides salanitronis DSM 18170 Bacteroidaceae
Bacteroides coprophilus DSM 18228 Bacteroidaceae
Bacteroides barnesiae DSM 18169 Bacteroidaceae

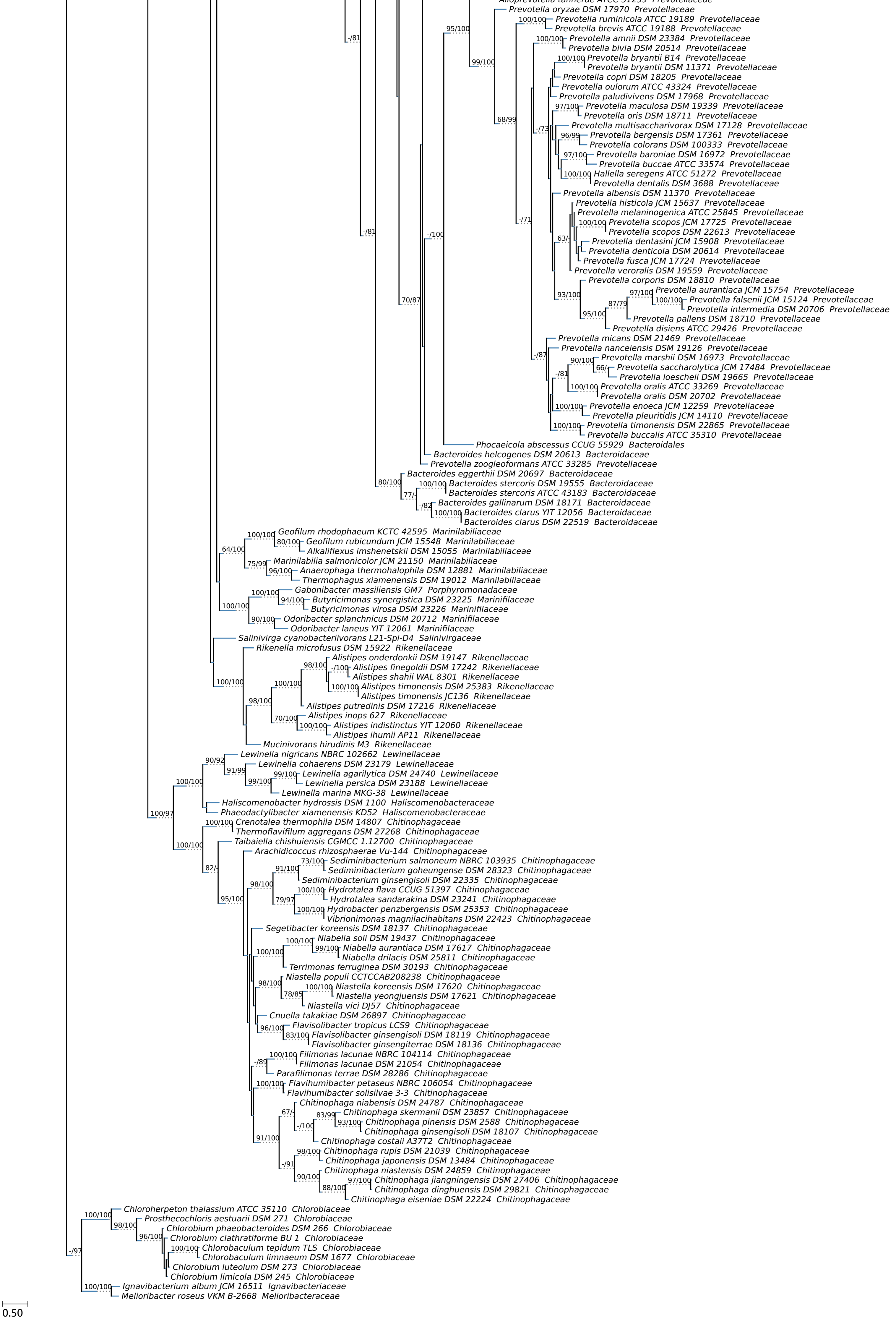
Prevotella oryzae DSM 17970 Prevotellaceae
Prevotella ruminicola ATCC 19189 Prevotellaceae
Prevotella brevis ATCC 19188 Prevotellaceae
Prevotella amnii DSM 23384 Prevotellaceae
Prevotella bivia DSM 20514 Prevotellaceae
Prevotella bryantii B14 Prevotellaceae
Prevotella bryantii DSM 11371 Prevotellaceae
Prevotella copri DSM 18205 Prevotellaceae
Prevotella oulorum ATCC 43324 Prevotellaceae
Prevotella paludivivens DSM 17968 Prevotellaceae
Prevotella maculosa DSM 19339 Prevotellaceae
Prevotella oris DSM 18711 Prevotellaceae
Prevotella multisaccharivorax DSM 17128 Prevotellaceae
Prevotella bergensis DSM 17361 Prevotellaceae
Prevotella colorans DSM 100333 Prevotellaceae
Prevotella baroniae DSM 16972 Prevotellaceae
Prevotella buccae ATCC 33574 Prevotellaceae
Hallella seregens ATCC 51272 Prevotellaceae
Prevotella dentalis DSM 3688 Prevotellaceae
Prevotella albensis DSM 11370 Prevotellaceae
Prevotella histicola JCM 15637 Prevotellaceae
Prevotella melaninogenica ATCC 25845 Prevotellaceae
Prevotella scopos JCM 17725 Prevotellaceae
Prevotella scopos DSM 22613 Prevotellaceae
Prevotella dentasini JCM 15908 Prevotellaceae
Prevotella denticola DSM 20614 Prevotellaceae
Prevotella fusca JCM 17724 Prevotellaceae
Prevotella veroralis DSM 19559 Prevotellaceae
Prevotella corporis DSM 18810 Prevotellaceae
Prevotella aurantiaca JCM 15754 Prevotellaceae
Prevotella falsenii JCM 15124 Prevotellaceae
Prevotella intermedia DSM 20706 Prevotellaceae
Prevotella pallens DSM 18710 Prevotellaceae
Prevotella disiens ATCC 29426 Prevotellaceae
Prevotella micans DSM 21469 Prevotellaceae
Prevotella nanceiensis DSM 19126 Prevotellaceae
Prevotella marshii DSM 16973 Prevotellaceae
Prevotella saccharolytica JCM 17484 Prevotellaceae
Prevotella loescheii DSM 19665 Prevotellaceae
Prevotella oralis ATCC 33269 Prevotellaceae
Prevotella oralis DSM 20702 Prevotellaceae
Prevotella enoeca JCM 12259 Prevotellaceae
Prevotella pleuritidis JCM 14110 Prevotellaceae
Prevotella timonensis DSM 22865 Prevotellaceae
Prevotella buccalis ATCC 35310 Prevotellaceae
Phocaeicola abscessus CCUG 55929 Bacteroidales
Bacteroides helcogenes DSM 20613 Bacteroidaceae
Prevotella zoogleoformans ATCC 33285 Prevotellaceae
Bacteroides eggerthii DSM 20697 Bacteroidaceae
Bacteroides stercoris DSM 19555 Bacteroidaceae
Bacteroides stercoris ATCC 43183 Bacteroidaceae
Bacteroides gallinarum DSM 18171 Bacteroidaceae
Bacteroides clarus YIT 12056 Bacteroidaceae
Bacteroides clarus DSM 22519 Bacteroidaceae
Geofilum rhodophaeum KCTC 42595 Marinilabiliaceae
Geofilum rubicundum JCM 15548 Marinilabiliaceae
Alkaliflexus imshenetskii DSM 15055 Marinilabiliaceae
Marinilabilia salmonicolor JCM 21150 Marinilabiliaceae
Anaerophaga thermohalophila DSM 12881 Marinilabiliaceae
Thermophagus xiamenensis DSM 19012 Marinilabiliaceae
Gabonibacter massiliensis GM7 Porphyromonadaceae
Butyricimonas synergistica DSM 23225 Marinifilaceae
Butyricimonas virosa DSM 23226 Marinifilaceae
Odoribacter splanchnicus DSM 20712 Marinifilaceae
Odoribacter laneus YIT 12061 Marinifilaceae
Salinivirga cyanobacteriivorans L21-Spi-D4 Salinivirgaceae
Rikenella microfusus DSM 15922 Rikenellaceae
Alistipes onderdonkii DSM 19147 Rikenellaceae
Alistipes finegoldii DSM 17242 Rikenellaceae
Alistipes shahii WAL 8301 Rikenellaceae
Alistipes timonensis DSM 25383 Rikenellaceae
Alistipes timonensis JC136 Rikenellaceae
Alistipes putredinis DSM 17216 Rikenellaceae
Alistipes inops 627 Rikenellaceae
Alistipes indistinctus YIT 12060 Rikenellaceae
Alistipes ihumii AP11 Rikenellaceae
Mucinivorans hirudinis M3 Rikenellaceae
Lewinella nigricans NBRC 102662 Lewinellaceae
Lewinella cohaerens DSM 23179 Lewinellaceae
Lewinella agarilytica DSM 24740 Lewinellaceae
Lewinella persica DSM 23188 Lewinellaceae
Lewinella marina MKG-38 Lewinellaceae
Haliscomenobacter hydrossis DSM 1100 Haliscomenobacteraceae
Phaeodactylibacter xiamenensis KD52 Haliscomenobacteraceae
Crenotalea thermophila DSM 14807 Chitinophagaceae
Thermoflavifilum aggregans DSM 27268 Chitinophagaceae
Taibaiella chishuiensis CGMCC 1.12700 Chitinophagaceae
Arachidicoccus rhizosphaerae Vu-144 Chitinophagaceae
Sediminibacterium salmoneum NBRC 103935 Chitinophagaceae
Sediminibacterium goheungense DSM 28323 Chitinophagaceae
Sediminibacterium ginsengisoli DSM 22335 Chitinophagaceae
Hydrotalea flava CCUG 51397 Chitinophagaceae
Hydrotalea sandarakina DSM 23241 Chitinophagaceae
Hydrobacter penzbergensis DSM 25353 Chitinophagaceae
Vibrionimonas magnilacihabitans DSM 22423 Chitinophagaceae
Segetibacter koreensis DSM 18137 Chitinophagaceae
Niabella soli DSM 19437 Chitinophagaceae
Niabella aurantiaca DSM 17617 Chitinophagaceae
Niabella drilacis DSM 25811 Chitinophagaceae
Terrimonas ferruginea DSM 30193 Chitinophagaceae
Niastella populi CCTCCAB208238 Chitinophagaceae
Niastella koreensis DSM 17620 Chitinophagaceae
Niastella yeongjuensis DSM 17621 Chitinophagaceae
Niastella vici DJ57 Chitinophagaceae
Cnuella takakiae DSM 26897 Chitinophagaceae
Flavisolibacter tropicus LCS9 Chitinophagaceae
Flavisolibacter ginsengisoli DSM 18119 Chitinophagaceae
Flavisolibacter ginsengiterrae DSM 18136 Chitinophagaceae
Filimonas lacunae NBRC 104114 Chitinophagaceae
Filimonas lacunae DSM 21054 Chitinophagaceae
Parafilimonas terrae DSM 28286 Chitinophagaceae
Flavihumibacter petaseus NBRC 106054 Chitinophagaceae
Flavihumibacter solisilvae 3-3 Chitinophagaceae
Chitinophaga niabensis DSM 24787 Chitinophagaceae
Chitinophaga skermanii DSM 23857 Chitinophagaceae
Chitinophaga pinensis DSM 2588 Chitinophagaceae
Chitinophaga ginsengisoli DSM 18107 Chitinophagaceae
Chitinophaga costaii A37T2 Chitinophagaceae
Chitinophaga rupis DSM 21039 Chitinophagaceae
Chitinophaga japonensis DSM 13484 Chitinophagaceae
Chitinophaga niastensis DSM 24859 Chitinophagaceae
Chitinophaga jiangningensis DSM 27406 Chitinophagaceae
Chitinophaga dinghuensis DSM 29821 Chitinophagaceae
Chitinophaga eiseniae DSM 22224 Chitinophagaceae
Chloroherpeton thalassium ATCC 35110 Chlorobiaceae
Prosthecochloris aestuarii DSM 271 Chlorobiaceae
Chlorobium phaeobacteroides DSM 266 Chlorobiaceae
Chlorobium clathratiforme BU 1 Chlorobiaceae
Chlorobaculum tepidum TLS Chlorobiaceae
Chlorobaculum limnaeum DSM 1677 Chlorobiaceae
Chlorobium luteolum DSM 273 Chlorobiaceae
Chlorobium limicola DSM 245 Chlorobiaceae
Ignavibacterium album JCM 16511 Ignavibacteriaceae
Melioribacter roseus VKM B-2668 Melioribacteraceae
0.50

Figure 6: 16S rRNA gene ML using the sampling from the GBDP tree (URT) inferred under the GTR+CAT model. The branches are scaled in terms of the expected number of substitutions per site. The numbers above the branches are support values when larger than 60% from ML (left) and MP (right) bootstrapping. Dotted parts of branches are filled in to allow proper placement of bootstrap values and are not part of the actual branch length. Each tip label ends with the family of the respective taxon.

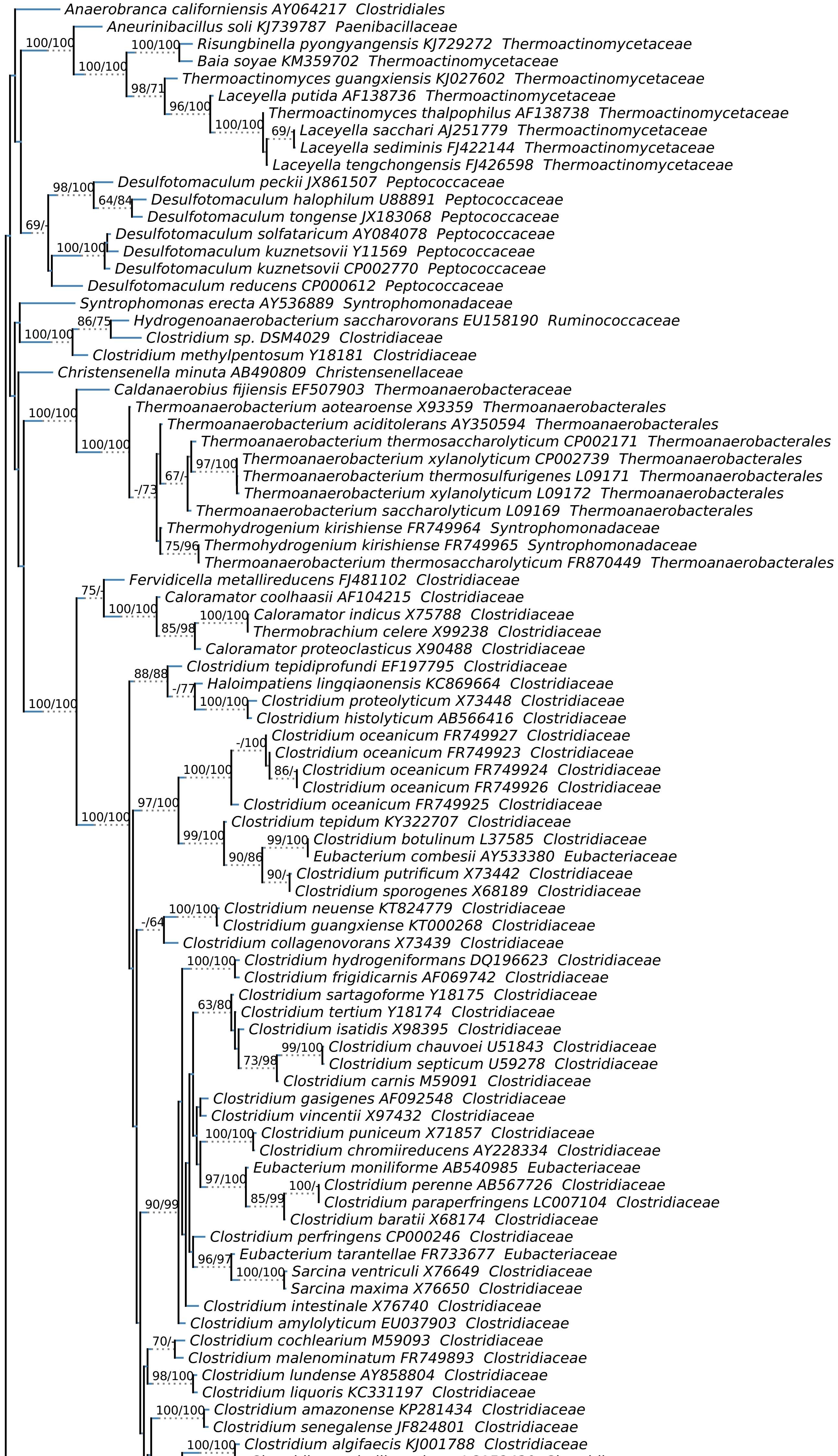
Anaerobranca californiensis AY064217 Clostridiales
Aneurinibacillus soli KJ739787 Paenibacillaceae
Risungbinella pyongyangensis KJ729272 Thermoactinomycetaceae
Baia soyae KM359702 Thermoactinomycetaceae
Thermoactinomyces guangxiensis KJ027602 Thermoactinomycetaceae
Laceyella putida AF138736 Thermoactinomycetaceae
Thermoactinomyces thalpophilus AF138738 Thermoactinomycetaceae
Laceyella sacchari AJ251779 Thermoactinomycetaceae
Laceyella sediminis FJ422144 Thermoactinomycetaceae
Laceyella tengchongensis FJ426598 Thermoactinomycetaceae
Desulfotomaculum peckii JX861507 Peptococcaceae
Desulfotomaculum halophilum U88891 Peptococcaceae
Desulfotomaculum tongense JX183068 Peptococcaceae
Desulfotomaculum solfataricum AY084078 Peptococcaceae
Desulfotomaculum kuznetsovii Y11569 Peptococcaceae
Desulfotomaculum kuznetsovii CP002770 Peptococcaceae
Desulfotomaculum reducens CP000612 Peptococcaceae
Syntrophomonas erecta AY536889 Syntrophomonadaceae
Hydrogenoanaerobacterium saccharovorans EU158190 Ruminococcaceae
Clostridium sp. DSM4029 Clostridiaceae
Clostridium methylpentosum Y18181 Clostridiaceae
Christensenella minuta AB490809 Christensenellaceae
Caldanaerobius fijiensis EF507903 Thermoanaerobacteraceae
Thermoanaerobacterium aotearoense X93359 Thermoanaerobacterales
Thermoanaerobacterium aciditolerans AY350594 Thermoanaerobacterales
Thermoanaerobacterium thermosaccharolyticum CP002171 Thermoanaerobacterales
Thermoanaerobacterium xylanolyticum CP002739 Thermoanaerobacterales
Thermoanaerobacterium thermosulfurigenes L09171 Thermoanaerobacterales
Thermoanaerobacterium xylanolyticum L09172 Thermoanaerobacterales
Thermoanaerobacterium saccharolyticum L09169 Thermoanaerobacterales
Thermohydrogenium kirishiense FR749964 Syntrophomonadaceae
Thermohydrogenium kirishiense FR749965 Syntrophomonadaceae
Thermoanaerobacterium thermosaccharolyticum FR870449 Thermoanaerobacterales
Fervidicella metallireducens FJ481102 Clostridiaceae
Caloramator coolhaasii AF104215 Clostridiaceae
Caloramator indicus X75788 Clostridiaceae
Thermobrachium celere X99238 Clostridiaceae
Caloramator proteoclasticus X90488 Clostridiaceae
Clostridium tepidiprofundi EF197795 Clostridiaceae
Haloimpatiens lingqiaonensis KC869664 Clostridiaceae
Clostridium proteolyticum X73448 Clostridiaceae
Clostridium histolyticum AB566416 Clostridiaceae
Clostridium oceanicum FR749927 Clostridiaceae
Clostridium oceanicum FR749923 Clostridiaceae
Clostridium oceanicum FR749924 Clostridiaceae
Clostridium oceanicum FR749926 Clostridiaceae
Clostridium oceanicum FR749925 Clostridiaceae
Clostridium tepidum KY322707 Clostridiaceae
Clostridium botulinum L37585 Clostridiaceae
Eubacterium combesii AY533380 Eubacteriaceae
Clostridium putrificum X73442 Clostridiaceae
Clostridium sporogenes X68189 Clostridiaceae
Clostridium neuense KT824779 Clostridiaceae
Clostridium guangxiense KT000268 Clostridiaceae
Clostridium collagenovorans X73439 Clostridiaceae
Clostridium hydrogeniformans DQ196623 Clostridiaceae
Clostridium frigidicarnis AF069742 Clostridiaceae
Clostridium sartagoforme Y18175 Clostridiaceae
Clostridium tertium Y18174 Clostridiaceae
Clostridium isatidis X98395 Clostridiaceae
Clostridium chauvoei U51843 Clostridiaceae
Clostridium septicum U59278 Clostridiaceae
Clostridium carnis M59091 Clostridiaceae
Clostridium gasigenes AF092548 Clostridiaceae
Clostridium vincentii X97432 Clostridiaceae
Clostridium puniceum X71857 Clostridiaceae
Clostridium chromiireducens AY228334 Clostridiaceae
Eubacterium moniliforme AB540985 Eubacteriaceae
Clostridium perenne AB567726 Clostridiaceae
Clostridium paraperfringens LC007104 Clostridiaceae
Clostridium baratii X68174 Clostridiaceae
Clostridium perfringens CP000246 Clostridiaceae
Eubacterium tarantellae FR733677 Eubacteriaceae
Sarcina ventriculi X76649 Clostridiaceae
Sarcina maxima X76650 Clostridiaceae
Clostridium intestinale X76740 Clostridiaceae
Clostridium amylolyticum EU037903 Clostridiaceae
Clostridium cochlearium M59093 Clostridiaceae
Clostridium malenominatum FR749893 Clostridiaceae
Clostridium lundense AY858804 Clostridiaceae
Clostridium liquoris KC331197 Clostridiaceae
Clostridium amazonense KP281434 Clostridiaceae
Clostridium senegalense JF824801 Clostridiaceae
Clostridium algifaecis KJ001788 Clostridiaceae

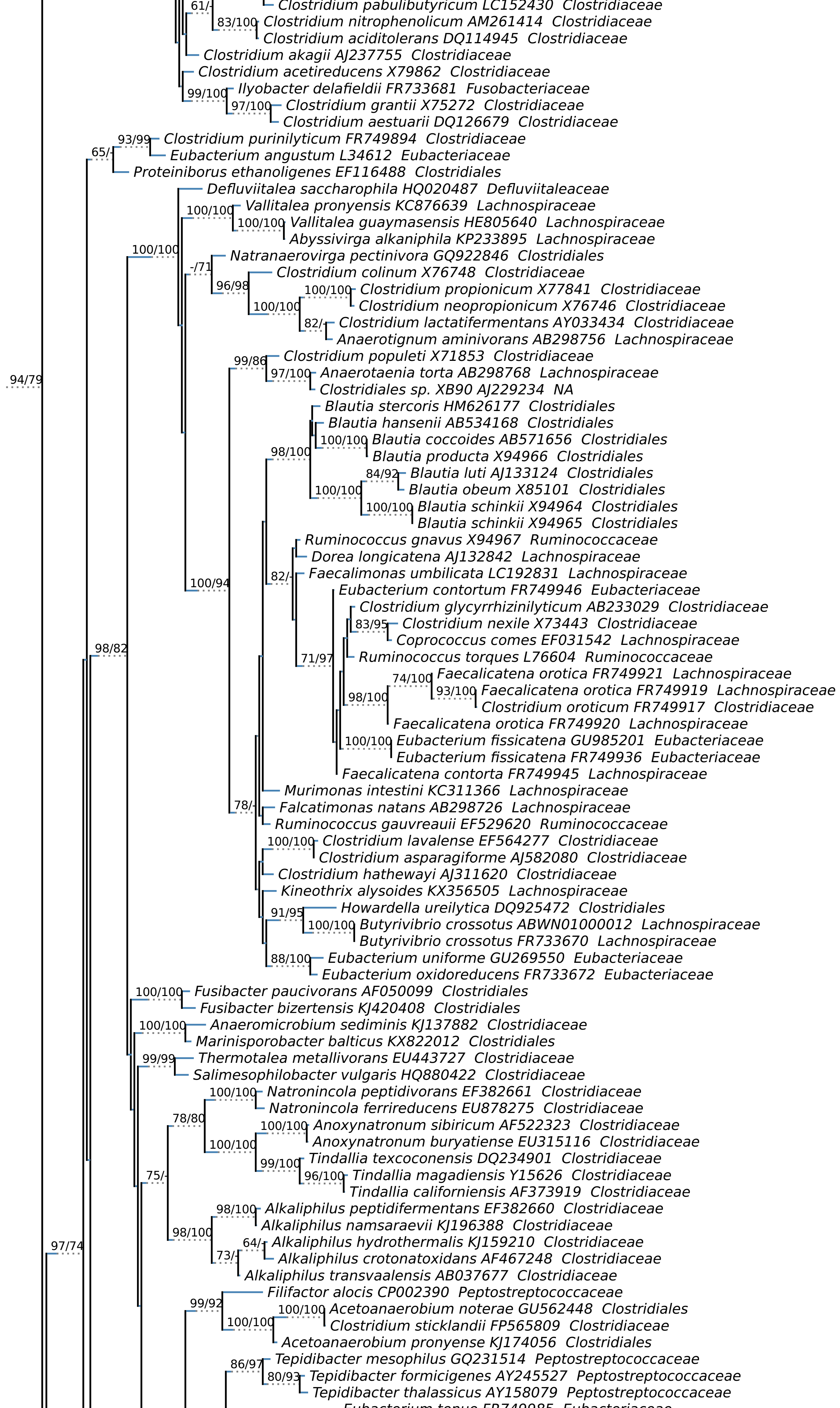
Clostridium pabulibutyricum LC152430 Clostridiaceae
Clostridium nitrophenolicum AM261414 Clostridiaceae
Clostridium aciditolerans DQ114945 Clostridiaceae
Clostridium akagii AJ237755 Clostridiaceae
Clostridium acetireducens X79862 Clostridiaceae
Ilyobacter delafieldii FR733681 Fusobacteriaceae
Clostridium grantii X75272 Clostridiaceae
Clostridium aestuarii DQ126679 Clostridiaceae
Clostridium purinilyticum FR749894 Clostridiaceae
Eubacterium angustum L34612 Eubacteriaceae
Proteiniborus ethanoligenes EF116488 Clostridiales
Defluviitalea saccharophila HQ020487 Defluviitaleaceae
Vallitalea pronyensis KC876639 Lachnospiraceae
Vallitalea guaymasensis HE805640 Lachnospiraceae
Abyssivirga alkaniphila KP233895 Lachnospiraceae
Natranaerovirga pectinivora GQ922846 Clostridiales
Clostridium colinum X76748 Clostridiaceae
Clostridium propionicum X77841 Clostridiaceae
Clostridium neopropionicum X76746 Clostridiaceae
Clostridium lactatifermentans AY033434 Clostridiaceae
Anaerotignum aminivorans AB298756 Lachnospiraceae
Clostridium populeti X71853 Clostridiaceae
Anaerotaenia torta AB298768 Lachnospiraceae
Clostridiales sp. XB90 AJ229234 NA
Blautia stercoris HM626177 Clostridiales
Blautia hansenii AB534168 Clostridiales
Blautia coccoides AB571656 Clostridiales
Blautia producta X94966 Clostridiales
Blautia luti AJ133124 Clostridiales
Blautia obeum X85101 Clostridiales
Blautia schinkii X94964 Clostridiales
Blautia schinkii X94965 Clostridiales
Ruminococcus gnavus X94967 Ruminococcaceae
Dorea longicatena AJ132842 Lachnospiraceae
Faecalimonas umbilicata LC192831 Lachnospiraceae
Eubacterium contortum FR749946 Eubacteriaceae
Clostridium glycyrrhizinilyticum AB233029 Clostridiaceae
Clostridium nexile X73443 Clostridiaceae
Coprococcus comes EF031542 Lachnospiraceae
Ruminococcus torques L76604 Ruminococcaceae
Faecalicatena orotica FR749921 Lachnospiraceae
Faecalicatena orotica FR749919 Lachnospiraceae
Clostridium oroticum FR749917 Clostridiaceae
Faecalicatena orotica FR749920 Lachnospiraceae
Eubacterium fissicatena GU985201 Eubacteriaceae
Eubacterium fissicatena FR749936 Eubacteriaceae
Faecalicatena contorta FR749945 Lachnospiraceae
Murimonas intestini KC311366 Lachnospiraceae
Falcatimonas natans AB298726 Lachnospiraceae
Ruminococcus gauvreauii EF529620 Ruminococcaceae
Clostridium lavalense EF564277 Clostridiaceae
Clostridium asparagiforme AJ582080 Clostridiaceae
Clostridium hathewayi AJ311620 Clostridiaceae
Kineothrix alysoides KX356505 Lachnospiraceae
Howardella ureilytica DQ925472 Clostridiales
Butyrivibrio crossotus ABWN01000012 Lachnospiraceae
Butyrivibrio crossotus FR733670 Lachnospiraceae
Eubacterium uniforme GU269550 Eubacteriaceae
Eubacterium oxidoreducens FR733672 Eubacteriaceae
Fusibacter paucivorans AF050099 Clostridiales
Fusibacter bizertensis KJ420408 Clostridiales
Anaeromicrobium sediminis KJ137882 Clostridiaceae
Marinisporobacter balticus KX822012 Clostridiales
Thermotalea metallivorans EU443727 Clostridiaceae
Salimesophilobacter vulgaris HQ880422 Clostridiaceae
Natronincola peptidivorans EF382661 Clostridiaceae
Natronincola ferrireducens EU878275 Clostridiaceae
Anoxynatronum sibiricum AF522323 Clostridiaceae
Anoxynatronum buryatiense EU315116 Clostridiaceae
Tindallia texcoconensis DQ234901 Clostridiaceae
Tindallia magadiensis Y15626 Clostridiaceae
Tindallia californiensis AF373919 Clostridiaceae
Alkaliphilus peptidifermentans EF382660 Clostridiaceae
Alkaliphilus namsaraevii KJ196388 Clostridiaceae
Alkaliphilus hydrothermalis KJ159210 Clostridiaceae
Alkaliphilus crotonatoxidans AF467248 Clostridiaceae
Alkaliphilus transvaalensis AB037677 Clostridiaceae
Filifactor alocis CP002390 Peptostreptococcaceae
Acetoanaerobium noterae GU562448 Clostridiales
Clostridium sticklandii FP565809 Clostridiaceae
Acetoanaerobium pronyense KJ174056 Clostridiales
Tepidibacter mesophilus GQ231514 Peptostreptococcaceae
Tepidibacter formicigenes AY245527 Peptostreptococcaceae
Tepidibacter thalassicus AY158079 Peptostreptococcaceae

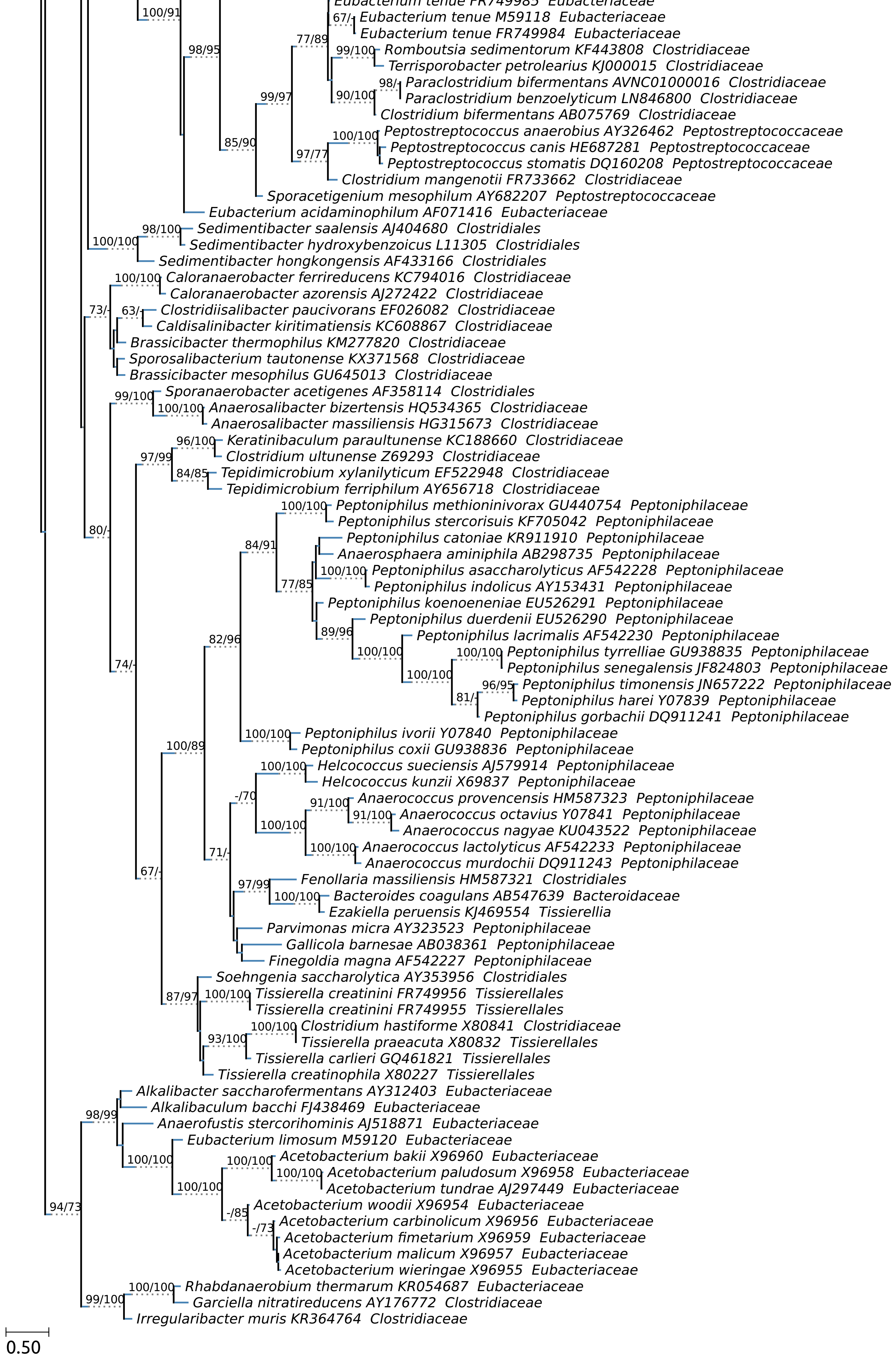

Eubacterium tenue FR749985 Eubacteriaceae
100/91
67/-
Eubacterium tenue M59118 Eubacteriaceae
Eubacterium tenue FR749984 Eubacteriaceae
77/89
98/95
99/100
Romboutsia sedimentorum KF443808 Clostridiaceae
Terrisporobacter petrolearius KJ000015 Clostridiaceae
98/-
Paraclostridium bifermentans AVNC01000016 Clostridiaceae
99/97
90/100
Paraclostridium benzoelyticum LN846800 Clostridiaceae
Clostridium bifermentans AB075769 Clostridiaceae
100/100
Peptostreptococcus anaerobius AY326462 Peptostreptococcaceae
85/90
Peptostreptococcus canis HE687281 Peptostreptococcaceae
97/77
Peptostreptococcus stomatis DQ160208 Peptostreptococcaceae
Clostridium mangenotii FR733662 Clostridiaceae
Sporacetigenium mesophilum AY682207 Peptostreptococcaceae
Eubacterium acidaminophilum AF071416 Eubacteriaceae
98/100
Sedimentibacter saalensis AJ404680 Clostridiales
100/100
Sedimentibacter hydroxybenzoicus L11305 Clostridiales
Sedimentibacter hongkongensis AF433166 Clostridiales
100/100
Caloranaerobacter ferrireducens KC794016 Clostridiaceae
Caloranaerobacter azorensis AJ272422 Clostridiaceae
73/-
63/-
Clostridiisalibacter paucivorans EF026082 Clostridiaceae
Caldisalinibacter kiritimatiensis KC608867 Clostridiaceae
Brassicibacter thermophilus KM277820 Clostridiaceae
Sporosalibacterium tautonense KX371568 Clostridiaceae
Brassicibacter mesophilus GU645013 Clostridiaceae
Sporanaerobacter acetigenes AF358114 Clostridiales
99/100
100/100
Anaerosalibacter bizertensis HQ534365 Clostridiaceae
Anaerosalibacter massiliensis HG315673 Clostridiaceae
96/100
Keratinibaculum paraultunense KC188660 Clostridiaceae
97/99
Clostridium ultunense Z69293 Clostridiaceae
84/85
Tepidimicrobium xylanilyticum EF522948 Clostridiaceae
Tepidimicrobium ferriphilum AY656718 Clostridiaceae
100/100
Peptoniphilus methioninivorax GU440754 Peptoniphilaceae
Peptoniphilus stercorisuis KF705042 Peptoniphilaceae
80/-
84/91
Peptoniphilus catoniae KR911910 Peptoniphilaceae
Anaerosphaera aminiphila AB298735 Peptoniphilaceae
100/100
Peptoniphilus asaccharolyticus AF542228 Peptoniphilaceae
77/85
Peptoniphilus indolicus AY153431 Peptoniphilaceae
Peptoniphilus koenoeneniae EU526291 Peptoniphilaceae
Peptoniphilus duerdenii EU526290 Peptoniphilaceae
82/96
89/96
Peptoniphilus lacrimalis AF542230 Peptoniphilaceae
100/100
100/100
Peptoniphilus tyrrelliae GU938835 Peptoniphilaceae
Peptoniphilus senegalensis JF824803 Peptoniphilaceae
74/-
100/100
96/95
Peptoniphilus timonensis JN657222 Peptoniphilaceae
81/-
Peptoniphilus harei Y07839 Peptoniphilaceae
Peptoniphilus gorbachii DQ911241 Peptoniphilaceae
100/100
Peptoniphilus ivorii Y07840 Peptoniphilaceae
100/89
Peptoniphilus coxii GU938836 Peptoniphilaceae
100/100
Helcococcus sueciensis AJ579914 Peptoniphilaceae
Helcococcus kunzii X69837 Peptoniphilaceae
-/70
91/100
Anaerococcus provencensis HM587323 Peptoniphilaceae
91/100
Anaerococcus octavius Y07841 Peptoniphilaceae
100/100
Anaerococcus nagyae KU043522 Peptoniphilaceae
71/-
100/100
Anaerococcus lactolyticus AF542233 Peptoniphilaceae
Anaerococcus murdochii DQ911243 Peptoniphilaceae
67/-
97/99
Fenollaria massiliensis HM587321 Clostridiales
100/100
Bacteroides coagulans AB547639 Bacteroidaceae
Ezakiella peruensis KJ469554 Tissierellia
Parvimonas micra AY323523 Peptoniphilaceae
Gallicola barnesae AB038361 Peptoniphilaceae
Finegoldia magna AF542227 Peptoniphilaceae
Soehngenia saccharolytica AY353956 Clostridiales
87/97
100/100
Tissierella creatinini FR749956 Tissierellales
Tissierella creatinini FR749955 Tissierellales
100/100
Clostridium hastiforme X80841 Clostridiaceae
93/100
Tissierella praeacuta X80832 Tissierellales
Tissierella carlieri GQ461821 Tissierellales
Tissierella creatinophila X80227 Tissierellales
Alkalibacter saccharofermentans AY312403 Eubacteriaceae
Alkalibaculum bacchi FJ438469 Eubacteriaceae
98/99
Anaerofustis stercorihominis AJ518871 Eubacteriaceae
Eubacterium limosum M59120 Eubacteriaceae
100/100
Acetobacterium bakii X96960 Eubacteriaceae
100/100
100/100
Acetobacterium paludosum X96958 Eubacteriaceae
100/100
Acetobacterium tundrae AJ297449 Eubacteriaceae
Acetobacterium woodii X96954 Eubacteriaceae
94/73
-/85
Acetobacterium carbinolicum X96956 Eubacteriaceae
-/73
Acetobacterium fimetarium X96959 Eubacteriaceae
Acetobacterium malicum X96957 Eubacteriaceae
Acetobacterium wieringae X96955 Eubacteriaceae
100/100
Rhabdanaerobium thermarum KR054687 Eubacteriaceae
99/100
Garciella nitratireducens AY176772 Clostridiaceae
Irregularibacter muris KR364764 Clostridiaceae
0.50

Figure 7: Unconstrained 16S rRNA gene ML tree, inferred under the GTR+CAT model, to resolve the placement of *Bacteroides coagulans* (in *Eubacteriales*). The branches are scaled in terms of the expected number of substitutions per site. The numbers above the branches are support values when larger than 60% from ML (left) and MP (right) bootstrapping. Dotted parts of branches are filled in to allow proper placement of bootstrap values and are not part of the actual branch length. Each tip label ends with the family of the respective taxon.

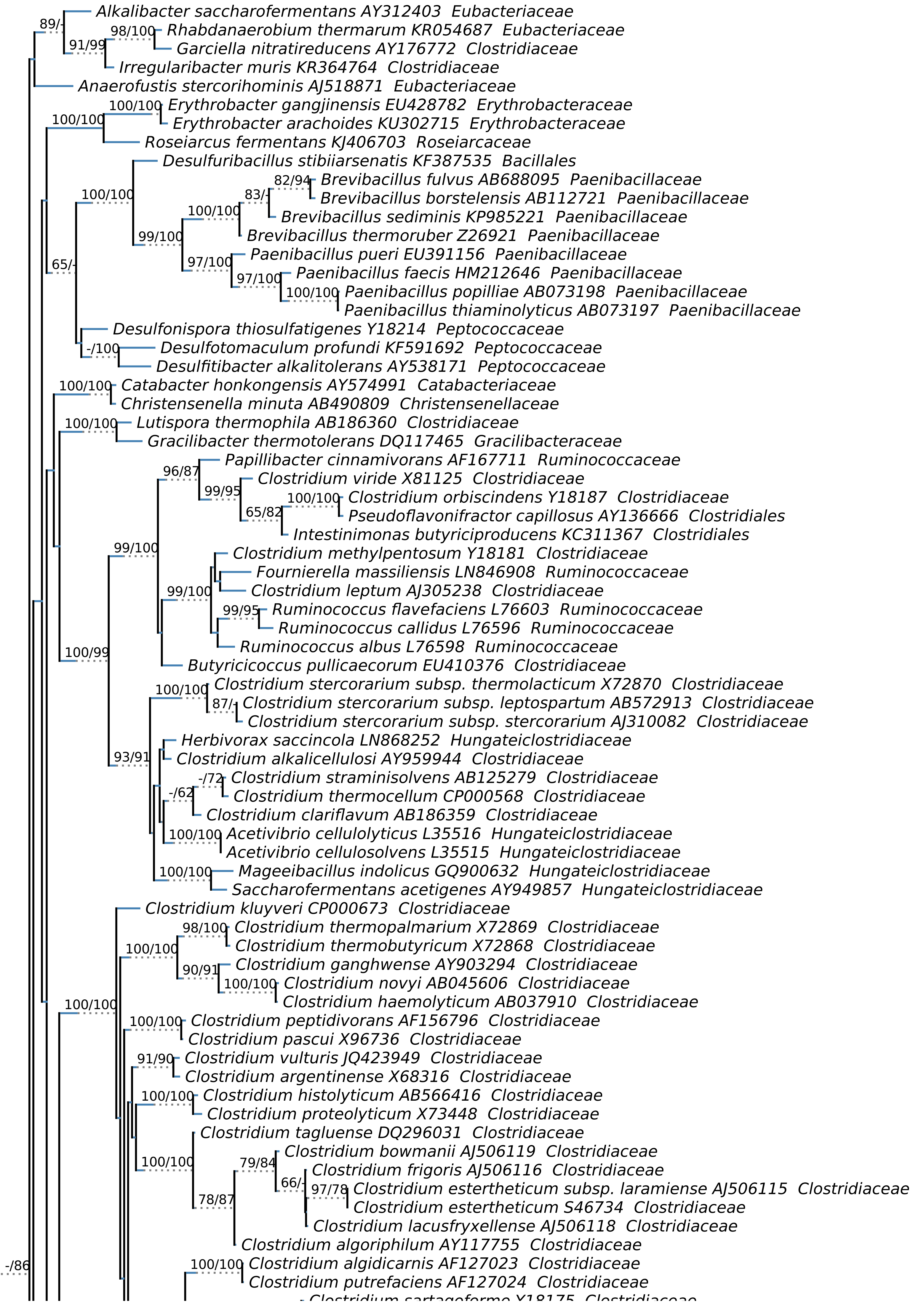
Alkalibacter saccharofermentans AY312403 Eubacteriaceae
Rhabdanaerobium thermarum KR054687 Eubacteriaceae
Garciella nitratireducens AY176772 Clostridiaceae
Irregularibacter muris KR364764 Clostridiaceae
Anaerofustis stercorihominis AJ518871 Eubacteriaceae
Erythrobacter gangjinensis EU428782 Erythrobacteraceae
Erythrobacter arachoides KU302715 Erythrobacteraceae
Roseiarcus fermentans KJ406703 Roseiarcaceae
Desulfuribacillus stibiiarsenatis KF387535 Bacillales
Brevibacillus fulvus AB688095 Paenibacillaceae
Brevibacillus borstelensis AB112721 Paenibacillaceae
Brevibacillus sediminis KP985221 Paenibacillaceae
Brevibacillus thermoruber Z26921 Paenibacillaceae
Paenibacillus pueri EU391156 Paenibacillaceae
Paenibacillus faecis HM212646 Paenibacillaceae
Paenibacillus popilliae AB073198 Paenibacillaceae
Paenibacillus thiaminolyticus AB073197 Paenibacillaceae
Desulfonispora thiosulfatigenes Y18214 Peptococcaceae
Desulfotomaculum profundi KF591692 Peptococcaceae
Desulfitibacter alkalitolerans AY538171 Peptococcaceae
Catabacter honkongensis AY574991 Catabacteriaceae
Christensenella minuta AB490809 Christensenellaceae
Lutispora thermophila AB186360 Clostridiaceae
Gracilibacter thermotolerans DQ117465 Gracilibacteraceae
Papillibacter cinnamivorans AF167711 Ruminococcaceae
Clostridium viride X81125 Clostridiaceae
Clostridium orbiscindens Y18187 Clostridiaceae
Pseudoflavonifractor capillosus AY136666 Clostridiales
Intestinimonas butyriciproducens KC311367 Clostridiales
Clostridium methylpentosum Y18181 Clostridiaceae
Fournierella massiliensis LN846908 Ruminococcaceae
Clostridium leptum AJ305238 Clostridiaceae
Ruminococcus flavefaciens L76603 Ruminococcaceae
Ruminococcus callidus L76596 Ruminococcaceae
Ruminococcus albus L76598 Ruminococcaceae
Butyricicoccus pullicaecorum EU410376 Clostridiaceae
Clostridium stercorarium subsp. thermolacticum X72870 Clostridiaceae
Clostridium stercorarium subsp. leptospartum AB572913 Clostridiaceae
Clostridium stercorarium subsp. stercorarium AJ310082 Clostridiaceae
Herbivorax saccincola LN868252 Hungateiclostridiaceae
Clostridium alkalicellulosi AY959944 Clostridiaceae
Clostridium straminisolvens AB125279 Clostridiaceae
Clostridium thermocellum CP000568 Clostridiaceae
Clostridium clariflavum AB186359 Clostridiaceae
Acetivibrio cellulolyticus L35516 Hungateiclostridiaceae
Acetivibrio cellulosolvens L35515 Hungateiclostridiaceae
Mageeibacillus indolicus GQ900632 Hungateiclostridiaceae
Saccharofermentans acetigenes AY949857 Hungateiclostridiaceae
Clostridium kluyveri CP000673 Clostridiaceae
Clostridium thermopalmarium X72869 Clostridiaceae
Clostridium thermobutyricum X72868 Clostridiaceae
Clostridium ganghwense AY903294 Clostridiaceae
Clostridium novyi AB045606 Clostridiaceae
Clostridium haemolyticum AB037910 Clostridiaceae
Clostridium peptidivorans AF156796 Clostridiaceae
Clostridium pascui X96736 Clostridiaceae
Clostridium vulturis JQ423949 Clostridiaceae
Clostridium argentinense X68316 Clostridiaceae
Clostridium histolyticum AB566416 Clostridiaceae
Clostridium proteolyticum X73448 Clostridiaceae
Clostridium tagluense DQ296031 Clostridiaceae
Clostridium bowmanii AJ506119 Clostridiaceae
Clostridium frigoris AJ506116 Clostridiaceae
Clostridium estertheticum subsp. laramiense AJ506115 Clostridiaceae
Clostridium estertheticum S46734 Clostridiaceae
Clostridium lacusfryxellense AJ506118 Clostridiaceae
Clostridium algoriphilum AY117755 Clostridiaceae
Clostridium algidicarnis AF127023 Clostridiaceae
Clostridium putrefaciens AF127024 Clostridiaceae

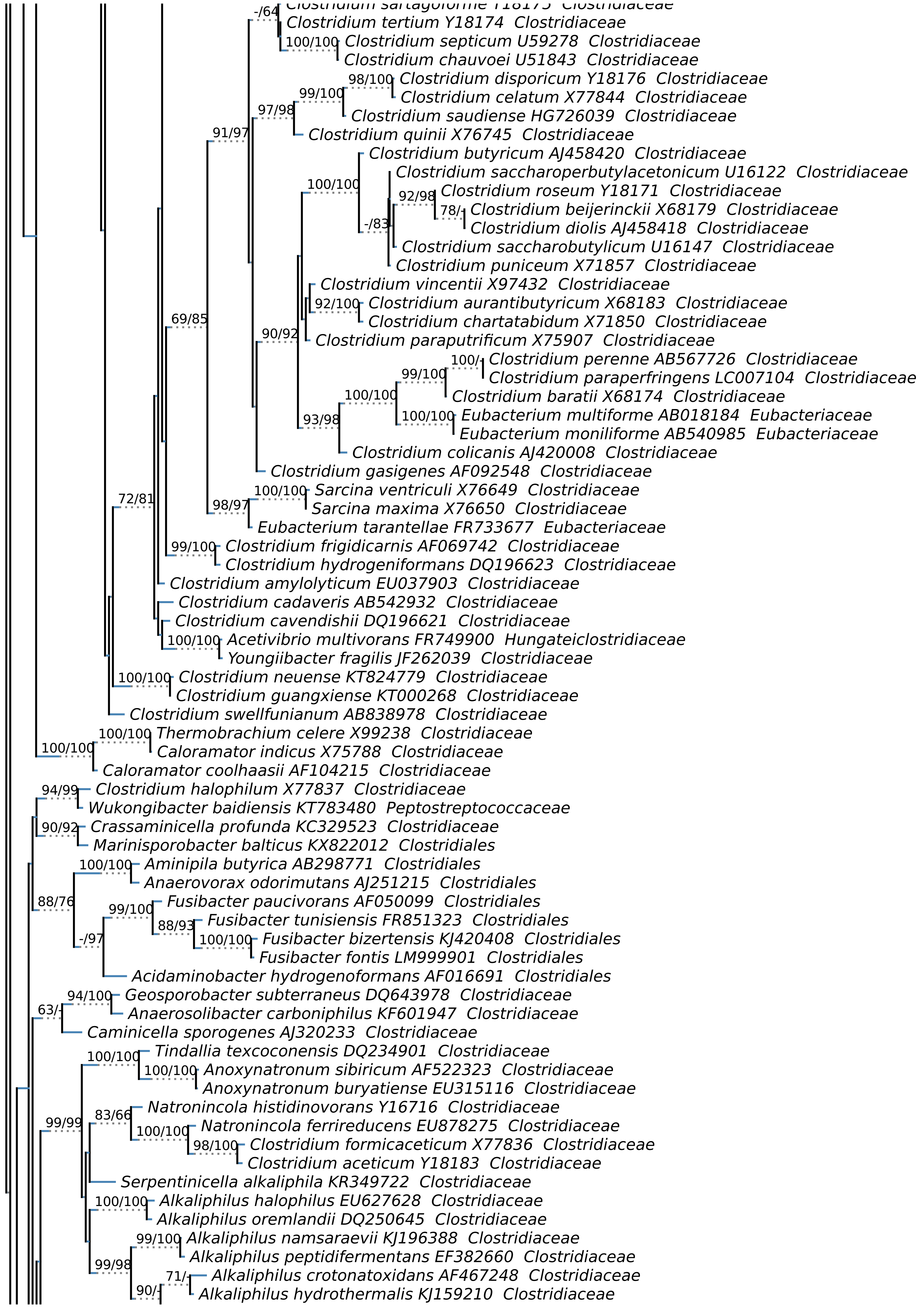

-/64
Clostridium tertium Y18174 Clostridiaceae
100/100
Clostridium septicum U59278 Clostridiaceae
Clostridium chauvoei U51843 Clostridiaceae
98/100
Clostridium disporicum Y18176 Clostridiaceae
99/100
Clostridium celatum X77844 Clostridiaceae
97/98
Clostridium saudiense HG726039 Clostridiaceae
91/97
Clostridium quinii X76745 Clostridiaceae
Clostridium butyricum AJ458420 Clostridiaceae
Clostridium saccharoperbutylacetonicum U16122 Clostridiaceae
100/100
92/98
Clostridium roseum Y18171 Clostridiaceae
78/-
Clostridium beijerinckii X68179 Clostridiaceae
-/83
Clostridium diolis AJ458418 Clostridiaceae
Clostridium saccharobutylicum U16147 Clostridiaceae
Clostridium puniceum X71857 Clostridiaceae
Clostridium vincentii X97432 Clostridiaceae
92/100
Clostridium aurantibutyricum X68183 Clostridiaceae
69/85
Clostridium chartatabidum X71850 Clostridiaceae
90/92
Clostridium paraputrificum X75907 Clostridiaceae
100/-
Clostridium perenne AB567726 Clostridiaceae
99/100
Clostridium paraperfringens LC007104 Clostridiaceae
100/100
Clostridium baratii X68174 Clostridiaceae
100/100
Eubacterium multiforme AB018184 Eubacteriaceae
93/98
Eubacterium moniliforme AB540985 Eubacteriaceae
Clostridium colicanis AJ420008 Clostridiaceae
Clostridium gasigenes AF092548 Clostridiaceae
100/100
Sarcina ventriculi X76649 Clostridiaceae
72/81
98/97
Sarcina maxima X76650 Clostridiaceae
Eubacterium tarantellae FR733677 Eubacteriaceae
99/100
Clostridium frigidicarnis AF069742 Clostridiaceae
Clostridium hydrogeniformans DQ196623 Clostridiaceae
Clostridium amylolyticum EU037903 Clostridiaceae
Clostridium cadaveris AB542932 Clostridiaceae
Clostridium cavendishii DQ196621 Clostridiaceae
100/100
Acetivibrio multivorans FR749900 Hungateiclostridiaceae
Youngiibacter fragilis JF262039 Clostridiaceae
100/100
Clostridium neuense KT824779 Clostridiaceae
Clostridium guangxiense KT000268 Clostridiaceae
Clostridium swellfunianum AB838978 Clostridiaceae
100/100
Thermobrachium celere X99238 Clostridiaceae
100/100
Caloramator indicus X75788 Clostridiaceae
Caloramator coolhaasii AF104215 Clostridiaceae
94/99
Clostridium halophilum X77837 Clostridiaceae
Wukongibacter baidiensis KT783480 Peptostreptococcaceae
90/92
Crassaminicella profunda KC329523 Clostridiaceae
Marinisporobacter balticus KX822012 Clostridiales
100/100
Aminipila butyrica AB298771 Clostridiales
Anaerovorax odorimutans AJ251215 Clostridiales
88/76
Fusibacter paucivorans AF050099 Clostridiales
99/100
88/93
Fusibacter tunisiensis FR851323 Clostridiales
-/97
100/100
Fusibacter bizertensis KJ420408 Clostridiales
Fusibacter fontis LM999901 Clostridiales
Acidaminobacter hydrogenoformans AF016691 Clostridiales
94/100
Geosporobacter subterraneus DQ643978 Clostridiaceae
63/-
Anaerosolibacter carboniphilus KF601947 Clostridiaceae
Caminicella sporogenes AJ320233 Clostridiaceae
100/100
Tindallia texcoconensis DQ234901 Clostridiaceae
100/100
Anoxynatronum sibiricum AF522323 Clostridiaceae
Anoxynatronum buryatiense EU315116 Clostridiaceae
Natronincola histidinovorans Y16716 Clostridiaceae
99/99
83/66
100/100
Natronincola ferrireducens EU878275 Clostridiaceae
98/100
Clostridium formicaceticum X77836 Clostridiaceae
Clostridium aceticum Y18183 Clostridiaceae
Serpentinicella alkaliphila KR349722 Clostridiaceae
100/100
Alkaliphilus halophilus EU627628 Clostridiaceae
Alkaliphilus oremlandii DQ250645 Clostridiaceae
99/100
Alkaliphilus namsaraevii KJ196388 Clostridiaceae
99/98
Alkaliphilus peptidifermentans EF382660 Clostridiaceae
71/-
Alkaliphilus crotonatoxidans AF467248 Clostridiaceae
90/-
Alkaliphilus hydrothermalis KJ159210 Clostridiaceae

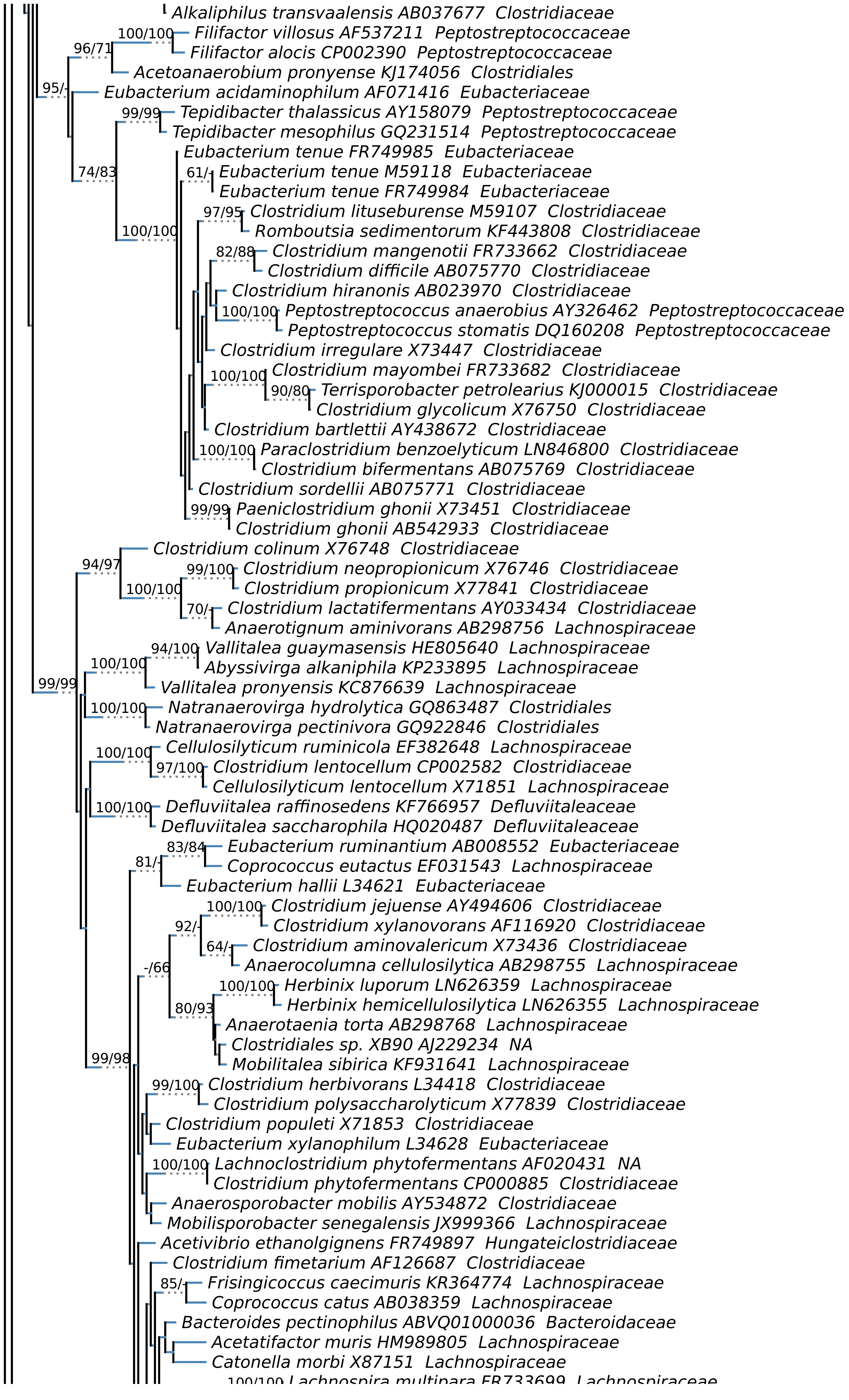

Alkaliphilus transvaalensis AB037677 Clostridiaceae
100/100
Filifactor villosus AF537211 Peptostreptococcaceae
96/71
Filifactor alocis CP002390 Peptostreptococcaceae
Acetoanaerobium pronyense KJ174056 Clostridiales
95/-
Eubacterium acidaminophilum AF071416 Eubacteriaceae
99/99
Tepidibacter thalassicus AY158079 Peptostreptococcaceae
Tepidibacter mesophilus GQ231514 Peptostreptococcaceae
Eubacterium tenue FR749985 Eubacteriaceae
74/83
61/-
Eubacterium tenue M59118 Eubacteriaceae
Eubacterium tenue FR749984 Eubacteriaceae
97/95
Clostridium lituseburense M59107 Clostridiaceae
100/100
Romboutsia sedimentorum KF443808 Clostridiaceae
82/88
Clostridium mangenotii FR733662 Clostridiaceae
Clostridium difficile AB075770 Clostridiaceae
Clostridium hiranonis AB023970 Clostridiaceae
100/100
Peptostreptococcus anaerobius AY326462 Peptostreptococcaceae
Peptostreptococcus stomatis DQ160208 Peptostreptococcaceae
Clostridium irregulare X73447 Clostridiaceae
100/100
Clostridium mayombei FR733682 Clostridiaceae
90/80
Terrisporobacter petrolearius KJ000015 Clostridiaceae
Clostridium glycolicum X76750 Clostridiaceae
Clostridium bartlettii AY438672 Clostridiaceae
100/100
Paraclostridium benzoelyticum LN846800 Clostridiaceae
Clostridium bifermentans AB075769 Clostridiaceae
Clostridium sordellii AB075771 Clostridiaceae
99/99
Paeniclostridium ghonii X73451 Clostridiaceae
Clostridium ghonii AB542933 Clostridiaceae
Clostridium colinum X76748 Clostridiaceae
94/97
99/100
Clostridium neopropionicum X76746 Clostridiaceae
100/100
Clostridium propionicum X77841 Clostridiaceae
70/-
Clostridium lactatifermentans AY033434 Clostridiaceae
Anaerotignum aminivorans AB298756 Lachnospiraceae
94/100
Vallitalea guaymasensis HE805640 Lachnospiraceae
100/100
Abyssivirga alkaniphila KP233895 Lachnospiraceae
99/99
Vallitalea pronyensis KC876639 Lachnospiraceae
100/100
Natranaerovirga hydrolytica GQ863487 Clostridiales
Natranaerovirga pectinivora GQ922846 Clostridiales
100/100
Cellulosilyticum ruminicola EF382648 Lachnospiraceae
97/100
Clostridium lentocellum CP002582 Clostridiaceae
Cellulosilyticum lentocellum X71851 Lachnospiraceae
100/100
Defluviitalea raffinosedens KF766957 Defluviitaleaceae
Defluviitalea saccharophila HQ020487 Defluviitaleaceae
83/84
Eubacterium ruminantium AB008552 Eubacteriaceae
81/-
Coprococcus eutactus EF031543 Lachnospiraceae
Eubacterium hallii L34621 Eubacteriaceae
100/100
Clostridium jejuense AY494606 Clostridiaceae
92/-
Clostridium xylanovorans AF116920 Clostridiaceae
64/-
Clostridium aminovalericum X73436 Clostridiaceae
-/66
Anaerocolumna cellulosilytica AB298755 Lachnospiraceae
100/100
Herbinix luporum LN626359 Lachnospiraceae
80/93
Herbinix hemicellulosilytica LN626355 Lachnospiraceae
Anaerotaenia torta AB298768 Lachnospiraceae
Clostridiales sp. XB90 AJ229234 NA
Mobilitalea sibirica KF931641 Lachnospiraceae
99/98
99/100
Clostridium herbivorans L34418 Clostridiaceae
Clostridium polysaccharolyticum X77839 Clostridiaceae
Clostridium populeti X71853 Clostridiaceae
Eubacterium xylanophilum L34628 Eubacteriaceae
100/100
Lachnoclostridium phytofermentans AF020431 NA
Clostridium phytofermentans CP000885 Clostridiaceae
Anaerosporobacter mobilis AY534872 Clostridiaceae
Mobilisporobacter senegalensis JX999366 Lachnospiraceae
Acetivibrio ethanolgignens FR749897 Hungateiclostridiaceae
Clostridium fimetarium AF126687 Clostridiaceae
85/-
Frisingicoccus caecimuris KR364774 Lachnospiraceae
Coprococcus catus AB038359 Lachnospiraceae
Bacteroides pectinophilus ABVQ01000036 Bacteroidaceae
Acetatifactor muris HM989805 Lachnospiraceae
Catonella morbi X87151 Lachnospiraceae

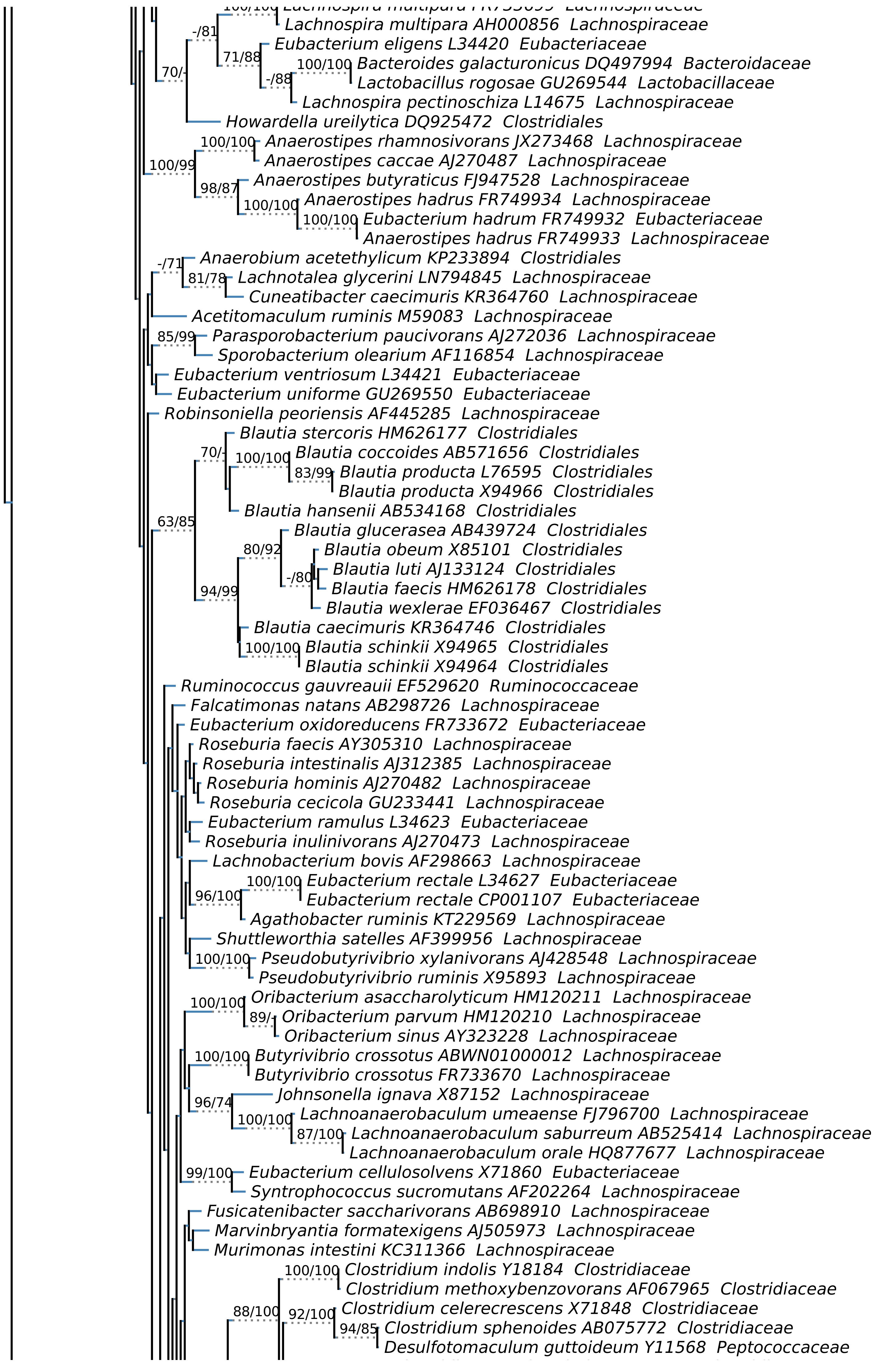
Lachnospira multipara AH000856 Lachnospiraceae
-/81
Eubacterium eligens L34420 Eubacteriaceae
71/88
100/100 Bacteroides galacturonicus DQ497994 Bacteroidaceae
70/-
-/88
Lactobacillus rogosae GU269544 Lactobacillaceae
Lachnospira pectinoschiza L14675 Lachnospiraceae
Howardella ureilytica DQ925472 Clostridiales
100/100 Anaerostipes rhamnosivorans JX273468 Lachnospiraceae
100/99
Anaerostipes caccae AJ270487 Lachnospiraceae
Anaerostipes butyraticus FJ947528 Lachnospiraceae
98/87
100/100 Anaerostipes hadrus FR749934 Lachnospiraceae
100/100 Eubacterium hadrum FR749932 Eubacteriaceae
Anaerostipes hadrus FR749933 Lachnospiraceae
-/71
Anaerobium acetethylicum KP233894 Clostridiales
81/78 Lachnotalea glycerini LN794845 Lachnospiraceae
Cuneatibacter caecimuris KR364760 Lachnospiraceae
Acetitomaculum ruminis M59083 Lachnospiraceae
85/99 Parasporobacterium paucivorans AJ272036 Lachnospiraceae
Sporobacterium olearium AF116854 Lachnospiraceae
Eubacterium ventriosum L34421 Eubacteriaceae
Eubacterium uniforme GU269550 Eubacteriaceae
Robinsoniella peoriensis AF445285 Lachnospiraceae
Blautia stercoris HM626177 Clostridiales
70/-
100/100 Blautia coccoides AB571656 Clostridiales
83/99 Blautia producta L76595 Clostridiales
Blautia producta X94966 Clostridiales
Blautia hansenii AB534168 Clostridiales
63/85
Blautia glucerasea AB439724 Clostridiales
80/92
Blautia obeum X85101 Clostridiales
-/80
Blautia luti AJ133124 Clostridiales
Blautia faecis HM626178 Clostridiales
94/99
Blautia wexlerae EF036467 Clostridiales
Blautia caecimuris KR364746 Clostridiales
100/100 Blautia schinkii X94965 Clostridiales
Blautia schinkii X94964 Clostridiales
Ruminococcus gauvreauii EF529620 Ruminococcaceae
Falcatimonas natans AB298726 Lachnospiraceae
Eubacterium oxidoreducens FR733672 Eubacteriaceae
Roseburia faecis AY305310 Lachnospiraceae
Roseburia intestinalis AJ312385 Lachnospiraceae
Roseburia hominis AJ270482 Lachnospiraceae
Roseburia cecicola GU233441 Lachnospiraceae
Eubacterium ramulus L34623 Eubacteriaceae
Roseburia inulinivorans AJ270473 Lachnospiraceae
Lachnobacterium bovis AF298663 Lachnospiraceae
100/100 Eubacterium rectale L34627 Eubacteriaceae
96/100
Eubacterium rectale CP001107 Eubacteriaceae
Agathobacter ruminis KT229569 Lachnospiraceae
Shuttleworthia satelles AF399956 Lachnospiraceae
100/100 Pseudobutyrivibrio xylanivorans AJ428548 Lachnospiraceae
Pseudobutyrivibrio ruminis X95893 Lachnospiraceae
100/100 Oribacterium asaccharolyticum HM120211 Lachnospiraceae
89/- Oribacterium parvum HM120210 Lachnospiraceae
Oribacterium sinus AY323228 Lachnospiraceae
100/100 Butyrivibrio crossotus ABWN01000012 Lachnospiraceae
Butyrivibrio crossotus FR733670 Lachnospiraceae
Johnsonella ignava X87152 Lachnospiraceae
96/74
100/100 Lachnoanaerobaculum umeaense FJ796700 Lachnospiraceae
87/100 Lachnoanaerobaculum saburreum AB525414 Lachnospiraceae
Lachnoanaerobaculum orale HQ877677 Lachnospiraceae
99/100 Eubacterium cellulosolvens X71860 Eubacteriaceae
Syntrophococcus sucromutans AF202264 Lachnospiraceae
Fusicatenibacter saccharivorans AB698910 Lachnospiraceae
Marvinbryantia formatexigens AJ505973 Lachnospiraceae
Murimonas intestini KC311366 Lachnospiraceae
100/100 Clostridium indolis Y18184 Clostridiaceae
Clostridium methoxybenzovorans AF067965 Clostridiaceae
88/100
92/100 Clostridium celerecrescens X71848 Clostridiaceae
94/85 Clostridium sphenoides AB075772 Clostridiaceae
Desulfotomaculum guttoideum Y11568 Peptococcaceae

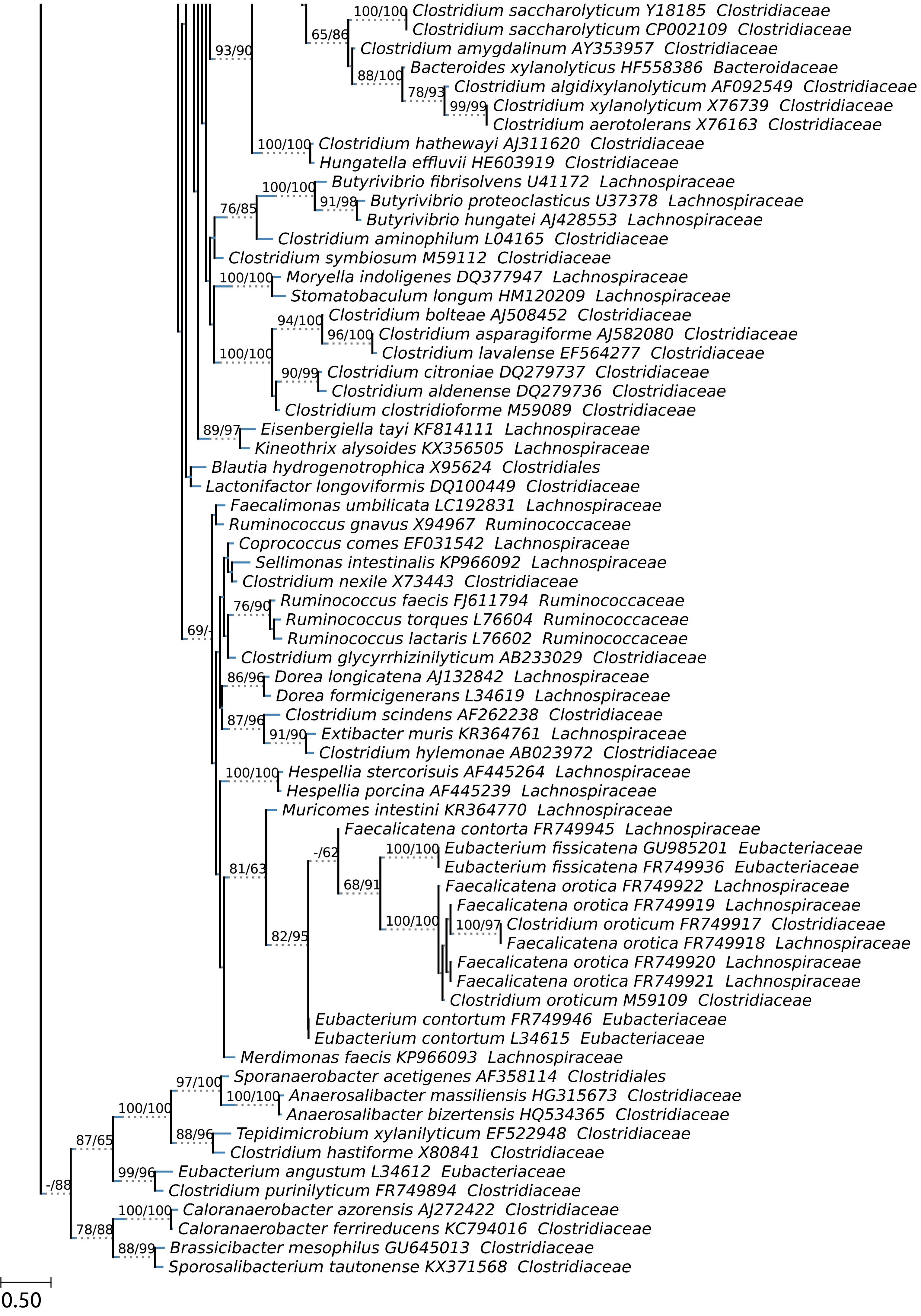

Clostridium saccharolyticum Y18185 Clostridiaceae
Clostridium saccharolyticum CP002109 Clostridiaceae
Clostridium amygdalinum AY353957 Clostridiaceae
Bacteroides xylanolyticus HF558386 Bacteroidaceae
Clostridium algidixylanolyticum AF092549 Clostridiaceae
Clostridium xylanolyticum X76739 Clostridiaceae
Clostridium aerotolerans X76163 Clostridiaceae
Clostridium hathewayi AJ311620 Clostridiaceae
Hungatella effluvii HE603919 Clostridiaceae
Butyrivibrio fibrisolvens U41172 Lachnospiraceae
Butyrivibrio proteoclasticus U37378 Lachnospiraceae
Butyrivibrio hungatei AJ428553 Lachnospiraceae
Clostridium aminophilum L04165 Clostridiaceae
Clostridium symbiosum M59112 Clostridiaceae
Moryella indoligenes DQ377947 Lachnospiraceae
Stomatobaculum longum HM120209 Lachnospiraceae
Clostridium bolteae AJ508452 Clostridiaceae
Clostridium asparagiforme AJ582080 Clostridiaceae
Clostridium lavalense EF564277 Clostridiaceae
Clostridium citroniae DQ279737 Clostridiaceae
Clostridium aldenense DQ279736 Clostridiaceae
Clostridium clostridioforme M59089 Clostridiaceae
Eisenbergiella tayi KF814111 Lachnospiraceae
Kineothrix alysoides KX356505 Lachnospiraceae
Blautia hydrogenotrophica X95624 Clostridiales
Lactonifactor longoviformis DQ100449 Clostridiaceae
Faecalimonas umbilicata LC192831 Lachnospiraceae
Ruminococcus gnavus X94967 Ruminococcaceae
Coprococcus comes EF031542 Lachnospiraceae
Sellimonas intestinalis KP966092 Lachnospiraceae
Clostridium nexile X73443 Clostridiaceae
Ruminococcus faecis FJ611794 Ruminococcaceae
Ruminococcus torques L76604 Ruminococcaceae
Ruminococcus lactaris L76602 Ruminococcaceae
Clostridium glycyrrhizinilyticum AB233029 Clostridiaceae
Dorea longicatena AJ132842 Lachnospiraceae
Dorea formicigenerans L34619 Lachnospiraceae
Clostridium scindens AF262238 Clostridiaceae
Extibacter muris KR364761 Lachnospiraceae
Clostridium hylemonae AB023972 Clostridiaceae
Hespellia stercorisuis AF445264 Lachnospiraceae
Hespellia porcina AF445239 Lachnospiraceae
Muricomes intestini KR364770 Lachnospiraceae
Faecalicatena contorta FR749945 Lachnospiraceae
Eubacterium fissicatena GU985201 Eubacteriaceae
Eubacterium fissicatena FR749936 Eubacteriaceae
Faecalicatena orotica FR749922 Lachnospiraceae
Faecalicatena orotica FR749919 Lachnospiraceae
Clostridium oroticum FR749917 Clostridiaceae
Faecalicatena orotica FR749918 Lachnospiraceae
Faecalicatena orotica FR749920 Lachnospiraceae
Faecalicatena orotica FR749921 Lachnospiraceae
Clostridium oroticum M59109 Clostridiaceae
Eubacterium contortum FR749946 Eubacteriaceae
Eubacterium contortum L34615 Eubacteriaceae
Merdimonas faecis KP966093 Lachnospiraceae
Sporanaerobacter acetigenes AF358114 Clostridiales
Anaerosalibacter massiliensis HG315673 Clostridiaceae
Anaerosalibacter bizertensis HQ534365 Clostridiaceae
Tepidimicrobium xylanilyticum EF522948 Clostridiaceae
Clostridium hastiforme X80841 Clostridiaceae
Eubacterium angustum L34612 Eubacteriaceae
Clostridium purinilyticum FR749894 Clostridiaceae
Caloranaerobacter azorensis AJ272422 Clostridiaceae
Caloranaerobacter ferrireducens KC794016 Clostridiaceae
Brassicibacter mesophilus GU645013 Clostridiaceae
Sporosalibacterium tautonense KX371568 Clostridiaceae
0.50

Figure 8: Unconstrained 16S rRNA gene ML tree, inferred under the GTR+CAT model, to resolve the placement of the three other orphan *Bacteroides* spp. (in *Eubacteriales*). The branches are scaled in terms of the expected number of substitutions per site. The numbers above the branches are support values when larger than 60% from ML (left) and MP (right) bootstrapping. Dotted parts of branches are filled in to allow proper placement of bootstrap values and are not part of the actual branch length. Each tip label ends with the family of the respective taxon.

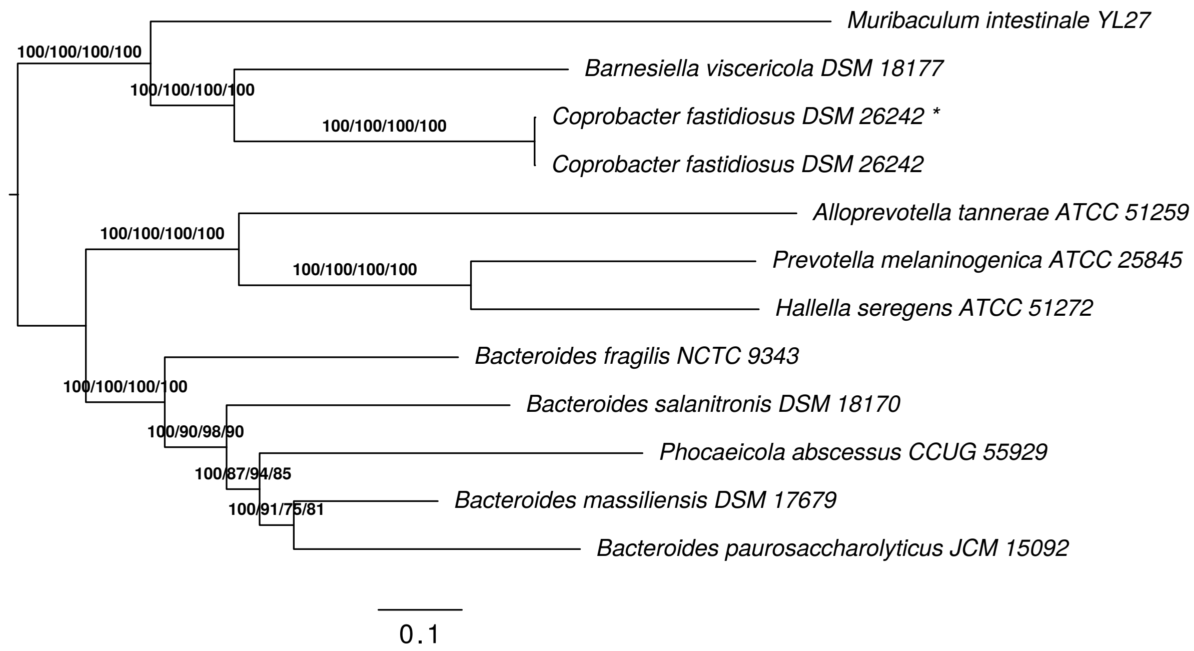

Figure 9: Phylogenetic tree inferred with RAxML from the *Bacteroides* supermatrix including single-copy core genes. Branches are scaled in terms of the expected number of changes per site. The first two numbers above branches (left to right) are partition bootstrap support values from (i) RAxML (ML) analysis and (ii) TNT (MP) analysis of the supermatrix that included single-copy genes that occurred in all of the genomes. The last two numbers above branches are partition bootstrap support values from (iii) RAxML (ML) analysis and (iv) TNT (MP) analysis of the supermatrix that included single-copy genes that occurred in at least four of the genomes.

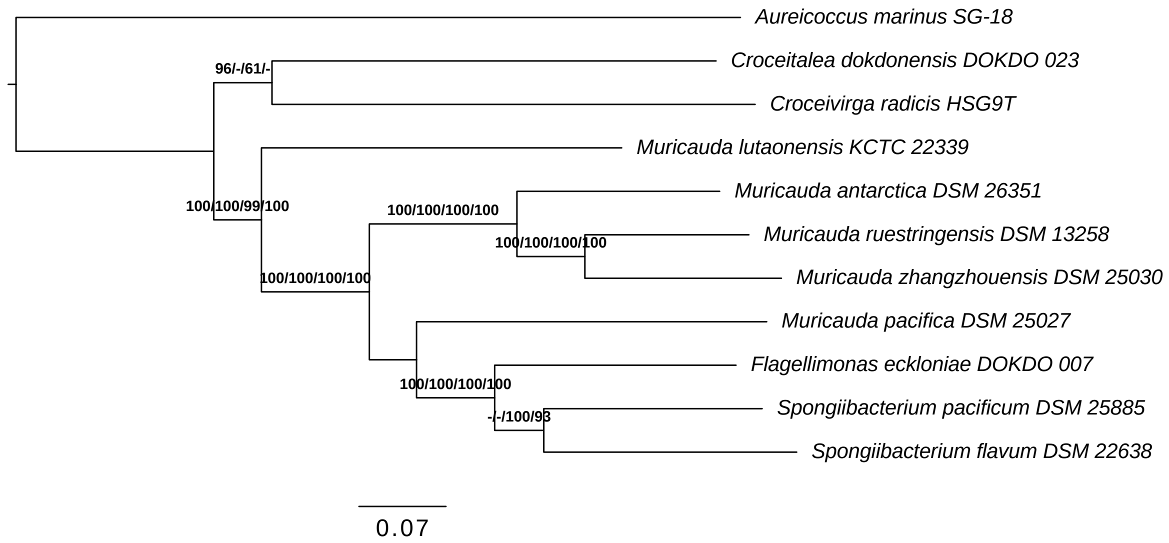

Figure 10: Phylogenetic ML tree inferred with RAxML from the *Croceitalea* supermatrix including single-copy core genes. Branches are scaled in terms of the expected number of changes per site. The first two numbers above branches (left to right) are partition bootstrap support values from (i) RAxML (ML) analysis and (ii) TNT (MP) analysis of the supermatrix that included single-copy genes that occurred in all of the genomes. The last two numbers above branches are partition bootstrap support values from (iii) RAxML (ML) analysis and (iv) TNT (MP) analysis of the supermatrix that included single-copy genes that occurred in at least four of the genomes.

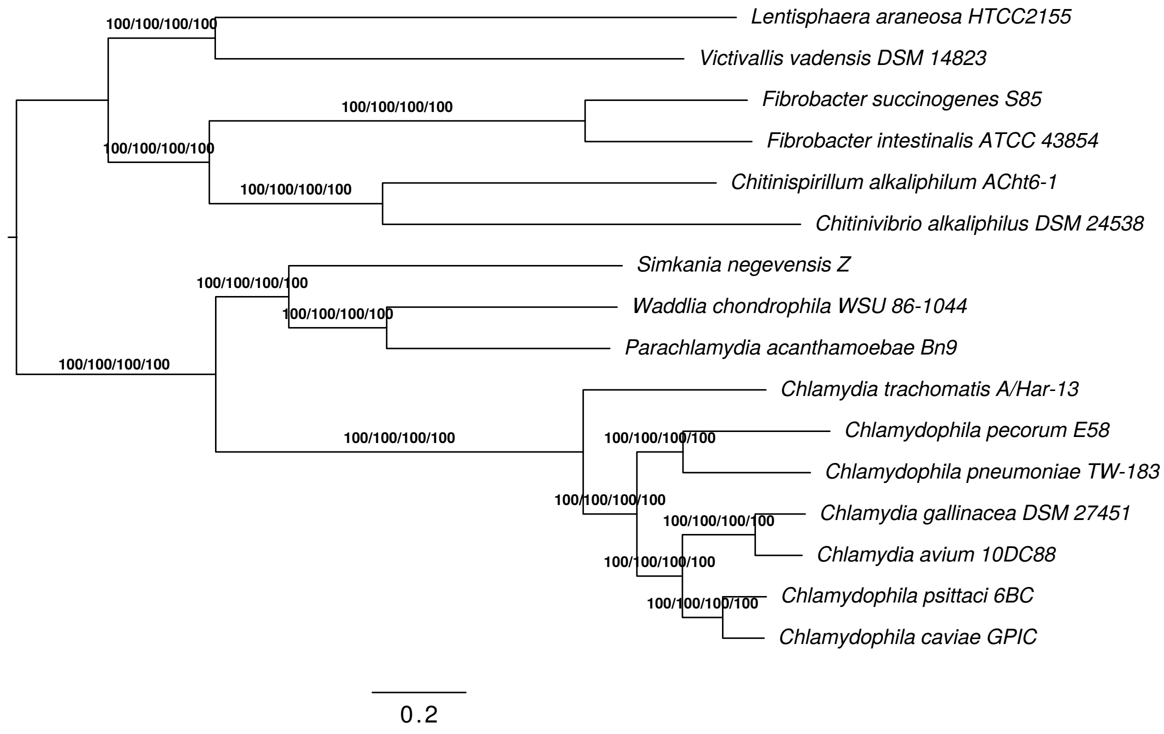

Figure 11: Phylogenetic ML tree inferred with RAxML from the *Fibrobacteres* supermatrix including single-copy core genes. Branches are scaled in terms of the expected number of changes per site. The first two numbers above branches (left to right) are partition bootstrap support values from (i) RAxML (ML) analysis and (ii) TNT (MP) analysis of the supermatrix that included single-copy genes that occurred in all of the genomes. The last two numbers above branches are partition bootstrap support values from (iii) RAxML (ML) analysis and (iv) TNT (MP) analysis of the supermatrix that included single-copy genes that occurred in at least four of the genomes.

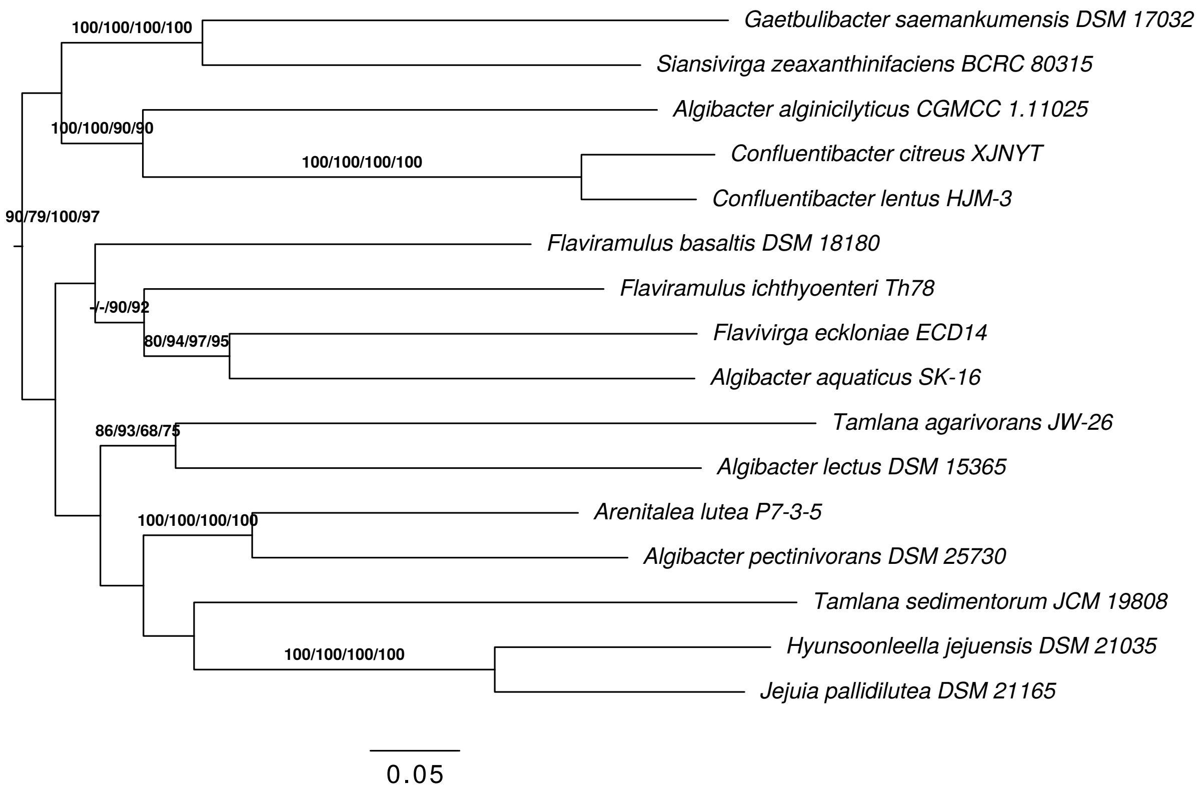

Figure 12: Phylogenetic ML tree inferred with RAxML from the *Flaviramulus* supermatrix including all single-copy genes that occured in at least four of the genomes. Branches are scaled in terms of the expected number of changes per site. The first two numbers above branches (left to right) are partition bootstrap support values from (i) RAxML (ML) analysis and (ii) TNT (MP) analysis of the supermatrix that included single-copy genes that occurred in all of the genomes. The last two numbers above branches are partition bootstrap support values from (iii) RAxML (ML) analysis and (iv) TNT (MP) analysis of the supermatrix that included single-copy genes that occurred in at least four of the genomes.

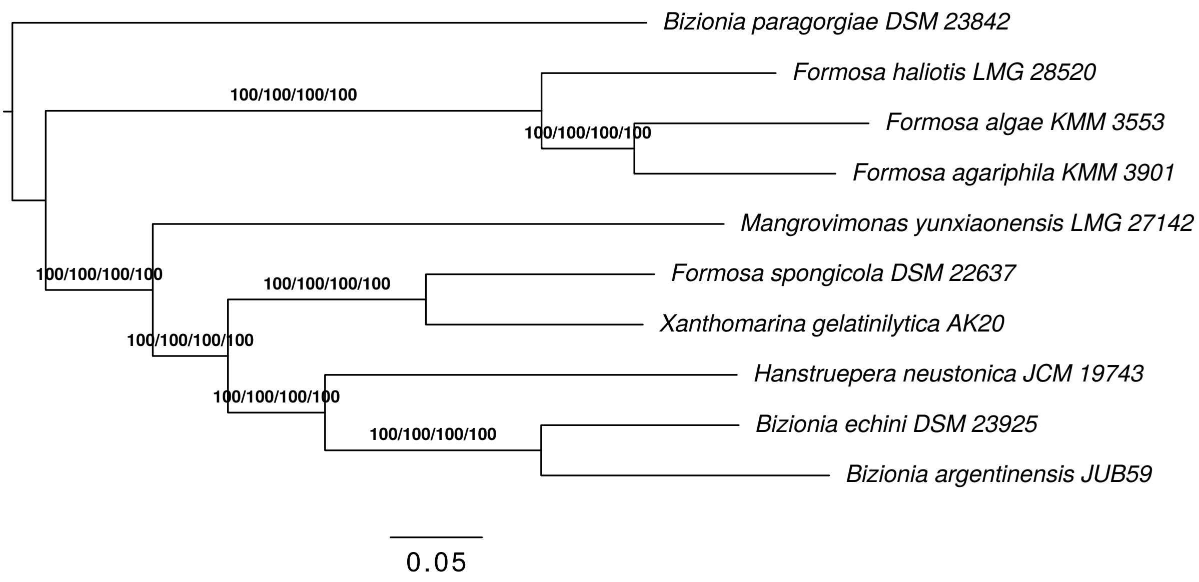

Figure 13: Phylogenetic ML tree inferred with RAxML from the *Formosa* supermatrix including all single-copy genes that occured in at least four of the genomes. Branches are scaled in terms of the expected number of changes per site. The first two numbers above branches (left to right) are partition bootstrap support values from (i) RAxML (ML) analysis and (ii) TNT (MP) analysis of the supermatrix that included single-copy genes that occurred in all of the genomes. The last two numbers above branches are partition bootstrap support values from (iii) RAxML (ML) analysis and (iv) TNT (MP) analysis of the supermatrix that included single-copy genes that occurred in at least four of the genomes.

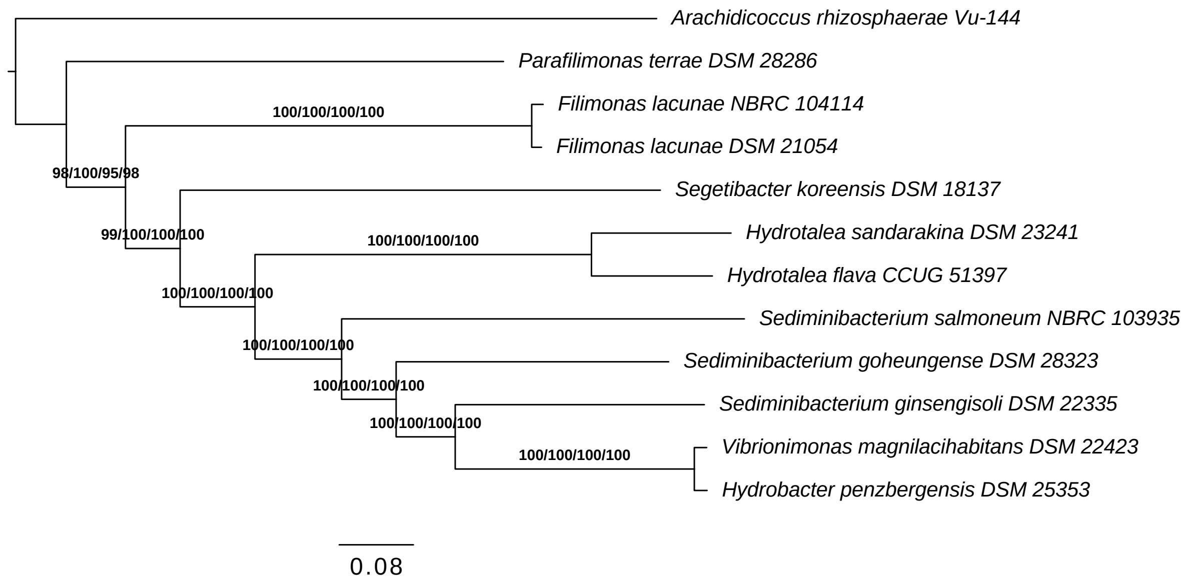

Figure 14: Phylogenetic ML tree inferred with RAxML from the *Hydrobacter* supermatrix including all single-copy genes that occured in at least four of the genomes. Branches are scaled in terms of the expected number of changes per site. The first two numbers above branches (left to right) are partition bootstrap support values from (i) RAxML (ML) analysis and (ii) TNT (MP) analysis of the supermatrix that included single-copy genes that occurred in all of the genomes. The last two numbers above branches are partition bootstrap support values from (iii) RAxML (ML) analysis and (iv) TNT (MP) analysis of the supermatrix that included single-copy genes that occurred in at least four of the genomes.

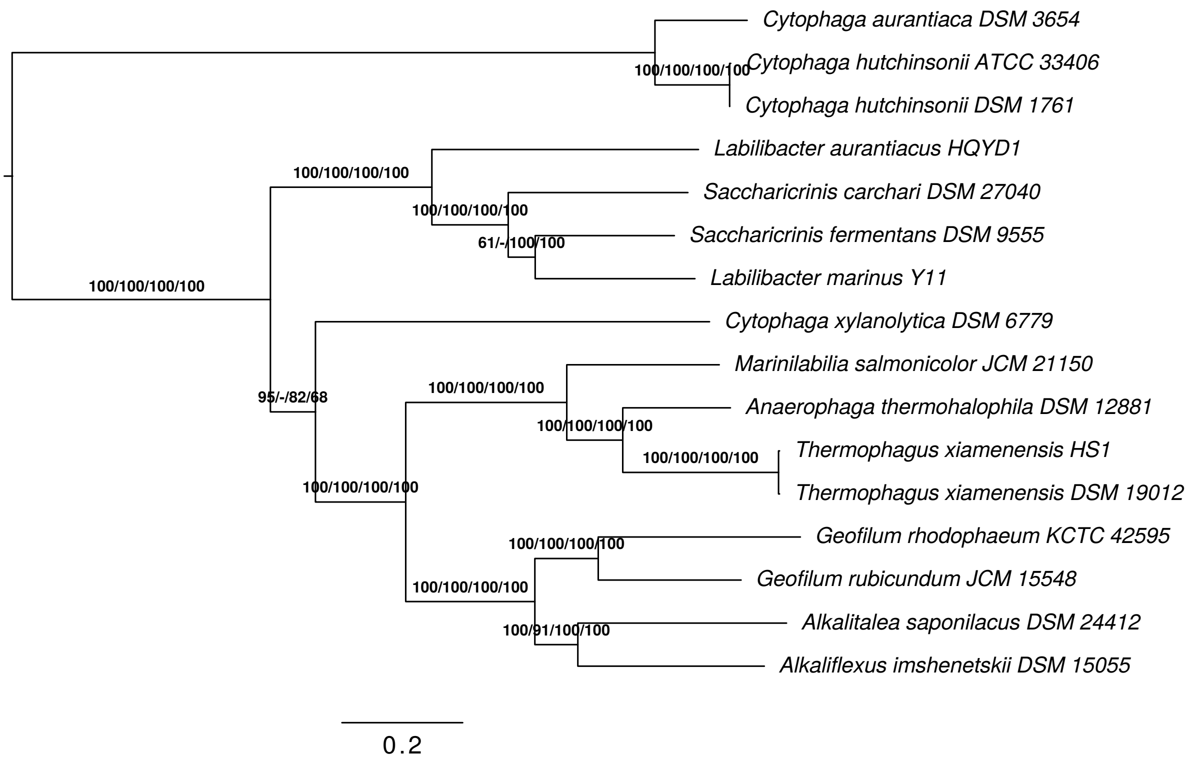

Figure 15: Phylogenetic ML tree inferred with RAxML from the *Labilibacter* supermatrix including all single-copy genes that occured in at least four of the genomes. Branches are scaled in terms of the expected number of changes per site. The first two numbers above branches (left to right) are partition bootstrap support values from (i) RAxML (ML) analysis and (ii) TNT (MP) analysis of the supermatrix that included single-copy genes that occurred in all of the genomes. The last two numbers above branches are partition bootstrap support values from (iii) RAxML (ML) analysis and (iv) TNT (MP) analysis of the supermatrix that included single-copy genes that occurred in at least four of the genomes.

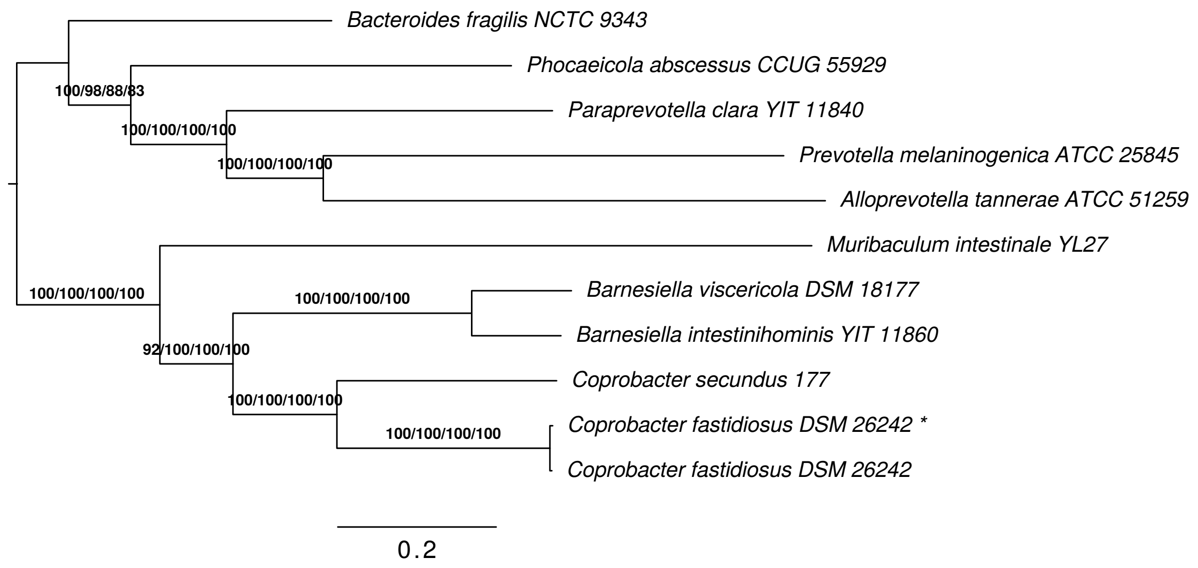

Figure 16: Phylogenetic ML tree inferred with RAxML from the *Muribaculum* supermatrix including all single-copy genes that occured in at least four of the genomes. Branches are scaled in terms of the expected number of changes per site. The first two numbers above branches (left to right) are partition bootstrap support values from (i) RAxML (ML) analysis and (ii) TNT (MP) analysis of the supermatrix that included single-copy genes that occurred in all of the genomes. The last two numbers above branches are partition bootstrap support values from (iii) RAxML (ML) analysis and (iv) TNT (MP) analysis of the supermatrix that included single-copy genes that occurred in at least four of the genomes.

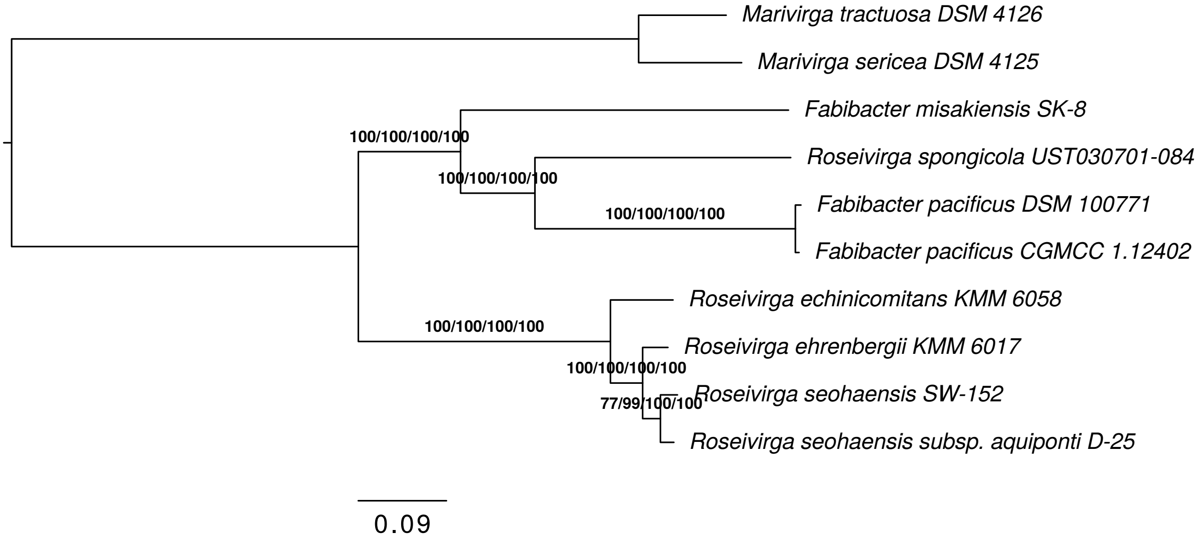

Figure 17: Phylogenetic ML tree inferred with RAxML from the *Roseivirga* supermatrix including all single-copy genes that occured in at least four of the genomes. Branches are scaled in terms of the expected number of changes per site. The first two numbers above branches (left to right) are partition bootstrap support values from (i) RAxML (ML) analysis and (ii) TNT (MP) analysis of the supermatrix that included single-copy genes that occurred in all of the genomes. The last two numbers above branches are partition bootstrap support values from (iii) RAxML (ML) analysis and (iv) TNT (MP) analysis of the supermatrix that included single-copy genes that occurred in at least four of the genomes.

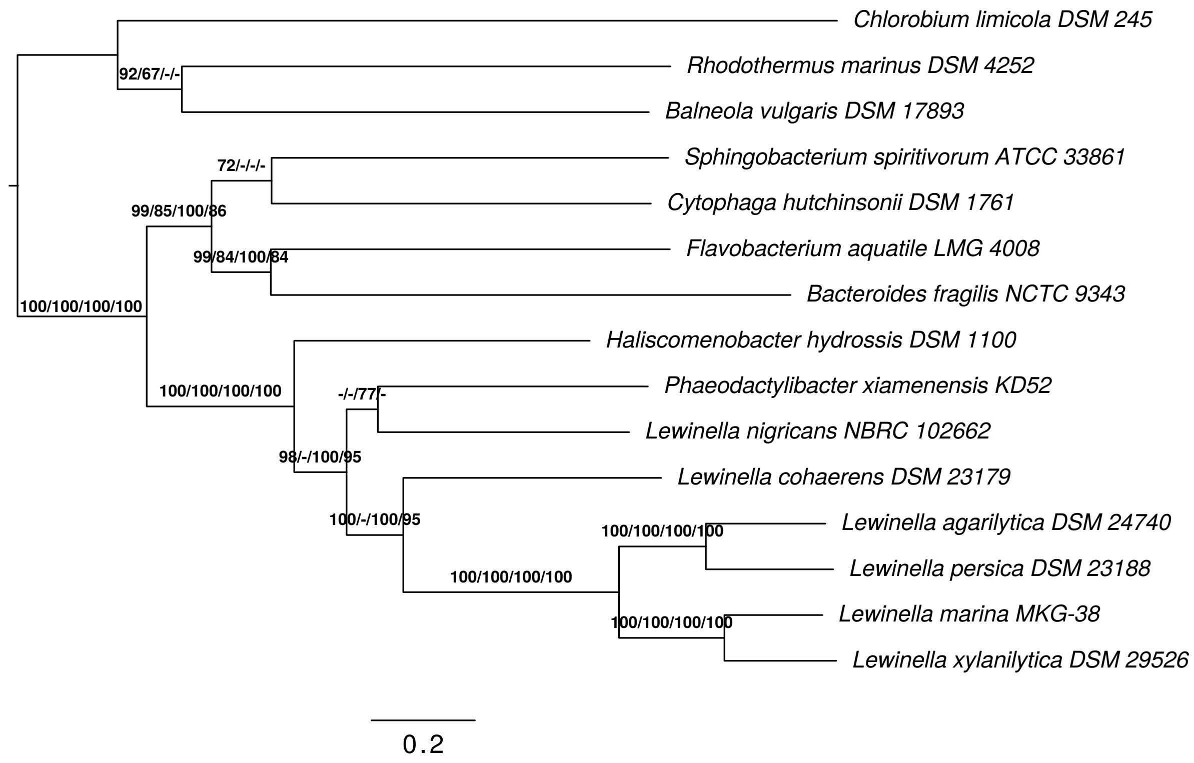

Figure 18: Phylogenetic ML tree with partition bootstrap support inferred with RAxML from the *Lewinella* supermatrix including all single-copy genes that occured in at least four of the genomes. Branches are scaled in terms of the expected number of changes per site. The first two numbers above branches (left to right) are partition bootstrap support values from (i) RAxML (ML) analysis and (ii) TNT (MP) analysis of the supermatrix that included single-copy genes that occurred in all of the genomes. The last two numbers above branches are partition bootstrap support values from (iii) RAxML (ML) analysis and (iv) TNT (MP) analysis of the supermatrix that included single-copy genes that occurred in at least four of the genomes.

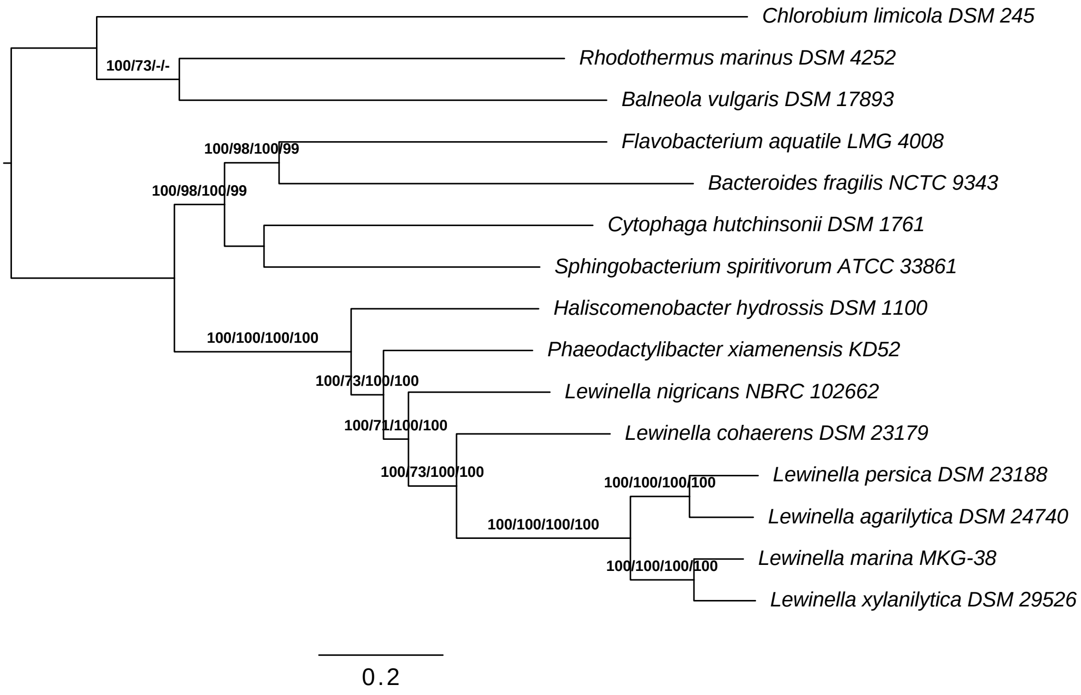

Figure 19: Phylogenetic ML tree with standard bootstrap support inferred with RAxML from the *Lewinella* supermatrix including all single-copy genes that occured in all of the genomes. Branches are scaled in terms of the expected number of changes per site. The first two numbers above branches (left to right) are standard bootstrap support values from (i) RAxML (ML) analysis and (ii) TNT (MP) analysis of the supermatrix that included single-copy genes that occurred in all of the genomes. The last two numbers above branches are standard bootstrap support values from (iii) RAxML (ML) analysis and (iv) TNT (MP) analysis of the supermatrix that included single-copy genes that occurred in at least four of the genomes.

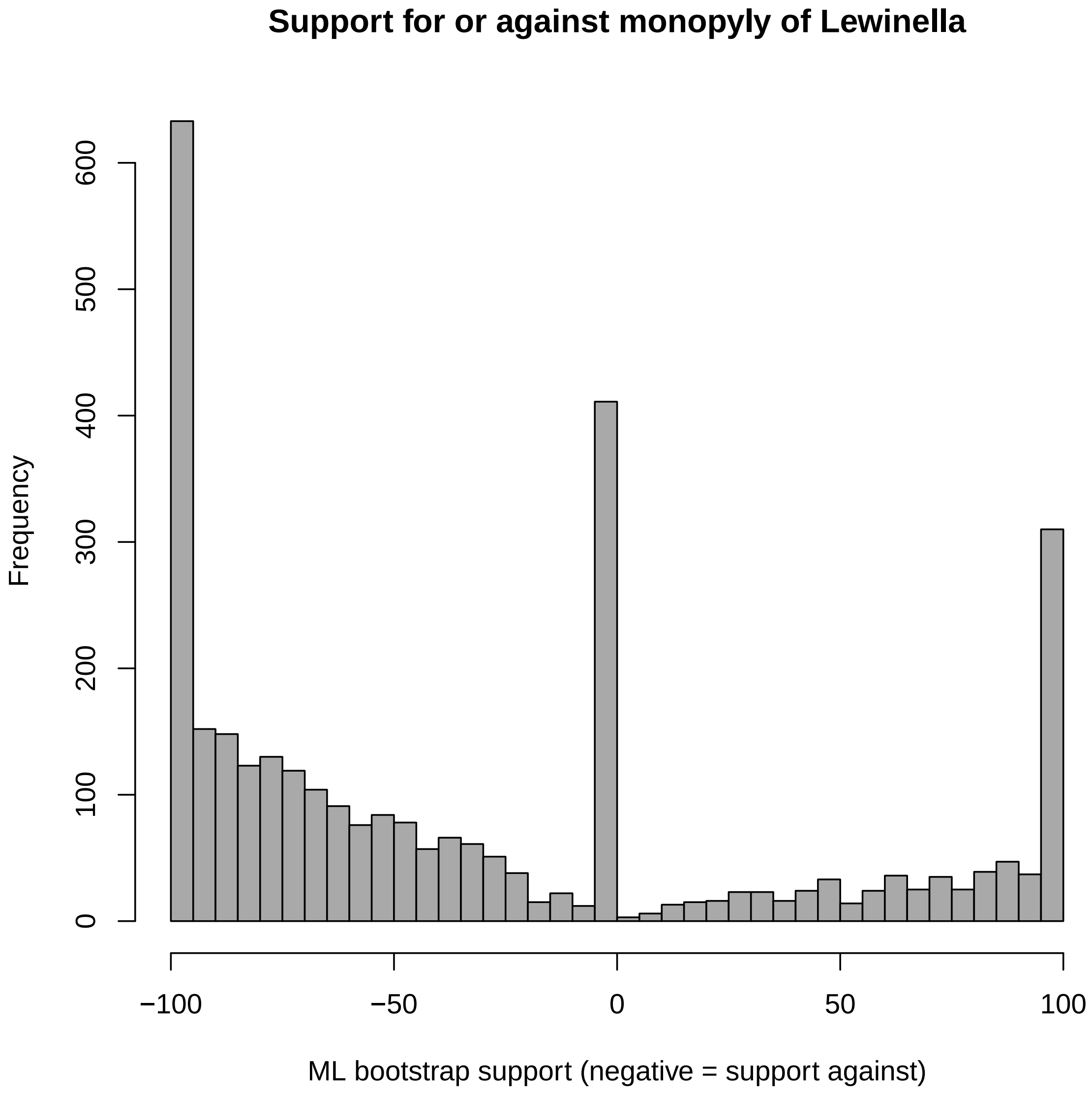

Figure 20: Histogram showing ML bootstrap support for or against monopyly of *Lewinella* in analyses of all single-copy genes that (a) occurred in at least for of those genomes considered in the last two figures and (b) were contained in at least two *Lewinella* genomes and at least one outgroup genome. In summary, there were roughly twice as many single genes (leftmost frequency bar) which displayed a support of $\geq$95% **against** Lewinella monophyly than single genes with a support $\geq$95% **for** Lewinella monophyly (rightmost frequency bar). As outgroup were tried, in turn, all (1) non-*Bacteroidetes*, (2) *Chlorobia*, (3) *Rhodothermia*, (4) *Balneolia* (5) *Bacteroidia*, (6) *Cytophagia*, (7) *Flavobacteriia*, (8) *Sphingobacteriia* and (9) *Haliscomenobacter* genomes as far as contained in the data set presented in the last two figures.
